# Promoter Complexity and Tissue-Specific Expression of Stress Response Components in *Mytilus galloprovincialis*, a Sessile Marine Invertebrate Species

**DOI:** 10.1371/journal.pcbi.1000847

**Published:** 2010-07-08

**Authors:** Chrysa Pantzartzi, Elena Drosopoulou, Minas Yiangou, Ignat Drozdov, Sophia Tsoka, Christos A. Ouzounis, Zacharias G. Scouras

**Affiliations:** 1 Department of Genetics, Development & Molecular Biology, School of Biology, Faculty of Sciences, Aristotle University of Thessaloniki, Thessaloniki, Greece; 2 Centre for Bioinformatics, School of Physical Sciences & Engineering, King's College London, London, United Kingdom; 3 BHF Centre of Research Excellence, Cardiovascular Division, School of Medicine, James Black Centre, Denmark Hill Campus, King's College London, London, United Kingdom; 4 Computational Genomics Unit, Institute of Agrobiotechnology, Centre for Research & Technology Hellas, Thessaloniki, Greece; University of California San Diego, United States of America

## Abstract

The mechanisms of stress tolerance in sessile animals, such as molluscs, can offer fundamental insights into the adaptation of organisms for a wide range of environmental challenges. One of the best studied processes at the molecular level relevant to stress tolerance is the heat shock response in the genus *Mytilus*. We focus on the upstream region of *Mytilus galloprovincialis* Hsp90 genes and their structural and functional associations, using comparative genomics and network inference. Sequence comparison of this region provides novel evidence that the transcription of Hsp90 is regulated via a dense region of transcription factor binding sites, also containing a region with similarity to the Gamera family of LINE-like repetitive sequences and a genus-specific element of unknown function. Furthermore, we infer a set of gene networks from tissue-specific expression data, and specifically extract an Hsp class-associated network, with 174 genes and 2,226 associations, exhibiting a complex pattern of expression across multiple tissue types. Our results (i) suggest that the heat shock response in the genus *Mytilus* is regulated by an unexpectedly complex upstream region, and (ii) provide new directions for the use of the heat shock process as a biosensor system for environmental monitoring.

## Author Summary

Adaptation of sessile animals, such as molluscs, to stress is achieved by a number of molecular mechanisms, few of which are clearly understood. Insights from this research can provide clues about stress tolerance both for sessile and mobile organisms. The Mediterranean mussel, of the genus *Mytilus*, is a model organism for the study of stress at the molecular level, with sufficient gene structure and function data available. We have thus investigated a key stress response gene, Hsp90, and in particular its upstream region, using a combination of sequence and expression analysis approaches. We demonstrate that this region, responsible for the regulation of heat shock-associated gene expression, exhibits an unparalleled structural and functional complexity compared to other model organisms, as well as subtle gene expression patterns across multiple tissues. These results form the basis upon which the heat shock response can be used as a molecular biosensor for environmental monitoring in the future.

## Introduction

The majority of molluscan species go through two principal developmental phases, a larval embryo (motile phase) followed by a clumping structure (sessile phase), when they are permanently attached to an underwater substrate. This lifecycle, common amongst marine invertebrates, poses challenges for adaptation and tolerance for a wide range of conditions at the littoral zone, including steep salinity or temperature gradients. Key model organisms for molluscan biology include species from the genus *Mytilus*, in particular *M. edulis*, *M. galloprovincialis* and *M. californianus*. Crucially, the latter species is a target organism for a genome sequencing project, whose results are eagerly expected by the community (http://www.jgi.doe.gov/sequencing/why/3090.html).

The *Mytilus* species group provides an ideal model both for fundamental questions of animal adaptation to stress response, as well as biotechnological applications, primarily as a pollution biosensor [1]. Its use extends into biomimetics [2], in particular protein-based medical adhesives [3], with potential applications in fields such as dentistry [4]. Moreover, its relatively complex developmental structure and higher taxonomic status as an invertebrate, combined with the fact that it can suffer from mussel haemic neoplasia, renders this organism a potential model for human leukemia and an ideal biomarker for pollution-induced disease [5]. In this context, it is important to understand the mechanisms by which mussels tolerate and cope with environmental stress, given that their behavioral options are highly restricted, due to the sessile phase of their lifecycle.

In the past, comparisons between motility and sessility for higher organisms have been primarily confined to animals versus plants [6], with follow-up studies focusing on comparisons between large animals, e.g. humans, versus large plants, e.g. trees, and the trade-offs for the tree body plan [7]. Less attention has been paid to adaptations by sessile animals, in particular intertidal invertebrates (or, “plant” equivalents) [8]–[9], and the molecular mechanisms through which they achieve tolerance to stress. One exception is represented by heat shock response, a key factor for temperature adaptation that has been studied in this context to a certain extent [8], and specifically in *Mytilus* with regard to the Hsp70 [10] and Hsp90 [11] genes.

Transcriptional regulation can be achieved either by an extensive repertoire of paralogs and transcription factors (‘gene content strategy’) or a complex structure of promoters (‘gene structure strategy’). Analysis of comprehensive datasets has clearly demonstrated that transcription factors (TFs) and transcription-associated proteins (TAPs) are not universally distributed but highly taxon-specific and that relative TF gene content increases with the taxonomic scale [12]–[13]. Such comparisons have been later extended by follow-up studies that analyzed TAP complements and their expansion rates in plants [14]–[15]. Thus, it is now known that one way by which plants, sessile organisms *par excellence*, achieve a finer degree of regulation is by the expansion of TF/TAP complements and a ‘gene content strategy’. Yet, it is unclear whether similar trends are followed in sessile animals, since entire genome sequences for those are lacking so far, limiting the range of comparative genome-wide studies that can be performed.

As far as paralogs are concerned, recent studies that have focused on the heat shock response in plants, and in particular *Arabidopsis thaliana*, have revealed that the process involves up to 21 known TFs and four heat shock protein (Hsp) families (Hsp20/70/90/100) [16]–[17]. Despite a cursory resemblance to mammals, in *Drosophila* thermal sensing is achieved by a unique repertoire of genes [18], including thermostat systems not exclusively involving heat shock proteins [19]. In other words, and probably for different reasons, a gene content strategy might prevail in both model organisms for plants (*A. thaliana*) and motile invertebrates (*Drosophila*). Thus, it is worth examining what are the mechanisms through which stress response is regulated in sessile marine invertebrates in general, and the *Mytilus* genus in particular, and which strategy dominates gene expression.

We focus on the Hsp90 family as a case study for stress response in sessile animals and examine the structure and function of the Hsp90 upstream region in *M. galloprovincialis*. Previously, two distinct Hsp90 genes with the same genomic organization have been isolated from *M. galloprovincialis* [11], herein called Mghsp90 genes. Detailed sequence analysis revealed that the two genes contain nine exons and exhibit great similarities in both the 5′ non-coding and the coding regions but differ in their 3′ non-coding regions, as well as in three introns, due to the presence of repeated sequences [11]. The 5′ non-coding region of both genes contains a non-translated exon and multiple binding sites for various transcription factors, highly suggestive of potential interactions of these factors with the Hsp90 promoter and subtle patterns of gene regulation [11].

A comparative analysis of Hsp90 gene content across all taxa with available sequence data has clearly shown that invertebrate genomes contain a relatively small number of Hsp90 genes (3–4 genes), compared to those of vertebrates (>5 genes) [20]. Thus, it appears that the *Mytilus* genome might contain a relatively small number of TFs (e.g. heat shock factors or HSFs – no such factors can be detected in the *Mytilus californianus* EST collection, not shown) and/or Hsp90 genes, raising the question how the expression of Hsp90 and other heat shock genes is regulated in sessile invertebrates.

In the present work, we perform a detailed analysis of the Mghsp90 upstream region in terms of structure and expression, and reveal the presence of previously undetected sequence elements of unknown function. Based on tissue-specific expression data, we also delineate the potential associations of Mghsp90 with another 174 genes that are involved in a complex pattern of expression across tissues. These two discoveries are discussed within the context of existing knowledge and are expected to contribute towards a deeper understanding of the heat shock response in sessile organisms.

## Results/Discussion

### Comparative analysis of the Mghsp90 upstream region reveals an unexpected complexity

**Figure 1:**
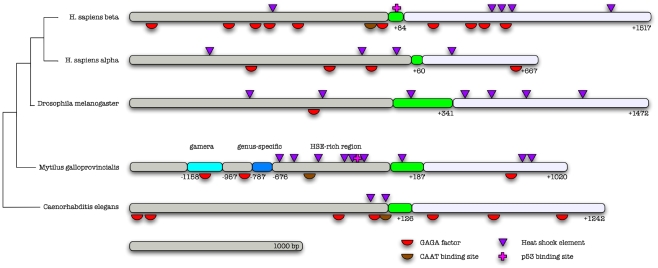
Comparative analysis of the *M. galloprovincialis* sequence 5′ region of Hsp90 genes and three animal species, including human. Dark grey bars correspond to the 5′ untranscribed region, green bars correspond to the first exon, and light grey bars to the first intron. Two regions found only in the *M. galloprovincialis* Hsp90 genes are marked (in blue). Numbering is relative to the transcription initiation site (first exon). Transcription factor binding sites and heat shock elements in different colors (bottom right) and scale (bottom left) are clearly shown. Relationships of the five genes are shown with a relative dendrogram (not drawn to scale).

The comparison of the 5′ upstream region of Mghsp90 genes to their homologs in two model organisms for which there is extensive genomic evidence and humans reveals an increase of complexity in TF binding sites including heat shock elements (HSEs, binding sites for HSFs – see **Methods**). The *M. galloprovincialis* Hsp90 region exhibits a peculiar degree of unexpected complexity with regard to its phylogenetic context, not only in terms of quantity of predicted elements but also in fine structure of the promoter (**Figure 1**). The *Mytilus* region contains more regulatory sites than the *D. melanogaster* region (namely, 14 sites *vs.* 8), a total count similar to that of the human Hsp90 beta gene (17 sites – **Figure 1**). Moreover, it is host to two newly identified elements (Gamera and a genus-specific sequence), both of unknown function (represented by blue bars, **Figure 1**), followed by a HSE-rich region with a CAAT binding site and a putative p53 binding site (see also below, and **Figure 1 in Protocol S1**).

### Detection of a LINE-like repetitive sequence in the Mghsp90 upstream region

**Figure 2:**
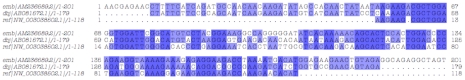
Alignment of the LINE-like upstream region in Mghsp90. Upstream sequenceis identical in both genes, Mghsp90-1 is shown only (AM236589.2), and similar to the LINE-like Gamera element in *O. curvinotus* (AB081572.1) and *S. mansoni* (NW_003038502.1). Identities in all sequences are represented as dark blue boxes and in two sequences in light blue boxes. Alignment visualized by JalView [91]. Sequence positions are relative to the alignment and do not correspond to those of the database entries.

Curiously, upstream of the first exon (1158 nucleotides) of Mghsp90, there exists a 201-base pair (bp) sequence element with a putative GAGA factor binding site (**Figure 1**), 69% identical over 181 nucleotides to the medaka fish *Oryzias curvinotus* LINE-like repetitive sequence Gamera [21]. The similarity extends over positions 1907–2085 of the *O. curvinotus* 4493-bp sequence entry (Genbank accession number AB081572, GI:19570857) and more specifically over the ‘open reading frame’ b (defined at positions 1353–3052) [21]. Thus, this region of approximately 200 nucleotides is only a fraction of the putative ORF b and, to our knowledge, it is the first time this segment is reported outside the *Oryzias* genus and its closest relatives [21] (**Figure 2**). Moreover, multiple copies of this region can also be identified in the genome of the blood fluke *Schistosoma mansoni* [22] (**Figure 2**, **Figure 2.1 in Protocol S1**). Fragments of this sequence are also present in (i) the Expressed Sequence Tag (EST) database, more specifically in the neural transcriptome and thus genome of the gastropod *Aplysia californica* [23], the termite *Hodotermopsis sjoestedti* [24], the African cichlid fish *Oreochromis niloticus* (Lee et al., unpublished, GI: 253867024), the mollusc *Lymnaea stagnalis* [25] and the sea anemone *Nematostella vectensis* [26] (in that order of sequence similarity – **Figure 2.2 in Protocol S1**); (ii) the unfinished high-throughput genomic sequence database (**Figure 2.3 in Protocol S1**), in the genome of sea urchin *Strongylocentrotus purpuratus* [27] and (iii) the Whole-Genome-Shotgun Sequence database (**Figure 2.4 in Protocol S1**) in the genome of the hemichordate *Saccoglossus kowalevskii* (unpublished).

The functional significance of this element is not clear, yet given that the region can be identified in at least ten – highly unrelated and primarily aquatic – species, the presence of a transposable element of a highly mobile nature (or its evolutionary relic) is indicated (**Figure 2**). In *M. galloprovincialis*, it has also been shown that mobile elements reside within introns of the Hsp70 genes [10], however there is no detectable sequence similarity between those elements and the Mghsp90 Gamera-like sequence presented here.

### A conserved genus-specific sequence of unknown function

**Figure 3:**
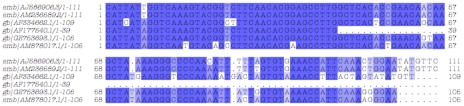
Alignment of the genus-specific upstream region in Mghsp90. Mghsp90-1/2 genes (AM236589.2 / AJ586906.3 respectively) are similar to the *M. edulis* lysozyme gene (AF334662.1), the *M. galloprovincialis* mytilin B precursor gene (AF177540.1) and two cDNAs from *M. californianus* and *M. edulis* (GE753693.1 / AM878017.1, respectively) Identities in all sequences are represented as dark blue boxes, other identity blocks in light blue boxes. Alignment visualization and sequence positions as in **Figure 2**.

Another feature of the Mghsp90 upstream region is a genus-specific sequence, approximately 100-bp long, located 787 positions before the first exon of Mghsp90 genes (**Figure 1**). This region is much more phylogenetically restricted than the Gamera element, found only in the genus *Mytilus*, namely the *M. galloprovincialis* mytilin B precursor gene [28]–[29] – (accession number: AF177540.1, positions 777–815 antisense strand, non-coding region), a lysozyme gene (AF334662.1, positions 1016–1050 sense strand, second intron) [30] and a cDNA (AM878017.1) both from *M. edulis*, and a cDNA sequence from *M. californianus* (GE753693.1) (**Figure 3**). This genus-specific sequence does not contain any transcription factor binding sites (**Figure 1**), thus its functional significance is not known at present.

It is worth noting that similarly to the mytilin B gene, another antimicrobial peptide gene, the *M. galloprovincialis* defensin 2 (MGD2) gene, contains a 160-bp long element with similarities to the *M. edulis* lysozyme gene (fourth intron), two glycosidase gene introns (endo-1,4-beta-D-glucanase – AJ308548.1, 2^nd^ intron; endo-1,4-mannanase – AJ271365.2, 5^th^ intron), the 3′-UTR of the *M. galloprovincialis* Hsp70-1 gene, all being similar to an ISSR sequence (AJ938114), indicating the presence of a transposable element [31]. The above mentioned genes all have catabolic roles and might indeed be connected to defense mechanisms, broadly associated with stress. Further study is required in order to understand the role of these genus-specific sequences in the molecular physiology of the above mentioned loci.

### The putative p53 binding site and the molluscan neoplasia connection

A putative binding site for p53 is located between two HSEs in the 5′ regulatory region of the Mghsp90 genes [11] (**Figure 1**), being identical to the consensus binding site of human p53 to retinoblastoma susceptibility gene [32]. This binding site is evidently absent from other species, including *C. elegans* and *D. melanogaster*, but present in the human Hsp90 beta gene [33] (**Figure 1**). The p53 proteins from two *Mytilus* species exhibit very high similarity to their human homologs, and especially in the DNA binding domain, the transcriptional activation domain (TAD) and the nuclear localization signal. In addition, residues mutated in various human cancers are also conserved in the *Mytilus* p53 proteins [34]. It should be noted that p53 is phylogenetically restricted to animals while the molluscan versions (Decapodiformes, Bivalvia and *Haliotis* sp.) exhibit a very high similarity to the vertebrate sequences (not shown). The prediction of the p53 binding site in *Mytilus* is based on the known association of p53 with the upstream region of the human Hsp90 beta gene [33], the conservation of the *Mytilus* p53 genes [34] and the observation that an identical site is present in human Hsp90 (an Mghsp90 homolog) [11].

In order to further establish the validity of the predicted p53 binding site in a phylogenetic context, we have searched the non-redundant nucleotide database with the Mghsp90 genes as queries (see **Methods**). We subsequently identified 215 homologous target regions, with the closest sequence-similar entries carefully selected to exclude cDNA clones or partial coding sequences, across a wide taxonomic spectrum (**Figure 1 in Protocol S1**). These sequences were scanned for putative p53 binding sites (732 matches in total, see **Methods**), conditioned on the p53 phylogenetic distribution mentioned above; in other words, sites found in organisms known to encode for p53 were considered as positive cases (727 in total), while the remainder were treated as negative cases (5 in total). Despite well-understood limitations, e.g. the under-representation of certain species in terms of comparable Hsp90 sequence data and the over-representation of others in terms of redundant sequences, it is evident that p53-containing species exhibit a high number of predicted p53 binding sites (primarily chordates), while other organisms (such as fungi or plants), present a sporadic pattern of false positive hits, as expected. The exception in this otherwise consistent picture is the molluscs (Bivalvia and *Haliotis* sp.), having a small number of predicted p53 binding sites (**Figure 1 in Protocol S1**). The shortage of sequence information for molluscs, coupled with a possibly non-canonical sequence motif, leaves the question open for the unambiguous detection and experimental confirmation of the elusive molluscan p53 binding site.

The presence of a putative p53 binding site in the promoter region of the *Mytilus* Hsp90 genes raises questions about the possible implication of Hsp90 proteins in molluscan leukemia. Very recent studies on the association of p53 with heat shock response [35], the differential expression of p53 in mussel haemic neoplasia [5], and the impact of pollutants on p53 expression [36] underline the potential involvement of p53 in both heat shock response and neoplasia and its irregular similarity to vertebrate homologs [37], as well as its potential use as a marker for environmental monitoring [34]. In other species, namely soft-shell clams, certain results also indicate that environmentally induced alterations in p53 might contribute to leukemia [38]–[39].

Indeed, expression studies have established that Hsp genes and a p53-like gene are abundant in *M. galloprovincialis* [40], especially in pollutant exposed mussels [41], now searchable through the Mytibase resource [42]. Moreover, there is evidence from proteomics studies that Hsp proteins are expressed in stress conditions and can potentially be used as pollution biomarkers [43]–[44] or temperature biosensor [45].

### Differential gene expression analysis consistent with tissue specificity

**Figure 4:**
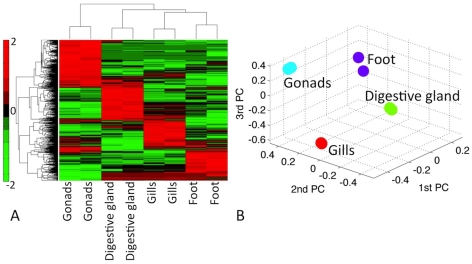
Differential gene expression patterns of tissue samples from *M. galloprovincialis* under normal conditions. (A) A two-way clustering identifies 547 differentially expressed genes across two replicates over four normal tissue types. Low-high gene expression values are represented by a green-red color scale; (B) Principal Component Analysis (PCA) of the data further confirms the inter-replicate reproducibility and tissue specificity. In this representation the 1^st^/2^nd^/3^rd^ principal components (PC) are mapped onto x-, y-, and z- axis respectively.

In order to investigate co-expression patterns for Mghsp90 genes, we have extracted tissue-specific gene expression data available in Mytibase, encompassing 3840 cDNA sequences [42]. Following normalization (see **Methods**), we detected 547 genes (14% of total, in the ‘original’ network) that are differentially expressed across all four tissue types under investigation (namely gills, gonads, foot and digestive gland – **Figure 4**).

A two-way clustering across genes and tissues confirms that the four tissue types can be accurately detected (**Figure 4A**). This step also suggests that the 547 differentially expressed genes can be clustered into four distinct classes corresponding to the four tissues, with relatively low overlap (**Figure 4A**). A Principal Component Analysis of the original network further confirms the inter-replicate reproducibility and tissue specificity, indicating the high quality and consistency of the initial gene expression data (**Figure 4B**).

### Co-expression analysis through network clustering across tissues

**Figure 5:**
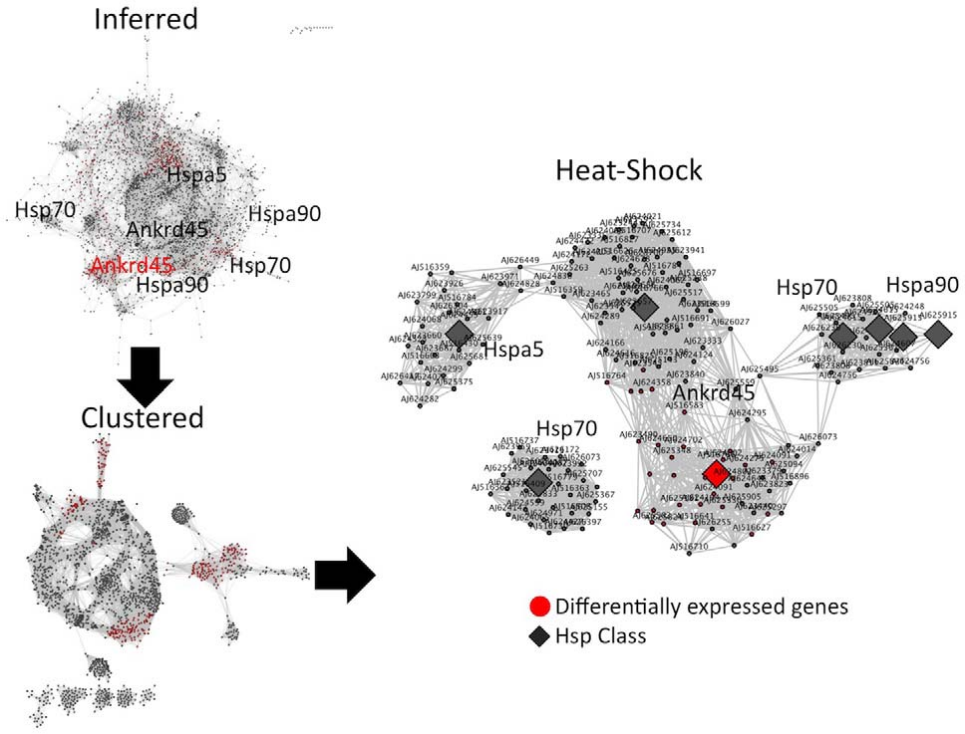
Composite cross-tissue networks of *M. galloprovincialis* co-expression and Hsp gene class associations. These networks are annotated as ‘differentially expressed genes’ (red) and ‘Hsp gene class’ (grey) (see text and **Methods**). The gene names for the 4 unique Hsp class members are shown, corresponding to 8 individual cDNA clones.

To infer gene associations via co-expression profiles, PCCs (see **Methods**) were computed for all possible pair-wise gene permutations of the original network. High PCC values correspond to a large similarity in expression profiles across four tissue types. Only those gene pairs with PCC>0.90 were further considered. This step yielded a global co-expression network, defined as the ‘inferred’ network, containing 3692 nodes and 57697 edges (**Figure 5**). The inferred network represents 96% of all cDNA clones in the original network. Such high coverage may be explained by the limited number of experimental replicates provided in the dataset.

To ensure that only significant associations are considered, MCL clustering (see **Methods**) was performed to produce a ‘clustered’ network with 1719 nodes and 43286 associations (**Table 1**). The clustered network represents a subset of the inferred network enriched with the most highly connected genes with the strongest co-expression values (**Figure 5**). Interestingly, of the 547 differentially expressed genes obtained initially, 271 (∼50%) are present in the clustered network, signifying a sufficient coverage of differential expression. This enriched network thus maintains 75% (43286/57697) of network edges, from which more reliable associations can then be extracted.

**Table 1:** Statistical information for co-expression networks.

Network	Nodes	Edges	Connectivity (avg)	Connectivity (max)	Degree (avg)	Out degree (max)	In degree (max)
Inferred	3692	57697	31.3	120	17.2	118	115
Clustered	1719	43286	50.4	119	25.9	116	113
Hsp	174	2226	25.6	56	13.3	47	44

### Associations of the Hsp class of genes

To delineate the involvement of the wider Hsp class of genes in normal *M. galloprovincialis* tissue, 8 cDNA sequences corresponding to 4 distinct *Mytilus* Hsp genes, labeled as Hspa5 (Grp78 homolog), Hsp70, Hspa90 (Mghsp90), and Ankrd45 (similar to heat shock 70 KD protein C precursor) were identified in the clustered network (**Figure 5**). The “Ankrd45”-like sequence (e.g. XP_290882.1) warrants description: its N-terminal part contains ankyrin repeats most similar to the ankyrin repeat domain of the human p53 binding protein [46], while its C-terminal part is similar to Grp78, a homolog of Hsp70 (**Figure 3 in Protocol S1**). Structural evidence indicates that the ankyrin repeats of p53 binding proteins (53BP2) bind to the L2 loop of p53 [47], implicating a configuration of ankyrin repeats such as the one found in Ankrd45, in a potentially mediated p53-Hsp70 domain interaction.

In fact, since the initial discovery that the Hsp70 promoter is regulated by p53 [48], there is mounting evidence that these two proteins are involved in various processes, including oral dysplasia [49], endometrial carcinomas [50], gastric cancers [51], ischemia [52] and wound healing [53]. These interactions have been reviewed elsewhere [54]–[55]. Similarly, it has been demonstrated that p53 requires the activity of Hsp90s [56] and the structural [57] and biochemical [58] basis of this interaction has been deciphered. In fact, it appears that p53, Hsp70 and Hsp90 are involved in a complex interplay during carcinogenesis [59].

To examine Hsp-related associations in greater detail, the nearest-neighbor members of 8 Hsp cDNA clones were selected, defined as the Hsp network (**Figure 5**). This network contained 174 genes and 2226 associations, accounting for 4.5% of genes in the original network (**Tables 1 and 2 in Protocol S1** – node labels refer to MyArray1.0 identifiers, see **Methods**). The Hsp network contains clones with similarity to perlucin (a biomineralization-associated protein) [60] and the *M. edulis* polyphenolic adhesive protein [61], among others (**Table 1 in Protocol S1**); it is curious that in this set, there is also a clone highly similar to the *M. edulis* gene for endo-1,4-mannanase, discussed above.

**Figure 6:**
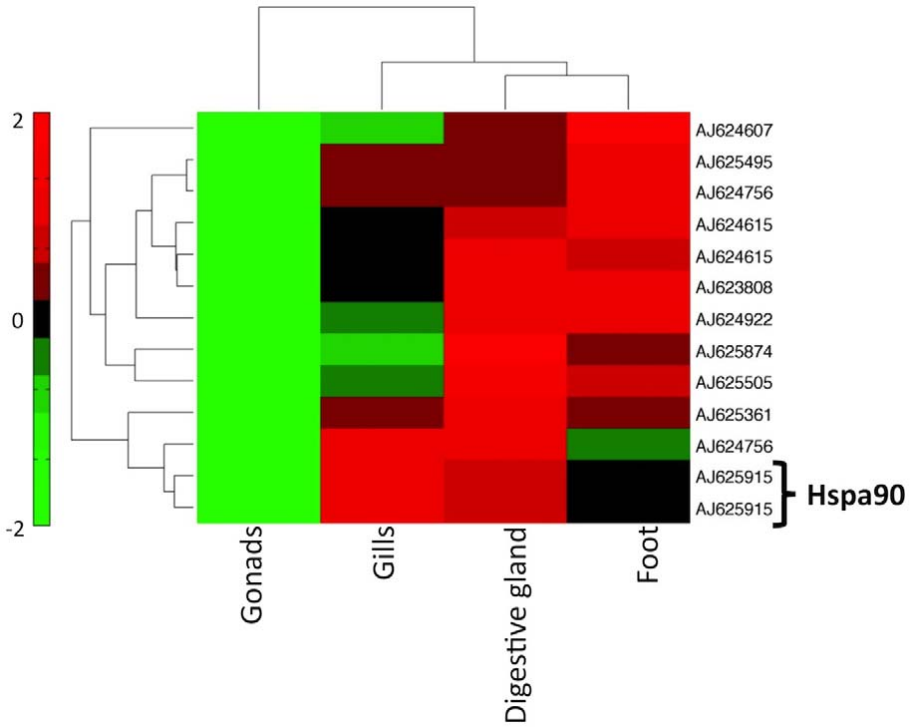
Hierarchical clustering of 10 unique probes corresponding to genes co-expressed with Hspa90. Expression of Hspa90 appears to be elevated in gills and digestive gland and down-regulated in gonads. The change in expression is not statistically significant (p>0.05).

Remarkably, 30/547 (5.5%) of differentially expressed genes are found to be co-expressed with the Ankrd45 clone. This suggests that members of the Hsp class are involved in complex transcription patterns across multiple tissue types rather than a single one. Indeed, the closest co-expression neighbors of Mghsp90 are two cDNAs for calreticulin – a calcium-binding chaperone (AJ624756/AJ625361) known to be associated with Hsp proteins [62] (**Figure 6**). Given the high-quality, yet limited data, the gene expression analysis outlined here strongly indicates that the known Hsp-associated genes in *Mytilus* are involved in intricate ways with each other, are possibly controlled by a small number of TFs over a number of tissues and conditions. It is thus possible that a mechanism for heat response might involve a ‘gene structure’ strategy, with few genes involved in a multitude of gene expression pathways.

### Conclusion and future perspectives

In this study, we have dissected computationally the upstream region of the Mghsp90 genes to investigate its structure and function. The structural complexity of this region strongly suggests that the transcription of Hsp90 stress response is tightly regulated via a dense region of heat shock elements and other regions of varying phylogenetic dispersion (**Figure 1**). Compared to other model organisms, such as *C. elegans* and *D. melanogaster*, this regulation appears to be achieved through a ‘gene structure’ strategy, i.e. a complex gene structure. In addition, expression analysis of the heat shock response indicates that a handful of key molecules belonging to the heat-shock class, exhibit a differential tissue-specific expression profile, possibly in gills and the digestive gland, while at the same time maintaining a multitude of associations through a complex co-expression network (**Figure 5**). Our results are consistent with current knowledge about chaperone function both within molecular [63]–[64] and ecological contexts [65]–[66], and demonstrate the efficacy of both comparative genomics and systems biology for the elucidation of complex relationships between genotype, environment and phenotype.

The nature of sessile animals, with the *Mytilus* genus as a model organism, can shed light into their metabolic capabilities [67], stress responses [68] and resilience of evolutionary extinction [69]. The stress response for sessile animals is of particular interest, especially in cases where different ecological niches can be compared for close relatives, e.g. different growth potential in varying hydrostatic pressure or temperature [70]. Heat shock proteins in particular are used as indicators of thermal stress [68]; for instance, in the case of marine snails (*Tegula* genus), the time course and magnitude of the heat shock response was measured in a field study by monitoring the synthesis of heat shock proteins [71]. In another field study on the Oregon coast, *M. californianus* and its predator *Pisaster ochraceus* were examined for production of the Hsp70 heat shock proteins; it was found that while mussels (a sessile species) have an increased production of Hsp70, its starfish predators (a mobile species) do not, potentially exhibiting decreased heat shock adaptation compared to their prey [72]. Sessile marine invertebrates have been studied in the context of rising sea temperatures, including *M. edulis* [73] and *Rhopaloeides odorabile*, a common Great Barrier Reef sponge [74].

In the future, the thermal ecology of stress response can potentially inform policy decisions for environmental management in the context of climate change [75] – including the analysis of biogeographical range shifts [76], particularly important for sessile animals, the understanding of complex prey-predator interactions e.g. the above mentioned pair of *P. ochraceus* and *M. californianus* [77], and instigate a more integrated approach that will eventually include both weather records and niche-level measurements [78]. Currently, more established approaches for the use of *Mytilus* relate to its use as a biosensor system for the environmental monitoring of coastal water pollution [79], heavy metals or organic pollutants [80] – including manufactured substances such as fiberglass [81]. In conclusion, this work forms a basis upon which the stress response in *Mytilus* will be better understood at the molecular level.

## Methods

### Sequence comparison

The *M. galloprovincialis* Hsp90 sequence was analyzed as previously [11]. The Hsp90 upstream regions from three other representative animal species, namely *Caenorhabditis elegans*, *Drosophila melanogaster* and *Homo sapiens*, were analyzed in a similar fashion. Previously published data concerning Hsp90 genes from these species were also taken into consideration for annotation purposes [82]–[88]. Sequence database searches were performed by BLAST (for nucleotide sequences, blastn, version 2.2.22) [89]. Sequence alignments were computed using ClustalW [90] and visualized by JalView [91].

### Promoter analysis

Regulatory elements, in the 5′ non-coding regions of the Mghsp90 genes were identified with Alibaba2 [92], P-MATCH [93] and the Transcription Element Search System (TESS) [94]. An extensive comparative analysis for p53 binding sites was performed using the Matrix Search analysis tool of the TRED database [95], scanning query sequences against the p53-specific sequence matrix (cut-off score 2) from the JASPAR collection [96].

### Expression analysis

Data were obtained from the Gene Expression Omnibus (GEO) database, representing one spotted cDNA array (Accession number: GSE2176) for gene expression in normal mussels (*M. galloprovincialis*), using the MyArray 1.0 platform targeting 1712 clones with a total of 3840 cDNA sequences [42]. This dataset encompasses the total RNA isolated from gills (n = 2), gonads (n = 2), foot (n = 2), and digestive gland (n = 2) [42]. Data normalization was performed by taking the binary logarithm (log_2_) of normalized intensities (defined as test signal/reference signal). Normalized data (‘original’ network) was subsequently subjected to statistical validation.

### Network modeling

Gene associations were identified by computing the Pearson Correlation Coefficient (PCC) for all gene pairs in the raw network. Gene pairs with positive correlations indicated by a PCC>0.90 were considered to be co-expressed. Co-expression patterns were represented as networks where each node corresponds to a unique gene and each edge represents a co-expression association. The final network (‘inferred’ network) was clustered using the Markov Clustering Algorithm (MCL) in order to both filter noisy associations and identify biologically meaningful clusters (‘clustered’ network), as previously described [97]. The inflation parameter for MCL was set to 3.0. Only clusters with >10 genes were further analyzed for biologically meaningful associations.

### Statistical analysis

Differential expression analysis was performed by applying Analysis of Variance (ANOVA) to all genes across four distinct tissue types. Only those genes with the overall p-value bellow 0.05 were considered as differentially expressed. Two-way unsupervised hierarchical clustering of differentially expressed gene signals was performed using Euclidean distance as a similarity measure. Principal component analysis (PCA) was also performed to confirm the validity of the analysis for the four tissue-specific datasets. All statistical analyses were performed with MATLAB (The MathWorks, Natick, MA – www.mathworks.com).

## Supporting Information

Protocol S1 18 Supplement files plus an index file: 3 Supplementary figures, 2 Supplementary tables - referenced in text as Protocol S1; index provided with an explanation of the directory contents.
(5.18 MB ZIP)
