# Supplementary material for: Promoter Complexity and Tissue-Specific Expression of Stress Response Components in Mytilus galloprovincialis, a Sessile Marine Invertebrate Species
Source: PLoS Comput Biol. 2010 Jul 8;6(7):e1000847. doi: 10.1371/journal.pcbi.1000847 (PMC2900285; doi:10.1371/journal.pcbi.1000847)
Supplement: Protocol S1 — 18 Supplement files plus an index file: 3 Supplementary figures, 2 Supplementary tables - referenced in text as Protocol S1; index provided with an explanation of the directory contents. (5.18 MB ZIP) [file pcbi.1000847.s001.zip › SUPPLEMENTS18/SupplementIndex.rtf]

INDEXFigure 1 TRED p53 sitesFigure 2.1 gamera vs nra: webarchive / pdfb: fastac: alignmentFigure 2.2 gamera vs estsa: webarchive / pdfb: fastac: alignmentd: fasta file for cFigure 2.3 gamera vs htgsa: webarchive / pdfFigure 2.4 gamera vs wgsa: webarchive / pdfFigure 3 Ankrd45a: webarchive / pdfTable 1174 heat shock network genes (excel format)Table 22226 heat shock network associations
