# Supplementary material for: Promoter Complexity and Tissue-Specific Expression of Stress Response Components in Mytilus galloprovincialis, a Sessile Marine Invertebrate Species
Source: PLoS Comput Biol. 2010 Jul 8;6(7):e1000847. doi: 10.1371/journal.pcbi.1000847 (PMC2900285; doi:10.1371/journal.pcbi.1000847)
Supplement: Protocol S1 — 18 Supplement files plus an index file: 3 Supplementary figures, 2 Supplementary tables - referenced in text as Protocol S1; index provided with an explanation of the directory contents. (5.18 MB ZIP) [file pcbi.1000847.s001.zip › SUPPLEMENTS18/SupplFigure2.1a.pdf]

BLAST Basic Local Alignment Search Tool

-

Edit and Resubmit Save Search Strategies Formatting options Download

Nucleotide Sequence (328 letters)

Results for: lcl|15647 None(201bp)

Your BLAST job specified more than one input sequence. This box lets you choose which input sequence to show BLAST results for.

Query ID
lcl|15647
Description
None
Molecule type
nucleic acid
Query Length
201

Database Name
nr
Description
All GenBank+EMBL+DDBJ+PDB sequences (but no EST, STS, GSS,environmental samples or phase 0, 1 or 2 HTGS sequences)
Program
BLASTN 2.2.22+ Citation

Reference
Stephen F. Altschul, Thomas L. Madden, Alejandro A. Schäffer, Jinghui Zhang, Zheng Zhang, Webb Miller, and David J. Lipman (1997), "Gapped BLAST and PSI-BLAST: a new generation of protein database search programs", Nucleic Acids Res. 25:3389-3402.
Other reports: Search Summary [Taxonomy reports] [Distance tree of results]

Search Parameters

Program	blastn
Word size	7
Expect value	10
Hitlist size	100
Match/Mismatch scores	2,-3
Gapcosts	5,2
Low Complexity Filter	Yes
Filter string	L;m;
Genetic Code	1

Database

Posted date	Oct 16, 2009 5:42 PM
Number of letters	29,200,301,913
Number of sequences	10,124,621
Entrez query	none

Karlin-Altschul statistics

Params	Ungapped	Gapped
Lambda	0.633731	0.625
K	0.408146	0.41
H	0.912438	0.78

Results Statistics

Length adjustment	34
Effective length of query	167
Effective length of database	28856064799
Effective search space	4818962821433
Effective search space used	4818962821433

Graphic Summary

Distribution of 223 Blast Hits on the Query Sequence

[?]

An overview of the database sequences aligned to the query sequence is shown. The score of each alignment is indicated by one of five different colors, which divides the range of scores into five groups. Multiple alignments on the same database sequence are connected by a striped line. Mousing over a hit sequence causes the definition and score to be shown in the window at the top, clicking on a hit sequence takes the user to the associated alignments. New: This graphic is an overview of database sequences aligned to the query sequence. Alignments are color-coded by score, within one of five score ranges. Multiple alignments on the same database sequence are connected by a dashed line. Mousing over an alignment shows the alignment definition and score in the box at the top. Clicking an alignment displays the alignment detail.

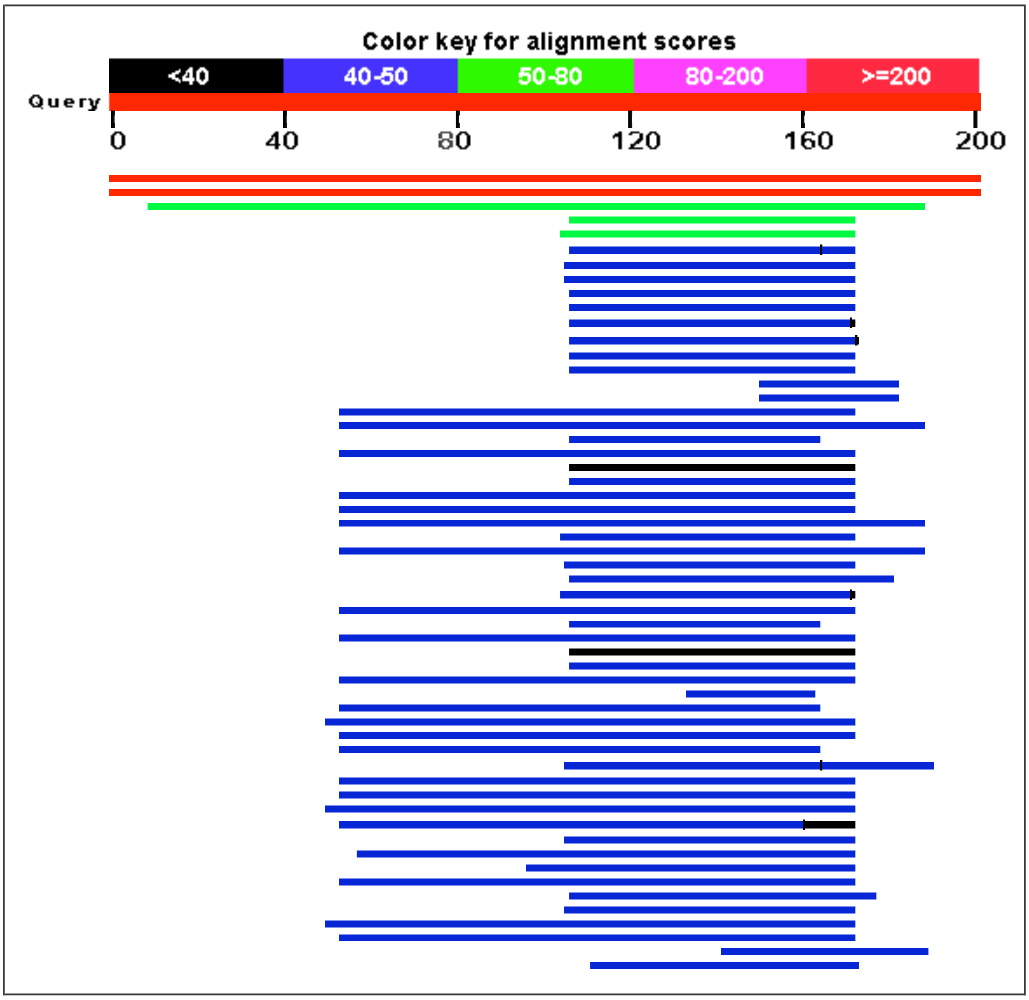

Descriptions

Legend for links to other resources: U UniGene E GEO G Gene S Structure M Map Viewer

Sequences producing significant alignments:
(Click headers to sort columns)

**AJ586906.3**	Mytilus galloprovincialis hsp90-2 gene for heat shock protein 90	363	363	100%	2e-97	100%
**AM236589.2**	Mytilus galloprovincialis hsp90-1 gene for heat shock protein 90, exons 1-9	363	363	100%	2e-97	100%
**AB081572.1**	Oryzias curvinotus DNA, LINE-like repetitive sequence Gamera, clone:Gamera-cur1	69.8	69.8	89%	5e-09	69%
**FN357346.1**	Schistosoma mansoni genome sequence supercontig Smp_scaff000055	57.2	57.2	32%	3e-05	78%
**FN357536.1**	Schistosoma mansoni genome sequence supercontig Smp_scaff000245	51.8	94.5	33%	0.001	77%
**FN357353.1**	Schistosoma mansoni genome sequence supercontig Smp_scaff000062	50.0	131	32%	0.004	81%
**FN357327.1**	Schistosoma mansoni genome sequence supercontig Smp_scaff000036	50.0	375	33%	0.004	77%
**FN357292.1**	Schistosoma mansoni genome sequence supercontig Smp_scaff000001	50.0	543	33%	0.004	87%
**NW_003027857.1**	Schistosoma mansoni genome sequence supercontig Smp_scaff014004	48.2	48.2	32%	0.015	75%
**FN357526.1**	Schistosoma mansoni genome sequence supercontig Smp_scaff000235	48.2	169	32%	0.015	75%
**FN357417.1**	Schistosoma mansoni genome sequence supercontig Smp_scaff000126	48.2	126	32%	0.015	78%
**FN357365.1**	Schistosoma mansoni genome sequence supercontig Smp_scaff000074	48.2	167	33%	0.015	87%
**FN357363.1**	Schistosoma mansoni genome sequence supercontig Smp_scaff000072	48.2	243	32%	0.015	75%
**FN371295.1**	Schistosoma mansoni genome sequence supercontig Smp_scaff014004	48.2	48.2	32%	0.015	75%
**AC192734.2**	Pan troglodytes BAC clone CH251-397M3 from chromosome 7, complete sequence	48.2	48.2	15%	0.015	93%
**AC147078.2**	Pan troglodytes BAC clone RP43-180L22 from chromosome 7, complete sequence	48.2	48.2	15%	0.015	93%
**NW_003038502.1**	Schistosoma mansoni genome sequence supercontig Smp_scaff001979	46.4	46.4	59%	0.053	69%
**NW_003038472.1**	Schistosoma mansoni genome sequence supercontig Smp_scaff001935	46.4	46.4	67%	0.053	69%
**NW_003035682.1**	Schistosoma mansoni genome sequence supercontig Smp_scaff001942	46.4	46.4	28%	0.053	77%
**NW_003033283.1**	Schistosoma mansoni genome sequence supercontig Smp_scaff003881	46.4	46.4	59%	0.053	69%
**NW_003026471.1**	Schistosoma mansoni genome sequence supercontig Smp_scaff000224	46.4	124	32%	0.053	85%
**NW_003025039.1**	Schistosoma mansoni genome sequence supercontig Smp_scaff009302	46.4	46.4	32%	0.053	77%
**NW_003022345.1**	Schistosoma mansoni genome sequence supercontig Smp_scaff013269	46.4	46.4	59%	0.053	71%

**FN359270.1**	Schistosoma mansoni genome sequence supercontig Smp_scaff001979	46.4	46.4	59%	0.053	69%	
**FN359226.1**	Schistosoma mansoni genome sequence supercontig Smp_scaff001935	46.4	46.4	67%	0.053	69%	
**FN357618.1**	Schistosoma mansoni genome sequence supercontig Smp_scaff000327	46.4	124	33%	0.053	85%	
**FN357592.1**	Schistosoma mansoni genome sequence supercontig Smp_scaff000301	46.4	46.4	67%	0.053	69%	
**FN357351.1**	Schistosoma mansoni genome sequence supercontig Smp_scaff000060	46.4	325	33%	0.053	78%	
**FN357335.1**	Schistosoma mansoni genome sequence supercontig Smp_scaff000044	46.4	163	37%	0.053	73%	
**FN357330.1**	Schistosoma mansoni genome sequence supercontig Smp_scaff000039	46.4	409	33%	0.053	80%	
**FN357299.1**	Schistosoma mansoni genome sequence supercontig Smp_scaff000008	46.4	333	59%	0.053	77%	
**FN359233.1**	Schistosoma mansoni genome sequence supercontig Smp_scaff001942	46.4	46.4	28%	0.053	77%	
**FN361172.1**	Schistosoma mansoni genome sequence supercontig Smp_scaff003881	46.4	46.4	59%	0.053	69%	
**FN357515.1**	Schistosoma mansoni genome sequence supercontig Smp_scaff000224	46.4	124	32%	0.053	85%	
**FN366593.1**	Schistosoma mansoni genome sequence supercontig Smp_scaff009302	46.4	46.4	32%	0.053	77%	
**FN370560.1**	Schistosoma mansoni genome sequence supercontig Smp_scaff013269	46.4	46.4	59%	0.053	71%	
**XM_001317411.1**	Trichomonas vaginalis G3 AT hook motif family protein (TVAG_474020) partial mRNA	46.4	46.4	14%	0.053	93%	G
**NW_003039011.1**	Schistosoma mansoni genome sequence supercontig Smp_scaff010546	44.6	44.6	55%	0.18	69%	
**NW_003033823.1**	Schistosoma mansoni genome sequence supercontig Smp_scaff010714	44.6	44.6	60%	0.18	69%	
**NW_003026315.1**	Schistosoma mansoni genome sequence supercontig Smp_scaff002909	44.6	44.6	59%	0.18	68%	
**FN367837.1**	Schistosoma mansoni genome sequence supercontig Smp_scaff010546	44.6	44.6	55%	0.18	69%	
**FN357707.1**	Schistosoma mansoni genome sequence supercontig Smp_scaff000416	44.6	128	42%	0.18	76%	
**FN357689.1**	Schistosoma mansoni genome sequence supercontig Smp_scaff000398	44.6	44.6	59%	0.18	68%	
**FN357447.1**	Schistosoma mansoni genome sequence supercontig Smp_scaff000156	44.6	83.7	59%	0.18	68%	
**FN357444.1**	Schistosoma mansoni genome sequence supercontig Smp_scaff000153	44.6	85.5	60%	0.18	73%	
**FN357435.1**	Schistosoma mansoni genome sequence supercontig Smp_scaff000144	44.6	240	59%	0.18	72%	
**FN357421.1**	Schistosoma mansoni genome sequence supercontig Smp_scaff000130	44.6	247	33%	0.18	74%	
**FN357350.1**	Schistosoma mansoni genome	44.6	83.7	57%	0.18	72%	

	sequence supercontig Smp_scaff000059					
**FN357331.1**	Schistosoma mansoni genome sequence supercontig Smp_scaff000040	44.6	122	37%	0.18	78%
**FN357326.1**	Schistosoma mansoni genome sequence supercontig Smp_scaff000035	44.6	83.7	59%	0.18	72%
**FN357322.1**	Schistosoma mansoni genome sequence supercontig Smp_scaff000031	44.6	126	35%	0.18	76%
**FN357316.1**	Schistosoma mansoni genome sequence supercontig Smp_scaff000025	44.6	122	33%	0.18	74%
**FN368005.1**	Schistosoma mansoni genome sequence supercontig Smp_scaff010714	44.6	44.6	60%	0.18	69%
**FN360200.1**	Schistosoma mansoni genome sequence supercontig Smp_scaff002909	44.6	44.6	59%	0.18	68%
**AP010341.1**	Lotus japonicus genomic DNA, chromosome 3, clone: LjT15I02, TM0666, complete sequence	44.6	44.6	23%	0.18	80%
**AF391293.1**	Branchiostoma floridae cosmid MPMGc117K0849, partial sequence	44.6	44.6	30%	0.18	75%
**AC150407.2**	Branchiostoma floridae clone CH302-54J24, complete sequence	44.6	122	21%	0.18	81%
**NW_003039129.1**	Schistosoma mansoni genome sequence supercontig Smp_scaff000238	42.8	81.9	32%	0.65	74%
**NW_003038510.1**	Schistosoma mansoni genome sequence supercontig Smp_scaff001998	42.8	42.8	32%	0.65	74%
**NW_003038509.1**	Schistosoma mansoni genome sequence supercontig Smp_scaff001996	42.8	42.8	32%	0.65	74%
**NW_003038070.1**	Schistosoma mansoni genome sequence supercontig Smp_scaff000314	42.8	162	41%	0.65	73%
**NW_003038013.1**	Schistosoma mansoni genome sequence supercontig Smp_scaff000239	42.8	81.9	32%	0.65	74%
**NW_003038004.1**	Schistosoma mansoni genome sequence supercontig Smp_scaff000229	42.8	81.9	59%	0.65	69%
**NW_003038002.1**	Schistosoma mansoni genome sequence supercontig Smp_scaff000227	42.8	85.5	32%	0.65	75%
**NW_003037995.1**	Schistosoma mansoni genome sequence supercontig Smp_scaff000219	42.8	81.9	32%	0.65	74%
**NW_003037937.1**	Schistosoma mansoni genome sequence supercontig Smp_scaff000151	42.8	81.9	33%	0.65	73%
**NW_003037936.1**	Schistosoma mansoni genome sequence supercontig Smp_scaff000150	42.8	160	32%	0.65	74%
**NW_003037929.1**	Schistosoma mansoni genome sequence supercontig Smp_scaff000143	42.8	81.9	32%	0.65	74%
**NW_003037772.1**	Schistosoma mansoni genome sequence supercontig Smp_scaff018982	42.8	42.8	32%	0.65	74%
**NW_003037196.1**	Schistosoma mansoni genome sequence supercontig Smp_scaff012462	42.8	42.8	59%	0.65	69%
**NW_003035875.1**	Schistosoma mansoni genome sequence supercontig Smp_scaff001988	42.8	42.8	32%	0.65	74%
**NW_003033841.1**	Schistosoma mansoni genome sequence supercontig Smp_scaff010734	42.8	42.8	32%	0.65	74%
**NW_003033601.1**	Schistosoma mansoni genome	42.8	42.8	32%	0.65	74%

	sequence supercontig Smp_scaff008383					
**NW_003032283.1**	Schistosoma mansoni genome sequence supercontig Smp_scaff010563	42.8	42.8	32%	0.65	74%
**NW_003031788.1**	Schistosoma mansoni genome sequence supercontig Smp_scaff014561	42.8	42.8	22%	0.65	80%
**NW_003029805.1**	Schistosoma mansoni genome sequence supercontig Smp_scaff018657	42.8	42.8	41%	0.65	71%
**NW_003029774.1**	Schistosoma mansoni genome sequence supercontig Smp_scaff018615	42.8	42.8	59%	0.65	69%
**NW_003029322.1**	Schistosoma mansoni genome sequence supercontig Smp_scaff010146	42.8	42.8	32%	0.65	74%
**NW_003029272.1**	Schistosoma mansoni genome sequence supercontig Smp_scaff010092	42.8	42.8	32%	0.65	74%
**NW_003026612.1**	Schistosoma mansoni genome sequence supercontig Smp_scaff000592	42.8	163	32%	0.65	75%
**NW_003026608.1**	Schistosoma mansoni genome sequence supercontig Smp_scaff000579	42.8	42.8	28%	0.65	75%
**NW_003026458.1**	Schistosoma mansoni genome sequence supercontig Smp_scaff000069	42.8	206	32%	0.65	78%
**NW_003026430.1**	Schistosoma mansoni genome sequence supercontig Smp_scaff003036	42.8	42.8	33%	0.65	74%
**NW_003024332.1**	Schistosoma mansoni genome sequence supercontig Smp_scaff013696	42.8	42.8	32%	0.65	74%
**NW_003023815.1**	Schistosoma mansoni genome sequence supercontig Smp_scaff015661	42.8	42.8	32%	0.65	74%
**NW_003023484.1**	Schistosoma mansoni genome sequence supercontig Smp_scaff017714	42.8	42.8	22%	0.65	80%
**FN357529.1**	Schistosoma mansoni genome sequence supercontig Smp_scaff000238	42.8	81.9	32%	0.65	74%
**FN357369.1**	Schistosoma mansoni genome sequence supercontig Smp_scaff000078	42.8	81.9	32%	0.65	74%
**FN359289.1**	Schistosoma mansoni genome sequence supercontig Smp_scaff001998	42.8	42.8	32%	0.65	74%
**FN359287.1**	Schistosoma mansoni genome sequence supercontig Smp_scaff001996	42.8	42.8	32%	0.65	74%
**FN359282.1**	Schistosoma mansoni genome sequence supercontig Smp_scaff001991	42.8	81.9	32%	0.65	74%
**FN359228.1**	Schistosoma mansoni genome sequence supercontig Smp_scaff001937	42.8	42.8	52%	0.65	70%
**FN358757.1**	Schistosoma mansoni genome sequence supercontig Smp_scaff001466	42.8	42.8	32%	0.65	74%
**FN357920.1**	Schistosoma mansoni genome sequence supercontig Smp_scaff000629	42.8	243	61%	0.65	76%
**FN357900.1**	Schistosoma mansoni genome sequence supercontig Smp_scaff000609	42.8	42.8	32%	0.65	74%
**FN357865.1**	Schistosoma mansoni genome sequence supercontig Smp_scaff000574	42.8	81.9	37%	0.65	78%
**FN357718.1**	Schistosoma mansoni genome sequence supercontig	42.8	81.9	32%	0.65	74%

	Smp_scaff000427					
**FN357715.1**	Schistosoma mansoni genome sequence supercontig Smp_scaff000424	42.8	42.8	32%	0.65	74%
**FN357703.1**	Schistosoma mansoni genome sequence supercontig Smp_scaff000412	42.8	85.5	59%	0.65	74%
**FN357691.1**	Schistosoma mansoni genome sequence supercontig Smp_scaff000400	42.8	42.8	32%	0.65	74%

Alignments Select All Get selected sequences Distance tree of results Multiple alignment

>**emb|AJ586906.3|** Mytilus galloprovincialis hsp90-2 gene for heat shock protein 90
Length=12435

```
 Score =  363 bits (402),  Expect = 2e-97
 Identities = 201/201 (100%), Gaps = 0/201 (0%)
 Strand=Plus/Plus

Query  1     AACGAGAACCTTTTCATCAGATGCCAACAACAAGATATAGCCACAACTATAATAAGAAGA  60
             ||||||||||||||||||||||||||||||||||||||||||||||||||||||||||||
Sbjct  329   AACGAGAACCTTTTCATCAGATGCCAACAACAAGATATAGCCACAACTATAATAAGAAGA  388

Query  61    CGCTGGAGGTGGATTCGGCATGTCCTACGGAAAGGCCAGGGGGATATCACAAAAACAGCA  120
             ||||||||||||||||||||||||||||||||||||||||||||||||||||||||||||
Sbjct  389   CGCTGGAGGTGGATTCGGCATGTCCTACGGAAAGGCCAGGGGGATATCACAAAAACAGCA  448

Query  121   CTCCACTGGACCCCAGAAGGTAAAAGAAAGAGAGGAAGACCTAAAATGACATGGAGAAGA  180
             ||||||||||||||||||||||||||||||||||||||||||||||||||||||||||||
Sbjct  449   CTCCACTGGACCCCAGAAGGTAAAAGAAAGAGAGGAAGACCTAAAATGACATGGAGAAGA  508

Query  181   ACTGTAGAGGCAGAGGCTAGT  201
             |||||||||||||||||||||
Sbjct  509   ACTGTAGAGGCAGAGGCTAGT  529
```

>**emb|AM236589.2|** Mytilus galloprovincialis hsp90-1 gene for heat shock protein 90, exons 1-9
Length=8538

```
 Score =  363 bits (402),  Expect = 2e-97
 Identities = 201/201 (100%), Gaps = 0/201 (0%)
 Strand=Plus/Plus

Query  1     AACGAGAACCTTTTCATCAGATGCCAACAACAAGATATAGCCACAACTATAATAAGAAGA  60
             ||||||||||||||||||||||||||||||||||||||||||||||||||||||||||||
Sbjct  329   AACGAGAACCTTTTCATCAGATGCCAACAACAAGATATAGCCACAACTATAATAAGAAGA  388

Query  61    CGCTGGAGGTGGATTCGGCATGTCCTACGGAAAGGCCAGGGGGATATCACAAAAACAGCA  120
             ||||||||||||||||||||||||||||||||||||||||||||||||||||||||||||
Sbjct  389   CGCTGGAGGTGGATTCGGCATGTCCTACGGAAAGGCCAGGGGGATATCACAAAAACAGCA  448

Query  121   CTCCACTGGACCCCAGAAGGTAAAAGAAAGAGAGGAAGACCTAAAATGACATGGAGAAGA  180
             ||||||||||||||||||||||||||||||||||||||||||||||||||||||||||||
Sbjct  449   CTCCACTGGACCCCAGAAGGTAAAAGAAAGAGAGGAAGACCTAAAATGACATGGAGAAGA  508

Query  181   ACTGTAGAGGCAGAGGCTAGT  201
             |||||||||||||||||||||
Sbjct  509   ACTGTAGAGGCAGAGGCTAGT  529
```

>**dbj|AB081572.1|** Oryzias curvinotus DNA, LINE-like repetitive sequence Gamera, clone:Gamera-cur1
Length=4493

```
 Score = 69.8 bits (76),  Expect = 5e-09
 Identities = 125/181 (69%), Gaps = 4/181 (2%)
 Strand=Plus/Plus

Query  10    CTTTTCATCAGATGCCAACAACAAGATATAGCCACAACTATAATAAGAAGACGCTGGAGG  69
             || ||| || |  || | ||| |||| ||  |  ||| |||  | | |||||||||||  
Sbjct  1907  CTATTCTTCCGCAGCAATCAAGAAGACATGTCATCAATTATCCTCAAAAGACGCTGGACA  1966

Query  70    TGGATTCGGCATGTCCTACGGAAAG--GCCAGGGGGATATCACAAAAACAGCACTCCACT  127
             |||||| | ||||| ||| |||  |  | ||     |||  | ||| |||||||| || |
Sbjct  1967  TGGATTGGACATGTACTAAGGAGTGAAGACAACACAATA--ATAAAGACAGCACTACATT  2024

Query  128   GGACCCCAGAAGGTAAAAGAAAGAGAGGAAGACCTAAAATGACATGGAGAAGAACTGTAG  187
             |||| ||||| || |||||||| | |||| | || || || |  ||| |  |||| ||||
Sbjct  2025  GGACACCAGATGGGAAAAGAAAAACAGGACGGCCGAAGATCATCTGGTGCCGAACAGTAG  2084

Query  188   A  188
             |
Sbjct  2085  A  2085
```

>**emb|FN357346.1|** Schistosoma mansoni genome sequence supercontig Smp_scaff000055
Length=1017060

Features flanking this part of subject sequence:

27596 bp at 5' side: subfamily A1A unassigned peptidase (A01 family)
32142 bp at 3' side: subfamily A1A unassigned peptidase (A01 family)

```
 Score = 57.2 bits (62),  Expect = 3e-05
 Identities = 52/66 (78%), Gaps = 0/66 (0%)
 Strand=Plus/Plus

Query  107     TCACAAAAACAGCACTCCACTGGACCCCAGAAGGTAAAAGAAAGAGAGGAAGACCTAAAA  166
               |||||| |  |||||||   ||||  || |||||| ||||||||||||||||||| || |
Sbjct  388440  TCACAAGACAAGCACTCATATGGAATCCTGAAGGTGAAAGAAAGAGAGGAAGACCAAAGA  388499

Query  167     TGACAT  172
                 ||||
Sbjct  388500  ACACAT  388505
```

>**emb|FN357536.1|** Schistosoma mansoni genome sequence supercontig Smp_scaff000245
Length=650426

Sort alignments for this subject sequence by:
E value **Score** **Percent identity**
Query start position **Subject start position**

Features flanking this part of subject sequence:
55264 bp at 5' side: hypothetical protein
11316 bp at 3' side: hypothetical protein

```
 Score = 51.8 bits (56),  Expect = 0.001
 Identities = 52/68 (76%), Gaps = 0/68 (0%)
 Strand=Plus/Plus

Query  105     TATCACAAAAACAGCACTCCACTGGACCCCAGAAGGTAAAAGAAAGAGAGGAAGACCTAA  164
               |||||||| |  ||| |||   ||||  || |||||  ||||||  ||||||||||| ||
Sbjct  268323  TATCACAAGACAAGCCCTCACATGGAATCCTGAAGGCCAAAGAAGAAGAGGAAGACCAAA  268382

Query  165     AATGACAT  172
               ||| ||||
Sbjct  268383  AATCACAT  268390
```

Features flanking this part of subject sequence:
2613 bp at 5' side: family S28 unassigned peptidase (S28 family)
3657 bp at 3' side: hypothetical protein

```
 Score = 42.8 bits (46),  Expect = 0.65
 Identities = 48/62 (77%), Gaps = 2/62 (3%)
 Strand=Plus/Minus

Query  106    ATCACAAAAACAGCACTCCACTGGACCCCAGAAGGT-AAAAGAAAGAGAGGAAGACCTAA  164
              |||||||    |||||||   |||| ||| |||||  |||||| | ||||||||||| ||
Sbjct  27359  ATCACAAGGCAAGCACTCATATGGAACCCTGAAGGCCAAAAGAGA-AGAGGAAGACCAAA  27301

Query  165    AA  166
              ||
Sbjct  27300  AA  27299
```

>**emb|FN357353.1|** Schistosoma mansoni genome sequence supercontig Smp_scaff000062
Length=1412270

Sort alignments for this subject sequence by:
E value **Score** **Percent identity**
Query start position **Subject start position**

Features flanking this part of subject sequence:
86498 bp at 5' side: hypothetical protein
68431 bp at 3' side: hypothetical protein

```
 Score = 50.0 bits (54),  Expect = 0.004
 Identities = 48/59 (81%), Gaps = 2/59 (3%)
 Strand=Plus/Plus

Query  107     TCACAAAAACAGCACTCCAC-TGGACCCCAGAAGGTAAAAGAAAGAGAGGAAGACCTAA  164
               |||||| |  ||||| |||| ||||  || |||||| ||||||| ||||||||||| ||
Sbjct  176834  TCACAAGATAAGCAC-CCACATGGAATCCTGAAGGTCAAAGAAAAAGAGGAAGACCAAA  176891
```

Features flanking this part of subject sequence:
16153 bp at 5' side: expressed protein
727 bp at 3' side: gsx family homeobox protein

```
 Score = 42.8 bits (46),  Expect = 0.65
 Identities = 49/66 (74%), Gaps = 0/66 (0%)
 Strand=Plus/Minus

Query  107     TCACAAAAACAGCACTCCACTGGACCCCAGAAGGTAAAAGAAAGAGAGGAAGACCTAAAA  166
               |||||| |  ||| |||   || |  || ||| || ||||||||||||||||||| || |
Sbjct  903003  TCACAAGACAAGCCCTCACATGAAATCCTGAAAGTGAAAGAAAGAGAGGAAGACCAAAGA  902944

Query  167     TGACAT  172
                 ||||
Sbjct  902943  ACACAT  902938
```

Features flanking this part of subject sequence:
43501 bp at 5' side: hypothetical protein
34429 bp at 3' side: hypothetical protein

```
 Score = 39.2 bits (42),  Expect = 7.9
 Identities = 44/58 (75%), Gaps = 1/58 (1%)
 Strand=Plus/Minus

Query  107    TCACAAAAACAGCACTCCACTGGACCCCAGAAGGTAAAAGAAAGAGAGGAAGACCTAA  164
              |||||| |  ||| ||| | ||||  || |||||| |||| |  ||||||||||| ||
Sbjct  55494  TCACAAGACAAGCCCTCAA-TGGAATCCTGAAGGTCAAAGGAGAAGAGGAAGACCAAA  55438
```

>**emb|FN357327.1|** Schistosoma mansoni genome sequence supercontig Smp_scaff000036
Length=2467244

Sort alignments for this subject sequence by:
E value **Score** **Percent identity**
Query start position **Subject start position**

Features in this part of subject sequence:
hypothetical protein

```
 Score = 50.0 bits (54),  Expect = 0.004
 Identities = 51/66 (77%), Gaps = 2/66 (3%)
 Strand=Plus/Minus

Query  107     TCACAAAAACAGCACTCCACTGGACCCCAGAAGGTAAAAGAAAGAGAGGAAGACCTAAAA  166
               ||||||  | |||||||   ||||  || |||||| |||| || |||||||||||  |||
Sbjct  191114  TCACAAGGAAAGCACTCACGTGGAATCCCGAAGGTCAAAGGAAAAGAGGAAGACC--AAA  191057

Query  167     TGACAT  172
               | ||||
Sbjct  191056  TAACAT  191051
```

Features in this part of subject sequence:
expressed protein

```
 Score = 42.8 bits (46),  Expect = 0.65
 Identities = 44/58 (75%), Gaps = 0/58 (0%)
 Strand=Plus/Plus

Query  107     TCACAAAAACAGCACTCCACTGGACCCCAGAAGGTAAAAGAAAGAGAGGAAGACCTAA  164
               |||||| |  ||| |||   ||||  || |||||| ||||||  ||||||||||| ||
Sbjct  619584  TCACAAGACAAGCCCTCACATGGAATCCTGAAGGTCAAAGAAGAAGAGGAAGACCAAA  619641
```

Features flanking this part of subject sequence:
7062 bp at 5' side: expressed protein
5650 bp at 3' side: dipeptidyl-peptidase III (M49 family)

```
 Score = 42.8 bits (46),  Expect = 0.65
 Identities = 49/66 (74%), Gaps = 0/66 (0%)
 Strand=Plus/Minus

Query  107     TCACAAAAACAGCACTCCACTGGACCCCAGAAGGTAAAAGAAAGAGAGGAAGACCTAAAA  166
               |||||| |  ||| |||   ||||  || |||||| |||| || ||||||||||| || |
Sbjct  746746  TCACAAGACAAGCCCTCATTTGGAATCCTGAAGGTCAAAGGAAAAGAGGAAGACCAAAGA  746687

Query  167     TGACAT  172
                 ||||
Sbjct  746686  ACACAT  746681
```

Features flanking this part of subject sequence:
13229 bp at 5' side: dipeptidyl-peptidase III (M49 family)
11211 bp at 3' side: choline/ethanolamine kinase, putative

```
 Score = 42.8 bits (46),  Expect = 0.65
 Identities = 49/66 (74%), Gaps = 0/66 (0%)
 Strand=Plus/Plus

Query  107     TCACAAAAACAGCACTCCACTGGACCCCAGAAGGTAAAAGAAAGAGAGGAAGACCTAAAA  166
               |||||| |  ||| |||   ||||  || |||||  ||||||  ||||||||||| ||||
Sbjct  772242  TCACAAGACAAGCCCTCACATGGAATCCTGAAGGCCAAAGAAGAAGAGGAAGACCAAAAA  772301

Query  167     TGACAT  172
                 ||||
Sbjct  772302  ACACAT  772307
```

Features flanking this part of subject sequence:
53403 bp at 5' side: hypothetical protein
19594 bp at 3' side: guanine-nucleotide-exchange-factor, putative

```
 Score = 41.0 bits (44),  Expect = 2.3
 Identities = 49/67 (73%), Gaps = 0/67 (0%)
 Strand=Plus/Minus

Query  106      ATCACAAAAACAGCACTCCACTGGACCCCAGAAGGTAAAAGAAAGAGAGGAAGACCTAAA  165
                ||||||| |  ||| |||   ||||  || |||||| |||| |  ||||||||||| ||
Sbjct  1271790  ATCACAAGACAAGCCCTCACATGGAATCCTGAAGGTCAAAGGAGAAGAGGAAGACCAAAG  1271731

Query  166      ATGACAT  172
                |  ||||
Sbjct  1271730  AACACAT  1271724
```

Features in this part of subject sequence:
subfamily A1A unassigned peptidase (A01 family)

```
 Score = 39.2 bits (42),  Expect = 7.9
```

```
 Identities = 48/66 (72%), Gaps = 0/66 (0%)
 Strand=Plus/Plus

Query  107    TCACAAAAACAGCACTCCACTGGACCCCAGAAGGTAAAAGAAAGAGAGGAAGACCTAAAA  166
              |||||| |  ||| |||   ||||  || |||||| |||| |  ||||||||||| || |
Sbjct  64470  TCACAAGACAAGCCCTCACATGGAATCCTGAAGGTCAAAGGAGAAGAGGAAGACCAAAGA  64529

Query  167    TGACAT  172
                ||||
Sbjct  64530  ACACAT  64535
```

Features flanking this part of subject sequence:
8144 bp at 5' side: hypothetical protein
1198 bp at 3' side: phosphoglycerate kinase

```
 Score = 39.2 bits (42),  Expect = 7.9
 Identities = 48/66 (72%), Gaps = 0/66 (0%)
 Strand=Plus/Plus

Query  107     TCACAAAAACAGCACTCCACTGGACCCCAGAAGGTAAAAGAAAGAGAGGAAGACCTAAAA  166
               |||||| |  ||| |||   ||||  || |||||| |||| |  ||||||||||| || |
Sbjct  336691  TCACAAGACAAGCCCTCACATGGAATCCTGAAGGTCAAAGGAGAAGAGGAAGACCAAAGA  336750

Query  167     TGACAT  172
                 ||||
Sbjct  336751  ACACAT  336756
```

Features in this part of subject sequence:
guanine-nucleotide-exchange-factor, putative

```
 Score = 39.2 bits (42),  Expect = 7.9
 Identities = 48/66 (72%), Gaps = 0/66 (0%)
 Strand=Plus/Plus

Query  107      TCACAAAAACAGCACTCCACTGGACCCCAGAAGGTAAAAGAAAGAGAGGAAGACCTAAAA  166
                |||||| |  ||| |||   ||||  || |||||| |||| |  ||||||||||| || |
Sbjct  1309925  TCACAAGACAAGCCCTCACGTGGAATCCTGAAGGTCAAAGGAGAAGAGGAAGACCAAAGA  1309984

Query  167      TGACAT  172
                  ||||
Sbjct  1309985  ACACAT  1309990
```

Features flanking this part of subject sequence:
18019 bp at 5' side: RER1 protein, putative
19850 bp at 3' side: hypothetical protein

```
 Score = 39.2 bits (42),  Expect = 7.9
 Identities = 48/66 (72%), Gaps = 0/66 (0%)
 Strand=Plus/Plus

Query  107      TCACAAAAACAGCACTCCACTGGACCCCAGAAGGTAAAAGAAAGAGAGGAAGACCTAAAA  166
                |||||| |  ||| |||   ||||  || |||||| |||| |  ||||||||||| || |
Sbjct  2337881  TCACAAGACAAGCCCTCACATGGAATCCTGAAGGTCAAAGGAGAAGAGGAAGACCAAAGA  2337940

Query  167      TGACAT  172
                  ||||
Sbjct  2337941  ACACAT  2337946
```

>**emb|FN357292.1|** D Schistosoma mansoni genome sequence supercontig Smp_scaff000001
Length=4179495

Sort alignments for this subject sequence by:
E value **Score** **Percent identity**
Query start position **Subject start position**

Features flanking this part of subject sequence:
42636 bp at 5' side: hypothetical conserved protein
37312 bp at 3' side: hypothetical conserved protein

```
 Score = 50.0 bits (54),  Expect = 0.004
 Identities = 39/46 (84%), Gaps = 2/46 (4%)
 Strand=Plus/Plus

Query  127      TGGACCCCAGAAGGTAAAAGAAAGAGAGGAAGACCTAAAATGACAT  172
                ||||  ||||||||||||||||| || ||||||||  ||| |||||
Sbjct  4139504  TGGAATCCAGAAGGTAAAAGAAAAAGGGGAAGACC--AAAGGACAT  4139547
```

Features flanking this part of subject sequence:
22184 bp at 5' side: similar to potassium channel-interacting protein 4 isofor...
30852 bp at 3' side: oxidoreductase, short chain dehydrogenase/reductase famil...

```
 Score = 44.6 bits (48),  Expect = 0.18
 Identities = 50/67 (74%), Gaps = 0/67 (0%)
 Strand=Plus/Plus

Query  106     ATCACAAAAACAGCACTCCACTGGACCCCAGAAGGTAAAAGAAAGAGAGGAAGACCTAAA  165
               ||||||| |  ||| |||   ||||  || |||||| ||||||  |||||||| || |||
Sbjct  996831  ATCACAAGACAAGCCCTCACATGGAATCCTGAAGGTCAAAGAAGAAGAGGAAGCCCAAAA  996890

Query  166     ATGACAT  172
               |  ||||
Sbjct  996891  AACACAT  996897
```

Features flanking this part of subject sequence:
28630 bp at 5' side: similar to potassium channel-interacting protein 4 isofor...
24406 bp at 3' side: oxidoreductase, short chain dehydrogenase/reductase famil...

Score = 44.6 bits (48), Expect = 0.18
Identities = 50/67 (74%), Gaps = 0/67 (0%)
Strand=Plus/Plus

```
Query  106      ATCACAAAAACAGCACTCCACTGGACCCCAGAAGGTAAAAGAAAGAGAGGAAGACCTAAA  165
                ||||||| |  ||| |||   ||||  || |||||| ||||||  |||||||| || |||
Sbjct  1003277  ATCACAAGACAAGCCCTCACATGGAATCCTGAAGGTCAAAGAAGAAGAGGAAGCCCAAAA  1003336

Query  166      ATGACAT  172
                |  ||||
Sbjct  1003337  AACACAT  1003343
```

Features flanking this part of subject sequence:
10312 bp at 5' side: homeodomain-interacting protein kinase 2 (ec 2.7.1.37) (h...
20826 bp at 3' side: ADP-ribosylation factor family

Score = 42.8 bits (46), Expect = 0.65
Identities = 37/46 (80%), Gaps = 0/46 (0%)
Strand=Plus/Minus

```
Query  127      TGGACCCCAGAAGGTAAAAGAAAGAGAGGAAGACCTAAAATGACAT  172
                ||||  || |||||| |||| || ||||||||||| ||||  ||||
Sbjct  2429644  TGGAATCCTGAAGGTCAAAGGAAAAGAGGAAGACCAAAAAACACAT  2429599
```

Features in this part of subject sequence:
hypothetical conserved protein

Score = 42.8 bits (46), Expect = 0.65
Identities = 49/65 (75%), Gaps = 2/65 (3%)
Strand=Plus/Plus

```
Query  107      TCACAAAAACAGCACTCCACTGGACCCCAGAAGGTAAAAGAAAGAGAGGAAGACCTAAAA  166
                ||||||||  ||| |||   |||| ||| |||||  |||| |  |||||||||||  |||
Sbjct  3830206  TCACAAAACAAGCCCTCACATGGAACCCTGAAGGCCAAAGGAGAAGAGGAAGACC--AAA  3830263

Query  167      TGACA  171
                 ||||
Sbjct  3830264  GGACA  3830268
```

Features in this part of subject sequence:
family M13 non-peptidase homologue (M13 family)

Score = 41.0 bits (44), Expect = 2.3
Identities = 28/32 (87%), Gaps = 0/32 (0%)
Strand=Plus/Minus

```
Query  133      CCAGAAGGTAAAAGAAAGAGAGGAAGACCTAA  164
                || ||||| ||| |||||||||||||||| ||
Sbjct  2529000  CCTGAAGGGAAACGAAAGAGAGGAAGACCAAA  2528969
```

Features flanking this part of subject sequence:
54225 bp at 5' side: mitotic phosphoprotein 44
28484 bp at 3' side: G-protein coupled receptor fragment, putative

Score = 41.0 bits (44), Expect = 2.3
Identities = 49/67 (73%), Gaps = 0/67 (0%)
Strand=Plus/Minus

```
Query  106      ATCACAAAAACAGCACTCCACTGGACCCCAGAAGGTAAAAGAAAGAGAGGAAGACCTAAA  165
                |||||||||| ||| |||   ||||  ||  |||||| |||| |  ||| ||| ||| ||
Sbjct  3004043  ATCACAAAAAAAGCTCTCACATGGAATCCTGAAGGTCAAAGGAGAAGAAGAACACCAAAG  3003984

Query  166      ATGACAT  172
                |  ||||
Sbjct  3003983  AACACAT  3003977
```

Features in this part of subject sequence:
similar to Protein C20orf152 homolog, putative

Score = 41.0 bits (44), Expect = 2.3
Identities = 45/59 (76%), Gaps = 1/59 (1%)
Strand=Plus/Plus

```
Query  107      TCACAAAAACAGCACTCCACTGGACCCCAGAAGGTAAAAGAAAGAGAGGAAGACCTAAA  165
                |||||| |  ||| |||   ||||  || |||||| ||||||  ||||||||| |||||
Sbjct  3492502  TCACAAGACGAGCCCTCACATGGAATCCTGAAGGTCAAAGAAGAAGAGGAAGA-CTAAA  3492559
```

Features flanking this part of subject sequence:
821 bp at 5' side: hypothetical protein
11527 bp at 3' side: similar to female-specific protein 800 (fs800)

Score = 39.2 bits (42), Expect = 7.9
Identities = 48/66 (72%), Gaps = 0/66 (0%)
Strand=Plus/Plus

```
Query  107      TCACAAAAACAGCACTCCACTGGACCCCAGAAGGTAAAAGAAAGAGAGGAAGACCTAAAA  166
                ||||||||  ||| |||   ||||  || |||||  |||| |  ||||||||||| || |
Sbjct  1243295  TCACAAAACAAGCCCTCACATGGAGTCCTGAAGGCCAAAGGAGAAGAGGAAGACCAAAGA  1243354
```

```
Query  167      TGACAT  172
                  ||||
Sbjct  1243355  ACACAT  1243360
```

Features flanking this part of subject sequence:
1520 bp at 5' side: hypothetical protein
2243 bp at 3' side: hypothetical protein

Score = 39.2 bits (42), Expect = 7.9
Identities = 48/66 (72%), Gaps = 0/66 (0%)
Strand=Plus/Minus

```
Query  107      TCACAAAAACAGCACTCCACTGGACCCCAGAAGGTAAAAGAAAGAGAGGAAGACCTAAAA  166
                |||||| |  ||| |||   ||||  || |||||| |||| |  |||||||||| || |
Sbjct  1640920  TCACAAGACAAGCCCTCACATGGAATCCTGAAGGTCAAAGGAGAAGAGGAAGACCAAAGA  1640861

Query  167      TGACAT  172
                  ||||
Sbjct  1640860  ACACAT  1640855
```

Features in this part of subject sequence:
similar to cancer susceptibility candidate 1; lung adenom...

Score = 39.2 bits (42), Expect = 7.9
Identities = 48/66 (72%), Gaps = 0/66 (0%)
Strand=Plus/Minus

```
Query  107      TCACAAAAACAGCACTCCACTGGACCCCAGAAGGTAAAAGAAAGAGAGGAAGACCTAAAA  166
                |||||| |  ||| |||   ||||  || |||||| |||| |  |||||||||| || |
Sbjct  3126511  TCACAAGACAAGCCCTCACATGGAATCCTGAAGGTCAAAGGAGAAGAGGAAGACCGAAGA  3126452

Query  167      TGACAT  172
                  ||||
Sbjct  3126451  ACACAT  3126446
```

Features in this part of subject sequence:
hypothetical conserved

Score = 39.2 bits (42), Expect = 7.9
Identities = 48/65 (73%), Gaps = 2/65 (3%)
Strand=Plus/Minus

```
Query  107      TCACAAAAACAGCACTCCACTGGACCCCAGAAGGTAAAAGAAAGAGAGGAAGACCTAAAA  166
                |||||| |  ||| |||   ||||  || |||||  ||||||  ||||||||||  |||
Sbjct  3344483  TCACAAGACAAGCCCTCACATGGAATCCTGAAGGCCAAAGAAGAAGAGGAAGACC--AAA  3344426

Query  167      TGACA  171
                | |||
Sbjct  3344425  TAACA  3344421
```

Features in this part of subject sequence:
hypothetical protein

Score = 39.2 bits (42), Expect = 7.9
Identities = 48/66 (72%), Gaps = 0/66 (0%)
Strand=Plus/Minus

```
Query  107      TCACAAAAACAGCACTCCACTGGACCCCAGAAGGTAAAAGAAAGAGAGGAAGACCTAAAA  166
                |||||| |  ||| |||   ||||  || |||||  ||||||| |||| |||||| || |
Sbjct  3615557  TCACAATACAAGCCCTCACATGGAATCCTGAAGGCCAAAGAAAAAGAGAAAGACCAAAGA  3615498

Query  167      TGACAT  172
                  ||||
Sbjct  3615497  ATACAT  3615492
```

>**ref|NW_003027857.1|** Schistosoma mansoni genome sequence supercontig Smp_scaff014004
Length=2895

Score = 48.2 bits (52), Expect = 0.015
Identities = 50/66 (75%), Gaps = 0/66 (0%)
Strand=Plus/Plus

```
Query  107   TCACAAAAACAGCACTCCACTGGACCCCAGAAGGTAAAAGAAAGAGAGGAAGACCTAAAA  166
             |||||| |  ||| |||   ||||  || |||||| |||||| ||||||||||  ||||
Sbjct  1820  TCACAAGACAAGCCCTCACATGGAATCCTGAAGGTCAAAGAAGGAGAGGAAGACAAAAAA  1879

Query  167   TGACAT  172
               ||||
Sbjct  1880  ACACAT  1885
```

>**emb|FN357526.1|** D Schistosoma mansoni genome sequence supercontig Smp_scaff000235
Length=709327

Sort alignments for this subject sequence by:
E value **Score** **Percent identity**
Query start position **Subject start position**

Features flanking this part of subject sequence:
14439 bp at 5' side: expressed protein
17252 bp at 3' side: expressed protein

Score = 48.2 bits (52), Expect = 0.015
Identities = 50/66 (75%), Gaps = 0/66 (0%)

Strand=Plus/Minus

```
Query  107     TCACAAAAACAGCACTCCACTGGACCCCAGAAGGTAAAAGAAAGAGAGGAAGACCTAAAA  166
               |||||| |  ||| ||| | ||||  ||||||||| |||| |  ||||||||||| || |
Sbjct  147001  TCACAAGACAAGCCCTCAAGTGGAATCCAGAAGGTCAAAGGAGAAGAGGAAGACCAAAGA  146942

Query  167     TGACAT  172
                 ||||
Sbjct  146941  ACACAT  146936
```

Features flanking this part of subject sequence:
4922 bp at 5' side: strawberry notch-related
327 bp at 3' side: Abnormal long morphology protein 1 (Sp8), putative

Score = 42.8 bits (46), Expect = 0.65
Identities = 49/66 (74%), Gaps = 0/66 (0%)
Strand=Plus/Plus

```
Query  107     TCACAAAAACAGCACTCCACTGGACCCCAGAAGGTAAAAGAAAGAGAGGAAGACCTAAAA  166
               |||||| |  ||| |||   ||||  || |||||| || | || ||||||||||| ||||
Sbjct  683299  TCACAAGACAAGCCCTCACATGGAATCCTGAAGGTCAACGGAAAAGAGGAAGACCAAAAA  683358

Query  167     TGACAT  172
                 ||||
Sbjct  683359  ACACAT  683364
```

Features flanking this part of subject sequence:
5120 bp at 5' side: expressed protein
8720 bp at 3' side: tubulin tyrosine ligase-related

Score = 39.2 bits (42), Expect = 7.9
Identities = 48/66 (72%), Gaps = 0/66 (0%)
Strand=Plus/Minus

```
Query  107     TCACAAAAACAGCACTCCACTGGACCCCAGAAGGTAAAAGAAAGAGAGGAAGACCTAAAA  166
               |||||| |  ||| |||   ||||  || |||||| |||| |  ||||||||||| || |
Sbjct  283315  TCACAAGACAAGCCCTCGCATGGAATCCTGAAGGTCAAAGGAGAAGAGGAAGACCAAAGA  283256

Query  167     TGACAT  172
                 ||||
Sbjct  283255  ACACAT  283250
```

Features in this part of subject sequence:
voltage-gated cation channel, putative

Score = 39.2 bits (42), Expect = 7.9
Identities = 48/66 (72%), Gaps = 0/66 (0%)
Strand=Plus/Minus

```
Query  107     TCACAAAAACAGCACTCCACTGGACCCCAGAAGGTAAAAGAAAGAGAGGAAGACCTAAAA  166
               |||||| |  ||| |||   ||||  || |||||  |||| |  ||||||||||| ||||
Sbjct  432912  TCACAAGATAAGCCCTCACATGGAATCCTGAAGGCCAAAGGAGAAGAGGAAGACCAAAAA  432853

Query  167     TGACAT  172
                 ||||
Sbjct  432852  ACACAT  432847
```

>**emb|FN357417.1|** D Schistosoma mansoni genome sequence supercontig Smp_scaff000126
Length=1580399

Sort alignments for this subject sequence by:
E value **Score** **Percent identity**
Query start position **Subject start position**

Features in this part of subject sequence:
heat shock protein, putative

Score = 48.2 bits (52), Expect = 0.015
Identities = 50/65 (76%), Gaps = 2/65 (3%)
Strand=Plus/Minus

```
Query  107     TCACAAAAACAGCACTCCACTGGACCCCAGAAGGTAAAAGAAAGAGAGGAAGACCTAAAA  166
               |||||| |  ||| |||   ||||  || |||||| |||| ||||||||||||||  |||
Sbjct  101604  TCACAAGACAAGCCCTCACATGGAATCCTGAAGGTCAAAGGAAGAGAGGAAGACC--AAA  101547

Query  167     TGACA  171
                ||||
Sbjct  101546  GGACA  101542
```

Features in this part of subject sequence:
ATP-dependent transporter, putative

Score = 39.2 bits (42), Expect = 7.9
Identities = 36/46 (78%), Gaps = 0/46 (0%)
Strand=Plus/Plus

```
Query  127     TGGACCCCAGAAGGTAAAAGAAAGAGAGGAAGACCTAAAATGACAT  172
               ||||  || |||||| |||| |  ||||||||||| ||||  ||||
Sbjct  827455  TGGAATCCTGAAGGTCAAAGGAGAAGAGGAAGACCAAAAAACACAT  827500
```

Features in this part of subject sequence:
hypothetical protein

Score = 39.2 bits (42), Expect = 7.9
Identities = 48/66 (72%), Gaps = 0/66 (0%)
Strand=Plus/Plus

```
Query  107      TCACAAAAACAGCACTCCACTGGACCCCAGAAGGTAAAAGAAAGAGAGGAAGACCTAAAA  166
                |||||| |  ||| |||   ||||  || |||||| |||| |  ||||||||||| || |
Sbjct  1389224  TCACAAGACAAGCCCTCACATGGAATCCTGAAGGTCAAAGGAGAAGAGGAAGACCAAAGA  1389283

Query  167      TGACAT  172
                  ||||
Sbjct  1389284  ACACAT  1389289
```

>**emb|FN357365.1|** Schistosoma mansoni genome sequence supercontig Smp_scaff000074
Length=1734107

Sort alignments for this subject sequence by:
E value **Score** **Percent identity**
Query start position **Subject start position**

Features in this part of subject sequence:
cation-transporting atpase fly, putative

Score = 48.2 bits (52), Expect = 0.015
Identities = 50/66 (75%), Gaps = 0/66 (0%)
Strand=Plus/Minus

```
Query  107      TCACAAAAACAGCACTCCACTGGACCCCAGAAGGTAAAAGAAAGAGAGGAAGACCTAAAA  166
                |||||| |  ||| |||   ||||  || ||||| ||||| || |||||||||||||| |
Sbjct  1682251  TCACAAGACAAGCCCTCACATGGAATCCTGAAGGCAAAAGGAAAAGAGGAAGACCTAAGA  1682192

Query  167      TGACAT  172
                  ||||
Sbjct  1682191  ACACAT  1682186
```

Features in this part of subject sequence:
expressed protein
expressed protein

Score = 41.0 bits (44), Expect = 2.3
Identities = 28/32 (87%), Gaps = 0/32 (0%)
Strand=Plus/Minus

```
Query  133    CCAGAAGGTAAAAGAAAGAGAGGAAGACCTAA  164
              || ||||| ||| |||||||||||||||| ||
Sbjct  87284  CCTGAAGGGAAACGAAAGAGAGGAAGACCAAA  87253
```

Features flanking this part of subject sequence:
53101 bp at 5' side: sh3 domain-binding glutamic acid-rich-like protein
71990 bp at 3' side: nephrin related

Score = 39.2 bits (42), Expect = 7.9
Identities = 27/31 (87%), Gaps = 0/31 (0%)
Strand=Plus/Plus

```
Query  143      AAAGAAAGAGAGGAAGACCTAAAATGACATG  173
                |||| |||||||||||||| ||||  |||||
Sbjct  1151363  AAAGGAAGAGAGGAAGACCAAAAAACACATG  1151393
```

Features flanking this part of subject sequence:
113237 bp at 5' side: sh3 domain-binding glutamic acid-rich-like protein
11819 bp at 3' side: nephrin related

Score = 39.2 bits (42), Expect = 7.9
Identities = 48/66 (72%), Gaps = 0/66 (0%)
Strand=Plus/Minus

```
Query  107      TCACAAAAACAGCACTCCACTGGACCCCAGAAGGTAAAAGAAAGAGAGGAAGACCTAAAA  166
                |||||| |  ||| |||   ||||  || |||||| |||| |  ||||||||||| || |
Sbjct  1211564  TCACAAGACAAGCCCTCACATGGAATCCTGAAGGTCAAAGGAGAAGAGGAAGACCAAAGA  1211505

Query  167      TGACAT  172
                  ||||
Sbjct  1211504  ACACAT  1211499
```

>**emb|FN357363.1|** Schistosoma mansoni genome sequence supercontig Smp_scaff000072
Length=875107

Sort alignments for this subject sequence by:
E value **Score** **Percent identity**
Query start position **Subject start position**

Features flanking this part of subject sequence:
53535 bp at 5' side: expressed protein
21948 bp at 3' side: expressed protein

Score = 48.2 bits (52), Expect = 0.015
Identities = 50/66 (75%), Gaps = 0/66 (0%)
Strand=Plus/Plus

```
Query  107     TCACAAAAACAGCACTCCACTGGACCCCAGAAGGTAAAAGAAAGAGAGGAAGACCTAAAA  166
               |||||| |  ||| |||   ||||  ||||||||  ||||||| ||||||||||| || |
Sbjct  164278  TCACAAGACAAGCCCTCACATGGAATCCAGAAGGCCAAAGAAAAAGAGGAAGACCAAAGA  164337
```

```
Query  167     TGACAT  172
                 ||||
Sbjct  164338  ACACAT  164343


 Features in this part of subject sequence:
   Intraflagellar transport 81 (Carnitine deficiency-associa...

 Score = 39.2 bits (42),  Expect = 7.9
 Identities = 48/66 (72%), Gaps = 0/66 (0%)
 Strand=Plus/Minus

Query  107     TCACAAAAACAGCACTCCACTGGACCCCAGAAGGTAAAAGAAAGAGAGGAAGACCTAAAA  166
               |||||| |  ||| |||   ||||  || |||||| |||| |  ||||||||||| || |
Sbjct  404304  TCACAAGACAAGCCCTCACATGGAATCCTGAAGGTCAAAGGAGAAGAGGAAGACCAAAGA  404245

Query  167     TGACAT  172
                 ||||
Sbjct  404244  ACACAT  404239


 Features flanking this part of subject sequence:
   7469 bp at 5' side: kinesin-like protein
   11731 bp at 3' side: expressed protein

 Score = 39.2 bits (42),  Expect = 7.9
 Identities = 48/66 (72%), Gaps = 0/66 (0%)
 Strand=Plus/Minus

Query  107     TCACAAAAACAGCACTCCACTGGACCCCAGAAGGTAAAAGAAAGAGAGGAAGACCTAAAA  166
               |||||| |  ||| |||   ||||  || |||||| |||| |  ||||||||||| || |
Sbjct  576576  TCACAAGACAAGCCCTCACATGGAATCCTGAAGGTCAAAGGAGAAGAGGAAGACCAAAGA  576517

Query  167     TGACAT  172
                 ||||
Sbjct  576516  ACACAT  576511


 Features flanking this part of subject sequence:
   12864 bp at 5' side: expressed protein
   20976 bp at 3' side: hypothetical protein

 Score = 39.2 bits (42),  Expect = 7.9
 Identities = 48/66 (72%), Gaps = 0/66 (0%)
 Strand=Plus/Plus

Query  107     TCACAAAAACAGCACTCCACTGGACCCCAGAAGGTAAAAGAAAGAGAGGAAGACCTAAAA  166
               |||||| |  ||| |||   ||||  || |||||| |||| |  ||||||||||| || |
Sbjct  613079  TCACAAGACAAGCCCTCACATGGAATCCTGAAGGTCAAAGGAGAAGAGGAAGACCAAAGA  613138

Query  167     TGACAT  172
                 ||||
Sbjct  613139  ACACAT  613144


 Features flanking this part of subject sequence:
   17826 bp at 5' side: expressed protein
   16014 bp at 3' side: hypothetical protein

 Score = 39.2 bits (42),  Expect = 7.9
 Identities = 48/66 (72%), Gaps = 0/66 (0%)
 Strand=Plus/Plus

Query  107     TCACAAAAACAGCACTCCACTGGACCCCAGAAGGTAAAAGAAAGAGAGGAAGACCTAAAA  166
               |||||| |  ||| |||   ||||  || |||||| |||| |  ||||||||||| || |
Sbjct  618041  TCACAAGACAAGCCCTCACATGGAATCCTGAAGGTCAAAGGAGAAGAGGAAGACCAAAGA  618100

Query  167     TGACAT  172
                 ||||
Sbjct  618101  ACACAT  618106


 Features flanking this part of subject sequence:
   10713 bp at 5' side: DNA replication licensing factor MCM7, putative
   15300 bp at 3' side: importin-beta 3, putative

 Score = 39.2 bits (42),  Expect = 7.9
 Identities = 48/66 (72%), Gaps = 0/66 (0%)
 Strand=Plus/Plus

Query  107     TCACAAAAACAGCACTCCACTGGACCCCAGAAGGTAAAAGAAAGAGAGGAAGACCTAAAA  166
               |||||| |  ||| |||   ||||  || |||||  |||| || ||||||||||| || |
Sbjct  777224  TCACAAGACAAGCCCTCACATGGAATCCTGAAGGCCAAAGGAAAAGAGGAAGACCAAAGA  777283

Query  167     TGACAT  172
                 ||||
Sbjct  777284  ACACAT  777289


>emb|FN371295.1|  Schistosoma mansoni genome sequence supercontig Smp_scaff014004
Length=2895

 Score = 48.2 bits (52),  Expect = 0.015
 Identities = 50/66 (75%), Gaps = 0/66 (0%)
 Strand=Plus/Plus

Query  107   TCACAAAAACAGCACTCCACTGGACCCCAGAAGGTAAAAGAAAGAGAGGAAGACCTAAAA  166
             |||||| |  ||| |||   ||||  || |||||| |||||| |||||||||||  ||||
Sbjct  1820  TCACAAGACAAGCCCTCACATGGAATCCTGAAGGTCAAAGAAGGAGAGGAAGACAAAAAA  1879
```

```
Query  167   TGACAT  172
               ||||
Sbjct  1880  ACACAT  1885


>gb|AC192734.2|  Pan troglodytes BAC clone CH251-397M3 from chromosome 7, complete
sequence
Length=145354

 Score = 48.2 bits (52),  Expect = 0.015
 Identities = 31/33 (93%), Gaps = 1/33 (3%)
 Strand=Plus/Minus

Query  151     AGAGGAAGA-CCTAAAATGACATGGAGAAGAAC  182
               ||||||||| |||| ||||||||||||||||||
Sbjct  136802  AGAGGAAGAGCCTAGAATGACATGGAGAAGAAC  136770


>gb|AC147078.2|  Pan troglodytes BAC clone RP43-180L22 from chromosome 7, complete
sequence
Length=217576

 Score = 48.2 bits (52),  Expect = 0.015
 Identities = 31/33 (93%), Gaps = 1/33 (3%)
 Strand=Plus/Minus

Query  151   AGAGGAAGA-CCTAAAATGACATGGAGAAGAAC  182
             ||||||||| |||| ||||||||||||||||||
Sbjct  7075  AGAGGAAGAGCCTAGAATGACATGGAGAAGAAC  7043


>ref|NW_003038502.1|  Schistosoma mansoni genome sequence supercontig Smp_scaff001979
Length=185055

 Score = 46.4 bits (50),  Expect = 0.053
 Identities = 85/122 (69%), Gaps = 7/122 (5%)
 Strand=Plus/Minus

Query  54     AAGAAGACGCTGGAGGTGGATTCGGCATGTCCTACGGAAAGGCC---AGGGGGATATCAC  110
              |||||| ||||||| ||||||| ||||   |||  |||||   |   |  | |   ||||
Sbjct  56776  AAGAAG-CGCTGGAAGTGGATTGGGCACACCCTGAGGAAATCACCTAATTGCG---TCAC  56721

Query  111    AAAAACAGCACTCCACTGGACCCCAGAAGGTAAAAGAAAGAGAGGAAGACCTAAAATGAC  170
              || |  |||||||   ||||  || |||||| |||| |  ||||||||||| || |  ||
Sbjct  56720  AAGACAAGCACTCACATGGAATCCTGAAGGTCAAAGGAGAAGAGGAAGACCAAAGAACAC  56661

Query  171    AT  172
              ||
Sbjct  56660  AT  56659


>ref|NW_003038472.1|  Schistosoma mansoni genome sequence supercontig Smp_scaff001935
Length=93144

 Score = 46.4 bits (50),  Expect = 0.053
 Identities = 96/139 (69%), Gaps = 7/139 (5%)
 Strand=Plus/Plus

Query  54   AAGAAGACGCTGGAGGTGGATTCGGCATGTCCTACGGAAA--GGCCAGGGGGATATCACA  111
            |||||| ||||||| ||||||| ||||     |  |||||  | ||| | |  |  ||||
Sbjct  418  AAGAAG-CGCTGGAAGTGGATTGGGCACTCTTTGAGGAAATCGCCCAAGTGCGT--CACA  474

Query  112  AAAACAGCACTCCACTGGACCCCAGAAGGTAAAAGAAAGAGAGGAAGACCTAAAATGACA  171
            | |  ||| |||   ||||  || ||||| ||||| |  ||||||||||| || |  |||
Sbjct  475  AGACAAGCCCTCACATGGAATCCTGAAGGCAAAAGGAGAAGAGGAAGACCAAAGAACACA  534

Query  172  TGGAG--AAGAACTGTAGA  188
            |  ||  ||||| || |||
Sbjct  535  TTAAGCCAAGAAATGGAGA  553


>ref|NW_003035682.1|  Schistosoma mansoni genome sequence supercontig Smp_scaff001942
Length=50903

 Score = 46.4 bits (50),  Expect = 0.053
 Identities = 45/58 (77%), Gaps = 0/58 (0%)
 Strand=Plus/Minus

Query  107    TCACAAAAACAGCACTCCACTGGACCCCAGAAGGTAAAAGAAAGAGAGGAAGACCTAA  164
              |||||| |  |||||||   ||||  || |||||| ||||||  ||||||||||| ||
Sbjct  28989  TCACAAGACAAGCACTCACATGGAATCCTGAAGGTCAAAGAAGAAGAGGAAGACCAAA  28932

>ref|NW_003033283.1|  Schistosoma mansoni genome sequence supercontig Smp_scaff003881
Length=1561

 Score = 46.4 bits (50),  Expect = 0.053
 Identities = 85/122 (69%), Gaps = 7/122 (5%)
 Strand=Plus/Minus

Query  54   AAGAAGACGCTGGAGGTGGATTCGGCATGTCCTACGGAAAGGCC---AGGGGGATATCAC  110
            |||||| ||||||| ||||||| ||||   |||  |||||   |   |  | |   ||||
Sbjct  955  AAGAAG-CGCTGGAAGTGGATTGGGCACACCCTGAGGAAATCACCTAATTGCG---TCAC  900

Query  111  AAAAACAGCACTCCACTGGACCCCAGAAGGTAAAAGAAAGAGAGGAAGACCTAAAATGAC  170
            || |  |||||||   ||||  || |||||| |||| |  ||||||||||| || |  ||
```

```
Sbjct  899  AAGACAAGCACTCACATGGAATCCTGAAGGTCAAAGGAGAAGAGGAAGACCAAAGAACAC  840

Query  171  AT  172
            ||
Sbjct  839  AT  838
```

>**ref|NW_003026471.1|** Schistosoma mansoni genome sequence supercontig Smp_scaff000224
Length=358445

Sort alignments for this subject sequence by:
E value **Score** **Percent identity**
Query start position **Subject start position**

Features flanking this part of subject sequence:
110898 bp at 5' side: hypothetical protein
18863 bp at 3' side: hypothetical protein

```
 Score = 46.4 bits (50),  Expect = 0.053
 Identities = 34/40 (85%), Gaps = 0/40 (0%)
 Strand=Plus/Plus

Query  127     TGGACCCCAGAAGGTAAAAGAAAGAGAGGAAGACCTAAAA  166
               ||||| || |||||| |||| || ||||||||||| ||||
Sbjct  196880  TGGACTCCTGAAGGTTAAAGGAAAAGAGGAAGACCAAAAA  196919
```

Features flanking this part of subject sequence:
24630 bp at 3' side: hypothetical protein

```
 Score = 39.2 bits (42),  Expect = 7.9
 Identities = 48/66 (72%), Gaps = 0/66 (0%)
 Strand=Plus/Minus

Query  107    TCACAAAAACAGCACTCCACTGGACCCCAGAAGGTAAAAGAAAGAGAGGAAGACCTAAAA  166
              |||||| |  ||| |||   ||||  || |||||| |||| |  ||||||||||| || |
Sbjct  13742  TCACAAGAGAAGCCCTCACATGGAATCCTGAAGGTCAAAGGAGAAGAGGAAGACCAAAGA  13683

Query  167    TGACAT  172
                ||||
Sbjct  13682  ACACAT  13677
```

Features flanking this part of subject sequence:
23365 bp at 3' side: hypothetical protein

```
 Score = 39.2 bits (42),  Expect = 7.9
 Identities = 48/66 (72%), Gaps = 0/66 (0%)
 Strand=Plus/Plus

Query  107    TCACAAAAACAGCACTCCACTGGACCCCAGAAGGTAAAAGAAAGAGAGGAAGACCTAAAA  166
              |||||| || ||| |||   ||||  || |||||  ||||||  ||||||||| | || |
Sbjct  14942  TCACAAGAAAAGCCCTCACATGGAATCCTGAAGGCCAAAGAAGAAGAGGAAGATCAAAGA  15001

Query  167    TGACAT  172
                ||||
Sbjct  15002  ACACAT  15007
```

>**ref|NW_003025039.1|** Schistosoma mansoni genome sequence supercontig Smp_scaff009302
Length=1500

```
 Score = 46.4 bits (50),  Expect = 0.053
 Identities = 52/67 (77%), Gaps = 2/67 (2%)
 Strand=Plus/Minus

Query  107  TCACAAAAACAGCACTCCAC-TGGACCCCAGAAGGTAAAAGAAAGAGAGGAAGACCTAAA  165
            |||||| |  ||| |||||| |||| ||| |||||  |||| | |||||||||||| ||
Sbjct  654  TCACAAGACAAGC-CTCCACATGGAACCCTGAAGGCCAAAGGAGGAGAGGAAGACCAAAG  596

Query  166  ATGACAT  172
            |  ||||
Sbjct  595  AACACAT  589
```

>**ref|NW_003022345.1|** Schistosoma mansoni genome sequence supercontig Smp_scaff013269
Length=2593

```
 Score = 46.4 bits (50),  Expect = 0.053
 Identities = 87/122 (71%), Gaps = 7/122 (5%)
 Strand=Plus/Plus

Query  54    AAGAAGACGCTGGAGGTGGATTCGGCATGTCCTACGGAAAGG--CCAGGGGGATATCACA  111
             |||||| ||||||| ||||||| |||||    |  |||||    ||||  |  | |||||
Sbjct  1427  AAGAAG-CGCTGGAAGTGGATTGGGCATACTTTGAGGAAATCACCCAGTTG--TGTCACA  1483

Query  112   AAAACAGCACTCCACTGGACCCCAGAAGGT-AAAAGAAAGAGAGGAAGACCTAAAATGAC  170
             | |  ||| |||   ||||  || |||||| |||||| | | ||||||||||| || |  ||
Sbjct  1484  AGACAAGCCCTCGCGTGGAATCCTGAAGGTCAAAAGAGA-AGAGGAAGACCAAAGAACAC  1542

Query  171   AT  172
             ||
Sbjct  1543  AT  1544
```

>**emb|FN359270.1|** Schistosoma mansoni genome sequence supercontig Smp_scaff001979
Length=185055

```
 Score = 46.4 bits (50),  Expect = 0.053
 Identities = 85/122 (69%), Gaps = 7/122 (5%)
 Strand=Plus/Minus

Query  54     AAGAAGACGCTGGAGGTGGATTCGGCATGTCCTACGGAAAGGCC---AGGGGGATATCAC  110
              |||||| ||||||| ||||||| ||||   |||  |||||   |   |  | |   ||||
Sbjct  56776  AAGAAG-CGCTGGAAGTGGATTGGGCACACCCTGAGGAAATCACCTAATTGCG---TCAC  56721

Query  111    AAAAACAGCACTCCACTGGACCCCAGAAGGTAAAAGAAAGAGAGGAAGACCTAAAATGAC  170
              || |  |||||||   ||||  || |||||| |||| |  ||||||||||| || |  ||
Sbjct  56720  AAGACAAGCACTCACATGGAATCCTGAAGGTCAAAGGAGAAGAGGAAGACCAAAGAACAC  56661

Query  171    AT  172
              ||
Sbjct  56660  AT  56659
```

>**emb|FN359226.1|** Schistosoma mansoni genome sequence supercontig Smp_scaff001935
Length=93144

```
 Score = 46.4 bits (50),  Expect = 0.053
 Identities = 96/139 (69%), Gaps = 7/139 (5%)
 Strand=Plus/Plus

Query  54   AAGAAGACGCTGGAGGTGGATTCGGCATGTCCTACGGAAA--GGCCAGGGGGATATCACA  111
            |||||| ||||||| ||||||| ||||     |  |||||    ||| | |  |  ||||
Sbjct  418  AAGAAG-CGCTGGAAGTGGATTGGGCACTCTTTGAGGAAATCGCCCAAGTGCGT--CACA  474

Query  112  AAAACAGCACTCCACTGGACCCCAGAAGGTAAAAGAAAGAGAGGAAGACCTAAAATGACA  171
            | |  ||| |||   ||||  || ||||| ||||| |  ||||||||||| || |  |||
Sbjct  475  AGACAAGCCCTCACATGGAATCCTGAAGGCAAAAGGAGAAGAGGAAGACCAAAGAACACA  534

Query  172  TGGAG--AAGAACTGTAGA  188
            |  ||  ||||| || |||
Sbjct  535  TTAAGCCAAGAAATGGAGA  553
```

>**emb|FN357618.1|** Schistosoma mansoni genome sequence supercontig Smp_scaff000327
Length=708014

Sort alignments for this subject sequence by:
E value **Score** **Percent identity**
Query start position **Subject start position**

Features in this part of subject sequence:
hypothetical protein

```
 Score = 46.4 bits (50),  Expect = 0.053
 Identities = 51/68 (75%), Gaps = 0/68 (0%)
 Strand=Plus/Minus

Query  105     TATCACAAAAACAGCACTCCACTGGACCCCAGAAGGTAAAAGAAAGAGAGGAAGACCTAA  164
               |||||||| |  ||| |||   ||||  || ||||||  ||| || ||||||||||| ||
Sbjct  199543  TATCACAAGACAAGCCCTCACGTGGAATCCTGAAGGTCGAAGGAAAAGAGGAAGACCAAA  199484

Query  165     AATGACAT  172
               ||  ||||
Sbjct  199483  AAACACAT  199476
```

Features in this part of subject sequence:
fibrillin-related

```
 Score = 39.2 bits (42),  Expect = 7.9
 Identities = 48/66 (72%), Gaps = 0/66 (0%)
 Strand=Plus/Minus

Query  107     TCACAAAAACAGCACTCCACTGGACCCCAGAAGGTAAAAGAAAGAGAGGAAGACCTAAAA  166
               |||||| |  ||| |||   |||| ||| |||||  ||||  | ||||||||||| || |
Sbjct  254086  TCACAAGACAAGCCCTCACATGGAACCCTGAAGGCCAAAGGTAAAGAGGAAGACCAAAGA  254027

Query  167     TGACAT  172
                 ||||
Sbjct  254026  ACACAT  254021
```

Features flanking this part of subject sequence:
41003 bp at 5' side: conserved hypothetical protein
37115 bp at 3' side: protocadherin gamma, putative

```
 Score = 39.2 bits (42),  Expect = 7.9
 Identities = 29/34 (85%), Gaps = 0/34 (0%)
 Strand=Plus/Minus

Query  133     CCAGAAGGTAAAAGAAAGAGAGGAAGACCTAAAA  166
               || ||||| ||| | |||||||||||||| ||||
Sbjct  656510  CCTGAAGGGAAACGGAAGAGAGGAAGACCAAAAA  656477
```

>**emb|FN357592.1|** Schistosoma mansoni genome sequence supercontig Smp_scaff000301
Length=345469

Features flanking this part of subject sequence:
901 bp at 5' side: tyrosine kinase

```
 Score = 46.4 bits (50),  Expect = 0.053
 Identities = 96/139 (69%), Gaps = 7/139 (5%)
 Strand=Plus/Minus
```

```
Query  54      AAGAAGACGCTGGAGGTGGATTCGGCATGTCCTACGGAAA--GGCCAGGGGGATATCACA  111
               |||||| ||||||| ||||||| ||||     |  |||||  | ||| | |  |  ||||
Sbjct  330840  AAGAAG-CGCTGGAAGTGGATTGGGCACTCTTTGAGGAAATCGCCCAAGTGCGT--CACA  330784

Query  112     AAAACAGCACTCCACTGGACCCCAGAAGGTAAAAGAAAGAGAGGAAGACCTAAAATGACA  171
               | |  ||| |||   ||||  || ||||| ||||| |  |||||||||||| || |  |||
Sbjct  330783  AGACAAGCCCTCACATGGAATCCTGAAGGCAAAAGGAGAAGAGGAAGACCAAAGAACACA  330724

Query  172     TGGAG--AAGAACTGTAGA  188
               |  ||  ||||| || |||
Sbjct  330723  TTAAGCCAAGAAATGGAGA  330705
```

>**emb|FN357351.1|** Schistosoma mansoni genome sequence supercontig Smp_scaff000060
Length=988485

Sort alignments for this subject sequence by:
E value **Score** **Percent identity**
Query start position **Subject start position**

Features flanking this part of subject sequence:
29064 bp at 5' side: hypothetical protein
1053 bp at 3' side: expressed protein

Score = 46.4 bits (50), Expect = 0.053
Identities = 43/55 (78%), Gaps = 0/55 (0%)
Strand=Plus/Minus

```
Query  118     GCACTCCACTGGACCCCAGAAGGTAAAAGAAAGAGAGGAAGACCTAAAATGACAT  172
               ||||||   ||||  || |||||  ||||||||||||||||||| || |  ||||
Sbjct  503744  GCACTCAGATGGAATCCTGAAGGCCAAAGAAAGAGAGGAAGACCAAAGAACACAT  503690
```

Features flanking this part of subject sequence:
59913 bp at 3' side: hypothetical protein

Score = 41.0 bits (44), Expect = 2.3
Identities = 49/67 (73%), Gaps = 0/67 (0%)
Strand=Plus/Minus

```
Query  106    ATCACAAAAACAGCACTCCACTGGACCCCAGAAGGTAAAAGAAAGAGAGGAAGACCTAAA  165
              ||||||||   | | |||   ||||  || |||||| |||| || ||||||||||| ||
Sbjct  82253  ATCACAAAGCAAACCCTCACATGGAATCCTGAAGGTCAAAGGAAAAGAGGAAGACCAAAG  82194

Query  166    ATGACAT  172
              |  ||||
Sbjct  82193  AACACAT  82187
```

Features flanking this part of subject sequence:
58336 bp at 3' side: hypothetical protein

Score = 41.0 bits (44), Expect = 2.3
Identities = 49/67 (73%), Gaps = 0/67 (0%)
Strand=Plus/Minus

```
Query  106    ATCACAAAAACAGCACTCCACTGGACCCCAGAAGGTAAAAGAAAGAGAGGAAGACCTAAA  165
              ||||||||   | | |||   ||||  || |||||| |||| || ||||||||||| ||
Sbjct  83830  ATCACAAAGCAAACCCTCACATGGAATCCTGAAGGTCAAAGGAAAAGAGGAAGACCAAAG  83771

Query  166    ATGACAT  172
              |  ||||
Sbjct  83770  AACACAT  83764
```

Features flanking this part of subject sequence:
15397 bp at 5' side: synbindin, putative
45448 bp at 3' side: Dentin sialophosphoprotein precursor [Contains: Dentin ph...

Score = 41.0 bits (44), Expect = 2.3
Identities = 49/67 (73%), Gaps = 0/67 (0%)
Strand=Plus/Plus

```
Query  106     ATCACAAAAACAGCACTCCACTGGACCCCAGAAGGTAAAAGAAAGAGAGGAAGACCTAAA  165
               |||||||    ||| |||   ||||  || |||||| |||| | |||||||||||| ||
Sbjct  895600  ATCACAAGGCAAGCCCTCACATGGAATCCTGAAGGTCAAAGGAGGAGAGGAAGACCAAAG  895659

Query  166     ATGACAT  172
               |  ||||
Sbjct  895660  AACACAT  895666
```

Features flanking this part of subject sequence:
35200 bp at 5' side: drug transporter, putative
5414 bp at 3' side: expressed protein

Score = 39.2 bits (42), Expect = 7.9
Identities = 48/66 (72%), Gaps = 0/66 (0%)
Strand=Plus/Minus

```
Query  107     TCACAAAAACAGCACTCCACTGGACCCCAGAAGGTAAAAGAAAGAGAGGAAGACCTAAAA  166
               |||||| |  |||||||  |||||  || |||||   || ||  ||||||||||| || |
Sbjct  564821  TCACAAGACAAGCACTCACCTGGAATCCTGAAGGCCGAAAAAGAAGAGGAAGACCGAAGA  564762

Query  167     TGACAT  172
                 ||||
Sbjct  564761  ACACAT  564756
```

```
 Features flanking this part of subject sequence:
   5686 bp at 5' side: hypothetical protein
   4722 bp at 3' side: expressed protein

 Score = 39.2 bits (42),  Expect = 7.9
 Identities = 48/66 (72%), Gaps = 0/66 (0%)
 Strand=Plus/Plus

Query  107     TCACAAAAACAGCACTCCACTGGACCCCAGAAGGTAAAAGAAAGAGAGGAAGACCTAAAA  166
               |||||| |  ||| |||   ||||  || |||||| |||| |  ||||||||||| || |
Sbjct  656596  TCACAAGACAAGCCCTCACATGGAATCCTGAAGGTCAAAGGAGAAGAGGAAGACCAAAGA  656655

Query  167     TGACAT  172
                 ||||
Sbjct  656656  ACACAT  656661


 Features flanking this part of subject sequence:
   49685 bp at 5' side: synbindin, putative
   11173 bp at 3' side: Dentin sialophosphoprotein precursor [Contains: Dentin ph...

 Score = 39.2 bits (42),  Expect = 7.9
 Identities = 43/55 (78%), Gaps = 2/55 (3%)
 Strand=Plus/Plus

Query  106     ATCACAAAAACAGCACTCCAC-TGGACCCCAGAAGGTAAAAGAAAGAGAGGAAGA  159
               ||||||| |  ||| || ||| ||||  || |||||| ||||||| ||| |||||
Sbjct  929888  ATCACAAGACAAGC-CTTCACATGGAATCCTGAAGGTCAAAGAAAAAGATGAAGA  929941


 Features flanking this part of subject sequence:
   57585 bp at 5' side: synbindin, putative
   3281 bp at 3' side: Dentin sialophosphoprotein precursor [Contains: Dentin ph...

 Score = 39.2 bits (42),  Expect = 7.9
 Identities = 36/46 (78%), Gaps = 0/46 (0%)
 Strand=Plus/Minus

Query  127     TGGACCCCAGAAGGTAAAAGAAAGAGAGGAAGACCTAAAATGACAT  172
               ||||  || |||||| |||| || ||||||||||| || |  ||||
Sbjct  937833  TGGAATCCTGAAGGTCAAAGGAAAAGAGGAAGACCAAAGAACACAT  937788


>emb|FN357335.1| D Schistosoma mansoni genome sequence supercontig Smp_scaff000044
Length=1160491

                                                            Sort alignments for this subject sequence by:
                                                              E value  Score  Percent identity
                                                              Query start position  Subject start position

 Features flanking this part of subject sequence:
   10353 bp at 5' side: expressed protein
   44203 bp at 3' side: 60S ribosomal protein L13 (BBC1 protein homolog), putative

 Score = 46.4 bits (50),  Expect = 0.053
 Identities = 55/75 (73%), Gaps = 0/75 (0%)
 Strand=Plus/Plus

Query  107     TCACAAAAACAGCACTCCACTGGACCCCAGAAGGTAAAAGAAAGAGAGGAAGACCTAAAA  166
               |||||| |  ||| |||   ||||| || ||||| ||||| |  ||||||||||| || |
Sbjct  221237  TCACAAGACAAGCCCTCACATGGACTCCTGAAGGCAAAAGGAGAAGAGGAAGACCAAAGA  221296

Query  167     TGACATGGAGAAGAA  181
                 ||||  || ||||
Sbjct  221297  ACACATTAAGCAGAA  221311


 Features flanking this part of subject sequence:
   37837 bp at 5' side: expressed protein
   16720 bp at 3' side: 60S ribosomal protein L13 (BBC1 protein homolog), putative

 Score = 39.2 bits (42),  Expect = 7.9
 Identities = 53/74 (71%), Gaps = 0/74 (0%)
 Strand=Plus/Plus

Query  107     TCACAAAAACAGCACTCCACTGGACCCCAGAAGGTAAAAGAAAGAGAGGAAGACCTAAAA  166
               |||||| |  ||| |||   ||||  || ||||| ||||| |  ||||||||| | ||||
Sbjct  248721  TCACAAGACAAGCCCTCATATGGAATCCTGAAGGCAAAAGCAGAAGAGGAAGATCAAAAA  248780

Query  167     TGACATGGAGAAGA  180
                 ||||  || |||
Sbjct  248781  ACACATTAAGCAGA  248794


 Features in this part of subject sequence:
   beta-hexosaminidase B, putative

 Score = 39.2 bits (42),  Expect = 7.9
 Identities = 48/66 (72%), Gaps = 0/66 (0%)
 Strand=Plus/Minus

Query  107     TCACAAAAACAGCACTCCACTGGACCCCAGAAGGTAAAAGAAAGAGAGGAAGACCTAAAA  166
               |||||| |  ||| |||   ||||  || |||||| |||| |  ||||||||||| || |
Sbjct  374038  TCACAAGACAAGCCCTCACATGGAATCCTGAAGGTCAAAGGAGAAGAGGAAGACCAAAGA  373979

Query  167     TGACAT  172
                 ||||
Sbjct  373978  ACACAT  373973
```

Features flanking this part of subject sequence:
22541 bp at 5' side: subfamily M12A unassigned peptidase (M12 family)
35229 bp at 3' side: malic enzyme, putative

Score = 39.2 bits (42), Expect = 7.9
Identities = 48/66 (72%), Gaps = 0/66 (0%)
Strand=Plus/Plus

```
Query  107      TCACAAAAACAGCACTCCACTGGACCCCAGAAGGTAAAAGAAAGAGAGGAAGACCTAAAA  166
                |||||| |  ||| |||   ||||  || |||||| |||| |  ||||||||||| || |
Sbjct  1004673  TCACAAGACAAGCCCTCACATGGAATCCTGAAGGTCAAAGGAGAAGAGGAAGACCAAAGA  1004732

Query  167      TGACAT  172
                  ||||
Sbjct  1004733  ACACAT  1004738
```

>**emb|FN357330.1|** Schistosoma mansoni genome sequence supercontig Smp_scaff000039
Length=1558831

Sort alignments for this subject sequence by:
E value **Score** **Percent identity**
Query start position **Subject start position**

Features flanking this part of subject sequence:
12490 bp at 5' side: serine/threonine kinase
56353 bp at 3' side: expressed protein

Score = 46.4 bits (50), Expect = 0.053
Identities = 51/67 (76%), Gaps = 2/67 (2%)
Strand=Plus/Plus

```
Query  105      TATCACAAAAACAGCACTCCACTGGACCCCAGAAGGTAAAAGAAAGAGAGGAAGACCTAA  164
                |||||||| |  ||| |||   ||||  || |||||  |||| || |||||||||||  |
Sbjct  1133666  TATCACAAGACAAGCCCTCACATGGAATCCTGAAGGCTAAAGTAAAAGAGGAAGACC--A  1133723

Query  165      AATGACA  171
                |||||||
Sbjct  1133724  AATGACA  1133730
```

Features in this part of subject sequence:
low-density lipoprotein receptor (ldl)

Score = 44.6 bits (48), Expect = 0.18
Identities = 45/59 (76%), Gaps = 0/59 (0%)
Strand=Plus/Minus

```
Query  107     TCACAAAAACAGCACTCCACTGGACCCCAGAAGGTAAAAGAAAGAGAGGAAGACCTAAA  165
               |||||| |  |||||||   ||||  ||  ||||  ||||||| ||||||||||| |||
Sbjct  316945  TCACAAGACAAGCACTCGCTTGGAATCCCCAAGGCCAAAGAAAAAGAGGAAGACCAAAA  316887
```

Features flanking this part of subject sequence:
42771 bp at 5' side: hypothetical protein
7950 bp at 3' side: expressed protein

Score = 44.6 bits (48), Expect = 0.18
Identities = 48/64 (75%), Gaps = 0/64 (0%)
Strand=Plus/Plus

```
Query  109     ACAAAAACAGCACTCCACTGGACCCCAGAAGGTAAAAGAAAGAGAGGAAGACCTAAAATG  168
               ||||||| ||| |||   | ||  || |||||| |||| || ||||||||||| || |
Sbjct  635388  ACAAAAAAAGCCCTCACATAGAATCCTGAAGGTCAAAGGAAAAGAGGAAGACCAAAGAAC  635447

Query  169     ACAT  172
               ||||
Sbjct  635448  ACAT  635451
```

Features flanking this part of subject sequence:
42666 bp at 5' side: 5'-3' exoribonuclease, putative
27062 bp at 3' side: expressed protein

Score = 39.2 bits (42), Expect = 7.9
Identities = 36/46 (78%), Gaps = 0/46 (0%)
Strand=Plus/Minus

```
Query  127     TGGACCCCAGAAGGTAAAAGAAAGAGAGGAAGACCTAAAATGACAT  172
               ||||  || |||||| |||| || ||||||||||| || |  ||||
Sbjct  173868  TGGAATCCTGAAGGTCAAAGGAAAAGAGGAAGACCAAAGAACACAT  173823
```

Features flanking this part of subject sequence:
3643 bp at 5' side: low-density lipoprotein receptor (ldl)
99502 bp at 3' side: ccr4-not transcription complex, putative

Score = 39.2 bits (42), Expect = 7.9
Identities = 48/66 (72%), Gaps = 0/66 (0%)
Strand=Plus/Minus

```
Query  107     TCACAAAAACAGCACTCCACTGGACCCCAGAAGGTAAAAGAAAGAGAGGAAGACCTAAAA  166
               |||||| |  ||| |||   ||||  || |||||| |||| |  ||||||||||| || |
Sbjct  369600  TCACAAGACAAGCCCTCACATGGAATCCTGAAGGTCAAAGGAGAAGAGGAAGACCAAAGA  369541

Query  167     TGACAT  172
                 ||||
```

```
Sbjct  369540  ACACAT  369535
```

Features flanking this part of subject sequence:
30905 bp at 5' side: low-density lipoprotein receptor (ldl)
72240 bp at 3' side: ccr4-not transcription complex, putative

Score = 39.2 bits (42), Expect = 7.9
Identities = 48/66 (72%), Gaps = 0/66 (0%)
Strand=Plus/Plus

```
Query  107     TCACAAAAACAGCACTCCACTGGACCCCAGAAGGTAAAAGAAAGAGAGGAAGACCTAAAA  166
               |||||| |  ||| |||   ||||  || |||||| |||| |  ||||||||||| || |
Sbjct  396797  TCACAAGACAAGCCCTCACATGGAATCCTGAAGGTCAAAGGAGAAGAGGAAGACCAAAGA  396856

Query  167     TGACAT  172
                 ||||
Sbjct  396857  ACACAT  396862
```

Features flanking this part of subject sequence:
36952 bp at 5' side: hypothetical protein
13792 bp at 3' side: expressed protein

Score = 39.2 bits (42), Expect = 7.9
Identities = 33/41 (80%), Gaps = 0/41 (0%)
Strand=Plus/Plus

```
Query  132     CCCAGAAGGTAAAAGAAAGAGAGGAAGACCTAAAATGACAT  172
               ||| |||||| |||| || ||||||||||| || |  ||||
Sbjct  629569  CCCTGAAGGTCAAAGGAAAAGAGGAAGACCAAAGAACACAT  629609
```

Features flanking this part of subject sequence:
70656 bp at 5' side: conserved hypothetical protein
126979 bp at 3' side: hypothetical protein

Score = 39.2 bits (42), Expect = 7.9
Identities = 48/66 (72%), Gaps = 0/66 (0%)
Strand=Plus/Minus

```
Query  107     TCACAAAAACAGCACTCCACTGGACCCCAGAAGGTAAAAGAAAGAGAGGAAGACCTAAAA  166
               |||||| |  ||| |||   ||||  || |||||| |||| |  ||||||||||| || |
Sbjct  843930  TCACAAGACAAGCCCTCGCATGGAATCCTGAAGGTCAAAGGAGAAGAGGAAGACCAAAGA  843871

Query  167     TGACAT  172
                 ||||
Sbjct  843870  ACACAT  843865
```

Features flanking this part of subject sequence:
9203 bp at 5' side: hypothetical protein
8385 bp at 3' side: restin-like

Score = 39.2 bits (42), Expect = 7.9
Identities = 48/66 (72%), Gaps = 0/66 (0%)
Strand=Plus/Plus

```
Query  107     TCACAAAAACAGCACTCCACTGGACCCCAGAAGGTAAAAGAAAGAGAGGAAGACCTAAAA  166
               |||||| |  ||| |||   ||||  || |||||| |||| |  ||||||||||| || |
Sbjct  980405  TCACAAGACAAGCCCTCACATGGAATCCTGAAGGTCAAAGGAGAAGAGGAAGACCAAAGA  980464

Query  167     TGACAT  172
                 ||||
Sbjct  980465  ACACAT  980470
```

Features flanking this part of subject sequence:
800 bp at 5' side: ribonucleoprotein-related
4538 bp at 3' side: ankyrin repeat-containing, putative

Score = 39.2 bits (42), Expect = 7.9
Identities = 48/66 (72%), Gaps = 0/66 (0%)
Strand=Plus/Minus

```
Query  107      TCACAAAAACAGCACTCCACTGGACCCCAGAAGGTAAAAGAAAGAGAGGAAGACCTAAAA  166
                |||||| |  ||| |||   ||||  || |||||| |||| |  ||||||||||| || |
Sbjct  1308834  TCACAACACAAGCCCTCACATGGAATCCTGAAGGTCAAAGGAGAAGAGGAAGACCAAAGA  1308775

Query  167      TGACAT  172
                  ||||
Sbjct  1308774  ACACAT  1308769
```

>**emb|FN357299.1|** D Schistosoma mansoni genome sequence supercontig Smp_scaff000008
Length=2460020

Sort alignments for this subject sequence by:
E value **Score** **Percent identity**
Query start position **Subject start position**

Features flanking this part of subject sequence:
23757 bp at 5' side: egg protein CP391S, putative
362 bp at 3' side: aquaglyceroporin, putative

Score = 46.4 bits (50), Expect = 0.053
Identities = 45/58 (77%), Gaps = 0/58 (0%)
Strand=Plus/Plus

```
Query  107     TCACAAAAACAGCACTCCACTGGACCCCAGAAGGTAAAAGAAAGAGAGGAAGACCTAA  164
               |||||| |  ||| |||   ||||  || |||||| ||||||| ||||||||||| ||
Sbjct  589012  TCACAAGACAAGCCCTCACATGGAATCCTGAAGGTCAAAGAAAAAGAGGAAGACCAAA  589069
```

Features flanking this part of subject sequence:
32925 bp at 5' side: expressed protein
15902 bp at 3' side: hypothetical protein

Score = 42.8 bits (46), Expect = 0.65
Identities = 49/66 (74%), Gaps = 0/66 (0%)
Strand=Plus/Minus

```
Query  107      TCACAAAAACAGCACTCCACTGGACCCCAGAAGGTAAAAGAAAGAGAGGAAGACCTAAAA  166
                |||||| |  ||| |||   ||||  || |||||| |||| ||||| |||||||| || |
Sbjct  1572064  TCACAAGACAAGCCCTCACATGGAATCCTGAAGGTCAAAGGAAGAGTGGAAGACCAAAGA  1572005

Query  167      TGACAT  172
                  ||||
Sbjct  1572004  ACACAT  1571999
```

Features flanking this part of subject sequence:
64875 bp at 5' side: cadherin, putative
37126 bp at 3' side: conserved hypothetical protein

Score = 42.8 bits (46), Expect = 0.65
Identities = 84/121 (69%), Gaps = 5/121 (4%)
Strand=Plus/Plus

```
Query  54       AAGAAGACGCTGGAGGTGGATTCGGCATGTCCTACGGAAAGG--CCAGGGGGATATCACA  111
                |||||| ||||||| ||||||| ||||     ||  |||||     |||    |  | |||||
Sbjct  2403851  AAGAAG-CGCTGGAAGTGGATTGGGCACACTCTGAGGAAATCACCCAATTG--TGTCACA  2403907

Query  112      AAAACAGCACTCCACTGGACCCCAGAAGGTAAAAGAAAGAGAGGAAGACCTAAAATGACA  171
                | |  ||| |||   ||||  || |||||| |||| |  ||||||||||| || |  |||
Sbjct  2403908  AGACAAGCCCTCACATGGAATCCTGAAGGTCAAAGGAGAAGAGGAAGACCAAAGAACACA  2403967

Query  172      T  172
                |
Sbjct  2403968  T  2403968
```

Features flanking this part of subject sequence:
80286 bp at 5' side: cadherin, putative
21767 bp at 3' side: conserved hypothetical protein

Score = 42.8 bits (46), Expect = 0.65
Identities = 49/66 (74%), Gaps = 0/66 (0%)
Strand=Plus/Plus

```
Query  107      TCACAAAAACAGCACTCCACTGGACCCCAGAAGGTAAAAGAAAGAGAGGAAGACCTAAAA  166
                ||||||||  ||| |||   ||||  || ||||||  ||| || ||||| ||||| ||||
Sbjct  2419262  TCACAAAACAAGCCCTCACATGGAGTCCTGAAGGTCGAAGGAAAAGAGGGAGACCAAAAA  2419321

Query  167      TGACAT  172
                  ||||
Sbjct  2419322  ACACAT  2419327
```

Features flanking this part of subject sequence:
9620 bp at 5' side: rab11, putative
4790 bp at 3' side: hypothetical protein

Score = 41.0 bits (44), Expect = 2.3
Identities = 49/67 (73%), Gaps = 0/67 (0%)
Strand=Plus/Minus

```
Query  106     ATCACAAAAACAGCACTCCACTGGACCCCAGAAGGTAAAAGAAAGAGAGGAAGACCTAAA  165
               |||||||||  | | |||   ||||  || |||||| |||| |  ||||||||||| ||
Sbjct  419704  ATCACAAAACAATCCCTCACATGGAATCCTGAAGGTCAAAGGAGAAGAGGAAGACCAAAG  419645

Query  166     ATGACAT  172
               |  ||||
Sbjct  419644  AACACAT  419638
```

Features flanking this part of subject sequence:
2373 bp at 5' side: expressed protein
2151 bp at 3' side: expressed protein

Score = 39.2 bits (42), Expect = 7.9
Identities = 48/66 (72%), Gaps = 0/66 (0%)
Strand=Plus/Minus

```
Query  107     TCACAAAAACAGCACTCCACTGGACCCCAGAAGGTAAAAGAAAGAGAGGAAGACCTAAAA  166
               |||||| |  ||| ||| | ||||  ||  ||||| |||| |  ||||||||||| || |
Sbjct  763388  TCACAAGACAAGCCCTCAAATGGAATCCTAAAGGTCAAAGGAGAAGAGGAAGACCAAAGA  763329

Query  167     TGACAT  172
                 ||||
Sbjct  763328  ACACAT  763323
```

Features in this part of subject sequence:
hypothetical protein

Score = 39.2 bits (42), Expect = 7.9
Identities = 48/66 (72%), Gaps = 0/66 (0%)

Strand=Plus/Minus

```
Query  107      TCACAAAAACAGCACTCCACTGGACCCCAGAAGGTAAAAGAAAGAGAGGAAGACCTAAAA  166
                |||||| |  ||| |||   ||||  || |||||| |||| |  ||||||||||| || |
Sbjct  2110691  TCACAAGACAAGCCCTCACATGGAATCCTGAAGGTCAAAGGAGAAGAGGAAGACCAAAGA  2110632

Query  167      TGACAT  172
                  ||||
Sbjct  2110631  ACACAT  2110626
```

Features in this part of subject sequence:
cadherin, putative

Score = 39.2 bits (42), Expect = 7.9
Identities = 48/66 (72%), Gaps = 0/66 (0%)
Strand=Plus/Minus

```
Query  107      TCACAAAAACAGCACTCCACTGGACCCCAGAAGGTAAAAGAAAGAGAGGAAGACCTAAAA  166
                |||||| |  ||| |||   ||||  || |||||| |||| |  ||||||||||| || |
Sbjct  2290134  TCACAAGACAAGCCCTCACATGGAATCCTGAAGGTCAAAGGAGAAGAGGAAGACCAAAGA  2290075

Query  167      TGACAT  172
                  ||||
Sbjct  2290074  ACACAT  2290069
```

>**emb|FN359233.1|** Schistosoma mansoni genome sequence supercontig Smp_scaff001942
Length=50903

Score = 46.4 bits (50), Expect = 0.053
Identities = 45/58 (77%), Gaps = 0/58 (0%)
Strand=Plus/Minus

```
Query  107    TCACAAAAACAGCACTCCACTGGACCCCAGAAGGTAAAAGAAAGAGAGGAAGACCTAA  164
              |||||| |  |||||||   ||||  || |||||| ||||||  ||||||||||| ||
Sbjct  28989  TCACAAGACAAGCACTCACATGGAATCCTGAAGGTCAAAGAAGAAGAGGAAGACCAAA  28932
```

>**emb|FN361172.1|** Schistosoma mansoni genome sequence supercontig Smp_scaff003881
Length=1561

Score = 46.4 bits (50), Expect = 0.053
Identities = 85/122 (69%), Gaps = 7/122 (5%)
Strand=Plus/Minus

```
Query  54   AAGAAGACGCTGGAGGTGGATTCGGCATGTCCTACGGAAAGGCC---AGGGGGATATCAC  110
            |||||| ||||||| ||||||| ||||   |||  |||||   |   |  | |   ||||
Sbjct  955  AAGAAG-CGCTGGAAGTGGATTGGGCACACCCTGAGGAAATCACCTAATTGCG---TCAC  900

Query  111  AAAAACAGCACTCCACTGGACCCCAGAAGGTAAAAGAAAGAGAGGAAGACCTAAAATGAC  170
            || |  |||||||   ||||  || |||||| |||| |  ||||||||||| || |  ||
Sbjct  899  AAGACAAGCACTCACATGGAATCCTGAAGGTCAAAGGAGAAGAGGAAGACCAAAGAACAC  840

Query  171  AT  172
            ||
Sbjct  839  AT  838
```

>**emb|FN357515.1|** Schistosoma mansoni genome sequence supercontig Smp_scaff000224
Length=358445

Sort alignments for this subject sequence by:
E value **Score** **Percent identity**
Query start position **Subject start position**

Features flanking this part of subject sequence:
110898 bp at 5' side: hypothetical protein
18863 bp at 3' side: hypothetical protein

Score = 46.4 bits (50), Expect = 0.053
Identities = 34/40 (85%), Gaps = 0/40 (0%)
Strand=Plus/Plus

```
Query  127     TGGACCCCAGAAGGTAAAAGAAAGAGAGGAAGACCTAAAA  166
               ||||| || |||||| |||| || ||||||||||| ||||
Sbjct  196880  TGGACTCCTGAAGGTTAAAGGAAAAGAGGAAGACCAAAAA  196919
```

Features flanking this part of subject sequence:
24630 bp at 3' side: hypothetical protein

Score = 39.2 bits (42), Expect = 7.9
Identities = 48/66 (72%), Gaps = 0/66 (0%)
Strand=Plus/Minus

```
Query  107    TCACAAAAACAGCACTCCACTGGACCCCAGAAGGTAAAAGAAAGAGAGGAAGACCTAAAA  166
              |||||| |  ||| |||   ||||  || |||||| |||| |  ||||||||||| || |
Sbjct  13742  TCACAAGAGAAGCCCTCACATGGAATCCTGAAGGTCAAAGGAGAAGAGGAAGACCAAAGA  13683

Query  167    TGACAT  172
                ||||
Sbjct  13682  ACACAT  13677
```

Features flanking this part of subject sequence:
23365 bp at 3' side: hypothetical protein

```
 Score = 39.2 bits (42),  Expect = 7.9
 Identities = 48/66 (72%), Gaps = 0/66 (0%)
 Strand=Plus/Plus

Query  107    TCACAAAAACAGCACTCCACTGGACCCCAGAAGGTAAAAGAAAGAGAGGAAGACCTAAAA  166
              |||||| || ||| |||   ||||  || |||||  ||||||  ||||||||| | || |
Sbjct  14942  TCACAAGAAAAGCCCTCACATGGAATCCTGAAGGCCAAAGAAGAAGAGGAAGATCAAAGA  15001

Query  167    TGACAT  172
                ||||
Sbjct  15002  ACACAT  15007
```

```
>emb|FN366593.1|  Schistosoma mansoni genome sequence supercontig Smp_scaff009302
Length=1500

 Score = 46.4 bits (50),  Expect = 0.053
 Identities = 52/67 (77%), Gaps = 2/67 (2%)
 Strand=Plus/Minus

Query  107  TCACAAAAACAGCACTCCAC-TGGACCCCAGAAGGTAAAAGAAAGAGAGGAAGACCTAAA  165
            |||||| |  ||| |||||| |||| ||| |||||  |||| | |||||||||||| || 
Sbjct  654  TCACAAGACAAGC-CTCCACATGGAACCCTGAAGGCCAAAGGAGGAGAGGAAGACCAAAG  596

Query  166  ATGACAT  172
            |  ||||
Sbjct  595  AACACAT  589
```

```
>emb|FN370560.1|  Schistosoma mansoni genome sequence supercontig Smp_scaff013269
Length=2593

 Score = 46.4 bits (50),  Expect = 0.053
 Identities = 87/122 (71%), Gaps = 7/122 (5%)
 Strand=Plus/Plus

Query  54    AAGAAGACGCTGGAGGTGGATTCGGCATGTCCTACGGAAAGG--CCAGGGGGATATCACA  111
             |||||| ||||||| ||||||| |||||    |  |||||    ||||  |  | |||||
Sbjct  1427  AAGAAG-CGCTGGAAGTGGATTGGGCATACTTTGAGGAAATCACCCAGTTG--TGTCACA  1483

Query  112   AAAACAGCACTCCACTGGACCCCAGAAGGT-AAAAGAAAGAGAGGAAGACCTAAAATGAC  170
             | |  ||| |||   ||||  || |||||| |||||| | ||||||||||| || |  ||
Sbjct  1484  AGACAAGCCCTCGCGTGGAATCCTGAAGGTCAAAAGAGA-AGAGGAAGACCAAAGAACAC  1542

Query  171   AT  172
             ||
Sbjct  1543  AT  1544
```

```
>ref|XM_001317411.1|  Trichomonas vaginalis G3 AT hook motif family protein (TVAG_474020)
partial mRNA
Length=1779

 GENE ID: 4763089 TVAG_474020 | AT hook motif family protein
[Trichomonas vaginalis G3] (10 or fewer PubMed links)

 Score = 46.4 bits (50),  Expect = 0.053
 Identities = 28/30 (93%), Gaps = 0/30 (0%)
 Strand=Plus/Plus

Query  134   CAGAAGGTAAAAGAAAGAGAGGAAGACCTA  163
             ||||||  ||||||||||||||||||||||
Sbjct  1397  CAGAAGTCAAAAGAAAGAGAGGAAGACCTA  1426
```

```
>ref|NW_003039011.1|  Schistosoma mansoni genome sequence supercontig Smp_scaff010546
Length=15387

 Score = 44.6 bits (48),  Expect = 0.18
 Identities = 77/111 (69%), Gaps = 1/111 (0%)
 Strand=Plus/Minus

Query  54    AAGAAGACGCTGGAGGTGGATTCGGCATGTCCTACGGAAAGGCCAGGGGGATATCACAAA  113
             |||||| ||||||| ||||||| ||||   | |  |||||   |  |     ||||||| 
Sbjct  6778  AAGAAG-CGCTGGAAGTGGATTGGGCACACCTTGAGGAAATCACCCGACAGCATCACAAG  6720

Query  114   AACAGCACTCCACTGGACCCCAGAAGGTAAAAGAAAGAGAGGAAGACCTAA  164
             |  ||| |||   ||||  || |||||| |||| |  ||||||||||| ||
Sbjct  6719  ACAAGCCCTCACATGGAATCCCGAAGGTCAAAGGAGAAGAGGAAGACCAAA  6669
```

```
>ref|NW_003033823.1|  Schistosoma mansoni genome sequence supercontig Smp_scaff010714
Length=3224

 Score = 44.6 bits (48),  Expect = 0.18
 Identities = 86/124 (69%), Gaps = 5/124 (4%)
 Strand=Plus/Minus

Query  51    AATAAGAAGACGCTGGAGGTGGATTCGGCATGTCCTACGGAAA--GGCCAGGGGGATATC  108
             |||| ||| |||||||| ||||||| ||||   | |  |||||   || |   |    ||
Sbjct  2786  AATACGAA-ACGCTGGAAGTGGATTGGGCACACCTTGAGGAAATCAGCTAATTG--CGTC  2730

Query  109   ACAAAAACAGCACTCCACTGGACCCCAGAAGGTAAAAGAAAGAGAGGAAGACCTAAAATG  168
             |||| |  ||| |||   ||||  || || ||| |||| |||||||||||||| || |  
Sbjct  2729  ACAAGACAAGCCCTCACATGGAATCCTGATGGTCAAAGGAAGAGAGGAAGACCAAAGAAC  2670

Query  169   ACAT  172
             ||||
```

```
Sbjct  2669  ACAT  2666
```

>**ref|NW_003026315.1|** Schistosoma mansoni genome sequence supercontig Smp_scaff002909
Length=2872

```
 Score = 44.6 bits (48),  Expect = 0.18
 Identities = 82/119 (68%), Gaps = 1/119 (0%)
 Strand=Plus/Minus

Query  54   AAGAAGACGCTGGAGGTGGATTCGGCATGTCCTACGGAAAGGCCAGGGGGATATCACAAA  113
            |||||| ||||||| ||||||| ||||   | |  |||||   | |       ||||||
Sbjct  679  AAGAAG-CGCTGGAAGTGGATTGGGCACACCTTGAGGAAATCACCGAATTGCGTCACAAT  621

Query  114  AACAGCACTCCACTGGACCCCAGAAGGTAAAAGAAAGAGAGGAAGACCTAAAATGACAT  172
            |  ||| |||   ||||  ||||||||| |||| |  ||||||||||| || |  ||||
Sbjct  620  ACAAGCCCTCACATGGAATCCAGAAGGTCAAAGGAGAAGAGGAAGACCAAAGAACACAT  562
```

>**emb|FN367837.1|** Schistosoma mansoni genome sequence supercontig Smp_scaff010546
Length=15387

```
 Score = 44.6 bits (48),  Expect = 0.18
 Identities = 77/111 (69%), Gaps = 1/111 (0%)
 Strand=Plus/Minus

Query  54    AAGAAGACGCTGGAGGTGGATTCGGCATGTCCTACGGAAAGGCCAGGGGGATATCACAAA  113
             |||||| ||||||| ||||||| ||||   | |  |||||   |  |     |||||||
Sbjct  6778  AAGAAG-CGCTGGAAGTGGATTGGGCACACCTTGAGGAAATCACCCGACAGCATCACAAG  6720

Query  114   AACAGCACTCCACTGGACCCCAGAAGGTAAAAGAAAGAGAGGAAGACCTAA  164
             |  ||| |||   ||||  || |||||| |||| |  ||||||||||| ||
Sbjct  6719  ACAAGCCCTCACATGGAATCCCGAAGGTCAAAGGAGAAGAGGAAGACCAAA  6669
```

>**emb|FN357707.1|** Schistosoma mansoni genome sequence supercontig Smp_scaff000416
Length=546141

Sort alignments for this subject sequence by:
E value **Score** **Percent identity**
Query start position **Subject start position**

Features in this part of subject sequence:
Protein C10orf118 (CTCL tumor antigen HD-CL-01/L14-2), pu...

```
 Score = 44.6 bits (48),  Expect = 0.18
 Identities = 45/59 (76%), Gaps = 0/59 (0%)
 Strand=Plus/Minus

Query  106     ATCACAAAAACAGCACTCCACTGGACCCCAGAAGGTAAAAGAAAGAGAGGAAGACCTAA  164
               ||||||| |  ||| |||   ||||  || |||||| ||||||  ||||||||||| ||
Sbjct  282007  ATCACAAGACAAGCCCTCACATGGAATCCTGAAGGTCAAAGAAGAAGAGGAAGACCAAA  281949
```

Features flanking this part of subject sequence:
53317 bp at 5' side: gamma-glutamyltransferase-like protein 3 (T03 family)
14926 bp at 3' side: paired box protein pax-6, putative

```
 Score = 44.6 bits (48),  Expect = 0.18
 Identities = 60/84 (71%), Gaps = 0/84 (0%)
 Strand=Plus/Plus

Query  107     TCACAAAAACAGCACTCCACTGGACCCCAGAAGGTAAAAGAAAGAGAGGAAGACCTAAAA  166
               |||||| || ||| |||   ||||  || |||||| |||  || ||||||| ||| ||||
Sbjct  486918  TCACAAGAAAAGCCCTCACATGGAATCCTGAAGGTCAAACGAAAAGAGGAAAACCAAAAA  486977

Query  167     TGACATGGAGAAGAACTGTAGAGG  190
                   ||| | || ||||| | |||
Sbjct  486978  AAGGATGAATAACAACTGAAAAGG  487001
```

Features in this part of subject sequence:
Protein C10orf118 (CTCL tumor antigen HD-CL-01/L14-2), pu...

```
 Score = 39.2 bits (42),  Expect = 7.9
 Identities = 48/66 (72%), Gaps = 0/66 (0%)
 Strand=Plus/Minus

Query  107     TCACAAAAACAGCACTCCACTGGACCCCAGAAGGTAAAAGAAAGAGAGGAAGACCTAAAA  166
               |||||| |  ||| |||   ||||  || |||||| |||| |  ||||||||||| || |
Sbjct  276780  TCACAAGACAAGCCCTCACATGGAATCCTGAAGGTCAAAGGAGAAGAGGAAGACCAAAGA  276721

Query  167     TGACAT  172
                 ||||
Sbjct  276720  ACACAT  276715
```

>**emb|FN357689.1|** Schistosoma mansoni genome sequence supercontig Smp_scaff000398
Length=248964

Features in this part of subject sequence:
expressed protein

```
 Score = 44.6 bits (48),  Expect = 0.18
 Identities = 82/119 (68%), Gaps = 1/119 (0%)
 Strand=Plus/Plus

Query  54      AAGAAGACGCTGGAGGTGGATTCGGCATGTCCTACGGAAAGGCCAGGGGGATATCACAAA  113
```

```
                 |||||| ||||||| ||||||| ||||   || |  |||||    |        | ||||||
Sbjct  235823  AAGAAG-CGCTGGAAGTGGATTGGGCACATCTTGAGGAAATCACCTAATTGTGTCACAAG  235881

Query  114     AACAGCACTCCACTGGACCCCAGAAGGTAAAAGAAAGAGAGGAAGACCTAAAATGACAT  172
               |  ||| |||    ||||   || |||||| ||||  |  ||||||||||| || |  ||||
Sbjct  235882  ACAAGCCCTCACATGGAATCCTGAAGGTCAAAGGAGAAGAGGAAGACCAAACAACACAT  235940
```

>**emb|FN357447.1|** D Schistosoma mansoni genome sequence supercontig Smp_scaff000156
Length=1261525

Sort alignments for this subject sequence by:
E value **Score** **Percent identity**
Query start position **Subject start position**

Features in this part of subject sequence:
expressed protein

```
 Score = 44.6 bits (48),  Expect = 0.18
 Identities = 82/119 (68%), Gaps = 1/119 (0%)
 Strand=Plus/Minus

Query  54      AAGAAGACGCTGGAGGTGGATTCGGCATGTCCTACGGAAAGGCCAGGGGGATATCACAAA  113
               |||||| ||||||| ||||||| ||||    | |  |||||    |   |     | ||||||
Sbjct  250979  AAGAAG-CGCTGGAAGTGGATTGGGCACACCTTGAGGAAATCACCTGATTGTGTCACAAG  250921

Query  114     AACAGCACTCCACTGGACCCCAGAAGGTAAAAGAAAGAGAGGAAGACCTAAAATGACAT  172
               |  ||| |||    ||||   || |||||| ||||  |  ||||||||||| || |  ||||
Sbjct  250920  ACAAGCCCTCACATGGAATCCTGAAGGTCAAAGGAGAAGAGGAAGACCAAAGAACACAT  250862
```

Features in this part of subject sequence:
hypothetical protein

```
 Score = 39.2 bits (42),  Expect = 7.9
 Identities = 74/108 (68%), Gaps = 1/108 (0%)
 Strand=Plus/Minus

Query  54     AAGAAGACGCTGGAGGTGGATTCGGCATGTCCTACGGAAAGGCCAGGGGGATATCACAAA  113
              |||||| ||||||| ||||||| ||||      |   |||||    |   |    | ||||||
Sbjct  78002  AAGAAG-CGCTGGAAGTGGATTGGGCACACTTTGAGGAAATCACCTAGTTGTGTCACAAG  77944

Query  114    AACAGCACTCCACTGGACCCCAGAAGGTAAAAGAAAGAGAGGAAGACC  161
              |  ||| |||    ||||   || |||||| ||||  |  |||||||||||
Sbjct  77943  ACAAGCCCTCACATGGAATCCTGAAGGTCAAAGGAGAAGAGGAAGACC  77896
```

>**emb|FN357444.1|** D Schistosoma mansoni genome sequence supercontig Smp_scaff000153
Length=872276

Sort alignments for this subject sequence by:
E value **Score** **Percent identity**
Query start position **Subject start position**

Features flanking this part of subject sequence:
3277 bp at 5' side: expressed protein
63120 bp at 3' side: hypothetical protein

```
 Score = 44.6 bits (48),  Expect = 0.18
 Identities = 86/124 (69%), Gaps = 5/124 (4%)
 Strand=Plus/Minus

Query  51      AATAAGAAGACGCTGGAGGTGGATTCGGCATGTCCTACGGAAA--GGCCAGGGGGATATC  108
               |||| ||| |||||||| ||||||| ||||    | |  |||||    || |    |     ||
Sbjct  102005  AATACGAA-ACGCTGGAAGTGGATTGGGCACACCTTGAGGAAATCAGCTAATTG--CGTC  101949

Query  109     ACAAAAACAGCACTCCACTGGACCCCAGAAGGTAAAAGAAAGAGAGGAAGACCTAAAATG  168
               |||| |  ||| |||    ||||   || || ||||| |||| ||||||||||||| || |
Sbjct  101948  ACAAGACAAGCCCTCACATGGAATCCTGATGGTCAAAGGAAGAGAGGAAGACCAAAGAAC  101889

Query  169     ACAT  172
               ||||
Sbjct  101888  ACAT  101885
```

Features flanking this part of subject sequence:
11642 bp at 5' side: cyclic-nucleotide-gated cation channel, putative
43581 bp at 3' side: cyclic-nucleotide-gated cation channel, putative

```
 Score = 41.0 bits (44),  Expect = 2.3
 Identities = 49/67 (73%), Gaps = 0/67 (0%)
 Strand=Plus/Plus

Query  106     ATCACAAAAACAGCACTCCACTGGACCCCAGAAGGTAAAAGAAAGAGAGGAAGACCTAAA  165
               ||||||| |  ||| |||    ||||  || |||||   |||| || ||||||||||| ||
Sbjct  579578  ATCACAAGACAAGCCCTCACATGGAATCCTGAAGGCCAAAGGAAAAGAGGAAGACCGAAG  579637

Query  166     ATGACAT  172
               |  ||||
Sbjct  579638  AACACAT  579644
```

>**emb|FN357435.1|** D Schistosoma mansoni genome sequence supercontig Smp_scaff000144
Length=974603

Sort alignments for this subject sequence by:
E value **Score** **Percent identity**
Query start position **Subject start position**

Features flanking this part of subject sequence:
5246 bp at 5' side: ER lumen protein retaining receptor, putative
15745 bp at 3' side: Adenine phosphoribosyltransferase, putative

```
 Score = 44.6 bits (48),  Expect = 0.18
 Identities = 77/109 (70%), Gaps = 5/109 (4%)
 Strand=Plus/Plus

Query  54      AAGAAGACGCTGGAGGTGGATTCGGCATGTCCTACGGAAAGG--CCAGGGGGATATCACA  111
               |||||| ||||||| ||||||| |||||  | |  |||||    |||      | |||||
Sbjct  390273  AAGAAG-CGCTGGAAGTGGATTGGGCATACCTTGAGGAAATCACCCAATTG--TGTCACA  390329

Query  112     AAAACAGCACTCCACTGGACCCCAGAAGGTAAAAGAAAGAGAGGAAGAC  160
               | |  ||| |||   ||||  || |||||| |||| |  ||||||||||
Sbjct  390330  AGACAAGCCCTCACATGGAATCCTGAAGGTCAAAGGAGAAGAGGAAGAC  390378
```

Features flanking this part of subject sequence:
5619 bp at 5' side: Adenine phosphoribosyltransferase, putative
50424 bp at 3' side: zinc finger protein, putative

```
 Score = 39.2 bits (42),  Expect = 7.9
 Identities = 48/66 (72%), Gaps = 0/66 (0%)
 Strand=Plus/Minus

Query  107     TCACAAAAACAGCACTCCACTGGACCCCAGAAGGTAAAAGAAAGAGAGGAAGACCTAAAA  166
               |||||| |  ||| |||   ||||  || |||||| |||| |  ||||||||||| || |
Sbjct  420831  TCACAAGACAAGCCCTCACATGGAATCCTGAAGGTCAAAGGAGAAGAGGAAGACCAAAGA  420772

Query  167     TGACAT  172
                 ||||
Sbjct  420771  ATACAT  420766
```

Features flanking this part of subject sequence:
17062 bp at 5' side: Adenine phosphoribosyltransferase, putative
38981 bp at 3' side: zinc finger protein, putative

```
 Score = 39.2 bits (42),  Expect = 7.9
 Identities = 48/66 (72%), Gaps = 0/66 (0%)
 Strand=Plus/Minus

Query  107     TCACAAAAACAGCACTCCACTGGACCCCAGAAGGTAAAAGAAAGAGAGGAAGACCTAAAA  166
               |||||| |  ||| |||   ||||  || |||||| |||| |  ||||||||||| || |
Sbjct  432274  TCACAAGACAAGCCCTCACATGGAATCCTGAAGGTCAAAGGAGAAGAGGAAGACCAAAGA  432215

Query  167     TGACAT  172
                 ||||
Sbjct  432214  ACACAT  432209
```

Features flanking this part of subject sequence:
31367 bp at 5' side: expressed protein
32350 bp at 3' side: camp-response element binding protein-related

```
 Score = 39.2 bits (42),  Expect = 7.9
 Identities = 48/66 (72%), Gaps = 0/66 (0%)
 Strand=Plus/Minus

Query  107     TCACAAAAACAGCACTCCACTGGACCCCAGAAGGTAAAAGAAAGAGAGGAAGACCTAAAA  166
               |||||| |  ||| |||   ||||  || |||||| |||| |  ||||||||||| || |
Sbjct  533170  TCACAAGACAAGCCCTCACATGGAATCCTGAAGGTCAAAGGAGAAGAGGAAGACCAAAGA  533111

Query  167     TGACAT  172
                 ||||
Sbjct  533110  ATACAT  533105
```

Features flanking this part of subject sequence:
61149 bp at 5' side: expressed protein
2568 bp at 3' side: camp-response element binding protein-related

```
 Score = 39.2 bits (42),  Expect = 7.9
 Identities = 48/66 (72%), Gaps = 0/66 (0%)
 Strand=Plus/Minus

Query  107     TCACAAAAACAGCACTCCACTGGACCCCAGAAGGTAAAAGAAAGAGAGGAAGACCTAAAA  166
               |||||| |  ||| |||   ||||  || |||||| |||| |  ||||||||||| || |
Sbjct  562952  TCACAAGACAAGCCCTCACATGGAATCCTGAAGGTCAAAGGAGAAGAGGAAGACCAAAGA  562893

Query  167     TGACAT  172
                 ||||
Sbjct  562892  ACACAT  562887
```

Features flanking this part of subject sequence:
8049 bp at 5' side: tyrosine kinase
31569 bp at 3' side: proto-oncogene tyrosine-protein kinase src, putative

```
 Score = 39.2 bits (42),  Expect = 7.9
 Identities = 48/66 (72%), Gaps = 0/66 (0%)
 Strand=Plus/Plus

Query  107     TCACAAAAACAGCACTCCACTGGACCCCAGAAGGTAAAAGAAAGAGAGGAAGACCTAAAA  166
               |||||| |  ||| |||   ||||  || |||||| |||| |  ||||||||||| || |
Sbjct  707155  TCACAAGACAAGCCCTCACATGGAATCCTGAAGGTCAAAGGAGAAGAGGAAGACCAAAGA  707214

Query  167     TGACAT  172
```

```
                   ||||
Sbjct  707215  ACACAT  707220
```

>**emb|FN357421.1|** D Schistosoma mansoni genome sequence supercontig Smp_scaff000130
Length=1530086

Sort alignments for this subject sequence by:
E value **Score** **Percent identity**
Query start position **Subject start position**

Features flanking this part of subject sequence:
17052 bp at 5' side: histone H4, putative
18085 bp at 3' side: UDP-N-acetylglucosamine--dolichyl-phosphate N-acetylgluco...

```
 Score = 44.6 bits (48),  Expect = 0.18
 Identities = 50/67 (74%), Gaps = 0/67 (0%)
 Strand=Plus/Minus

Query  106     ATCACAAAAACAGCACTCCACTGGACCCCAGAAGGTAAAAGAAAGAGAGGAAGACCTAAA  165
               |||||||||  ||| |||   ||||  || |||||| |||| |  ||||||||||| || 
Sbjct  574201  ATCACAAAACAAGCTCTCACATGGAATCCTGAAGGTCAAAGGAGAAGAGGAAGACCAAAG  574142

Query  166     ATGACAT  172
               |  ||||
Sbjct  574141  AACACAT  574135
```

Features flanking this part of subject sequence:
31675 bp at 5' side: hypothetical protein
1622 bp at 3' side: hypothetical protein

```
 Score = 42.8 bits (46),  Expect = 0.65
 Identities = 49/66 (74%), Gaps = 0/66 (0%)
 Strand=Plus/Minus

Query  107     TCACAAAAACAGCACTCCACTGGACCCCAGAAGGTAAAAGAAAGAGAGGAAGACCTAAAA  166
               |||||| |  |||||||   ||||  || |||||| |||| |  ||||||||||| || |
Sbjct  259367  TCACAAGACAAGCACTCACATGGAATCCTGAAGGTCAAAGGAGAAGAGGAAGACCAAAGA  259308

Query  167     TGACAT  172
                 ||||
Sbjct  259307  ACACAT  259302
```

Features in this part of subject sequence:
hypothetical protein

```
 Score = 42.8 bits (46),  Expect = 0.65
 Identities = 49/66 (74%), Gaps = 0/66 (0%)
 Strand=Plus/Minus

Query  107     TCACAAAAACAGCACTCCACTGGACCCCAGAAGGTAAAAGAAAGAGAGGAAGACCTAAAA  166
               |||||| |  |||||||   ||||  || |||||| |||| |  ||||||||||| || |
Sbjct  266672  TCACAAGACAAGCACTCACATGGAATCCTGAAGGTCAAAGGAGAAGAGGAAGACCAAAGA  266613

Query  167     TGACAT  172
                 ||||
Sbjct  266612  ACACAT  266607
```

Features flanking this part of subject sequence:
25624 bp at 5' side: hypothetical protein
7673 bp at 3' side: hypothetical protein

```
 Score = 39.2 bits (42),  Expect = 7.9
 Identities = 48/66 (72%), Gaps = 0/66 (0%)
 Strand=Plus/Plus

Query  107     TCACAAAAACAGCACTCCACTGGACCCCAGAAGGTAAAAGAAAGAGAGGAAGACCTAAAA  166
               |||||| |  |||||||   ||||  || |||||  |||| |  ||||||||||| || |
Sbjct  253251  TCACAAGACAAGCACTCGCATGGAATCCTGAAGGCCAAAGGAGAAGAGGAAGACCAAAGA  253310

Query  167     TGACAT  172
                 ||||
Sbjct  253311  ACACAT  253316
```

Features flanking this part of subject sequence:
32467 bp at 5' side: tar DNA-binding protein, putative
58182 bp at 3' side: pou3/brn-1, putative

```
 Score = 39.2 bits (42),  Expect = 7.9
 Identities = 48/66 (72%), Gaps = 0/66 (0%)
 Strand=Plus/Minus

Query  107     TCACAAAAACAGCACTCCACTGGACCCCAGAAGGTAAAAGAAAGAGAGGAAGACCTAAAA  166
               |||||| |  ||| |||   ||||  || |||||| |||| |  ||||||||||| || |
Sbjct  321173  TCACAAGACAAGCCCTCACATGGAATCCTGAAGGTCAAAGGAGAAGAGGAAGACCAAAGA  321114

Query  167     TGACAT  172
                 ||||
Sbjct  321113  ACACAT  321108
```

Features in this part of subject sequence:
hypothetical protein

```
 Score = 39.2 bits (42),  Expect = 7.9
```

Identities = 48/66 (72%), Gaps = 0/66 (0%)
Strand=Plus/Minus

```
Query  107     TCACAAAAACAGCACTCCACTGGACCCCAGAAGGTAAAAGAAAGAGAGGAAGACCTAAAA  166
               |||||| |  ||| |||   |||| ||| |||||  |||| |  ||||||||||| || |
Sbjct  624283  TCACAAGACAAGCCCTCACATGGAACCCTGAAGGCCAAAGGAGAAGAGGAAGACCAAAGA  624224

Query  167     TGACAT  172
                 ||||
Sbjct  624223  ACACAT  624218
```

>**emb|FN357350.1|** D Schistosoma mansoni genome sequence supercontig Smp_scaff000059
Length=1417180

Sort alignments for this subject sequence by:
E value **Score** **Percent identity**
Query start position **Subject start position**

Features in this part of subject sequence:
1-acyl-sn-glycerol-3-phosphate acyltransferase epsilon, f...

Score = 44.6 bits (48), Expect = 0.18
Identities = 81/117 (69%), Gaps = 4/117 (3%)
Strand=Plus/Minus

```
Query  58      AGACGCTGGAGGTGGATTCGGCATGTCCTACGGAAAGG--CCAGGGGGATATCACAAAAA  115
               || ||||||| ||||||| ||||  || |  |||||    |||   |  | |||||| |
Sbjct  861403  AGGCGCTGGAAGTGGATTGGGCACATCTTGAGGAAATCACCCAATTG--TGTCACAAGAC  861346

Query  116     CAGCACTCCACTGGACCCCAGAAGGTAAAAGAAAGAGAGGAAGACCTAAAATGACAT  172
                ||| |||   ||||  || |||||| |||| |  ||||||||||| || |  ||||
Sbjct  861345  AAGCCCTCACATGGAGTCCTGAAGGTCAAAGGAGAAGAGGAAGACCAAAGAACACAT  861289
```

Features flanking this part of subject sequence:
6826 bp at 5' side: conserved hypothetical protein
7656 bp at 3' side: stomatin-related

Score = 39.2 bits (42), Expect = 7.9
Identities = 48/66 (72%), Gaps = 0/66 (0%)
Strand=Plus/Minus

```
Query  107     TCACAAAAACAGCACTCCACTGGACCCCAGAAGGTAAAAGAAAGAGAGGAAGACCTAAAA  166
               |||||| |  ||| |||   ||||  || |||||| |||| |  ||||||||||| || |
Sbjct  630695  TCACAAGACAAGCCCTCACATGGAATCCTGAAGGTCAAAGGAGAAGAGGAAGACCAAAGA  630636

Query  167     TGACAT  172
                 ||||
Sbjct  630635  ACACAT  630630
```

>**emb|FN357331.1|** D Schistosoma mansoni genome sequence supercontig Smp_scaff000040
Length=1760029

Sort alignments for this subject sequence by:
E value **Score** **Percent identity**
Query start position **Subject start position**

Features in this part of subject sequence:
expressed protein

Score = 44.6 bits (48), Expect = 0.18
Identities = 56/76 (73%), Gaps = 1/76 (1%)
Strand=Plus/Plus

```
Query  97       CAGGGGGATATCACAAAAACAGCACTCCACTGGACCCCAGAAGGTAAAAGAAAGAGAGGA  156
                |||| ||| ||||||| || ||| ||    ||||  || |||||  |||| |  ||||||
Sbjct  1071957  CAGGCGGA-ATCACAAGAAAAGCCCTAACATGGAATCCTGAAGGCCAAAGGAGAAGAGGA  1072015

Query  157      AGACCTAAAATGACAT  172
                ||||| ||||  ||||
Sbjct  1072016  AGACCAAAAAACACAT  1072031
```

Features flanking this part of subject sequence:
11327 bp at 5' side: hypothetical protein
44045 bp at 3' side: hypothetical protein

Score = 39.2 bits (42), Expect = 7.9
Identities = 48/66 (72%), Gaps = 0/66 (0%)
Strand=Plus/Minus

```
Query  107     TCACAAAAACAGCACTCCACTGGACCCCAGAAGGTAAAAGAAAGAGAGGAAGACCTAAAA  166
               |||||| |  |||||||   ||||  || || ||| |||| |  ||||||||||| || |
Sbjct  412513  TCACAAGACAAGCACTCACATGGAATCCTGAGGGTCAAAGGAGAAGAGGAAGACCGAAGA  412454

Query  167     TGACAT  172
                 ||||
Sbjct  412453  ACACAT  412448
```

Features flanking this part of subject sequence:
59462 bp at 5' side: conserved hypothetical protein
18844 bp at 3' side: conserved hypothetical protein

Score = 39.2 bits (42), Expect = 7.9
Identities = 36/46 (78%), Gaps = 0/46 (0%)

```
 Strand=Plus/Minus

Query  127      TGGACCCCAGAAGGTAAAAGAAAGAGAGGAAGACCTAAAATGACAT  172
                ||||  || |||||| ||||||  ||||||||||| || |  ||||
Sbjct  1001426  TGGAATCCTGAAGGTCAAAGAAGAAGAGGAAGACCAAAGAACACAT  1001381
```

>**emb|FN357326.1|** Schistosoma mansoni genome sequence supercontig Smp_scaff000035
Length=1622525

Sort alignments for this subject sequence by:
E value **Score** **Percent identity**
Query start position **Subject start position**

Features flanking this part of subject sequence:
1958 bp at 5' side: hypothetical protein
12166 bp at 3' side: expressed protein

```
 Score = 44.6 bits (48),  Expect = 0.18
 Identities = 82/119 (68%), Gaps = 1/119 (0%)
 Strand=Plus/Plus

Query  54      AAGAAGACGCTGGAGGTGGATTCGGCATGTCCTACGGAAAGGCCAGGGGGATATCACAAA  113
               |||||| ||||||| ||||||| ||||   | |  |||||   |  |      ||||||
Sbjct  221252  AAGAAG-CGCTGGAAGTGGATTGGGCACACCTTGAGGAAATCACCTGATTGCGTCACAAG  221310

Query  114     AACAGCACTCCACTGGACCCCAGAAGGTAAAAGAAAGAGAGGAAGACCTAAAATGACAT  172
               |  ||| |||   ||||  || |||||| |||| || ||||||||||| || |  ||||
Sbjct  221311  ACAAGCCCTCACATGGAATCCTGAAGGTCAAAGGAAAAGAGGAAGACCAAAGAACACAT  221369
```

Features flanking this part of subject sequence:
85184 bp at 5' side: nascent polypeptide associated complex alpha subunit (nac...
10339 bp at 3' side: cadherin, putative

```
 Score = 39.2 bits (42),  Expect = 7.9
 Identities = 48/66 (72%), Gaps = 0/66 (0%)
 Strand=Plus/Minus

Query  107     TCACAAAAACAGCACTCCACTGGACCCCAGAAGGTAAAAGAAAGAGAGGAAGACCTAAAA  166
               |||||| |  ||| |||   ||||  || |||||| |||| |  ||||||||||| || |
Sbjct  875791  TCACAAGACAAGCCCTCACATGGAATCCTGAAGGTCAAAGGAGAAGAGGAAGACCAAAGA  875732

Query  167     TGACAT  172
                 ||||
Sbjct  875731  ACACAT  875726
```

>**emb|FN357322.1|** Schistosoma mansoni genome sequence supercontig Smp_scaff000031
Length=1014221

Sort alignments for this subject sequence by:
E value **Score** **Percent identity**
Query start position **Subject start position**

Features flanking this part of subject sequence:
12878 bp at 5' side: homeobox protein vsx-1-related
115128 bp at 3' side: hypothetical protein

```
 Score = 44.6 bits (48),  Expect = 0.18
 Identities = 55/72 (76%), Gaps = 4/72 (5%)
 Strand=Plus/Minus

Query  107     TCACAAAAACAGCACTCCAC-TGGACCCCAGAAGGTAAAAGAAAGAGAGGAAGACCTAAA  165
               |||||| |  ||| || ||| ||||  ||||||||  |||| || |||||||||||  ||
Sbjct  424414  TCACAAGACAAGC-CTTCACATGGAATCCAGAAGGCCAAAGGAAAAGAGGAAGACC--AA  424358

Query  166     ATGACATGGAGA  177
               |  | |||||||
Sbjct  424357  AGAAAATGGAGA  424346
```

Features flanking this part of subject sequence:
17809 bp at 5' side: hypothetical protein
142294 bp at 3' side: 40S ribosomal protein S3, putative

```
 Score = 42.8 bits (46),  Expect = 0.65
 Identities = 44/58 (75%), Gaps = 0/58 (0%)
 Strand=Plus/Plus

Query  107    TCACAAAAACAGCACTCCACTGGACCCCAGAAGGTAAAAGAAAGAGAGGAAGACCTAA  164
              |||||| |  ||| |||   ||||  || |||||  |||| |||||||||||||| ||
Sbjct  44480  TCACAAGATAAGCCCTCACATGGAGTCCTGAAGGCCAAAGGAAGAGAGGAAGACCAAA  44537
```

Features in this part of subject sequence:
ubiquitin-specific peptidase 9X (C19 family)

```
 Score = 39.2 bits (42),  Expect = 7.9
 Identities = 48/66 (72%), Gaps = 0/66 (0%)
 Strand=Plus/Plus

Query  107     TCACAAAAACAGCACTCCACTGGACCCCAGAAGGTAAAAGAAAGAGAGGAAGACCTAAAA  166
               |||||| |  ||| |||   ||||  || |||||| ||||    ||||||||||| ||||
Sbjct  941523  TCACAAGACAAGCTCTCACATGGAAACCTGAAGGTCAAAGGCGAAGAGGAAGACCAAAAA  941582

Query  167     TGACAT  172
                 ||||
```

```
Sbjct  941583  ACACAT  941588
```

>**emb|FN357316.1|** D Schistosoma mansoni genome sequence supercontig Smp_scaff000025
Length=1332955

Sort alignments for this subject sequence by:
E value **Score** **Percent identity**
Query start position **Subject start position**

Features flanking this part of subject sequence:
10308 bp at 5' side: hypothetical protein
4521 bp at 3' side: expressed protein

Score = 44.6 bits (48), Expect = 0.18
Identities = 50/67 (74%), Gaps = 0/67 (0%)
Strand=Plus/Plus

```
Query  106      ATCACAAAAACAGCACTCCACTGGACCCCAGAAGGTAAAAGAAAGAGAGGAAGACCTAAA  165
                ||||||| |  ||| |||    ||||  || |||||  |||| || |||||||||| |||
Sbjct  1234502  ATCACAAGACAAGCCCTCACATGGAATCCTGAAGGCCAAAGGAAAAGAGGAAGACCAAAA  1234561

Query  166      ATGACAT  172
                |  ||||
Sbjct  1234562  AACACAT  1234568
```

Features flanking this part of subject sequence:
31736 bp at 5' side: enhancer of polycomb, putative
12821 bp at 3' side: expressed protein

Score = 39.2 bits (42), Expect = 7.9
Identities = 48/66 (72%), Gaps = 0/66 (0%)
Strand=Plus/Plus

```
Query  107    TCACAAAAACAGCACTCCACTGGACCCCAGAAGGTAAAAGAAAGAGAGGAAGACCTAAAA  166
              |||||| |  ||| |||    ||||  || |||||| |||| |  ||||||||||| || |
Sbjct  37242  TCACAAGACAAGCCCTCACATGGAATCCTGAAGGTCAAAGGAGAAGAGGAAGACCAAAGA  37301

Query  167    TGACAT  172
                ||||
Sbjct  37302  ACACAT  37307
```

Features flanking this part of subject sequence:
21134 bp at 5' side: nck2/grb4, putative
17856 bp at 3' side: expressed protein

Score = 39.2 bits (42), Expect = 7.9
Identities = 48/66 (72%), Gaps = 0/66 (0%)
Strand=Plus/Minus

```
Query  107     TCACAAAAACAGCACTCCACTGGACCCCAGAAGGTAAAAGAAAGAGAGGAAGACCTAAAA  166
               |||||| |  ||| |||    ||||  || |||||| |||| |  ||||||||||| || |
Sbjct  768317  TCACAAGACAAGCCCTCACATGGAATCCTGAAGGTCAAAGGAGAAGAGGAAGACCAAAGA  768258

Query  167     TGACAT  172
                 ||||
Sbjct  768257  ACACAT  768252
```

>**emb|FN368005.1|** Schistosoma mansoni genome sequence supercontig Smp_scaff010714
Length=3224

Score = 44.6 bits (48), Expect = 0.18
Identities = 86/124 (69%), Gaps = 5/124 (4%)
Strand=Plus/Minus

```
Query  51    AATAAGAAGACGCTGGAGGTGGATTCGGCATGTCCTACGGAAA--GGCCAGGGGGATATC  108
             |||| ||| |||||||| ||||||| ||||    | |  |||||    || |    |    ||
Sbjct  2786  AATACGAA-ACGCTGGAAGTGGATTGGGCACACCTTGAGGAAATCAGCTAATTG--CGTC  2730

Query  109   ACAAAAACAGCACTCCACTGGACCCCAGAAGGTAAAAGAAAGAGAGGAAGACCTAAAATG  168
             |||| |  ||| |||    ||||   || || ||| ||||  ||||||||||||| || |
Sbjct  2729  ACAAGACAAGCCCTCACATGGAATCCTGATGGTCAAAGGAAGAGAGGAAGACCAAAGAAC  2670

Query  169   ACAT  172
             ||||
Sbjct  2669  ACAT  2666
```

>**emb|FN360200.1|** Schistosoma mansoni genome sequence supercontig Smp_scaff002909
Length=2872

Score = 44.6 bits (48), Expect = 0.18
Identities = 82/119 (68%), Gaps = 1/119 (0%)
Strand=Plus/Minus

```
Query  54   AAGAAGACGCTGGAGGTGGATTCGGCATGTCCTACGGAAAGGCCAGGGGGATATCACAAA  113
            |||||| ||||||| ||||||| ||||    | |  |||||    | |         ||||||
Sbjct  679  AAGAAG-CGCTGGAAGTGGATTGGGCACACCTTGAGGAAATCACCGAATTGCGTCACAAT  621

Query  114  AACAGCACTCCACTGGACCCCAGAAGGTAAAAGAAAGAGAGGAAGACCTAAAATGACAT  172
            |  ||| |||    ||||  ||||||||| |||| |  ||||||||||| || |  ||||
Sbjct  620  ACAAGCCCTCACATGGAATCCAGAAGGTCAAAGGAGAAGAGGAAGACCAAAGAACACAT  562
```

>**dbj|AP010341.1|** D Lotus japonicus genomic DNA, chromosome 3, clone: LjT15I02, TM0666,

complete sequence
Length=108521

Score = 44.6 bits (48), Expect = 0.18
Identities = 42/52 (80%), Gaps = 4/52 (7%)
Strand=Plus/Plus

```
Query  142    AAAAGAAAGAGA--GGAAGACCTAAAATGACATGGAGAAG--AACTGTAGAG  189
              ||||||| ||||  |||||||| | ||||| |||| ||||  || |||||||
Sbjct  35689  AAAAGAAGGAGATCGGAAGACCGAGAATGAGATGGGGAAGAAAAATGTAGAG  35740
```

>**gb|AF391293.1|** Branchiostoma floridae cosmid MPMGc117K0849, partial sequence
Length=36049

Score = 44.6 bits (48), Expect = 0.18
Identities = 49/65 (75%), Gaps = 3/65 (4%)
Strand=Plus/Plus

```
Query  112    AAAACAGCACTCCACTGGACCCCAG---AAGGTAAAAGAAAGAGAGGAAGACCTAAAATG  168
              ||||||||||| |||||||  ||||   | || ||||||||    |||||||| ||| | 
Sbjct  17052  AAAACAGCACTGCACTGGAAACCAGGCAATGGAAAAAGAAAACAGGGAAGACCAAAATTA  17111

Query  169    ACATG  173
              || ||
Sbjct  17112  ACTTG  17116
```

>**gb|AC150407.2|** Branchiostoma floridae clone CH302-54J24, complete sequence
Length=175896

Sort alignments for this subject sequence by:
E value **Score** **Percent identity**
Query start position **Subject start position**

Score = 44.6 bits (48), Expect = 0.18
Identities = 36/44 (81%), Gaps = 0/44 (0%)
Strand=Plus/Minus

```
Query  145     AGAAAGAGAGGAAGACCTAAAATGACATGGAGAAGAACTGTAGA  188
               |||||||| |||||||     ||||| ||||||||||| |||||
Sbjct  169086  AGAAAGAGGGGAAGACAAGCCATGACGTGGAGAAGAACAGTAGA  169043
```

Score = 39.2 bits (42), Expect = 7.9
Identities = 35/44 (79%), Gaps = 0/44 (0%)
Strand=Plus/Minus

```
Query  145     AGAAAGAGAGGAAGACCTAAAATGACATGGAGAAGAACTGTAGA  188
               |||||||| ||| |||     ||||| ||||||||||| |||||
Sbjct  168842  AGAAAGAGGGGAGGACAAGCCATGACGTGGAGAAGAACAGTAGA  168799
```

Score = 39.2 bits (42), Expect = 7.9
Identities = 35/44 (79%), Gaps = 0/44 (0%)
Strand=Plus/Minus

```
Query  145     AGAAAGAGAGGAAGACCTAAAATGACATGGAGAAGAACTGTAGA  188
               |||||||| ||| |||     ||||| ||||||||||| |||||
Sbjct  168964  AGAAAGAGGGGAGGACAAGCCATGACGTGGAGAAGAACAGTAGA  168921
```

>**ref|NW_003039129.1|** Schistosoma mansoni genome sequence supercontig Smp_scaff000238
Length=434718

Sort alignments for this subject sequence by:
E value **Score** **Percent identity**
Query start position **Subject start position**

Features flanking this part of subject sequence:
915 bp at 5' side: protein arginine n-methyltransferase
1387 bp at 3' side: dynein light chain

Score = 42.8 bits (46), Expect = 0.65
Identities = 49/66 (74%), Gaps = 0/66 (0%)
Strand=Plus/Minus

```
Query  107     TCACAAAAACAGCACTCCACTGGACCCCAGAAGGTAAAAGAAAGAGAGGAAGACCTAAAA  166
               |||||| |  ||| |||   |||| ||| |||||| |||| |  ||||||||||| || |
Sbjct  313371  TCACAAGACAAGCCCTCACATGGAACCCTGAAGGTCAAAGGAGAAGAGGAAGACCAAAGA  313312

Query  167     TGACAT  172
                 ||||
Sbjct  313311  ACACAT  313306
```

Features flanking this part of subject sequence:
21084 bp at 5' side: hypothetical protein

Score = 39.2 bits (42), Expect = 7.9
Identities = 48/66 (72%), Gaps = 0/66 (0%)
Strand=Plus/Plus

```
Query  107     TCACAAAAACAGCACTCCACTGGACCCCAGAAGGTAAAAGAAAGAGAGGAAGACCTAAAA  166
               |||||| |  ||| |||   ||||  || |||||| |||| |  ||||||||||| || |
Sbjct  426498  TCACAAGACAAGCCCTCACATGGAATCCTGAAGGTCAAAGGAGAAGAGGAAGACCAAAGA  426557

Query  167     TGACAT  172
```

```
                  ||||
Sbjct  426558  ACACAT  426563
```

>**ref|NW_003038510.1|** Schistosoma mansoni genome sequence supercontig Smp_scaff001998
Length=63486

```
 Score = 42.8 bits (46),  Expect = 0.65
 Identities = 49/66 (74%), Gaps = 0/66 (0%)
 Strand=Plus/Minus

Query  107    TCACAAAAACAGCACTCCACTGGACCCCAGAAGGTAAAAGAAAGAGAGGAAGACCTAAAA  166
              |||||| |  ||| |||   || |  || ||| || ||||||||||||||||||| || |
Sbjct  29987  TCACAAGACAAGCCCTCACATGAAATCCTGAAAGTGAAAGAAAGAGAGGAAGACCAAAGA  29928

Query  167    TGACAT  172
                ||||
Sbjct  29927  ACACAT  29922
```

>**ref|NW_003038509.1|** Schistosoma mansoni genome sequence supercontig Smp_scaff001996
Length=334775

Features flanking this part of subject sequence:
56869 bp at 5' side: hypothetical protein
9200 bp at 3' side: hypothetical protein

```
 Score = 42.8 bits (46),  Expect = 0.65
 Identities = 49/66 (74%), Gaps = 0/66 (0%)
 Strand=Plus/Plus

Query  107     TCACAAAAACAGCACTCCACTGGACCCCAGAAGGTAAAAGAAAGAGAGGAAGACCTAAAA  166
               |||||| |  ||| |||   ||||  || |||||| |||| || ||||||||| | ||||
Sbjct  274534  TCACAACACAAGCCCTCACATGGAATCCTGAAGGTCAAAGGAAAAGAGGAAGATCAAAAA  274593

Query  167     TGACAT  172
                 ||||
Sbjct  274594  ACACAT  274599
```

>**ref|NW_003038070.1|** Schistosoma mansoni genome sequence supercontig Smp_scaff000314
Length=465702

Sort alignments for this subject sequence by:
E value **Score** **Percent identity**
Query start position **Subject start position**

Features in this part of subject sequence:
nucleolysin tia-1

```
 Score = 42.8 bits (46),  Expect = 0.65
 Identities = 61/85 (71%), Gaps = 2/85 (2%)
 Strand=Plus/Minus

Query  106     ATCACAAAAACAGCACTCCACTGGACCCCAGAAGGTAAAAGAAAGAGAGGAAGACCTAAA  165
               ||||||| |  |||||||   ||||  || | |||  |||| |  ||||||||||| ||
Sbjct  397270  ATCACAAGACAAGCACTCATATGGAATCCTGGAGGCCAAAGGAGAAGAGGAAGACCAAAG  397211

Query  166     ATGACATGGAG--AAGAACTGTAGA  188
               |  |||| | |  ||||| ||||||
Sbjct  397210  AACACATTGTGCCAAGAAATGTAGA  397186
```

Features flanking this part of subject sequence:
6517 bp at 5' side: nucleolysin tia-1

```
 Score = 41.0 bits (44),  Expect = 2.3
 Identities = 49/67 (73%), Gaps = 0/67 (0%)
 Strand=Plus/Plus

Query  106     ATCACAAAAACAGCACTCCACTGGACCCCAGAAGGTAAAAGAAAGAGAGGAAGACCTAAA  165
               |||||||    |||||||   ||||  || |||||| | || |  ||||||||||||||
Sbjct  454583  ATCACAAGGCAAGCACTCACATGGAATCCTGAAGGTCAGAGGAGAAGAGGAAGACCTAAG  454642

Query  166     ATGACAT  172
               |  ||||
Sbjct  454643  AACACAT  454649
```

Features flanking this part of subject sequence:
1779 bp at 5' side: hypothetical protein
5898 bp at 3' side: hypothetical protein

```
 Score = 39.2 bits (42),  Expect = 7.9
 Identities = 48/66 (72%), Gaps = 0/66 (0%)
 Strand=Plus/Minus

Query  107     TCACAAAAACAGCACTCCACTGGACCCCAGAAGGTAAAAGAAAGAGAGGAAGACCTAAAA  166
               |||||| |  ||| |||   ||||  || |||||| |||| |  ||||||||||| || |
Sbjct  239746  TCACAAGACAAGCCCTCACATGGAATCCTGAAGGTCAAAGGAGAAGAGGAAGACCAAAGA  239687

Query  167     TGACAT  172
                 ||||
Sbjct  239686  ACACAT  239681
```

Features flanking this part of subject sequence:
41224 bp at 5' side: hypothetical protein

8615 bp at 3' side: hypothetical protein

```
 Score = 39.2 bits (42),  Expect = 7.9
 Identities = 45/61 (73%), Gaps = 0/61 (0%)
 Strand=Plus/Minus

Query  106     ATCACAAAAACAGCACTCCACTGGACCCCAGAAGGTAAAAGAAAGAGAGGAAGACCTAAA  165
               |||| || |  ||| |||   ||||  || |||||| |||| |  ||||||||||| |||
Sbjct  363849  ATCATAAGACAAGCTCTCACATGGAATCCTGAAGGTCAAAGGAGAAGAGGAAGACCAAAA  363790

Query  166     A  166
               |
Sbjct  363789  A  363789
```

>**ref|NW_003038013.1|** D Schistosoma mansoni genome sequence supercontig Smp_scaff000239
Length=350963

Sort alignments for this subject sequence by:
E value **Score** **Percent identity**
Query start position **Subject start position**

Features flanking this part of subject sequence:
8050 bp at 5' side: hypothetical protein
3581 bp at 3' side: hypothetical protein

```
 Score = 42.8 bits (46),  Expect = 0.65
 Identities = 49/66 (74%), Gaps = 0/66 (0%)
 Strand=Plus/Plus

Query  107     TCACAAAAACAGCACTCCACTGGACCCCAGAAGGTAAAAGAAAGAGAGGAAGACCTAAAA  166
               |||||| |  ||| |||   ||||  || |||||| |||| |  ||||||||||| ||||
Sbjct  332302  TCACAAGACAAGCCCTCACATGGAATCCTGAAGGTCAAAGGAGAAGAGGAAGACCAAAAA  332361

Query  167     TGACAT  172
                 ||||
Sbjct  332362  ACACAT  332367
```

Features flanking this part of subject sequence:
14042 bp at 5' side: sodium/chloride dependent neurotransmitter transporter
14378 bp at 3' side: hypothetical protein

```
 Score = 39.2 bits (42),  Expect = 7.9
 Identities = 48/66 (72%), Gaps = 0/66 (0%)
 Strand=Plus/Minus

Query  107     TCACAAAAACAGCACTCCACTGGACCCCAGAAGGTAAAAGAAAGAGAGGAAGACCTAAAA  166
               |||||| |  ||| |||   || |  || |||||| |||| || ||||||||||| || |
Sbjct  213773  TCACAAGACAAGCTCTCACATGAAATCCTGAAGGTCAAAGGAAAAGAGGAAGACCAAAGA  213714

Query  167     TGACAT  172
                 ||||
Sbjct  213713  ACACAT  213708
```

>**ref|NW_003038004.1|** D Schistosoma mansoni genome sequence supercontig Smp_scaff000229
Length=237986

Sort alignments for this subject sequence by:
E value **Score** **Percent identity**
Query start position **Subject start position**

Features flanking this part of subject sequence:
22107 bp at 5' side: hypothetical protein

```
 Score = 42.8 bits (46),  Expect = 0.65
 Identities = 78/113 (69%), Gaps = 1/113 (0%)
 Strand=Plus/Plus

Query  54      AAGAAGACGCTGGAGGTGGATTCGGCATGTCCTACGGAAAGGCCAGGGGGATATCACAAA  113
               |||||| ||||||| ||||||| ||||   | |  |||||   |  ||     ||||||
Sbjct  120351  AAGAAG-CGCTGGAAGTGGATTGGGCACACCTTGAGGAAATCACCTGGTAGCGTCACAAT  120409

Query  114     AACAGCACTCCACTGGACCCCAGAAGGTAAAAGAAAGAGAGGAAGACCTAAAA  166
               |  ||| |||   ||||  ||||||||| |||| |  | | ||||||| ||||
Sbjct  120410  ACAAGCCCTCACATGGAATCCAGAAGGTCAAAGGAGAAAATGAAGACCAAAAA  120462
```

Features flanking this part of subject sequence:
13855 bp at 5' side: hypothetical protein

```
 Score = 39.2 bits (42),  Expect = 7.9
 Identities = 83/120 (69%), Gaps = 3/120 (2%)
 Strand=Plus/Minus

Query  54      AAGAAGACGCTGGAGGTGGATTCGGCATGTCCTACGGAAAGGCCAGGGGGATATCACAAA  113
               |||||| ||||||| ||||||| ||||   | |  |||||   |  ||     ||||||
Sbjct  112216  AAGAAG-CGCTGGAAGTGGATTGGGCACACCTTGAGGAAATCACCTGGTTGCGTCACAAT  112158

Query  114     AACAGCACTCCACTGGACCCCAGAAGGTAAAAGAAAGAGAGG-AAGACCTAAAATGACAT  172
               |  ||| |||   ||||  ||||||||| ||||  |||| || |||||| || |  ||||
Sbjct  112157  ACAAGCCCTCACATGGAATCCAGAAGGTCAAAG-GAGAGTGGAAAGACCAAAGAACACAT  112099
```

>**ref|NW_003038002.1|** D Schistosoma mansoni genome sequence supercontig Smp_scaff000227
Length=378973

Sort alignments for this subject sequence by:
E value **Score** **Percent identity**
Query start position **Subject start position**

Features in this part of subject sequence:
hypothetical protein

```
 Score = 42.8 bits (46),  Expect = 0.65
 Identities = 49/66 (74%), Gaps = 0/66 (0%)
 Strand=Plus/Minus

Query  107     TCACAAAAACAGCACTCCACTGGACCCCAGAAGGTAAAAGAAAGAGAGGAAGACCTAAAA  166
               |||||| |  ||| |||   ||||  || |||||| ||||||  ||||||||||| || |
Sbjct  190635  TCACAAGACAAGCCCTCACATGGAATCCTGAAGGTCAAAGAAGAAGAGGAAGACCAAAGA  190576

Query  167     TGACAT  172
                 ||||
Sbjct  190575  ACACAT  190570
```

Features flanking this part of subject sequence:
13833 bp at 5' side: hypothetical protein
8724 bp at 3' side: FTZ-F1 nuclear receptor-like protein

```
 Score = 42.8 bits (46),  Expect = 0.65
 Identities = 44/58 (75%), Gaps = 0/58 (0%)
 Strand=Plus/Plus

Query  107     TCACAAAAACAGCACTCCACTGGACCCCAGAAGGTAAAAGAAAGAGAGGAAGACCTAA  164
               ||||||||  ||| |||   || |  || |||||| |||| || ||||||||||| ||
Sbjct  249950  TCACAAAACAAGCTCTCACATGAAATCCTGAAGGTTAAAGGAAAAGAGGAAGACCAAA  250007
```

>**ref|NW_003037995.1|** D Schistosoma mansoni genome sequence supercontig Smp_scaff000219
Length=555008

Sort alignments for this subject sequence by:
E value **Score** **Percent identity**
Query start position **Subject start position**

Features flanking this part of subject sequence:
41387 bp at 5' side: hypothetical protein

```
 Score = 42.8 bits (46),  Expect = 0.65
 Identities = 49/66 (74%), Gaps = 0/66 (0%)
 Strand=Plus/Minus

Query  107     TCACAAAAACAGCACTCCACTGGACCCCAGAAGGTAAAAGAAAGAGAGGAAGACCTAAAA  166
               |||||| |  |||||||   ||||  || | |||  |||| |||||||||||||| || |
Sbjct  545980  TCACAAGACAAGCACTCACATGGAATCCTGGAGGCCAAAGGAAGAGAGGAAGACCAAAGA  545921

Query  167     TGACAT  172
                 ||||
Sbjct  545920  ACACAT  545915
```

Features flanking this part of subject sequence:
14892 bp at 5' side: hypothetical protein
12727 bp at 3' side: cadherin

```
 Score = 39.2 bits (42),  Expect = 7.9
 Identities = 48/66 (72%), Gaps = 0/66 (0%)
 Strand=Plus/Plus

Query  107     TCACAAAAACAGCACTCCACTGGACCCCAGAAGGTAAAAGAAAGAGAGGAAGACCTAAAA  166
               |||||| |  ||| |||   ||||  || |||||| |||| |  ||||||||||| || |
Sbjct  212110  TCACAAGACAAGCCCTCACATGGAATCCCGAAGGTCAAAGGAGAAGAGGAAGACCAAAGA  212169

Query  167     TGACAT  172
                 ||||
Sbjct  212170  ACACAT  212175
```

>**ref|NW_003037937.1|** D Schistosoma mansoni genome sequence supercontig Smp_scaff000151
Length=837104

Sort alignments for this subject sequence by:
E value **Score** **Percent identity**
Query start position **Subject start position**

Features in this part of subject sequence:
hypothetical protein

```
 Score = 42.8 bits (46),  Expect = 0.65
 Identities = 50/68 (73%), Gaps = 0/68 (0%)
 Strand=Plus/Plus

Query  105     TATCACAAAAACAGCACTCCACTGGACCCCAGAAGGTAAAAGAAAGAGAGGAAGACCTAA  164
               |||||||| |  ||| |||   ||||  || |||||| |||| |  ||||||||||| ||
Sbjct  743382  TATCACAAGACAAGCCCTCACATGGAATCCTGAAGGTCAAAGGATAAGAGGAAGACCAAA  743441

Query  165     AATGACAT  172
                |  ||||
Sbjct  743442  GAACACAT  743449
```

Features flanking this part of subject sequence:
38039 bp at 5' side: hypothetical protein

22950 bp at 3' side: fad NAD binding oxidoreductases

```
 Score = 39.2 bits (42),  Expect = 7.9
 Identities = 48/66 (72%), Gaps = 0/66 (0%)
 Strand=Plus/Plus

Query  107     TCACAAAAACAGCACTCCACTGGACCCCAGAAGGTAAAAGAAAGAGAGGAAGACCTAAAA  166
               |||||| |  ||| |||   |||| ||| ||| || |||| |  ||||||||||| || |
Sbjct  141116  TCACAAGACAAGCCCTCACATGGAACCCTGAAAGTGAAAGGAGAAGAGGAAGACCAAAGA  141175

Query  167     TGACAT  172
                 ||||
Sbjct  141176  ACACAT  141181
```

>**ref|NW_003037936.1|** D Schistosoma mansoni genome sequence supercontig Smp_scaff000150
Length=760080

Sort alignments for this subject sequence by:
E value **Score** **Percent identity**
Query start position **Subject start position**

Features flanking this part of subject sequence:
92247 bp at 5' side: PI3kinase
31884 bp at 3' side: rhodopsin-like orphan GPCR

```
 Score = 42.8 bits (46),  Expect = 0.65
 Identities = 49/66 (74%), Gaps = 0/66 (0%)
 Strand=Plus/Minus

Query  107     TCACAAAAACAGCACTCCACTGGACCCCAGAAGGTAAAAGAAAGAGAGGAAGACCTAAAA  166
               |||||| |  ||| |||   ||||  || |||||| |||| |  |||||||||||||| |
Sbjct  587700  TCACAAGACAAGCCCTCACATGGAATCCTGAAGGTCAAAGGAGAAGAGGAAGACCTAAGA  587641

Query  167     TGACAT  172
                 ||||
Sbjct  587640  ACACAT  587635
```

Features in this part of subject sequence:
PI3kinase

```
 Score = 39.2 bits (42),  Expect = 7.9
 Identities = 48/66 (72%), Gaps = 0/66 (0%)
 Strand=Plus/Plus

Query  107     TCACAAAAACAGCACTCCACTGGACCCCAGAAGGTAAAAGAAAGAGAGGAAGACCTAAAA  166
               |||||| |  ||| |||   ||||  || |||||| |||| |  ||||||||||| || |
Sbjct  468667  TCACAAGACAAGCCCTCACATGGAATCCTGAAGGTCAAAGGAGAAGAGGAAGACCAAAGA  468726

Query  167     TGACAT  172
                 ||||
Sbjct  468727  ACACAT  468732
```

Features in this part of subject sequence:
PI3kinase

```
 Score = 39.2 bits (42),  Expect = 7.9
 Identities = 48/66 (72%), Gaps = 0/66 (0%)
 Strand=Plus/Plus

Query  107     TCACAAAAACAGCACTCCACTGGACCCCAGAAGGTAAAAGAAAGAGAGGAAGACCTAAAA  166
               |||||| |  ||| |||   |||| ||| |||||| |||| |  | ||||||||| || |
Sbjct  480519  TCACAAGACAAGCCCTCACATGGAACCCTGAAGGTCAAAGGAGAAAAGGAAGACCAAAGA  480578

Query  167     TGACAT  172
                 ||||
Sbjct  480579  ACACAT  480584
```

Features flanking this part of subject sequence:
19920 bp at 5' side: PI3kinase
104211 bp at 3' side: rhodopsin-like orphan GPCR

```
 Score = 39.2 bits (42),  Expect = 7.9
 Identities = 48/66 (72%), Gaps = 0/66 (0%)
 Strand=Plus/Plus

Query  107     TCACAAAAACAGCACTCCACTGGACCCCAGAAGGTAAAAGAAAGAGAGGAAGACCTAAAA  166
               |||||| |  ||| |||   ||||  || |||||| |||| |  ||||||||||| || |
Sbjct  515308  TCACAAGACAAGCCCTCACATGGAATCCTGAAGGTCAAAGGAGAAGAGGAAGACCAAAGA  515367

Query  167     TGACAT  172
                 ||||
Sbjct  515368  ACACAT  515373
```

>**ref|NW_003037929.1|** D Schistosoma mansoni genome sequence supercontig Smp_scaff000143
Length=617086

Sort alignments for this subject sequence by:
E value **Score** **Percent identity**
Query start position **Subject start position**

Features in this part of subject sequence:
hypothetical protein

```
 Score = 42.8 bits (46),  Expect = 0.65
 Identities = 49/66 (74%), Gaps = 0/66 (0%)
 Strand=Plus/Plus

Query  107     TCACAAAAACAGCACTCCACTGGACCCCAGAAGGTAAAAGAAAGAGAGGAAGACCTAAAA  166
               ||||||||  ||| |||   ||||  || |||||| |||| |  ||||||||||| || |
Sbjct  431554  TCACAAAACAAGCCCTCATATGGAATCCTGAAGGTCAAAGGAGAAGAGGAAGACCAAAGA  431613

Query  167     TGACAT  172
                 ||||
Sbjct  431614  ACACAT  431619


 Features in this part of subject sequence:
   twik family of potassium channels-related

 Score = 39.2 bits (42),  Expect = 7.9
 Identities = 48/66 (72%), Gaps = 0/66 (0%)
 Strand=Plus/Plus

Query  107     TCACAAAAACAGCACTCCACTGGACCCCAGAAGGTAAAAGAAAGAGAGGAAGACCTAAAA  166
               |||||| |  ||| |||   ||||  || |||||| |||| |  ||||||||||| || |
Sbjct  187688  TCACAAGACAAGCCCTCACATGGAATCCTGAAGGTCAAAGGAGAAGAGGAAGACCAAAGA  187747

Query  167     TGACAT  172
                 ||||
Sbjct  187748  ACACAT  187753


>ref|NW_003037772.1|  Schistosoma mansoni genome sequence supercontig Smp_scaff018982
Length=84897

 Score = 42.8 bits (46),  Expect = 0.65
 Identities = 49/66 (74%), Gaps = 0/66 (0%)
 Strand=Plus/Plus

Query  107    TCACAAAAACAGCACTCCACTGGACCCCAGAAGGTAAAAGAAAGAGAGGAAGACCTAAAA  166
              |||||| |  ||| |||   ||||  || ||||||||||| |  ||||||||||| || |
Sbjct  46074  TCACAAGACAAGCCCTCACATGGAATCCTGAAGGTAAAAGGAGAAGAGGAAGACCAAAGA  46133

Query  167    TGACAT  172
                ||||
Sbjct  46134  AAACAT  46139


>ref|NW_003037196.1|  Schistosoma mansoni genome sequence supercontig Smp_scaff012462
Length=4546

 Score = 42.8 bits (46),  Expect = 0.65
 Identities = 84/121 (69%), Gaps = 5/121 (4%)
 Strand=Plus/Minus

Query  54    AAGAAGACGCTGGAGGTGGATTCGGCATGTCCTACGGAAAGG--CCAGGGGGATATCACA  111
             |||||| ||||||| ||||||| ||||   | |  |||||    |||   |  | |||||
Sbjct  2692  AAGAAG-CGCTGGAAGTGGATTGGGCACACCTTGAGGAAATCACCCAATTG--TGTCACA  2636

Query  112   AAAACAGCACTCCACTGGACCCCAGAAGGTAAAAGAAAGAGAGGAAGACCTAAAATGACA  171
             | |  ||| |||   ||||  || |||||| |||| |  ||||||||||| || |  |||
Sbjct  2635  AGACAAGCCCTCGCATGGAATCCTGAAGGTCAAAGGAGAAGAGGAAGACCAAAGAACACA  2576

Query  172   T  172
             |
Sbjct  2575  T  2575


>ref|NW_003035875.1|  Schistosoma mansoni genome sequence supercontig Smp_scaff001988
Length=22923

 Score = 42.8 bits (46),  Expect = 0.65
 Identities = 49/66 (74%), Gaps = 0/66 (0%)
 Strand=Plus/Minus

Query  107    TCACAAAAACAGCACTCCACTGGACCCCAGAAGGTAAAAGAAAGAGAGGAAGACCTAAAA  166
              |||||| |  ||| |||   ||||  ||||||||| |||| |  ||||||||||| || |
Sbjct  13805  TCACAAGACAAGCCCTCACATGGAATCCAGAAGGTCAAAGGAGAAGAGGAAGACCAAAGA  13746

Query  167    TGACAT  172
                ||||
Sbjct  13745  ACACAT  13740


>ref|NW_003033841.1|  Schistosoma mansoni genome sequence supercontig Smp_scaff010734
Length=2029

 Score = 42.8 bits (46),  Expect = 0.65
 Identities = 49/66 (74%), Gaps = 0/66 (0%)
 Strand=Plus/Minus

Query  107  TCACAAAAACAGCACTCCACTGGACCCCAGAAGGTAAAAGAAAGAGAGGAAGACCTAAAA  166
            |||||| || ||| |||   ||||  || |||||| |||| |  ||||||||||| || |
Sbjct  465  TCACAAGAAAAGCCCTCACATGGAATCCTGAAGGTCAAAGGAGAAGAGGAAGACCAAAGA  406

Query  167  TGACAT  172
              ||||
Sbjct  405  ACACAT  400


>ref|NW_003033601.1|  Schistosoma mansoni genome sequence supercontig Smp_scaff008383
```

[illegible]
Length=1139

```
 Score = 42.8 bits (46),  Expect = 0.65
 Identities = 49/66 (74%), Gaps = 0/66 (0%)
 Strand=Plus/Plus

Query  107  TCACAAAAACAGCACTCCACTGGACCCCAGAAGGTAAAAGAAAGAGAGGAAGACCTAAAA  166
            |||||| |  |||||||   ||||  || |||||| |||| |  ||||||||||| || |
Sbjct  448  TCACAAGACAAGCACTCACATGGAATCCTGAAGGTCAAAGGAGAAGAGGAAGACCAAAGA  507

Query  167  TGACAT  172
              ||||
Sbjct  508  ACACAT  513
```

>**ref|NW_003032283.1|** Schistosoma mansoni genome sequence supercontig Smp_scaff010563
Length=2314

```
 Score = 42.8 bits (46),  Expect = 0.65
 Identities = 49/66 (74%), Gaps = 0/66 (0%)
 Strand=Plus/Minus

Query  107   TCACAAAAACAGCACTCCACTGGACCCCAGAAGGTAAAAGAAAGAGAGGAAGACCTAAAA  166
             |||||| |  |||||||   ||||  || |||||| |||| |  ||||||||||| | ||
Sbjct  1619  TCACAAGACAAGCACTCACATGGAATCCTGAAGGTCAAAGGAGAAGAGGAAGACCAACAA  1560

Query  167   TGACAT  172
               ||||
Sbjct  1559  ACACAT  1554
```

>**ref|NW_003031788.1|** Schistosoma mansoni genome sequence supercontig Smp_scaff014561
Length=1308

```
 Score = 42.8 bits (46),  Expect = 0.65
 Identities = 37/46 (80%), Gaps = 0/46 (0%)
 Strand=Plus/Minus

Query  127   TGGACCCCAGAAGGTAAAAGAAAGAGAGGAAGACCTAAAATGACAT  172
             ||||  || |||||  |||| || ||||||||||| ||||| ||||
Sbjct  1230  TGGAATCCTGAAGGCCAAAGGAAAAGAGGAAGACCAAAAATCACAT  1185
```

>**ref|NW_003029805.1|** Schistosoma mansoni genome sequence supercontig Smp_scaff018657
Length=5303

```
 Score = 42.8 bits (46),  Expect = 0.65
 Identities = 61/85 (71%), Gaps = 2/85 (2%)
 Strand=Plus/Minus

Query  106   ATCACAAAAACAGCACTCCACTGGACCCCAGAAGGTAAAAGAAAGAGAGGAAGACCTAAA  165
             ||||||| |  ||| |||   ||||  |  |||||| |||| |  ||||||||||| ||
Sbjct  4189  ATCACAAGACAAGCCCTCACATGGAATCTTGAAGGTCAAAGGAGAAGAGGAAGACCAAAG  4130

Query  166   ATGACAT--GGAGAAGAACTGTAGA  188
             |  ||||   | |||||| ||||||
Sbjct  4129  AACACATTACGCGAAGAAATGTAGA  4105
```

>**ref|NW_003029774.1|** Schistosoma mansoni genome sequence supercontig Smp_scaff018615
Length=6755

```
 Score = 42.8 bits (46),  Expect = 0.65
 Identities = 84/121 (69%), Gaps = 5/121 (4%)
 Strand=Plus/Minus

Query  54   AAGAAGACGCTGGAGGTGGATTCGGCATGTCCTACGGAAAGG--CCAGGGGGATATCACA  111
            |||||| ||||||| ||||||| |||||  | |  |||||    |||   |  | |||||
Sbjct  732  AAGAAG-CGCTGGAAGTGGATTGGGCATACCTTGAGGAAATCACCCAATTG--TGTCACA  676

Query  112  AAAACAGCACTCCACTGGACCCCAGAAGGTAAAAGAAAGAGAGGAAGACCTAAAATGACA  171
            | |  ||| |||   ||||  || ||||||  ||| |  ||||||||||| || |  |||
Sbjct  675  AGACAAGCCCTCACATGGAATCCTGAAGGTCGAAGGAGAAGAGGAAGACCAAAGAACACA  616

Query  172  T  172
            |
Sbjct  615  T  615
```

>**ref|NW_003029322.1|** Schistosoma mansoni genome sequence supercontig Smp_scaff010146
Length=1166

```
 Score = 42.8 bits (46),  Expect = 0.65
 Identities = 49/66 (74%), Gaps = 0/66 (0%)
 Strand=Plus/Minus

Query  107   TCACAAAAACAGCACTCCACTGGACCCCAGAAGGTAAAAGAAAGAGAGGAAGACCTAAAA  166
             |||||| |  ||| |||   ||||  || |||||| |||| || ||||||||||| || |
Sbjct  1034  TCACAAGACAAGCCCTCACGTGGAATCCTGAAGGTCAAAGGAAAAGAGGAAGACCAAAGA  975

Query  167   TGACAT  172
               ||||
Sbjct  974   ACACAT  969
```

>**ref|NW_003029272.1|** Schistosoma mansoni genome sequence supercontig Smp_scaff010092
Length=1058

```
 Score = 42.8 bits (46),  Expect = 0.65
```

```
 Identities = 49/66 (74%), Gaps = 0/66 (0%)
 Strand=Plus/Minus

Query  107  TCACAAAAACAGCACTCCACTGGACCCCAGAAGGTAAAAGAAAGAGAGGAAGACCTAAAA  166
            |||||| |  ||| |||   ||||  || |||||| ||||||  ||||||||||| || |
Sbjct  981  TCACAAGACAAGCCCTCACATGGAATCCTGAAGGTCAAAGAAGAAGAGGAAGACCAAAGA  922

Query  167  TGACAT  172
              ||||
Sbjct  921  ACACAT  916
```

>ref|NW_003026612.1| D Schistosoma mansoni genome sequence supercontig Smp_scaff000592
Length=277612

Sort alignments for this subject sequence by:
E value **Score** **Percent identity**
Query start position **Subject start position**

Features flanking this part of subject sequence:
7830 bp at 3' side: phosphate transporter

```
 Score = 42.8 bits (46),  Expect = 0.65
 Identities = 49/66 (74%), Gaps = 0/66 (0%)
 Strand=Plus/Plus

Query  107   TCACAAAAACAGCACTCCACTGGACCCCAGAAGGTAAAAGAAAGAGAGGAAGACCTAAAA  166
             |||||| |  ||| |||   ||||  || |||||| |||| |  ||||||||||| ||||
Sbjct  5299  TCACAAGACAAGCCCTCACATGGAATCCTGAAGGTGAAAGGAGAAGAGGAAGACCAAAAA  5358

Query  167   TGACAT  172
               ||||
Sbjct  5359  ACACAT  5364
```

Features in this part of subject sequence:
replication factor C / DNA polymerase III gamma-tau subunit

```
 Score = 42.8 bits (46),  Expect = 0.65
 Identities = 49/66 (74%), Gaps = 0/66 (0%)
 Strand=Plus/Plus

Query  107     TCACAAAAACAGCACTCCACTGGACCCCAGAAGGTAAAAGAAAGAGAGGAAGACCTAAAA  166
               |||||| |  ||| |||   ||||  || |||||| |||| |  ||||||||||| || |
Sbjct  166281  TCACAAGACAAGCCCTCACATGGAATCCTGAAGGTCAAAGGAGAAGAGGAAGACCAAAGA  166340

Query  167     TGACAT  172
                |||||
Sbjct  166341  AGACAT  166346
```

Features flanking this part of subject sequence:
41285 bp at 5' side: phosphate transporter
13773 bp at 3' side: hypothetical protein

```
 Score = 39.2 bits (42),  Expect = 7.9
 Identities = 44/58 (75%), Gaps = 1/58 (1%)
 Strand=Plus/Plus

Query  107    TCACAAAAACAGCACTCCACTGGACCCCAGAAGGTAAAAGAAAGAGAGGAAGACCTAA  164
              ||||||||  ||| |||   ||||  || ||| |  ||||||| ||||||||||| ||
Sbjct  56692  TCACAAAATAAGC-CTCTCATGGAATCCTGAAAGCTAAAGAAAAAGAGGAAGACCAAA  56748
```

Features flanking this part of subject sequence:
1820 bp at 5' side: polyhomeotic
4744 bp at 3' side: replication factor C / DNA polymerase III gamma-tau subunit

```
 Score = 39.2 bits (42),  Expect = 7.9
 Identities = 48/66 (72%), Gaps = 0/66 (0%)
 Strand=Plus/Minus

Query  107     TCACAAAAACAGCACTCCACTGGACCCCAGAAGGTAAAAGAAAGAGAGGAAGACCTAAAA  166
               |||||| |  ||| |||   ||||  || |||||| |||| |  ||||||||||| || |
Sbjct  155478  TCACAAGACAAGCCCTCACATGGAATCCTGAAGGTCAAAGGAGAAGAGGAAGACCAAAGA  155419

Query  167     TGACAT  172
                 ||||
Sbjct  155418  ACACAT  155413
```

>ref|NW_003026608.1| D Schistosoma mansoni genome sequence supercontig Smp_scaff000579
Length=133508

```
 Score = 42.8 bits (46),  Expect = 0.65
 Identities = 44/58 (75%), Gaps = 0/58 (0%)
 Strand=Plus/Plus

Query  107   TCACAAAAACAGCACTCCACTGGACCCCAGAAGGTAAAAGAAAGAGAGGAAGACCTAA  164
             |||||| |  ||| |||   ||||  || |||||| |||| || ||||||||||| ||
Sbjct  1659  TCACAAGACAAGCCCTCACATGGAATCCTGAAGGTCAAAGGAAAAGAGGAAGACCGAA  1716
```

>ref|NW_003026458.1| D Schistosoma mansoni genome sequence supercontig Smp_scaff000069
Length=810511

Sort alignments for this subject sequence by:
E value **Score** **Percent identity**

Query start position Subject start position

Features flanking this part of subject sequence:
33498 bp at 3' side: hypothetical protein

Score = 42.8 bits (46), Expect = 0.65
Identities = 49/66 (74%), Gaps = 0/66 (0%)
Strand=Plus/Minus

```
Query  107    TCACAAAAACAGCACTCCACTGGACCCCAGAAGGTAAAAGAAAGAGAGGAAGACCTAAAA  166
              |||||| |  ||| |||   ||||  || |||||| |||| | |||||||||||| || |
Sbjct  32256  TCACAAGACAAGCCCTCACATGGAATCCTGAAGGTCAAAGGAGGAGAGGAAGACCAAAGA  32197

Query  167    TGACAT  172
                ||||
Sbjct  32196  ACACAT  32191
```

Features in this part of subject sequence:
adenomatous polyposis coli protein

Score = 42.8 bits (46), Expect = 0.65
Identities = 49/66 (74%), Gaps = 0/66 (0%)
Strand=Plus/Plus

```
Query  107     TCACAAAAACAGCACTCCACTGGACCCCAGAAGGTAAAAGAAAGAGAGGAAGACCTAAAA  166
               |||||| || || ||||   ||||  || |||||  ||||||  ||||||||||| || |
Sbjct  230007  TCACAAGAAAAGTACTCACATGGAGTCCTGAAGGAGAAAGAAGAAGAGGAAGACCAAAGA  230066

Query  167     TGACAT  172
                 ||||
Sbjct  230067  ACACAT  230072
```

Features flanking this part of subject sequence:
27335 bp at 5' side: hypothetical protein

Score = 42.8 bits (46), Expect = 0.65
Identities = 49/65 (75%), Gaps = 2/65 (3%)
Strand=Plus/Minus

```
Query  107     TCACAAAAACAGCACTCCACTGGACCCCAGAAGGTAAAAGAAAGAGAGGAAGACCTAAAA  166
               |||||| |  ||| ||| | ||||  || |||||| || |||  |||||||||||  |||
Sbjct  753042  TCACAAGATAAGCCCTCAAATGGAATCCTGAAGGTCAATGAAGAAGAGGAAGACC--AAA  752985

Query  167     TGACA  171
                ||||
Sbjct  752984  GGACA  752980
```

Features flanking this part of subject sequence:
41286 bp at 3' side: hypothetical protein

Score = 39.2 bits (42), Expect = 7.9
Identities = 36/46 (78%), Gaps = 0/46 (0%)
Strand=Plus/Minus

```
Query  127    TGGACCCCAGAAGGTAAAAGAAAGAGAGGAAGACCTAAAATGACAT  172
              ||||  || |||||| |||| || ||||||||||| || |  ||||
Sbjct  24468  TGGAATCCTGAAGGTCAAAGGAAAAGAGGAAGACCAAAGAACACAT  24423
```

Features flanking this part of subject sequence:
9527 bp at 5' side: hypothetical protein

Score = 39.2 bits (42), Expect = 7.9
Identities = 48/66 (72%), Gaps = 0/66 (0%)
Strand=Plus/Plus

```
Query  107     TCACAAAAACAGCACTCCACTGGACCCCAGAAGGTAAAAGAAAGAGAGGAAGACCTAAAA  166
               |||||| |  ||| |||   ||||  || |||||| |||| |  ||||||||||| || |
Sbjct  735172  TCACAAGACAAGCCCTCACATGGAATCCTGAAGGTCAAAGGAGAAGAGGAAGACCAAAGA  735231

Query  167     TGACAT  172
                 ||||
Sbjct  735232  ACACAT  735237
```

>**ref|NW_003026430.1|** Schistosoma mansoni genome sequence supercontig Smp_scaff003036
Length=2657

Score = 42.8 bits (46), Expect = 0.65
Identities = 52/70 (74%), Gaps = 2/70 (2%)
Strand=Plus/Plus

```
Query  127   TGGACCCCAGAAGGTAAAAGAAAGAGAGGAAGACCTAAAATGACATGGAGAA--GAACTG  184
             ||||  || ||||||||||| |||||||||||| | || |    ||| ||| |  ||| ||
Sbjct  1037  TGGAATCCTGAAGGTAAAAGGAAGAGAGGAAGAACAAAGAACGCATTGAGCAGTGAATTG  1096

Query  185   TAGAGGCAGA  194
              ||| | |||
Sbjct  1097  GAGACGGAGA  1106
```

>**ref|NW_003024332.1|** Schistosoma mansoni genome sequence supercontig Smp_scaff013696
Length=1128

Score = 42.8 bits (46), Expect = 0.65
Identities = 49/66 (74%), Gaps = 0/66 (0%)

```
 Strand=Plus/Plus

Query  107  TCACAAAAACAGCACTCCACTGGACCCCAGAAGGTAAAAGAAAGAGAGGAAGACCTAAAA  166
            |||||| |  |||||||   ||||  || |||||| |||| |  ||||||||||| || |
Sbjct  431  TCACAAGACAAGCACTCACATGGAATCCTGAAGGTCAAAGGAGAAGAGGAAGACCAAAGA  490

Query  167  TGACAT  172
              ||||
Sbjct  491  ACACAT  496
```

>**ref|NW_003023815.1|** Schistosoma mansoni genome sequence supercontig Smp_scaff015661
Length=1668

```
 Score = 42.8 bits (46),  Expect = 0.65
 Identities = 49/66 (74%), Gaps = 0/66 (0%)
 Strand=Plus/Minus

Query  107   TCACAAAAACAGCACTCCACTGGACCCCAGAAGGTAAAAGAAAGAGAGGAAGACCTAAAA  166
             ||||||||  ||| |||   |||| ||| |||||| || | |  ||||||||||| || |
Sbjct  1429  TCACAAAACAAGCCCTCACATGGAACCCTGAAGGTCAATGGAGAAGAGGAAGACCAAAGA  1370

Query  167   TGACAT  172
               ||||
Sbjct  1369  ACACAT  1364
```

>**ref|NW_003023484.1|** Schistosoma mansoni genome sequence supercontig Smp_scaff017714
Length=1873

```
 Score = 42.8 bits (46),  Expect = 0.65
 Identities = 37/46 (80%), Gaps = 0/46 (0%)
 Strand=Plus/Plus

Query  127   TGGACCCCAGAAGGTAAAAGAAAGAGAGGAAGACCTAAAATGACAT  172
             ||||  || |||||| |||| || ||||||||||| ||||  ||||
Sbjct  1497  TGGAATCCTGAAGGTCAAAGGAAAAGAGGAAGACCAAAAAACACAT  1542
```

>**emb|FN357529.1|** Schistosoma mansoni genome sequence supercontig Smp_scaff000238
Length=434718

Sort alignments for this subject sequence by:
E value **Score** **Percent identity**
Query start position **Subject start position**

Features flanking this part of subject sequence:
915 bp at 5' side: protein arginine n-methyltransferase, putative
1387 bp at 3' side: dynein light chain, putative

```
 Score = 42.8 bits (46),  Expect = 0.65
 Identities = 49/66 (74%), Gaps = 0/66 (0%)
 Strand=Plus/Minus

Query  107     TCACAAAAACAGCACTCCACTGGACCCCAGAAGGTAAAAGAAAGAGAGGAAGACCTAAAA  166
               |||||| |  ||| |||   |||| ||| |||||| |||| |  ||||||||||| || |
Sbjct  313371  TCACAAGACAAGCCCTCACATGGAACCCTGAAGGTCAAAGGAGAAGAGGAAGACCAAAGA  313312

Query  167     TGACAT  172
                 ||||
Sbjct  313311  ACACAT  313306
```

Features flanking this part of subject sequence:
21084 bp at 5' side: hypothetical protein

```
 Score = 39.2 bits (42),  Expect = 7.9
 Identities = 48/66 (72%), Gaps = 0/66 (0%)
 Strand=Plus/Plus

Query  107     TCACAAAAACAGCACTCCACTGGACCCCAGAAGGTAAAAGAAAGAGAGGAAGACCTAAAA  166
               |||||| |  ||| |||   ||||  || |||||| |||| |  ||||||||||| || |
Sbjct  426498  TCACAAGACAAGCCCTCACATGGAATCCTGAAGGTCAAAGGAGAAGAGGAAGACCAAAGA  426557

Query  167     TGACAT  172
                 ||||
Sbjct  426558  ACACAT  426563
```

>**emb|FN357369.1|** Schistosoma mansoni genome sequence supercontig Smp_scaff000078
Length=2523220

Sort alignments for this subject sequence by:
E value **Score** **Percent identity**
Query start position **Subject start position**

Features flanking this part of subject sequence:
27633 bp at 5' side: hypothetical protein
6527 bp at 3' side: Septation ring formation regulator ezrA, putative

```
 Score = 42.8 bits (46),  Expect = 0.65
 Identities = 49/66 (74%), Gaps = 0/66 (0%)
 Strand=Plus/Minus

Query  107      TCACAAAAACAGCACTCCACTGGACCCCAGAAGGTAAAAGAAAGAGAGGAAGACCTAAAA  166
                |||||| |  ||| |||   ||||  || |||||| |||| |  ||||||||||| ||||
Sbjct  2115756  TCACAAGACAAGCCCTCACATGGAATCCTGAAGGTCAAAGGAGAAGAGGAAGACCAAAAA  2115697
```

```
Query  167      TGACAT  172
                  ||||
Sbjct  2115696  ACACAT  2115691
```

Features flanking this part of subject sequence:
2127 bp at 5' side: hypothetical protein
29112 bp at 3' side: ras GTP exchange factor, son of sevenless, putative

Score = 39.2 bits (42), Expect = 7.9
Identities = 48/66 (72%), Gaps = 0/66 (0%)
Strand=Plus/Minus

```
Query  107      TCACAAAAACAGCACTCCACTGGACCCCAGAAGGTAAAAGAAAGAGAGGAAGACCTAAAA  166
                |||||| |  ||| |||   ||||  || ||||||  ||| || ||||||||||| || |
Sbjct  1067368  TCACAAGACAAGCCCTCACATGGAATCCTGAAGGTCGAAGGAAAAGAGGAAGACCAAAGA  1067309

Query  167      TGACAT  172
                  ||||
Sbjct  1067308  ACACAT  1067303
```

>**emb|FN359289.1|** Schistosoma mansoni genome sequence supercontig Smp_scaff001998
Length=63486

Score = 42.8 bits (46), Expect = 0.65
Identities = 49/66 (74%), Gaps = 0/66 (0%)
Strand=Plus/Minus

```
Query  107    TCACAAAAACAGCACTCCACTGGACCCCAGAAGGTAAAAGAAAGAGAGGAAGACCTAAAA  166
              |||||| |  ||| |||   || |  || ||| || ||||||||||||||||||| || |
Sbjct  29987  TCACAAGACAAGCCCTCACATGAAATCCTGAAAGTGAAAGAAAGAGAGGAAGACCAAAGA  29928

Query  167    TGACAT  172
                ||||
Sbjct  29927  ACACAT  29922
```

>**emb|FN359287.1|** Schistosoma mansoni genome sequence supercontig Smp_scaff001996
Length=334775

Features flanking this part of subject sequence:
56869 bp at 5' side: WD-repeat protein, putative
9200 bp at 3' side: hypothetical protein

Score = 42.8 bits (46), Expect = 0.65
Identities = 49/66 (74%), Gaps = 0/66 (0%)
Strand=Plus/Plus

```
Query  107     TCACAAAAACAGCACTCCACTGGACCCCAGAAGGTAAAAGAAAGAGAGGAAGACCTAAAA  166
               |||||| |  ||| |||   ||||  || |||||| |||| || ||||||||| | ||||
Sbjct  274534  TCACAACACAAGCCCTCACATGGAATCCTGAAGGTCAAAGGAAAAGAGGAAGATCAAAAA  274593

Query  167     TGACAT  172
                 ||||
Sbjct  274594  ACACAT  274599
```

>**emb|FN359282.1|** Schistosoma mansoni genome sequence supercontig Smp_scaff001991
Length=162506

Sort alignments for this subject sequence by:
E value **Score** **Percent identity**
Query start position **Subject start position**

Score = 42.8 bits (46), Expect = 0.65
Identities = 49/66 (74%), Gaps = 0/66 (0%)
Strand=Plus/Minus

```
Query  107    TCACAAAAACAGCACTCCACTGGACCCCAGAAGGTAAAAGAAAGAGAGGAAGACCTAAAA  166
              |||||| |  |||||||   ||||  || |||||| |||| |  ||||||||||| || |
Sbjct  11800  TCACAAGACGAGCACTCACTTGGAATCCTGAAGGTCAAAGGAGAAGAGGAAGACCAAAGA  11741

Query  167    TGACAT  172
                ||||
Sbjct  11740  ACACAT  11735
```

Score = 39.2 bits (42), Expect = 7.9
Identities = 48/66 (72%), Gaps = 0/66 (0%)
Strand=Plus/Plus

```
Query  107    TCACAAAAACAGCACTCCACTGGACCCCAGAAGGTAAAAGAAAGAGAGGAAGACCTAAAA  166
              |||||| |  ||| |||   ||||  || |||||  |||| || ||||||||||| || |
Sbjct  31514  TCACAAGACAAGCCCTCACATGGAATCCTGAAGGCCAAAGGAAAAGAGGAAGACCAAAGA  31573

Query  167    TGACAT  172
                ||||
Sbjct  31574  ACACAT  31579
```

>**emb|FN359228.1|** Schistosoma mansoni genome sequence supercontig Smp_scaff001937
Length=84969

Score = 42.8 bits (46), Expect = 0.65
Identities = 76/108 (70%), Gaps = 5/108 (4%)
Strand=Plus/Minus

```
Query  54     AAGAAGACGCTGGAGGTGGATTCGGCATGTCCTACGGAAA--GGCCAGGGGGATATCACA  111
              |||||| ||||||| ||||||| ||||     || |||||  | |||   |  | ||| |
Sbjct  65230  AAGAAG-CGCTGGAAGTGGATTGGGCACACTTTAAGGAAATCGCCCAATTG--TGTCATA  65174

Query  112    AAAACAGCACTCCACTGGACCCCAGAAGGTAAAAGAAAGAGAGGAAGA  159
              | |  |||||||   ||||  || |||||| |||| |  |||||||||
Sbjct  65173  AGACAAGCACTCACATGGAATCCTGAAGGTCAAAGGAGAAGAGGAAGA  65126
```

>**emb|FN358757.1|** D Schistosoma mansoni genome sequence supercontig Smp_scaff001466
Length=55385

```
 Score = 42.8 bits (46),  Expect = 0.65
 Identities = 49/66 (74%), Gaps = 0/66 (0%)
 Strand=Plus/Plus

Query  107    TCACAAAAACAGCACTCCACTGGACCCCAGAAGGTAAAAGAAAGAGAGGAAGACCTAAAA  166
              |||||| |  ||| |||   ||||  || |||||  |||| || ||||||||||| ||||
Sbjct  52247  TCACAAGACAAGCCCTCACATGGAATCCTGAAGGCCAAAGGAAAAGAGGAAGACCAAAAA  52306

Query  167    TGACAT  172
                ||||
Sbjct  52307  ACACAT  52312
```

>**emb|FN357920.1|** D Schistosoma mansoni genome sequence supercontig Smp_scaff000629
Length=1064850

Sort alignments for this subject sequence by:
E value **Score** **Percent identity**
Query start position **Subject start position**

Features in this part of subject sequence:
tetraspanin 42, invertebrate, putative

```
 Score = 42.8 bits (46),  Expect = 0.65
 Identities = 44/58 (75%), Gaps = 0/58 (0%)
 Strand=Plus/Minus

Query  107     TCACAAAAACAGCACTCCACTGGACCCCAGAAGGTAAAAGAAAGAGAGGAAGACCTAA  164
               |||||| |  |||||||   ||||   | |||||| |||| || ||||||||||| ||
Sbjct  564183  TCACAAGACAAGCACTCACATGGAATTCTGAAGGTCAAAGGAAAAGAGGAAGACCAAA  564126
```

Features flanking this part of subject sequence:
39148 bp at 5' side: serine/threonine kinase
49168 bp at 3' side: dep domain containing protein, putative

```
 Score = 42.8 bits (46),  Expect = 0.65
 Identities = 43/56 (76%), Gaps = 0/56 (0%)
 Strand=Plus/Minus

Query  117     AGCACTCCACTGGACCCCAGAAGGTAAAAGAAAGAGAGGAAGACCTAAAATGACAT  172
               ||| ||| | ||||  || |||||| |||| | |||||||||||| || | |  ||||
Sbjct  908579  AGCCCTCAAATGGAATCCTGAAGGTCAAAGGAGGAGAGGAAGACCAAAGAACACAT  908524
```

Features in this part of subject sequence:
dep domain containing protein, putative

```
 Score = 41.0 bits (44),  Expect = 2.3
 Identities = 51/70 (72%), Gaps = 0/70 (0%)
 Strand=Plus/Minus

Query  107      TCACAAAAACAGCACTCCACTGGACCCCAGAAGGTAAAAGAAAGAGAGGAAGACCTAAAA  166
                |||||| |  ||| |||   ||||  || |||||  |||| || |||||||| || ||||
Sbjct  1011973  TCACAAGACAAGCCCTCACATGGAATCCTGAAGGCCAAAGTAAAAGAGGAAGGCCAAAAA  1011914

Query  167      TGACATGGAG  176
                  |||| |||
Sbjct  1011913  ACACATTGAG  1011904
```

Features in this part of subject sequence:
serine/threonine kinase

```
 Score = 39.2 bits (42),  Expect = 7.9
 Identities = 83/121 (68%), Gaps = 5/121 (4%)
 Strand=Plus/Plus

Query  54      AAGAAGACGCTGGAGGTGGATTCGGCATGTCCTACGGAAAGG--CCAGGGGGATATCACA  111
               |||||| ||||||| ||||||| ||||   | |  ||||||   |||   |  |  ||||
Sbjct  149955  AAGAAG-CGCTGGAAGTGGATTGGGCACACCTTGAGGAAAGCACCCAATCGCGT--CACA  150011

Query  112     AAAACAGCACTCCACTGGACCCCAGAAGGTAAAAGAAAGAGAGGAAGACCTAAAATGACA  171
               | |  ||| |||   ||||  || |||||  |||| |  ||||||||||| || |  |||
Sbjct  150012  AGACAAGCCCTCACATGGAATCCTGAAGGCCAAAGGAGAAGAGGAAGACCAAAGAACACA  150071

Query  172     T  172
               |
Sbjct  150072  T  150072
```

Features in this part of subject sequence:
phospholipase C-like protein 1, plc-1

```
 Score = 39.2 bits (42),  Expect = 7.9
```

```
 Identities = 48/66 (72%), Gaps = 0/66 (0%)
 Strand=Plus/Minus

Query  107     TCACAAAAACAGCACTCCACTGGACCCCAGAAGGTAAAAGAAAGAGAGGAAGACCTAAAA  166
               |||||| |  ||| |||   ||||  || |||||  |||| || ||||||||||| || |
Sbjct  277703  TCACAAGACAAGCCCTCACATGGAGTCCTGAAGGCCAAAGGAAAAGAGGAAGACCAAAGA  277644

Query  167     TGACAT  172
                 ||||
Sbjct  277643  ACACAT  277638
```

 Features in this part of subject sequence:
   tetraspanin 42, invertebrate, putative

```
 Score = 39.2 bits (42),  Expect = 7.9
 Identities = 48/66 (72%), Gaps = 0/66 (0%)
 Strand=Plus/Minus

Query  107     TCACAAAAACAGCACTCCACTGGACCCCAGAAGGTAAAAGAAAGAGAGGAAGACCTAAAA  166
               |||||| |  | |||||   ||||  || |||||  |||| | |||||||||||| || |
Sbjct  568671  TCACAAGACAAACACTCACATGGAATCCTGAAGGCCAAAGGAGGAGAGGAAGACCAAAGA  568612

Query  167     TGACAT  172
                 ||||
Sbjct  568611  ACACAT  568606
```

>**emb|FN357900.1|** D Schistosoma mansoni genome sequence supercontig Smp_scaff000609
Length=362439

 Features in this part of subject sequence:
   endosomal trafficking protein, putative

```
 Score = 42.8 bits (46),  Expect = 0.65
 Identities = 49/66 (74%), Gaps = 0/66 (0%)
 Strand=Plus/Plus

Query  107    TCACAAAAACAGCACTCCACTGGACCCCAGAAGGTAAAAGAAAGAGAGGAAGACCTAAAA  166
              |||||| |  ||| |||   ||||  || |||||| |||| |  ||||||||||| ||||
Sbjct  84902  TCACAAGACAAGCCCTCACATGGAATCCTGAAGGTCAAAGGAGAAGAGGAAGACCAAAAA  84961

Query  167    TGACAT  172
                ||||
Sbjct  84962  ACACAT  84967
```

>**emb|FN357865.1|** D Schistosoma mansoni genome sequence supercontig Smp_scaff000574
Length=338805

Sort alignments for this subject sequence by:
  E value **Score** **Percent identity**
  Query start position **Subject start position**

 Features in this part of subject sequence:
   inositol monophosphatase
   inositol monophosphatase

```
 Score = 42.8 bits (46),  Expect = 0.65
 Identities = 43/55 (78%), Gaps = 1/55 (1%)
 Strand=Plus/Minus

Query  127     TGGACCCCAGAAGGTAAAAGAAAGAGAGGAAGACCTAAAATGACATGGAGAAGAA  181
               ||||  || |||||| |||| || |||||||| || || |  |||| ||||||||
Sbjct  157655  TGGAATCCTGAAGGTCAAAGGAAAAGAGGAAGGCCAAAGAACACAT-GAGAAGAA  157602
```

 Features flanking this part of subject sequence:
   1945 bp at 5' side: phosphate transporter, putative
   23314 bp at 3' side: expressed protein

```
 Score = 39.2 bits (42),  Expect = 7.9
 Identities = 48/66 (72%), Gaps = 0/66 (0%)
 Strand=Plus/Plus

Query  107     TCACAAAAACAGCACTCCACTGGACCCCAGAAGGTAAAAGAAAGAGAGGAAGACCTAAAA  166
               |||||| |  ||| |||   ||||  || |||||| |||| |  ||||||||||| || |
Sbjct  196651  TCACAAGACAAGCCCTCACATGGAATCCTGAAGGTCAAAGGAGAAGAGGAAGACCAAAGA  196710

Query  167     TGACAT  172
                 ||||
Sbjct  196711  ACACAT  196716
```

>**emb|FN357718.1|** D Schistosoma mansoni genome sequence supercontig Smp_scaff000427
Length=632148

Sort alignments for this subject sequence by:
  E value **Score** **Percent identity**
  Query start position **Subject start position**

 Features flanking this part of subject sequence:
   9394 bp at 5' side: pseudouridylate synthase, putative
   647 bp at 3' side: serine/threonine kinase

```
 Score = 42.8 bits (46),  Expect = 0.65
 Identities = 49/66 (74%), Gaps = 0/66 (0%)
 Strand=Plus/Plus
```

```
Query  107     TCACAAAAACAGCACTCCACTGGACCCCAGAAGGTAAAAGAAAGAGAGGAAGACCTAAAA  166
               |||||| |  ||| |||   ||||  || |||||| |||| | |||||||||||| || |
Sbjct  602602  TCACAAGACAAGCCCTCACATGGAATCCTGAAGGTCAAAGGAGGAGAGGAAGACCAAAGA  602661

Query  167     TGACAT  172
                 ||||
Sbjct  602662  ACACAT  602667
```

Features flanking this part of subject sequence:
13924 bp at 5' side: serine/threonine protein kinase
115238 bp at 3' side: expressed protein

```
 Score = 39.2 bits (42),  Expect = 7.9
 Identities = 48/66 (72%), Gaps = 0/66 (0%)
 Strand=Plus/Minus

Query  107     TCACAAAAACAGCACTCCACTGGACCCCAGAAGGTAAAAGAAAGAGAGGAAGACCTAAAA  166
               |||||| |  ||| |||   ||||  || |||||| |||| |  ||||||||||| || |
Sbjct  228747  TCACAAGACAAGCCCTCGCATGGAATCCTGAAGGTCAAAGGAGAAGAGGAAGACCAAAGA  228688

Query  167     TGACAT  172
                 ||||
Sbjct  228687  ACACAT  228682
```

>**emb|FN357715.1|** Schistosoma mansoni genome sequence supercontig Smp_scaff000424
Length=325309

Features in this part of subject sequence:
60S ribosomal protein L19, putative

```
 Score = 42.8 bits (46),  Expect = 0.65
 Identities = 49/66 (74%), Gaps = 0/66 (0%)
 Strand=Plus/Minus

Query  107     TCACAAAAACAGCACTCCACTGGACCCCAGAAGGTAAAAGAAAGAGAGGAAGACCTAAAA  166
               |||||| |  ||| |||   ||||  || |||||| |||| || ||||||||||| || |
Sbjct  218444  TCACAAGACAAGCCCTCACATGGAATCCTGAAGGTCAAAGGAAAAGAGGAAGACCAAAGA  218385

Query  167     TGACAT  172
                 ||||
Sbjct  218384  ACACAT  218379
```

>**emb|FN357703.1|** Schistosoma mansoni genome sequence supercontig Smp_scaff000412
Length=194211

Sort alignments for this subject sequence by:
E value **Score** **Percent identity**
Query start position **Subject start position**

```
 Score = 42.8 bits (46),  Expect = 0.65
 Identities = 49/66 (74%), Gaps = 0/66 (0%)
 Strand=Plus/Minus

Query  107    TCACAAAAACAGCACTCCACTGGACCCCAGAAGGTAAAAGAAAGAGAGGAAGACCTAAAA  166
              |||||| |  ||| |||   ||||  || |||||| |||| || ||||||||||| || |
Sbjct  99639  TCACAAGACAAGCCCTCATATGGAATCCTGAAGGTCAAAGGAAAAGAGGAAGACCAAAGA  99580

Query  167    TGACAT  172
                ||||
Sbjct  99579  ACACAT  99574


 Score = 42.8 bits (46),  Expect = 0.65
 Identities = 84/121 (69%), Gaps = 5/121 (4%)
 Strand=Plus/Minus

Query  54      AAGAAGACGCTGGAGGTGGATTCGGCATGTCCTACGGAAAGG--CCAGGGGGATATCACA  111
               |||||| ||||||| ||||||| |||||  | |  |||||    |||   |  | |||||
Sbjct  104848  AAGAAG-CGCTGGAAGTGGATTGGGCATACCTTGAGGAAATCACCCAATTG--TGTCACA  104792

Query  112     AAAACAGCACTCCACTGGACCCCAGAAGGTAAAAGAAAGAGAGGAAGACCTAAAATGACA  171
               | |  |||  |||   ||||  || ||||||  ||| |  ||||||||||| || |  |||
Sbjct  104791  AGACAAGCCCTCACATGGAATCCTGAAGGTCGAAGGAGAAGAGGAAGACCAAAGAACACA  104732

Query  172     T  172
               |
Sbjct  104731  T  104731
```

>**emb|FN357691.1|** Schistosoma mansoni genome sequence supercontig Smp_scaff000400
Length=249630

Features flanking this part of subject sequence:
23685 bp at 5' side: G-protein coupled receptor fragment, putative
68418 bp at 3' side: coatomer epsilon subunit, putative

```
 Score = 42.8 bits (46),  Expect = 0.65
 Identities = 49/66 (74%), Gaps = 0/66 (0%)
 Strand=Plus/Minus

Query  107    TCACAAAAACAGCACTCCACTGGACCCCAGAAGGTAAAAGAAAGAGAGGAAGACCTAAAA  166
              |||||| |  ||| |||   ||||  || |||||| ||||||  ||||||||||| || |
Sbjct  82781  TCACAAGACAAGCCCTCACATGGAATCCTGAAGGTCAAAGAAGAAGAGGAAGACCAAAGA  82722
```

```
Query  167    TGACAT  172
                ||||
Sbjct  82721  ACACAT  82716
```

Select All Get selected sequences Distance tree of results Multiple alignment NEW
