# Supplementary material for: Promoter Complexity and Tissue-Specific Expression of Stress Response Components in Mytilus galloprovincialis, a Sessile Marine Invertebrate Species
Source: PLoS Comput Biol. 2010 Jul 8;6(7):e1000847. doi: 10.1371/journal.pcbi.1000847 (PMC2900285; doi:10.1371/journal.pcbi.1000847)
Supplement: Protocol S1 — 18 Supplement files plus an index file: 3 Supplementary figures, 2 Supplementary tables - referenced in text as Protocol S1; index provided with an explanation of the directory contents. (5.18 MB ZIP) [file pcbi.1000847.s001.zip › SUPPLEMENTS18/SupplFigure2.2a.pdf]

BLAST Basic Local Alignment Search Tool

-

Edit and Resubmit Save Search Strategies Formatting options Download

Nucleotide Sequence (328 letters)

Results for: lcl|56899 None(201bp)

Your BLAST job specified more than one input sequence. This box lets you choose which input sequence to show BLAST results for.

Query ID
lcl|56899
Description
None
Molecule type
nucleic acid
Query Length
201

Database Name
est
Description
Database of GenBank+EMBL+DDBJ sequences from EST Divisions
Program
BLASTN 2.2.22+ Citation

Reference
Stephen F. Altschul, Thomas L. Madden, Alejandro A. Schäffer, Jinghui Zhang, Zheng Zhang, Webb Miller, and David J. Lipman (1997), "Gapped BLAST and PSI-BLAST: a new generation of protein database search programs", Nucleic Acids Res. 25:3389-3402.
Other reports: Search Summary [Taxonomy reports] [Distance tree of results]

Search Parameters

Program	blastn
Word size	7
Expect value	10
Hitlist size	100
Match/Mismatch scores	2,-3
Gapcosts	5,2
Low Complexity Filter	Yes
Filter string	L;m;
Genetic Code	1

Database

Posted date	Oct 15, 2009 5:49 PM
Number of letters	34,724,607,482
Number of sequences	63,169,455
Entrez query	none

Karlin-Altschul statistics

Params	Ungapped	Gapped
Lambda	0.633731	0.625
K	0.408146	0.41
H	0.912438	0.78

Results Statistics

Length adjustment	34
Effective length of query	167
Effective length of database	36871813308
Effective search space	5440333284004
Effective search space used	5440333284004

Graphic Summary

Distribution of 99 Blast Hits on the Query Sequence

[?]

An overview of the database sequences aligned to the query sequence is shown. The score of each alignment is indicated by one of five different colors, which divides the range of scores into five groups. Multiple alignments on the same database sequence are connected by a striped line. Mousing over a hit sequence causes the definition and score to be shown in the window at the top, clicking on a hit sequence takes the user to the associated alignments. New: This graphic is an overview of database sequences aligned to the query sequence. Alignments are color-coded by score, within one of five score ranges. Multiple alignments on the same database sequence are connected by a dashed line. Mousing over an alignment shows the alignment definition and score in the box at the top. Clicking an alignment displays the alignment detail.

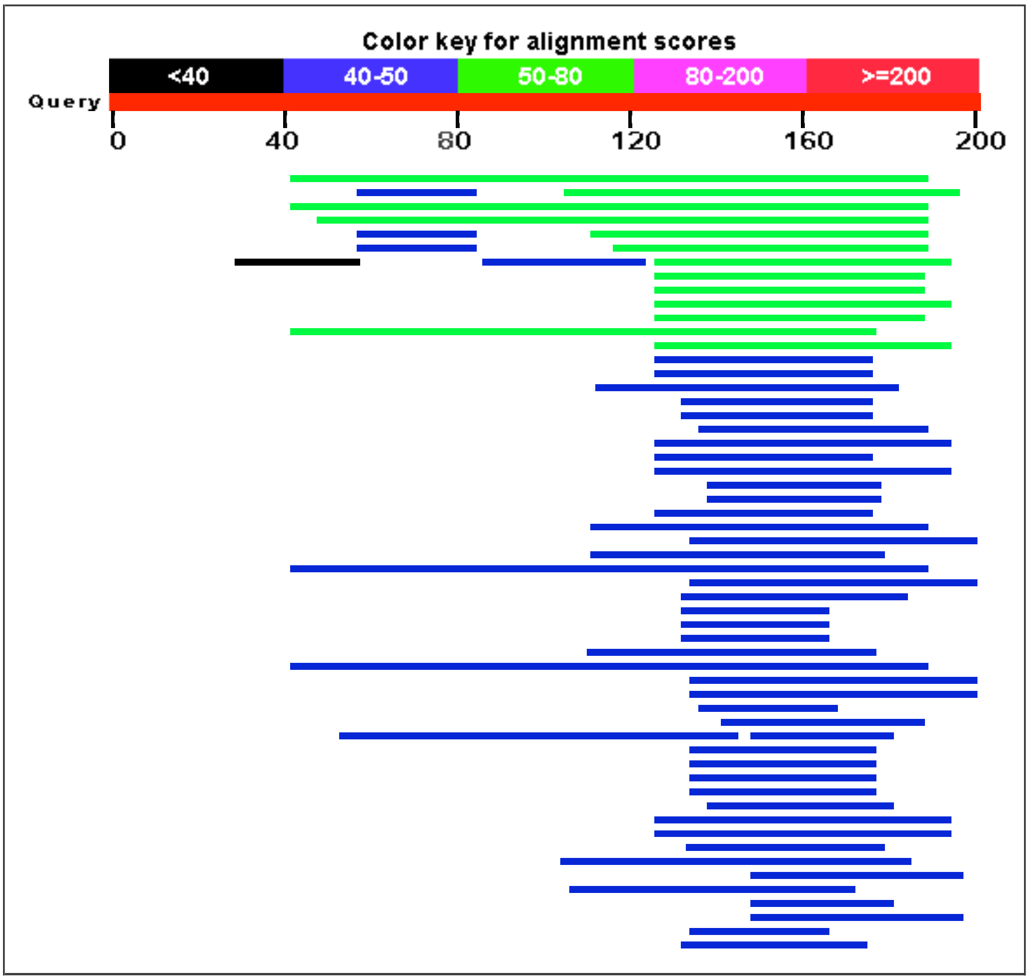

Descriptions

Legend for links to other resources: U UniGene E GEO G Gene S Structure M Map Viewer

Sequences producing significant alignments:
(Click headers to sort columns)

**EB293654.1**	CNSN01-F-043219-501 Normalized CNS library (juvenile 1) Aplysia californica cDNA clone CNSN01-F-043219 5', mRNA sequence	68.0	68.0	73%	2e-08	71%	U
**DC239387.1**	DC239387 Hodotermopsis sjoestedti whole body Hodotermopsis sjoestedti cDNA clone MY0816BHsBody_74 5', mRNA sequence	66.2	66.2	45%	6e-08	75%	
**EB306939.1**	CNSN01-F-067703-501 Normalized CNS library (juvenile 1) Aplysia californica cDNA clone CNSN01-F-067703 5', mRNA sequence	66.2	66.2	73%	6e-08	70%	U
**EB256885.1**	CNSN01-C-002780-501 Normalized CNS library (juvenile 1) Aplysia californica cDNA clone CNSN01-C-002780 5', mRNA sequence	64.4	64.4	70%	2e-07	71%	
**EB241937.1**	PEG003-C-218670-501 Normalized Pedal-Pleural Ganglia Aplysia californica cDNA clone PEG003-C-218670 5', mRNA sequence	60.8	60.8	38%	3e-06	76%	U
**GR632685.1**	cNOnD16-40_013_O22.ab1_c Tilapia juveniles 16-40 days post-fertilization library Oreochromis niloticus cDNA 5', mRNA sequence	59.0	59.0	36%	9e-06	79%	
**ES575416.1**	FPS013.C7.1_B22 LSG10-01 Lymnaea stagnalis cDNA clone FPS013_B22 3', mRNA sequence	59.0	59.0	33%	9e-06	80%	
**FC308956.1**	CAIC1777.fwd CAIC Nematostella vectensis Nemve whole embryos normalized Nematostella vectensis cDNA clone CAIC1777 5', mRNA sequence	57.2	57.2	30%	3e-05	82%	U
**FC308955.1**	CAIC1777.rev CAIC Nematostella vectensis Nemve whole embryos normalized Nematostella vectensis cDNA clone CAIC1777 3', mRNA sequence	57.2	57.2	30%	3e-05	82%	U
**EB294962.1**	CNSN01-F-045712-501 Normalized CNS library (juvenile 1) Aplysia californica cDNA clone CNSN01-F-045712 5', mRNA sequence	55.4	55.4	33%	1e-04	77%	U
**FC255983.1**	CAGN10221.fwd CAGN Nematostella vectensis Nemve mixed stages unfert eggs to primary polyps Nematostella vectensis cDNA clone CAGN10221 5', mRNA sequence	53.6	53.6	30%	4e-04	80%	U
**EB261739.1**	MCCN20-F-112769-501 Normalized MCC Neurons Aplysia californica cDNA clone MCCN20-F-112769 5', mRNA sequence	53.6	53.6	67%	4e-04	70%	U
**ES574907.1**	FPS012.C7_L21 LSG10-01 Lymnaea stagnalis cDNA clone FPS012_L21 3', mRNA sequence	51.8	51.8	33%	0.001	76%	
**EB270647.1**	MCC013-F-017597-501 Non-Normalized MCC Processes 2 Aplysia californica cDNA clone MCC013-F-017597 5', mRNA sequence	50.0	50.0	24%	0.005	82%	U
**EB191171.1**	PEG001-C-001551-501 Non-Normalized Pedal-Pleural Ganglia Aplysia californica cDNA clone PEG001-C-001651 5', mRNA sequence	50.0	50.0	24%	0.005	82%	U
**FF450528.1**	G178P60128RH7.T0 Acorn worm gastrula/neurula pCMVSport6 library Saccoglossus kowalevskii cDNA 5' end, mRNA sequence	48.2	48.2	34%	0.017	74%	
**EB336391.1**	CNSN01-F-143880-501 Normalized CNS library (juvenile 1) Aplysia californica cDNA clone CNSN01-F-143880 5', mRNA sequence	48.2	48.2	21%	0.017	84%	U
**EB305425.1**	CNSN01-F-065399-501 Normalized CNS library (juvenile 1) Aplysia californica cDNA clone CNSN01-F-065399 5', mRNA sequence	48.2	48.2	21%	0.017	84%	U
**FK741040.1**	av02108j08r1.1 Symbiotic sea anemone	46.4	46.4	26%	0.060	79%	

	(Anemonia viridis) cDNA library Anemonia viridis cDNA, mRNA sequence					
**GD231885.1**	G1045P366FM15.T1 Aplysia californica Pooled Normalized Library Aplysia californica cDNA, mRNA sequence	46.4	46.4	33%	0.060	75%
**GD227921.1**	G1045P383FB14.T1 Aplysia californica Pooled Normalized Library Aplysia californica cDNA, mRNA sequence	46.4	46.4	24%	0.060	80%
**FF065968.1**	G1045P312FO24.T0 Aplysia californica Pooled Normalized Library Aplysia californica cDNA, mRNA sequence	46.4	46.4	33%	0.060	75%
**EH634254.1**	EST5362 LK04 Laupala kohalensis cDNA clone 1061021796904 5', mRNA sequence	46.4	46.4	19%	0.060	85%
**EH631425.1**	EST2532 LK04 Laupala kohalensis cDNA clone 1061021471401 5', mRNA sequence	46.4	46.4	19%	0.060	85%
**EB334966.1**	CNSN01-F-141995-501 Normalized CNS library (juvenile 1) Aplysia californica cDNA clone CNSN01-F-141995 5', mRNA sequence	46.4	46.4	24%	0.060	80%
**EB196418.1**	PEG001-C-004164-301 Non-Normalized Pedal-Pleural Ganglia Aplysia californica cDNA clone PEG001-C-004164 3', mRNA sequence	46.4	46.4	38%	0.060	73%
**AJ884446.1**	AJ884446 Trichophyton rubrum CHUV862.00 Trichophyton rubrum cDNA clone TrMZE09ACQ, mRNA sequence	46.4	46.4	32%	0.060	78%
**GO599025.1**	VVSP01B09 spotted halibut SMART cDNA library from spleen Verasper variegatus cDNA 5', mRNA sequence	44.6	44.6	33%	0.21	75%
**FF060787.1**	G1045P39RG14.T0 Aplysia californica Pooled Normalized Library Aplysia californica cDNA, mRNA sequence	44.6	44.6	73%	0.21	68%
**FE027206.1**	C0009262F10.Q1KAM13R KN511 Ovis aries rectum and large colon from E. coli challenged and unchallenged animals Ovis aries cDNA clone C0009262F10 3', mRNA sequence	44.6	44.6	13%	0.21	96%
**AJ882815.1**	AJ882815 Trichophyton rubrum CHUV862.00 Trichophyton rubrum cDNA clone TrMZG04ABD, mRNA sequence	44.6	44.6	32%	0.21	76%
**EB350742.1**	CNSN01-F-145533-501 Normalized CNS library (juvenile 1) Aplysia californica cDNA clone CNSN01-F-145533 5', mRNA sequence	44.6	44.6	25%	0.21	78%
**EB316976.1**	CNSN01-F-083105-501 Normalized CNS library (juvenile 1) Aplysia californica cDNA clone CNSN01-F-083105 5', mRNA sequence	44.6	44.6	16%	0.21	88%
**EB301938.1**	CNSN01-F-060224-501 Normalized CNS library (juvenile 1) Aplysia californica cDNA clone CNSN01-F-060224 5', mRNA sequence	44.6	44.6	16%	0.21	88%
**EB254720.1**	CNSN01-C-006972-501 Normalized CNS library (juvenile 1) Aplysia californica cDNA clone CNSN01-C-006972 5', mRNA sequence	44.6	44.6	16%	0.21	88%
**EB244927.1**	PEG003-C-227362-501 Normalized Pedal-Pleural Ganglia Aplysia californica cDNA clone PEG003-C-227362 5', mRNA sequence	44.6	44.6	33%	0.21	74%
**EB227176.1**	PEG002-C-110975-501 Normalized Pedal-Pleural Ganglia Aplysia californica cDNA clone PEG002-C-110975 5', mRNA sequence	44.6	44.6	73%	0.21	66%
**DW707361.1**	EST030842 Trichophyton rubrum cDNA library 7 Trichophyton rubrum cDNA clone plasmid:FUNGI_9_119B_85, mRNA sequence	44.6	44.6	32%	0.21	76%
**DW702650.1**	EST026131 Trichophyton rubrum cDNA library 7 Trichophyton rubrum cDNA clone plasmid:FUNGI_9_059b_14, mRNA sequence	44.6	44.6	32%	0.21	76%
**CN200508.1**	Tor10181 Gametophyte rehydration Library Syntrichia ruralis cDNA, mRNA sequence	44.6	44.6	15%	0.21	90%

**CK876667.1**	SGP137954 Atlantic salmon Eye cDNA library Salmo salar cDNA clone OY4-0858 5', mRNA sequence	44.6	44.6	23%	0.21	80%	
**GD213660.1**	G1045P365RD9.T1 Aplysia californica Pooled Normalized Library Aplysia californica cDNA, mRNA sequence	42.8	42.8	45%	0.73	71%	
**FG561909.1**	BN18DYSC_UP_107_G12_31MAR2008_084 BN18DYSC Brassica napus cDNA 5', mRNA sequence	42.8	42.8	16%	0.73	87%	U
**EX838591.1**	CBNB7529.fwd CBNB Phycomyces blakesleeanus NRRL1555 Vegetative mycelium 48h old Dark L Phycomyces blakesleeanus cDNA clone CBNB7529 5', mRNA sequence	42.8	42.8	21%	0.73	83%	
**EX838590.1**	CBNB7529.rev CBNB Phycomyces blakesleeanus NRRL1555 Vegetative mycelium 48h old Dark L Phycomyces blakesleeanus cDNA clone CBNB7529 3', mRNA sequence	42.8	42.8	21%	0.73	83%	
**EX817665.1**	CBNA5689.fwd CBNA Phycomyces blakesleeanus NRRL1555 Vegetative mycelium 48h old Dark H Phycomyces blakesleeanus cDNA clone CBNA5689 5', mRNA sequence	42.8	42.8	21%	0.73	83%	
**EX817664.1**	CBNA5689.rev CBNA Phycomyces blakesleeanus NRRL1555 Vegetative mycelium 48h old Dark H Phycomyces blakesleeanus cDNA clone CBNA5689 3', mRNA sequence	42.8	42.8	21%	0.73	83%	
**AM847008.1**	AM847008 COL, cold overnight library Nicotiana tabacum cDNA clone nt006035064, mRNA sequence	42.8	42.8	21%	0.73	81%	
**EB238537.1**	PEG003-C-224292-501 Normalized Pedal-Pleural Ganglia Aplysia californica cDNA clone PEG003-C-224292 5', mRNA sequence	42.8	42.8	33%	0.73	73%	
**EB226379.1**	PEG002-C-108747-501 Normalized Pedal-Pleural Ganglia Aplysia californica cDNA clone PEG002-C-108747 5', mRNA sequence	42.8	42.8	33%	0.73	73%	U
**EG830862.1**	EST_ssal_eve_43068 ssaleve thyroid Salmo salar cDNA Salmo salar cDNA clone ssal_eve_558_218_rev 3', mRNA sequence	42.8	42.8	22%	0.73	80%	U
**BW777455.1**	BW777455 Amphioxus Branchiostoma floridae unpublished cDNA library, gastrula whole animal Branchiostoma floridae cDNA clone bfga045o16 5', mRNA sequence	42.8	42.8	40%	0.73	71%	
**CV297687.1**	EST886064 petunia floral development cDNA library Petunia x hybrida cDNA clone Petunia-DevA-18RR-F07 5' end, mRNA sequence	42.8	42.8	18%	0.73	84%	
**AJ672649.1**	AJ672649 KN224 Bos taurus cDNA clone KN224-009_N08, mRNA sequence	42.8	42.8	24%	0.73	80%	
**CD096407.1**	ME1-0008T-L087-D01-U.B ME1-0008 Schistosoma mansoni cDNA clone ME1-0008T-L087-D01.B, mRNA sequence	42.8	42.8	32%	0.73	74%	
**CD815515.1**	BN15.026J08F020214 BN15 Brassica napus cDNA clone BN15026J08, mRNA sequence	42.8	42.8	16%	0.73	87%	
**BI976185.1**	484936 MARC 2BOV Bos taurus cDNA 5', mRNA sequence	42.8	42.8	24%	0.73	80%	
**GO780561.1**	001020OFSA008082HT OFSA Ovis aries cDNA 5', mRNA sequence	41.0	41.0	13%	2.5	93%	U
**FL490534.1**	Mg_Nor01_49D21 Nor01 Mytilus galloprovincialis cDNA 3', mRNA sequence	41.0	41.0	15%	2.5	87%	
**BP999030.1**	BP999030 Perionyx excavatus regenerating tissue cDNA library Perionyx excavatus cDNA clone PER10677 3' similar to reverse transcriptase family member (1F383), mRNA sequence	41.0	41.0	21%	2.5	84%	
**ES406593.1**	MUT08-N18.x1d-t SHGC-MUT Mytilus californianus cDNA 5', mRNA sequence	41.0	41.0	22%	2.5	80%	
**ES401741.1**	MUT03-D23.y1d-s SHGC-MUT Mytilus	41.0	41.0	14%	2.5	90%	

	californianus cDNA 3', mRNA sequence						
**EH639442.1**	EST10550 LK04 Laupala kohalensis cDNA clone 1061021774416 5', mRNA sequence	41.0	41.0	17%	2.5	85%	
**EH634555.1**	EST5663 LK04 Laupala kohalensis cDNA clone 1061021821420 5', mRNA sequence	41.0	41.0	17%	2.5	85%	
**EB320170.1**	CNSN01-F-085761-501 Normalized CNS library (juvenile 1) Aplysia californica cDNA clone CNSN01-F-085761 5', mRNA sequence	41.0	41.0	34%	2.5	72%	U
**EB317104.1**	CNSN01-F-083310-501 Normalized CNS library (juvenile 1) Aplysia californica cDNA clone CNSN01-F-083310 5', mRNA sequence	41.0	41.0	34%	2.5	72%	U
**EB313998.1**	CNSN01-F-078486-501 Normalized CNS library (juvenile 1) Aplysia californica cDNA clone CNSN01-F-078486 5', mRNA sequence	41.0	41.0	34%	2.5	72%	U
**EB304909.1**	CNSN01-F-064655-501 Normalized CNS library (juvenile 1) Aplysia californica cDNA clone CNSN01-F-064655 5', mRNA sequence	41.0	41.0	24%	2.5	78%	U
**EB276974.1**	CNSN01-F-090235-501 Normalized CNS library (juvenile 1) Aplysia californica cDNA clone CNSN01-F-090235 5', mRNA sequence	41.0	41.0	20%	2.5	83%	
**EB245706.1**	PEG003-C-228360-501 Normalized Pedal-Pleural Ganglia Aplysia californica cDNA clone PEG003-C-228360 5', mRNA sequence	41.0	41.0	19%	2.5	82%	U
**EB236260.1**	PEG003-C-211457-501 Normalized Pedal-Pleural Ganglia Aplysia californica cDNA clone PEG003-C-211457 5', mRNA sequence	41.0	41.0	34%	2.5	72%	U
**DY020678.1**	53COT5_T3_008_H12_19JUL2004_082 Brassica napus 36hr germinating seed library 53COT5 Brassica napus cDNA 5', mRNA sequence	41.0	41.0	15%	2.5	87%	U
**EC021868.1**	3742948 KZ41 Caenorhabditis elegans cDNA clone 1414262, mRNA sequence	41.0	41.0	19%	2.5	85%	U
**EC003640.1**	7403423 CE04 Caenorhabditis elegans cDNA clone 2711032, mRNA sequence	41.0	41.0	19%	2.5	85%	U
**EB170901.1**	EST005800 injuried spinal cord cDNA library in Gecko Gekko japonicus cDNA clone SH_36_29, mRNA sequence	41.0	41.0	19%	2.5	82%	
**DY502648.1**	sh2P0044I13_F.ab1 adult sheep fracture callus 7d Ovis aries cDNA, mRNA sequence	41.0	41.0	13%	2.5	93%	U
**DW559163.1**	EST_ssal_rgb2_23582 rgb2 Salmo salar cDNA clone ssal_rgb2_538_115_rev 5', mRNA sequence	41.0	41.0	35%	2.5	72%	U
**AM048069.1**	AM048069 Schistosoma mansoni lung schistosomulum Schistosoma mansoni cDNA clone SmlC54g08.q1k, mRNA sequence	41.0	41.0	59%	2.5	68%	
**DN563352.1**	90876293 Sea Urchin primary mesenchyme cell cDNA library Strongylocentrotus purpuratus cDNA clone PMCSPR2-121A11 3', mRNA sequence	41.0	41.0	10%	2.5	100%	U
**CN633496.1**	taf11f01.y1 Hydra EST Darmstadt I Hydra magnipapillata cDNA 5', mRNA sequence	41.0	41.0	15%	2.5	90%	
**CF503562.1**	ML1-0002T-M131-C01-U.G ML1-0002 Schistosoma mansoni cDNA clone ML1-0002T-M131-C01.G similar to putative retrotransposon, mRNA sequence	41.0	41.0	28%	2.5	77%	
**AL789715.2**	AL789715 XGC-neurula Xenopus (Silurana) tropicalis cDNA clone TNeu133e22 5', mRNA sequence	41.0	41.0	16%	2.5	88%	
**CD066558.1**	MA1-0049T-R059-D06-U.G MA1-0049 Schistosoma mansoni cDNA clone MA1-0049T-R059-D06.G, mRNA sequence	41.0	41.0	33%	2.5	73%	
**CF445071.1**	EST681416 normalized cDNA library of onion Allium cepa cDNA clone ACAIH85, mRNA sequence	41.0	41.0	10%	2.5	100%	
**CB696383.1**	AMGNNUC:SRPB2-00223-H2-A srpb2 (10220) Rattus norvegicus cDNA clone srpb2-	41.0	41.0	25%	2.5	76%	

	00223-h2 5', mRNA sequence						
**BU432148.1**	603831116F1 CSEQRBN10 Gallus gallus cDNA clone ChEST821p3 5', mRNA sequence	41.0	41.0	19%	2.5	85%	
**CA330194.1**	hab02a12.y1 Fugu hgmpJ adult kidney Takifugu rubripes cDNA clone 6362398 5', mRNA sequence	41.0	41.0	19%	2.5	84%	
**AU140774.2**	AU140774 PLACE4 Homo sapiens cDNA clone PLACE4000206 5', mRNA sequence	41.0	41.0	13%	2.5	92%	U
**AL698619.1**	DKFZp686K22109_r1 686 (synonym: hlcc3) Homo sapiens cDNA clone DKFZp686K22109 5', mRNA sequence	41.0	41.0	13%	2.5	92%	U
**BG499451.1**	602546677F1 NIH_MGC_60 Homo sapiens cDNA clone IMAGE:4669146 5', mRNA sequence	41.0	41.0	28%	2.5	76%	
**AU132863.1**	AU132863 NT2RP4 Homo sapiens cDNA clone NT2RP4000740 5', mRNA sequence	41.0	41.0	13%	2.5	92%	U
**GR672193.1**	cN_n_V10_7_N07.ab1_c Tilapia adult ovary library Oreochromis niloticus cDNA 5', mRNA sequence	39.2	78.3	12%	8.9	92%	
**GO645697.1**	EST_afim_evh_1056146 afimevh mixed_tissue Anoplopoma fimbria cDNA Anoplopoma fimbria cDNA clone afim_evh_530_050 3', mRNA sequence	39.2	39.2	11%	8.9	95%	
**GO645696.1**	EST_afim_evh_1054994 afimevh mixed_tissue Anoplopoma fimbria cDNA Anoplopoma fimbria cDNA clone afim_evh_530_050 5', mRNA sequence	39.2	39.2	11%	8.9	95%	
**GD065748.1**	KS15025G02 KS15 Capsicum annuum cDNA, mRNA sequence	39.2	39.2	19%	8.9	82%	
**FM973896.1**	FM973896 T.brucei infected salivary gland Glossina morsitans morsitans cDNA clone GMsg74h11.q1k, mRNA sequence	39.2	39.2	16%	8.9	87%	
**FG294822.1**	1108770719004 New World Screwworm Larvae 9387 ESTs Cochliomyia hominivorax cDNA, mRNA sequence	39.2	39.2	14%	8.9	89%	
**GE578522.1**	CCPU2630.b1_L10.ab1 CCP(UWX) Globe Artichoke Cynara scolymus cDNA clone CCPU2630, mRNA sequence	39.2	39.2	22%	8.9	80%	

NOP
ERROR.ERROR.ERROR.ERROR

Alignments Select All Get selected sequences Distance tree of results Multiple alignment

```
>gb|EB293654.1| CNSN01-F-043219-501 Normalized CNS library (juvenile 1) Aplysia
californica cDNA clone CNSN01-F-043219 5', mRNA sequence.
Length=244

 Score = 68.0 bits (74),  Expect = 2e-08
 Identities = 107/150 (71%), Gaps = 4/150 (2%)
 Strand=Plus/Minus

Query  43   ACAACTATAATAAGA--AGACGCTGGAGGTGGATTCGGCATGTCCTACGGAAAG-GCCAG  99
            ||||| ||||||| |  ||| | ||||| |||||| | ||||| ||  | | || |||||
Sbjct  182  ACAACCATAATAAAAGGAGAAGGTGGAGATGGATTGGACATGTTCTGAGAAGAGAGCCAG  123

Query  100  GGGGATATCACAAAAACAGCACTCCACTGGACCCCAGAAGGTAAAAGAAAGAGAGGAAGA  159
                 |||    || || || ||||||||||| || |||||     | ||||| || |||
Sbjct  122  ACTC-TATTGTGAAGACTGCCCTCCACTGGACACCCGAAGGACGTCGCAAGAGGGGCAGA  64

Query  160  CCTAAAATGACATGGAGAAGAACTGTAGAG  189
            || ||| |||| |||||| | || ||||||
Sbjct  63   CCCAAAGTGACCTGGAGACGCACAGTAGAG  34
```

```
>dbj|DC239387.1| DC239387 Hodotermopsis sjoestedti whole body Hodotermopsis sjoestedti
cDNA clone MY0816BHsBody_74 5', mRNA sequence.
Length=757

 Score = 66.2 bits (72),  Expect = 6e-08
 Identities = 69/91 (75%), Gaps = 0/91 (0%)
 Strand=Plus/Minus

Query  106  ATCACAAAAACAGCACTCCACTGGACCCCAGAAGGTAAAAGAAAGAGAGGAAGACCTAAA  165
            |||| |||   ||||||| | |||| |||| | ||| |  |||||  ||||||||| |||
Sbjct  439  ATCAAAAAGCAAGCACTCTATTGGAACCCACAGGGTCAGCGAAAGCAAGGAAGACCAAAA  380

Query  166  ATGACATGGAGAAGAACTGTAGAGGCAGAGG  196
            ||||  ||| | |||| ||| |||| |||||
Sbjct  379  ATGAATTGGCGGAGAAGTGTGGAGGAAGAGG  349
```

```
>gb|EB306939.1| CNSN01-F-067703-501 Normalized CNS library (juvenile 1) Aplysia
californica cDNA clone CNSN01-F-067703 5', mRNA sequence.
Length=651

 Score = 66.2 bits (72),  Expect = 6e-08
 Identities = 107/151 (70%), Gaps = 5/151 (3%)
 Strand=Plus/Minus

Query  43   ACAACTATAATAAGAAG---ACGCTGGAGGTGGATTCGGCATGTCCTACGGAAAG-GCCA  98
            ||||| ||||||| |||   | | ||||| |||||| | ||||| ||  | | || ||||
Sbjct  372  ACAACCATAATAAAAAGGAGAAGGTGGAGATGGATTGGACATGTTCTGAGAAGAGAGCCA  313

Query  99   GGGGGATATCACAAAAACAGCACTCCACTGGACCCCAGAAGGTAAAAGAAAGAGAGGAAG  158
            |     |||    || || || ||||||||||| || |||||     | ||||| || ||
Sbjct  312  GACTC-TATTGTGAAGACTGCCCTCCACTGGACACCCGAAGGACGTCGCAAGAGGGGCAG  254

Query  159  ACCTAAAATGACATGGAGAAGAACTGTAGAG  189
            ||| ||| |||| |||||| | || ||||||
Sbjct  253  ACCCAAAGTGACCTGGAGACGCACAGTAGAG  223
```

```
>gb|EB256885.1| CNSN01-C-002780-501 Normalized CNS library (juvenile 1) Aplysia
californica cDNA clone CNSN01-C-002780 5', mRNA sequence.
Length=739

 Score = 64.4 bits (70),  Expect = 2e-07
 Identities = 101/142 (71%), Gaps = 2/142 (1%)
 Strand=Plus/Minus

Query  49   ATAATAAGAAGACGCTGGAGGTGGATTCGGCATGTCCTACGGAAAGG-CCAGGGGGATAT  107
            |||| ||| ||| | |||||||||||| ||||||| |  || | ||  ||||    | ||
Sbjct  599  ATAACAAGGAGAAGATGGAGGTGGATTGGGCATGTTCAGCGAAGAGAACCAGACTCA-AT  541

Query  108  CACAAAAACAGCACTCCACTGGACCCCAGAAGGTAAAAGAAAGAGAGGAAGACCTAAAAT  167
               ||| || |||||||||| ||| || |||||  |  | ||||| || ||||| ||  |
Sbjct  540  TGTAAAGACTGCACTCCACTAGACACCCGAAGGACATCGCAAGAGGGGCAGACCCAAGGT  481

Query  168  GACATGGAGAAGAACTGTAGAG  189
            ||| |||| |   | |||||||
Sbjct  480  GACCTGGATACACATTGTAGAG  459
```

>**gb|EB241937.1|** PEG003-C-218670-501 Normalized Pedal-Pleural Ganglia Aplysia californica cDNA clone PEG003-C-218670 5', mRNA sequence.
Length=710

```
 Score = 60.8 bits (66),  Expect = 3e-06
 Identities = 60/78 (76%), Gaps = 0/78 (0%)
 Strand=Plus/Minus

Query  112  AAAACAGCACTCCACTGGACCCCAGAAGGTAAAAGAAAGAGAGGAAGACCTAAAATGACA  171
            || || || ||||||||||| || |||||  |  | ||||| || ||||| ||| |||| 
Sbjct  154  AAGACTGCCCTCCACTGGACACCCGAAGGACATCGCAAGAGGGGCAGACCCAAAGTGACC  95

Query  172  TGGAGAAGAACTGTAGAG  189
            |||||| | || ||||||
Sbjct  94   TGGAGACGCACAGTAGAG  77
```

>**gb|GR632685.1|** cNOnD16-40_013_O22.ab1_c Tilapia juveniles 16-40 days post-fertilization library Oreochromis niloticus cDNA 5', mRNA sequence.
Length=543

```
 Score = 59.0 bits (64),  Expect = 9e-06
 Identities = 59/74 (79%), Gaps = 2/74 (2%)
 Strand=Plus/Minus

Query  117  AGCACTCCACTGGACCCCAGAAGGTAAAAG-AAAGAGAGGAAGACCTAAAATGACATGGA  175
            ||||||  |||||| |||| |||| ||||| |||| | ||| |||| |||  ||| ||||
Sbjct  260  AGCACTGGACTGGAACCCACAAGGAAAAAGGAAAGTGGGGA-GACCCAAACAGACGTGGA  202

Query  176  GAAGAACTGTAGAG  189
            ||||| |||| |||
Sbjct  201  GAAGATCTGTCGAG  188
```

>**gb|ES575416.1|** FPS013.C7.1_B22 LSG10-01 Lymnaea stagnalis cDNA clone FPS013_B22 3', mRNA sequence.
Length=892

```
 Score = 59.0 bits (64),  Expect = 9e-06
 Identities = 56/70 (80%), Gaps = 4/70 (5%)
 Strand=Plus/Minus

Query  127  TGGACCCCAGAAGGTAAAAGAAAGAGAGGAAGACCTAAAATGACATGGAGAA--GAACTG  184
            ||||  ||| | || ||||||||||||||||| ||||| |  |||||||||   ||||| 
Sbjct  417  TGGAATCCACAGGGAAAAAGAAAGAGAGGAAGGCCTAAGAAAACATGGAGACTCGAACT-  359

Query  185  TAGAGGCAGA  194
             |||||||||
Sbjct  358  -AGAGGCAGA  350
```

>**gb|FC308956.1|** CAIC1777.fwd CAIC Nematostella vectensis Nemve whole embryos normalized Nematostella vectensis cDNA clone CAIC1777 5', mRNA sequence.
Length=626

```
 Score = 57.2 bits (62),  Expect = 3e-05
 Identities = 52/63 (82%), Gaps = 2/63 (3%)
 Strand=Plus/Minus

Query  127  TGGACCCCAGAAGGTAAAAG-AAAGAGAGGAAGACCTAAAATGACATGGAGAAGAACTGT  185
            ||||| |||||||| | ||| ||| || |||||||| ||||| |||||| |||| |||||
Sbjct  157  TGGACACCAGAAGGCAGAAGGAAACAG-GGAAGACCCAAAATAACATGGCGAAGGACTGT  99

Query  186  AGA  188
             ||
Sbjct  98   GGA  96
```

>**gb|FC308955.1|** CAIC1777.rev CAIC Nematostella vectensis Nemve whole embryos normalized Nematostella vectensis cDNA clone CAIC1777 3', mRNA sequence.
Length=613

```
 Score = 57.2 bits (62),  Expect = 3e-05
 Identities = 52/63 (82%), Gaps = 2/63 (3%)
 Strand=Plus/Plus

Query  127  TGGACCCCAGAAGGTA-AAAGAAAGAGAGGAAGACCTAAAATGACATGGAGAAGAACTGT  185
            ||||| |||||||| | || |||| || |||||||| ||||| |||||| |||| |||||
Sbjct  549  TGGACACCAGAAGGCAGAAGGAAACAG-GGAAGACCCAAAATAACATGGCGAAGGACTGT  607

Query  186  AGA  188
             ||
Sbjct  608  GGA  610
```

>**gb|EB294962.1|** CNSN01-F-045712-501 Normalized CNS library (juvenile 1) Aplysia californica cDNA clone CNSN01-F-045712 5', mRNA sequence.
Length=441

```
 Score = 55.4 bits (60),  Expect = 1e-04
 Identities = 53/68 (77%), Gaps = 0/68 (0%)
 Strand=Plus/Plus

Query  127  TGGACCCCAGAAGGTAAAAGAAAGAGAGGAAGACCTAAAATGACATGGAGAAGAACTGTA  186
```

```
             |||| |||| |||| ||| ||||||  |||||||| || |||||||||||  || | |||
Sbjct  164   TGGAACCCACAAGGAAAACGAAAGAAGGGAAGACCAAAGATGACATGGAGGCGAGCGGTA  223

Query  187   GAGGCAGA  194
              | || ||
Sbjct  224   CAAGCTGA  231
```

>**gb|FC255983.1|** CAGN10221.fwd CAGN Nematostella vectensis Nemve mixed stages unfert eggs to primary polyps Nematostella vectensis cDNA clone CAGN10221 5', mRNA sequence.
Length=870

```
 Score = 53.6 bits (58),  Expect = 4e-04
 Identities = 51/63 (80%), Gaps = 2/63 (3%)
 Strand=Plus/Plus

Query  127   TGGACCCCAGAAGGTA-AAAGAAAGAGAGGAAGACCTAAAATGACATGGAGAAGAACTGT  185
             ||||| |||||||| | || |||| || |||||||| ||||  |||||| |||| |||||
Sbjct  509   TGGACACCAGAAGGCAGAAGGAAACAG-GGAAGACCCAAAACAACATGGCGAAGGACTGT  567

Query  186   AGA  188
              ||
Sbjct  568   GGA  570
```

>**gb|EB261739.1|** MCCN20-F-112769-501 Normalized MCC Neurons Aplysia californica cDNA clone MCCN20-F-112769 5', mRNA sequence.
Length=356

```
 Score = 53.6 bits (58),  Expect = 4e-04
 Identities = 98/139 (70%), Gaps = 6/139 (4%)
 Strand=Plus/Minus

Query  43    ACAACTATAATAAGAAG---ACGCTGGAGGTGGATTCGGCATGTCCTACGGAAAG-GCCA  98
             ||||| ||||||| |||    | | ||||| |||||| | ||||| ||  | | || ||||
Sbjct  139   ACAACCATAATAAAAAGGAGAAGGTGGAGATGGATTGGACATGTTCTGAGAAGAGAGCCA  80

Query  99    GGGGGATATCACAAAAACAGCACTCCACTGGACCCCAGAAGGTAAAAGAAAGAGAGGAAG  158
             |     |||    || || || ||||||||||| || |||||     | ||||| || ||
Sbjct  79    GACTC-TATTGTGAAGACTGCCCTCCACTGGACACCCGAAGGACGTCGCAAGAG-GGCAG  22

Query  159   ACCTAAAATGACATGGAGA  177
             ||| ||| |||| ||||||
Sbjct  21    ACCCAAAGTGACCTGGAGA  3
```

>**gb|ES574907.1|** FPS012.C7_L21 LSG10-01 Lymnaea stagnalis cDNA clone FPS012_L21 3', mRNA sequence.
Length=900

```
 Score = 51.8 bits (56),  Expect = 0.001
 Identities = 52/68 (76%), Gaps = 0/68 (0%)
 Strand=Plus/Minus

Query  127   TGGACCCCAGAAGGTAAAAGAAAGAGAGGAAGACCTAAAATGACATGGAGAAGAACTGTA  186
             |||| ||||  ||| ||||||||||||||||| ||||  |  ||| ||||| |     ||
Sbjct  112   TGGAACCCACGAGGAAAAAGAAAGAGAGGAAGGCCTAGGAATACACGGAGACGCGAGCTA  53

Query  187   GAGGCAGA  194
             ||||||||
Sbjct  52    GAGGCAGA  45
```

>**gb|EB270647.1|** MCC013-F-017597-501 Non-Normalized MCC Processes 2 Aplysia californica cDNA clone MCC013-F-017597 5', mRNA sequence.
Length=617

```
 Score = 50.0 bits (54),  Expect = 0.005
 Identities = 41/50 (82%), Gaps = 0/50 (0%)
 Strand=Plus/Plus

Query  127   TGGACCCCAGAAGGTAAAAGAAAGAGAGGAAGACCTAAAATGACATGGAG  176
             ||||  ||| |||| ||| | |||||||||||||  || |||||||||||
Sbjct  34    TGGAATCCACAAGGAAAATGTAAGAGAGGAAGACAAAAGATGACATGGAG  83
```

>**gb|EB191171.1|** PEG001-C-001551-501 Non-Normalized Pedal-Pleural Ganglia Aplysia californica cDNA clone PEG001-C-001651 5', mRNA sequence.
Length=453

```
 Score = 50.0 bits (54),  Expect = 0.005
 Identities = 41/50 (82%), Gaps = 0/50 (0%)
 Strand=Plus/Plus

Query  127   TGGACCCCAGAAGGTAAAAGAAAGAGAGGAAGACCTAAAATGACATGGAG  176
             ||||  |||||||| ||| | |||| ||||||||| || ||| |||||||
Sbjct  160   TGGATTCCAGAAGGAAAACGTAAGAAAGGAAGACCAAAGATGGCATGGAG  209
```

>**gb|FF450528.1|** G178P60128RH7.T0 Acorn worm gastrula/neurula pCMVSport6 library Saccoglossus kowalevskii cDNA 5' end, mRNA sequence.
Length=606

```
 Score = 48.2 bits (52),  Expect = 0.017
 Identities = 55/74 (74%), Gaps = 4/74 (5%)
 Strand=Plus/Minus
```

```
Query  113   AAACAGCACTCCACTGGACCCCAGAAGGTAAAAGAA----AGAGAGGAAGACCTAAAATG  168
             |||||||||| | |||||| ||| | ||| || |||    |||||||  | |  |||
Sbjct  393   AAACAGCACTTCGCTGGACGCCACAGGGTCAATGAAACAGAGAGAGGGGGGCAAAAACCC  334

Query  169   ACATGGAGAAGAAC  182
             ||||||||||||||
Sbjct  333   ACATGGAGAAGAAC  320
```

>**gb|EB336391.1|** CNSN01-F-143880-501 Normalized CNS library (juvenile 1) Aplysia californica cDNA clone CNSN01-F-143880 5', mRNA sequence.
Length=528

```
 Score = 48.2 bits (52),  Expect = 0.017
 Identities = 37/44 (84%), Gaps = 0/44 (0%)
 Strand=Plus/Plus

Query  133   CCAGAAGGTAAAAGAAAGAGAGGAAGACCTAAAATGACATGGAG  176
             |||||||| ||| | |||| ||||||||| || ||| |||||||
Sbjct  334   CCAGAAGGAAAACGTAAGAAAGGAAGACCAAAGATGGCATGGAG  377
```

>**gb|EB305425.1|** CNSN01-F-065399-501 Normalized CNS library (juvenile 1) Aplysia californica cDNA clone CNSN01-F-065399 5', mRNA sequence.
Length=514

```
 Score = 48.2 bits (52),  Expect = 0.017
 Identities = 37/44 (84%), Gaps = 0/44 (0%)
 Strand=Plus/Plus

Query  133   CCAGAAGGTAAAAGAAAGAGAGGAAGACCTAAAATGACATGGAG  176
             |||||||| ||| | |||| ||||||||| || ||| |||||||
Sbjct  337   CCAGAAGGAAAACGTAAGAAAGGAAGACCAAAGATGGCATGGAG  380
```

>**gb|FK741040.1|** av02108j08r1.1 Symbiotic sea anemone (Anemonia viridis) cDNA library Anemonia viridis cDNA, mRNA sequence.
Length=688

```
 Score = 46.4 bits (50),  Expect = 0.060
 Identities = 42/53 (79%), Gaps = 0/53 (0%)
 Strand=Plus/Minus

Query  137   AAGGTAAAAGAAAGAGAGGAAGACCTAAAATGACATGGAGAAGAACTGTAGAG  189
             |||| |||| ||  | ||||||||  ||||  ||||||||| | |||||||||
Sbjct  478   AAGGAAAAAAAAGAAAAGGAAGACTGAAAACAACATGGAGATGGACTGTAGAG  426
```

>**gb|GD231885.1|** G1045P366FM15.T1 Aplysia californica Pooled Normalized Library Aplysia californica cDNA, mRNA sequence.
Length=785

```
 Score = 46.4 bits (50),  Expect = 0.060
 Identities = 51/68 (75%), Gaps = 0/68 (0%)
 Strand=Plus/Minus

Query  127   TGGACCCCAGAAGGTAAAAGAAAGAGAGGAAGACCTAAAATGACATGGAGAAGAACTGTA  186
             |||| |||| |||| |||   ||||  |||||||| || |||||||||||  |  |||||
Sbjct  128   TGGAACCCACAAGGAAAACTTAAGAAGGGAAGACCAAAGATGACATGGAGGCGGGCTGTA  69

Query  187   GAGGCAGA  194
              | || ||
Sbjct  68    CAAGCGGA  61
```

>**gb|GD227921.1|** G1045P383FB14.T1 Aplysia californica Pooled Normalized Library Aplysia californica cDNA, mRNA sequence.
Length=790

```
 Score = 46.4 bits (50),  Expect = 0.060
 Identities = 40/50 (80%), Gaps = 0/50 (0%)
 Strand=Plus/Plus

Query  127   TGGACCCCAGAAGGTAAAAGAAAGAGAGGAAGACCTAAAATGACATGGAG  176
             ||||  ||| |||| ||| | |||| |||||| || || |||||||||||
Sbjct  144   TGGAATCCACAAGGAAAACGTAAGAAAGGAAGGCCAAAGATGACATGGAG  193
```

>**gb|FF065968.1|** G1045P312FO24.T0 Aplysia californica Pooled Normalized Library Aplysia californica cDNA, mRNA sequence.
Length=765

```
 Score = 46.4 bits (50),  Expect = 0.060
 Identities = 51/68 (75%), Gaps = 0/68 (0%)
 Strand=Plus/Minus

Query  127   TGGACCCCAGAAGGTAAAAGAAAGAGAGGAAGACCTAAAATGACATGGAGAAGAACTGTA  186
             |||| |||| |||| |||   ||||  |||||||| || |||||||||||  |  |||||
Sbjct  122   TGGAACCCACAAGGAAAACTTAAGAAGGGAAGACCAAAGATGACATGGAGGCGGGCTGTA  63

Query  187   GAGGCAGA  194
              | || ||
Sbjct  62    CAAGCGGA  55
```

>**gb|EH634254.1|** EST5362 LK04 Laupala kohalensis cDNA clone 1061021796904 5',

```
mRNA sequence.
Length=795

 Score = 46.4 bits (50),  Expect = 0.060
 Identities = 34/40 (85%), Gaps = 0/40 (0%)
 Strand=Plus/Minus

Query  139  GGTAAAAGAAAGAGAGGAAGACCTAAAATGACATGGAGAA  178
            |||| | | ||||||||||| || ||||||| ||||||||
Sbjct  349  GGTAGACGGAAGAGAGGAAGGCCGAAAATGAAATGGAGAA  310
```

>gb|EH631425.1| EST2532 LK04 Laupala kohalensis cDNA clone 1061021471401 5', mRNA sequence.

```
Length=795

 Score = 46.4 bits (50),  Expect = 0.060
 Identities = 34/40 (85%), Gaps = 0/40 (0%)
 Strand=Plus/Minus

Query  139  GGTAAAAGAAAGAGAGGAAGACCTAAAATGACATGGAGAA  178
            |||| | | ||||||||||| || ||||||| ||||||||
Sbjct  349  GGTAGACGGAAGAGAGGAAGGCCGAAAATGAAATGGAGAA  310
```

>gb|EB334966.1| CNSN01-F-141995-501 Normalized CNS library (juvenile 1) Aplysia californica cDNA clone CNSN01-F-141995 5', mRNA sequence.

```
Length=650

 Score = 46.4 bits (50),  Expect = 0.060
 Identities = 40/50 (80%), Gaps = 0/50 (0%)
 Strand=Plus/Plus

Query  127  TGGACCCCAGAAGGTAAAAGAAAGAGAGGAAGACCTAAAATGACATGGAG  176
            |||| |||| |||| |||   ||||  |||||||| || |||||||||||
Sbjct  197  TGGAACCCACAAGGAAAACATAAGAAGGGAAGACCAAAGATGACATGGAG  246
```

>gb|EB196418.1| PEG001-C-004164-301 Non-Normalized Pedal-Pleural Ganglia Aplysia californica cDNA clone PEG001-C-004164 3', mRNA sequence.

```
Length=686

 Score = 46.4 bits (50),  Expect = 0.060
 Identities = 57/78 (73%), Gaps = 0/78 (0%)
 Strand=Plus/Plus

Query  112  AAAACAGCACTCCACTGGACCCCAGAAGGTAAAAGAAAGAGAGGAAGACCTAAAATGACA  171
            || || || ||||||||||| || |||||      | || || || ||||| ||| || |
Sbjct  146  AAGACTGCCCTCCACTGGACACCCGAAGGACGTCGCAAAAGGGGCAGACCCAAAGTGGCC  205

Query  172  TGGAGAAGAACTGTAGAG  189
            |||||| | || ||||||
Sbjct  206  TGGAGACGCACAGTAGAG  223
```

>emb|AJ884446.1| AJ884446 Trichophyton rubrum CHUV862.00 Trichophyton rubrum cDNA clone TrMZE09ACQ, mRNA sequence.

```
Length=465

 Score = 46.4 bits (50),  Expect = 0.060
 Identities = 54/69 (78%), Gaps = 7/69 (10%)
 Strand=Plus/Plus

Query  135  AGAAGGTAAAAGAAAGAGAGGAAGACCTAAAATGAC---ATGGAGAAGAACTGTAGAGGC  191
            ||||||| || ||| |||||||||||| |||| |||    | |||||||||  | ||| |
Sbjct  279  AGAAGGTGAA-GAAGGAGAGGAAGACC-AAAAGGACAAGAAGGAGAAGAA--GGAGAAGA  334

Query  192  AGAGGCTAG  200
            ||| |||||
Sbjct  335  AGAAGCTAG  343
```

>gb|GO599025.1| VVSP01B09 spotted halibut SMART cDNA library from spleen Verasper variegatus cDNA 5', mRNA sequence.

```
Length=832

 Score = 44.6 bits (48),  Expect = 0.21
 Identities = 52/69 (75%), Gaps = 1/69 (1%)
 Strand=Plus/Minus

Query  112  AAAACAG-CACTCCACTGGACCCCAGAAGGTAAAAGAAAGAGAGGAAGACCTAAAATGAC  170
            ||||||| ||||     |||| |||| |||| |||  |   | |||||| |||| ||||  ||
Sbjct  347  AAAACAGGCACTGACATGGAACCCACAAGGCAAACGGGATAGAGGATGACCCAAAAACAC  288

Query  171  ATGGAGAAG  179
             ||||||||
Sbjct  287  CTGGAGAAG  279
```

>gb|FF060787.1| G1045P39RG14.T0 Aplysia californica Pooled Normalized Library Aplysia californica cDNA, mRNA sequence.

```
Length=808

 Score = 44.6 bits (48),  Expect = 0.21
 Identities = 103/151 (68%), Gaps = 7/151 (4%)
 Strand=Plus/Plus

Query  43   ACAACTATAATAAGAAGACGCTGGAGGTGGATTCGGCATGTCCTACGGAAAGGCCAGGGG  102
```

```
                ||||| ||||||| |||| | |||||  ||||| | ||||| ||   | | ||   |   |
Sbjct  514   ACAACCATAATAAAAAGAAGGTGGAGA-GGATTGGACATGTTCTGAGAAGAGAACCAGAC  572

Query  103   GATATCACAAAAACAGCACTCCACTGG-ACCCCAGAAGGTAAAAGA---AAGAGAGGAAG  158
               |||     || || || || ||||||| || || ||||  ||   |     || || || ||
Sbjct  573   TCTATTGTGAAGACTGCCCTACACTGGGACACCCGAAG--AACGGTCGCAAAAGGGGCAG  630

Query  159   ACCTAAAATGACATGGAGAAGAACTGTAGAG  189
             ||| |||||||| |||||| | || ||||||
Sbjct  631   ACCCAAAATGACCTGGAGACGCACAGTAGAG  661
```

>**gb|FE027206.1|** C0009262F10.Q1KAM13R KN511 Ovis aries rectum and large colon from E. coli challenged and unchallenged animals Ovis aries cDNA clone C0009262F10 3', mRNA sequence.
Length=674

```
 Score = 44.6 bits (48),  Expect = 0.21
 Identities = 28/29 (96%), Gaps = 1/29 (3%)
 Strand=Plus/Minus

Query  58    AGACGCTGGAGGTGGAT-TCGGCATGTCC  85
             |||||||||||||||| |||||||||||
Sbjct  336   AGACGCTGGAGGTGGATGTCGGCATGTCC  308
```

>**emb|AJ882815.1|** AJ882815 Trichophyton rubrum CHUV862.00 Trichophyton rubrum cDNA clone TrMZG04ABD, mRNA sequence.
Length=907

```
 Score = 44.6 bits (48),  Expect = 0.21
 Identities = 53/69 (76%), Gaps = 6/69 (8%)
 Strand=Plus/Plus

Query  135   AGAAGGTAAAAGAAAGAGAGGAAGACCTAAAATGAC---ATGGAGAAGAACTGTAGAGGC  191
             ||||||| || ||| |||||||||||| | || |||    | |||||||||  | ||| |
Sbjct  292   AGAAGGTGAA-GAAGGAGAGGAAGACCAAGAAGGACAAGAAGGAGAAGAA--GGAGAAGA  348

Query  192   AGAGGCTAG  200
             ||| |||||
Sbjct  349   AGAAGCTAG  357
```

>**gb|EB350742.1|** CNSN01-F-145533-501 Normalized CNS library (juvenile 1) Aplysia californica cDNA clone CNSN01-F-145533 5', mRNA sequence.
Length=230

```
 Score = 44.6 bits (48),  Expect = 0.21
 Identities = 41/52 (78%), Gaps = 0/52 (0%)
 Strand=Plus/Plus

Query  133   CCAGAAGGTAAAAGAAAGAGAGGAAGACCTAAAATGACATGGAGAAGAACTG  184
             ||||| || ||| | |||| ||||||||| || ||| |||||||   || |||
Sbjct  130   CCAGATGGAAAACGTAAGAAAGGAAGACCAAAGATGGCATGGAGGCGAGCTG  181
```

>**gb|EB316976.1|** CNSN01-F-083105-501 Normalized CNS library (juvenile 1) Aplysia californica cDNA clone CNSN01-F-083105 5', mRNA sequence.
Length=459

```
 Score = 44.6 bits (48),  Expect = 0.21
 Identities = 30/34 (88%), Gaps = 0/34 (0%)
 Strand=Plus/Minus

Query  133   CCAGAAGGTAAAAGAAAGAGAGGAAGACCTAAAA  166
             ||| ||||||| ||||||| ||||||||||| ||
Sbjct  95    CCACAAGGTAAGAGAAAGAAAGGAAGACCTATAA  62
```

>**gb|EB301938.1|** CNSN01-F-060224-501 Normalized CNS library (juvenile 1) Aplysia californica cDNA clone CNSN01-F-060224 5', mRNA sequence.
Length=118

```
 Score = 44.6 bits (48),  Expect = 0.21
 Identities = 30/34 (88%), Gaps = 0/34 (0%)
 Strand=Plus/Minus

Query  133   CCAGAAGGTAAAAGAAAGAGAGGAAGACCTAAAA  166
             ||| ||||||| ||||||| ||||||||||| ||
Sbjct  97    CCACAAGGTAAGAGAAAGAAAGGAAGACCTATAA  64
```

>**gb|EB254720.1|** CNSN01-C-006972-501 Normalized CNS library (juvenile 1) Aplysia californica cDNA clone CNSN01-C-006972 5', mRNA sequence.
Length=729

```
 Score = 44.6 bits (48),  Expect = 0.21
 Identities = 30/34 (88%), Gaps = 0/34 (0%)
 Strand=Plus/Minus

Query  133   CCAGAAGGTAAAAGAAAGAGAGGAAGACCTAAAA  166
             ||| ||||||| ||||||| ||||||||||| ||
Sbjct  476   CCACAAGGTAAGAGAAAGAAAGGAAGACCTATAA  443
```

>**gb|EB244927.1|** PEG003-C-227362-501 Normalized Pedal-Pleural Ganglia Aplysia californica cDNA clone PEG003-C-227362 5', mRNA sequence.

Length=920

```
 Score = 44.6 bits (48),  Expect = 0.21
 Identities = 50/67 (74%), Gaps = 0/67 (0%)
 Strand=Plus/Plus

Query  111  AAAAACAGCACTCCACTGGACCCCAGAAGGTAAAAGAAAGAGAGGAAGACCTAAAATGAC  170
            ||| || |||||||||||||  ||  ||||  |   |||||| || ||||| ||| ||||
Sbjct  202  AAAGACTGCACTCCACTGGATACCCAAAGGACATCAAAAGAGGGGCAGACCCAAAGTGAC  261

Query  171  ATGGAGA  177
              |||||
Sbjct  262  CCGGAGA  268
```

>**gb|EB227176.1|** PEG002-C-110975-501 Normalized Pedal-Pleural Ganglia Aplysia californica cDNA clone PEG002-C-110975 5', mRNA sequence.
Length=653

```
 Score = 44.6 bits (48),  Expect = 0.21
 Identities = 100/150 (66%), Gaps = 3/150 (2%)
 Strand=Plus/Plus

Query  43   ACAACTATAATAAGAAG---ACGCTGGAGGTGGATTCGGCATGTCCTACGGAAAGGCCAG  99
            ||||| ||||||| |||   | | ||||| |||||| | ||||| ||  | | ||  ||
Sbjct  403  ACAACCATAATAAAAAGGAGAAGATGGAGATGGATTGGACATGTTCTGAGAAGAGAACAA  462

Query  100  GGGGATATCACAAAAACAGCACTCCACTGGACCCCAGAAGGTAAAAGAAAGAGAGGAAGA  159
            |    |||    |  || || ||||||||||| || || ||     | || || || |||
Sbjct  463  GACTCTATTGTGATGACTGCCCTCCACTGGACACCCGAGGGACGTCGCAAAAGGGGCAGA  522

Query  160  CCTAAAATGACATGGAGAAGAACTGTAGAG  189
            || ||| |||| |||||  | ||  |||||
Sbjct  523  CCCAAAGTGACCTGGAGTTGTACATTAGAG  552
```

>**gb|DW707361.1|** EST030842 Trichophyton rubrum cDNA library 7 Trichophyton rubrum cDNA clone plasmid:FUNGI_9_119B_85, mRNA sequence.
Length=353

```
 Score = 44.6 bits (48),  Expect = 0.21
 Identities = 53/69 (76%), Gaps = 6/69 (8%)
 Strand=Plus/Plus

Query  135  AGAAGGTAAAAGAAAGAGAGGAAGACCTAAAATGAC---ATGGAGAAGAACTGTAGAGGC  191
            ||||||| || ||| |||||||||||| | || |||   | |||||||||  | ||| |
Sbjct  239  AGAAGGTGAA-GAAGGAGAGGAAGACCAAGAAGGACAAGAAGGAGAAGAA--GGAGAAGA  295

Query  192  AGAGGCTAG  200
            ||| |||||
Sbjct  296  AGAAGCTAG  304
```

>**gb|DW702650.1|** EST026131 Trichophyton rubrum cDNA library 7 Trichophyton rubrum cDNA clone plasmid:FUNGI_9_059b_14, mRNA sequence.
Length=469

```
 Score = 44.6 bits (48),  Expect = 0.21
 Identities = 53/69 (76%), Gaps = 6/69 (8%)
 Strand=Plus/Plus

Query  135  AGAAGGTAAAAGAAAGAGAGGAAGACCTAAAATGAC---ATGGAGAAGAACTGTAGAGGC  191
            ||||||| || ||| |||||||||||| | || |||   | |||||||||  | ||| |
Sbjct  238  AGAAGGTGAA-GAAGGAGAGGAAGACCAAGAAGGACAAGAAGGAGAAGAA--GGAGAAGA  294

Query  192  AGAGGCTAG  200
            ||| |||||
Sbjct  295  AGAAGCTAG  303
```

>**gb|CN200508.1|** Tor10181 Gametophyte rehydration Library Syntrichia ruralis cDNA, mRNA sequence.
Length=849

```
 Score = 44.6 bits (48),  Expect = 0.21
 Identities = 29/32 (90%), Gaps = 0/32 (0%)
 Strand=Plus/Minus

Query  137  AAGGTAAAAGAAAGAGAGGAAGACCTAAAATG  168
            || |||||||||||| || |||||||||||||
Sbjct  686  AAAGTAAAAGAAAGAAAGAAAGACCTAAAATG  655
```

>**gb|CK876667.1|** SGP137954 Atlantic salmon Eye cDNA library Salmo salar cDNA clone OY4-0858 5', mRNA sequence.
Length=551

```
 Score = 44.6 bits (48),  Expect = 0.21
 Identities = 38/47 (80%), Gaps = 0/47 (0%)
 Strand=Plus/Minus

Query  142  AAAAGAAAGAGAGGAAGACCTAAAATGACATGGAGAAGAACTGTAGA  188
            || ||||||| |||| | || |||  |||||||||||||||  ||||
Sbjct  72   AAGAGAAAGACAGGACGCCCAAAAGAGACATGGAGAAGAACAATAGA  26
```

>**gb|GD213660.1|** G1045P365RD9.T1 Aplysia californica Pooled Normalized Library Aplysia californica cDNA, mRNA sequence.
Length=835

```
 Score = 42.8 bits (46),  Expect = 0.73
 Identities = 67/94 (71%), Gaps = 4/94 (4%)
 Strand=Plus/Plus

Query  54   AAGAAGACGCTGGAGGTGGATTCGGCATGTCCTACGGAAAGGCCAGGGGGAT--ATCACA  111
            |||||| || ||| | |||||| | ||||| ||  |||||   ||||  | |  || |||
Sbjct  164  AAGAAGGCGGTGGCGATGGATTAGACATGTTCTCGGGAAAT--CAGGCAGCTCCATAACA  221

Query  112  AAAACAGCACTCCACTGGACCCCAGAAGGTAAAA  145
            || |||| |||| | | ||  ||| | |||||||
Sbjct  222  AAGACAGTACTCGATTTGAATCCACAGGGTAAAA  255
```

>**gb|FG561909.1|** BN18DYSC_UP_107_G12_31MAR2008_084 BN18DYSC Brassica napus cDNA 5', mRNA sequence.
Length=759

```
 Score = 42.8 bits (46),  Expect = 0.73
 Identities = 29/33 (87%), Gaps = 0/33 (0%)
 Strand=Plus/Plus

Query  149  AGAGAGGAAGACCTAAAATGACATGGAGAAGAA  181
            ||||||| |||||||||| |  |||||||||||
Sbjct  2    AGAGAGGGAGACCTAAAAAGCTATGGAGAAGAA  34
```

>**gb|EX838591.1|** CBNB7529.fwd CBNB Phycomyces blakesleeanus NRRL1555 Vegetative mycelium 48h old Dark L Phycomyces blakesleeanus cDNA clone CBNB7529 5', mRNA sequence.
Length=699

```
 Score = 42.8 bits (46),  Expect = 0.73
 Identities = 36/43 (83%), Gaps = 1/43 (2%)
 Strand=Plus/Plus

Query  135  AGAAGGTAAAAGAAAGAGAGGAAGACCTAAAATGACATGGAGA  177
            |||||| |||||||||||| ||||| | | || |||| |||||
Sbjct  212  AGAAGG-AAAAGAAAGAGAAGAAGAGCAAGAAAGACAAGGAGA  253
```

>**gb|EX838590.1|** CBNB7529.rev CBNB Phycomyces blakesleeanus NRRL1555 Vegetative mycelium 48h old Dark L Phycomyces blakesleeanus cDNA clone CBNB7529 3', mRNA sequence.
Length=699

```
 Score = 42.8 bits (46),  Expect = 0.73
 Identities = 36/43 (83%), Gaps = 1/43 (2%)
 Strand=Plus/Minus

Query  135  AGAAGGTAAAAGAAAGAGAGGAAGACCTAAAATGACATGGAGA  177
            |||||| |||||||||||| ||||| | | || |||| |||||
Sbjct  488  AGAAGG-AAAAGAAAGAGAAGAAGAGCAAGAAAGACAAGGAGA  447
```

>**gb|EX817665.1|** CBNA5689.fwd CBNA Phycomyces blakesleeanus NRRL1555 Vegetative mycelium 48h old Dark H Phycomyces blakesleeanus cDNA clone CBNA5689 5', mRNA sequence.
Length=761

```
 Score = 42.8 bits (46),  Expect = 0.73
 Identities = 36/43 (83%), Gaps = 1/43 (2%)
 Strand=Plus/Plus

Query  135  AGAAGGTAAAAGAAAGAGAGGAAGACCTAAAATGACATGGAGA  177
            |||||| |||||||||||| ||||| | | || |||| |||||
Sbjct  251  AGAAGG-AAAAGAAAGAGAAGAAGAGCAAGAAAGACAAGGAGA  292
```

>**gb|EX817664.1|** CBNA5689.rev CBNA Phycomyces blakesleeanus NRRL1555 Vegetative mycelium 48h old Dark H Phycomyces blakesleeanus cDNA clone CBNA5689 3', mRNA sequence.
Length=607

```
 Score = 42.8 bits (46),  Expect = 0.73
 Identities = 36/43 (83%), Gaps = 1/43 (2%)
 Strand=Plus/Minus

Query  135  AGAAGGTAAAAGAAAGAGAGGAAGACCTAAAATGACATGGAGA  177
            |||||| |||||||||||| ||||| | | || |||| |||||
Sbjct  509  AGAAGG-AAAAGAAAGAGAAGAAGAGCAAGAAAGACAAGGAGA  468
```

>**emb|AM847008.1|** AM847008 COL, cold overnight library Nicotiana tabacum cDNA clone nt006035064, mRNA sequence.
Length=545

```
 Score = 42.8 bits (46),  Expect = 0.73
 Identities = 35/43 (81%), Gaps = 0/43 (0%)
 Strand=Plus/Plus

Query  139  GGTAAAAGAAAGAGAGGAAGACCTAAAATGACATGGAGAAGAA  181
            |||||||| ||  |||| ||||||||||| || || |||| ||
Sbjct  66   GGTAAAAGCAAATGAGGTAGACCTAAAATCACGTGAAGAAAAA  108
```

>**gb|EB238537.1|** PEG003-C-224292-501 Normalized Pedal-Pleural Ganglia Aplysia californica cDNA clone PEG003-C-224292 5', mRNA sequence.
Length=692

```
 Score = 42.8 bits (46),  Expect = 0.73
 Identities = 50/68 (73%), Gaps = 0/68 (0%)
 Strand=Plus/Minus

Query  127  TGGACCCCAGAAGGTAAAAGAAAGAGAGGAAGACCTAAAATGACATGGAGAAGAACTGTA  186
            ||||  ||| |||| ||| | ||||  |||||||| || |||| ||||||   | | |||
Sbjct  549  TGGAATCCACAAGGAAAACGTAAGAAGGGAAGACCAAAGATGATATGGAGGCAAGCGGTA  490

Query  187  GAGGCAGA  194
             | |||||
Sbjct  489  CAAGCAGA  482
```

>**gb|EB226379.1|** PEG002-C-108747-501 Normalized Pedal-Pleural Ganglia Aplysia californica cDNA clone PEG002-C-108747 5', mRNA sequence.
Length=598

```
 Score = 42.8 bits (46),  Expect = 0.73
 Identities = 50/68 (73%), Gaps = 0/68 (0%)
 Strand=Plus/Minus

Query  127  TGGACCCCAGAAGGTAAAAGAAAGAGAGGAAGACCTAAAATGACATGGAGAAGAACTGTA  186
            |||| |||| |||| ||  | ||||  || ||||| || |||||||||||  || | |||
Sbjct  224  TGGAACCCACAAGGAAAGCGTAAGAAGGGGAGACCAAAGATGACATGGAGGCGAGCGGTA  165

Query  187  GAGGCAGA  194
             | || ||
Sbjct  164  CAAGCTGA  157
```

>**gb|EG830862.1|** EST_ssal_eve_43068 ssaleve thyroid Salmo salar cDNA Salmo salar cDNA clone ssal_eve_558_218_rev 3', mRNA sequence.
Length=620

```
 Score = 42.8 bits (46),  Expect = 0.73
 Identities = 37/46 (80%), Gaps = 0/46 (0%)
 Strand=Plus/Minus

Query  134  CAGAAGGTAAAAGAAAGAGAGGAAGACCTAAAATGACATGGAGAAG  179
            ||||| | | ||||||||||||||||| || || |||| | | |||
Sbjct  168  CAGAATGGAGAAGAAAGAGAGGAAGACATAGAAAGACAAGAACAAG  123
```

>**dbj|BW777455.1|** BW777455 Amphioxus Branchiostoma floridae unpublished cDNA library, gastrula whole animal Branchiostoma floridae cDNA clone bfga045o16 5', mRNA sequence.
Length=541

```
 Score = 42.8 bits (46),  Expect = 0.73
 Identities = 58/81 (71%), Gaps = 0/81 (0%)
 Strand=Plus/Plus

Query  105  TATCACAAAAACAGCACTCCACTGGACCCCAGAAGGTAAAAGAAAGAGAGGAAGACCTAA  164
            |||| | |||   || || || ||| |||| || || || |||||  | ||| | |||||
Sbjct  274  TATCCCCAAAGTTGCTCTACATTGGGCCCCTGAGGGAAAGAGAAAACGGGGACGTCCTAA  333

Query  165  AATGACATGGAGAAGAACTGT  185
            ||  ||||||||  | |||||
Sbjct  334  AACCACATGGAGGCGCACTGT  354
```

>**gb|CV297687.1|** EST886064 petunia floral development cDNA library Petunia x hybrida cDNA clone Petunia-DevA-18RR-F07 5' end, mRNA sequence.
Length=524

```
 Score = 42.8 bits (46),  Expect = 0.73
 Identities = 32/38 (84%), Gaps = 0/38 (0%)
 Strand=Plus/Minus

Query  87   ACGGAAAGGCCAGGGGGATATCACAAAAACAGCACTCC  124
            |||||||| | |||||||| ||| | |||||| |||||
Sbjct  297  ACGGAAAGACTAGGGGGATTTCATATAAACAGAACTCC  260
```

>**emb|AJ672649.1|** AJ672649 KN224 Bos taurus cDNA clone KN224-009_N08, mRNA sequence.
Length=365

```
 Score = 42.8 bits (46),  Expect = 0.73
 Identities = 40/50 (80%), Gaps = 1/50 (2%)
 Strand=Plus/Minus

Query  149  AGAGA-GGAAGACCTAAAATGACATGGAGAAGAACTGTAGAGGCAGAGGC  197
            ||||| ||||||||| ||| |||| |||||| || || ||||   |||||
Sbjct  281  AGAGAAGGAAGACCTTAAAAGACACGGAGAATAAGTGCAGAGTTTGAGGC  232
```

>**gb|CD096407.1|** ME1-0008T-L087-D01-U.B ME1-0008 Schistosoma mansoni cDNA clone ME1-0008T-L087-D01.B, mRNA sequence.
Length=593

```
 Score = 42.8 bits (46),  Expect = 0.73
 Identities = 49/66 (74%), Gaps = 0/66 (0%)
 Strand=Plus/Plus

Query  107  TCACAAAAACAGCACTCCACTGGACCCCAGAAGGTAAAAGAAAGAGAGGAAGACCTAAAA  166
            |||||| |  ||| |||   ||||  || |||||| ||||||  ||||||||||| || |
Sbjct  163  TCACAAGACAAGCCCTCACATGGAGTCCTGAAGGTCAAAGAAGAAGAGGAAGACCAAAGA  222
```

```
Query  167  TGACAT  172
              ||||
Sbjct  223  ACACAT  228
```

>gb|CD815515.1| BN15.026J08F020214 BN15 Brassica napus cDNA clone BN15026J08, mRNA sequence.
Length=732

```
 Score = 42.8 bits (46),  Expect = 0.73
 Identities = 29/33 (87%), Gaps = 0/33 (0%)
 Strand=Plus/Plus

Query  149  AGAGAGGAAGACCTAAAATGACATGGAGAAGAA  181
            ||||||| |||||||||| |  |||||||||||
Sbjct  8    AGAGAGGGAGACCTAAAAAGCTATGGAGAAGAA  40
```

>gb|BI976185.1| 484936 MARC 2BOV Bos taurus cDNA 5', mRNA sequence.
Length=571

```
 Score = 42.8 bits (46),  Expect = 0.73
 Identities = 40/50 (80%), Gaps = 1/50 (2%)
 Strand=Plus/Minus

Query  149  AGAGA-GGAAGACCTAAAATGACATGGAGAAGAACTGTAGAGGCAGAGGC  197
            ||||| ||||||||| ||| |||| |||||| || || ||||   |||||
Sbjct  341  AGAGAAGGAAGACCTTAAAAGACACGGAGAATAAGTGCAGAGTTTGAGGC  292
```

>gb|GO780561.1| 001020OFSA008082HT OFSA Ovis aries cDNA 5', mRNA sequence.
Length=606

```
 Score = 41.0 bits (44),  Expect = 2.5
 Identities = 27/29 (93%), Gaps = 1/29 (3%)
 Strand=Plus/Plus

Query  58  AGACGCTGGAGGTGGAT-TCGGCATGTCC  85
           ||||||||||||||||| ||| |||||||
Sbjct  55  AGACGCTGGAGGTGGATGTCGACATGTCC  83
```

>gb|FL490534.1| Mg_Nor01_49D21 Nor01 Mytilus galloprovincialis cDNA 3', mRNA sequence.
Length=784

```
 Score = 41.0 bits (44),  Expect = 2.5
 Identities = 28/32 (87%), Gaps = 0/32 (0%)
 Strand=Plus/Plus

Query  135  AGAAGGTAAAAGAAAGAGAGGAAGACCTAAAA  166
            |||||| || ||||||||||||||||  ||||
Sbjct  394  AGAAGGAAAGAGAAAGAGAGGAAGACAGAAAA  425
```

>dbj|BP999030.1| BP999030 Perionyx excavatus regenerating tissue cDNA library Perionyx excavatus cDNA clone PER10677 3' similar to reverse transcriptase family member (1F383), mRNA sequence.
Length=603

```
 Score = 41.0 bits (44),  Expect = 2.5
 Identities = 37/44 (84%), Gaps = 2/44 (4%)
 Strand=Plus/Minus

Query  133  CCAGAAGGTAAAAGAAAGAGAGGAAGACCTAAAATGAC-ATGGA  175
            |||||||| ||||||   ||||||||||| |||| ||| |||||
Sbjct  264  CCAGAAGGAAAAAGATCCAGAGGAAGACCGAAAA-GACGATGGA  222
```

>gb|ES406593.1| MUT08-N18.x1d-t SHGC-MUT Mytilus californianus cDNA 5', mRNA sequence.
Length=1035

```
 Score = 41.0 bits (44),  Expect = 2.5
 Identities = 36/45 (80%), Gaps = 0/45 (0%)
 Strand=Plus/Plus

Query  129  GACCCCAGAAGGTAAAAGAAAGAGAGGAAGACCTAAAATGACATG  173
            ||| |||||  ||| || ||||||||| ||||| || | ||||||
Sbjct  232  GACACCAGATAGTACAAAAAAGAGAGGCAGACCAAAGAAGACATG  276
```

>gb|ES401741.1| MUT03-D23.y1d-s SHGC-MUT Mytilus californianus cDNA 3', mRNA sequence.
Length=954

```
 Score = 41.0 bits (44),  Expect = 2.5
 Identities = 27/30 (90%), Gaps = 0/30 (0%)
 Strand=Plus/Minus

Query  137  AAGGTAAAAGAAAGAGAGGAAGACCTAAAA  166
            ||| |||||||||||| || ||||||||||
Sbjct  197  AAGTTAAAAGAAAGAGGGGTAGACCTAAAA  168
```

>gb|EH639442.1| EST10550 LK04 Laupala kohalensis cDNA clone 1061021774416 5', mRNA sequence.
Length=719

```
 Score = 41.0 bits (44),  Expect = 2.5
 Identities = 30/35 (85%), Gaps = 0/35 (0%)
 Strand=Plus/Plus

Query  139  GGTAAAAGAAAGAGAGGAAGACCTAAAATGACATG  173
            || || ||||| |||||||||||||||| || |||
Sbjct  322  GGAAATAGAAAAAGAGGAAGACCTAAAAAGAAATG  356


>gb|EH634555.1|  EST5663 LK04 Laupala kohalensis cDNA clone 1061021821420 5',
mRNA sequence.
Length=883

 Score = 41.0 bits (44),  Expect = 2.5
 Identities = 30/35 (85%), Gaps = 0/35 (0%)
 Strand=Plus/Plus

Query  139  GGTAAAAGAAAGAGAGGAAGACCTAAAATGACATG  173
            || || ||||| |||||||||||||||| || |||
Sbjct  109  GGAAATAGAAAAAGAGGAAGACCTAAAAAGAAATG  143


>gb|EB320170.1|  CNSN01-F-085761-501 Normalized CNS library (juvenile 1) Aplysia
californica cDNA clone CNSN01-F-085761 5', mRNA sequence.
Length=673

 Score = 41.0 bits (44),  Expect = 2.5
 Identities = 51/70 (72%), Gaps = 0/70 (0%)
 Strand=Plus/Minus

Query  125  ACTGGACCCCAGAAGGTAAAAGAAAGAGAGGAAGACCTAAAATGACATGGAGAAGAACTG  184
            ||||||| || |||||  |  |  | || || ||||| |||  ||| |||||| | || |
Sbjct  592  ACTGGACTCCCGAAGGACATCGCGAAAGGGGCAGACCCAAAGAGACCTGGAGACGCACAG  533

Query  185  TAGAGGCAGA  194
            ||||||| ||
Sbjct  532  TAGAGGCTGA  523


>gb|EB317104.1|  CNSN01-F-083310-501 Normalized CNS library (juvenile 1) Aplysia
californica cDNA clone CNSN01-F-083310 5', mRNA sequence.
Length=711

 Score = 41.0 bits (44),  Expect = 2.5
 Identities = 51/70 (72%), Gaps = 0/70 (0%)
 Strand=Plus/Minus

Query  125  ACTGGACCCCAGAAGGTAAAAGAAAGAGAGGAAGACCTAAAATGACATGGAGAAGAACTG  184
            ||||||| || |||||  |  |  | || || ||||| |||  ||| |||||| | || |
Sbjct  599  ACTGGACTCCCGAAGGACATCGCGAAAGGGGCAGACCCAAAGAGACCTGGAGACGCACAG  540

Query  185  TAGAGGCAGA  194
            ||||||| ||
Sbjct  539  TAGAGGCTGA  530


>gb|EB313998.1|  CNSN01-F-078486-501 Normalized CNS library (juvenile 1) Aplysia
californica cDNA clone CNSN01-F-078486 5', mRNA sequence.
Length=522

 Score = 41.0 bits (44),  Expect = 2.5
 Identities = 51/70 (72%), Gaps = 0/70 (0%)
 Strand=Plus/Minus

Query  125  ACTGGACCCCAGAAGGTAAAAGAAAGAGAGGAAGACCTAAAATGACATGGAGAAGAACTG  184
            ||||||| || |||||  |  |  | || || ||||| |||  ||| |||||| | || |
Sbjct  413  ACTGGACTCCCGAAGGACATCGCGAAAGGGGCAGACCCAAAGAGACCTGGAGACGCACAG  354

Query  185  TAGAGGCAGA  194
            ||||||| ||
Sbjct  353  TAGAGGCTGA  344


>gb|EB304909.1|  CNSN01-F-064655-501 Normalized CNS library (juvenile 1) Aplysia
californica cDNA clone CNSN01-F-064655 5', mRNA sequence.
Length=206

 Score = 41.0 bits (44),  Expect = 2.5
 Identities = 39/50 (78%), Gaps = 0/50 (0%)
 Strand=Plus/Minus

Query  127  TGGACCCCAGAAGGTAAAAGAAAGAGAGGAAGACCTAAAATGACATGGAG  176
            |||| |||| ||||  ||   ||||  |||||||| || |||||||||||
Sbjct  167  TGGAACCCACAAGGAGAACATAAGAAGGGAAGACCAAAGATGACATGGAG  118


>gb|EB276974.1|  CNSN01-F-090235-501 Normalized CNS library (juvenile 1) Aplysia
californica cDNA clone CNSN01-F-090235 5', mRNA sequence.
Length=612

 Score = 41.0 bits (44),  Expect = 2.5
 Identities = 35/42 (83%), Gaps = 1/42 (2%)
 Strand=Plus/Plus

Query  137  AAGGTAAAA-GAAAGAGAGGAAGACCTAAAATGACATGGAGA  177
            |||| |||| | ||||  |||||||| || ||||||||||||
```

```
Sbjct  303  AAGGAAAAACGTAAGAAGGGAAGACCAAAGATGACATGGAGA  344


>gb|EB245706.1|  PEG003-C-228360-501 Normalized Pedal-Pleural Ganglia Aplysia
californica cDNA clone PEG003-C-228360 5', mRNA sequence.
Length=634

 Score = 41.0 bits (44),  Expect = 2.5
 Identities = 33/40 (82%), Gaps = 0/40 (0%)
 Strand=Plus/Minus

Query  127  TGGACCCCAGAAGGTAAAAGAAAGAGAGGAAGACCTAAAA  166
            |||| ||||  ||| || | ||||| ||||||||||||||
Sbjct  329  TGGAACCCACTAGGAAAGAAAAAGAAAGGAAGACCTAAAA  290


>gb|EB236260.1|  PEG003-C-211457-501 Normalized Pedal-Pleural Ganglia Aplysia
californica cDNA clone PEG003-C-211457 5', mRNA sequence.
Length=720

 Score = 41.0 bits (44),  Expect = 2.5
 Identities = 51/70 (72%), Gaps = 0/70 (0%)
 Strand=Plus/Minus

Query  125  ACTGGACCCCAGAAGGTAAAAGAAAGAGAGGAAGACCTAAAATGACATGGAGAAGAACTG  184
            ||||||| || |||||  |  |  | || || ||||| |||  ||| |||||| | || |
Sbjct  322  ACTGGACTCCCGAAGGACATCGCGAAAGGGGCAGACCCAAAGAGACCTGGAGACGCACAG  263

Query  185  TAGAGGCAGA  194
            ||||||| ||
Sbjct  262  TAGAGGCTGA  253


>gb|DY020678.1|  53COT5_T3_008_H12_19JUL2004_082 Brassica napus 36hr germinating
seed library 53COT5 Brassica napus cDNA 5', mRNA sequence.
Length=755

 Score = 41.0 bits (44),  Expect = 2.5
 Identities = 28/32 (87%), Gaps = 0/32 (0%)
 Strand=Plus/Plus

Query  150  GAGAGGAAGACCTAAAATGACATGGAGAAGAA  181
            |||||| |||||||||| |  |||||||||||
Sbjct  11   GAGAGGGAGACCTAAAAAGCTATGGAGAAGAA  42


>gb|EC021868.1|  3742948 KZ41 Caenorhabditis elegans cDNA clone 1414262, mRNA
sequence.
Length=418

 Score = 41.0 bits (44),  Expect = 2.5
 Identities = 35/41 (85%), Gaps = 2/41 (4%)
 Strand=Plus/Plus

Query  142  AAAAGAAAGAGAGGAAG-ACCTAAAATGACATGGAGAAGAA  181
            |||||||||||| |||| |||| ||| || || ||||||||
Sbjct  296  AAAAGAAAGAGAAGAAGAACCTGAAA-GAAATAGAGAAGAA  335


>gb|EC003640.1|  7403423 CE04 Caenorhabditis elegans cDNA clone 2711032, mRNA
sequence.
Length=410

 Score = 41.0 bits (44),  Expect = 2.5
 Identities = 35/41 (85%), Gaps = 2/41 (4%)
 Strand=Plus/Plus

Query  142  AAAAGAAAGAGAGGAAG-ACCTAAAATGACATGGAGAAGAA  181
            |||||||||||| |||| |||| ||| || || ||||||||
Sbjct  295  AAAAGAAAGAGAAGAAGAACCTGAAA-GAAATAGAGAAGAA  334


>gb|EB170901.1|  EST005800 injuried spinal cord cDNA library in Gecko Gekko japonicus
cDNA clone SH_36_29, mRNA sequence.
Length=684

 Score = 41.0 bits (44),  Expect = 2.5
 Identities = 33/40 (82%), Gaps = 0/40 (0%)
 Strand=Plus/Minus

Query  136  GAAGGTAAAAGAAAGAGAGGAAGACCTAAAATGACATGGA  175
            ||||| | ||| || ||||||||||| || |||| |||||
Sbjct  217  GAAGGCAGAAGGAAAAGAGGAAGACCCAACATGAGATGGA  178


>gb|DY502648.1|  sh2P0044I13_F.ab1 adult sheep fracture callus 7d Ovis aries cDNA,
mRNA sequence.
Length=800

 Score = 41.0 bits (44),  Expect = 2.5
 Identities = 27/29 (93%), Gaps = 1/29 (3%)
 Strand=Plus/Plus

Query  58   AGACGCTGGAGGTGGAT-TCGGCATGTCC  85
            ||||||||||||||||| ||| |||||||
Sbjct  718  AGACGCTGGAGGTGGATGTCGACATGTCC  746
```

```
>gb|DW559163.1|  EST_ssal_rgb2_23582 rgb2 Salmo salar cDNA clone ssal_rgb2_538_115_rev
5', mRNA sequence.
Length=713

 Score = 41.0 bits (44),  Expect = 2.5
 Identities = 52/72 (72%), Gaps = 0/72 (0%)
 Strand=Plus/Minus

Query  106  ATCACAAAAACAGCACTCCACTGGACCCCAGAAGGTAAAAGAAAGAGAGGAAGACCTAAA  165
            |||||||||   || ||    |||| |||| |||| ||  |  | |||||||||||||||
Sbjct  322  ATCACAAAACAGGCCCTAACATGGAACCCACAAGGCAACCGCTAAAGAGGAAGACCTAAA  263

Query  166  ATGACATGGAGA  177
            |  || | ||||
Sbjct  262  ACCACCTAGAGA  251


>emb|AM048069.1|  AM048069 Schistosoma mansoni lung schistosomulum Schistosoma
mansoni cDNA clone SmlC54g08.q1k, mRNA sequence.
Length=613

 Score = 41.0 bits (44),  Expect = 2.5
 Identities = 81/119 (68%), Gaps = 1/119 (0%)
 Strand=Plus/Plus

Query  54   AAGAAGACGCTGGAGGTGGATTCGGCATGTCCTACGGAAAGGCCAGGGGGATATCACAAA  113
            |||||| ||||||| ||||||| ||||   | |  |||||   |       | ||||||
Sbjct  260  AAGAAG-CGCTGGAAGTGGATTGGGCACACCTTGAGGAAATCACTTAATTGTGTCACAAG  318

Query  114  AACAGCACTCCACTGGACCCCAGAAGGTAAAAGAAAGAGAGGAAGACCTAAAATGACAT  172
            |  ||| |||   ||||  || |||||| |||| |  ||||||||||| || |  ||||
Sbjct  319  ACAAGCCCTCACATGGAATCCTGAAGGTCAAAGGAGAAGAGGAAGACCAAAGAACACAT  377


>gb|DN563352.1|  90876293 Sea Urchin primary mesenchyme cell cDNA library Strongylocentrotus
purpuratus cDNA clone PMCSPR2-121A11 3', mRNA
sequence.
Length=1071

 Score = 41.0 bits (44),  Expect = 2.5
 Identities = 22/22 (100%), Gaps = 0/22 (0%)
 Strand=Plus/Plus

Query  138  AGGTAAAAGAAAGAGAGGAAGA  159
            ||||||||||||||||||||||
Sbjct  953  AGGTAAAAGAAAGAGAGGAAGA  974


>gb|CN633496.1|  taf11f01.y1 Hydra EST Darmstadt I Hydra magnipapillata cDNA 5',
mRNA sequence.
Length=589

 Score = 41.0 bits (44),  Expect = 2.5
 Identities = 29/32 (90%), Gaps = 1/32 (3%)
 Strand=Plus/Plus

Query  142  AAAAGAAAGAGAGGAAGACCTAAAATGACATG  173
            || ||||||||||||||||| |||||| ||||
Sbjct  270  AAGAGAAAGAGAGGAAGACCAAAAATG-CATG  300


>gb|CF503562.1|  ML1-0002T-M131-C01-U.G ML1-0002 Schistosoma mansoni cDNA clone
ML1-0002T-M131-C01.G similar to putative retrotransposon,
mRNA sequence.
Length=623

 Score = 41.0 bits (44),  Expect = 2.5
 Identities = 46/59 (77%), Gaps = 2/59 (3%)
 Strand=Plus/Minus

Query  107  TCACAAAAACAGCACTC-CACTGGACCCCAGAAGGTAAAAGAAAGAGAGGAAGACCTAA  164
            |||||| |  ||| ||| ||| |||  || |||||| ||||||  ||||||||||| ||
Sbjct  581  TCACAAGACAAGCCCTCACAC-GGAGTCCTGAAGGTCAAAGAAGAAGAGGAAGACCAAA  524


>emb|AL789715.2|  AL789715 XGC-neurula Xenopus (Silurana) tropicalis cDNA clone
TNeu133e22 5', mRNA sequence.
Length=468

 Score = 41.0 bits (44),  Expect = 2.5
 Identities = 30/34 (88%), Gaps = 1/34 (2%)
 Strand=Plus/Minus

Query  142  AAAAGAAAGAGAGGAAGACCT-AAAATGACATGG  174
            |||||||| |||||||| ||| ||||||||| ||
Sbjct  163  AAAAGAAAAAGAGGAAGCCCTCAAAATGACAAGG  130


>gb|CD066558.1|  MA1-0049T-R059-D06-U.G MA1-0049 Schistosoma mansoni cDNA clone
MA1-0049T-R059-D06.G, mRNA sequence.
Length=543

 Score = 41.0 bits (44),  Expect = 2.5
 Identities = 49/67 (73%), Gaps = 0/67 (0%)
 Strand=Plus/Plus
```

```
Query  106  ATCACAAAAACAGCACTCCACTGGACCCCAGAAGGTAAAAGAAAGAGAGGAAGACCTAAA  165
            ||||||| |  | | |||   ||||  || |||||| |||| |  ||||||||||||||
Sbjct  54   ATCACAAGACAACCCCTCACATGGAATCCTGAAGGTCAAAGGACAAGAGGAAGACCTAAG  113

Query  166  ATGACAT  172
            |  ||||
Sbjct  114  AACACAT  120
```

>**gb|CF445071.1|** EST681416 normalized cDNA library of onion Allium cepa cDNA clone ACAIH85, mRNA sequence.
Length=692

```
 Score = 41.0 bits (44),  Expect = 2.5
 Identities = 22/22 (100%), Gaps = 0/22 (0%)
 Strand=Plus/Plus

Query  142  AAAAGAAAGAGAGGAAGACCTA  163
            ||||||||||||||||||||||
Sbjct  113  AAAAGAAAGAGAGGAAGACCTA  134
```

>**gb|CB696383.1|** AMGNNUC:SRPB2-00223-H2-A srpb2 (10220) Rattus norvegicus cDNA clone srpb2-00223-h2 5', mRNA sequence.
Length=425

```
 Score = 41.0 bits (44),  Expect = 2.5
 Identities = 40/52 (76%), Gaps = 0/52 (0%)
 Strand=Plus/Minus

Query  145  AGAAAGAGAGGAAGACCTAAAATGACATGGAGAAGAACTGTAGAGGCAGAGG  196
            ||| ||| ||  ||| | ||| ||||||||||||| || | | ||| |||||
Sbjct  285  AGAGAGATAGAGAGAGCAAAAGTGACATGGAGAAGTACGGAAAAGGAAGAGG  234
```

>**gb|BU432148.1|** 603831116F1 CSEQRBN10 Gallus gallus cDNA clone ChEST821p3 5', mRNA sequence.
Length=689

```
 Score = 41.0 bits (44),  Expect = 2.5
 Identities = 35/41 (85%), Gaps = 2/41 (4%)
 Strand=Plus/Plus

Query  129  GACCCCAGAAGGTAAAAGAAAGAG-AGGAAGACCTAAAATG  168
            ||||||| ||||||||||||| || ||||||  |||| |||
Sbjct  114  GACCCCA-AAGGTAAAAGAAATAGTAGGAAGTGCTAAGATG  153
```

>**gb|CA330194.1|** hab02a12.y1 Fugu hgmpJ adult kidney Takifugu rubripes cDNA clone 6362398 5', mRNA sequence.
Length=266

```
 Score = 41.0 bits (44),  Expect = 2.5
 Identities = 33/39 (84%), Gaps = 1/39 (2%)
 Strand=Plus/Plus

Query  143  AAAGAAAGAGAGGAAGACCTAAAATGACATGGAGAAGAA  181
            |||||||||||||||||  ||||| ||| |||| || ||
Sbjct  225  AAAGAAAGAGAGGAAGAGATAAAAAGAC-TGGAAAAAAA  262
```

>**dbj|AU140774.2|** AU140774 PLACE4 Homo sapiens cDNA clone PLACE4000206 5', mRNA sequence.
Length=891

```
 Score = 41.0 bits (44),  Expect = 2.5
 Identities = 25/27 (92%), Gaps = 0/27 (0%)
 Strand=Plus/Plus

Query  135  AGAAGGTAAAAGAAAGAGAGGAAGACC  161
            ||||||||||||||| | |||||||||
Sbjct  629  AGAAGGTAAAAGAAACACAGGAAGACC  655
```

>**emb|AL698619.1|** DKFZp686K22109_r1 686 (synonym: hlcc3) Homo sapiens cDNA clone DKFZp686K22109 5', mRNA sequence.
Length=671

```
 Score = 41.0 bits (44),  Expect = 2.5
 Identities = 25/27 (92%), Gaps = 0/27 (0%)
 Strand=Plus/Plus

Query  135  AGAAGGTAAAAGAAAGAGAGGAAGACC  161
            ||||||||||||||| | |||||||||
Sbjct  630  AGAAGGTAAAAGAAACACAGGAAGACC  656
```

>**gb|BG499451.1|** 602546677F1 NIH_MGC_60 Homo sapiens cDNA clone IMAGE:4669146 5', mRNA sequence.
Length=561

```
 Score = 41.0 bits (44),  Expect = 2.5
 Identities = 45/59 (76%), Gaps = 2/59 (3%)
 Strand=Plus/Plus

Query  103  GATATCACAAAAACAGCAC--TCCACTGGACCCCAGAAGGTAAAAGAAAGAGAGGAAGA  159
            ||||||||||  || || |  ||| |||||   |||||||  || |||||||||| |||
Sbjct  216  GATATCACAATTACGGCCCCGTCCCCTGGAGAACAGAAGGAGAAGGAAAGAGAGGGAGA  274
```

```
>dbj|AU132863.1|  AU132863 NT2RP4 Homo sapiens cDNA clone NT2RP4000740 5', mRNA
sequence.
Length=685

 Score = 41.0 bits (44),  Expect = 2.5
 Identities = 25/27 (92%), Gaps = 0/27 (0%)
 Strand=Plus/Plus

Query  137  AAGGTAAAAGAAAGAGAGGAAGACCTA  163
            ||||||||||||| | |||||||||||
Sbjct  630  AAGGTAAAAGAAACACAGGAAGACCTA  656


>gb|GR672193.1|  cN_n_V10_7_N07.ab1_c Tilapia adult ovary library Oreochromis
niloticus cDNA 5', mRNA sequence.
Length=603
                                                                  Sort alignments for this subject sequence by:
                                                                    E value  Score  Percent identity
                                                                    Query start position  Subject start position

 Score = 39.2 bits (42),  Expect = 8.9
 Identities = 24/26 (92%), Gaps = 0/26 (0%)
 Strand=Plus/Plus

Query  137  AAGGTAAAAGAAAGAGAGGAAGACCT  162
            ||||||||||||||| || |||||||
Sbjct  12   AAGGTAAAAGAAAGAAAGAAAGACCT  37


 Score = 39.2 bits (42),  Expect = 8.9
 Identities = 24/26 (92%), Gaps = 0/26 (0%)
 Strand=Plus/Plus

Query  137  AAGGTAAAAGAAAGAGAGGAAGACCT  162
            ||||||||||||||| || |||||||
Sbjct  185  AAGGTAAAAGAAAGAAAGAAAGACCT  210


>gb|GO645697.1|  EST_afim_evh_1056146 afimevh mixed_tissue Anoplopoma fimbria
cDNA Anoplopoma fimbria cDNA clone afim_evh_530_050 3', mRNA
sequence.
Length=685

 Score = 39.2 bits (42),  Expect = 8.9
 Identities = 23/24 (95%), Gaps = 0/24 (0%)
 Strand=Plus/Minus

Query  137  AAGGTAAAAGAAAGAGAGGAAGAC  160
            |||| |||||||||||||||||||
Sbjct  296  AAGGCAAAAGAAAGAGAGGAAGAC  273


>gb|GO645696.1|  EST_afim_evh_1054994 afimevh mixed_tissue Anoplopoma fimbria
cDNA Anoplopoma fimbria cDNA clone afim_evh_530_050 5', mRNA
sequence.
Length=685

 Score = 39.2 bits (42),  Expect = 8.9
 Identities = 23/24 (95%), Gaps = 0/24 (0%)
 Strand=Plus/Plus

Query  137  AAGGTAAAAGAAAGAGAGGAAGAC  160
            |||| |||||||||||||||||||
Sbjct  390  AAGGCAAAAGAAAGAGAGGAAGAC  413


>gb|GD065748.1|  KS15025G02 KS15 Capsicum annuum cDNA, mRNA sequence.
Length=648

 Score = 39.2 bits (42),  Expect = 8.9
 Identities = 34/41 (82%), Gaps = 1/41 (2%)
 Strand=Plus/Minus

Query  146  GAAAGAGAGGAAGACCTAAAATGACATGGAGA-AGAACTGT  185
            |||||||||||||||| | || || | ||||| |||| |||
Sbjct  215  GAAAGAGAGGAAGACCAAGAAAGAGAGGGAGAGAGAAGTGT  175


>emb|FM973896.1|  FM973896 T.brucei infected salivary gland Glossina morsitans
morsitans cDNA clone GMsg74h11.q1k, mRNA sequence.
Length=842

 Score = 39.2 bits (42),  Expect = 8.9
 Identities = 29/33 (87%), Gaps = 1/33 (3%)
 Strand=Plus/Plus

Query  134  CAGAAGGTAAAAGAAAGAGAGGAAGACCTAAAA  166
            ||||| | |||||||| |||||||||| |||||
Sbjct  36   CAGAAAG-AAAAGAAACAGAGGAAGACTTAAAA  67


>gb|FG294822.1|  1108770719004 New World Screwworm Larvae 9387 ESTs Cochliomyia
hominivorax cDNA, mRNA sequence.
Length=959

 Score = 39.2 bits (42),  Expect = 8.9
```

```
 Identities = 26/29 (89%), Gaps = 0/29 (0%)
 Strand=Plus/Plus

Query  30   ACAAGATATAGCCACAACTATAATAAGAA  58
            |||| ||||||| ||||| ||||||||||
Sbjct  65   ACAAAATATAGCAACAACAATAATAAGAA  93
```

>**gb|GE578522.1|** CCPU2630.b1_L10.ab1 CCP(UWX) Globe Artichoke Cynara scolymus
cDNA clone CCPU2630, mRNA sequence.
Length=798

```
 Score = 39.2 bits (42),  Expect = 8.9
 Identities = 37/46 (80%), Gaps = 1/46 (2%)
 Strand=Plus/Plus

Query  134  CAGAAGGTAAAAGA-AAGAGAGGAAGACCTAAAATGACATGGAGAA  178
            |||| | ||  ||| |||||||| ||||||| | ||| ||||||||
Sbjct  197  CAGATGTTAGGAGAGAAGAGAGGTAGACCTAGACTGAGATGGAGAA  242
```

Select All Get selected sequences Distance tree of results Multiple alignment NEW
