# Supplementary material for: Promoter Complexity and Tissue-Specific Expression of Stress Response Components in Mytilus galloprovincialis, a Sessile Marine Invertebrate Species
Source: PLoS Comput Biol. 2010 Jul 8;6(7):e1000847. doi: 10.1371/journal.pcbi.1000847 (PMC2900285; doi:10.1371/journal.pcbi.1000847)
Supplement: Protocol S1 — 18 Supplement files plus an index file: 3 Supplementary figures, 2 Supplementary tables - referenced in text as Protocol S1; index provided with an explanation of the directory contents. (5.18 MB ZIP) [file pcbi.1000847.s001.zip › SUPPLEMENTS18/SupplFigure2.3a.pdf]

BLAST Basic Local Alignment Search Tool

-

Edit and Resubmit Save Search Strategies Formatting options Download

Nucleotide Sequence (328 letters)

Results for: lcl|12019 None(201bp)

Your BLAST job specified more than one input sequence. This box lets you choose which input sequence to show BLAST results for.

Query ID
lcl|12019
Description
None
Molecule type
nucleic acid
Query Length
201

Database Name
htgs
Description
Unfinished High Throughput Genomic Sequences; Sequences: phases 0,1 and 2
Program
BLASTN 2.2.22+ Citation

Reference
Stephen F. Altschul, Thomas L. Madden, Alejandro A. Schäffer, Jinghui Zhang, Zheng Zhang, Webb Miller, and David J. Lipman (1997), "Gapped BLAST and PSI-BLAST: a new generation of protein database search programs", Nucleic Acids Res. 25:3389-3402.
Other reports: Search Summary [Taxonomy reports] [Distance tree of results]

Search Parameters

Program	blastn
Word size	7
Expect value	10
Hitlist size	100
Match/Mismatch scores	2,-3
Gapcosts	5,2
Low Complexity Filter	Yes
Filter string	L;m;
Genetic Code	1

Database

Posted date	Oct 16, 2009 5:41 PM
Number of letters	24,032,946,866
Number of sequences	143,532
Entrez query	none

Karlin-Altschul statistics

Params	Ungapped	Gapped
Lambda	0.633731	0.625
K	0.408146	0.41
H	0.912438	0.78

Results Statistics

Length adjustment	34
Effective length of query	167
Effective length of database	24028066778
Effective search space	4012687151926
Effective search space used	4012687151926

Graphic Summary

Distribution of 105 Blast Hits on the Query Sequence

[?]

An overview of the database sequences aligned to the query sequence is shown. The score of each alignment is indicated by one of five different colors, which divides the range of scores into five groups. Multiple alignments on the same database sequence are connected by a striped line. Mousing over a hit sequence causes the definition and score to be shown in the window at the top, clicking on a hit sequence takes the user to the associated alignments. New: This graphic is an overview of database sequences aligned to the query sequence. Alignments are color-coded by score, within one of five score ranges. Multiple alignments on the same database sequence are connected by a dashed line. Mousing over an alignment shows the alignment definition and score in the box at the top. Clicking an alignment displays the alignment detail.

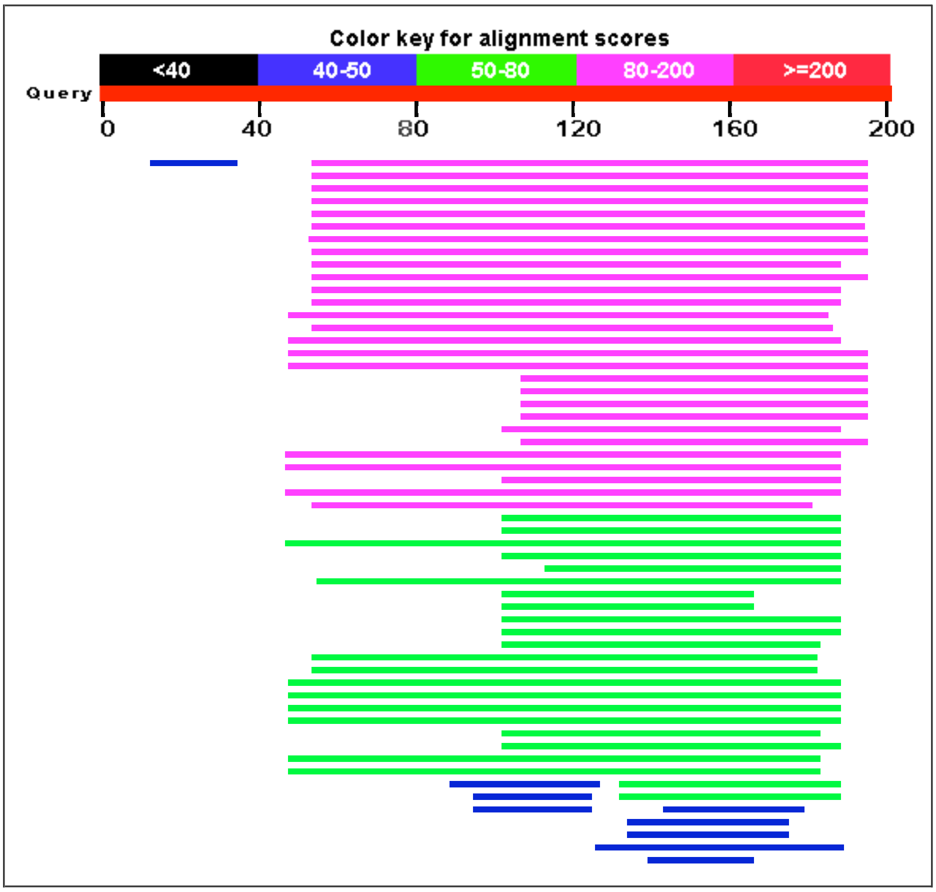

Descriptions

Legend for links to other resources: U UniGene E GEO G Gene S Structure M Map Viewer

Sequences producing significant alignments:
(Click headers to sort columns)

**AC178801.1**	Strongylocentrotus purpuratus clone R3-22F15, WORKING DRAFT SEQUENCE, 31 unordered pieces	118	118	70%	9e-24	79%
**AC178175.1**	Strongylocentrotus purpuratus clone R3-3082B11, WORKING DRAFT SEQUENCE, 36 unordered pieces	118	118	70%	9e-24	79%
**AC177733.1**	Strongylocentrotus purpuratus clone R3-1008K18, WORKING DRAFT SEQUENCE, 19 unordered pieces	118	118	70%	9e-24	79%
**AC179085.1**	Strongylocentrotus purpuratus clone R3-4009J12, WORKING DRAFT SEQUENCE, 29 unordered pieces	114	114	70%	1e-22	78%
**AC181240.1**	Strongylocentrotus purpuratus clone R3-4010K20, WORKING DRAFT SEQUENCE, 35 unordered pieces	113	113	69%	4e-22	78%
**AC181026.1**	Strongylocentrotus purpuratus clone R3-3050M7, WORKING DRAFT SEQUENCE, 26 unordered pieces	113	113	69%	4e-22	78%
**AC184348.1**	Strongylocentrotus purpuratus clone R3-46M7, WORKING DRAFT SEQUENCE, 38 unordered pieces	111	111	70%	1e-21	78%
**AC181800.1**	Strongylocentrotus purpuratus clone R3-1028H12, WORKING DRAFT SEQUENCE, 27 unordered pieces	107	206	70%	2e-20	78%
**AC179917.1**	Strongylocentrotus purpuratus clone R3-1003C21, WORKING DRAFT SEQUENCE, 18 unordered pieces	102	102	66%	7e-19	77%
**AC177991.1**	Strongylocentrotus purpuratus clone R3-3051L15, WORKING DRAFT SEQUENCE, 24 unordered pieces	102	102	70%	7e-19	77%
**AC177193.1**	Strongylocentrotus purpuratus clone R3-1034P02, WORKING DRAFT SEQUENCE, 31 unordered pieces	102	102	66%	7e-19	78%
**AC176372.1**	Strongylocentrotus purpuratus clone R3-3048J17, WORKING DRAFT SEQUENCE, 24 unordered pieces	102	102	66%	7e-19	77%
**AC184353.1**	Strongylocentrotus purpuratus clone R3-3115G18, WORKING DRAFT SEQUENCE, 15 unordered pieces	98.7	98.7	68%	8e-18	76%
**AC180305.1**	Strongylocentrotus purpuratus clone R3-3119N05, WORKING DRAFT SEQUENCE, 23 unordered pieces	98.7	98.7	65%	8e-18	77%
**AC177064.1**	Strongylocentrotus purpuratus clone R3-4004H24, WORKING DRAFT SEQUENCE, 23 unordered pieces	98.7	188	69%	8e-18	76%
**AC184535.1**	Strongylocentrotus purpuratus clone R3-3110O22, WORKING DRAFT SEQUENCE, 22 unordered pieces	96.9	96.9	73%	3e-17	75%
**AC180658.1**	Strongylocentrotus purpuratus clone R3-3089E7, WORKING DRAFT SEQUENCE, 20 unordered pieces	96.9	96.9	73%	3e-17	75%
**AC168761.2**	Strongylocentrotus purpuratus clone R3-3004F17, WORKING DRAFT SEQUENCE, 63 unordered pieces	91.5	91.5	43%	1e-15	82%
**AC168503.2**	Strongylocentrotus purpuratus clone R3-3117B4, WORKING DRAFT SEQUENCE, 28 unordered pieces	91.5	91.5	43%	1e-15	82%
**AC179157.1**	Strongylocentrotus purpuratus clone R3-4012P17, WORKING DRAFT SEQUENCE, 22 unordered pieces	91.5	91.5	43%	1e-15	82%
**AC178911.1**	Strongylocentrotus purpuratus clone R3-3115A3, WORKING DRAFT SEQUENCE, 29 unordered pieces	91.5	91.5	43%	1e-15	82%
**AC168600.2**	Strongylocentrotus purpuratus clone R3-3046N4, WORKING DRAFT SEQUENCE, 19 unordered pieces	87.8	87.8	42%	1e-14	82%
**AC168563.2**	Strongylocentrotus purpuratus clone R3-3091B10, WORKING DRAFT SEQUENCE, 43 unordered pieces	87.8	87.8	43%	1e-14	81%
**AC179536.1**	Strongylocentrotus purpuratus clone R3-6I5, WORKING DRAFT SEQUENCE, 30 unordered pieces	87.8	87.8	70%	1e-14	73%
**AC178562.1**	Strongylocentrotus purpuratus clone R3-3025J10, WORKING DRAFT SEQUENCE, 14 unordered pieces	87.8	87.8	70%	1e-14	73%

**AC178103.1**	Strongylocentrotus purpuratus clone R3-3073B9, WORKING DRAFT SEQUENCE, 19 unordered pieces	87.8	87.8	42%	1e-14	82%
**AC176885.1**	Strongylocentrotus purpuratus clone R3-1110C3, WORKING DRAFT SEQUENCE, 12 unordered pieces	87.8	166	70%	1e-14	73%
**AC174018.2**	Strongylocentrotus purpuratus clone R3-1040A23, WORKING DRAFT SEQUENCE, 13 unordered pieces	86.0	86.0	63%	5e-14	75%
**AC174801.2**	Strongylocentrotus purpuratus clone R3-17H5, WORKING DRAFT SEQUENCE, 9 unordered pieces	78.8	78.8	42%	7e-12	80%
**AC180809.1**	Strongylocentrotus purpuratus clone R3-3071J15, WORKING DRAFT SEQUENCE, 12 unordered pieces	78.8	78.8	42%	7e-12	80%
**AC179624.1**	Strongylocentrotus purpuratus clone R3-1028F05, WORKING DRAFT SEQUENCE, 10 unordered pieces	78.8	78.8	70%	7e-12	72%
**AC176193.1**	Strongylocentrotus purpuratus clone R3-3064A03, WORKING DRAFT SEQUENCE, 13 unordered pieces	78.8	78.8	42%	7e-12	80%
**AC181785.1**	Strongylocentrotus purpuratus clone R3-1030O21, WORKING DRAFT SEQUENCE, 18 unordered pieces	77.0	77.0	37%	3e-11	82%
**AC181382.1**	Strongylocentrotus purpuratus clone R3-1114M19, WORKING DRAFT SEQUENCE, 14 unordered pieces	77.0	77.0	66%	3e-11	74%
**AC168645.2**	Strongylocentrotus purpuratus clone R3-3017K16, WORKING DRAFT SEQUENCE, 24 unordered pieces	75.2	75.2	31%	9e-11	85%
**AC180087.1**	Strongylocentrotus purpuratus clone R3-4008L11, WORKING DRAFT SEQUENCE, 28 unordered pieces	75.2	75.2	31%	9e-11	85%
**AC178239.1**	Strongylocentrotus purpuratus clone R3-3086F16, WORKING DRAFT SEQUENCE, 20 unordered pieces	75.2	75.2	42%	9e-11	79%
**AC201443.1**	Strongylocentrotus purpuratus clone R3-3022G16, WORKING DRAFT SEQUENCE, 29 unordered pieces	66.2	66.2	42%	5e-08	77%
**AC179225.1**	Strongylocentrotus purpuratus clone R3-41F23, WORKING DRAFT SEQUENCE, 23 unordered pieces	66.2	66.2	40%	5e-08	77%
**AC177677.1**	Strongylocentrotus purpuratus clone R3-1017E19, WORKING DRAFT SEQUENCE, 27 unordered pieces	66.2	66.2	63%	5e-08	73%
**AC177168.1**	Strongylocentrotus purpuratus clone R3-4015N17, WORKING DRAFT SEQUENCE, 18 unordered pieces	66.2	66.2	63%	5e-08	73%
**AC149918.2**	Strongylocentrotus purpuratus clone R3-14A13, WORKING DRAFT SEQUENCE, 11 unordered pieces	64.4	64.4	69%	2e-07	71%
**AC180918.1**	Strongylocentrotus purpuratus clone R3-3063E24, WORKING DRAFT SEQUENCE, 16 unordered pieces	64.4	64.4	69%	2e-07	71%
**AC180392.1**	Strongylocentrotus purpuratus clone R3-3106E8, WORKING DRAFT SEQUENCE, 13 unordered pieces	64.4	64.4	69%	2e-07	71%
**AC173487.2**	Strongylocentrotus purpuratus clone R3-3025A17, WORKING DRAFT SEQUENCE, 27 unordered pieces	64.4	64.4	69%	2e-07	71%
**AC180780.1**	Strongylocentrotus purpuratus clone R3-3076F4, WORKING DRAFT SEQUENCE, 17 unordered pieces	60.8	60.8	40%	2e-06	76%
**AC177669.1**	Strongylocentrotus purpuratus clone R3-1017H4, WORKING DRAFT SEQUENCE, 8 unordered pieces	60.8	60.8	42%	2e-06	75%
**AC180771.1**	Strongylocentrotus purpuratus clone R3-3073N16, WORKING DRAFT SEQUENCE, 27 unordered pieces	55.4	55.4	67%	9e-05	70%
**AC178759.1**	Strongylocentrotus purpuratus clone R3-19C07, WORKING DRAFT SEQUENCE, 16 unordered pieces	55.4	55.4	67%	9e-05	70%
**AC174871.2**	Strongylocentrotus purpuratus clone R3-3112P21, WORKING DRAFT SEQUENCE, 9 unordered pieces	51.8	51.8	27%	0.001	80%
**AC178982.1**	Strongylocentrotus purpuratus clone R3-3122I22, WORKING DRAFT SEQUENCE, 12 unordered pieces	51.8	51.8	27%	0.001	80%
**AC211231.4**	Zea mays chromosome 6 clone CH201-465L2; ZMMBBc0465L02, *** SEQUENCING IN PROGRESS ***, 19 unordered pieces	48.2	48.2	17%	0.013	88%

**AC203468.2**	Echinops telfairi clone CH238-500M2, WORKING DRAFT SEQUENCE, 4 ordered pieces	48.2	48.2	20%	0.013	85%
**AC195424.2**	Echinops telfairi clone CH238-291E4, WORKING DRAFT SEQUENCE, 3 ordered pieces	48.2	48.2	20%	0.013	85%
**AC206128.4**	Saccoglossus kowalevskii clone CUGI_SK_BA 001B19, WORKING DRAFT SEQUENCE, 2 unordered pieces	46.4	46.4	31%	0.044	76%
**AC183238.2**	Bos taurus clone CH240-97P5, WORKING DRAFT SEQUENCE, 4 unordered pieces	44.6	44.6	13%	0.15	96%
**AC159723.3**	Bos taurus clone CH240-79H4, WORKING DRAFT SEQUENCE, 8 unordered pieces	44.6	44.6	19%	0.15	84%
**AP008007.2**	Lotus japonicus chromosome 3 clone LjB03J23, *** SEQUENCING IN PROGRESS ***, 20 unordered pieces	44.6	44.6	23%	0.15	80%
**AC159735.3**	Bos taurus clone CH240-35L21, WORKING DRAFT SEQUENCE	44.6	44.6	23%	0.15	82%
**AC181518.1**	Strongylocentrotus purpuratus clone R3-62E1, WORKING DRAFT SEQUENCE, 28 unordered pieces	44.6	44.6	23%	0.15	80%
**AC180090.1**	Strongylocentrotus purpuratus clone R3-3001I05, WORKING DRAFT SEQUENCE, 31 unordered pieces	44.6	44.6	19%	0.15	84%
**AC162133.2**	Loxodonta africana clone VMRC15-344J20, WORKING DRAFT SEQUENCE, 6 ordered pieces	44.6	85.5	20%	0.15	90%
**AC176910.1**	Strongylocentrotus purpuratus clone R3-1104N16, WORKING DRAFT SEQUENCE, 15 unordered pieces	44.6	44.6	19%	0.15	84%
**AC164836.2**	Bos taurus clone CH240-140B23, *** SEQUENCING IN PROGRESS ***, 22 unordered pieces	44.6	44.6	19%	0.15	84%
**AC164913.2**	Bos taurus clone CH240-155C17, *** SEQUENCING IN PROGRESS ***, 7 unordered pieces	44.6	44.6	23%	0.15	82%
**FP312800.2**	Sus scrofa chromosome 6 clone CH242-341I3, WORKING DRAFT SEQUENCE	42.8	42.8	18%	0.54	84%
**AC134160.3**	Rattus norvegicus clone CH230-93N17, WORKING DRAFT SEQUENCE, 2 ordered pieces	42.8	42.8	22%	0.54	80%
**CU856279.2**	Sus scrofa chromosome 15 clone CH242-212D4, WORKING DRAFT SEQUENCE, 11 unordered pieces	42.8	42.8	12%	0.54	96%
**AC194836.3**	Zea mays chromosome 10 clone CH201-479A12; ZMMBBc0479A12, *** SEQUENCING IN PROGRESS ***, 18 unordered pieces	42.8	42.8	18%	0.54	87%
**AC199408.4**	Zea mays chromosome 10 clone CH201-196G4; ZMMBBc0196G04, *** SEQUENCING IN PROGRESS ***, 8 unordered pieces	42.8	42.8	18%	0.54	87%
**AC230641.1**	Bos taurus clone CH240-502O9, WORKING DRAFT SEQUENCE, 8 unordered pieces	42.8	42.8	13%	0.54	92%
**AC220510.1**	Bos taurus clone CH240-355P4, WORKING DRAFT SEQUENCE, 11 unordered pieces	42.8	42.8	24%	0.54	80%
**AC219995.1**	Bos taurus clone CH240-316G8, WORKING DRAFT SEQUENCE, 4 unordered pieces	42.8	42.8	13%	0.54	92%
**AC217613.2**	Procavia capensis clone CH280-98O19, WORKING DRAFT SEQUENCE, 9 ordered pieces	42.8	83.7	20%	0.54	82%
**AC216304.2**	Equus caballus clone CH241-277H1, WORKING DRAFT SEQUENCE, 5 ordered pieces	42.8	42.8	20%	0.54	82%
**AC216525.1**	Loxodonta africana clone VMRC15-120J1, WORKING DRAFT SEQUENCE, 3 unordered pieces	42.8	42.8	20%	0.54	82%
**AC213170.2**	Equus caballus clone CH241-385H17, WORKING DRAFT SEQUENCE, 6 ordered pieces	42.8	42.8	20%	0.54	82%
**AC121463.4**	Rattus norvegicus clone CH230-33J4, WORKING DRAFT SEQUENCE, 4 ordered pieces	42.8	42.8	22%	0.54	80%
**AC190377.2**	Loxodonta africana clone VMRC15-393J21, WORKING DRAFT SEQUENCE, 10 ordered pieces	42.8	42.8	20%	0.54	82%
**AC185202.2**	Loxodonta africana clone VMRC15-2I2, WORKING DRAFT SEQUENCE, 9 ordered pieces	42.8	42.8	20%	0.54	82%
**AC183883.2**	Loxodonta africana clone VMRC15-544N16, WORKING DRAFT SEQUENCE, 8	42.8	42.8	20%	0.54	82%

	ordered pieces					
**AC168586.2**	Strongylocentrotus purpuratus clone R3-3065F16, WORKING DRAFT SEQUENCE, 25 unordered pieces	42.8	42.8	26%	0.54	77%
**AC168548.2**	Strongylocentrotus purpuratus clone R3-3091I21, WORKING DRAFT SEQUENCE, 32 unordered pieces	42.8	42.8	21%	0.54	81%
**AC128025.2**	Rattus norvegicus clone CH230-124G14, *** SEQUENCING IN PROGRESS ***	42.8	42.8	16%	0.54	87%
**AC023199.2**	Homo sapiens chromosome 7 clone RP11-33L7 map 7, WORKING DRAFT SEQUENCE, 11 unordered pieces	42.8	42.8	15%	0.54	90%
**AC097013.7**	Schistosoma mansoni chromosome 0 clone Sm1-48C10, *** SEQUENCING IN PROGRESS ***, 5 unordered pieces	42.8	42.8	32%	0.54	74%
**AC236086.3**	Chlorocebus aethiops clone CH252-214P17, WORKING DRAFT SEQUENCE, 6 ordered pieces	41.0	41.0	25%	1.9	76%
**AC212779.3**	Zea mays chromosome 2 clone CH201-110I6; ZMMBBc0110I06, *** SEQUENCING IN PROGRESS ***, 5 unordered pieces	41.0	41.0	23%	1.9	81%
**AC231323.2**	Loxodonta africana clone VMRC15-463J4, WORKING DRAFT SEQUENCE, 11 ordered pieces	41.0	41.0	20%	1.9	80%
**EU875592.1**	Branchiostoma floridae clone Bac 100J9, *** SEQUENCING IN PROGRESS ***, 2 ordered pieces	41.0	41.0	27%	1.9	76%
**AC149662.4**	Bos taurus clone CH240-66A10, WORKING DRAFT SEQUENCE, 8 unordered pieces	41.0	41.0	10%	1.9	100%
**AC230587.1**	Bos taurus clone CH240-504C12, WORKING DRAFT SEQUENCE, 8 unordered pieces	41.0	41.0	14%	1.9	90%
**AP010078.1**	Lotus japonicus clone LjT33M20, *** SEQUENCING IN PROGRESS ***, 24 unordered pieces	41.0	41.0	19%	1.9	82%
**AC225966.1**	Loxodonta africana clone VMRC15-206J12, WORKING DRAFT SEQUENCE, 3 unordered pieces	41.0	41.0	20%	1.9	80%
**CU633680.2**	Sus scrofa chromosome 13 clone CH242-251F9, WORKING DRAFT SEQUENCE	41.0	41.0	10%	1.9	100%
**AC217228.3**	Procavia capensis clone CH280-20O24, WORKING DRAFT SEQUENCE, 4 ordered pieces	41.0	41.0	20%	1.9	80%
**AC214731.3**	Procavia capensis clone CH280-198C7, WORKING DRAFT SEQUENCE, 6 ordered pieces	41.0	41.0	17%	1.9	85%
**AC215573.2**	Procavia capensis clone CH280-21J17, WORKING DRAFT SEQUENCE, 4 ordered pieces	41.0	41.0	20%	1.9	80%
**AC162188.4**	Bos taurus clone CH240-116P1, WORKING DRAFT SEQUENCE, 9 unordered pieces	41.0	41.0	14%	1.9	90%
**AC196583.6**	Macaca mulatta clone CH250-66M21, WORKING DRAFT SEQUENCE, 4 ordered pieces	41.0	41.0	26%	1.9	77%

Alignments Select All Get selected sequences Distance tree of results Multiple alignment NEW

```
>gb|AC178801.1| D Strongylocentrotus purpuratus clone R3-22F15, WORKING DRAFT SEQUENCE,
31 unordered pieces
Length=165463

 Score =  118 bits (130),  Expect = 9e-24
 Identities = 113/142 (79%), Gaps = 2/142 (1%)
 Strand=Plus/Plus

Query  55    AGAAGACGCTGGAGGTGGATTCGGCATGTCCTACGGAAAGGCCAG-GGGGATATCACAAA  113
             |||||| | ||||| |||||  |||||||  |    |||||| ||| |  || |||||| |
Sbjct  8346  AGAAGAAGGTGGAGATGGATCGGGCATGTAGT-GAGAAAGGACAGAGATGACATCACACA  8404

Query  114   AACAGCACTCCACTGGACCCCAGAAGGTAAAAGAAAGAGAGGAAGACCTAAAATGACATG  173
             |||||| ||||||||||| |||||||| ||||||||||| ||| | |||||||  || ||
Sbjct  8405  AACAGCCCTCCACTGGACACCAGAAGGCAAAAGAAAGAGGGGACGTCCTAAAACCACTTG  8464

Query  174   GAGAAGAACTGTAGAGGCAGAG  195
             | |||||||||| || | ||||
Sbjct  8465  GCGAAGAACTGTGGAAGGAGAG  8486


>gb|AC178175.1| D Strongylocentrotus purpuratus clone R3-3082B11, WORKING DRAFT
SEQUENCE, 36 unordered pieces
Length=153471

 Score =  118 bits (130),  Expect = 9e-24
 Identities = 113/142 (79%), Gaps = 2/142 (1%)
 Strand=Plus/Plus

Query  55     AGAAGACGCTGGAGGTGGATTCGGCATGTCCTACGGAAAGGCCAG-GGGGATATCACAAA  113
              |||||| | ||||| |||||  |||||||  |    |||||| ||| |  || |||||| |
Sbjct  72530  AGAAGAAGGTGGAGATGGATCGGGCATGTAGT-GAGAAAGGACAGAGATGACATCACACA  72588

Query  114    AACAGCACTCCACTGGACCCCAGAAGGTAAAAGAAAGAGAGGAAGACCTAAAATGACATG  173
              |||||| ||||||||||| |||||||| ||||||||||| ||| | |||||||  || ||
Sbjct  72589  AACAGCCCTCCACTGGACACCAGAAGGCAAAAGAAAGAGGGGACGTCCTAAAACCACTTG  72648

Query  174    GAGAAGAACTGTAGAGGCAGAG  195
              | |||||||||| || | ||||
Sbjct  72649  GCGAAGAACTGTGGAAGGAGAG  72670


>gb|AC177733.1| D Strongylocentrotus purpuratus clone R3-1008K18, WORKING DRAFT
SEQUENCE, 19 unordered pieces
Length=114730

 Score =  118 bits (130),  Expect = 9e-24
 Identities = 113/142 (79%), Gaps = 2/142 (1%)
 Strand=Plus/Minus

Query  55     AGAAGACGCTGGAGGTGGATTCGGCATGTCCTACGGAAAGGCCAGGGG-GATATCACAAA  113
              |||||| | ||||| |||||  |||||||  |  | ||||| ||| |  || |||||| |
Sbjct  44912  AGAAGAAGGTGGAGATGGATCGGGCATGTAGTGAG-AAAGGACAGAGATGACATCACACA  44854

Query  114    AACAGCACTCCACTGGACCCCAGAAGGTAAAAGAAAGAGAGGAAGACCTAAAATGACATG  173
              |||||| ||||||||||| |||||||| ||||||||||| ||| | |||||||  || ||
Sbjct  44853  AACAGCCCTCCACTGGACACCAGAAGGCAAAAGAAAGAGGGGACGTCCTAAAACCACTTG  44794

Query  174    GAGAAGAACTGTAGAGGCAGAG  195
              | |||||||||| || | ||||
Sbjct  44793  GCGAAGAACTGTGGAAGGAGAG  44772


>gb|AC179085.1| D Strongylocentrotus purpuratus clone R3-4009J12, WORKING DRAFT
SEQUENCE, 29 unordered pieces
Length=222950

 Score =  114 bits (126),  Expect = 1e-22
 Identities = 112/142 (78%), Gaps = 2/142 (1%)
 Strand=Plus/Plus

Query  55      AGAAGACGCTGGAGGTGGATTCGGCATGTCCTACGGAAAGGCCAG-GGGGATATCACAAA  113
               |||||| | ||||| |||||  |||||||  |    |||||| ||| |  || ||||||
Sbjct  198457  AGAAGAAGGTGGAGATGGATCGGGCATGTAAT-GAGAAAGGACAGAGATGACATCACACG  198515

Query  114     AACAGCACTCCACTGGACCCCAGAAGGTAAAAGAAAGAGAGGAAGACCTAAAATGACATG  173
               |||||| ||||||||||| |||||||| ||||||||||| ||| | |||||||  || ||
Sbjct  198516  AACAGCCCTCCACTGGACACCAGAAGGCAAAAGAAAGAGGGGACGTCCTAAAACCACTTG  198575

Query  174     GAGAAGAACTGTAGAGGCAGAG  195
               | |||||||||| || | ||||
Sbjct  198576  GCGAAGAACTGTGGAAGGAGAG  198597


>gb|AC181240.1| D Strongylocentrotus purpuratus clone R3-4010K20, WORKING DRAFT
SEQUENCE, 35 unordered pieces
Length=191934
```

```
 Score =  113 bits (124),  Expect = 4e-22
 Identities = 111/141 (78%), Gaps = 2/141 (1%)
 Strand=Plus/Minus

Query  55    AGAAGACGCTGGAGGTGGATTCGGCATGTCCTACGGAAAGGCCAGGGG-GATATCACAAA  113
             |||||| | ||||| |||||  |||||||  |  | ||||| ||| |  || ||||||
Sbjct  3153  AGAAGAAGGTGGAGATGGATCGGGCATGTAATGAG-AAAGGACAGAGATGACATCACACG  3095

Query  114   AACAGCACTCCACTGGACCCCAGAAGGTAAAAGAAAGAGAGGAAGACCTAAAATGACATG  173
             |||||| ||||||||||| |||||||| ||||||||||| ||| | |||||||  || ||
Sbjct  3094  AACAGCCCTCCACTGGACACCAGAAGGCAAAAGAAAGAGGGGACGTCCTAAAACCACTTG  3035

Query  174   GAGAAGAACTGTAGAGGCAGA  194
             | |||||||||| || | |||
Sbjct  3034  GCGAAGAACTGTGGAAGGAGA  3014
```

>**gb|AC181026.1|** Strongylocentrotus purpuratus clone R3-3050M7, WORKING DRAFT SEQUENCE, 26 unordered pieces
Length=142237

```
 Score =  113 bits (124),  Expect = 4e-22
 Identities = 111/141 (78%), Gaps = 2/141 (1%)
 Strand=Plus/Minus

Query  55      AGAAGACGCTGGAGGTGGATTCGGCATGTCCTACGGAAAGGCCAGGGG-GATATCACAAA  113
               |||||| | ||||| |||||  |||||||  |  | ||||| ||| |  || ||||||
Sbjct  131673  AGAAGAAGGTGGAGATGGATCGGGCATGTAATGAG-AAAGGACAGAGATGACATCACACG  131615

Query  114     AACAGCACTCCACTGGACCCCAGAAGGTAAAAGAAAGAGAGGAAGACCTAAAATGACATG  173
               |||||| ||||||||||| |||||||| ||||||||||| ||| | |||||||  || ||
Sbjct  131614  AACAGCCCTCCACTGGACACCAGAAGGCAAAAGAAAGAGGGGACGTCCTAAAACCACTTG  131555

Query  174     GAGAAGAACTGTAGAGGCAGA  194
               | |||||||||| || | |||
Sbjct  131554  GCGAAGAACTGTGGAAGGAGA  131534
```

>**gb|AC184348.1|** Strongylocentrotus purpuratus clone R3-46M7, WORKING DRAFT SEQUENCE, 38 unordered pieces
Length=160379

```
 Score =  111 bits (122),  Expect = 1e-21
 Identities = 112/143 (78%), Gaps = 2/143 (1%)
 Strand=Plus/Plus

Query  54     AAGAAGACGCTGGAGGTGGATTCGGCATGTCCTACGGAAAGGCCAG-GGGGATATCACAA  112
              ||||||| | ||||| |||||  |||||||  |    |||||| ||| |  || |||| |
Sbjct  26769  AAGAAGAAGGTGGAGATGGATCGGGCATGTAAT-GAGAAAGGACAGAGATGACATCATAC  26827

Query  113    AAACAGCACTCCACTGGACCCCAGAAGGTAAAAGAAAGAGAGGAAGACCTAAAATGACAT  172
               |||||| ||||||||||| |||||||| ||||||||||| ||| | |||||||  || |
Sbjct  26828  GAACAGCCCTCCACTGGACACCAGAAGGCAAAAGAAAGAGGGGACGTCCTAAAACCACTT  26887

Query  173    GGAGAAGAACTGTAGAGGCAGAG  195
              || |||||||||| || | ||||
Sbjct  26888  GGCGAAGAACTGTGGAAGGAGAG  26910
```

>**gb|AC181800.1|** Strongylocentrotus purpuratus clone R3-1028H12, WORKING DRAFT SEQUENCE, 27 unordered pieces
Length=129213

Sort alignments for this subject sequence by:
E value **Score** **Percent identity**
Query start position **Subject start position**

```
 Score =  107 bits (118),  Expect = 2e-20
 Identities = 112/143 (78%), Gaps = 3/143 (2%)
 Strand=Plus/Plus

Query  55     AGAAGACGCTGGAGGTGGATTCGGCATGTCCTACGGAAAGGCCAG-GGGGATATCACAAA  113
              |||||| | ||||| |||||  |||||||  |    |||||| ||| |  || ||||||
Sbjct  19678  AGAAGAAGGTGGAGATGGATCTGGCATGTAAT-GAGAAAGGACAGAGATGACATCACACG  19736

Query  114    AACAGCACTCCACTGGACCCCAGAAGGTAAAAGAAAGAGAGGAAGACCT-AAAATGACAT  172
              |||||| ||||||||||| |||||||| ||||||||||| ||| | ||| ||||  || |
Sbjct  19737  AACAGCCCTCCACTGGACACCAGAAGGCAAAAGAAAGAGGGGACGTCCTAAAAACCACTT  19796

Query  173    GGAGAAGAACTGTAGAGGCAGAG  195
              || |||||||||| || | ||||
Sbjct  19797  GGCGAAGAACTGTGGAAGGAGAG  19819


 Score = 98.7 bits (108),  Expect = 8e-18
 Identities = 105/135 (77%), Gaps = 3/135 (2%)
 Strand=Plus/Plus

Query  55      AGAAGACGCTGGAGGTGGATTCGGCATGTCCTACGGAAAGGCCAGGGG-GATATCACAAA  113
               |||||| | ||||| |||||  |||||||  |  | ||||  ||| |  || |||| |
Sbjct  125366  AGAAGAAGGTGGAGATGGATCGGGCATGTAATGAG-AAAGAACAGAGATGACATCATACG  125424

Query  114     AACAGCACTCCACTGGACCCCAGAAGGTAAAAGAAAGAGAGGAAGACCTAAAATGACATG  173
               |||||| ||||||||||||| |||||| ||||||||||| ||| | |||||||  || ||
Sbjct  125425  AACAGCCCTCCACTGGACCC-AGAAGGCAAAAGAAAGAGGGGACGTCCTAAAACCACTTG  125483

Query  174     GAGAAGAACTGTAGA  188
               | |||||||||| ||
Sbjct  125484  GCGAAGAACTGTGGA  125498
```

>**gb|AC179917.1|** Strongylocentrotus purpuratus clone R3-1003C21, WORKING DRAFT SEQUENCE, 18 unordered pieces
Length=149824

```
 Score =  102 bits (112),  Expect = 7e-19
```

```
 Identities = 105/135 (77%), Gaps = 2/135 (1%)
 Strand=Plus/Plus

Query  55     AGAAGACGCTGGAGGTGGATTCGGCATGTCCTACGGAAAGGCCAGGGG-GATATCACAAA  113
              |||||| | ||||| |||||  |||||||  |  | ||||  ||| |  || ||||||
Sbjct  41807  AGAAGAAGGTGGAGATGGATCGGGCATGTAATGAG-AAAGAACAGAGATGACATCACACG  41865

Query  114    AACAGCACTCCACTGGACCCCAGAAGGTAAAAGAAAGAGAGGAAGACCTAAAATGACATG  173
              |||||| ||||||||||| |||||||| ||||||||||| ||| | |||||||  || ||
Sbjct  41866  AACAGCCCTCCACTGGACACCAGAAGGCAAAAGAAAGAGGGGACGTCCTAAAACCACTTG  41925

Query  174    GAGAAGAACTGTAGA  188
                |||||||||| ||
Sbjct  41926  ACGAAGAACTGTGGA  41940
```

>**gb|AC177991.1|** D Strongylocentrotus purpuratus clone R3-3051L15, WORKING DRAFT SEQUENCE, 24 unordered pieces
Length=164932

```
 Score =  102 bits (112),  Expect = 7e-19
 Identities = 111/144 (77%), Gaps = 4/144 (2%)
 Strand=Plus/Minus

Query  55     AGAAGACGCTGGAGGTGGATTCGGCATGTCCTACGGAAAGGCCAGGGG-GATATCACAAA  113
              |||||| | ||||| |||||  |||||||  |  | ||||| ||| |  || ||||||
Sbjct  77113  AGAAGAAGGTGGAGATGGATCGGGCATGTAATGAG-AAAGGACAGAGATGACATCACACG  77055

Query  114    AACAGCACTCCACTGGACCCCAGAAGG--TAAAAGAAAGAGAGGAAGACCTAAAATGACA  171
              |||||| ||||||||||| ||||||||    |||| |||||| ||| | |||||||  ||
Sbjct  77054  AACAGCCCTCCACTGGACACCAGAAGGCAAAAAAAAAAGAGGGGACGTCCTAAAACCACT  76995

Query  172    TGGAGAAGAACTGTAGAGGCAGAG  195
              ||| |||||||||| || | ||||
Sbjct  76994  TGGCGAAGAACTGTGGAAGGAGAG  76971
```

>**gb|AC177193.1|** D Strongylocentrotus purpuratus clone R3-1034P02, WORKING DRAFT SEQUENCE, 31 unordered pieces
Length=151241

```
 Score =  102 bits (112),  Expect = 7e-19
 Identities = 106/135 (78%), Gaps = 3/135 (2%)
 Strand=Plus/Plus

Query  55     AGAAGACGCTGGAGGTGGATTCGGCATGTCCTACGGAAAGGCCAGGGG-GATATCACAAA  113
              |||||| | ||||| |||||  |||||||  |  | ||||  ||| |  || ||||||
Sbjct  77338  AGAAGAAGGTGGAGATGGATCGGGCATGTAATGAG-AAAGAACAGAGATGACATCACACG  77396

Query  114    AACAGCACTCCACTGGACCCCAGAAGGTAAAAGAAAGAGAGGAAGACCTAAAATGACATG  173
              |||||| ||||||||||||| |||||| ||||||||||| ||| | |||||||  || ||
Sbjct  77397  AACAGCCCTCCACTGGACCC-AGAAGGCAAAAGAAAGAGGGGACGTCCTAAAACCACTTG  77455

Query  174    GAGAAGAACTGTAGA  188
              | |||||||||| ||
Sbjct  77456  GCGAAGAACTGTGGA  77470
```

>**gb|AC176372.1|** D Strongylocentrotus purpuratus clone R3-3048J17, WORKING DRAFT SEQUENCE, 24 unordered pieces
Length=153054

```
 Score =  102 bits (112),  Expect = 7e-19
 Identities = 105/135 (77%), Gaps = 2/135 (1%)
 Strand=Plus/Plus

Query  55     AGAAGACGCTGGAGGTGGATTCGGCATGTCCTACGGAAAGGCCAGGGG-GATATCACAAA  113
              |||||| | ||||| |||||  |||||||  |  | ||||  ||| |  || ||||||
Sbjct  26952  AGAAGAAGGTGGAGATGGATCGGGCATGTAATGAG-AAAGAACAGAGATGACATCACACG  27010

Query  114    AACAGCACTCCACTGGACCCCAGAAGGTAAAAGAAAGAGAGGAAGACCTAAAATGACATG  173
              |||||| ||||||||||| |||||||| ||||||||||| ||| | |||||||  || ||
Sbjct  27011  AACAGCCCTCCACTGGACACCAGAAGGCAAAAGAAAGAGGGGACGTCCTAAAACCACTTG  27070

Query  174    GAGAAGAACTGTAGA  188
                |||||||||| ||
Sbjct  27071  ACGAAGAACTGTGGA  27085
```

>**gb|AC184353.1|** D Strongylocentrotus purpuratus clone R3-3115G18, WORKING DRAFT SEQUENCE, 15 unordered pieces
Length=115447

```
 Score = 98.7 bits (108),  Expect = 8e-18
 Identities = 106/138 (76%), Gaps = 2/138 (1%)
 Strand=Plus/Plus

Query  49      ATAATAAGAAGACGCTGGAGGTGGATTCGGCATGTCCTACGGAAAGGCCAGGGG-GATAT  107
               || || |||||| | ||||| |||||  | |||||  |  | ||||  ||  |  || ||
Sbjct  100199  ATCATGAGAAGAAGGTGGAGATGGATCGGACATGTAATGAG-AAAGAACATAGATGACAT  100257

Query  108     CACAAAAACAGCACTCCACTGGACCCCAGAAGGTAAAAGAAAGAGAGGAAGACCTAAAAT  167
               ||||  |||||| ||||||||||| |||||||| ||||||||||| ||| | |||||||
Sbjct  100258  CACATGAACAGCCCTCCACTGGACACCAGAAGGCAAAAGAAAGAGGGGACGTCCTAAAAA  100317

Query  168     GACATGGAGAAGAACTGT  185
                || ||| ||||||||||
Sbjct  100318  CACTTGGCGAAGAACTGT  100335
```

>**gb|AC180305.1|** D Strongylocentrotus purpuratus clone R3-3119N05, WORKING DRAFT SEQUENCE, 23 unordered pieces
Length=144155

```
 Score = 98.7 bits (108),  Expect = 8e-18
 Identities = 103/133 (77%), Gaps = 2/133 (1%)
 Strand=Plus/Plus
```

```
Query  55     AGAAGACGCTGGAGGTGGATTCGGCATGTCCTACGGAAAGGCCAGGGG-GATATCACAAA  113
              |||||| | ||||| |||||  |||||||  |  | ||||  ||| |  || ||||||
Sbjct  74410  AGAAGAAGGTGGAGATGGATCGGGCATGTAATGAG-AAAGAACAGAGATGACATCACACG  74468

Query  114    AACAGCACTCCACTGGACCCCAGAAGGTAAAAGAAAGAGAGGAAGACCTAAAATGACATG  173
              |||||| ||||||||||| |||||||| |||||||| || ||| | |||||||  || ||
Sbjct  74469  AACAGCCCTCCACTGGACACCAGAAGGCAAAAGAAAAAGGGGACGTCCTAAAACCACTTG  74528

Query  174    GAGAAGAACTGTA  186
              | ||||||| |||
Sbjct  74529  GCGAAGAACAGTA  74541
```

>**gb|AC177064.1|** Strongylocentrotus purpuratus clone R3-4004H24, WORKING DRAFT
SEQUENCE, 23 unordered pieces
Length=163262

Sort alignments for this subject sequence by:
E value **Score** **Percent identity**
Query start position **Subject start position**

Score = 98.7 bits (108), Expect = 8e-18
Identities = 108/141 (76%), Gaps = 2/141 (1%)
Strand=Plus/Plus

```
Query  49      ATAATAAGAAGACGCTGGAGGTGGATTCGGCATGTCCTACGGAAAGGCCAGGGG-GATAT  107
               || || |||||| | ||||| |||||| | |||||  |  | ||||  ||| |  || ||
Sbjct  152151  ATCATGAGAAGAAGGTGGAGATGGATTGGACATGTAATGAG-AAAGAACAGAGATGACAT  152209

Query  108     CACAAAAACAGCACTCCACTGGACCCCAGAAGGTAAAAGAAAGAGAGGAAGACCTAAAAT  167
               ||||  |||||| ||||||||||| ||| |||| |||||| |||| ||| | |||||||
Sbjct  152210  CACACGAACAGCCCTCCACTGGACACCAAAAGGCAAAAGAGAGAGGGGACGTCCTAAAAA  152269

Query  168     GACATGGAGAAGAACTGTAGA  188
                || ||| |||||||||| ||
Sbjct  152270  CACTTGGCGAAGAACTGTGGA  152290
```

Score = 89.7 bits (98), Expect = 4e-15
Identities = 106/141 (75%), Gaps = 2/141 (1%)
Strand=Plus/Minus

```
Query  49      ATAATAAGAAGACGCTGGAGGTGGATTCGGCATGTCCTACGGAAAGGCCAGGGG-GATAT  107
               || || |||||| | ||||  |||||| | |||||  |  | ||||  ||  |  ||  |
Sbjct  126863  ATCATGAGAAGAAGGTGGATATGGATTGGACATGTAATGAG-AAAGAACATAGATGACGT  126805

Query  108     CACAAAAACAGCACTCCACTGGACCCCAGAAGGTAAAAGAAAGAGAGGAAGACCTAAAAT  167
               ||||  |||||| ||||||||||| ||| |||| ||||||||||| ||| | |||||||
Sbjct  126804  CACACGAACAGCCCTCCACTGGACACCAAAAGGCAAAAGAAAGAGGGGACGTCCTAAAAA  126745

Query  168     GACATGGAGAAGAACTGTAGA  188
                || ||| |||||||||| ||
Sbjct  126744  CACTTGGCGAAGAACTGTGGA  126724
```

>**gb|AC184535.1|** Strongylocentrotus purpuratus clone R3-3110O22, WORKING DRAFT
SEQUENCE, 22 unordered pieces
Length=141799

Score = 96.9 bits (106), Expect = 3e-17
Identities = 111/148 (75%), Gaps = 1/148 (0%)
Strand=Plus/Minus

```
Query  49     ATAATAAGAAGACGCTGGAGGTGGATTCGGCATGTCCTACGGAAAGGCCAGGGG-GATAT  107
              || || |||||| | | ||| |||||  | |||||  |  | |||   ||  |  || ||
Sbjct  71698  ATCATGAGAAGAAGGTCGAGATGGATCGGACATGTATTGAGAAAAAAACATAGATGACAT  71639

Query  108    CACAAAAACAGCACTCCACTGGACCCCAGAAGGTAAAAGAAAGAGAGGAAGACCTAAAAT  167
              ||||  |||||| ||||||||||| |||||||| ||||||||||| ||| | |||||||
Sbjct  71638  CACACGAACAGCCCTCCACTGGACACCAGAAGGCAAAAGAAAGAGGGGACGTCCTAAAAA  71579

Query  168    GACATGGAGAAGAACTGTAGAGGCAGAG  195
               || ||| |||||||||| || | ||||
Sbjct  71578  CACTTGGCGAAGAACTGTGGAAGGAGAG  71551
```

>**gb|AC180658.1|** Strongylocentrotus purpuratus clone R3-3089E7, WORKING DRAFT
SEQUENCE, 20 unordered pieces
Length=149709

Score = 96.9 bits (106), Expect = 3e-17
Identities = 111/148 (75%), Gaps = 1/148 (0%)
Strand=Plus/Plus

```
Query  49      ATAATAAGAAGACGCTGGAGGTGGATTCGGCATGTCCTACGGAAAGGCCAGGGG-GATAT  107
               || || |||||| | | ||| |||||  | |||||  |  | |||   ||  |  || ||
Sbjct  129594  ATCATGAGAAGAAGGTCGAGATGGATCGGACATGTATTGAGAAAAAAACATAGATGACAT  129653

Query  108     CACAAAAACAGCACTCCACTGGACCCCAGAAGGTAAAAGAAAGAGAGGAAGACCTAAAAT  167
               ||||  |||||| ||||||||||| |||||||| ||||||||||| ||| | |||||||
Sbjct  129654  CACACGAACAGCCCTCCACTGGACACCAGAAGGCAAAAGAAAGAGGGGACGTCCTAAAAA  129713

Query  168     GACATGGAGAAGAACTGTAGAGGCAGAG  195
                || ||| |||||||||| || | ||||
Sbjct  129714  CACTTGGCGAAGAACTGTGGAAGGAGAG  129741
```

>**gb|AC168761.2|** Strongylocentrotus purpuratus clone R3-3004F17, WORKING DRAFT
SEQUENCE, 63 unordered pieces
Length=236260

Score = 91.5 bits (100), Expect = 1e-15
Identities = 73/88 (82%), Gaps = 0/88 (0%)
Strand=Plus/Minus

```
Query  108     CACAAAAACAGCACTCCACTGGACCCCAGAAGGTAAAAGAAAGAGAGGAAGACCTAAAAT  167
```

```
                  ||||  |||||| ||||||||||| |||||||| ||||||||||| ||| | |||||||
Sbjct  217084  CACACGAACAGCCCTCCACTGGACACCAGAAGGCAAAAGAAAGAGGGGACGTCCTAAAAC  217025

Query  168     GACATGGAGAAGAACTGTAGAGGCAGAG  195
                || ||| |||||||||| || | ||||
Sbjct  217024  CACTTGGCGAAGAACTGTGGAAGGAGAG  216997


>gb|AC168503.2| D Strongylocentrotus purpuratus clone R3-3117B4, WORKING DRAFT
SEQUENCE, 28 unordered pieces
Length=146426

 Score = 91.5 bits (100),  Expect = 1e-15
 Identities = 73/88 (82%), Gaps = 0/88 (0%)
 Strand=Plus/Plus

Query  108    CACAAAAACAGCACTCCACTGGACCCCAGAAGGTAAAAGAAAGAGAGGAAGACCTAAAAT  167
              ||||  |||||| ||||||||||| |||||||| ||||||||||| ||| | |||||||
Sbjct  56206  CACACGAACAGCCCTCCACTGGACACCAGAAGGCAAAAGAAAGAGGGGACGTCCTAAAAC  56265

Query  168    GACATGGAGAAGAACTGTAGAGGCAGAG  195
               || ||| |||||||||| || | ||||
Sbjct  56266  CACTTGGCGAAGAACTGTGGAAGGAGAG  56293


>gb|AC179157.1| D Strongylocentrotus purpuratus clone R3-4012P17, WORKING DRAFT
SEQUENCE, 22 unordered pieces
Length=133531

 Score = 91.5 bits (100),  Expect = 1e-15
 Identities = 73/88 (82%), Gaps = 0/88 (0%)
 Strand=Plus/Minus

Query  108    CACAAAAACAGCACTCCACTGGACCCCAGAAGGTAAAAGAAAGAGAGGAAGACCTAAAAT  167
              ||||  |||||| ||||||||||| |||||||| ||||||||||| ||| | |||||||
Sbjct  19183  CACACGAACAGCCCTCCACTGGACACCAGAAGGCAAAAGAAAGAGGGGACGTCCTAAAAC  19124

Query  168    GACATGGAGAAGAACTGTAGAGGCAGAG  195
               || ||| |||||||||| || | ||||
Sbjct  19123  CACTTGGCGAAGAACTGTGGAAGGAGAG  19096


>gb|AC178911.1| D Strongylocentrotus purpuratus clone R3-3115A3, WORKING DRAFT
SEQUENCE, 29 unordered pieces
Length=201058

 Score = 91.5 bits (100),  Expect = 1e-15
 Identities = 73/88 (82%), Gaps = 0/88 (0%)
 Strand=Plus/Plus

Query  108     CACAAAAACAGCACTCCACTGGACCCCAGAAGGTAAAAGAAAGAGAGGAAGACCTAAAAT  167
               ||||  |||||| ||||||||||| |||||||| ||||||||||| ||| | |||||||
Sbjct  163760  CACACGAACAGCCCTCCACTGGACACCAGAAGGCAAAAGAAAGAGGGGACGTCCTAAAAC  163819

Query  168     GACATGGAGAAGAACTGTAGAGGCAGAG  195
                || ||| |||||||||| || | ||||
Sbjct  163820  CACTTGGCGAAGAACTGTGGAAGGAGAG  163847


>gb|AC168600.2| D Strongylocentrotus purpuratus clone R3-3046N4, WORKING DRAFT
SEQUENCE, 19 unordered pieces
Length=140829

 Score = 87.8 bits (96),  Expect = 1e-14
 Identities = 71/86 (82%), Gaps = 0/86 (0%)
 Strand=Plus/Minus

Query  103    GATATCACAAAAACAGCACTCCACTGGACCCCAGAAGGTAAAAGAAAGAGAGGAAGACCT  162
              || ||||||  |||||| ||||||||||| |||||||| ||||||||||| ||| | |||
Sbjct  24277  GACATCACACGAACAGCCCTCCACTGGACACCAGAAGGCAAAAGAAAGAGGGGACGTCCT  24218

Query  163    AAAATGACATGGAGAAGAACTGTAGA  188
              ||||  || ||  |||||||||| ||
Sbjct  24217  AAAAACACTTGCCGAAGAACTGTGGA  24192


>gb|AC168563.2| D Strongylocentrotus purpuratus clone R3-3091B10, WORKING DRAFT
SEQUENCE, 43 unordered pieces
Length=263763

 Score = 87.8 bits (96),  Expect = 1e-14
 Identities = 72/88 (81%), Gaps = 0/88 (0%)
 Strand=Plus/Plus

Query  108    CACAAAAACAGCACTCCACTGGACCCCAGAAGGTAAAAGAAAGAGAGGAAGACCTAAAAT  167
              ||||  |||||| ||||||||||| |||||||| ||||||||||| ||| | |||| ||
Sbjct  68309  CACACGAACAGCCCTCCACTGGACACCAGAAGGCAAAAGAAAGAGGGGACGTCCTANAAC  68368

Query  168    GACATGGAGAAGAACTGTAGAGGCAGAG  195
               || ||| |||||||||| || | ||||
Sbjct  68369  CACTTGGCGAAGAACTGTGGAAGGAGAG  68396


>gb|AC179536.1| D Strongylocentrotus purpuratus clone R3-6I5, WORKING DRAFT SEQUENCE,
30 unordered pieces
Length=170469

 Score = 87.8 bits (96),  Expect = 1e-14
 Identities = 104/141 (73%), Gaps = 0/141 (0%)
 Strand=Plus/Minus

Query  48     TATAATAAGAAGACGCTGGAGGTGGATTCGGCATGTCCTACGGAAAGGCCAGGGGATAT  107
              ||| || |||||| | ||||| |||||  | |||||  |  | |||    |   |  ||
Sbjct  74960  TATCATGAGAAGAAGGTGGAGATGGATCGGACATGTAATGAGAAAAACATAGATGGCAT  74901

Query  108    CACAAAAACAGCACTCCACTGGACCCCAGAAGGTAAAAGAAAGAGAGGAAGACCTAAAAT  167
```

```
                ||  |  |||||| ||||||||||| |||||||| ||||||||||| ||| | |||||||
Sbjct  74900  CATACGAACAGCCCTCCACTGGACACCAGAAGGCAAAAGAAAGAGGGGACGTCCTAAAAA  74841

Query  168    GACATGGAGAAGAACTGTAGA  188
               || ||||| |||| ||| ||
Sbjct  74840  CACTTGGAGGAGAAATGTGGA  74820
```

>**gb|AC178562.1|** D Strongylocentrotus purpuratus clone R3-3025J10, WORKING DRAFT SEQUENCE, 14 unordered pieces
Length=162394

Score = 87.8 bits (96), Expect = 1e-14
Identities = 104/141 (73%), Gaps = 0/141 (0%)
Strand=Plus/Plus

```
Query  48     TATAATAAGAAGACGCTGGAGGTGGATTCGGCATGTCCTACGGAAAGGCCAGGGGGATAT  107
              ||| || |||||| | ||||| |||||  | |||||  |  | |||  |   |  |  ||
Sbjct  39215  TATCATGAGAAGAAGGTGGAGATGGATCGGACATGTAATGAGAAAAAACATAGATGGCAT  39274

Query  108    CACAAAAACAGCACTCCACTGGACCCCAGAAGGTAAAAGAAAGAGAGGAAGACCTAAAAT  167
              || |  |||||| ||||||||||| |||||||| ||||||||||| ||| | |||||||
Sbjct  39275  CATACGAACAGCCCTCCACTGGACACCAGAAGGCAAAAGAAAGAGGGGACGTCCTAAAAA  39334

Query  168    GACATGGAGAAGAACTGTAGA  188
               || ||||| |||| ||| ||
Sbjct  39335  CACTTGGAGGAGAAATGTGGA  39355
```

>**gb|AC178103.1|** D Strongylocentrotus purpuratus clone R3-3073B9, WORKING DRAFT SEQUENCE, 19 unordered pieces
Length=196567

Score = 87.8 bits (96), Expect = 1e-14
Identities = 71/86 (82%), Gaps = 0/86 (0%)
Strand=Plus/Plus

```
Query  103    GATATCACAAAAACAGCACTCCACTGGACCCCAGAAGGTAAAAGAAAGAGAGGAAGACCT  162
              || ||||||  |||||| ||||| ||||| |||||||| ||||||||||| ||| | |||
Sbjct  46335  GACATCACACGAACAGCCCTCCATTGGACACCAGAAGGCAAAAGAAAGAGGGGACGTCCT  46394

Query  163    AAAATGACATGGAGAAGAACTGTAGA  188
              ||||  || ||| |||||||||| ||
Sbjct  46395  AAAAACACTTGGCGAAGAACTGTGGA  46420
```

>**gb|AC176885.1|** D Strongylocentrotus purpuratus clone R3-1110C3, WORKING DRAFT SEQUENCE, 12 unordered pieces
Length=160781

Sort alignments for this subject sequence by:
E value **Score** **Percent identity**
Query start position **Subject start position**

Score = 87.8 bits (96), Expect = 1e-14
Identities = 104/141 (73%), Gaps = 0/141 (0%)
Strand=Plus/Minus

```
Query  48     TATAATAAGAAGACGCTGGAGGTGGATTCGGCATGTCCTACGGAAAGGCCAGGGGGATAT  107
              ||| || |||||| | ||||| |||||  | |||||  |  | |||  |   |  |  ||
Sbjct  14327  TATCATGAGAAGAAGGTGGAGATGGATCGGACATGTAATGAGAAAAAACATAGATGGCAT  14268

Query  108    CACAAAAACAGCACTCCACTGGACCCCAGAAGGTAAAAGAAAGAGAGGAAGACCTAAAAT  167
              || |  |||||| ||||||||||| |||||||| ||||||||||| ||| | |||||||
Sbjct  14267  CATACGAACAGCCCTCCACTGGACACCAGAAGGCAAAAGAAAGAGGGGACGTCCTAAAAA  14208

Query  168    GACATGGAGAAGAACTGTAGA  188
               || ||||| |||| ||| ||
Sbjct  14207  CACTTGGAGGAGAAATGTGGA  14187
```

Score = 78.8 bits (86), Expect = 7e-12
Identities = 102/141 (72%), Gaps = 0/141 (0%)
Strand=Plus/Plus

```
Query  48      TATAATAAGAAGACGCTGGAGGTGGATTCGGCATGTCCTACGGAAAGGCCAGGGGGATAT  107
               ||| || |||||| | ||||| |||||  | |||||  |  | |||  |   |  |  ||
Sbjct  156727  TATCATGAGAAGAAGGTGGAGATGGATCGGACATGTAATGAGAAAAAACATAGATGGCAT  156786

Query  108     CACAAAAACAGCACTCCACTGGACCCCAGAAGGTAAAAGAAAGAGAGGAAGACCTAAAAT  167
               || |  || ||| ||||||||||| |||||||| ||||||||||| ||| | |||||||
Sbjct  156787  CATACGAATAGCCCTCCACTGGACACCAGAAGGCAAAAGAAAGAGGGGACGTCCTAAAAA  156846

Query  168     GACATGGAGAAGAACTGTAGA  188
                || ||   |||||||| ||
Sbjct  156847  CACTTGGCAGAGAACTGTGGA  156867
```

>**gb|AC174018.2|** D Strongylocentrotus purpuratus clone R3-1040A23, WORKING DRAFT SEQUENCE, 13 unordered pieces
Length=100800

Score = 86.0 bits (94), Expect = 5e-14
Identities = 96/127 (75%), Gaps = 1/127 (0%)
Strand=Plus/Plus

```
Query  55     AGAAGACGCTGGAGGTGGATTCGGCATGTCCTACGGAAAGGCCAGGGGGATATCACAAAA  114
              || ||| | ||||| |||||  | |||||  |  | |||| | | |  || ||||||  |
Sbjct  73292  AGGAGAAGGTGGAGATGGATCGGACATGTAATGAG-AAAGACAAAGATGACATCACACGA  73350

Query  115    ACAGCACTCCACTGGACCCCAGAAGGTAAAAGAAAGAGAGGAAGACCTAAAATGACATGG  174
              ||||| ||||||||||| |||||||| || |||||||| ||| | |||||||  || |||
Sbjct  73351  ACAGCCCTCCACTGGACACCAGAAGGCAACAGAAAGAGGGGACGTCCTAAAAACACTTGG  73410

Query  175    AGAAGAA  181
               ||||||
Sbjct  73411  CGAAGAA  73417
```

```
>gb|AC174801.2|  Strongylocentrotus purpuratus clone R3-17H5, WORKING DRAFT SEQUENCE,
9 unordered pieces
Length=167078

 Score = 78.8 bits (86),  Expect = 7e-12
 Identities = 69/86 (80%), Gaps = 0/86 (0%)
 Strand=Plus/Minus

Query  103    GATATCACAAAAACAGCACTCCACTGGACCCCAGAAGGTAAAAGAAAGAGAGGAAGACCT  162
              || ||||||  |||||| ||||||||||| |||||||| || |||||||| | | |  ||
Sbjct  70863  GAAATCACACGAACAGCCCTCCACTGGACACCAGAAGGCAACAGAAAGAGGGAACGTTCT  70804

Query  163    AAAATGACATGGAGAAGAACTGTAGA  188
              ||||  || ||| |||||||||| ||
Sbjct  70803  AAAAACACTTGGCGAAGAACTGTGGA  70778


>gb|AC180809.1|  Strongylocentrotus purpuratus clone R3-3071J15, WORKING DRAFT
SEQUENCE, 12 unordered pieces
Length=161052

 Score = 78.8 bits (86),  Expect = 7e-12
 Identities = 69/86 (80%), Gaps = 0/86 (0%)
 Strand=Plus/Plus

Query  103     GATATCACAAAAACAGCACTCCACTGGACCCCAGAAGGTAAAAGAAAGAGAGGAAGACCT  162
               || ||||||  |||||| ||||||||||| |||||||| || |||||||| | | |  ||
Sbjct  107847  GAAATCACACGAACAGCCCTCCACTGGACACCAGAAGGCAACAGAAAGAGGGAACGTTCT  107906

Query  163     AAAATGACATGGAGAAGAACTGTAGA  188
               ||||  || ||| |||||||||| ||
Sbjct  107907  AAAAACACTTGGCGAAGAACTGTGGA  107932


>gb|AC179624.1|  Strongylocentrotus purpuratus clone R3-1028F05, WORKING DRAFT
SEQUENCE, 10 unordered pieces
Length=140196

 Score = 78.8 bits (86),  Expect = 7e-12
 Identities = 102/141 (72%), Gaps = 0/141 (0%)
 Strand=Plus/Minus

Query  48     TATAATAAGAAGACGCTGGAGGTGGATTCGGCATGTCCTACGGAAAGGCCAGGGGGATAT  107
              ||| || |||||| | ||||| |||||  | |||||  |  | |||  |   |  |  ||
Sbjct  12964  TATCATGAGAAGAAGGTGGAGATGGATCGGACATGTAATGAGAAAAAACATAGATGGCAT  12905

Query  108    CACAAAAACAGCACTCCACTGGACCCCAGAAGGTAAAAGAAAGAGAGGAAGACCTAAAAT  167
              || |  || ||| ||||||||||| |||||||| ||||||||||| ||| | |||||||
Sbjct  12904  CATACGAATAGCCCTCCACTGGACACCAGAAGGCAAAAGAAAGAGGGGACGTCCTAAAAA  12845

Query  168    GACATGGAGAAGAACTGTAGA  188
               || |||   |||||||| ||
Sbjct  12844  CACTTGGCAGAGAACTGTGGA  12824


>gb|AC176193.1|  Strongylocentrotus purpuratus clone R3-3064A03, WORKING DRAFT
SEQUENCE, 13 unordered pieces
Length=182542

 Score = 78.8 bits (86),  Expect = 7e-12
 Identities = 69/86 (80%), Gaps = 0/86 (0%)
 Strand=Plus/Plus

Query  103    GATATCACAAAAACAGCACTCCACTGGACCCCAGAAGGTAAAAGAAAGAGAGGAAGACCT  162
              || ||||||  |||||| ||||||||||| |||||||| || | |||||| ||| | |||
Sbjct  82337  GACATCACATGAACAGCCCTCCACTGGACACCAGAAGGCAACAAAAAGAGGGGACGTCCT  82396

Query  163    AAAATGACATGGAGAAGAACTGTAGA  188
              ||||  || ||| | |||||||| ||
Sbjct  82397  AAAAACACTTGGTGTAGAACTGTGGA  82422


>gb|AC181785.1|  Strongylocentrotus purpuratus clone R3-1030O21, WORKING DRAFT
SEQUENCE, 18 unordered pieces
Length=135168

 Score = 77.0 bits (84),  Expect = 3e-11
 Identities = 62/75 (82%), Gaps = 0/75 (0%)
 Strand=Plus/Minus

Query  114    AACAGCACTCCACTGGACCCCAGAAGGTAAAAGAAAGAGAGGAAGACCTAAAATGACATG  173
              |||||| ||||||||||| |||||||| ||| ||||||| ||| |||||||||  ||  |
Sbjct  26044  AACAGCCCTCCACTGGACACCAGAAGGCAAACGAAAGAGGGGATGACCTAAAAACACTCG  25985

Query  174    GAGAAGAACTGTAGA  188
              |  ||||||||| ||
Sbjct  25984  GCCAAGAACTGTGGA  25970


>gb|AC181382.1|  Strongylocentrotus purpuratus clone R3-1114M19, WORKING DRAFT
SEQUENCE, 14 unordered pieces
Length=121798

 Score = 77.0 bits (84),  Expect = 3e-11
 Identities = 100/135 (74%), Gaps = 4/135 (2%)
 Strand=Plus/Minus

Query  56     GAAGACGCTGGAGGTGGATTCGGCATGTCCTACG-GAAAGGCCAGGGG-GATATCACAAA  113
              ||||| | ||||| |||||  | |||||  || | |||||  ||  |  || ||||||
Sbjct  31095  GAAGAAGGTGGAGATGGATCGGACATGT--TATGAGAAAGAACATAGATGACATCACACG  31038

Query  114    AACAGCACTCCACTGGACCCCAGAAGGTAAAAGAAAGAGAGGAAGACCTAAAATGACATG  173
              |||||| ||||||||||| ||||| || |||||||||||  || | |||||||  |  ||
Sbjct  31037  AACAGCCCTCCACTGGACACCAGACGGCAAAAGAAAGAGGAGACGTCCTAAAAACAATTG  30978
```

```
Query  174    GAGAAGAACTGTAGA  188
              | | | |||||| ||
Sbjct  30977  GTGGATAACTGTGGA  30963


>gb|AC168645.2|  Strongylocentrotus purpuratus clone R3-3017K16, WORKING DRAFT
SEQUENCE, 24 unordered pieces
Length=157250

 Score = 75.2 bits (82),  Expect = 9e-11
 Identities = 55/64 (85%), Gaps = 0/64 (0%)
 Strand=Plus/Minus

Query  103    GATATCACAAAAACAGCACTCCACTGGACCCCAGAAGGTAAAAGAAAGAGAGGAAGACCT  162
              || ||||||  |||||| ||||||||||| |||||||| ||||||||||| ||| | |||
Sbjct  86201  GACATCACACGAACAGCCCTCCACTGGACACCAGAAGGCAAAAGAAAGAGGGGACGTCCT  86142

Query  163    AAAA  166
              ||||
Sbjct  86141  AAAA  86138


>gb|AC180087.1|  Strongylocentrotus purpuratus clone R3-4008L11, WORKING DRAFT
SEQUENCE, 28 unordered pieces
Length=109175

 Score = 75.2 bits (82),  Expect = 9e-11
 Identities = 55/64 (85%), Gaps = 0/64 (0%)
 Strand=Plus/Plus

Query  103    GATATCACAAAAACAGCACTCCACTGGACCCCAGAAGGTAAAAGAAAGAGAGGAAGACCT  162
              || ||||||  |||||| ||||||||||| |||||||| ||||||||||| ||| | |||
Sbjct  74587  GACATCACACGAACAGCCCTCCACTGGACACCAGAAGGCAAAAGAAAGAGGGGACGTCCT  74646

Query  163    AAAA  166
              ||||
Sbjct  74647  AAAA  74650


>gb|AC178239.1|  Strongylocentrotus purpuratus clone R3-3086F16, WORKING DRAFT
SEQUENCE, 20 unordered pieces
Length=211087

 Score = 75.2 bits (82),  Expect = 9e-11
 Identities = 70/88 (79%), Gaps = 2/88 (2%)
 Strand=Plus/Minus

Query  103     GATATCACAAAAACAGCACTCCACTGGACCCCAGAAGG--TAAAAGAAAGAGAGGAAGAC  160
               || ||||||  |||||| ||||||||||| ||||||||   |||| |||||| ||| | |
Sbjct  108432  GACATCACACGAACAGCCCTCCACTGGACACCAGAAGGCAAAAAAAAAAGAGGGGACGTC  108373

Query  161     CTAAAATGACATGGAGAAGAACTGTAGA  188
               ||||||  || ||| | |||||||| ||
Sbjct  108372  CTAAAAACACTTGGTGTAGAACTGTGGA  108345


>gb|AC201443.1|  Strongylocentrotus purpuratus clone R3-3022G16, WORKING DRAFT
SEQUENCE, 29 unordered pieces
Length=148764

 Score = 66.2 bits (72),  Expect = 5e-08
 Identities = 67/86 (77%), Gaps = 1/86 (1%)
 Strand=Plus/Plus

Query  103    GATATCACAAAAACAGCACTCCACTGGACCCCAGAAGGTAAAAGAAAGAGAGGAAGACCT  162
              || ||||||  |||||| ||||||||||| |||||  | || | |||||| ||| | |||
Sbjct  94884  GACATCACATGAACAGCCCTCCACTGGACACCAGATTGCAACAAAAAGAGGGGACGTCCT  94943

Query  163    AAAATGACATGGAGAAGAACTGTAGA  188
              ||||  || ||| | |||||||| ||
Sbjct  94944  AAAA-CACTTGGTGTAGAACTGTGGA  94968


>gb|AC179225.1|  Strongylocentrotus purpuratus clone R3-41F23, WORKING DRAFT SEQUENCE,
23 unordered pieces
Length=185371

 Score = 66.2 bits (72),  Expect = 5e-08
 Identities = 63/81 (77%), Gaps = 0/81 (0%)
 Strand=Plus/Minus

Query  103     GATATCACAAAAACAGCACTCCACTGGACCCCAGAAGGTAAAAGAAAGAGAGGAAGACCT  162
               || ||||||  || ||| ||||||||||| |||||||| || |||||||| | | |  ||
Sbjct  112198  GAAATCACACGAATAGCCCTCCACTGGACACCAGAAGGCAACAGAAAGAGGGAACGTTCT  112139

Query  163     AAAATGACATGGAGAAGAACT  183
               ||||  || ||  ||||||||
Sbjct  112138  AAAACCACTTGACGAAGAACT  112118


>gb|AC177677.1|  Strongylocentrotus purpuratus clone R3-1017E19, WORKING DRAFT
SEQUENCE, 27 unordered pieces
Length=134486

 Score = 66.2 bits (72),  Expect = 5e-08
 Identities = 95/130 (73%), Gaps = 3/130 (2%)
 Strand=Plus/Minus

Query  55     AGAAGACGCTGGAGGTGGATTCGGCATGTCCTACGGAAAGGCCAGGGG-GATATCACAAA  113
              |||||| | ||||| |||||  | |||||  |  | ||||  ||  |  |||||||||
Sbjct  71319  AGAAGAAGATGGAGATGGATCGGACATGTAATGAG-AAAGAACATAGATGATATCACACG  71261

Query  114    AACAGCACTCCACTGGACCCCAGAAGGTAAAAGAAAGAGAGGAAGACCT-AAAATGACAT  172
              |||||| ||||| ||||| ||| | || ||| |||| || ||| | ||| ||||  || |
Sbjct  71260  AACAGCCCTCCATTGGACACCACACGGCAAAGGAAATAGGGGACGTCCTAAAAAACACTT  71201
```

```
Query  173    GGAGAAGAAC  182
              || |||||||
Sbjct  71200  GGCGAAGAAC  71191


>gb|AC177168.1|  Strongylocentrotus purpuratus clone R3-4015N17, WORKING DRAFT
SEQUENCE, 18 unordered pieces
Length=104150

 Score = 66.2 bits (72),  Expect = 5e-08
 Identities = 95/130 (73%), Gaps = 3/130 (2%)
 Strand=Plus/Minus

Query  55     AGAAGACGCTGGAGGTGGATTCGGCATGTCCTACGGAAAGGCCAGGGG-GATATCACAAA  113
              |||||| | ||||| |||||  | |||||  |  | ||||  ||  |  |||||||||
Sbjct  50670  AGAAGAAGATGGAGATGGATCGGACATGTAATGAG-AAAGAACATAGATGATATCACACG  50612

Query  114    AACAGCACTCCACTGGACCCCAGAAGGTAAAAGAAAGAGAGGAAGACCT-AAAATGACAT  172
              |||||| ||||| ||||| ||| | || ||| |||| || ||| | ||| ||||  || |
Sbjct  50611  AACAGCCCTCCATTGGACACCACACGGCAAAGGAAATAGGGGACGTCCTAAAAAACACTT  50552

Query  173    GGAGAAGAAC  182
              || |||||||
Sbjct  50551  GGCGAAGAAC  50542


>gb|AC149918.2|  Strongylocentrotus purpuratus clone R3-14A13, WORKING DRAFT SEQUENCE,
11 unordered pieces
Length=225704

 Score = 64.4 bits (70),  Expect = 2e-07
 Identities = 101/141 (71%), Gaps = 3/141 (2%)
 Strand=Plus/Minus

Query  49      ATAATAAGAAGACGCTGGAGGTGGATTCGGCATGTCCTACGGAAAGGCCAGGGG-GATAT  107
               || || || ||| | ||||| |||||  | |||||  |    ||||| ||  |  || ||
Sbjct  117050  ATCATGAGCAGAAGGTGGAGATGGATCGGACATGTAATGAA-AAAGGACATAGATGACAT  116992

Query  108     CACAAAAACAGCACTCCACTGGACCCCAGAAGGTAAAAGAAAGAGAGGAAGACCTAAAAT  167
               ||||  |||||| | | ||||||| | ||||||  | |||| ||| ||| | ||| |||
Sbjct  116991  CACACGAACAGCCC-CAACTGGACACTAGAAGGCGATAGAAGGAGGGGAGGTCCTTAAAA  116933

Query  168     GACATGGAGAAGAACTGTAGA  188
                || |||||||||||||| ||
Sbjct  116932  CACTTGGAGAAGAACTGTGGA  116912


>gb|AC180918.1|  Strongylocentrotus purpuratus clone R3-3063E24, WORKING DRAFT
SEQUENCE, 16 unordered pieces
Length=97535

 Score = 64.4 bits (70),  Expect = 2e-07
 Identities = 101/141 (71%), Gaps = 3/141 (2%)
 Strand=Plus/Minus

Query  49     ATAATAAGAAGACGCTGGAGGTGGATTCGGCATGTCCTACGGAAAGGCCAGGGG-GATAT  107
              || || || ||| | ||||| |||||  | |||||  |    ||||| ||  |  || ||
Sbjct  71988  ATCATGAGCAGAAGGTGGAGATGGATCGGACATGTAATGAA-AAAGGACATAGATGACAT  71930

Query  108    CACAAAAACAGCACTCCACTGGACCCCAGAAGGTAAAAGAAAGAGAGGAAGACCTAAAAT  167
              ||||  |||||| | | ||||||| | ||||||  | |||| ||| ||| | ||| |||
Sbjct  71929  CACACGAACAGCCC-CAACTGGACACTAGAAGGCGATAGAAGGAGGGGAGGTCCTTAAAA  71871

Query  168    GACATGGAGAAGAACTGTAGA  188
               || |||||||||||||| ||
Sbjct  71870  CACTTGGAGAAGAACTGTGGA  71850


>gb|AC180392.1|  Strongylocentrotus purpuratus clone R3-3106E8, WORKING DRAFT
SEQUENCE, 13 unordered pieces
Length=127767

 Score = 64.4 bits (70),  Expect = 2e-07
 Identities = 101/141 (71%), Gaps = 3/141 (2%)
 Strand=Plus/Plus

Query  49     ATAATAAGAAGACGCTGGAGGTGGATTCGGCATGTCCTACGGAAAGGCCAGGGG-GATAT  107
              || || || ||| | ||||| |||||  | |||||  |    ||||| ||  |  || ||
Sbjct  70451  ATCATGAGCAGAAGGTGGAGATGGATCGGACATGTAATGAA-AAAGGACATAGATGACAT  70509

Query  108    CACAAAAACAGCACTCCACTGGACCCCAGAAGGTAAAAGAAAGAGAGGAAGACCTAAAAT  167
              ||||  |||||| | | ||||||| | ||||||  | |||| ||| ||| | ||| |||
Sbjct  70510  CACACGAACAGCCC-CAACTGGACACTAGAAGGCGATAGAAGGAGGGGAGGTCCTTAAAA  70568

Query  168    GACATGGAGAAGAACTGTAGA  188
               || |||||||||||||| ||
Sbjct  70569  CACTTGGAGAAGAACTGTGGA  70589


>gb|AC173487.2|  Strongylocentrotus purpuratus clone R3-3025A17, WORKING DRAFT
SEQUENCE, 27 unordered pieces
Length=131365

 Score = 64.4 bits (70),  Expect = 2e-07
 Identities = 101/141 (71%), Gaps = 3/141 (2%)
 Strand=Plus/Plus

Query  49      ATAATAAGAAGACGCTGGAGGTGGATTCGGCATGTCCTACGGAAAGGCCAGGGG-GATAT  107
               || || || ||| | ||||| |||||  | |||||  |    ||||| ||  |  || ||
Sbjct  127864  ATCATGAGCAGAAGGTGGAGATGGATCGGACATGTAATGAA-AAAGGACATAGATGACAT  127922

Query  108     CACAAAAACAGCACTCCACTGGACCCCAGAAGGTAAAAGAAAGAGAGGAAGACCTAAAAT  167
               ||||  |||||| | | ||||||| | ||||||  | |||| ||| ||| | ||| |||
Sbjct  127923  CACACGAACAGCCC-CAACTGGACACTAGAAGGCGATAGAAGGAGGGGAGGTCCTTAAAA  127981

Query  168     GACATGGAGAAGAACTGTAGA  188
```

```
                ||  ||||||||||||||  ||
Sbjct  127982  CACTTGGAGAAGAACTGTGGA  128002


>gb|AC180780.1| Strongylocentrotus purpuratus clone R3-3076F4, WORKING DRAFT
SEQUENCE, 17 unordered pieces
Length=145637

 Score = 60.8 bits (66),  Expect = 2e-06
 Identities = 62/81 (76%), Gaps = 0/81 (0%)
 Strand=Plus/Minus

Query  103    GATATCACAAAAACAGCACTCCACTGGACCCCAGAAGGTAAAAGAAAGAGAGGAAGACCT  162
              || ||||||  |||| | |   ||||||| |||||||| ||| ||||||| ||| | |||
Sbjct  16657  GACATCACACGAACATCCCCAAACTGGACACCAGAAGGCAAACGAAAGAGGGGACGTCCT  16598

Query  163    AAAATGACATGGAGAAGAACT  183
              ||||| || | |  |||||||
Sbjct  16597  AAAATCACTTAGCAAAGAACT  16577


>gb|AC177669.1| Strongylocentrotus purpuratus clone R3-1017H4, WORKING DRAFT
SEQUENCE, 8 unordered pieces
Length=118774

 Score = 60.8 bits (66),  Expect = 2e-06
 Identities = 65/86 (75%), Gaps = 5/86 (5%)
 Strand=Plus/Plus

Query  103     GATATCACAAAAACAGCACTCCACTGGACCCCAGAAGGTAAAAGAAAGAGAGGAAGACCT  162
               || ||||||  |||||| ||||||||||| ||||     |||  |||||| ||| | |||
Sbjct  101716  GACATCACACGAACAGCCCTCCACTGGACACCAG-----AAAGCAAAGAGTGGACGTCCT  101770

Query  163     AAAATGACATGGAGAAGAACTGTAGA  188
               ||||  ||  || |||||||||| ||
Sbjct  101771  AAAAATACTCGGCGAAGAACTGTGGA  101796


>gb|AC180771.1| Strongylocentrotus purpuratus clone R3-3073N16, WORKING DRAFT
SEQUENCE, 27 unordered pieces
Length=196165

 Score = 55.4 bits (60),  Expect = 9e-05
 Identities = 96/136 (70%), Gaps = 3/136 (2%)
 Strand=Plus/Plus

Query  49     ATAATAAGAAGACGCTGGAGGTGGATTCGGCATGTCCTACGGAAAGGCCAGGGG-GATAT  107
              || || || ||| | ||||| |||||  | |||||  |    ||||| ||  |  || ||
Sbjct  12677  ATCATGAGCAGAAGGTGGAGATGGATCGGACATGTAATGAA-AAAGGACATAGATGACAT  12735

Query  108    CACAAAAACAGCACTCCACTGGACCCCAGAAGGTAAAAGAAAGAGAGGAAGACCTAAAAT  167
              ||||  |||||| | | ||||||| ||||||||  | |||| ||| ||| | |||  ||
Sbjct  12736  CACACGAACAGCCC-CAACTGGACACCAGAAGGCGATAGAAGGAGGGGACGTCCTTCAAA  12794

Query  168    GACATGGAGAAGAACT  183
               |  ||||||||||||
Sbjct  12795  CAATTGGAGAAGAACT  12810


>gb|AC178759.1| Strongylocentrotus purpuratus clone R3-19C07, WORKING DRAFT SEQUENCE,
16 unordered pieces
Length=178001

 Score = 55.4 bits (60),  Expect = 9e-05
 Identities = 96/136 (70%), Gaps = 3/136 (2%)
 Strand=Plus/Minus

Query  49     ATAATAAGAAGACGCTGGAGGTGGATTCGGCATGTCCTACGGAAAGGCCAGGGG-GATAT  107
              || || || ||| | ||||| |||||  | |||||  |    ||||| ||  |  || ||
Sbjct  37373  ATCATGAGCAGAAGGTGGAGATGGATCGGACATGTAATGAA-AAAGGACATAGATGACAT  37315

Query  108    CACAAAAACAGCACTCCACTGGACCCCAGAAGGTAAAAGAAAGAGAGGAAGACCTAAAAT  167
              ||||  |||||| | | ||||||| ||||||||  | |||| ||| ||| | |||  ||
Sbjct  37314  CACACGAACAGCCC-CAACTGGACACCAGAAGGCGATAGAAGGAGGGGACGTCCTTCAAA  37256

Query  168    GACATGGAGAAGAACT  183
               |  ||||||||||||
Sbjct  37255  CAATTGGAGAAGAACT  37240


>gb|AC174871.2| Strongylocentrotus purpuratus clone R3-3112P21, WORKING DRAFT
SEQUENCE, 9 unordered pieces
Length=127983

 Score = 51.8 bits (56),  Expect = 0.001
 Identities = 45/56 (80%), Gaps = 0/56 (0%)
 Strand=Plus/Minus

Query  133    CCAGAAGGTAAAAGAAAGAGAGGAAGACCTAAAATGACATGGAGAAGAACTGTAGA  188
              |||||| | ||||||||||| ||| | |||||||  || ||  |||||||||| ||
Sbjct  14826  CCAGAAAGCAAAAGAAAGAGCGGACGTCCTAAAAACACTTGACGAAGAACTGTGGA  14771


>gb|AC178982.1| Strongylocentrotus purpuratus clone R3-3122I22, WORKING DRAFT
SEQUENCE, 12 unordered pieces
Length=103136

 Score = 51.8 bits (56),  Expect = 0.001
 Identities = 45/56 (80%), Gaps = 0/56 (0%)
 Strand=Plus/Minus

Query  133    CCAGAAGGTAAAAGAAAGAGAGGAAGACCTAAAATGACATGGAGAAGAACTGTAGA  188
              |||||| | ||||||||||| ||| | |||||||  || ||  |||||||||| ||
Sbjct  80126  CCAGAAAGCAAAAGAAAGAGCGGACGTCCTAAAAACACTTGACGAAGAACTGTGGA  80071
```

```
>gb|AC211231.4| Zea mays chromosome 6 clone CH201-465L2; ZMMBBc0465L02, *** SEQUENCING
IN PROGRESS ***, 19 unordered pieces
Length=172956

 Score = 48.2 bits (52),  Expect = 0.013
 Identities = 32/36 (88%), Gaps = 0/36 (0%)
 Strand=Plus/Minus

Query  144     AAGAAAGAGAGGAAGACCTAAAATGACATGGAGAAG  179
               ||||| |||| |||||||||||||| ||| ||||||
Sbjct  114473  AAGAAGGAGAAGAAGACCTAAAATGCCATCGAGAAG  114438


>gb|AC203468.2| Echinops telfairi clone CH238-500M2, WORKING DRAFT SEQUENCE,
4 ordered pieces
Length=179294

 Score = 48.2 bits (52),  Expect = 0.013
 Identities = 35/41 (85%), Gaps = 0/41 (0%)
 Strand=Plus/Minus

Query  135    AGAAGGTAAAAGAAAGAGAGGAAGACCTAAAATGACATGGA  175
              ||| ||||||||||| |||||||||||   ||||| |||||
Sbjct  63333  AGATGGTAAAAGAAATAGAGGAAGACCCTCAATGAGATGGA  63293


>gb|AC195424.2| Echinops telfairi clone CH238-291E4, WORKING DRAFT SEQUENCE,
3 ordered pieces
Length=208205

 Score = 48.2 bits (52),  Expect = 0.013
 Identities = 35/41 (85%), Gaps = 0/41 (0%)
 Strand=Plus/Minus

Query  135     AGAAGGTAAAAGAAAGAGAGGAAGACCTAAAATGACATGGA  175
               ||| ||||||||||| |||||||||||   ||||| |||||
Sbjct  161384  AGATGGTAAAAGAAATAGAGGAAGACCCTCAATGAGATGGA  161344


>gb|AC206128.4| Saccoglossus kowalevskii clone CUGI_SK_BA 001B19, WORKING DRAFT
SEQUENCE, 2 unordered pieces
Length=129006

 Score = 46.4 bits (50),  Expect = 0.044
 Identities = 48/63 (76%), Gaps = 0/63 (0%)
 Strand=Plus/Minus

Query  127    TGGACCCCAGAAGGTAAAAGAAAGAGAGGAAGACCTAAAATGACATGGAGAAGAACTGTA  186
              ||| | |||||||| ||||| ||  | || ||||| ||||  || ||| || | ||||||
Sbjct  11608  TGGGCACCAGAAGGGAAAAGGAAATGGGGTAGACCCAAAACAACTTGGCGACGCACTGTA  11549

Query  187    GAG  189
              |||
Sbjct  11548  GAG  11546


>gb|AC183238.2| Bos taurus clone CH240-97P5, WORKING DRAFT SEQUENCE, 4 unordered
pieces
Length=206136

 Score = 44.6 bits (48),  Expect = 0.15
 Identities = 26/27 (96%), Gaps = 0/27 (0%)
 Strand=Plus/Minus

Query  140    GTAAAAGAAAGAGAGGAAGACCTAAAA  166
              ||||||||||||||||||||||| |||
Sbjct  84552  GTAAAAGAAAGAGAGGAAGACCTGAAA  84526


>gb|AC159723.3| Bos taurus clone CH240-79H4, WORKING DRAFT SEQUENCE, 8 unordered
pieces
Length=222234

 Score = 44.6 bits (48),  Expect = 0.15
 Identities = 33/39 (84%), Gaps = 0/39 (0%)
 Strand=Plus/Plus

Query  136     GAAGGTAAAAGAAAGAGAGGAAGACCTAAAATGACATGG  174
               |||||||||||||  |||||||| |  ||||||| ||||
Sbjct  146482  GAAGGTAAAAGAAGAAGAGGAAGGCAGAAAATGAAATGG  146520


>dbj|AP008007.2| Lotus japonicus chromosome 3 clone LjB03J23, *** SEQUENCING IN
PROGRESS ***, 20 unordered pieces
Length=96846

 Score = 44.6 bits (48),  Expect = 0.15
 Identities = 42/52 (80%), Gaps = 4/52 (7%)
 Strand=Plus/Minus

Query  142    AAAAGAAAGAGA--GGAAGACCTAAAATGACATGGAGAAG--AACTGTAGAG  189
              ||||||| ||||  |||||||| | ||||| |||| ||||  || |||||||
Sbjct  14254  AAAAGAAGGAGATCGGAAGACCGAGAATGAGATGGGGAAGAAAAATGTAGAG  14203


>gb|AC159735.3| Bos taurus clone CH240-35L21, WORKING DRAFT SEQUENCE
Length=162749

 Score = 44.6 bits (48),  Expect = 0.15
 Identities = 39/47 (82%), Gaps = 4/47 (8%)
 Strand=Plus/Plus

Query  132    CCCAGAAGGTAAAAGAAAGAGAGGAAGACCTAAAATGACATGGAGAA  178
              ||||||||| ||||||||||| |||||||   |||| |  |||||||
Sbjct  54027  CCCAGAAGG-AAAAGAAAGAGGGGAAGAC---AAATTATTTGGAGAA  54069
```

>**gb|AC181518.1|** Strongylocentrotus purpuratus clone R3-62E1, WORKING DRAFT SEQUENCE, 28 unordered pieces
Length=170173

```
 Score = 44.6 bits (48),  Expect = 0.15
 Identities = 38/47 (80%), Gaps = 0/47 (0%)
 Strand=Plus/Minus

Query  142     AAAAGAAAGAGAGGAAGACCTAAAATGACATGGAGAAGAACTGTAGA  188
               |||||||||||||| |||||   ||| | |||||| |||||  ||||
Sbjct  115491  AAAAGAAAGAGAGGTAGACCAGTAATAAAATGGAGGAGAACAATAGA  115445
```

>**gb|AC180090.1|** Strongylocentrotus purpuratus clone R3-3001I05, WORKING DRAFT SEQUENCE, 31 unordered pieces
Length=202729

```
 Score = 44.6 bits (48),  Expect = 0.15
 Identities = 33/39 (84%), Gaps = 0/39 (0%)
 Strand=Plus/Minus

Query  133    CCAGAAGGTAAAAGAAAGAGAGGAAGACCTAAAATGACA  171
              ||||  || |||||||||||||||||||| | ||| |||
Sbjct  44650  CCAGCTGGAAAAAGAAAGAGAGGAAGACCAATAATTACA  44612
```

>**gb|AC162133.2|** Loxodonta africana clone VMRC15-344J20, WORKING DRAFT SEQUENCE, 6 ordered pieces
Length=126422

Sort alignments for this subject sequence by:
E value **Score** **Percent identity**
Query start position **Subject start position**

```
 Score = 44.6 bits (48),  Expect = 0.15
 Identities = 35/42 (83%), Gaps = 0/42 (0%)
 Strand=Plus/Plus

Query  134    CAGAAGGTAAAAGAAAGAGAGGAAGACCTAAAATGACATGGA  175
              |||||||| |  ||||||||||||||||   ||||| |||||
Sbjct  33536  CAGAAGGTCAGTGAAAGAGAGGAAGACCCTCAATGAGATGGA  33577


 Score = 41.0 bits (44),  Expect = 1.9
 Identities = 27/30 (90%), Gaps = 0/30 (0%)
 Strand=Plus/Plus

Query  146    GAAAGAGAGGAAGACCTAAAATGACATGGA  175
              |||||||||||||||||  ||||| |||||
Sbjct  38486  GAAAGAGAGGAAGACCTTCAATGAGATGGA  38515
```

>**gb|AC176910.1|** Strongylocentrotus purpuratus clone R3-1104N16, WORKING DRAFT SEQUENCE, 15 unordered pieces
Length=151914

```
 Score = 44.6 bits (48),  Expect = 0.15
 Identities = 33/39 (84%), Gaps = 0/39 (0%)
 Strand=Plus/Plus

Query  133    CCAGAAGGTAAAAGAAAGAGAGGAAGACCTAAAATGACA  171
              ||||  || |||||||||||||||||||| | ||| |||
Sbjct  17622  CCAGCTGGAAAAAGAAAGAGAGGAAGACCAATAATTACA  17660
```

>**gb|AC164836.2|** Bos taurus clone CH240-140B23, *** SEQUENCING IN PROGRESS ***, 22 unordered pieces
Length=208739

```
 Score = 44.6 bits (48),  Expect = 0.15
 Identities = 33/39 (84%), Gaps = 0/39 (0%)
 Strand=Plus/Minus

Query  136     GAAGGTAAAAGAAAGAGAGGAAGACCTAAAATGACATGG  174
               |||||||||||||  |||||||| |  ||||||| ||||
Sbjct  183570  GAAGGTAAAAGAAGAAGAGGAAGGCAGAAAATGAAATGG  183532
```

>**gb|AC164913.2|** Bos taurus clone CH240-155C17, *** SEQUENCING IN PROGRESS ***, 7 unordered pieces
Length=199475

```
 Score = 44.6 bits (48),  Expect = 0.15
 Identities = 39/47 (82%), Gaps = 4/47 (8%)
 Strand=Plus/Minus

Query  132    CCCAGAAGGTAAAAGAAAGAGAGGAAGACCTAAAATGACATGGAGAA  178
              ||||||||| ||||||||||| |||||||    |||| |  |||||||
Sbjct  40310  CCCAGAAGG-AAAAGAAAGAGGGGAAGAC---AAATTATTTGGAGAA  40268
```

>**emb|FP312800.2|** Sus scrofa chromosome 6 clone CH242-341I3, WORKING DRAFT SEQUENCE
Length=203145

```
 Score = 42.8 bits (46),  Expect = 0.54
 Identities = 32/38 (84%), Gaps = 0/38 (0%)
 Strand=Plus/Minus

Query  90      GAAAGGCCAGGGGGATATCACAAAAACAGCACTCCACT  127
               ||||  ||| ||||| || ||| |||||||||||||||
Sbjct  177484  GAAATTCCACGGGGAAATTACAGAAACAGCACTCCACT  177447
```

>**gb|AC134160.3|** Rattus norvegicus clone CH230-93N17, WORKING DRAFT SEQUENCE, 2 ordered pieces
Length=246397

```
 Score = 42.8 bits (46),  Expect = 0.54
 Identities = 37/46 (80%), Gaps = 0/46 (0%)
 Strand=Plus/Plus

Query  134     CAGAAGGTAAAAGAAAGAGAGGAAGACCTAAAATGACATGGAGAAG  179
               |||||| |||||||||| || |||||  |||| |||| | |||||
Sbjct  118056  CAGAAGAAAAAAGAAAGACAGAAAGACAGAAAAAGACAGGAAGAAG  118101
```

>**emb|CU856279.2|** Sus scrofa chromosome 15 clone CH242-212D4, WORKING DRAFT SEQUENCE, 11 unordered pieces
Length=168065

```
 Score = 42.8 bits (46),  Expect = 0.54
 Identities = 25/26 (96%), Gaps = 0/26 (0%)
 Strand=Plus/Minus

Query  134    CAGAAGGTAAAAGAAAGAGAGGAAGA  159
              ||||||| ||||||||||||||||||
Sbjct  71179  CAGAAGGAAAAAGAAAGAGAGGAAGA  71154
```

>**gb|AC194836.3|** Zea mays chromosome 10 clone CH201-479A12; ZMMBBc0479A12, *** SEQUENCING IN PROGRESS ***, 18 unordered pieces
Length=187446

```
 Score = 42.8 bits (46),  Expect = 0.54
 Identities = 34/39 (87%), Gaps = 3/39 (7%)
 Strand=Plus/Plus

Query  144    AAGAAAGA--GAGGAAGACCTAAAATGACATGGAGAAGA  180
              ||||||||  |||||||||| ||| |||||||| |||||
Sbjct  92756  AAGAAAGAAAGAGGAAGACCGAAACTGACATGG-GAAGA  92793
```

>**gb|AC199408.4|** Zea mays chromosome 10 clone CH201-196G4; ZMMBBc0196G04, *** SEQUENCING IN PROGRESS ***, 8 unordered pieces
Length=153153

```
 Score = 42.8 bits (46),  Expect = 0.54
 Identities = 34/39 (87%), Gaps = 3/39 (7%)
 Strand=Plus/Minus

Query  144    AAGAAAGA--GAGGAAGACCTAAAATGACATGGAGAAGA  180
              ||||||||  |||||||||| ||| |||||||| |||||
Sbjct  23530  AAGAAAGAAAGAGGAAGACCGAAACTGACATGG-GAAGA  23493
```

>**gb|AC230641.1|** Bos taurus clone CH240-502O9, WORKING DRAFT SEQUENCE, 8 unordered pieces
Length=197142

```
 Score = 42.8 bits (46),  Expect = 0.54
 Identities = 26/28 (92%), Gaps = 0/28 (0%)
 Strand=Plus/Plus

Query  136    GAAGGTAAAAGAAAGAGAGGAAGACCTA  163
              ||||| |||||||||| |||||||||||
Sbjct  91177  GAAGGAAAAAGAAAGAAAGGAAGACCTA  91204
```

>**gb|AC220510.1|** Bos taurus clone CH240-355P4, WORKING DRAFT SEQUENCE, 11 unordered pieces
Length=190319

```
 Score = 42.8 bits (46),  Expect = 0.54
 Identities = 40/50 (80%), Gaps = 1/50 (2%)
 Strand=Plus/Plus

Query  149    AGAGA-GGAAGACCTAAAATGACATGGAGAAGAACTGTAGAGGCAGAGGC  197
              ||||| ||||||||| ||| |||| |||||| || || ||||   |||||
Sbjct  35264  AGAGAAGGAAGACCTTAAAAGACACGGAGAATAAGTGCAGAGTTTGAGGC  35313
```

>**gb|AC219995.1|** Bos taurus clone CH240-316G8, WORKING DRAFT SEQUENCE, 4 unordered pieces
Length=199877

```
 Score = 42.8 bits (46),  Expect = 0.54
 Identities = 26/28 (92%), Gaps = 0/28 (0%)
 Strand=Plus/Minus

Query  136     GAAGGTAAAAGAAAGAGAGGAAGACCTA  163
               ||||| |||||||||| |||||||||||
Sbjct  178210  GAAGGAAAAAGAAAGAAAGGAAGACCTA  178183
```

>**gb|AC217613.2|** Procavia capensis clone CH280-98O19, WORKING DRAFT SEQUENCE, 9 ordered pieces
Length=176492

Sort alignments for this subject sequence by:
E value **Score** **Percent identity**
Query start position **Subject start position**

```
 Score = 42.8 bits (46),  Expect = 0.54
 Identities = 34/41 (82%), Gaps = 0/41 (0%)
 Strand=Plus/Plus

Query  134    CAGAAGGTAAAAGAAAGAGAGGAAGACCTAAAATGACATGG  174
              |||| |||||| |||| ||| ||||||||  |||| |||||
```

```
Sbjct  85431  CAGAGGGTAAATGAAAAAGATGAAGACCTTCAATGGCATGG  85471


 Score = 41.0 bits (44),  Expect = 1.9
 Identities = 34/42 (80%), Gaps = 0/42 (0%)
 Strand=Plus/Minus

Query  134    CAGAAGGTAAAAGAAAGAGAGGAAGACCTAAAATGACATGGA  175
              |||| ||| || |||| |||||||||||   ||||| |||||
Sbjct  26776  CAGAGGGTCAATGAAAAAGAGGAAGACCCTGAATGAGATGGA  26735
```

>**gb|AC216304.2|** Equus caballus clone CH241-277H1, WORKING DRAFT SEQUENCE, 5 ordered pieces
Length=271050

```
 Score = 42.8 bits (46),  Expect = 0.54
 Identities = 34/41 (82%), Gaps = 0/41 (0%)
 Strand=Plus/Minus

Query  135     AGAAGGTAAAAGAAAGAGAGGAAGACCTAAAATGACATGGA  175
               |||||| || || || ||||||||||| || |||| |||||
Sbjct  218900  AGAAGGCAACAGCAAAAGAGGAAGACCAAACATGAGATGGA  218860
```

>**gb|AC216525.1|** Loxodonta africana clone VMRC15-120J1, WORKING DRAFT SEQUENCE, 3 unordered pieces
Length=135082

```
 Score = 42.8 bits (46),  Expect = 0.54
 Identities = 34/41 (82%), Gaps = 0/41 (0%)
 Strand=Plus/Plus

Query  135    AGAAGGTAAAAGAAAGAGAGGAAGACCTAAAATGACATGGA  175
              ||| ||| ||||||| |||||||||||  | |||| |||||
Sbjct  74825  AGAGGGTTAAAGAAAAAGAGGAAGACCCTACATGAGATGGA  74865
```

>**gb|AC213170.2|** Equus caballus clone CH241-385H17, WORKING DRAFT SEQUENCE, 6 ordered pieces
Length=195585

```
 Score = 42.8 bits (46),  Expect = 0.54
 Identities = 34/41 (82%), Gaps = 0/41 (0%)
 Strand=Plus/Minus

Query  135    AGAAGGTAAAAGAAAGAGAGGAAGACCTAAAATGACATGGA  175
              |||||| || || || ||||||||||| || |||| |||||
Sbjct  54467  AGAAGGCAACAGCAAAAGAGGAAGACCAAACATGAGATGGA  54427
```

>**gb|AC121463.4|** Rattus norvegicus clone CH230-33J4, WORKING DRAFT SEQUENCE, 4 ordered pieces
Length=261450

```
 Score = 42.8 bits (46),  Expect = 0.54
 Identities = 37/46 (80%), Gaps = 0/46 (0%)
 Strand=Plus/Minus

Query  134     CAGAAGGTAAAAGAAAGAGAGGAAGACCTAAAATGACATGGAGAAG  179
               ||||||  |||||||||| || |||||  |||| |||| | |||||
Sbjct  239005  CAGAAGAAAAAAGAAAGACAGAAAGACAGAAAAAGACAGGAAGAAG  238960
```

>**gb|AC190377.2|** Loxodonta africana clone VMRC15-393J21, WORKING DRAFT SEQUENCE, 10 ordered pieces
Length=135246

```
 Score = 42.8 bits (46),  Expect = 0.54
 Identities = 34/41 (82%), Gaps = 0/41 (0%)
 Strand=Plus/Plus

Query  135    AGAAGGTAAAAGAAAGAGAGGAAGACCTAAAATGACATGGA  175
              ||| ||| ||||||| |||||||||||  | |||| |||||
Sbjct  10032  AGAGGGTTAAAGAAAAAGAGGAAGACCCTACATGAGATGGA  10072
```

>**gb|AC185202.2|** Loxodonta africana clone VMRC15-2I2, WORKING DRAFT SEQUENCE, 9 ordered pieces
Length=129662

```
 Score = 42.8 bits (46),  Expect = 0.54
 Identities = 34/41 (82%), Gaps = 0/41 (0%)
 Strand=Plus/Plus

Query  135    AGAAGGTAAAAGAAAGAGAGGAAGACCTAAAATGACATGGA  175
              ||| ||| ||||||| |||||||||||  | |||| |||||
Sbjct  68586  AGAGGGTTAAAGAAAAAGAGGAAGACCCTACATGAGATGGA  68626
```

>**gb|AC183883.2|** Loxodonta africana clone VMRC15-544N16, WORKING DRAFT SEQUENCE, 8 ordered pieces
Length=153068

```
 Score = 42.8 bits (46),  Expect = 0.54
 Identities = 34/41 (82%), Gaps = 0/41 (0%)
 Strand=Plus/Minus

Query  135     AGAAGGTAAAAGAAAGAGAGGAAGACCTAAAATGACATGGA  175
               || |||| || ||||||||||||||||   ||||| |||||
Sbjct  111828  AGTAGGTCAATGAAAGAGAGGAAGACCCTTAATGAGATGGA  111788
```

>**gb|AC168586.2|** Strongylocentrotus purpuratus clone R3-3065F16, WORKING DRAFT SEQUENCE, 25 unordered pieces

Length=173543

Score = 42.8 bits (46), Expect = 0.54
Identities = 41/53 (77%), Gaps = 0/53 (0%)
Strand=Plus/Minus

```
Query  136    GAAGGTAAAAGAAAGAGAGGAAGACCTAAAATGACATGGAGAAGAACTGTAGA  188
              ||| | ||||||||||  |||   |||||||  || ||| |||||||||| ||
Sbjct  29501  GAATGCAAAAGAAAGAAGGGACATCCTAAAAACACTTGGCGAAGAACTGTGGA  29449
```

>**gb|AC168548.2|** Strongylocentrotus purpuratus clone R3-3091I21, WORKING DRAFT SEQUENCE, 32 unordered pieces
Length=171325

Score = 42.8 bits (46), Expect = 0.54
Identities = 35/43 (81%), Gaps = 0/43 (0%)
Strand=Plus/Minus

```
Query  103    GATATCACAAAAACAGCACTCCACTGGACCCCAGAAGGTAAAA  145
              || ||||||   ||||| ||||||||||| ||||||  |||||
Sbjct  95094  GACATCACACGGACAGCCCTCCACTGGACACCAGAATTTAAAA  95052
```

>**gb|AC128025.2|** Rattus norvegicus clone CH230-124G14, *** SEQUENCING IN PROGRESS ***
Length=266038

Score = 42.8 bits (46), Expect = 0.54
Identities = 29/33 (87%), Gaps = 0/33 (0%)
Strand=Plus/Minus

```
Query  143     AAAGAAAGAGAGGAAGACCTAAAATGACATGGA  175
               ||||||||| || |||| ||||||||||| |||
Sbjct  171904  AAAGAAAGAAAGAAAGATCTAAAATGACAAGGA  171872
```

>**gb|AC023199.2|** Homo sapiens chromosome 7 clone RP11-33L7 map 7, WORKING DRAFT SEQUENCE, 11 unordered pieces
Length=165301

Score = 42.8 bits (46), Expect = 0.54
Identities = 30/33 (90%), Gaps = 1/33 (3%)
Strand=Plus/Plus

```
Query  151     AGAGGAAGA-CCTAAAATGACATGGAGAAGAAC  182
               ||||||||| |||| ||||||| ||||||||||
Sbjct  141677  AGAGGAAGAGCCTAGAATGACACGGAGAAGAAC  141709
```

>**gb|AC097013.7|** Schistosoma mansoni chromosome 0 clone Sm1-48C10, *** SEQUENCING IN PROGRESS ***, 5 unordered pieces
Length=141690

Score = 42.8 bits (46), Expect = 0.54
Identities = 49/66 (74%), Gaps = 0/66 (0%)
Strand=Plus/Minus

```
Query  107    TCACAAAAACAGCACTCCACTGGACCCCAGAAGGTAAAAGAAAGAGAGGAAGACCTAAAA  166
              |||||| |  |||||||   ||||  || |||||| |||| |  ||||||||||| || |
Sbjct  68633  TCACAAGACAAGCACTCACATGGAATCCTGAAGGTCAAAGGAGAAGAGGAAGACCAAAGA  68574

Query  167    TGACAT  172
                ||||
Sbjct  68573  ACACAT  68568
```

>**gb|AC236086.3|** Chlorocebus aethiops clone CH252-214P17, WORKING DRAFT SEQUENCE, 6 ordered pieces
Length=186833

Score = 41.0 bits (44), Expect = 1.9
Identities = 40/52 (76%), Gaps = 0/52 (0%)
Strand=Plus/Minus

```
Query  135    AGAAGGTAAAAGAAAGAGAGGAAGACCTAAAATGACATGGAGAAGAACTGTA  186
              |||||| |||||||||| || ||   ||||| | | |  ||||||| | |||||
Sbjct  76303  AGAAGGAAAAAGAAAGAAAGAAACTGCTAAACTAAATTGGAGAATAGCTGTA  76252
```

>**gb|AC212779.3|** Zea mays chromosome 2 clone CH201-110I6; ZMMBBc0110I06, *** SEQUENCING IN PROGRESS ***, 5 unordered pieces
Length=180756

Score = 41.0 bits (44), Expect = 1.9
Identities = 40/49 (81%), Gaps = 2/49 (4%)
Strand=Plus/Plus

```
Query  148    AAGAGAGGAAGACCTAAAATGACATGGAGAAGA-ACTGTAGAGGCAGAG  195
              |||||||||||||| ||  ||||||||| |||| || ||| | | ||||
Sbjct  43788  AAGAGAGGAAGACCGAAGTTGACATGGA-AAGAGACAGTAAAAGAAGAG  43835
```

>**gb|AC231323.2|** Loxodonta africana clone VMRC15-463J4, WORKING DRAFT SEQUENCE, 11 ordered pieces
Length=143192

Score = 41.0 bits (44), Expect = 1.9
Identities = 34/42 (80%), Gaps = 0/42 (0%)
Strand=Plus/Plus

```
Query  134    CAGAAGGTAAAAGAAAGAGAGGAAGACCTAAAATGACATGGA  175
              |||| ||| | ||||| |||||||||||   ||||| |||||
Sbjct  80871  CAGAGGGTCAGAGAAAAAGAGGAAGACCCTCAATGATATGGA  80912
```

>**gb|EU875592.1|** Branchiostoma floridae clone Bac 100J9, *** SEQUENCING IN PROGRESS ***, 2 ordered pieces
Length=177370

```
 Score = 41.0 bits (44),  Expect = 1.9
 Identities = 42/55 (76%), Gaps = 0/55 (0%)
 Strand=Plus/Plus

Query  131     CCCCAGAAGGTAAAAGAAAGAGAGGAAGACCTAAAATGACATGGAGAAGAACTGT  185
               ||| ||| || | |||||| ||||||||||| ||||  |  ||||| | ||| ||
Sbjct  113161  CCCTAGATGGAAGAAGAAAAAGAGGAAGACCCAAAACCAAGTGGAGGAAAACAGT  113215
```

>**gb|AC149662.4|** Bos taurus clone CH240-66A10, WORKING DRAFT SEQUENCE, 8 unordered pieces
Length=257617

```
 Score = 41.0 bits (44),  Expect = 1.9
 Identities = 22/22 (100%), Gaps = 0/22 (0%)
 Strand=Plus/Minus

Query  129    GACCCCAGAAGGTAAAAGAAAG  150
              ||||||||||||||||||||||
Sbjct  34838  GACCCCAGAAGGTAAAAGAAAG  34817
```

>**gb|AC230587.1|** Bos taurus clone CH240-504C12, WORKING DRAFT SEQUENCE, 8 unordered pieces
Length=188909

```
 Score = 41.0 bits (44),  Expect = 1.9
 Identities = 27/30 (90%), Gaps = 0/30 (0%)
 Strand=Plus/Minus

Query  96     CCAGGGGGATATCACAAAAACAGCACTCCA  125
              ||||||||| || ||| |||||||||||||
Sbjct  11771  CCAGGGGGAAATTACAGAAACAGCACTCCA  11742
```

>**dbj|AP010078.1|** Lotus japonicus clone LjT33M20, *** SEQUENCING IN PROGRESS ***, 24 unordered pieces
Length=124181

```
 Score = 41.0 bits (44),  Expect = 1.9
 Identities = 33/40 (82%), Gaps = 0/40 (0%)
 Strand=Plus/Plus

Query  142    AAAAGAAAGAGAGGAAGACCTAAAATGACATGGAGAAGAA  181
              |||||||||||| ||| |   ||||||| ||||| |||||
Sbjct  12706  AAAAGAAAGAGAAGAATAGTGAAAATGAGATGGAAAAGAA  12745
```

>**gb|AC225966.1|** Loxodonta africana clone VMRC15-206J12, WORKING DRAFT SEQUENCE, 3 unordered pieces
Length=149345

```
 Score = 41.0 bits (44),  Expect = 1.9
 Identities = 34/42 (80%), Gaps = 0/42 (0%)
 Strand=Plus/Minus

Query  134    CAGAAGGTAAAAGAAAGAGAGGAAGACCTAAAATGACATGGA  175
              |||||| | || |||| |||||||||||   ||||| |||||
Sbjct  50977  CAGAAGTTCAATGAAAAAGAGGAAGACCGTCAATGAGATGGA  50936
```

>**emb|CU633680.2|** Sus scrofa chromosome 13 clone CH242-251F9, WORKING DRAFT SEQUENCE
Length=185674

```
 Score = 41.0 bits (44),  Expect = 1.9
 Identities = 22/22 (100%), Gaps = 0/22 (0%)
 Strand=Plus/Plus

Query  14      TCATCAGATGCCAACAACAAGA  35
               ||||||||||||||||||||||
Sbjct  115999  TCATCAGATGCCAACAACAAGA  116020
```

>**gb|AC217228.3|** Procavia capensis clone CH280-20O24, WORKING DRAFT SEQUENCE, 4 ordered pieces
Length=180133

```
 Score = 41.0 bits (44),  Expect = 1.9
 Identities = 34/42 (80%), Gaps = 0/42 (0%)
 Strand=Plus/Minus

Query  134     CAGAAGGTAAAAGAAAGAGAGGAAGACCTAAAATGACATGGA  175
               |||| ||| || |||| |||||||||||   ||||| |||||
Sbjct  173768  CAGAGGGTCAATGAAAAAGAGGAAGACCCTGAATGAGATGGA  173727
```

>**gb|AC214731.3|** Procavia capensis clone CH280-198C7, WORKING DRAFT SEQUENCE, 6 ordered pieces
Length=202419

```
 Score = 41.0 bits (44),  Expect = 1.9
 Identities = 30/35 (85%), Gaps = 0/35 (0%)
 Strand=Plus/Minus

Query  135    AGAAGGTAAAAGAAAGAGAGGAAGACCTAAAATGA  169
              ||||||| | ||||| ||||||||||||  |||||
Sbjct  88927  AGAAGGTCAGAGAAAAAGAGGAAGACCTTCAATGA  88893
```

>**gb|AC215573.2|** Procavia capensis clone CH280-21J17, WORKING DRAFT SEQUENCE, 4 ordered pieces

```
Length=211098

 Score = 41.0 bits (44),  Expect = 1.9
 Identities = 34/42 (80%), Gaps = 0/42 (0%)
 Strand=Plus/Minus

Query  134     CAGAAGGTAAAAGAAAGAGAGGAAGACCTAAAATGACATGGA  175
               |||||||| | ||||| |||||||||||   || || |||||
Sbjct  130230  CAGAAGGTCAGAGAAAAAGAGGAAGACCCTCAACGAGATGGA  130189
```

>**gb|AC162188.4|** D Bos taurus clone CH240-116P1, WORKING DRAFT SEQUENCE, 9 unordered pieces

```
Length=204345

 Score = 41.0 bits (44),  Expect = 1.9
 Identities = 27/30 (90%), Gaps = 0/30 (0%)
 Strand=Plus/Minus

Query  96      CCAGGGGGATATCACAAAAACAGCACTCCA  125
               ||||||||| || ||| |||||||||||||
Sbjct  155179  CCAGGGGGAAATTACAGAAACAGCACTCCA  155150
```

>**gb|AC196583.6|** D Macaca mulatta clone CH250-66M21, WORKING DRAFT SEQUENCE, 4 ordered pieces

```
Length=167834

 Score = 41.0 bits (44),  Expect = 1.9
 Identities = 42/54 (77%), Gaps = 1/54 (1%)
 Strand=Plus/Plus

Query  112     AAAACAGCACTCCACTGGACCCCAGAAGGTAAAAGAAAGAGAGGAAGACCTAAA  165
               ||||||| ||  ||||||| ||||| ||| || ||||| |||| | || | |||
Sbjct  126730  AAAACAG-ACATCACTGGATCCCAGGAGGAAAGAGAAAAAGAGAATGAGCAAAA  126782
```

Select All Get selected sequences Distance tree of results Multiple alignment NEW
