# Supplementary material for: Promoter Complexity and Tissue-Specific Expression of Stress Response Components in Mytilus galloprovincialis, a Sessile Marine Invertebrate Species
Source: PLoS Comput Biol. 2010 Jul 8;6(7):e1000847. doi: 10.1371/journal.pcbi.1000847 (PMC2900285; doi:10.1371/journal.pcbi.1000847)
Supplement: Protocol S1 — 18 Supplement files plus an index file: 3 Supplementary figures, 2 Supplementary tables - referenced in text as Protocol S1; index provided with an explanation of the directory contents. (5.18 MB ZIP) [file pcbi.1000847.s001.zip › SUPPLEMENTS18/SupplFigure2.4a.pdf]

BLAST Basic Local Alignment Search Tool

-

Edit and Resubmit Save Search Strategies Formatting options Download

Nucleotide Sequence (328 letters)

Results for: lcl|12199 None(201bp)

Your BLAST job specified more than one input sequence. This box lets you choose which input sequence to show BLAST results for.

Query ID
lcl|12199
Description
None
Molecule type
nucleic acid
Query Length
201

Database Name
wgs
Description
Whole-Genome-Shotgun Sequences
Program
BLASTN 2.2.22+ Citation

Reference
Stephen F. Altschul, Thomas L. Madden, Alejandro A. Schäffer, Jinghui Zhang, Zheng Zhang, Webb Miller, and David J. Lipman (1997), "Gapped BLAST and PSI-BLAST: a new generation of protein database search programs", Nucleic Acids Res. 25:3389-3402.
Other reports: Search Summary [Taxonomy reports] [Distance tree of results]

Search Parameters

Program	blastn
Word size	7
Expect value	10
Hitlist size	100
Match/Mismatch scores	2,-3
Gapcosts	5,2
Low Complexity Filter	Yes
Filter string	L;m;
Genetic Code	1

Database

Posted date	Oct 16, 2009 5:42 PM
Number of letters	144,802,826,610
Number of sequences	30,486,747
Entrez query	none

Karlin-Altschul statistics

Params	Ungapped	Gapped
Lambda	0.633731	0.625
K	0.408146	0.41
H	0.912438	0.78

Results Statistics

Length adjustment	36
Effective length of query	165
Effective length of database	143705303718
Effective search space	23711375113470
Effective search space used	23711375113470

Graphic Summary

Distribution of 103 Blast Hits on the Query Sequence

[?]

An overview of the database sequences aligned to the query sequence is shown. The score of each alignment is indicated by one of five different colors, which divides the range of scores into five groups. Multiple alignments on the same database sequence are connected by a striped line. Mousing over a hit sequence causes the definition and score to be shown in the window at the top, clicking on a hit sequence takes the user to the associated alignments. New: This graphic is an overview of database sequences aligned to the query sequence. Alignments are color-coded by score, within one of five score ranges. Multiple alignments on the same database sequence are connected by a dashed line. Mousing over an alignment shows the alignment definition and score in the box at the top. Clicking an alignment displays the alignment detail.

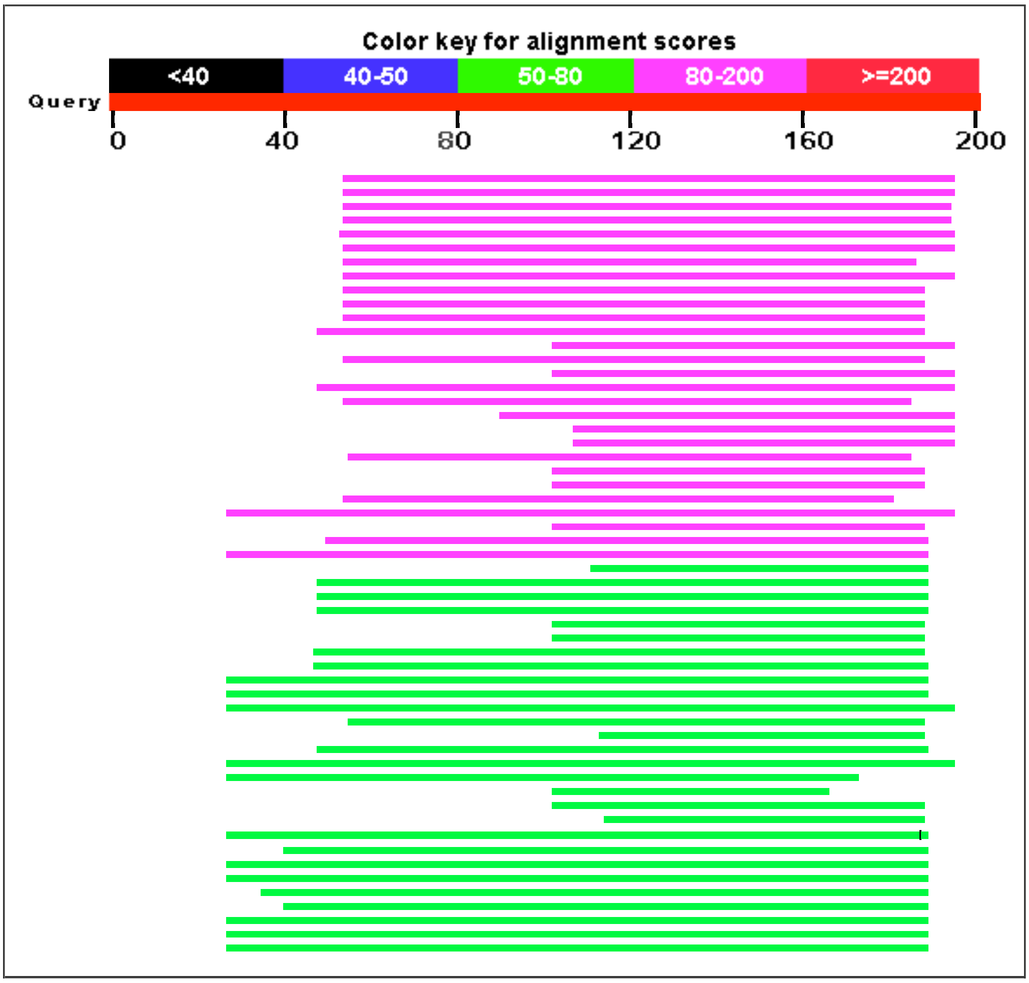

Descriptions

Legend for links to other resources: U UniGene E GEO G Gene S Structure M Map Viewer

Sequences producing significant alignments:
(Click headers to sort columns)

**AAGJ02119123.1**	Strongylocentrotus purpuratus Ctg119403, whole genome shotgun sequence	114	114	70%	6e-22	78%	
**AAGJ02135338.1**	Strongylocentrotus purpuratus Ctg135634, whole genome shotgun sequence	114	114	70%	6e-22	78%	
**AAGJ02037510.1**	Strongylocentrotus purpuratus Ctg37690, whole genome shotgun sequence	113	113	69%	2e-21	78%	
**AAGJ02153877.1**	Strongylocentrotus purpuratus Ctg154180, whole genome shotgun sequence	113	113	69%	2e-21	78%	
**AAGJ02006042.1**	Strongylocentrotus purpuratus Ctg6093, whole genome shotgun sequence	111	111	70%	7e-21	78%	G
**AAGJ02064279.1**	Strongylocentrotus purpuratus Ctg64483, whole genome shotgun sequence	109	109	70%	3e-20	78%	
**AAGJ02072849.1**	Strongylocentrotus purpuratus Ctg73060, whole genome shotgun sequence	102	102	65%	4e-18	78%	
**AAGJ02119842.1**	Strongylocentrotus purpuratus Ctg120124, whole genome shotgun sequence	102	102	70%	4e-18	77%	G
**AAGJ02145152.1**	Strongylocentrotus purpuratus Ctg145453, whole genome shotgun sequence	102	102	66%	4e-18	77%	
**AAGJ02150273.1**	Strongylocentrotus purpuratus Ctg150576, whole genome shotgun sequence	102	102	66%	4e-18	77%	
**AAGJ02150274.1**	Strongylocentrotus purpuratus Ctg150577, whole genome shotgun sequence	102	102	66%	4e-18	77%	
**AAGJ02114862.1**	Strongylocentrotus purpuratus Ctg115138, whole genome shotgun sequence	98.7	98.7	69%	5e-17	76%	G
**AAGJ02005132.1**	Strongylocentrotus purpuratus Ctg5180, whole genome shotgun sequence	96.9	96.9	46%	2e-16	82%	
**AAGJ02055927.1**	Strongylocentrotus purpuratus Ctg56123, whole genome shotgun sequence	96.9	96.9	66%	2e-16	77%	
**AAGJ02112265.1**	Strongylocentrotus purpuratus Ctg112541, whole genome shotgun sequence	96.9	96.9	46%	2e-16	82%	
**AAGJ02122414.1**	Strongylocentrotus purpuratus Ctg122698, whole genome shotgun sequence	96.9	96.9	73%	2e-16	75%	
**AAGJ02196717.1**	Strongylocentrotus purpuratus Ctg197221, whole genome shotgun sequence	96.9	157	65%	2e-16	84%	
**AAGJ02135744.1**	Strongylocentrotus purpuratus Ctg136040, whole genome shotgun sequence	93.3	93.3	52%	2e-15	80%	
**AAGJ02035393.1**	Strongylocentrotus purpuratus Ctg35570, whole genome shotgun sequence	91.5	91.5	43%	7e-15	82%	G
**AAGJ02037856.1**	Strongylocentrotus purpuratus Ctg38036, whole genome shotgun sequence	91.5	91.5	43%	7e-15	82%	G
**AAGJ02112289.1**	Strongylocentrotus purpuratus Ctg112565, whole genome shotgun sequence	89.7	89.7	64%	2e-14	76%	
**AAGJ02000190.1**	Strongylocentrotus purpuratus Ctg194, whole genome shotgun sequence	87.8	87.8	42%	9e-14	82%	
**AAGJ02016152.1**	Strongylocentrotus purpuratus Ctg16272, whole genome shotgun	87.8	87.8	42%	9e-14	82%	G

	sequence					
**AAGJ02022053.1**	Strongylocentrotus purpuratus Ctg22198, whole genome shotgun sequence	86.0	86.0	63%	3e-13	75%
**BAAE01263722.1**	Oryzias latipes DNA, contig263653 in scaffold23984, strain: HNI, whole genome shotgun sequence	84.2	84.2	83%	1e-12	71%
**AAGJ02094208.1**	Strongylocentrotus purpuratus Ctg94447, whole genome shotgun sequence	84.2	84.2	42%	1e-12	82%
**AASC02006447.1**	Aplysia californica cont2.6446, whole genome shotgun sequence	80.6	80.6	69%	1e-11	72%
**AASC02029508.1**	Aplysia californica cont2.29507, whole genome shotgun sequence	80.6	80.6	80%	1e-11	70%
**AASC02009772.1**	Aplysia californica cont2.9771, whole genome shotgun sequence	78.8	78.8	38%	4e-11	82%
**AASC02030911.1**	Aplysia californica cont2.30910, whole genome shotgun sequence	78.8	78.8	70%	4e-11	72%
**AASC02035229.1**	Aplysia californica cont2.35228, whole genome shotgun sequence	78.8	78.8	70%	4e-11	72%
**AASC02063867.1**	Aplysia californica cont2.63866, whole genome shotgun sequence	78.8	78.8	70%	4e-11	72%
**AAGJ02030884.1**	Strongylocentrotus purpuratus Ctg31050, whole genome shotgun sequence	78.8	78.8	42%	4e-11	80%
**AAGJ02112094.1**	Strongylocentrotus purpuratus Ctg112370, whole genome shotgun sequence	78.8	78.8	42%	4e-11	80%
**AAGJ02140935.1**	Strongylocentrotus purpuratus Ctg141235, whole genome shotgun sequence	78.8	78.8	70%	4e-11	72%
**AASC02024690.1**	Aplysia californica cont2.24689, whole genome shotgun sequence	77.0	77.0	70%	2e-10	71%
**AASC02032210.1**	Aplysia californica cont2.32209, whole genome shotgun sequence	77.0	77.0	80%	2e-10	70%
**AASC02040983.1**	Aplysia californica cont2.40982, whole genome shotgun sequence	77.0	77.0	80%	2e-10	70%
**BAAF04076102.1**	Oryzias latipes DNA, contig76102 in scaffold235, strain: Hd-rR, whole genome shotgun sequence	77.0	77.0	83%	2e-10	70%
**AAGJ02121827.1**	Strongylocentrotus purpuratus Ctg122110, whole genome shotgun sequence	77.0	77.0	66%	2e-10	74%
**AAGJ02190055.1**	Strongylocentrotus purpuratus Ctg190360, whole genome shotgun sequence	77.0	77.0	37%	2e-10	82%
**AASC02023390.1**	Aplysia californica cont2.23389, whole genome shotgun sequence	75.2	75.2	70%	5e-10	71%
**BAAE01046442.1**	Oryzias latipes DNA, contig46371 in scaffold1431, strain: HNI, whole genome shotgun sequence	75.2	75.2	83%	5e-10	70%
**BAAE01011739.1**	Oryzias latipes DNA, contig12122 in scaffold253, strain: HNI, whole genome shotgun sequence	75.2	75.2	72%	5e-10	71%
**AAGJ02012985.1**	Strongylocentrotus purpuratus Ctg13077, whole genome shotgun sequence	75.2	75.2	31%	5e-10	85%
**AAGJ02032830.1**	Strongylocentrotus purpuratus Ctg33001, whole genome shotgun sequence	75.2	75.2	42%	5e-10	79%
**AAGJ02122723.1**	Strongylocentrotus purpuratus Ctg123007, whole genome shotgun sequence	75.2	75.2	36%	5e-10	82%
**AASC02052968.1**	Aplysia californica cont2.52967, whole genome shotgun sequence	73.4	130	80%	2e-09	70%
**AASC02009483.1**	Aplysia californica cont2.9482, whole genome shotgun sequence	71.6	71.6	74%	7e-09	70%
**AASC02025249.1**	Aplysia californica cont2.25248, whole genome shotgun sequence	71.6	71.6	80%	7e-09	69%
**AASC02029272.1**	Aplysia californica cont2.29271, whole genome shotgun sequence	71.6	71.6	80%	7e-09	69%

**AASC02029386.1**	Aplysia californica cont2.29385, whole genome shotgun sequence	71.6	71.6	76%	7e-09	70%
**AASC02033525.1**	Aplysia californica cont2.33524, whole genome shotgun sequence	71.6	71.6	74%	7e-09	71%
**AASC02040771.1**	Aplysia californica cont2.40770, whole genome shotgun sequence	71.6	71.6	80%	7e-09	69%
**AASC02041111.1**	Aplysia californica cont2.41110, whole genome shotgun sequence	71.6	71.6	80%	7e-09	69%
**AASC02045802.1**	Aplysia californica cont2.45801, whole genome shotgun sequence	71.6	71.6	80%	7e-09	69%
**AASC02049491.1**	Aplysia californica cont2.49490, whole genome shotgun sequence	71.6	71.6	80%	7e-09	70%
**AASC02061593.1**	Aplysia californica cont2.61592, whole genome shotgun sequence	71.6	71.6	80%	7e-09	69%
**BAAF04071142.1**	Oryzias latipes DNA, contig71142 in scaffold204, strain: Hd-rR, whole genome shotgun sequence	71.6	71.6	83%	7e-09	69%
**BAAE01034390.1**	Oryzias latipes DNA, contig34339 in scaffold972, strain: HNI, whole genome shotgun sequence	71.6	71.6	83%	7e-09	70%
**ACQM01047541.1**	Saccoglossus kowalevskii Contig47657, whole genome shotgun sequence	69.8	69.8	35%	2e-08	81%
**AASC02028998.1**	Aplysia californica cont2.28997, whole genome shotgun sequence	69.8	69.8	62%	2e-08	72%
**AASC02048108.1**	Aplysia californica cont2.48107, whole genome shotgun sequence	69.8	69.8	73%	2e-08	70%
**BAAF04105806.1**	Oryzias latipes DNA, contig105806 in scaffold746, strain: Hd-rR, whole genome shotgun sequence	69.8	69.8	75%	2e-08	70%
**ACQM01054906.1**	Saccoglossus kowalevskii Contig55035, whole genome shotgun sequence	68.0	68.0	38%	8e-08	79%
**AASC02012961.1**	Aplysia californica cont2.12960, whole genome shotgun sequence	68.0	68.0	80%	8e-08	69%
**AASC02016796.1**	Aplysia californica cont2.16795, whole genome shotgun sequence	68.0	68.0	80%	8e-08	69%
**AASC02036896.1**	Aplysia californica cont2.36895, whole genome shotgun sequence	68.0	68.0	80%	8e-08	69%
**AASC02040053.1**	Aplysia californica cont2.40052, whole genome shotgun sequence	68.0	68.0	80%	8e-08	69%
**AASC02043334.1**	Aplysia californica cont2.43333, whole genome shotgun sequence	68.0	114	75%	8e-08	75%
**AASC02051169.1**	Aplysia californica cont2.51168, whole genome shotgun sequence	68.0	68.0	80%	8e-08	69%
**AASC02059661.1**	Aplysia californica cont2.59660, whole genome shotgun sequence	68.0	68.0	80%	8e-08	69%
**BAAF04001490.1**	Oryzias latipes DNA, contig1490 in scaffold2, strain: Hd-rR, whole genome shotgun sequence	68.0	68.0	92%	8e-08	68%
**BAAF04083036.1**	Oryzias latipes DNA, contig83036 in scaffold286, strain: Hd-rR, whole genome shotgun sequence	68.0	68.0	80%	8e-08	69%
**BAAF04102545.1**	Oryzias latipes DNA, contig102545 in scaffold615, strain: Hd-rR, whole genome shotgun sequence	68.0	68.0	89%	8e-08	68%
**BAAF04112029.1**	Oryzias latipes DNA, contig112029 in scaffold1180, strain: Hd-rR, whole genome shotgun sequence	68.0	68.0	89%	8e-08	68%
**BAAE01274428.1**	Oryzias latipes DNA, contig274338 in scaffold27113, strain: HNI, whole genome shotgun sequence	68.0	68.0	89%	8e-08	68%
**BAAE01229859.1**	Oryzias latipes DNA, contig229807 in scaffold16894, strain: HNI, whole genome shotgun sequence	68.0	68.0	89%	8e-08	68%
**BAAE01149167.1**	Oryzias latipes DNA, contig149040 in scaffold7529, strain: HNI, whole genome shotgun sequence	68.0	68.0	89%	8e-08	68%
**BAAE01154250.1**	Oryzias latipes DNA, contig154165 in scaffold7957, strain: HNI, whole genome shotgun sequence	68.0	68.0	89%	8e-08	68%

**BAAE01123166.1**	Oryzias latipes DNA, contig123078 in scaffold5559, strain: HNI, whole genome shotgun sequence	68.0	68.0	89%	8e-08	68%	
**BAAE01123168.1**	Oryzias latipes DNA, contig123080 in scaffold5559, strain: HNI, whole genome shotgun sequence	68.0	68.0	89%	8e-08	68%	
**BAAE01093172.1**	Oryzias latipes DNA, contig92264 in scaffold3686, strain: HNI, whole genome shotgun sequence	68.0	68.0	89%	8e-08	68%	
**BAAE01079160.1**	Oryzias latipes DNA, contig79199 in scaffold2924, strain: HNI, whole genome shotgun sequence	68.0	68.0	39%	8e-08	78%	
**BAAE01049990.1**	Oryzias latipes DNA, contig49924 in scaffold1580, strain: HNI, whole genome shotgun sequence	68.0	68.0	80%	8e-08	69%	
**AAGJ02119249.1**	Strongylocentrotus purpuratus Ctg119529, whole genome shotgun sequence	68.0	68.0	37%	8e-08	80%	G
**AAGJ02135339.1**	Strongylocentrotus purpuratus Ctg135635, whole genome shotgun sequence	68.0	68.0	34%	8e-08	81%	
**ACQM01008863.1**	Saccoglossus kowalevskii Contig8891, whole genome shotgun sequence	66.2	66.2	35%	3e-07	80%	
**ACQM01064549.1**	Saccoglossus kowalevskii Contig64697, whole genome shotgun sequence	66.2	66.2	35%	3e-07	80%	
**ACQM01132338.1**	Saccoglossus kowalevskii Contig132621, whole genome shotgun sequence	66.2	66.2	35%	3e-07	80%	
**AASC02012236.1**	Aplysia californica cont2.12235, whole genome shotgun sequence	66.2	66.2	70%	3e-07	70%	
**AASC02013680.1**	Aplysia californica cont2.13679, whole genome shotgun sequence	66.2	66.2	70%	3e-07	70%	
**AASC02013687.1**	Aplysia californica cont2.13686, whole genome shotgun sequence	66.2	66.2	32%	3e-07	81%	
**AASC02020422.1**	Aplysia californica cont2.20421, whole genome shotgun sequence	66.2	66.2	69%	3e-07	70%	
**AASC02024688.1**	Aplysia californica cont2.24687, whole genome shotgun sequence	66.2	66.2	73%	3e-07	70%	
**AASC02031740.1**	Aplysia californica cont2.31739, whole genome shotgun sequence	66.2	66.2	39%	3e-07	78%	
**AASC02043777.1**	Aplysia californica cont2.43776, whole genome shotgun sequence	66.2	66.2	70%	3e-07	70%	
**AASC02048297.1**	Aplysia californica cont2.48296, whole genome shotgun sequence	66.2	66.2	80%	3e-07	69%	
**AASC02062985.1**	Aplysia californica cont2.62984, whole genome shotgun sequence	66.2	66.2	32%	3e-07	81%	
**BAAF04033128.1**	Oryzias latipes DNA, contig33128 in scaffold54, strain: Hd-rR, whole genome shotgun sequence	66.2	66.2	75%	3e-07	70%	

Alignments Select All Get selected sequences Distance tree of results Multiple alignment NEW

```
>gb|AAGJ02119123.1|  Strongylocentrotus purpuratus Ctg119403, whole genome shotgun
sequence
Length=33468

 Score =  114 bits (126),  Expect = 6e-22
 Identities = 112/142 (78%), Gaps = 2/142 (1%)
 Strand=Plus/Minus

Query  55    AGAAGACGCTGGAGGTGGATTCGGCATGTCCTACGGAAAGGCCAGGGG-GATATCACAAA  113
             |||||| | ||||| |||||  |||||||  |  | ||||| ||| |  || ||||||
Sbjct  9287  AGAAGAAGGTGGAGATGGATCGGGCATGTAATGAG-AAAGGACAGAGATGACATCACACG  9229

Query  114   AACAGCACTCCACTGGACCCCAGAAGGTAAAAGAAAGAGAGGAAGACCTAAAATGACATG  173
             |||||| ||||||||||| |||||||| ||||||||||| ||| | |||||||  || ||
Sbjct  9228  AACAGCCCTCCACTGGACACCAGAAGGCAAAAGAAAGAGGGGACGTCCTAAAACCACTTG  9169

Query  174   GAGAAGAACTGTAGAGGCAGAG  195
             | |||||||||| || | ||||
Sbjct  9168  GCGAAGAACTGTGGAAGGAGAG  9147


>gb|AAGJ02135338.1|  Strongylocentrotus purpuratus Ctg135634, whole genome shotgun
sequence
Length=1111

 Score =  114 bits (126),  Expect = 6e-22
 Identities = 112/142 (78%), Gaps = 2/142 (1%)
 Strand=Plus/Plus

Query  55   AGAAGACGCTGGAGGTGGATTCGGCATGTCCTACGGAAAGGCCAG-GGGGATATCACAAA  113
            |||||| | ||||| |||||  |||||||  |    |||||| ||| |  || ||||||
Sbjct  196  AGAAGAAGGTGGAGATGGATCGGGCATGTAAT-GAGAAAGGACAGAGATGACATCACACG  254

Query  114  AACAGCACTCCACTGGACCCCAGAAGGTAAAAGAAAGAGAGGAAGACCTAAAATGACATG  173
            |||||| ||||||||||| |||||||| ||||||||||| ||| | |||||||  || ||
Sbjct  255  AACAGCCCTCCACTGGACACCAGAAGGCAAAAGAAAGAGGGGACGTCCTAAAACCACTTG  314

Query  174  GAGAAGAACTGTAGAGGCAGAG  195
            | |||||||||| || | ||||
Sbjct  315  GCGAAGAACTGTGGAAGGAGAG  336


>gb|AAGJ02037510.1|  Strongylocentrotus purpuratus Ctg37690, whole genome shotgun
sequence
Length=1481

 Score =  113 bits (124),  Expect = 2e-21
 Identities = 111/141 (78%), Gaps = 2/141 (1%)
 Strand=Plus/Minus

Query  55   AGAAGACGCTGGAGGTGGATTCGGCATGTCCTACGGAAAGGCCAGGGG-GATATCACAAA  113
            |||||| | ||||| |||||  |||||||  |  | ||||| ||| |  || ||||||
Sbjct  420  AGAAGAAGGTGGAGATGGATCGGGCATGTAATGAG-AAAGGACAGAGATGACATCACACG  362

Query  114  AACAGCACTCCACTGGACCCCAGAAGGTAAAAGAAAGAGAGGAAGACCTAAAATGACATG  173
            |||||| ||||||||||| |||||||| ||||||||||| ||| | |||||||  || ||
Sbjct  361  AACAGCCCTCCACTGGACACCAGAAGGCAAAAGAAAGAGGGGACGTCCTAAAACCACTTG  302

Query  174  GAGAAGAACTGTAGAGGCAGA  194
            | |||||||||| || | |||
Sbjct  301  GCGAAGAACTGTGGAAGGAGA  281


>gb|AAGJ02153877.1|  Strongylocentrotus purpuratus Ctg154180, whole genome shotgun
sequence
Length=1074

 Score =  113 bits (124),  Expect = 2e-21
 Identities = 111/141 (78%), Gaps = 2/141 (1%)
 Strand=Plus/Plus

Query  55   AGAAGACGCTGGAGGTGGATTCGGCATGTCCTACGGAAAGGCCAG-GGGGATATCACAAA  113
            |||||| | ||||| |||||  |||||||  |    |||||| ||| |  || ||||||
Sbjct  383  AGAAGAAGGTGGAGATGGATCGGGCATGTAAT-GAGAAAGGACAGAGATGACATCACACG  441

Query  114  AACAGCACTCCACTGGACCCCAGAAGGTAAAAGAAAGAGAGGAAGACCTAAAATGACATG  173
            |||||| ||||||||||| |||||||| ||||||||||| ||| | |||||||  || ||
Sbjct  442  AACAGCCCTCCACTGGACACCAGAAGGCAAAAGAAAGAGGGGACGTCCTAAAACCACTTG  501
```

```
Query  174  GAGAAGAACTGTAGAGGCAGA  194
            | |||||||||| || | |||
Sbjct  502  GCGAAGAACTGTGGAAGGAGA  522
```

>**gb|AAGJ02006042.1|** Strongylocentrotus purpuratus Ctg6093, whole genome shotgun sequence
Length=6423

GENE ID: 592447 LOC592447 | hypothetical LOC592447
[Strongylocentrotus purpuratus]

Score = 111 bits (122), Expect = 7e-21
Identities = 112/143 (78%), Gaps = 2/143 (1%)
Strand=Plus/Minus

```
Query  54    AAGAAGACGCTGGAGGTGGATTCGGCATGTCCTACGGAAAGGCCAGGGG-GATATCACAA  112
             ||||||| | ||||| |||||  |||||||  |  | ||||| ||| |  || |||| |
Sbjct  6327  AAGAAGAAGGTGGAGATGGATCGGGCATGTAATGAG-AAAGGACAGAGATGACATCATAC  6269

Query  113   AAACAGCACTCCACTGGACCCCAGAAGGTAAAAGAAAGAGAGGAAGACCTAAAATGACAT  172
              |||||| ||||||||||| |||||||| ||||||||||| ||| | |||||||  || |
Sbjct  6268  GAACAGCCCTCCACTGGACACCAGAAGGCAAAAGAAAGAGGGGACGTCCTAAAACCACTT  6209

Query  173   GGAGAAGAACTGTAGAGGCAGAG  195
             || |||||||||| || | ||||
Sbjct  6208  GGCGAAGAACTGTGGAAGGAGAG  6186
```

>**gb|AAGJ02064279.1|** Strongylocentrotus purpuratus Ctg64483, whole genome shotgun sequence
Length=894

Score = 109 bits (120), Expect = 3e-20
Identities = 111/142 (78%), Gaps = 2/142 (1%)
Strand=Plus/Minus

```
Query  55   AGAAGACGCTGGAGGTGGATTCGGCATGTCCTACGGAAAGGCCAGGGG-GATATCACAAA  113
            |||||| | ||||| |||||  |||||||  |  | ||||| ||| |  || ||||||
Sbjct  445  AGAAGAAGGTGGAGATGGATCGGGCATGTAGTGAG-AAAGGACAGAGATGACATCACACG  387

Query  114  AACAGCACTCCACTGGACCCCAGAAGGTAAAAGAAAGAGAGGAAGACCTAAAATGACATG  173
            |||||| ||||||||||| |||||||| ||||||| ||| ||| | |||||||  || ||
Sbjct  386  AACAGCCCTCCACTGGACACCAGAAGGCAAAAGAACGAGGGGACGTCCTAAAACCACTTG  327

Query  174  GAGAAGAACTGTAGAGGCAGAG  195
            | |||||||||| || | ||||
Sbjct  326  GCGAAGAACTGTGGAAGGAGAG  305
```

>**gb|AAGJ02072849.1|** Strongylocentrotus purpuratus Ctg73060, whole genome shotgun sequence
Length=1359

Score = 102 bits (112), Expect = 4e-18
Identities = 104/133 (78%), Gaps = 2/133 (1%)
Strand=Plus/Minus

```
Query  55   AGAAGACGCTGGAGGTGGATTCGGCATGTCCTACGGAAAGGCCAGGGG-GATATCACAAA  113
            |||||| | ||||| |||||  |||||||  |  | ||||  ||| |  || ||||||
Sbjct  848  AGAAGAAGGTGGAGATGGATCGGGCATGTAATGAG-AAAGAACAGAGATGACATCACACG  790

Query  114  AACAGCACTCCACTGGACCCCAGAAGGTAAAAGAAAGAGAGGAAGACCTAAAATGACATG  173
            |||||| ||||||||||| |||||||| ||||||||||| ||| | |||||||  || ||
Sbjct  789  AACAGCCCTCCACTGGACACCAGAAGGCAAAAGAAAGAGGGGACGTCCTAAAACCACTTG  730

Query  174  GAGAAGAACTGTA  186
            | ||||||| |||
Sbjct  729  GCGAAGAACAGTA  717
```

>**gb|AAGJ02119842.1|** Strongylocentrotus purpuratus Ctg120124, whole genome shotgun sequence
Length=6747

GENE ID: 583534 LOC583534 | similar to SAM domain- and HD domain-containing protein 1 [Strongylocentrotus purpuratus]

Score = 102 bits (112), Expect = 4e-18
Identities = 111/144 (77%), Gaps = 4/144 (2%)
Strand=Plus/Plus

```
Query  55    AGAAGACGCTGGAGGTGGATTCGGCATGTCCTACGGAAAGGCCAG-GGGGATATCACAAA  113
             |||||| | ||||| |||||  |||||||  |   |||||| ||| |  || ||||||
Sbjct  5125  AGAAGAAGGTGGAGATGGATCGGGCATGTAAT-GAGAAAGGACAGAGATGACATCACACG  5183

Query  114   AACAGCACTCCACTGGACCCCAGAAGG--TAAAAGAAAGAGAGGAAGACCTAAAATGACA  171
             |||||| ||||||||||| ||||||||   |||| |||||| ||| | |||||||  ||
Sbjct  5184  AACAGCCCTCCACTGGACACCAGAAGGCAAAAAAAAAAGAGGGGACGTCCTAAAACCACT  5243

Query  172   TGGAGAAGAACTGTAGAGGCAGAG  195
             ||| |||||||||| || | ||||
Sbjct  5244  TGGCGAAGAACTGTGGAAGGAGAG  5267
```

>**gb|AAGJ02145152.1|** Strongylocentrotus purpuratus Ctg145453, whole genome shotgun sequence
Length=10802

```
 Score =  102 bits (112),  Expect = 4e-18
 Identities = 105/135 (77%), Gaps = 2/135 (1%)
 Strand=Plus/Minus

Query  55    AGAAGACGCTGGAGGTGGATTCGGCATGTCCTACGGAAAGGCCAGGGG-GATATCACAAA  113
             |||||| | ||||| |||||  |||||||  |  | ||||  ||| |  || ||||||
Sbjct  2508  AGAAGAAGGTGGAGATGGATCGGGCATGTAATGAG-AAAGAACAGAGATGACATCACACG  2450

Query  114   AACAGCACTCCACTGGACCCCAGAAGGTAAAAGAAAGAGAGGAAGACCTAAAATGACATG  173
             |||||| ||||||||||| |||||||| ||||||||||| ||| | |||||||  || ||
Sbjct  2449  AACAGCCCTCCACTGGACACCAGAAGGCAAAAGAAAGAGGGGACGTCCTAAAACCACTTG  2390

Query  174   GAGAAGAACTGTAGA  188
               |||||||||| ||
Sbjct  2389  ACGAAGAACTGTGGA  2375
```

>**gb|AAGJ02150273.1|** Strongylocentrotus purpuratus Ctg150576, whole genome shotgun sequence
Length=6491

```
 Score =  102 bits (112),  Expect = 4e-18
 Identities = 105/135 (77%), Gaps = 2/135 (1%)
 Strand=Plus/Plus

Query  55    AGAAGACGCTGGAGGTGGATTCGGCATGTCCTACGGAAAGGCCAGGGG-GATATCACAAA  113
             |||||| | ||||| |||||  |||||||  |  | ||||  ||| |  || ||||||
Sbjct  1317  AGAAGAAGGTGGAGATGGATCGGGCATGTAATGAG-AAAGAACAGAGATGACATCACACG  1375

Query  114   AACAGCACTCCACTGGACCCCAGAAGGTAAAAGAAAGAGAGGAAGACCTAAAATGACATG  173
             |||||| ||||||||||| |||||||| ||||||||||  ||| | |||||||  || ||
Sbjct  1376  AACAGCCCTCCACTGGACACCAGAAGGCAAAAGAAAGAAGGGACGTCCTAAAACCACTTG  1435

Query  174   GAGAAGAACTGTAGA  188
             | |||||||||| ||
Sbjct  1436  GCGAAGAACTGTGGA  1450
```

>**gb|AAGJ02150274.1|** D Strongylocentrotus purpuratus Ctg150577, whole genome shotgun sequence
Length=12214

```
 Score =  102 bits (112),  Expect = 4e-18
 Identities = 105/135 (77%), Gaps = 2/135 (1%)
 Strand=Plus/Minus

Query  55    AGAAGACGCTGGAGGTGGATTCGGCATGTCCTACGGAAAGGCCAGGGG-GATATCACAAA  113
             |||||| | ||||| |||||  |||||||  |  | ||||  ||| |  || ||||||
Sbjct  9884  AGAAGAAGGTGGAGATGGATCGGGCATGTAATGAG-AAAGAACAGAGATGACATCACACG  9826

Query  114   AACAGCACTCCACTGGACCCCAGAAGGTAAAAGAAAGAGAGGAAGACCTAAAATGACATG  173
             |||||| ||||||||||| |||||||| ||||||||||  ||| | |||||||  || ||
Sbjct  9825  AACAGCCCTCCACTGGACACCAGAAGGCAAAAGAAAGAAGGGACGTCCTAAAACCACTTG  9766

Query  174   GAGAAGAACTGTAGA  188
             | |||||||||| ||
Sbjct  9765  GCGAAGAACTGTGGA  9751
```

>**gb|AAGJ02114862.1|** G Strongylocentrotus purpuratus Ctg115138, whole genome shotgun sequence
Length=6131

 GENE ID: 592256 LOC592256 | similar to p21 (CDKN1A)-activated kinase 1
[Strongylocentrotus purpuratus]

```
 Score = 98.7 bits (108),  Expect = 5e-17
 Identities = 108/141 (76%), Gaps = 2/141 (1%)
 Strand=Plus/Plus

Query  49    ATAATAAGAAGACGCTGGAGGTGGATTCGGCATGTCCTACGGAAAGGCCAGGGG-GATAT  107
             || || |||||| | ||||| |||||| | |||||  |  | ||||  ||| |  || ||
Sbjct  4540  ATCATGAGAAGAAGGTGGAGATGGATTGGACATGTAATGAG-AAAGAACAGAGATGACAT  4598

Query  108   CACAAAAACAGCACTCCACTGGACCCCAGAAGGTAAAAGAAAGAGAGGAAGACCTAAAAT  167
             ||||  |||||| ||||||||||| ||| |||| |||||| |||| ||| | |||||||
Sbjct  4599  CACACGAACAGCCCTCCACTGGACACCAAAAGGCAAAAGAGAGAGGGGACGTCCTAAAAA  4658

Query  168   GACATGGAGAAGAACTGTAGA  188
              || ||| |||||||||| ||
Sbjct  4659  CACTTGGCGAAGAACTGTGGA  4679
```

>**gb|AAGJ02005132.1|** Strongylocentrotus purpuratus Ctg5180, whole genome shotgun sequence
Length=6203

```
 Score = 96.9 bits (106),  Expect = 2e-16
 Identities = 77/93 (82%), Gaps = 0/93 (0%)
 Strand=Plus/Minus

Query  103   GATATCACAAAAACAGCACTCCACTGGACCCCAGAAGGTAAAAGAAAGAGAGGAAGACCT  162
             || ||||||  |||||| ||||||||||| ||||| || ||||||||||| ||||| |||
Sbjct  3548  GACATCACACGAACAGCCCTCCACTGGACACCAGAGGGCAAAAGAAAGAGGGGAAGCCCT  3489

Query  163   AAAATGACATGGAGAAGAACTGTAGAGGCAGAG  195
             ||||  || ||| |||||||||| || | ||||
Sbjct  3488  AAAAACACTTGGCGAAGAACTGTGGAAGGAGAG  3456
```

>**gb|AAGJ02055927.1|** Strongylocentrotus purpuratus Ctg56123, whole genome shotgun sequence
Length=1223

```
 Score = 96.9 bits (106),  Expect = 2e-16
 Identities = 104/135 (77%), Gaps = 2/135 (1%)
 Strand=Plus/Minus

Query  55   AGAAGACGCTGGAGGTGGATTCGGCATGTCCTACGGAAAGGCCAGGGG-GATATCACAAA  113
            |||||| | ||||| |||||  ||||| |  |  | ||||  ||| |  || ||||||
Sbjct  832  AGAAGAAGGTGGAGATGGATCGGGCATTTAATGAG-AAAGAACAGAGATGACATCACACG  774

Query  114  AACAGCACTCCACTGGACCCCAGAAGGTAAAAGAAAGAGAGGAAGACCTAAAATGACATG  173
            |||||| |||||||||||  ||||||| ||||||||||| ||| | |||||||  || ||
Sbjct  773  AACAGCCCTCCACTGGACAACAGAAGGCAAAAGAAAGAGGGGACGTCCTAAAACCACTTG  714

Query  174  GAGAAGAACTGTAGA  188
            | |||||||||| ||
Sbjct  713  GCGAAGAACTGTGGA  699
```

>**gb|AAGJ02112265.1|** D Strongylocentrotus purpuratus Ctg112541, whole genome shotgun sequence
Length=11131

```
 Score = 96.9 bits (106),  Expect = 2e-16
 Identities = 77/93 (82%), Gaps = 0/93 (0%)
 Strand=Plus/Plus

Query  103   GATATCACAAAAACAGCACTCCACTGGACCCCAGAAGGTAAAAGAAAGAGAGGAAGACCT  162
             || ||||||  |||||| ||||||||||| ||||| || ||||||||||| ||||| |||
Sbjct  2795  GACATCACACGAACAGCCCTCCACTGGACACCAGAGGGCAAAAGAAAGAGGGGAAGCCCT  2854

Query  163   AAAATGACATGGAGAAGAACTGTAGAGGCAGAG  195
             ||||  || ||| |||||||||| || | ||||
Sbjct  2855  AAAAACACTTGGCGAAGAACTGTGGAAGGAGAG  2887
```

>**gb|AAGJ02122414.1|** Strongylocentrotus purpuratus Ctg122698, whole genome shotgun sequence
Length=3590

```
 Score = 96.9 bits (106),  Expect = 2e-16
 Identities = 111/148 (75%), Gaps = 1/148 (0%)
 Strand=Plus/Minus

Query  49    ATAATAAGAAGACGCTGGAGGTGGATTCGGCATGTCCTACGGAAAGGCCAGGGG-GATAT  107
             || || |||||| | | ||| |||||  | |||||  |  | |||   ||  |  || ||
Sbjct  1086  ATCATGAGAAGAAGGTCGAGATGGATCGGACATGTATTGAGAAAAAAACATAGATGACAT  1027

Query  108   CACAAAAACAGCACTCCACTGGACCCCAGAAGGTAAAAGAAAGAGAGGAAGACCTAAAAT  167
             ||||  |||||| ||||||||||| |||||||| ||||||||||| ||| | |||||||
Sbjct  1026  CACACGAACAGCCCTCCACTGGACACCAGAAGGCAAAAGAAAGAGGGGACGTCCTAAAAA  967

Query  168   GACATGGAGAAGAACTGTAGAGGCAGAG  195
              || ||| |||||||||| || | ||||
Sbjct  966   CACTTGGCGAAGAACTGTGGAAGGAGAG  939
```

>**gb|AAGJ02196717.1|** Strongylocentrotus purpuratus Ctg197221, whole genome shotgun sequence
Length=640

Sort alignments for this subject sequence by:
E value **Score** **Percent identity**
Query start position **Subject start position**

```
 Score = 96.9 bits (106),  Expect = 2e-16
 Identities = 102/132 (77%), Gaps = 2/132 (1%)
 Strand=Plus/Minus

Query  55   AGAAGACGCTGGAGGTGGATTCGGCATGTCCTACGGAAAGGCCAGGGG-GATATCACAAA  113
            |||||| | ||||| |||||  | |||||  |  | ||||  ||  |  || ||||||
Sbjct  339  AGAAGAAGGTGGAGATGGATCGGACATGTAATGAG-AAAGAACATAGATGACATCACACG  281

Query  114  AACAGCACTCCACTGGACCCCAGAAGGTAAAAGAAAGAGAGGAAGACCTAAAATGACATG  173
            |||||| ||||||||||| |||||||| ||||||||||| ||| | |||||||  || ||
Sbjct  280  AACAGCCCTCCACTGGACACCAGAAGGCAAAAGAAAGAGGGGACGTCCTAAAAACACTTG  221

Query  174  GAGAAGAACTGT  185
            | ||||||||||
Sbjct  220  GCGAAGAACTGT  209


 Score = 60.8 bits (66),  Expect = 1e-05
 Identities = 45/53 (84%), Gaps = 0/53 (0%)
 Strand=Plus/Minus

Query  133  CCAGAAGGTAAAAGAAAGAGAGGAAGACCTAAAATGACATGGAGAAGAACTGT  185
            |||||||| ||||||||||| ||| | |||||||  || ||| ||||||||||
Sbjct  638  CCAGAAGGCAAAAGAAAGAGGGGACGTCCTAAAAACACTTGGCGAAGAACTGT  586
```

>**gb|AAGJ02135744.1|** D Strongylocentrotus purpuratus Ctg136040, whole genome shotgun sequence
Length=15396

```
 Score = 93.3 bits (102),  Expect = 2e-15
 Identities = 85/106 (80%), Gaps = 1/106 (0%)
 Strand=Plus/Minus

Query  91    AAAGGCCAGGGG-GATATCACAAAAACAGCACTCCACTGGACCCCAGAAGGTAAAAGAAA  149
             ||||| ||| |  || ||||||  |||||| ||||||||||| |||||||| ||||||||
Sbjct  6746  AAAGGACAGAGATGACATCACACGAACAGCCCTCCACTGGACACCAGAAGGCAAAAGAAA  6687

Query  150   GAGAGGAAGACCTAAAATGACATGGAGAAGAACTGTAGAGGCAGAG  195
             ||| ||| | |||||||  || ||| ||||||| || || | ||||
Sbjct  6686  GAGGGGACGTCCTAAAACCACTTGGCGAAGAACCGTGGAAGGAGAG  6641
```

>gb|AAGJ02035393.1| Strongylocentrotus purpuratus Ctg35570, whole genome shotgun sequence
Length=14986

GENE ID: 584275 LOC584275 | similar to ENSANGP00000016497
[Strongylocentrotus purpuratus]

```
 Score = 91.5 bits (100),  Expect = 7e-15
 Identities = 73/88 (82%), Gaps = 0/88 (0%)
 Strand=Plus/Plus

Query  108   CACAAAAACAGCACTCCACTGGACCCCAGAAGGTAAAAGAAAGAGAGGAAGACCTAAAAT  167
             ||||  |||||| ||||||||||| |||||||| ||||||||||| ||| | ||||||| 
Sbjct  9330  CACACGAACAGCCCTCCACTGGACACCAGAAGGCAAAAGAAAGAGGGGACGTCCTAAAAC  9389

Query  168   GACATGGAGAAGAACTGTAGAGGCAGAG  195
              || ||| ||||||||||  || | ||||
Sbjct  9390  CACTTGGCGAAGAACTGTGGAAGGAGAG  9417
```

>gb|AAGJ02037856.1| Strongylocentrotus purpuratus Ctg38036, whole genome shotgun sequence
Length=14654

```
 Score = 91.5 bits (100),  Expect = 7e-15
 Identities = 73/88 (82%), Gaps = 0/88 (0%)
 Strand=Plus/Minus

Query  108   CACAAAAACAGCACTCCACTGGACCCCAGAAGGTAAAAGAAAGAGAGGAAGACCTAAAAT  167
             ||||  |||||| ||||||||||| |||||||| ||||||||||| ||| | ||||||| 
Sbjct  2721  CACACGAACAGCCCTCCACTGGACACCAGAAGGCAAAAGAAAGAGGGGACGTCCTAAAAC  2662

Query  168   GACATGGAGAAGAACTGTAGAGGCAGAG  195
              || ||| ||||||||||  || | ||||
Sbjct  2661  CACTTGGCGAAGAACTGTGGAAGGAGAG  2634
```

>gb|AAGJ02112289.1| Strongylocentrotus purpuratus Ctg112565, whole genome shotgun sequence
Length=7468

```
 Score = 89.7 bits (98),  Expect = 2e-14
 Identities = 101/132 (76%), Gaps = 4/132 (3%)
 Strand=Plus/Plus

Query  56    GAAGACGCTGGAGGTGGATTCGGCATGTCCTACGGAAAGGC-CAGGGG-GATATCACAAA  113
             ||||| | ||||| |||||  | |||||  || |  ||||  ||  |  || ||||||  
Sbjct  5522  GAAGAAGGTGGAGATGGATCGGACATGT--TATGAGAAGGAACATAGATGACATCACACG  5579

Query  114   AACAGCACTCCACTGGACCCCAGAAGGTAAAAGAAAGAGAGGAAGACCTAAAATGACATG  173
             |||||| ||||||||||| |||||||| |||| |||||| ||| | |||||||  || ||
Sbjct  5580  AACAGCCCTCCACTGGACACCAGAAGGCAAAATAAAGAGGGGACGTCCTAAAAACACTTG  5639

Query  174   GAGAAGAACTGT  185
             | ||||||||||
Sbjct  5640  GCGAAGAACTGT  5651
```

>gb|AAGJ02000190.1| Strongylocentrotus purpuratus Ctg194, whole genome shotgun sequence
Length=5889

```
 Score = 87.8 bits (96),  Expect = 9e-14
 Identities = 71/86 (82%), Gaps = 0/86 (0%)
 Strand=Plus/Minus

Query  103   GATATCACAAAAACAGCACTCCACTGGACCCCAGAAGGTAAAAGAAAGAGAGGAAGACCT  162
             || ||||||  |||||| ||||| ||||| |||||||| ||||||||||| ||| | |||
Sbjct  1591  GACATCACACGAACAGCCCTCCATTGGACACCAGAAGGCAAAAGAAAGAGGGGACGTCCT  1532

Query  163   AAAATGACATGGAGAAGAACTGTAGA  188
             ||||  || ||| |||||||||| ||
Sbjct  1531  AAAAACACTTGGCGAAGAACTGTGGA  1506
```

>gb|AAGJ02016152.1| Strongylocentrotus purpuratus Ctg16272, whole genome shotgun sequence
Length=8937

GENE ID: 762641 LOC762641 | hypothetical protein LOC762641
[Strongylocentrotus purpuratus]

```
 Score = 87.8 bits (96),  Expect = 9e-14
 Identities = 71/86 (82%), Gaps = 0/86 (0%)
 Strand=Plus/Minus
```

```
Query  103   GATATCACAAAAACAGCACTCCACTGGACCCCAGAAGGTAAAAGAAAGAGAGGAAGACCT  162
             || ||||||  |||||| ||||||||||| |||||||| ||||||||||| ||| | |||
Sbjct  6727  GACATCACACGAACAGCCCTCCACTGGACACCAGAAGGCAAAAGAAAGAGGGGACGTCCT  6668

Query  163   AAAATGACATGGAGAAGAACTGTAGA  188
             ||||  || ||  |||||||||| ||
Sbjct  6667  AAAAACACTTGCCGAAGAACTGTGGA  6642
```

>**gb|AAGJ02022053.1|** Strongylocentrotus purpuratus Ctg22198, whole genome shotgun sequence
Length=21601

```
 Score = 86.0 bits (94),  Expect = 3e-13
 Identities = 96/127 (75%), Gaps = 1/127 (0%)
 Strand=Plus/Minus

Query  55   AGAAGACGCTGGAGGTGGATTCGGCATGTCCTACGGAAAGGCCAGGGGGATATCACAAAA  114
            || ||| | ||||| |||||  | |||||  |  | |||| | | |  || ||||||  |
Sbjct  559  AGGAGAAGGTGGAGATGGATCGGACATGTAATGAG-AAAGACAAAGATGACATCACACGA  501

Query  115  ACAGCACTCCACTGGACCCCAGAAGGTAAAAGAAAGAGAGGAAGACCTAAAATGACATGG  174
            ||||| ||||||||||| |||||||| || |||||||| ||| | |||||||  || |||
Sbjct  500  ACAGCCCTCCACTGGACACCAGAAGGCAACAGAAAGAGGGGACGTCCTAAAAACACTTGG  441

Query  175  AGAAGAA  181
             ||||||
Sbjct  440  CGAAGAA  434
```

>**dbj|BAAE01263722.1|** Oryzias latipes DNA, contig263653 in scaffold23984, strain: HNI, whole genome shotgun sequence
Length=1148

```
 Score = 84.2 bits (92),  Expect = 1e-12
 Identities = 122/171 (71%), Gaps = 6/171 (3%)
 Strand=Plus/Minus

Query  28   CAACAAGATATAGCCACAACTATAATAAGAAGACGCTGGAGGTGGATTCGGCATGTCCTA  87
            ||| |||| ||  | |||| |||  | | |||||||||||  |||||| | ||||| ||
Sbjct  219  CAAGAAGACATGTCAACAATTATCTTCAAAAGACGCTGGATATGGATTGGACATGTACTC  160

Query  88   CGGAAAGGCCAGGGGGATA---TCACAAAAACAGCACTCCACTGGACCCCAGAAGGTAAA  144
             |||   | || |   | |   ||| ||| |||||||| |||||| | ||||| || |||
Sbjct  159  AGGA---GTCAAGAAAACACGATCATAAAGACAGCACTACACTGGGCACCAGATGGAAAA  103

Query  145  AGAAAGAGAGGAAGACCTAAAATGACATGGAGAAGAACTGTAGAGGCAGAG  195
            ||||| ||||||   || || || || ||| | ||||| ||||| || |||
Sbjct  102  AGAAAAAGAGGACAGCCGAAGATCACCTGGCGCAGAACAGTAGAAGCTGAG  52
```

>**gb|AAGJ02094208.1|** Strongylocentrotus purpuratus Ctg94447, whole genome shotgun sequence
Length=5041

```
 Score = 84.2 bits (92),  Expect = 1e-12
 Identities = 71/86 (82%), Gaps = 1/86 (1%)
 Strand=Plus/Minus

Query  103   GATATCACAAAAACAGCACTCCACTGGACCCCAGAAGGTAAAAGAAAGAGAGGAAGACCT  162
             || ||||||  || ||| ||||||||||| |||||||| ||||||||||| ||| | |||
Sbjct  2905  GACATCACACGAAAAGCCCTCCACTGGACACCAGAAGGCAAAAGAAAGAGGGGACGTCCT  2846

Query  163   AAAATGACATGGAGAAGAACTGTAGA  188
             ||||  || ||| |||||||||| ||
Sbjct  2845  AAAA-CACTTGGCGAAGAACTGTGGA  2821
```

>**gb|AASC02006447.1|** Aplysia californica cont2.6446, whole genome shotgun sequence
Length=50899

```
 Score = 80.6 bits (88),  Expect = 1e-11
 Identities = 101/139 (72%), Gaps = 0/139 (0%)
 Strand=Plus/Minus

Query  51     AATAAGAAGACGCTGGAGGTGGATTCGGCATGTCCTACGGAAAGGCCAGGGGGATATCAC  110
              || ||| ||| | ||||| |||||| ||||||| ||  | | ||  |  ||  ||||
Sbjct  10360  AACAAGGAGAAGATGGAGATGGATTGGGCATGTTCTGAGAAGAGAACCAGGCTATATTGT  10301

Query  111    AAAAACAGCACTCCACTGGACCCCAGAAGGTAAAAGAAAGAGAGGAAGACCTAAAATGAC  170
              ||| || || ||||||||||| || ||||   |  | ||||| || ||||| ||||||||
Sbjct  10300  AAAGACTGCCCTCCACTGGACACCCGAAGAACATCGCAAGAGGGGCAGACCCAAAATGAC  10241

Query  171    ATGGAGAAGAACTGTAGAG  189
               |||||| | || ||||||
Sbjct  10240  CTGGAGACGCACAGTAGAG  10222
```

>**gb|AASC02029508.1|** Aplysia californica cont2.29507, whole genome shotgun sequence
Length=51566

```
 Score = 80.6 bits (88),  Expect = 1e-11
 Identities = 115/162 (70%), Gaps = 0/162 (0%)
 Strand=Plus/Plus

Query  28     CAACAAGATATAGCCACAACTATAATAAGAAGACGCTGGAGGTGGATTCGGCATGTCCTA  87
              ||| | || ||  |||| |  |||| ||| ||| | ||||| |||||| ||||||| ||
Sbjct  50638  CAAGAGGACATGTCCACCATAATAACAAGGAGAAGATGGAGATGGATTGGGCATGTTCTG  50697
```

```
Query  88     CGGAAAGGCCAGGGGGATATCACAAAAACAGCACTCCACTGGACCCCAGAAGGTAAAAGA  147
               | | ||  |  |    |||   ||  || |||||||||||||| || |||||  |  |
Sbjct  50698  AGAAGAGAACCAGACTCTATTGTAAGGACTGCACTCCACTGGACACCCGAAGGACATCGC  50757

Query  148    AAGAGAGGAAGACCTAAAATGACATGGAGAAGAACTGTAGAG  189
              ||||| || ||||| |||||||| |||||| | || ||||||
Sbjct  50758  AAGAGGGGCAGACCCAAAATGACCTGGAGACGCACAGTAGAG  50799
```

>**gb|AASC02009772.1|** Aplysia californica cont2.9771, whole genome shotgun sequence
Length=18443

```
 Score = 78.8 bits (86),  Expect = 4e-11
 Identities = 64/78 (82%), Gaps = 0/78 (0%)
 Strand=Plus/Plus

Query  112    AAAACAGCACTCCACTGGACCCCAGAAGGTAAAAGAAAGAGAGGAAGACCTAAAATGACA  171
              ||||| || ||||||||||| || ||||| ||  | |||||||| ||||| ||| ||||
Sbjct  10225  AAAACTGCCCTCCACTGGACACCCGAAGGAAATCGCAAGAGAGGCAGACCCAAAGTGACC  10284

Query  172    TGGAGAAGAACTGTAGAG  189
              |||||| | |||||||||
Sbjct  10285  TGGAGACGCACTGTAGAG  10302
```

>**gb|AASC02030911.1|** Aplysia californica cont2.30910, whole genome shotgun sequence
Length=7524

```
 Score = 78.8 bits (86),  Expect = 4e-11
 Identities = 102/141 (72%), Gaps = 0/141 (0%)
 Strand=Plus/Minus

Query  49    ATAATAAGAAGACGCTGGAGGTGGATTCGGCATGTCCTACGGAAAGGCCAGGGGGATATC  108
             |||| ||| ||| | ||||| |||||| ||||||||||  | | ||  |  |    |||
Sbjct  4534  ATAACAAGGAGAAGATGGAGATGGATTGGGCATGTCCTGAGAAGAGAACCAGACTTTATT  4475

Query  109   ACAAAAACAGCACTCCACTGGACCCCAGAAGGTAAAAGAAAGAGAGGAAGACCTAAAATG  168
               ||| || |||||||||||||| || |||||  |  | ||||| || ||||| ||  ||
Sbjct  4474  GTAAAGACTGCACTCCACTGGACACCCGAAGGACATTGCAAGAGGGGCAGACCCAAGGTG  4415

Query  169   ACATGGAGAAGAACTGTAGAG  189
             || |||||| | || ||||||
Sbjct  4414  ACCTGGAGACGCACAGTAGAG  4394
```

>**gb|AASC02035229.1|** Aplysia californica cont2.35228, whole genome shotgun sequence
Length=31032

```
 Score = 78.8 bits (86),  Expect = 4e-11
 Identities = 102/141 (72%), Gaps = 0/141 (0%)
 Strand=Plus/Minus

Query  49    ATAATAAGAAGACGCTGGAGGTGGATTCGGCATGTCCTACGGAAAGGCCAGGGGGATATC  108
             |||| ||| ||| | ||||| |||||| ||||||| ||| | | ||  |  |    |||
Sbjct  8496  ATAACAAGGAGAAGATGGAGATGGATTGGGCATGTTCTAAGAAGAGAACCAGACTCTATT  8437

Query  109   ACAAAAACAGCACTCCACTGGACCCCAGAAGGTAAAAGAAAGAGAGGAAGACCTAAAATG  168
               ||| || || ||||||||||| || |||||  |  | ||||| || ||||| ||||||
Sbjct  8436  GTAAAGACTGCCCTCCACTGGACACCCGAAGGACATCGCAAGAGGGGCAGACCCAAAATG  8377

Query  169   ACATGGAGAAGAACTGTAGAG  189
             || |||||| | || || |||
Sbjct  8376  ACCTGGAGACGCACAGTGGAG  8356
```

>**gb|AASC02063867.1|** Aplysia californica cont2.63866, whole genome shotgun sequence
Length=5896

```
 Score = 78.8 bits (86),  Expect = 4e-11
 Identities = 102/141 (72%), Gaps = 0/141 (0%)
 Strand=Plus/Minus

Query  49    ATAATAAGAAGACGCTGGAGGTGGATTCGGCATGTCCTACGGAAAGGCCAGGGGGATATC  108
             |||| ||| ||| | ||||| |||||| | ||||| ||| ||| ||  |  |    |||
Sbjct  3949  ATAACAAGGAGAAGATGGAGATGGATTGGTCATGTTCTAAGGAGAGAACCAGATTCTATT  3890

Query  109   ACAAAAACAGCACTCCACTGGACCCCAGAAGGTAAAAGAAAGAGAGGAAGACCTAAAATG  168
               ||| || || ||||||||||| || |||||  |  | ||||| || ||||| ||| ||
Sbjct  3889  GTAAAGACTGCCCTCCACTGGACACCCGAAGGACATCGCAAGAGGGGCAGACCCAAAGTG  3830

Query  169   ACATGGAGAAGAACTGTAGAG  189
             || |||||| | || ||||||
Sbjct  3829  ACCTGGAGACGCACAGTAGAG  3809
```

>**gb|AAGJ02030884.1|** Strongylocentrotus purpuratus Ctg31050, whole genome shotgun sequence
Length=72401

```
 Score = 78.8 bits (86),  Expect = 4e-11
 Identities = 69/86 (80%), Gaps = 0/86 (0%)
 Strand=Plus/Plus

Query  103    GATATCACAAAAACAGCACTCCACTGGACCCCAGAAGGTAAAAGAAAGAGAGGAAGACCT  162
              || ||||||  |||||| ||||||||||| |||||||| || | |||||| ||| | |||
Sbjct  53304  GACATCACATGAACAGCCCTCCACTGGACACCAGAAGGCAACAAAAAGAGGGGACGTCCT  53363
```

```
Query  163    AAAATGACATGGAGAAGAACTGTAGA  188
              ||||  || ||| | |||||||| ||
Sbjct  53364  AAAAACACTTGGTGTAGAACTGTGGA  53389
```

>gb|AAGJ02112094.1| Strongylocentrotus purpuratus Ctg112370, whole genome shotgun sequence
Length=59792

```
 Score = 78.8 bits (86),  Expect = 4e-11
 Identities = 69/86 (80%), Gaps = 0/86 (0%)
 Strand=Plus/Minus

Query  103    GATATCACAAAAACAGCACTCCACTGGACCCCAGAAGGTAAAAGAAAGAGAGGAAGACCT  162
              || ||||||  |||||| ||||||||||| |||||||| || |||||||| | | |  ||
Sbjct  18986  GAAATCACACGAACAGCCCTCCACTGGACACCAGAAGGCAACAGAAAGAGGGAACGTTCT  18927

Query  163    AAAATGACATGGAGAAGAACTGTAGA  188
              ||||  || ||| |||||||||| ||
Sbjct  18926  AAAAACACTTGGCGAAGAACTGTGGA  18901
```

>gb|AAGJ02140935.1| Strongylocentrotus purpuratus Ctg141235, whole genome shotgun sequence
Length=11188

```
 Score = 78.8 bits (86),  Expect = 4e-11
 Identities = 102/141 (72%), Gaps = 0/141 (0%)
 Strand=Plus/Minus

Query  48    TATAATAAGAAGACGCTGGAGGTGGATTCGGCATGTCCTACGGAAAGGCCAGGGGGATAT  107
             ||| || |||||| | ||||| |||||  | |||||  |  | |||  |   |  |  ||
Sbjct  3752  TATCATGAGAAGAAGGTGGAGATGGATCGGACATGTAATGAGAAAAAACATAGATGGCAT  3693

Query  108   CACAAAAACAGCACTCCACTGGACCCCAGAAGGTAAAAGAAAGAGAGGAAGACCTAAAAT  167
             || |  || ||| ||||||||||| |||||||| ||||||||||| ||| | |||||||
Sbjct  3692  CATACGAATAGCCCTCCACTGGACACCAGAAGGCAAAAGAAAGAGGGGACGTCCTAAAAA  3633

Query  168   GACATGGAGAAGAACTGTAGA  188
              || |||   |||||||| ||
Sbjct  3632  CACTTGGCAGAGAACTGTGGA  3612
```

>gb|AASC02024690.1| Aplysia californica cont2.24689, whole genome shotgun sequence
Length=1444

```
 Score = 77.0 bits (84),  Expect = 2e-10
 Identities = 102/142 (71%), Gaps = 0/142 (0%)
 Strand=Plus/Plus

Query  48   TATAATAAGAAGACGCTGGAGGTGGATTCGGCATGTCCTACGGAAAGGCCAGGGGGATAT  107
            ||||| ||| ||| | ||||| |||||| ||||||| ||  | | ||  |  |    |||
Sbjct  164  TATAACAAGGAGAGGATGGAGATGGATTGGGCATGTTCTGAGAAGAGAACCAGACTCTAT  223

Query  108  CACAAAAACAGCACTCCACTGGACCCCAGAAGGTAAAAGAAAGAGAGGAAGACCTAAAAT  167
               ||| || || ||||||||||| || |||||  |  | ||||| || ||||| ||| |
Sbjct  224  TGTAAAGACTGCCCTCCACTGGACACCCGAAGGACATCGCAAGAGGGGCAGACCCAAAGT  283

Query  168  GACATGGAGAAGAACTGTAGAG  189
            ||| |||||| | || ||||||
Sbjct  284  GACCTGGAGACGCACAGTAGAG  305
```

>gb|AASC02032210.1| Aplysia californica cont2.32209, whole genome shotgun sequence
Length=43152

```
 Score = 77.0 bits (84),  Expect = 2e-10
 Identities = 114/162 (70%), Gaps = 0/162 (0%)
 Strand=Plus/Plus

Query  28     CAACAAGATATAGCCACAACTATAATAAGAAGACGCTGGAGGTGGATTCGGCATGTCCTA  87
              ||| | || ||  |||| |  |||| ||| ||| | || || |||||| ||  ||| |||
Sbjct  37302  CAAGAGGACATGACCACCATAATAACAAGGAGAAGATGAAGATGGATTGGGAGTGTTCTA  37361

Query  88     CGGAAAGGCCAGGGGGATATCACAAAAACAGCACTCCACTGGACCCCAGAAGGTAAAAGA  147
               | | ||  |  |    |||   ||| ||||| ||||||||||| || |||||  |  |
Sbjct  37362  AGAAGAGAACCAGACTTTATTGTAAAGACAGCCCTCCACTGGACACCCGAAGGACATTGC  37421

Query  148    AAGAGAGGAAGACCTAAAATGACATGGAGAAGAACTGTAGAG  189
              ||||| || ||||| ||| ||||||||||| | || ||||||
Sbjct  37422  AAGAGGGGCAGACCCAAAGTGACATGGAGACGCACAGTAGAG  37463
```

>gb|AASC02040983.1| Aplysia californica cont2.40982, whole genome shotgun sequence
Length=25081

```
 Score = 77.0 bits (84),  Expect = 2e-10
 Identities = 114/162 (70%), Gaps = 0/162 (0%)
 Strand=Plus/Plus

Query  28    CAACAAGATATAGCCACAACTATAATAAGAAGACGCTGGAGGTGGATTCGGCATGTCCTA  87
             ||| | || ||  |||| |  | || ||| ||| | ||||| ||||||||| |||| ||
Sbjct  6342  CAAGAGGACATGACCACCAAAACAACAAGGAGAAGGTGGAGATGGATTCGGTATGTTCTG  6401

Query  88    CGGAAAGGCCAGGGGGATATCACAAAAACAGCACTCCACTGGACCCCAGAAGGTAAAAGA  147
              | ||||  |  |    |||   ||| || || ||||||||||| || |||||  |  |
Sbjct  6402  AGAAAAGAACCAGACTCTATTGTAAAGACTGCTCTCCACTGGACACCCGAAGGACACCGC  6461
```

```
Query  148   AAGAGAGGAAGACCTAAAATGACATGGAGAAGAACTGTAGAG  189
             ||||| || ||||| ||| |||| |||||| | || ||||||
Sbjct  6462  AAGAGGGGCAGACCCAAAGTGACCTGGAGACGCACAGTAGAG  6503
```

>**dbj|BAAF04076102.1|** Oryzias latipes DNA, contig76102 in scaffold235, strain: Hd-rR, whole genome shotgun sequence
Length=10135

```
 Score = 77.0 bits (84),  Expect = 2e-10
 Identities = 119/169 (70%), Gaps = 1/169 (0%)
 Strand=Plus/Plus

Query  28    CAACAAGATATAGCCACAACTATAATAAGAAGACGCTGGAGGTGGATTCGGCA-TGTCCT  86
             ||| |||| ||  | | || |||  ||| |||||||||||  |||||| | || ||| ||
Sbjct  4679  CAAGAAGACATGTCAATAATTATCCTAAAAAGACGCTGGACATGGATTGGACAATGTACT  4738

Query  87    ACGGAAAGGCCAGGGGGATATCACAAAAACAGCACTCCACTGGACCCCAGAAGGTAAAAG  146
               |||  |   |       |||| ||| |||||||| |||||||| ||||| || |||||
Sbjct  4739  CAGGAGTGAAGAATACACAATCATAAAGACAGCACTACACTGGACACCAGATGGAAAAAG  4798

Query  147   AAAGAGAGGAAGACCTAAAATGACATGGAGAAGAACTGTAGAGGCAGAG  195
             ||| |||||| |||| || || || | | |  |||| | ||| || |||
Sbjct  4799  AAAAAGAGGATGACCCAAGATCACCTTGCGCCGAACAGCAGAAGCCGAG  4847
```

>**gb|AAGJ02121827.1|** Strongylocentrotus purpuratus Ctg122110, whole genome shotgun sequence
Length=13320

```
 Score = 77.0 bits (84),  Expect = 2e-10
 Identities = 100/135 (74%), Gaps = 4/135 (2%)
 Strand=Plus/Plus

Query  56    GAAGACGCTGGAGGTGGATTCGGCATGTCCTACG-GAAAGGCCAGGGG-GATATCACAAA  113
             ||||| | ||||| |||||  | |||||  || | |||||  ||  |  || ||||||
Sbjct  6958  GAAGAAGGTGGAGATGGATCGGACATGT--TATGAGAAAGAACATAGATGACATCACACG  7015

Query  114   AACAGCACTCCACTGGACCCCAGAAGGTAAAAGAAAGAGAGGAAGACCTAAAATGACATG  173
             |||||| ||||||||||| ||||| || |||||||||||  || | |||||||  |  ||
Sbjct  7016  AACAGCCCTCCACTGGACACCAGACGGCAAAAGAAAGAGGAGACGTCCTAAAAACAATTG  7075

Query  174   GAGAAGAACTGTAGA  188
             | | | |||||| ||
Sbjct  7076  GTGGATAACTGTGGA  7090
```

>**gb|AAGJ02190055.1|** Strongylocentrotus purpuratus Ctg190360, whole genome shotgun sequence
Length=937

```
 Score = 77.0 bits (84),  Expect = 2e-10
 Identities = 62/75 (82%), Gaps = 0/75 (0%)
 Strand=Plus/Minus

Query  114  AACAGCACTCCACTGGACCCCAGAAGGTAAAAGAAAGAGAGGAAGACCTAAAATGACATG  173
            |||||| ||||||||||| |||||||| ||| ||||||| ||| |||||||||  ||  |
Sbjct  895  AACAGCCCTCCACTGGACACCAGAAGGCAAACGAAAGAGGGGATGACCTAAAAACACTCG  836

Query  174  GAGAAGAACTGTAGA  188
            |  ||||||||| ||
Sbjct  835  GCCAAGAACTGTGGA  821
```

>**gb|AASC02023390.1|** Aplysia californica cont2.23389, whole genome shotgun sequence
Length=15090

```
 Score = 75.2 bits (82),  Expect = 5e-10
 Identities = 101/141 (71%), Gaps = 0/141 (0%)
 Strand=Plus/Minus

Query  49    ATAATAAGAAGACGCTGGAGGTGGATTCGGCATGTCCTACGGAAAGGCCAGGGGGATATC  108
             |||| ||| ||||| ||||| |||||| ||||||| ||  | | ||  |  |    |||
Sbjct  5761  ATAACAAGGAGACGATGGAGATGGATTGGGCATGTTCTGAGAAGAGAACCAGACTCTATA  5702

Query  109   ACAAAAACAGCACTCCACTGGACCCCAGAAGGTAAAAGAAAGAGAGGAAGACCTAAAATG  168
               ||| || ||||||||||||||  | |||||  |  | ||||| || ||||||  || ||
Sbjct  5701  GTAAAGACTGCACTCCACTGGACATCCGAAGGACATCGTAAGAGGGGCAGACCCAAGGTG  5642

Query  169   ACATGGAGAAGAACTGTAGAG  189
             || |||||| | || ||||||
Sbjct  5641  ACCTGGAGACGCACAGTAGAG  5621
```

>**dbj|BAAE01046442.1|** Oryzias latipes DNA, contig46371 in scaffold1431, strain: HNI, whole genome shotgun sequence
Length=2757

```
 Score = 75.2 bits (82),  Expect = 5e-10
 Identities = 120/171 (70%), Gaps = 6/171 (3%)
 Strand=Plus/Minus

Query  28   CAACAAGATATAGCCACAACTATAATAAGAAGACGCTGGAGGTGGATTCGGCATGTCCTA  87
            ||| |||||||  | |||| |||  | | |||| ||||||  |||||| | ||||| |
Sbjct  539  CAAGAAGATATGTCAACAATTATCCTCAAAAGATGCTGGACATGGATTGGACATGTACTC  480

Query  88   CGGA---AAGGCCAGGGGGATATCACAAAAACAGCACTCCACTGGACCCCAGAAGGTAAA  144
```

```
           |||   ||| | |       |||| ||| |||||||| ||||||||  |||| || |||
Sbjct  479  AGGAGTGAAGACAACACA---ATCATAAAGACAGCACTACACTGGACATCAGATGGAAAA  423

Query  145  AGAAAGAGAGGAAGACCTAAAATGACATGGAGAAGAACTGTAGAGGCAGAG  195
            ||||| |||||| | ||   |  | || ||| |   |||| ||||| || |||
Sbjct  422  AGAAAAAGAGGACGGCCGCAGCTCACCTGGTGCCGAACAGTAGAAGCCGAG  372
```

>**dbj|BAAE01011739.1|** Oryzias latipes DNA, contig12122 in scaffold253, strain: HNI, whole genome shotgun sequence
Length=1542

```
 Score = 75.2 bits (82),  Expect = 5e-10
 Identities = 104/146 (71%), Gaps = 11/146 (7%)
 Strand=Plus/Minus

Query  28   CAACAAGATATAGCCACAACTATAATAAGAAGACGCTGGAGGTGGATTCGGCATGTCCTA  87
            ||| |||| ||   | |||| |||   | | |||||||||||  |||||| | ||||| ||
Sbjct  342  CAAGAAGACATGTCAACAATTATCCTCAAAAGACGCTGGACATGGATTGGACATGTACTC  283

Query  88   CGGAAAGGCCAGGGGGATATCACAAAAACAGCACTCCACTGGACCCCAGAAGGTAAAAGA  147
             |||         |||||  ||   || |||||||  |||||||| ||||| || ||||||
Sbjct  282  AGGA---------GGGAT--CATGAAGACAGCACCACACTGGACACCAGATGGAAAAAGA  234

Query  148  AAGAGAGGAAGACCTAAAATGACATG  173
             | |||||||| || || || |||||
Sbjct  233  CAAAGAGGAAGGCCGAAGATCACATG  208
```

>**gb|AAGJ02012985.1|** Strongylocentrotus purpuratus Ctg13077, whole genome shotgun sequence
Length=16852

```
 Score = 75.2 bits (82),  Expect = 5e-10
 Identities = 55/64 (85%), Gaps = 0/64 (0%)
 Strand=Plus/Minus

Query  103  GATATCACAAAAACAGCACTCCACTGGACCCCAGAAGGTAAAAGAAAGAGAGGAAGACCT  162
            || ||||||  |||||| ||||||||||| |||||||| |||||||||| ||| | |||
Sbjct  513  GACATCACACGAACAGCCCTCCACTGGACACCAGAAGGCAAAAGAAAGAGGGGACGTCCT  454

Query  163  AAAA  166
            ||||
Sbjct  453  AAAA  450
```

>**gb|AAGJ02032830.1|** Strongylocentrotus purpuratus Ctg33001, whole genome shotgun sequence
Length=33717

```
 Score = 75.2 bits (82),  Expect = 5e-10
 Identities = 70/88 (79%), Gaps = 2/88 (2%)
 Strand=Plus/Minus

Query  103    GATATCACAAAAACAGCACTCCACTGGACCCCAGAAGG--TAAAAGAAAGAGAGGAAGAC  160
              || ||||||  |||||| ||||||||||| ||||||||    |||| |||||| ||| | |
Sbjct  32830  GACATCACACGAACAGCCCTCCACTGGACACCAGAAGGCAAAAAAAAAAGAGGGGACGTC  32771

Query  161    CTAAAATGACATGGAGAAGAACTGTAGA  188
              ||||||  || ||| | |||||||| ||
Sbjct  32770  CTAAAAACACTTGGTGTAGAACTGTGGA  32743
```

>**gb|AAGJ02122723.1|** Strongylocentrotus purpuratus Ctg123007, whole genome shotgun sequence
Length=7881

```
 Score = 75.2 bits (82),  Expect = 5e-10
 Identities = 61/74 (82%), Gaps = 0/74 (0%)
 Strand=Plus/Minus

Query  115   ACAGCACTCCACTGGACCCCAGAAGGTAAAAGAAAGAGAGGAAGACCTAAAATGACATGG  174
             ||||| ||||||||||| |||||||| |||| |||||| ||| | ||||||    || |||
Sbjct  4110  ACAGCCCTCCACTGGACACCAGAAGGCAAAATAAAGAGGGGACGTCCTAAAGACACTTGG  4051

Query  175   AGAAGAACTGTAGA  188
              |||||||||| ||
Sbjct  4050  CGAAGAACTGTGGA  4037
```

>**gb|AASC02052968.1|** Aplysia californica cont2.52967, whole genome shotgun sequence
Length=24837

Sort alignments for this subject sequence by:
E value **Score** **Percent identity**
Query start position **Subject start position**

```
 Score = 73.4 bits (80),  Expect = 2e-09
 Identities = 112/160 (70%), Gaps = 0/160 (0%)
 Strand=Plus/Plus

Query  28     CAACAAGATATAGCCACAACTATAATAAGAAGACGCTGGAGGTGGATTCGGCATGTCCTA  87
              ||| | || ||  |||| |  |||| ||| ||| | ||||| |||||| ||||||| ||
Sbjct  18508  CAAGAGGACATGACCACCATAATAACAAGGAGAAGATGGAGATGGATTGGGCATGTTCTG  18567

Query  88     CGGAAAGGCCAGGGGGATATCACAAAAACAGCACTCCACTGGACCCCAGAAGGTAAAAGA  147
               | | ||  |  |     |||    ||| |  || ||||||||||| || |||||   |  |
Sbjct  18568  AGAAGAGAACCAGACTTTATTGTAAAGATTGCCCTCCACTGGACACCCGAAGGACATTGC  18627
```

```
Query  148    AAGAGAGGAAGACCTAAAATGACATGGAGAAGAACTGTAG  187
               ||||||||||||| ||| |||| |||||| | || ||||
Sbjct  18628  TAGAGAGGAAGACCCAAAGTGACCTGGAGACGCACAGTAG  18667


 Score = 57.2 bits (62),  Expect = 1e-04
 Identities = 105/151 (69%), Gaps = 5/151 (3%)
 Strand=Plus/Plus

Query  43    ACAACTATAATAAGAA---GACGCTGGAGGTGGATTCGGCATGTCCTACGGA-AAGGCCA  98
             ||||| ||||||| ||   || | ||||| |||||| | ||||||||  | | || ||||
Sbjct  6099  ACAACCATAATAAAAAAGAGAAGGTGGAGATGGATTGGACATGTCCTGAGCAGAAAGCCA  6158

Query  99    GGGGGATATCACAAAAACAGCACTCCACTGGACCCCAGAAGGTAAAAGAAAGAGAGGAAG  158
             |     |||    || || || ||||||||||| || |||||     | ||  | || ||
Sbjct  6159  GACTC-TATTGTGAAGACTGCCCTCCACTGGACACCCGAAGGACGTCGCAAAGGGGGCAG  6217

Query  159   ACCTAAAATGACATGGAGAAGAACTGTAGAG  189
             ||| ||| |||  |||||| | || ||||||
Sbjct  6218  ACCCAAAGTGAACTGGAGACGCACAGTAGAG  6248
```

>**gb|AASC02009483.1|** Aplysia californica cont2.9482, whole genome shotgun sequence
Length=65660

```
 Score = 71.6 bits (78),  Expect = 7e-09
 Identities = 105/149 (70%), Gaps = 0/149 (0%)
 Strand=Plus/Minus

Query  41     CCACAACTATAATAAGAAGACGCTGGAGGTGGATTCGGCATGTCCTACGGAAAGGCCAGG  100
              |||| |  |||| ||| ||| | |||||  ||||| ||||||| ||  | | ||  |  |
Sbjct  40689  CCACCATAATAAAAAGGAGAAGATGGAGACGGATTGGGCATGTTCTGAGAAGAGAACCAG  40630

Query  101    GGGATATCACAAAAACAGCACTCCACTGGACCCCAGAAGGTAAAAGAAAGAGAGGAAGAC  160
                  ||     ||||| || ||||||||||| || |||||  |  | ||||| |||||||
Sbjct  40629  ACTCTACTGTGAAAACTGCCCTCCACTGGACACCTGAAGGACATTGCAAGAGGGGAAGAC  40570

Query  161    CTAAAATGACATGGAGAAGAACTGTAGAG  189
              | ||| |||| |||||| | || ||||||
Sbjct  40569  CCAAAGTGACCTGGAGACGTACAGTAGAG  40541
```

>**gb|AASC02025249.1|** Aplysia californica cont2.25248, whole genome shotgun sequence
Length=34021

```
 Score = 71.6 bits (78),  Expect = 7e-09
 Identities = 113/162 (69%), Gaps = 0/162 (0%)
 Strand=Plus/Minus

Query  28     CAACAAGATATAGCCACAACTATAATAAGAAGACGCTGGAGGTGGATTCGGCATGTCCTA  87
              ||| | || ||  ||||||  |||| ||| ||| |  |||| |||||| ||||||| ||
Sbjct  13182  CAAGAGGACATGACCACAATAATAACAAGGAGAAGAGGGAGATGGATTGGGCATGTTCTG  13123

Query  88     CGGAAAGGCCAGGGGGATATCACAAAAACAGCACTCCACTGGACCCCAGAAGGTAAAAGA  147
               | | ||     |    |||   ||| || || |||| |||||| || |||||  |  |
Sbjct  13122  AGAAGAGAATCAGACTCTATTGTAAAGACTGCCCTCCCCTGGACACCTGAAGGACATCGG  13063

Query  148    AAGAGAGGAAGACCTAAAATGACATGGAGAAGAACTGTAGAG  189
              ||||| || ||||| ||| |||| |||||| |||| ||||||
Sbjct  13062  AAGAGGGGCAGACCCAAAGTGACCTGGAGACGAACAGTAGAG  13021
```

>**gb|AASC02029272.1|** Aplysia californica cont2.29271, whole genome shotgun sequence
Length=2285

```
 Score = 71.6 bits (78),  Expect = 7e-09
 Identities = 113/162 (69%), Gaps = 0/162 (0%)
 Strand=Plus/Plus

Query  28    CAACAAGATATAGCCACAACTATAATAAGAAGACGCTGGAGGTGGATTCGGCATGTCCTA  87
             ||| | || ||  |||| || |||| ||| ||| | ||||| |||||| ||||||| |||
Sbjct  1101  CAAGAGGACATGACCACCACAATAAGAAGGAGAAGATGGAGATGGATTGGGCATGTTCTA  1160

Query  88    CGGAAAGGCCAGGGGGATATCACAAAAACAGCACTCCACTGGACCCCAGAAGGTAAAAGA  147
              | | ||  |   |   |||   ||| || || ||||||||||| || |||||  |  |
Sbjct  1161  AGAAGAGAACCAAGCTCTATTGTAAAGACTGCCCTCCACTGGACACCCGAAGGACATTGC  1220

Query  148   AAGAGAGGAAGACCTAAAATGACATGGAGAAGAACTGTAGAG  189
             ||||| || ||||| | | |||| | |||| | |  ||||||
Sbjct  1221  AAGAGGGGCAGACCCAGAGTGACCTAGAGACGCATAGTAGAG  1262
```

>**gb|AASC02029386.1|** Aplysia californica cont2.29385, whole genome shotgun sequence
Length=51440

```
 Score = 71.6 bits (78),  Expect = 7e-09
 Identities = 109/154 (70%), Gaps = 1/154 (0%)
 Strand=Plus/Plus

Query  36     TATAGCCACAACTATAATAAGAAGACGCTGGAGGTGGATTCGGCATGTCCTACGGAAAGG  95
              |||| |||| |  || | ||| ||| | ||||| |||||| ||||||| ||  | | ||
Sbjct  48508  TATA-CCACCATAATTAAAAGGAGAAGATGGAGATGGATTGGGCATGTTCTGAGAAGAGA  48566

Query  96     CCAGGGGGATATCACAAAAACAGCACTCCACTGGACCCCAGAAGGTAAAAGAAAGAGAGG  155
               |  |    |||    || || || ||||||||||| || ||||| ||  | ||||| ||
Sbjct  48567  ACCAGACTCTATTGGGAAGACTGCCCTCCACTGGACACCCGAAGGAAATCGCAAGAGGGG  48626
```

```
Query  156    AAGACCTAAAATGACATGGAGAAGAACTGTAGAG  189
               ||||| ||| |||| |||||| | || ||||||
Sbjct  48627  CAGACCCAAAGTGACCTGGAGACGCACAGTAGAG  48660
```

>**gb|AASC02033525.1|** Aplysia californica cont2.33524, whole genome shotgun sequence
Length=13560

```
 Score = 71.6 bits (78),  Expect = 7e-09
 Identities = 109/153 (71%), Gaps = 8/153 (5%)
 Strand=Plus/Minus

Query  41     CCACAACTATAATAAGAAGACGCTGGAGGTGGATTCGGCATGTCCTACGGAAAGGCCAGG  100
              |||| || |||| ||| ||| | ||||| |||||| ||||||| ||  | | ||  |
Sbjct  9112   CCACCACAATAACAAGGAGAAGATGGAGATGGATTGGGCATGTTCTGAGAACAGAAC---  9056

Query  101    GGGATATCAC----AAAAACAGCACTCCACTGGACCCCAGAAGGTAAAAGAAAGAGAGGA  156
              | ||  ||||    ||| || || ||||||||||| || |||||  |  | ||||| ||
Sbjct  9055   GAGAC-TCACTTGTAAAGACTGCCCTCCACTGGACACCCGAAGGACATCGCAAGAGGGGC  8997

Query  157    AGACCTAAAATGACATGGAGAAGAACTGTAGAG  189
              ||||| ||| ||||  ||||| | || ||||||
Sbjct  8996   AGACCCAAAGTGACCCGGAGACGCACAGTAGAG  8964
```

>**gb|AASC02040771.1|** Aplysia californica cont2.40770, whole genome shotgun sequence
Length=49979

```
 Score = 71.6 bits (78),  Expect = 7e-09
 Identities = 113/162 (69%), Gaps = 0/162 (0%)
 Strand=Plus/Minus

Query  28     CAACAAGATATAGCCACAACTATAATAAGAAGACGCTGGAGGTGGATTCGGCATGTCCTA  87
              ||| | || ||  |||| |  |||| | | ||| | ||||| |||||| ||||||| ||
Sbjct  46736  CAAGAGGACATGACCACCATAATAACAGGGAGAAGATGGAGATGGATTGGGCATGTTCTG  46677

Query  88     CGGAAAGGCCAGGGGGATATCACAAAAACAGCACTCCACTGGACCCCAGAAGGTAAAAGA  147
               | | ||  || |    |||   ||| || ||||||| ||||||||| || ||  |  |
Sbjct  46676  AGAAGAGAACAAGATTCTATAGTAAAGACTGCACTCCTCTGGACCCCCGATGGACATCGC  46617

Query  148    AAGAGAGGAAGACCTAAAATGACATGGAGAAGAACTGTAGAG  189
              ||||| || ||||| ||  |||| |||||| | || ||||||
Sbjct  46616  AAGAGGGGCAGACCCAAGGTGACCTGGAGACGCACAGTAGAG  46575
```

>**gb|AASC02041111.1|** Aplysia californica cont2.41110, whole genome shotgun sequence
Length=51507

```
 Score = 71.6 bits (78),  Expect = 7e-09
 Identities = 113/162 (69%), Gaps = 0/162 (0%)
 Strand=Plus/Minus

Query  28     CAACAAGATATAGCCACAACTATAATAAGAAGACGCTGGAGGTGGATTCGGCATGTCCTA  87
              ||| | || ||  |||| |  |||| ||| ||| | |||||  ||||| ||||||| ||
Sbjct  15326  CAAGAGGACATGACCACCATAATAACAAGGAGAAGATGGAGACGGATTGGGCATGTTCTG  15267

Query  88     CGGAAAGGCCAGGGGGATATCACAAAAACAGCACTCCACTGGACCCCAGAAGGTAAAAGA  147
               | | ||  |  |    |||   ||| || || ||||||||||| || |||||  |  |
Sbjct  15266  AGAAGAGAACCAGACTCTATTGTAAAGACTGCCCTCCACTGGACACCCGAAGGACATCGC  15207

Query  148    AAGAGAGGAAGACCTAAAATGACATGGAGAAGAACTGTAGAG  189
              ||||| || ||||| |||||||| ||||||   || ||||||
Sbjct  15206  AAGAGGGGCAGACCCAAAATGACCTGGAGACACACAGTAGAG  15165
```

>**gb|AASC02045802.1|** Aplysia californica cont2.45801, whole genome shotgun sequence
Length=59272

```
 Score = 71.6 bits (78),  Expect = 7e-09
 Identities = 113/162 (69%), Gaps = 0/162 (0%)
 Strand=Plus/Minus

Query  28     CAACAAGATATAGCCACAACTATAATAAGAAGACGCTGGAGGTGGATTCGGCATGTCCTA  87
              ||| | || ||  |||| |  |||| ||| ||| | ||||| |||||  ||||||| |||
Sbjct  42118  CAAGAGGACATGACCACCATAATAACAAGGAGAAGATGGAGATGGATCAGGCATGTTCTA  42059

Query  88     CGGAAAGGCCAGGGGGATATCACAAAAACAGCACTCCACTGGACCCCAGAAGGTAAAAGA  147
               | | ||  |  |    |||   ||| || || ||||||||||| || |||||     |
Sbjct  42058  AGAAGAGAACCAGACTCTATTGTAAAGACTGCCCTCCACTGGACACCCGAAGGACGTCGC  41999

Query  148    AAGAGAGGAAGACCTAAAATGACATGGAGAAGAACTGTAGAG  189
              ||||| || ||||| |||||||| ||||||   || ||||||
Sbjct  41998  AAGAGGGGCAGACCCAAAATGACCTGGAGACACACAGTAGAG  41957
```

>**gb|AASC02049491.1|** Aplysia californica cont2.49490, whole genome shotgun sequence
Length=9605

```
 Score = 71.6 bits (78),  Expect = 7e-09
 Identities = 115/163 (70%), Gaps = 2/163 (1%)
 Strand=Plus/Minus

Query  28     CAACAAGATATAGCCACAACTATAATAAGAAGACGCTGGAGGTGGATTCGGCATGTCCTA  87
              ||| | || ||  |||| || |||| ||| ||| | ||||| |||||| | ||||| ||
Sbjct  7108   CAAGAGGACATGACCACCACAATAAAAAGGAGAAGATGGAGATGGATTGGACATGTTCTG  7049

Query  88     CGGAAAG-GCCAGGGGGATATCACAAAAACAGCACTCCACTGGACCCCAGAAGGTAAAAG  146
```

```
             | | || |||||      |||     || || || ||||||||||| || ||||    |  |
Sbjct  7048  AGAAGAGAGCCAGACTC-TATTGTGAAGACTGCCCTCCACTGGACACCCGAAGTACATCG  6990

Query  147   AAAGAGAGGAAGACCTAAAATGACATGGAGAAGAACTGTAGAG  189
              ||||| || ||||| ||| |||| |||||| | || ||||||
Sbjct  6989  CAAGAGGGGCAGACCCAAAGTGACCTGGAGACGCACAGTAGAG  6947
```

>**gb|AASC02061593.1|** Aplysia californica cont2.61592, whole genome shotgun sequence
Length=8763

```
 Score = 71.6 bits (78),  Expect = 7e-09
 Identities = 113/162 (69%), Gaps = 0/162 (0%)
 Strand=Plus/Minus

Query  28    CAACAAGATATAGCCACAACTATAATAAGAAGACGCTGGAGGTGGATTCGGCATGTCCTA  87
             ||| | || ||  |||| |  |||  ||| ||| | ||||||||||||  | ||||| ||
Sbjct  4639  CAAAAGGACATGACCACCATAATAGAAAGGAGAAGATGGAGGTGGATTAGACATGTTCTG  4580

Query  88    CGGAAAGGCCAGGGGGATATCACAAAAACAGCACTCCACTGGACCCCAGAAGGTAAAAGA  147
              | ||||  |  |     |||    ||| || || ||||||||||| || |||||  |  |
Sbjct  4579  AGAAAAGAACCAGACTCTATTTTAAAGACTGCCCTCCACTGGACACCCGAAGGACATCGC  4520

Query  148   AAGAGAGGAAGACCTAAAATGACATGGAGAAGAACTGTAGAG  189
              |||| || ||||| ||| |||| |||||| | || ||||||
Sbjct  4519  CAGAGGGGCAGACCCAAAGTGACCTGGAGACGCACAGTAGAG  4478
```

>**dbj|BAAF04071142.1|** Oryzias latipes DNA, contig71142 in scaffold204, strain: Hd-rR, whole genome shotgun sequence
Length=22055

```
 Score = 71.6 bits (78),  Expect = 7e-09
 Identities = 119/171 (69%), Gaps = 6/171 (3%)
 Strand=Plus/Minus

Query  28    CAACAAGATATAGCCACAACTATAATAAGAAGACGCTGGAGGTGGATTCGGCATGTCCTA  87
             ||| |||| ||  | |||| |||  | | |||||||||||  |||||| | ||||| ||
Sbjct  6308  CAAGAAGACATGTCAACAATTATCTTCAAAAGACGCTGGATATGGATTGGACATGTACTC  6249

Query  88    CGGAAAGGCCAGGGGGATA---TCACAAAAACAGCACTCCACTGGACCCCAGAAGGTAAA  144
               ||   | || |    | |    ||| ||| |||||||| |||||| | || || || |||
Sbjct  6248  AAGA---GTCAAGAAAACACGATCATAAAGACAGCACTACACTGGGCACCGGATGGAAAA  6192

Query  145   AGAAAGAGAGGAAGACCTAAAATGACATGGAGAAGAACTGTAGAGGCAGAG  195
             ||||| ||||||    || || || || |||    ||||| ||||| || |||
Sbjct  6191  AGAAAAAGAGGACAGCCGAAGATCACCTGGCACAGAACAGTAGAAGCTGAG  6141
```

>**dbj|BAAE01034390.1|** Oryzias latipes DNA, contig34339 in scaffold972, strain: HNI, whole genome shotgun sequence
Length=1137

```
 Score = 71.6 bits (78),  Expect = 7e-09
 Identities = 119/170 (70%), Gaps = 4/170 (2%)
 Strand=Plus/Plus

Query  28   CAACAAGATATAGCCACAACTATAATAAGAAGACGCTGGAGGTGGATTCGGCATGTCCTA  87
            ||| |||| ||  |  ||| |||   | | |||||||||||  |||||| | ||||| ||
Sbjct  208  CAAGAAGACATGTCATCAATTATCCTCAAAAGACGCTGGACATGGATTGGACATGTACTC  267

Query  88   CGGAAAG--GCCAGGGGGATATCACAAAAACAGCACTCCACTGGACCCCAGAAGGTAAAA  145
             |||  |  | ||     |||  | ||| |||||| | || ||||| ||||| || ||||
Sbjct  268  AGGAGTGAAGACAACACAATA--ATAAAGACAGCATTACATTGGACACCAGATGGAAAAA  325

Query  146  GAAAGAGAGGAAGACCTAAAATGACATGGAGAAGAACTGTAGAGGCAGAG  195
            |||| |||||| | || || || || ||| |  |||| ||||| || |||
Sbjct  326  GAAAAAGAGGACGGCCGAAGATCACCTGGCGCCGAACAGTAGAAGCCGAG  375
```

>**gb|ACQM01047541.1|** Saccoglossus kowalevskii Contig47657, whole genome shotgun sequence
Length=11608

```
 Score = 69.8 bits (76),  Expect = 2e-08
 Identities = 58/71 (81%), Gaps = 0/71 (0%)
 Strand=Plus/Plus

Query  112   AAAACAGCACTCCACTGGACCCCAGAAGGTAAAAGAAAGAGAGGAAGACCTAAAATGACA  171
             ||||||||| | | |||||| ||| | ||| || |||| ||||| ||||| ||||  |||
Sbjct  1305  AAAACAGCAATTCGCTGGACACCACATGGTCAACGAAACAGAGGGAGACCAAAAACCACA  1364

Query  172   TGGAGAAGAAC  182
             |||||||||||
Sbjct  1365  TGGAGAAGAAC  1375
```

>**gb|AASC02028998.1|** Aplysia californica cont2.28997, whole genome shotgun sequence
Length=3048

```
 Score = 69.8 bits (76),  Expect = 2e-08
 Identities = 91/126 (72%), Gaps = 0/126 (0%)
 Strand=Plus/Minus

Query  64    TGGAGGTGGATTCGGCATGTCCTACGGAAAGGCCAGGGGGATATCACAAAAACAGCACTC  123
             ||||| |||||| ||||||| ||  | | ||  |  |     || |  ||| || || |||
Sbjct  1404  TGGAGATGGATTGGGCATGTTCTGAGAAGAGAACCAGACTCTAGCGTAAAGACTGCCCTC  1345

Query  124   CACTGGACCCCAGAAGGTAAAAGAAAGAGAGGAAGACCTAAAATGACATGGAGAAGAACT  183
```

```
             |||||||| ||||||||  |  | ||||| || ||||| ||| |||| |||||| | ||
Sbjct  1344  CACTGGACACCAGAAGGACATCGCAAGAGGGGCAGACCCAAAGTGACCTGGAGACGCACA  1285

Query  184   GTAGAG  189
             ||||||
Sbjct  1284  GTAGAG  1279
```

>**gb|AASC02048108.1|** D Aplysia californica cont2.48107, whole genome shotgun sequence
Length=19131

```
 Score = 69.8 bits (76),  Expect = 2e-08
 Identities = 104/148 (70%), Gaps = 0/148 (0%)
 Strand=Plus/Plus

Query  41    CCACAACTATAATAAGAAGACGCTGGAGGTGGATTCGGCATGTCCTACGGAAAGGCCAGG  100
             |||| |  |||| ||| ||| | ||||| |||||| | ||||| ||  | ||||  |  |
Sbjct  7226  CCACCATAATAAAAAGGAGAAGATGGAGATGGATTGGACATGTTCTGAGAAAAGAACCAG  7285

Query  101   GGGATATCACAAAAACAGCACTCCACTGGACCCCAGAAGGTAAAAGAAAGAGAGGAAGAC  160
                 |||    || || || ||||||||||| || |||||  |  | ||||| || ||||
Sbjct  7286  ACTCTATTGTGAAGACTGCCCTCCACTGGACACCCGAAGGACATCGCAAGAGGGGCAGAC  7345

Query  161   CTAAAATGACATGGAGAAGAACTGTAGA  188
             | ||| |||| |||||| | || |||||
Sbjct  7346  CCAAAGTGACCTGGAGACGCACAGTAGA  7373
```

>**dbj|BAAF04105806.1|** D Oryzias latipes DNA, contig105806 in scaffold746, strain: Hd-rR,
whole genome shotgun sequence
Length=16191

```
 Score = 69.8 bits (76),  Expect = 2e-08
 Identities = 109/154 (70%), Gaps = 4/154 (2%)
 Strand=Plus/Minus

Query  44     CAACTATAATAAGAAGACGCTGGAGGTGGATTCGGCATGTCCTACGGAAAG--GCCAGGG  101
              ||| |||  | | |||||||||||  |||||| | ||||| ||  |||  |  | ||
Sbjct  13701  CAATTATCCTCAAAAGACGCTGGACATGGATTGGACATGTACTCGGGAGTGAAGACAACA  13642

Query  102    GGATATCACAAAAACAGCACTCCACTGGACCCCAGAAGGTAAAAGAAAGAGAGGAAGACC  161
                |||  ||||| ||||| || || ||||| ||||| || |||||||  |||||| | ||
Sbjct  13641  CAATA--ACAAAGACAGCGCTACATTGGACACCAGATGGAAAAAGAACAAGAGGACGGCC  13584

Query  162    TAAAATGACATGGAGAAGAACTGTAGAGGCAGAG  195
               || || || ||| |  |||| ||||| || |||
Sbjct  13583  GAAGATCACCTGGCGCCGAACAGTAGAAGCTGAG  13550
```

>**gb|ACQM01054906.1|** D Saccoglossus kowalevskii Contig55035, whole genome shotgun sequence
Length=44668

```
 Score = 68.0 bits (74),  Expect = 8e-08
 Identities = 61/77 (79%), Gaps = 0/77 (0%)
 Strand=Plus/Plus

Query  106    ATCACAAAAACAGCACTCCACTGGACCCCAGAAGGTAAAAGAAAGAGAGGAAGACCTAAA  165
              ||| | |||||||||||   |||||| ||| | ||| || |||| ||||| || || |||
Sbjct  22125  ATCCCCAAAACAGCACTTTGCTGGACGCCAAAGGGTCAACGAAACAGAGGGAGGCCAAAA  22184

Query  166    ATGACATGGAGAAGAAC  182
              |  ||||||||||||||
Sbjct  22185  ACCACATGGAGAAGAAC  22201
```

>**gb|AASC02012961.1|** D Aplysia californica cont2.12960, whole genome shotgun sequence
Length=23384

```
 Score = 68.0 bits (74),  Expect = 8e-08
 Identities = 112/162 (69%), Gaps = 0/162 (0%)
 Strand=Plus/Minus

Query  28    CAACAAGATATAGCCACAACTATAATAAGAAGACGCTGGAGGTGGATTCGGCATGTCCTA  87
             ||||| || ||  |||  |  |||| ||| ||| | ||||  |||||| ||||||| ||
Sbjct  2787  CAACAGGACATGACCATCATAATAAAAAGGAGAAGATGGATATGGATTGGGCATGTTCTG  2728

Query  88    CGGAAAGGCCAGGGGGATATCACAAAAACAGCACTCCACTGGACCCCAGAAGGTAAAAGA  147
              | | ||  |  |    |||    || || || ||||||||||| || ||||| ||
Sbjct  2727  AGAAGAGAACCAGACTCTATTGTGAAGACTGCCCTCCACTGGACACCCGAAGGAAATCTC  2668

Query  148   AAGAGAGGAAGACCTAAAATGACATGGAGAAGAACTGTAGAG  189
             ||||| || ||||| ||| |||| |||||| | || ||||||
Sbjct  2667  AAGAGGGGCAGACCCAAAGTGACCTGGAGACGCACAGTAGAG  2626
```

>**gb|AASC02016796.1|** D Aplysia californica cont2.16795, whole genome shotgun sequence
Length=30874

```
 Score = 68.0 bits (74),  Expect = 8e-08
 Identities = 112/162 (69%), Gaps = 0/162 (0%)
 Strand=Plus/Plus

Query  28     CAACAAGATATAGCCACAACTATAATAAGAAGACGCTGGAGGTGGATTCGGCATGTCCTA  87
              ||| | || ||  |||| |  |||| ||| ||| | ||||| |||||| | ||||| ||
Sbjct  19171  CAAGAGGAAATGACCACTATAATAACAAGGAGAAGATGGAGATGGATTGGACATGTTCTG  19230

Query  88     CGGAAAGGCCAGGGGGATATCACAAAAACAGCACTCCACTGGACCCCAGAAGGTAAAAGA  147
```

```
              | | ||  |  |    |||   ||| |  || ||||||||||| || |||||| |  |
Sbjct  19231  TGAAGAGAACCTGACTCTATTGTAAAGATTGCCCTCCACTGGACACCCGAAGGTCATCGC  19290

Query  148    AAGAGAGGAAGACCTAAAATGACATGGAGAAGAACTGTAGAG  189
              ||||| | |||||| | | |||| |||||| | || ||||||
Sbjct  19291  AAGAGGGAAAGACCCACAGTGACCTGGAGACGCACAGTAGAG  19332
```

>**gb|AASC02036896.1|** Aplysia californica cont2.36895, whole genome shotgun sequence
Length=6427

```
 Score = 68.0 bits (74),  Expect = 8e-08
 Identities = 112/162 (69%), Gaps = 0/162 (0%)
 Strand=Plus/Minus

Query  28    CAACAAGATATAGCCACAACTATAATAAGAAGACGCTGGAGGTGGATTCGGCATGTCCTA  87
             ||| | || ||  |||| |  |||| ||| ||| | ||||| |||||| ||||||| ||
Sbjct  2477  CAAGAGGACATGACCACTATAATAAAAAGGAGAAGATGGAGATGGATTGGGCATGTTCTG  2418

Query  88    CGGAAAGGCCAGGGGGATATCACAAAAACAGCACTCCACTGGACCCCAGAAGGTAAAAGA  147
              | | ||  |     | |||    || || || ||||||||||| || ||||| ||  |
Sbjct  2417  AGAAGAGAACCACACGCTATTGTGAAGACTGCCCTCCACTGGACACCCGAAGGAAATCGC  2358

Query  148   AAGAGAGGAAGACCTAAAATGACATGGAGAAGAACTGTAGAG  189
             || |  |||||||| ||| |||| |||||| |  | ||||||
Sbjct  2357  AAAAAGGGAAGACCCAAAGTGACCTGGAGATGCTCAGTAGAG  2316
```

>**gb|AASC02040053.1|** Aplysia californica cont2.40052, whole genome shotgun sequence
Length=24458

```
 Score = 68.0 bits (74),  Expect = 8e-08
 Identities = 112/162 (69%), Gaps = 0/162 (0%)
 Strand=Plus/Minus

Query  28     CAACAAGATATAGCCACAACTATAATAAGAAGACGCTGGAGGTGGATTCGGCATGTCCTA  87
              ||| | || ||  |||| |  |||| ||  ||| | ||||| | |||| ||||||| |||
Sbjct  18218  CAAAAGGACATGACCACCATAATAACAAAGAGAAGATGGAGATAGATTGGGCATGTTCTA  18159

Query  88     CGGAAAGGCCAGGGGGATATCACAAAAACAGCACTCCACTGGACCCCAGAAGGTAAAAGA  147
               | | ||  |  |  | |||   ||| || || ||||| ||||| || |||||  |  |
Sbjct  18158  AGAAGAGAACCAGACGCTATTGTAAAGACTGCCCTCCAATGGACACCCGAAGGACATCGC  18099

Query  148    AAGAGAGGAAGACCTAAAATGACATGGAGAAGAACTGTAGAG  189
              || || || ||||| ||| |||| |||||| | || ||||||
Sbjct  18098  AATAGGGGCAGACCCAAAGTGACCTGGAGATGCACAGTAGAG  18057
```

>**gb|AASC02043334.1|** Aplysia californica cont2.43333, whole genome shotgun sequence
Length=76695

Sort alignments for this subject sequence by:
E value **Score** **Percent identity**
Query start position **Subject start position**

```
 Score = 68.0 bits (74),  Expect = 8e-08
 Identities = 104/147 (70%), Gaps = 1/147 (0%)
 Strand=Plus/Plus

Query  43     ACAACTATAATAAGAAGACGCTGGAGGTGGATTCGGCATGTCCTACGGAAAGG-CCAGGG  101
              ||||| ||||||| |||| | ||||| |||||| | ||||| ||  | | ||  ||||
Sbjct  35368  ACAACCATAATAAAAAGAAGATGGAGATGGATTGGACATGTTCTGAGAAGAGAACCAGAC  35427

Query  102    GGATATCACAAAAACAGCACTCCACTGGACCCCAGAAGGTAAAAGAAAGAGAGGAAGACC  161
                 ||| |  || || || ||||||||||| || |||||     | || || || |||||
Sbjct  35428  TCTTATTATGAAGACTGCCCTCCACTGGACACCCGAAGGACGTCGCAAAAGGGGCAGACC  35487

Query  162    TAAAATGACATGGAGAAGAACTGTAGA  188
               ||| | || |||||| | || |||||
Sbjct  35488  CAAAGTAACCTGGAGACGCACAGTAGA  35514


 Score = 46.4 bits (50),  Expect = 0.26
 Identities = 51/68 (75%), Gaps = 0/68 (0%)
 Strand=Plus/Plus

Query  127    TGGACCCCAGAAGGTAAAAGAAAGAGAGGAAGACCTAAAATGACATGGAGAAGAACTGTA  186
              |||| |||| |||| ||| | || |  |||||||| || |||||||||||  || | |||
Sbjct  35993  TGGAACCCACAAGGAAAACGTAAAAATGGAAGACCAAAGATGACATGGAGGCGAGCGGTA  36052

Query  187    GAGGCAGA  194
               | || ||
Sbjct  36053  CAAGCGGA  36060
```

>**gb|AASC02051169.1|** Aplysia californica cont2.51168, whole genome shotgun sequence
Length=7080

```
 Score = 68.0 bits (74),  Expect = 8e-08
 Identities = 114/163 (69%), Gaps = 4/163 (2%)
 Strand=Plus/Plus

Query  28    CAACAAGATATAGCCACAACTATAATAAGAAGACGCTGGAGGTGGATTCGGCATGTCCTA  87
             ||| | || ||  |||| |  |||| ||| ||| | ||||| |||||| | ||||| ||
Sbjct  6088  CAAGAGGACATGACCACCATAATAAAAAGGAGAAGATGGAGATGGATTGGACATGTTCTG  6147

Query  88    CGGAAAGG-CCAGGGGGATATCACAAAAACAGCACTCCACTGGACCCCAGAAGGTAAAAG  146
              | | ||  ||||     |||   ||| || |||||||||||||| ||  ||||  |  |
```

```
Sbjct  6148   AGAAGAGAACCAGAC---TATTGTAAAGACTGCACTCCACTGGACACCCAAAGGACATCG  6204

Query  147    AAAGAGAGGAAGACCTAAAATGACATGGAGAAGAACTGTAGAG  189
               || || || ||||| ||| |||| |||||| | || ||||||
Sbjct  6205   CAAAAGGGGCAGACCCAAAGTGACCTGGAGACGCACAGTAGAG  6247
```

>**gb|AASC02059661.1|** Aplysia californica cont2.59660, whole genome shotgun sequence
Length=14977

```
 Score = 68.0 bits (74),  Expect = 8e-08
 Identities = 112/162 (69%), Gaps = 0/162 (0%)
 Strand=Plus/Minus

Query  28     CAACAAGATATAGCCACAACTATAATAAGAAGACGCTGGAGGTGGATTCGGCATGTCCTA  87
              ||| | || ||  |||| |  |||| ||| ||| | ||||| |||||| | ||||| ||
Sbjct  4766   CAAGAGGACATGACCACCATAATAAAAAGGAGAAGATGGAGATGGATTGGACATGTTCTG  4707

Query  88     CGGAAAGGCCAGGGGGATATCACAAAAACAGCACTCCACTGGACCCCAGAAGGTAAAAGA  147
               | ||||  |  |    |||    || || || ||||||||||| || |||||     ||
Sbjct  4706   AGAAAAGAACCAGACTCTATTGTGAAGACTGCCCTCCACTGGACACCCGAAGGACGTCGA  4647

Query  148    AAGAGAGGAAGACCTAAAATGACATGGAGAAGAACTGTAGAG  189
              || || || ||||| ||| |||| |||||| | || ||||||
Sbjct  4646   AAAAGGGGCAGACCCAAAGTGACCTGGAGACGCACAGTAGAG  4605
```

>**dbj|BAAF04001490.1|** Oryzias latipes DNA, contig1490 in scaffold2, strain: Hd-rR, whole genome shotgun sequence
Length=17815

```
 Score = 68.0 bits (74),  Expect = 8e-08
 Identities = 129/188 (68%), Gaps = 4/188 (2%)
 Strand=Plus/Plus

Query  10     CTTTTCATCAGATGCCAACAACAAGATATAGCCACAACTATAATAAGAAGACGCTGGAGG  69
              || ||| || |  || | ||| |||| ||  |  ||| |||  | | |||||||||||
Sbjct  7927   CTATTCTTCTGCAGCAATCAAGAAGACATTTCATCAATTATCCTCAAAAGACGCTGGACA  7986

Query  70     TGGATTCGGCATGTCCTACGGAAAG--GCCAGGGGGATATCACAAAAACAGCACTCCACT  127
              |||||| | ||||| ||  |||  |  | ||     |||  | ||| |||||||| || |
Sbjct  7987   TGGATTGGACATGTACTCAGGAGTGAAGACAACACAATA--ATAAAGACAGCACTACATT  8044

Query  128    GGACCCCAGAAGGTAAAAGAAAGAGAGGAAGACCTAAAATGACATGGAGAAGAACTGTAG  187
              |||| ||||| || |||||||| | |||| | ||  | || || ||| |  |||| ||||
Sbjct  8045   GGACACCAGATGGAAAAAGAAAAAAAGGACGGCCATAGATCACCTGGCGCCGAACAGTAG  8104

Query  188    AGGCAGAG  195
              | || |||
Sbjct  8105   AAGCCGAG  8112
```

>**dbj|BAAF04083036.1|** Oryzias latipes DNA, contig83036 in scaffold286, strain: Hd-rR, whole genome shotgun sequence
Length=8214

```
 Score = 68.0 bits (74),  Expect = 8e-08
 Identities = 114/164 (69%), Gaps = 6/164 (3%)
 Strand=Plus/Plus

Query  28     CAACAAGATATAGCCACAACTATAATAAGAAGACGCTGGAGGTGGATTCGGCATGTCCTA  87
              ||| |||| ||  |  ||| |||  | | |||||||||||  |||||| | ||||| ||
Sbjct  7137   CAAGAAGACATGTCATCAATTATCCTCAAAAGACGCTGGACATGGATTGGACATGTACTC  7196

Query  88     CGGA---AAGGCCAGGGGGATATCACAAAAACAGCACTCCACTGGACCCCAGAAGGTAAA  144
               |||   ||| |||       || | ||  |||||||| || ||||| ||||| || |||
Sbjct  7197   AGGAGTGAAGACCATACA---ATAATAAGGACAGCACTACATTGGACACCAGATGGAAAA  7253

Query  145    AGAAAGAGAGGAAGACCTAAAATGACATGGAGAAGAACTGTAGA  188
              ||||| | |||| | || || || || ||| |  |||| |||||
Sbjct  7254   AGAAAAAAAGGACGGCCGAAGATCACCTGGCGCCGAACAGTAGA  7297
```

>**dbj|BAAF04102545.1|** Oryzias latipes DNA, contig102545 in scaffold615, strain: Hd-rR, whole genome shotgun sequence
Length=1382

```
 Score = 68.0 bits (74),  Expect = 8e-08
 Identities = 125/182 (68%), Gaps = 6/182 (3%)
 Strand=Plus/Minus

Query  10     CTTTTCATCAGATGCCAACAACAAGATATAGCCACAACTATAATAAGAAGACGCTGGAGG  69
              || ||| || |  || | ||| |||| ||  |  ||| |||  | | |||||||||||
Sbjct  1095   CTATTCTTCCGCAGCAATCAAGAAGACATGTCATCAATTATCCTCAAAAGACGCTGGACA  1036

Query  70     TGGATTCGGCATGTCCTACGGA---AAGGCCAGGGGGATATCACAAAAACAGCACTCCAC  126
              |||||| | ||||| ||  |||   ||| |||       || | ||| |||||||| ||
Sbjct  1035   TGGATTGGACATGTACTCAGGAGTGAAGACCACACA---ATAATAAAGACAGCACTACAT  979

Query  127    TGGACCCCAGAAGGTAAAAGAAAGAGAGGAAGACCTAAAATGACATGGAGAAGAACTGTA  186
              ||||| ||| | || |||||||| | |||| | || || || || ||| | | ||| |||
Sbjct  978    TGGACACCAAATGGAAAAAGAAAAAAAGGACGGCCGAAGATCACCTGGCGCAAAACGGTA  919

Query  187    GA  188
              ||
Sbjct  918    GA  917
```

```
>dbj|BAAF04112029.1|  Oryzias latipes DNA, contig112029 in scaffold1180, strain: Hd-rR,
whole genome shotgun sequence
Length=4916

 Score = 68.0 bits (74),  Expect = 8e-08
 Identities = 125/182 (68%), Gaps = 6/182 (3%)
 Strand=Plus/Plus

Query  10   CTTTTCATCAGATGCCAACAACAAGATATAGCCACAACTATAATAAGAAGACGCTGGAGG  69
            || ||| || |  || | ||| |||| ||  |  ||| |||  | | |||||||||||
Sbjct  741  CTATTCCTCCGCAGCAATCAAGAAGACATGTCATCAATTATCCTCAAAAGACGCTGGACA  800

Query  70   TGGATTCGGCATGTCCTACGGA---AAGGCCAGGGGGATATCACAAAAACAGCACTCCAC  126
            |||||| | ||| | ||  |||   ||| |||       || | ||| |||||||| ||
Sbjct  801  TGGATTGGACATATACTCAGGAGTGAAGACCACACA---ATAATAAAGACAGCACTACAT  857

Query  127  TGGACCCCAGAAGGTAAAAGAAAGAGAGGAAGACCTAAAATGACATGGAGAAGAACTGTA  186
            ||||| ||||| || |||||||| | |||| | || || || || ||| |  |||| |||
Sbjct  858  TGGACACCAGATGGAAAAAGAAAAAAAGGACGGCCGAAGATCACCTGGCGCCGAACAGTA  917

Query  187  GA  188
            ||
Sbjct  918  GA  919

>dbj|BAAE01274428.1|  Oryzias latipes DNA, contig274338 in scaffold27113, strain: HNI,
whole genome shotgun sequence
Length=936

 Score = 68.0 bits (74),  Expect = 8e-08
 Identities = 125/182 (68%), Gaps = 6/182 (3%)
 Strand=Plus/Minus

Query  10   CTTTTCATCAGATGCCAACAACAAGATATAGCCACAACTATAATAAGAAGACGCTGGAGG  69
            || ||| || |  || | ||| |||| ||  |  ||| |||  | | |||||||||||
Sbjct  318  CTATTCCTCCGCAGCAATCAAGAAGACATGTCATCAATTATCCTCAAAAGACGCTGGACA  259

Query  70   TGGATTCGGCATGTCCTACGGA---AAGGCCAGGGGGATATCACAAAAACAGCACTCCAC  126
            |||||  | ||||| ||  |||   ||| |||       || | ||| |||||||| ||
Sbjct  258  TGGATAGGACATGTACTCAGGAGTGAAGACCACACA---ATAATAAAGACAGCACTACAT  202

Query  127  TGGACCCCAGAAGGTAAAAGAAAGAGAGGAAGACCTAAAATGACATGGAGAAGAACTGTA  186
            ||||| ||||| || |||||||| | |||| | || || || || ||| |  |||| |||
Sbjct  201  TGGACACCAGATGGAAAAAGAAAAAAAGGACGGCCGAAGATCACCTGGCGCCGAACAGTA  142

Query  187  GA  188
            ||
Sbjct  141  GA  140

>dbj|BAAE01229859.1|  Oryzias latipes DNA, contig229807 in scaffold16894, strain: HNI,
whole genome shotgun sequence
Length=997

 Score = 68.0 bits (74),  Expect = 8e-08
 Identities = 125/182 (68%), Gaps = 6/182 (3%)
 Strand=Plus/Minus

Query  10   CTTTTCATCAGATGCCAACAACAAGATATAGCCACAACTATAATAAGAAGACGCTGGAGG  69
            || ||| || |  || | ||| |||| ||  |  ||| |||  | | |||||||||||
Sbjct  695  CTATTCCTCCGCAGCAATCAAGAAGACATGTCATCAATTATCCTCAAAAGACGCTGGACA  636

Query  70   TGGATTCGGCATGTCCTACGGA---AAGGCCAGGGGGATATCACAAAAACAGCACTCCAC  126
            |||||  | ||||| ||  |||   ||| |||       || | ||| |||||||| ||
Sbjct  635  TGGATAGGACATGTACTCAGGAGTGAAGACCACACA---ATAATAAAGACAGCACTACAT  579

Query  127  TGGACCCCAGAAGGTAAAAGAAAGAGAGGAAGACCTAAAATGACATGGAGAAGAACTGTA  186
            ||||| ||||| || |||||||| | |||| | || || || || ||| |  |||| |||
Sbjct  578  TGGACACCAGATGGAAAAAGAAAAAAAGGACGGCCGAAGATCACCTGGCGCCGAACAGTA  519

Query  187  GA  188
            ||
Sbjct  518  GA  517

>dbj|BAAE01149167.1|  Oryzias latipes DNA, contig149040 in scaffold7529, strain: HNI,
whole genome shotgun sequence
Length=5789

 Score = 68.0 bits (74),  Expect = 8e-08
 Identities = 125/182 (68%), Gaps = 6/182 (3%)
 Strand=Plus/Plus

Query  10   CTTTTCATCAGATGCCAACAACAAGATATAGCCACAACTATAATAAGAAGACGCTGGAGG  69
            || ||| || |  || | ||| |||| ||  |  ||| |||  | | |||||||||||
Sbjct  285  CTATTCCTCCGCAGCAATCAAGAAGACATGTCATCAATTATCCTCAAAAGACGCTGGACA  344

Query  70   TGGATTCGGCATGTCCTACGGA---AAGGCCAGGGGGATATCACAAAAACAGCACTCCAC  126
            |||||  | ||||| ||  |||   ||| |||       || | ||| |||||||| ||
Sbjct  345  TGGATAGGACATGTACTCAGGAGTGAAGACCACACA---ATAATAAAGACAGCACTACAT  401

Query  127  TGGACCCCAGAAGGTAAAAGAAAGAGAGGAAGACCTAAAATGACATGGAGAAGAACTGTA  186
            ||||| ||||| || |||||||| | |||| | || || || || ||| |  |||| |||
Sbjct  402  TGGACACCAGATGGAAAAAGAAAAAAAGGACGGCCGAAGATCACCTGGCGCCGAACAGTA  461

Query  187  GA  188
            ||
Sbjct  462  GA  463
```

>**dbj|BAAE01154250.1|** Oryzias latipes DNA, contig154165 in scaffold7957, strain: HNI, whole genome shotgun sequence
Length=1001

```
 Score = 68.0 bits (74),  Expect = 8e-08
 Identities = 125/182 (68%), Gaps = 6/182 (3%)
 Strand=Plus/Minus

Query  10   CTTTTCATCAGATGCCAACAACAAGATATAGCCACAACTATAATAAGAAGACGCTGGAGG  69
            || ||| || |  || | ||| |||| ||  |  ||| |||  | | |||||||||||
Sbjct  732  CTATTCTTCCGCAGCAATCAAGAAGACATGTCATCAATTATCCTCAAAAGACGCTGGATA  673

Query  70   TGGATTCGGCATGTCCTACGGA---AAGGCCAGGGGGATATCACAAAAACAGCACTCCAC  126
            || ||| | ||||| ||  |||   |||  ||        |||||||| ||||||| ||
Sbjct  672  TGTATTGGACATGTACTCAGGAGTGAAGATCACACA---ATCACAAAGACAGCACTACAT  616

Query  127  TGGACCCCAGAAGGTAAAAGAAAGAGAGGAAGACCTAAAATGACATGGAGAAGAACTGTA  186
            ||||| ||||| || |||||||| | ||||   || || || || ||| |  |||| |||
Sbjct  615  TGGACACCAGATGGAAAAAGAAAAAAAGGACAGCCGAAGATCACCTGGCGCCGAACAGTA  556

Query  187  GA  188
            ||
Sbjct  555  GA  554
```

>**dbj|BAAE01123166.1|** Oryzias latipes DNA, contig123078 in scaffold5559, strain: HNI, whole genome shotgun sequence
Length=1123

```
 Score = 68.0 bits (74),  Expect = 8e-08
 Identities = 125/182 (68%), Gaps = 6/182 (3%)
 Strand=Plus/Plus

Query  10    CTTTTCATCAGATGCCAACAACAAGATATAGCCACAACTATAATAAGAAGACGCTGGAGG  69
             || ||| || |  || | ||| |||| ||  |  ||| |||  | | |||||||||||
Sbjct  939   CTATTCCTCCGCAGCAATCAAGAAGACATGTCATCAATTATCCTCAAAAGACGCTGGACA  998

Query  70    TGGATTCGGCATGTCCTACGGA---AAGGCCAGGGGGATATCACAAAAACAGCACTCCAC  126
             |||||  | ||||| ||  |||   ||| |||        || | ||| |||||||| ||
Sbjct  999   TGGATAGGACATGTACTCAGGAGTGAAGACCACACA---ATAATAAAGACAGCACTACAT  1055

Query  127   TGGACCCCAGAAGGTAAAAGAAAGAGAGGAAGACCTAAAATGACATGGAGAAGAACTGTA  186
             ||||| ||||| || |||||||| | |||| | || || || || ||| |  |||| |||
Sbjct  1056  TGGACACCAGATGGAAAAAGAAAAAAAGGACGGCCGAAGATCACCTGGCGCCGAACAGTA  1115

Query  187   GA  188
             ||
Sbjct  1116  GA  1117
```

>**dbj|BAAE01123168.1|** Oryzias latipes DNA, contig123080 in scaffold5559, strain: HNI, whole genome shotgun sequence
Length=5925

```
 Score = 68.0 bits (74),  Expect = 8e-08
 Identities = 125/182 (68%), Gaps = 6/182 (3%)
 Strand=Plus/Plus

Query  10   CTTTTCATCAGATGCCAACAACAAGATATAGCCACAACTATAATAAGAAGACGCTGGAGG  69
            || ||| || |  || | ||| |||| ||  |  ||| |||  | | |||||||||||
Sbjct  366  CTATTCCTCCGCAGCAATCAAGAAGACATGTCATCAATTATCCTCAAAAGACGCTGGACA  425

Query  70   TGGATTCGGCATGTCCTACGGA---AAGGCCAGGGGGATATCACAAAAACAGCACTCCAC  126
            |||||  | ||||| ||  |||   ||| |||        || | ||| |||||||| ||
Sbjct  426  TGGATAGGACATGTACTCAGGAGTGAAGACCACACA---ATAATAAAGACAGCACTACAT  482

Query  127  TGGACCCCAGAAGGTAAAAGAAAGAGAGGAAGACCTAAAATGACATGGAGAAGAACTGTA  186
            ||||| ||||| || |||||||| | |||| | || || || || ||| |  |||| |||
Sbjct  483  TGGACACCAGATGGAAAAAGAAAAAAAGGACGGCCGAAGATCACCTGGCGCCGAACAGTA  542

Query  187  GA  188
            ||
Sbjct  543  GA  544
```

>**dbj|BAAE01093172.1|** Oryzias latipes DNA, contig92264 in scaffold3686, strain: HNI, whole genome shotgun sequence
Length=769

```
 Score = 68.0 bits (74),  Expect = 8e-08
 Identities = 125/182 (68%), Gaps = 6/182 (3%)
 Strand=Plus/Plus

Query  10   CTTTTCATCAGATGCCAACAACAAGATATAGCCACAACTATAATAAGAAGACGCTGGAGG  69
            || ||| || |  || | ||| |||| ||  |  ||| |||  | | |||||||||||
Sbjct  366  CTATTCCTCCGCAGCAATCAAGAAGACATGTCATCAATTATCCTCAAAAGACGCTGGACA  425

Query  70   TGGATTCGGCATGTCCTACGGA---AAGGCCAGGGGGATATCACAAAAACAGCACTCCAC  126
            |||||  | ||||| ||  |||   ||| |||        || | ||| |||||||| ||
Sbjct  426  TGGATAGGACATGTACTCAGGAGTGAAGACCACACA---ATAATAAAGACAGCACTACAT  482

Query  127  TGGACCCCAGAAGGTAAAAGAAAGAGAGGAAGACCTAAAATGACATGGAGAAGAACTGTA  186
            ||||| ||||| || |||||||| | |||| | || || || || ||| |  |||| |||
Sbjct  483  TGGACACCAGATGGAAAAAGAAAAAAAGGACGGCCGAAGATCACCTGGCGCCGAACAGTA  542

Query  187  GA  188
            ||
Sbjct  543  GA  544
```

```
>dbj|BAAE01079160.1|  Oryzias latipes DNA, contig79199 in scaffold2924, strain: HNI,
whole genome shotgun sequence
Length=2732

 Score = 68.0 bits (74),  Expect = 8e-08
 Identities = 63/80 (78%), Gaps = 0/80 (0%)
 Strand=Plus/Minus

Query  109   ACAAAAACAGCACTCCACTGGACCCCAGAAGGTAAAAGAAAGAGAGGAAGACCTAAAATG  168
             ||||| |||||||| || ||||| ||||| || |||||||| | |||| |||| || ||
Sbjct  2606  ACAAAGACAGCACTACATTGGACACCAGATGGAAAAAGAAAAAAAGGACGACCGAAGATC  2547

Query  169   ACATGGAGAAGAACTGTAGA  188
             || ||| |  |||| |||||
Sbjct  2546  ACCTGGCGCCGAACAGTAGA  2527


>dbj|BAAE01049990.1|  Oryzias latipes DNA, contig49924 in scaffold1580, strain: HNI,
whole genome shotgun sequence
Length=3478

 Score = 68.0 bits (74),  Expect = 8e-08
 Identities = 114/164 (69%), Gaps = 6/164 (3%)
 Strand=Plus/Plus

Query  28    CAACAAGATATAGCCACAACTATAATAAGAAGACGCTGGAGGTGGATTCGGCATGTCCTA  87
             ||| |||| ||  |  ||| |||  | | |||||||||||  |||||  | ||||| ||
Sbjct  372   CAAGAAGACATGTCATCAATTATCCTCAAAAGACGCTGGACATGGATAGGACATGTACTC  431

Query  88    CGGA---AAGGCCAGGGGGATATCACAAAAACAGCACTCCACTGGACCCCAGAAGGTAAA  144
              |||   ||| |||       || | ||| |||||||| || ||||| ||||| || |||
Sbjct  432   AGGAGTGAAGACCACACA---ATAATAAAGACAGCACTACATTGGACACCAGATGGAAAA  488

Query  145   AGAAAGAGAGGAAGACCTAAAATGACATGGAGAAGAACTGTAGA  188
             ||||| | |||| | || || || || ||| |  |||| |||||
Sbjct  489   AGAAAAAAAGGACGGCCGAAGATCACCTGGCGCCGAACAGTAGA  532


>gb|AAGJ02119249.1|  Strongylocentrotus purpuratus Ctg119529, whole genome shotgun
sequence
Length=1063

 GENE ID: 591735 LOC591735 | hypothetical LOC591735
[Strongylocentrotus purpuratus]

 Score = 68.0 bits (74),  Expect = 8e-08
 Identities = 60/75 (80%), Gaps = 0/75 (0%)
 Strand=Plus/Plus

Query  114   AACAGCACTCCACTGGACCCCAGAAGGTAAAAGAAAGAGAGGAAGACCTAAAATGACATG  173
             |||||| ||||| ||||| |||||||| ||||||||||| ||| | || ||||  ||  |
Sbjct  733   AACAGCCCTCCAATGGACACCAGAAGGCAAAAGAAAGAGGGGATGTCCGAAAAACACTCG  792

Query  174   GAGAAGAACTGTAGA  188
             |  ||||||||| ||
Sbjct  793   GCCAAGAACTGTGGA  807

>gb|AAGJ02135339.1|  Strongylocentrotus purpuratus Ctg135635, whole genome shotgun
sequence
Length=1735

 Score = 68.0 bits (74),  Expect = 8e-08
 Identities = 57/70 (81%), Gaps = 0/70 (0%)
 Strand=Plus/Minus

Query  126   CTGGACCCCAGAAGGTAAAAGAAAGAGAGGAAGACCTAAAATGACATGGAGAAGAACTGT  185
             |||||| |||||||| ||||||||||| ||| | || ||||  || ||| ||||||||||
Sbjct  1273  CTGGACACCAGAAGGCAAAAGAAAGAGGGGACGTCCAAAAACCACTTGGCGAAGAACTGT  1214

Query  186   AGAGGCAGAG  195
              || | ||||
Sbjct  1213  GGAAGGAGAG  1204

>gb|ACQM01008863.1|  Saccoglossus kowalevskii Contig8891, whole genome shotgun sequence
Length=7222

 Score = 66.2 bits (72),  Expect = 3e-07
 Identities = 57/71 (80%), Gaps = 0/71 (0%)
 Strand=Plus/Minus

Query  112   AAAACAGCACTCCACTGGACCCCAGAAGGTAAAAGAAAGAGAGGAAGACCTAAAATGACA  171
             ||||||||||| | |||||| ||| | ||| || |||| ||||| || || ||||  |||
Sbjct  210   AAAACAGCACTTCGCTGGACACCAAAGGGTCAACGAAACAGAGGGAGGCCAAAAACCACA  151

Query  172   TGGAGAAGAAC  182
             ||||||| |||
Sbjct  150   TGGAGAACAAC  140


>gb|ACQM01064549.1|  Saccoglossus kowalevskii Contig64697, whole genome shotgun sequence
Length=29739

 Score = 66.2 bits (72),  Expect = 3e-07
 Identities = 57/71 (80%), Gaps = 0/71 (0%)
```

[illegible]

```
 Strand=Plus/Plus

Query  112    AAAACAGCACTCCACTGGACCCCAGAAGGTAAAAGAAAGAGAGGAAGACCTAAAATGACA  171
              ||||||||||| | |||||| ||| |  || ||||||| ||| |||| || ||||   ||
Sbjct  20372  AAAACAGCACTTCGCTGGACGCCACAGTGTCAAAGAAACAGAAGAAGGCCAAAAACCGCA  20431

Query  172    TGGAGAAGAAC  182
              |||||||||||
Sbjct  20432  TGGAGAAGAAC  20442
```

>**gb|ACQM01132338.1|** Saccoglossus kowalevskii Contig132621, whole genome shotgun sequence
Length=1842

```
 Score = 66.2 bits (72),  Expect = 3e-07
 Identities = 57/71 (80%), Gaps = 0/71 (0%)
 Strand=Plus/Minus

Query  112   AAAACAGCACTCCACTGGACCCCAGAAGGTAAAAGAAAGAGAGGAAGACCTAAAATGACA  171
             ||||||||||| | |||||| ||| | ||| |  |||| ||||| || || ||||  |||
Sbjct  1396  AAAACAGCACTTCGCTGGACACCACAGGGTCATCGAAACAGAGGGAGGCCAAAAACCACA  1337

Query  172   TGGAGAAGAAC  182
             |||||||||||
Sbjct  1336  TGGAGAAGAAC  1326
```

>**gb|AASC02012236.1|** Aplysia californica cont2.12235, whole genome shotgun sequence
Length=7642

```
 Score = 66.2 bits (72),  Expect = 3e-07
 Identities = 99/141 (70%), Gaps = 0/141 (0%)
 Strand=Plus/Minus

Query  49    ATAATAAGAAGACGCTGGAGGTGGATTCGGCATGTCCTACGGAAAGGCCAGGGGGATATC  108
             |||| ||| ||| | |||||||||||| | ||||| ||  ||| ||  |  |    |||
Sbjct  2400  ATAAAAAGGAGAAGGTGGAGGTGGATTGGACATGTTCTGAGGAGAGAACCAGACTCTATT  2341

Query  109   ACAAAAACAGCACTCCACTGGACCCCAGAAGGTAAAAGAAAGAGAGGAAGACCTAAAATG  168
                || || |||||| ||||||| || |||||     | || || || ||||| ||| ||
Sbjct  2340  GTGAAGACTGCACTCTACTGGACACCCGAAGGACGTCGCAAAAGGGGCAGACCCAAAGTG  2281

Query  169   ACATGGAGAAGAACTGTAGAG  189
             || |||||| | || ||||||
Sbjct  2280  ACCTGGAGACGCACAGTAGAG  2260
```

>**gb|AASC02013680.1|** Aplysia californica cont2.13679, whole genome shotgun sequence
Length=14036

```
 Score = 66.2 bits (72),  Expect = 3e-07
 Identities = 99/141 (70%), Gaps = 0/141 (0%)
 Strand=Plus/Plus

Query  49     ATAATAAGAAGACGCTGGAGGTGGATTCGGCATGTCCTACGGAAAGGCCAGGGGGATATC  108
              |||| ||| ||| | ||||| |||||| ||||||| ||  | | ||  |  |    |||
Sbjct  11862  ATAACAAGGAGAAGATGGAGATGGATTGGGCATGTTCTGAGAAGAGAACCAGACTCTATA  11921

Query  109    ACAAAAACAGCACTCCACTGGACCCCAGAAGGTAAAAGAAAGAGAGGAAGACCTAAAATG  168
                 || || || |||||||||||  | |||||  |  | ||||| || ||||| ||| ||
Sbjct  11922  GTGAAGACTGCCCTCCACTGGACATCCGAAGGACATCGCAAGAGGGGCAGACCCAAAGTG  11981

Query  169    ACATGGAGAAGAACTGTAGAG  189
              || |||||| | || ||||||
Sbjct  11982  ACCTGGAGACGCACAGTAGAG  12002
```

>**gb|AASC02013687.1|** Aplysia californica cont2.13686, whole genome shotgun sequence
Length=51227

```
 Score = 66.2 bits (72),  Expect = 3e-07
 Identities = 54/66 (81%), Gaps = 0/66 (0%)
 Strand=Plus/Plus

Query  127   TGGACCCCAGAAGGTAAAAGAAAGAGAGGAAGACCTAAAATGACATGGAGAAGAACTGTA  186
             ||||  ||| |||| ||| |||||| ||||||||| ||||||||||||||  || | |||
Sbjct  9590  TGGAATCCACAAGGAAAACGAAAGAAAGGAAGACCAAAAATGACATGGAGGCGAGCCGTA  9649

Query  187   GAGGCA  192
              |||||
Sbjct  9650  CAGGCA  9655
```

>**gb|AASC02020422.1|** Aplysia californica cont2.20421, whole genome shotgun sequence
Length=9046

```
 Score = 66.2 bits (72),  Expect = 3e-07
 Identities = 98/139 (70%), Gaps = 0/139 (0%)
 Strand=Plus/Plus

Query  51    AATAAGAAGACGCTGGAGGTGGATTCGGCATGTCCTACGGAAAGGCCAGGGGGATATCAC  110
             || ||| ||| | ||||| |||||| | ||||| ||  | | ||  |  |    |||
Sbjct  3020  AAAAAGGAGAAGATGGAGATGGATTGGACATGTTCTGAGAAGAGAACCAGACTCTATAGT  3079

Query  111   AAAAACAGCACTCCACTGGACCCCAGAAGGTAAAAGAAAGAGAGGAAGACCTAAAATGAC  170
              || || || ||||||||||| || ||||| ||  | ||||| || ||||| ||| ||||
Sbjct  3080  GAAGACTGCCCTCCACTGGACACCCGAAGGAAATCGCAAGAGGGGCAGACCCAAAGTGAC  3139
```

```
Query  171   ATGGAGAAGAACTGTAGAG  189
              |||||| | || ||||||
Sbjct  3140  CTGGAGACGCACAGTAGAG  3158
```

>**gb|AASC02024688.1|** Aplysia californica cont2.24687, whole genome shotgun sequence
Length=97462

```
 Score = 66.2 bits (72),  Expect = 3e-07
 Identities = 107/151 (70%), Gaps = 5/151 (3%)
 Strand=Plus/Minus

Query  43     ACAACTATAATAAGAAG---ACGCTGGAGGTGGATTCGGCATGTCCTACGGAAAG-GCCA  98
              ||||| ||||||| |||   | | ||||| |||||| | ||||| ||  | | || ||||
Sbjct  24907  ACAACCATAATAAAAAGGAGAAGGTGGAGATGGATTGGACATGTTCTGAGAAGAGAGCCA  24848

Query  99     GGGGGATATCACAAAAACAGCACTCCACTGGACCCCAGAAGGTAAAAGAAAGAGAGGAAG  158
              |     |||    || || || ||||||||||| || |||||     | ||||| || ||
Sbjct  24847  GACTC-TATTGTGAAGACTGCCCTCCACTGGACACCCGAAGGACGTCGCAAGAGGGGCAG  24789

Query  159    ACCTAAAATGACATGGAGAAGAACTGTAGAG  189
              ||| ||| |||| |||||| | || ||||||
Sbjct  24788  ACCCAAAGTGACCTGGAGACGCACAGTAGAG  24758
```

>**gb|AASC02031740.1|** Aplysia californica cont2.31739, whole genome shotgun sequence
Length=5530

```
 Score = 66.2 bits (72),  Expect = 3e-07
 Identities = 62/79 (78%), Gaps = 0/79 (0%)
 Strand=Plus/Minus

Query  111   AAAAACAGCACTCCACTGGACCCCAGAAGGTAAAAGAAAGAGAGGAAGACCTAAAATGAC  170
             ||| || || ||||||||||| || |||||  |  ||||||| || ||||| ||| ||||
Sbjct  2601  AAAGACTGCCCTCCACTGGACACCTGAAGGACATCGAAAGAGGGGCAGACCCAAAGTGAC  2542

Query  171   ATGGAGAAGAACTGTAGAG  189
              |||||| | || ||||||
Sbjct  2541  CTGGAGACGCACAGTAGAG  2523
```

>**gb|AASC02043777.1|** Aplysia californica cont2.43776, whole genome shotgun sequence
Length=13433

```
 Score = 66.2 bits (72),  Expect = 3e-07
 Identities = 99/141 (70%), Gaps = 0/141 (0%)
 Strand=Plus/Plus

Query  49     ATAATAAGAAGACGCTGGAGGTGGATTCGGCATGTCCTACGGAAAGGCCAGGGGGATATC  108
              |||| ||| ||| | ||||| |||||| ||||||| ||  | | ||  |  |    |||
Sbjct  12666  ATAACAAGGAGAAGATGGAGATGGATTGGGCATGTTCTGAGAAGAGAACCAGACTCTATT  12725

Query  109    ACAAAAACAGCACTCCACTGGACCCCAGAAGGTAAAAGAAAGAGAGGAAGACCTAAAATG  168
                ||| || || ||||||||||| || ||| |  |  | ||||| || ||||| ||| ||
Sbjct  12726  GTAAAGACTGCCCTCCACTGGACACCTGAATGACATCGCAAGAGGGGCAGACCCAAAGTG  12785

Query  169    ACATGGAGAAGAACTGTAGAG  189
              || |||||| | || ||| ||
Sbjct  12786  ACCTGGAGACGCACAGTACAG  12806
```

>**gb|AASC02048297.1|** Aplysia californica cont2.48296, whole genome shotgun sequence
Length=8258

```
 Score = 66.2 bits (72),  Expect = 3e-07
 Identities = 114/165 (69%), Gaps = 3/165 (1%)
 Strand=Plus/Plus

Query  28    CAACAAGATATAGCCACAACTATAATAAGAAGACGCTGGAGGTGGATTCGGCATGTCCTA  87
             ||| |||| || ||||| |  |||| ||| ||| | |||||||||||| |||||||  ||
Sbjct  5738  CAAGAAGACATGGCCACCATAATAACAAGGAGAAGATGGAGGTGGATTGGGCATGTTTTA  5797

Query  88    CGGAAAGGCCAGGGGGATA---TCACAAAAACAGCACTCCACTGGACCCCAGAAGGTAAA  144
              | | ||  |  |   |||    | | |||  | || |||||||||||| || || |   |
Sbjct  5798  AGAAGAGAACCAGACTATATTGTAATAAAGCCTGCCCTCCACTGGACACCCGAGGAACAT  5857

Query  145   AGAAAGAGAGGAAGACCTAAAATGACATGGAGAAGAACTGTAGAG  189
              | ||||| || || || ||| |||| |||||| | || | ||||
Sbjct  5858  CGGAAGAGGGGCAGTCCCAAAGTGACCTGGAGACGCACAGCAGAG  5902
```

>**gb|AASC02062985.1|** Aplysia californica cont2.62984, whole genome shotgun sequence
Length=6783

```
 Score = 66.2 bits (72),  Expect = 3e-07
 Identities = 54/66 (81%), Gaps = 0/66 (0%)
 Strand=Plus/Minus

Query  127   TGGACCCCAGAAGGTAAAAGAAAGAGAGGAAGACCTAAAATGACATGGAGAAGAACTGTA  186
             ||||  ||| |||| ||| |||||| ||||||||| ||||||||||||||  || | |||
Sbjct  4744  TGGAATCCACAAGGAAAACGAAAGAAAGGAAGACCAAAAATGACATGGAGGCGAGCCGTA  4685

Query  187   GAGGCA  192
              |||||
Sbjct  4684  CAGGCA  4679
```

>**dbj|BAAF04033128.1|** Oryzias latipes DNA, contig33128 in scaffold54, strain: Hd-rR,

[illegible]

```
whole genome shotgun sequence
Length=1667

 Score = 66.2 bits (72),  Expect = 3e-07
 Identities = 108/154 (70%), Gaps = 4/154 (2%)
 Strand=Plus/Plus

Query  44   CAACTATAATAAGAAGACGCTGGAGGTGGATTCGGCATGTCCTACGGAAAG--GCCAGGG  101
            ||| |||  | | |||||||||||  |||||| | ||||| ||  |||  |  | ||
Sbjct  840  CAATTATCCTCAAAAGACGCTGGACATGGATTGGACATGTACTCAGGAGTGAAGACAACA  899

Query  102  GGATATCACAAAAACAGCACTCCACTGGACCCCAGAAGGTAAAAGAAAGAGAGGAAGACC  161
              |||  | ||| |||||||| || ||||| ||||| || |||||| | |||||| |  |
Sbjct  900  CAATA--ATAAAGACAGCACTACATTGGACACCAGATGGAAAAAGACAAAGAGGACGGGC  957

Query  162  TAAAATGACATGGAGAAGAACTGTAGAGGCAGAG  195
             || || || ||| |  |||| ||||| || |||
Sbjct  958  GAAGATCACCTGGCGCCGAACAGTAGAAGCCGAG  991
```

Select All Get selected sequences Distance tree of results Multiple alignment NEW
