# Supplementary material for: Promoter Complexity and Tissue-Specific Expression of Stress Response Components in Mytilus galloprovincialis, a Sessile Marine Invertebrate Species
Source: PLoS Comput Biol. 2010 Jul 8;6(7):e1000847. doi: 10.1371/journal.pcbi.1000847 (PMC2900285; doi:10.1371/journal.pcbi.1000847)
Supplement: Protocol S1 — 18 Supplement files plus an index file: 3 Supplementary figures, 2 Supplementary tables - referenced in text as Protocol S1; index provided with an explanation of the directory contents. (5.18 MB ZIP) [file pcbi.1000847.s001.zip › SUPPLEMENTS18/SupplFigure3a.pdf]

# BLAST Basic Local Alignment Search Tool

- 

Edit and Resubmit Save Search Strategies Formatting options Download

PSI blast Iteration 1

## ref|XP_290882.1| (266 letters)

Results for: ref|XP_290882.1 similar to HEAT SHOCK 70 KD PROTEIN C PRECURSOR [Homo sapiens](266aa)

Your BLAST job specified more than one input sequence. This box lets you choose which input sequence to show BLAST results for.

**Query ID**
gi|29728737|ref|XP_290882.1|
**Description**
similar to HEAT SHOCK 70 KD PROTEIN C PRECURSOR [Homo sapiens]
**Molecule type**
amino acid
**Query Length**
266

**Database Name**
nr
**Description**
All non-redundant GenBank CDS translations+PDB+SwissProt+PIR+PRF excluding environmental samples from WGS projects
**Program**
BLASTP 2.2.22+ Citation


Reference
Stephen F. Altschul, Thomas L. Madden, Alejandro A. Schäffer, Jinghui Zhang, Zheng Zhang, Webb Miller, and David J. Lipman (1997), "Gapped BLAST and PSI-BLAST: a new generation of protein database search programs", Nucleic Acids Res. 25:3389-3402.
Reference - compositional score matrix adjustment
Stephen F. Altschul, John C. Wootton, E. Michael Gertz, Richa Agarwala, Aleksandr Morgulis, Alejandro A. Schäffer, and Yi-Kuo Yu (2005) "Protein database searches using compositionally adjusted substitution matrices", FEBS J. 272:5101-5109.

Other reports: Search Summary [Taxonomy reports] [Distance tree of results] [Related Structures] [Multiple alignment] NEW

## Search Parameters

| | |
|---|---|
| Program | blastp |
| Word size | 3 |
| Expect value | 10 |
| Hitlist size | 500 |
| Gapcosts | 11,1 |
| Matrix | BLOSUM62 |
| Threshold | 11 |
| Composition-based stats | 2 |
| Filter string | F |
| Genetic Code | 1 |
| Window Size | 40 |

## Database

| | |
|---|---|
| Posted date | Oct 20, 2009 5:42 PM |
| Number of letters | 3,385,075,628 |
| Number of sequences | 9,920,714 |
| Entrez query | none |

## Karlin-Altschul statistics

| Params | Ungapped | Gapped |
|---|---|---|
| Lambda | 0.310194 | 0.267 |
| K | 0.12683 | 0.041 |
| H | 0.354829 | 0.14 |

## Results Statistics

| | |
|---|---|
| Length adjustment | 134 |
| Effective length of query | 132 |
| Effective length of database | 2055699952 |
| Effective search space | 271352393664 |
| Effective search space used | 271352393664 |

Graphic Summary
Show Conserved Domains

Putative conserved domains have been detected, click on the image below for detailed results.

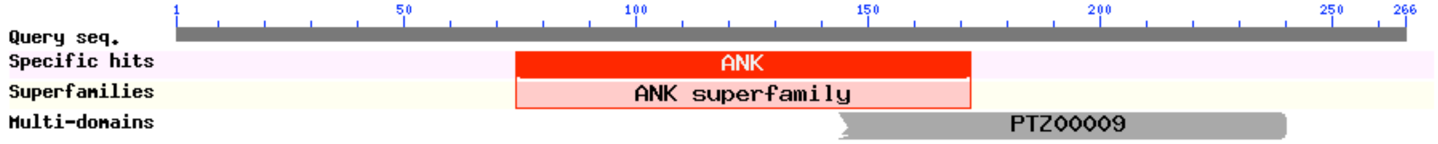


### Distribution of 108 Blast Hits on the Query Sequence

[?]

An overview of the database sequences aligned to the query sequence is shown. The score of each alignment is indicated by one of five different colors, which divides the range of scores into five groups. Multiple alignments on the same database sequence are connected by a striped line. Mousing over a hit sequence causes the definition and score to be shown in the window at the top, clicking on a hit sequence takes the user to the associated alignments. New: This graphic is an overview of database sequences aligned to the query sequence. Alignments are color-coded by score, within one of five score ranges. Multiple alignments on the same database sequence are connected by a dashed line. Mousing over an alignment shows the alignment definition and score in the box at the top. Clicking an alignment displays the alignment detail.

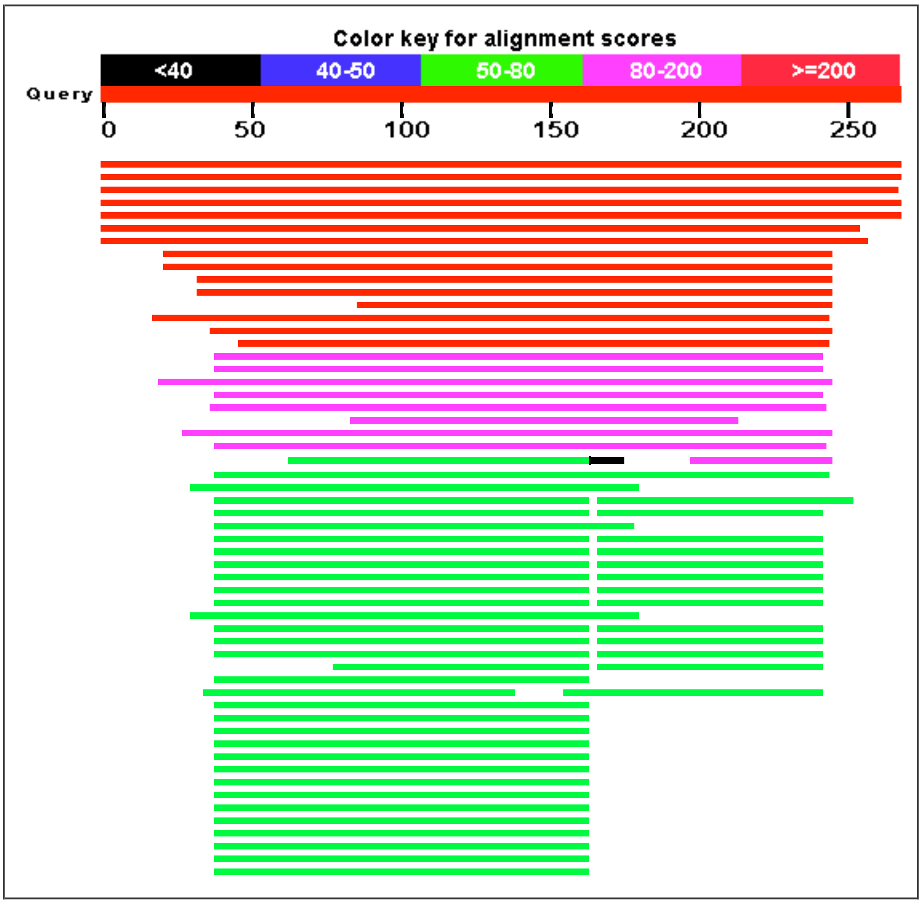

Descriptions

- NEW - alignment score below the threshold on the previous iteration
- ● - alignment was checked on the previous iteration

Run PSI-Blast iteration 2 with max

```
Sequences with    pattern at position  and  E-value BETTER than threshold
```

Sequences producing significant alignments:

| | Sequence | Description | Score (Bits) | E Value | |
|---|---|---|---|---|---|
| NEW | sp\|Q5TZF3.1\|ANR45_HUMAN | RecName: Full=Ankyrin repeat domain-c... | 549 | 1e-154 | G |
| NEW | ref\|NP_940895.1\| | ankyrin repeat domain 45 [Homo sapiens] >dbj... | 548 | 1e-154 | UG |
| NEW | gb\|EAW90951.1\| | hCG2039282 [Homo sapiens] | 546 | 7e-154 | |
| NEW | ref\|XP_001148842.1\| | PREDICTED: hypothetical protein [Pan trog... | 545 | 2e-153 | UG |
| NEW | ref\|XP_001101749.1\| | PREDICTED: similar to ankyrin repeat doma... | 500 | 5e-140 | UG |
| NEW | ref\|XP_853964.1\| | PREDICTED: similar to ankyrin repeat domain ... | 450 | 5e-125 | UG |
| NEW | ref\|XP_001493013.1\| | PREDICTED: similar to Ankyrin repeat doma... | 422 | 2e-116 | UG |
| NEW | gb\|EDM09419.1\| | ankyrin repeat domain 45 (predicted) [Rattus n... | 380 | 7e-104 | G |
| NEW | ref\|XP_213908.4\| | PREDICTED: similar to ankyrin repeat domain ... | 379 | 2e-103 | UG |
| NEW | sp\|Q810N6.1\|ANR45_MOUSE | RecName: Full=Ankyrin repeat domain-c... | 374 | 6e-102 | G |
| NEW | ref\|NP_082940.1\| | ankyrin repeat domain 45 [Mus musculus] >gb\|... | 373 | 1e-101 | UG |
| NEW | dbj\|BAB30413.1\| | unnamed protein product [Mus musculus] | 273 | 1e-71 | G |
| NEW | ref\|XP_001373170.1\| | PREDICTED: similar to hCG2039282 [Monodel... | 262 | 2e-68 | UG |
| NEW | ref\|NP_001088745.1\| | hypothetical protein LOC496009 [Xenopus l... | 218 | 6e-55 | UG |
| NEW | gb\|AAH84990.1\| | Ankrd45-prov protein [Xenopus tropicalis] | 206 | 1e-51 | G |
| NEW | emb\|CAQ14704.1\| | novel protein (zgc:112418) [Danio rerio] | 189 | 3e-46 | |
| NEW | ref\|NP_001018606.1\| | ankyrin repeat domain 45 [Danio rerio] >g... | 188 | 4e-46 | UG |
| NEW | ref\|XP_002131508.1\| | PREDICTED: similar to mCG9408 [Ciona inte... | 154 | 6e-36 | G |
| NEW | ref\|XP_001639234.1\| | predicted protein [Nematostella vectensis... | 147 | 8e-34 | UG |
| NEW | ref\|XP_002609257.1\| | hypothetical protein BRAFLDRAFT_124756 [B... | 146 | 2e-33 | G |
| NEW | gb\|ACC54983.1\| | ankyrin repeat domain 45 [Xenopus borealis] | 144 | 1e-32 | |
| NEW | ref\|XP_783791.1\| | PREDICTED: similar to Ankrd45 protein [Stron... | 128 | 5e-28 | G |
| NEW | ref\|XP_002109388.1\| | hypothetical protein TRIADDRAFT_53367 [Tr... | 102 | 4e-20 | G |
| NEW | ref\|XP_001255172.1\| | PREDICTED: similar to solute carrier fami... | 92.4 | 5e-17 | UG |
| NEW | ref\|XP_002572929.1\| | Ankrd45 protein [Schistosoma mansoni] >em... | 67.8 | 1e-09 | G |
| NEW | ref\|XP_002423923.1\| | hspc200, putative [Pediculus humanus corp... | 62.8 | 3e-08 | G |
| NEW | ref\|XP_002585519.1\| | hypothetical protein BRAFLDRAFT_133193 [B... | 62.0 | 6e-08 | G |
| NEW | ref\|NP_999979.1\| | tumor protein p53 binding protein, 2 [Danio ... | 62.0 | 7e-08 | UG |
| NEW | ref\|XP_001918932.1\| | PREDICTED: hypothetical protein [Danio re... | 61.6 | 7e-08 | UG |
| NEW | gb\|ABA54552.1\| | 70 kDa heat shock protein [Mytilus edulis] | 61.6 | 7e-08 | |
| NEW | ref\|XP_001368866.1\| | PREDICTED: similar to hCG2042411 [Monodel... | 61.6 | 8e-08 | UG |
| NEW | ref\|XP_001513723.1\| | PREDICTED: similar to tumor protein p53 b... | 60.8 | 1e-07 | UG |
| NEW | ref\|XP_419394.2\| | PREDICTED: similar to tumor protein p53 bind... | 60.8 | 1e-07 | G |
| NEW | gb\|AAH98968.1\| | LOC414498 protein [Xenopus laevis] | 60.5 | 2e-07 | G |
| NEW | gb\|AAH68956.1\| | LOC414498 protein [Xenopus laevis] | 60.5 | 2e-07 | G |
| NEW | ref\|NP_001087084.1\| | hypothetical protein LOC446947 [Xenopus l... | 60.5 | 2e-07 | UG |
| NEW | gb\|AAI71141.1\| | LOC548790 protein [Xenopus tropicalis] | 60.5 | 2e-07 | G |
| NEW | ref\|XP_002591229.1\| | hypothetical protein BRAFLDRAFT_131415 [B... | 60.5 | 2e-07 | G |
| NEW | emb\|CAF98676.1\| | unnamed protein product [Tetraodon nigroviridis] | 60.5 | 2e-07 | |
| NEW | ref\|NP_001016036.2\| | hypothetical protein LOC548790 [Xenopus (... | 60.5 | 2e-07 | UG |
| NEW | ref\|XP_002196970.1\| | PREDICTED: tumor protein p53 binding prot... | 60.5 | 2e-07 | UG |
| NEW | ref\|XP_002407854.1\| | conserved hypothetical protein [Ixodes sc... | 60.5 | 2e-07 | G |
| NEW | dbj\|BAE90962.1\| | unnamed protein product [Macaca fascicularis] | 60.5 | 2e-07 | |
| NEW | ref\|XP_001897569.1\| | Ankyrin repeat containing protein [Brugia... | 59.7 | 3e-07 | G |
| NEW | gb\|AAI42600.1\| | Si:dkeyp-34c12.2 protein [Danio rerio] | 59.7 | 3e-07 | G |
| NEW | ref\|NP_001038618.1\| | tumor protein p53 binding protein, 2 [Dan... | 59.7 | 3e-07 | UG |
| NEW | ref\|NP_001103240.2\| | tumor protein p53 binding protein, 2-like... | 59.7 | 3e-07 | UG |
| NEW | ref\|XP_001923587.1\| | PREDICTED: hypothetical protein LOC793439... | 59.7 | 3e-07 | UG |
| NEW | dbj\|BAG58337.1\| | unnamed protein product [Homo sapiens] | 59.3 | 4e-07 | G |
| NEW | ref\|XP_863359.1\| | PREDICTED: similar to 78 kDa glucose-regulat... | 59.3 | 4e-07 | UG |
| NEW | gb\|AAH98984.1\| | LOC446947 protein [Xenopus laevis] | 59.3 | 4e-07 | G |
| NEW | gb\|AAI10930.1\| | LOC446947 protein [Xenopus laevis] | 59.3 | 4e-07 | G |
| NEW | gb\|AAH81262.1\| | LOC446947 protein [Xenopus laevis] | 59.3 | 4e-07 | G |
| NEW | dbj\|BAE91720.1\| | unnamed protein product [Macaca fascicularis] | 58.9 | 4e-07 | |
| NEW | ref\|XP_002192655.1\| | PREDICTED: heat shock 70kDa protein 5 [Ta... | 58.9 | 5e-07 | UG |
| NEW | dbj\|BAH14474.1\| | unnamed protein product [Homo sapiens] | 58.9 | 5e-07 | G |

| | | | | | |
|---|---|---|---|---|---|
| NEW | dbj\|BAG58037.1\| | unnamed protein product [Homo sapiens] >dbj\|B... | 58.9 | 5e-07 | G |
| NEW | dbj\|BAF38391.1\| | heat shock protein 70kDa [Coturnix japonica] | 58.9 | 5e-07 | |
| NEW | ref\|XP_001480035.1\| | PREDICTED: similar to hCG2042411 [Mus mus... | 58.9 | 5e-07 | UG |
| NEW | dbj\|BAD12571.1\| | heat shock protein [Numida meleagris] | 58.9 | 5e-07 | |
| NEW | dbj\|BAH11835.1\| | unnamed protein product [Homo sapiens] | 58.9 | 5e-07 | G |
| NEW | ref\|NP_990822.1\| | heat shock 70kDa protein 5 precursor [Gallus... | 58.9 | 5e-07 | UG |
| NEW | gb\|AAH30894.1\| | Trp53bp2 protein [Mus musculus] | 58.9 | 5e-07 | G |
| NEW | gb\|EDL13107.1\| | transformation related protein 53 binding prot... | 58.9 | 6e-07 | G |
| NEW | gb\|AAA21597.1\| | p53-binding protein | 58.9 | 6e-07 | G |
| NEW | gb\|EAW93247.1\| | tumor protein p53 binding protein, 2, isoform ... | 58.9 | 6e-07 | G |
| NEW | dbj\|BAE22185.1\| | unnamed protein product [Mus musculus] | 58.5 | 6e-07 | G |
| NEW | ref\|NP_001026855.2\| | tumor protein p53 binding protein, 2 isof... | 58.5 | 6e-07 | UG |
| NEW | ref\|NP_005417.1\| | tumor protein p53 binding protein, 2 isoform... | 58.5 | 6e-07 | UG |
| NEW | ref\|XP_001511765.1\| | PREDICTED: similar to hCG2042411 [Ornitho... | 58.5 | 6e-07 | UG |
| NEW | ref\|XP_576520.1\| | PREDICTED: similar to ankyrin repeat domain ... | 58.5 | 6e-07 | G |
| NEW | sp\|Q13625.2\|ASPP2_HUMAN | RecName: Full=Apoptosis-stimulating o... | 58.5 | 6e-07 | G |
| NEW | ref\|XP_223012.4\| | PREDICTED: similar to Apoptosis-stimulating ... | 58.5 | 6e-07 | UG |
| NEW | ref\|XP_001063503.1\| | PREDICTED: similar to Apoptosis-stimulati... | 58.5 | 7e-07 | UG |
| NEW | gb\|EDL94874.1\| | rCG20309 [Rattus norvegicus] | 58.5 | 7e-07 | |
| NEW | gb\|AAH40247.1\| | TP53BP2 protein [Homo sapiens] | 58.5 | 7e-07 | G |
| NEW | sp\|Q8CG79.3\|ASPP2_MOUSE | RecName: Full=Apoptosis-stimulating o... | 58.5 | 7e-07 | G |
| NEW | gb\|EAW93248.1\| | tumor protein p53 binding protein, 2, isoform ... | 58.5 | 7e-07 | G |
| NEW | ref\|NP_775554.2\| | tumor protein p53 binding protein, 2 [Mus mu... | 58.2 | 8e-07 | UG |
| NEW | ref\|XP_547518.2\| | PREDICTED: similar to Apoptosis stimulating ... | 58.2 | 9e-07 | UG |
| NEW | ref\|XP_001488127.2\| | PREDICTED: tumor protein p53 binding prot... | 58.2 | 9e-07 | UG |
| NEW | gb\|ABQ22601.1\| | 78 kDa glucose regulated protein precursor-lik... | 58.2 | 9e-07 | |
| NEW | ref\|XP_002433656.1\| | heat shock protein, putative [Ixodes scap... | 57.8 | 1e-06 | UG |
| NEW | ref\|XP_001376253.1\| | PREDICTED: similar to tumor protein p53 b... | 57.8 | 1e-06 | UG |
| NEW | ref\|XP_002578195.1\| | fetal globin-inducing factor [Schistosoma... | 57.4 | 1e-06 | UG |
| NEW | emb\|CAQ52955.1\| | CD4-specific ankyrin repeat protein D27.2 [sy... | 57.4 | 1e-06 | |
| NEW | ref\|XP_001926385.1\| | PREDICTED: ankyrin 1, erythrocytic, parti... | 57.4 | 1e-06 | UG |
| NEW | ref\|XP_001093747.1\| | PREDICTED: tumor protein p53 binding prot... | 57.4 | 1e-06 | UG |
| NEW | emb\|CAQ52952.1\| | CD4-specific ankyrin repeat protein D6.1 [syn... | 57.4 | 1e-06 | |
| NEW | emb\|CAQ52956.1\| | CD4-specific ankyrin repeat protein D29.2 [sy... | 57.4 | 2e-06 | |
| NEW | ref\|XP_002345952.1\| | PREDICTED: similar to ankyrin repeat doma... | 57.4 | 2e-06 | UG |
| NEW | ref\|NP_001155907.1\| | hypothetical protein LOC100287718 [Homo s... | 57.4 | 2e-06 | UG |
| NEW | ref\|XP_001917133.1\| | PREDICTED: similar to heat shock 70kDa pr... | 57.0 | 2e-06 | UG |
| NEW | pdb\|1YCS\|B | Chain B, P53-53bp2 Complex | 57.0 | 2e-06 | S |
| NEW | gb\|AAA37315.1\| | immunoglobulin heavy chain binding protein | 57.0 | 2e-06 | G |
| NEW | ref\|XP_641640.1\| | hypothetical protein [Dictyostelium discoide... | 56.6 | 2e-06 | G |
| NEW | dbj\|BAD90025.1\| | glucose-regulated protein 78kDa [Oncorhynchus... | 56.6 | 2e-06 | G |
| NEW | ref\|NP_001135114.1\| | 78 kDa glucose-regulated protein [Salmo s... | 56.6 | 2e-06 | UG |
| NEW | ref\|XP_001144115.1\| | PREDICTED: heat shock 70kDa protein 5 (gl... | 56.6 | 2e-06 | UG |
| NEW | gb\|EAW87621.1\| | heat shock 70kDa protein 5 (glucose-regulated ... | 56.6 | 3e-06 | G |
| NEW | ref\|XP_518520.2\| | PREDICTED: meprin A, alpha (PABA peptide hyd... | 56.6 | 3e-06 | UG |
| NEW | dbj\|BAE39187.1\| | unnamed protein product [Mus musculus] | 56.6 | 3e-06 | G |
| NEW | ref\|XP_392417.3\| | PREDICTED: similar to CG4393-PA [Apis mellif... | 56.6 | 3e-06 | UG |
| NEW | ref\|XP_974604.2\| | PREDICTED: similar to CG4393 CG4393-PA [Trib... | 56.2 | 3e-06 | UG |
| NEW | ref\|XP_001098999.1\| | PREDICTED: heat shock 70kDa protein 5 (gl... | 56.2 | 3e-06 | UG |
| NEW | gb\|AAA52614.1\| | GRP78 precursor >emb\|CAA61201.1\| BiP [Homo sap... | 56.2 | 3e-06 | G |
| NEW | ref\|NP_001068616.1\| | heat shock 70kDa protein 5 precursor [Bos... | 56.2 | 3e-06 | UG |
| NEW | ref\|NP_001126927.1\| | heat shock 70kDa protein 5 (glucose-regul... | 56.2 | 3e-06 | G |
| NEW | sp\|Q3S4T7.1\|GRP78_SPETR | RecName: Full=78 kDa glucose-regulate... | 56.2 | 3e-06 | |
| NEW | dbj\|BAB23387.1\| | unnamed protein product [Mus musculus] | 56.2 | 3e-06 | G |
| NEW | gb\|EDL08617.1\| | heat shock 70kD protein 5 (glucose-regulated p... | 56.2 | 3e-06 | G |
| NEW | ref\|XP_537847.2\| | PREDICTED: similar to 78 kDa glucose-regulat... | 56.2 | 3e-06 | UG |
| NEW | dbj\|BAE30705.1\| | unnamed protein product [Mus musculus] | 56.2 | 3e-06 | G |
| NEW | ref\|NP_005338.1\| | heat shock 70kDa protein 5 [Homo sapiens] >r... | 56.2 | 3e-06 | UG |
| NEW | ref\|ZP_01731588.1\| | hypothetical protein CY0110_09016 [Cyanoth... | 56.2 | 3e-06 | |
| NEW | ref\|XP_863385.1\| | PREDICTED: similar to 78 kDa glucose-regulat... | 56.2 | 3e-06 | UG |
| NEW | dbj\|BAE79724.1\| | immunoglobulin heavy-chain binding protein [M... | 56.2 | 3e-06 | |
| NEW | dbj\|BAA11462.1\| | 78 kDa glucose-regulated protein [Mus musculus] | 56.2 | 3e-06 | G |
| NEW | emb\|CAA05361.1\| | BiP [Mus musculus] | 56.2 | 3e-06 | G |
| NEW | gb\|AAF13605.1\|AF188611_1 | BiP protein [Homo sapiens] | 56.2 | 3e-06 | G |
| NEW | ref\|NP_071705.3\| | heat shock protein 5 precursor [Mus musculus... | 56.2 | 3e-06 | UG |

| | | | | | |
|---|---|---|---|---|---|
| NEW | dbj\|BAE35314.1\| | unnamed protein product [Mus musculus] | 56.2 | 3e-06 | G |
| NEW | ref\|XP_001927830.1\| | PREDICTED: heat shock 70kDa protein 5, pa... | 56.2 | 3e-06 | UG |
| NEW | sp\|P07823.1\|GRP78_MESAU | RecName: Full=78 kDa glucose-regulate... | 56.2 | 3e-06 | |
| NEW | gb\|ACT46911.1\| | heat shock 70kDa protein 5 isoform 2 [Cervus e... | 56.2 | 3e-06 | |
| NEW | gb\|EDL93171.1\| | heat shock 70kDa protein 5 (glucose-regulated ... | 56.2 | 4e-06 | G |
| NEW | gb\|ACJ65009.1\| | GRP78 [Ctenopharyngodon idella] | 56.2 | 4e-06 | |
| NEW | ref\|NP_037215.1\| | heat shock protein 5 precursor [Rattus norve... | 55.8 | 4e-06 | UG |
| NEW | ref\|ZP_02178274.1\| | ankyrin repeat domain protein [Hydrogenivi... | 55.8 | 4e-06 | |
| NEW | ref\|NP_001080064.1\| | heat shock 70kDa protein 5 (glucose-regul... | 55.8 | 4e-06 | UG |
| NEW | gb\|AAV66400.1\| | heat-shock 70-kDa protein 5 [Macaca fascicularis] | 55.8 | 4e-06 | |
| NEW | ref\|XP_782071.1\| | PREDICTED: hypothetical protein [Strongyloce... | 55.8 | 4e-06 | G |
| NEW | gb\|AAH77757.1\| | LOC397850 protein [Xenopus laevis] | 55.8 | 4e-06 | G |
| NEW | ref\|XP_001328606.1\| | ankyrin repeat protein [Trichomonas vagin... | 55.8 | 4e-06 | G |
| NEW | ref\|XP_785346.1\| | PREDICTED: hypothetical protein, partial [St... | 55.8 | 4e-06 | G |
| NEW | ref\|NP_001081462.1\| | heavy-chain binding protein BiP precursor... | 55.8 | 4e-06 | UG |
| NEW | ref\|YP_002730164.1\| | ankyrin domain protein [Persephonella mar... | 55.8 | 5e-06 | G |
| NEW | ref\|XP_002053831.1\| | GJ23129 [Drosophila virilis] >gb\|EDW67351... | 55.8 | 5e-06 | G |
| NEW | gb\|AAD10949.1\| | ankyrin repeat-containing protein 2 [Arabidops... | 55.5 | 5e-06 | |
| NEW | ref\|XP_001198426.1\| | PREDICTED: similar to ankyrin 2,3/unc44 [... | 55.5 | 5e-06 | G |
| NEW | ref\|XP_799947.2\| | PREDICTED: similar to multiple ankyrin repea... | 55.5 | 5e-06 | G |
| NEW | emb\|CAQ52948.1\| | CD4-specific ankyrin repeat protein D2.1 [syn... | 55.5 | 6e-06 | |
| NEW | ref\|YP_001958145.1\| | hypothetical protein Aasi_1071 [Candidatu... | 55.1 | 6e-06 | G |
| NEW | ref\|XP_001993641.1\| | GH20822 [Drosophila grimshawi] >gb\|EDV943... | 55.1 | 6e-06 | G |
| NEW | gb\|AAA37742.1\| | glucose-regulated protein 78 | 55.1 | 6e-06 | G |
| NEW | emb\|CAQ52951.1\| | CD4-specific ankyrin repeat protein D5.1 [syn... | 55.1 | 7e-06 | |
| NEW | ref\|XP_001602889.1\| | PREDICTED: similar to IP14385p [Nasonia v... | 55.1 | 7e-06 | UG |
| NEW | gb\|AAH93536.1\| | LOC733200 protein [Xenopus laevis] | 55.1 | 7e-06 | G |
| NEW | ref\|XP_692620.3\| | PREDICTED: similar to cask-interacting prote... | 55.1 | 7e-06 | UG |
| NEW | ref\|XP_001365714.1\| | PREDICTED: similar to glucose-regulated p... | 55.1 | 8e-06 | UG |
| NEW | gb\|ABK23212.1\| | unknown [Picea sitchensis] | 54.7 | 9e-06 | |
| NEW | ref\|XP_002001063.1\| | GI22180 [Drosophila mojavensis] >gb\|EDW16... | 54.7 | 9e-06 | G |
| NEW | ref\|YP_920685.1\| | ankyrin [Thermofilum pendens Hrk 5] >gb\|ABL7... | 54.7 | 1e-05 | G |
| NEW | ref\|XP_424401.2\| | PREDICTED: hypothetical protein [Gallus gallus] | 54.7 | 1e-05 | UG |
| NEW | ref\|XP_001095508.1\| | PREDICTED: similar to TRP (transient rece... | 54.7 | 1e-05 | UG |
| NEW | ref\|XP_001307066.1\| | ankyrin repeat protein [Trichomonas vagin... | 54.7 | 1e-05 | G |
| NEW | ref\|XP_001703461.1\| | hypothetical protein CHLREDRAFT_127770 [C... | 54.3 | 1e-05 | UG |
| NEW | ref\|XP_794552.2\| | PREDICTED: similar to ankyrin 2,3/unc44 [Str... | 54.3 | 1e-05 | UG |
| NEW | ref\|NP_998223.1\| | heat shock 70kDa protein 5 [Danio rerio] >gb... | 54.3 | 1e-05 | UG |
| NEW | ref\|NP_195270.1\| | AKR2 (ANKYRIN REPEAT-CONTAINING PROTEIN 2); ... | 54.3 | 1e-05 | UG |
| NEW | ref\|XP_001703462.1\| | hypothetical protein CHLREDRAFT_127770 [C... | 54.3 | 1e-05 | UG |
| NEW | gb\|AAH63946.1\| | Heat shock protein 5 [Danio rerio] >gb\|AAT6806... | 54.3 | 1e-05 | G |
| NEW | emb\|CAQ52949.1\| | CD4-specific ankyrin repeat protein D3.1 [syn... | 54.3 | 1e-05 | |
| NEW | pdb\|1SVX\|A | Chain A, Crystal Structure Of A Designed Selected ... | 54.3 | 1e-05 | S |
| NEW | ref\|XP_002137001.1\| | GA26825 [Drosophila pseudoobscura pseudoo... | 54.3 | 1e-05 | G |
| NEW | ref\|XP_313120.4\| | AGAP004215-PA [Anopheles gambiae str. PEST] ... | 54.3 | 1e-05 | UG |
| NEW | gb\|AAA28074.1\| | BiP, heat shock protein 3 | 53.9 | 1e-05 | |
| NEW | emb\|CAG12424.1\| | unnamed protein product [Tetraodon nigroviridis] | 53.9 | 2e-05 | |
| NEW | dbj\|BAE31621.1\| | unnamed protein product [Mus musculus] | 53.9 | 2e-05 | G |
| NEW | pdb\|2BKG\|A | Chain A, Crystal Structure Of E3_19 An Designed An... | 53.9 | 2e-05 | S |
| NEW | ref\|XP_794262.1\| | PREDICTED: similar to ankyrin 2,3/unc44, par... | 53.9 | 2e-05 | G |
| NEW | ref\|XP_001663294.1\| | hypothetical protein AaeL_AAEL013079 [Aed... | 53.9 | 2e-05 | UG |
| NEW | ref\|XP_001179198.1\| | PREDICTED: similar to ankyrin 2,3/unc44 [... | 53.9 | 2e-05 | G |
| NEW | ref\|XP_002188655.1\| | PREDICTED: ankyrin repeat domain 42 [Taen... | 53.9 | 2e-05 | UG |
| NEW | ref\|NP_001088961.1\| | hypothetical protein LOC496341 [Xenopus l... | 53.9 | 2e-05 | UG |
| NEW | ref\|NP_001087157.1\| | MGC83480 protein [Xenopus laevis] >gb\|AAH... | 53.9 | 2e-05 | UG |
| NEW | gb\|AAW25061.1\| | SJCHGC02512 protein [Schistosoma japonicum] | 53.9 | 2e-05 | |
| NEW | dbj\|BAD15288.1\| | 78kDa glucose regulated protein [Crassostrea ... | 53.9 | 2e-05 | |
| NEW | sp\|Q96KQ4.2\|ASPP1_HUMAN | RecName: Full=Apoptosis-stimulating o... | 53.5 | 2e-05 | G |
| NEW | ref\|NP_056131.2\| | apoptosis-stimulating protein of p53, 1 [Hom... | 53.5 | 2e-05 | UG |
| NEW | ref\|XP_001844143.1\| | ankyrin repeat domain-containing protein ... | 53.5 | 2e-05 | UG |
| NEW | ref\|XP_001374556.1\| | PREDICTED: similar to ankyrin repeat doma... | 53.5 | 2e-05 | UG |
| NEW | ref\|NP_001093487.1\| | ankyrin repeat and sterile alpha motif do... | 53.5 | 2e-05 | UG |
| NEW | emb\|CAI11564.1\| | novel protein similar to human and mouse CASK... | 53.5 | 2e-05 | G |
| NEW | ref\|XP_002109829.1\| | hypothetical protein TRIADDRAFT_63625 [Tr... | 53.5 | 2e-05 | G |
| NEW | ref\|NP_787123.1\| | ankyrin, isoform C [Drosophila melanogaster]... | 53.5 | 2e-05 | UG |

| | | | | | |
|---|---|---|---|---|---|
| NEW | ref\|XP_001607835.1\| | PREDICTED: similar to ENSANGP00000016511 ... | 53.5 | 2e-05 | G |
| NEW | ref\|XP_002054184.1\| | GJ24299 [Drosophila virilis] >gb\|EDW67704... | 53.5 | 2e-05 | G |
| NEW | ref\|NP_849499.1\| | AKR2 (ANKYRIN REPEAT-CONTAINING PROTEIN 2); ... | 53.5 | 2e-05 | UG |
| NEW | gb\|AAC37208.1\| | ankyrin [Drosophila melanogaster] >prf\|\|202234... | 53.5 | 2e-05 | E |
| NEW | ref\|XP_391790.1\| | hypothetical protein FG11614.1 [Gibberella z... | 53.5 | 2e-05 | G |
| NEW | ref\|XP_001319508.1\| | ankyrin repeat protein [Trichomonas vagin... | 53.5 | 2e-05 | G |
| NEW | gb\|AAA28076.1\| | heat shock protein 4 [Caenorhabditis elegans] | 53.5 | 2e-05 | |
| NEW | ref\|XP_001994442.1\| | GH16234 [Drosophila grimshawi] >gb\|EDV910... | 53.5 | 2e-05 | G |
| NEW | ref\|XP_001553065.1\| | hypothetical protein BC1G_08957 [Botryoti... | 53.5 | 2e-05 | G |
| NEW | ref\|XP_002020191.1\| | GL13852 [Drosophila persimilis] >gb\|EDW39... | 53.5 | 2e-05 | G |
| NEW | dbj\|BAF80467.1\| | HSP70 protein [Poecilia reticulata] | 53.1 | 2e-05 | |
| NEW | ref\|XP_001330150.1\| | ankyrin repeat protein [Trichomonas vagin... | 53.1 | 3e-05 | G |
| NEW | gb\|EDL99058.1\| | similar to hypothetical protein DKFZp434D2328 ... | 53.1 | 3e-05 | |
| NEW | dbj\|BAA34491.1\| | KIAA0771 protein [Homo sapiens] | 53.1 | 3e-05 | G |
| NEW | ref\|XP_001235479.1\| | PREDICTED: hypothetical protein [Gallus g... | 53.1 | 3e-05 | UG |
| NEW | ref\|XP_692689.2\| | PREDICTED: similar to cask-interacting prote... | 53.1 | 3e-05 | UG |
| NEW | ref\|NP_065208.2\| | ankyrin 1 isoform 4 [Homo sapiens] >gb\|EAW63... | 53.1 | 3e-05 | G |
| NEW | pir\|\|B35049 | ankyrin 1, erythrocyte splice form 3 - human | 53.1 | 3e-05 | |
| NEW | gb\|EDL00012.1\| | mCG117548 [Mus musculus] | 53.1 | 3e-05 | |
| NEW | ref\|XP_539957.2\| | PREDICTED: similar to ankyrin 1 isoform 3 [C... | 53.1 | 3e-05 | UG |
| NEW | ref\|XP_001139287.1\| | PREDICTED: ankyrin 1 isoform 1 [Pan trogl... | 53.1 | 3e-05 | UG |
| NEW | ref\|XP_001139606.1\| | PREDICTED: ankyrin 1 isoform 4 [Pan trogl... | 53.1 | 3e-05 | G |
| NEW | ref\|NP_065209.2\| | ankyrin 1 isoform 1 [Homo sapiens] >sp\|P1615... | 53.1 | 3e-05 | UG |
| NEW | ref\|XP_001099591.1\| | PREDICTED: similar to ankyrin 1 isoform 1... | 53.1 | 3e-05 | UG |
| NEW | ref\|NP_001074902.1\| | ankyrin repeat domain 44 [Mus musculus] | 53.1 | 3e-05 | UG |
| NEW | sp\|B2RXR6.1\|ANR44_MOUSE | RecName: Full=Serine/threonine-protei... | 53.1 | 3e-05 | G |
| NEW | ref\|NP_001135918.1\| | ankyrin 1 isoform 9 [Homo sapiens] | 53.1 | 3e-05 | UG |
| NEW | dbj\|BAD92655.1\| | ankyrin 1 isoform 4 variant [Homo sapiens] | 53.1 | 3e-05 | G |
| NEW | ref\|XP_001790685.1\| | PREDICTED: myosin XVI [Bos taurus] | 53.1 | 3e-05 | UG |
| NEW | emb\|CAQ51694.1\| | ankyrin repeat and SAM domain containing 1 [M... | 53.1 | 3e-05 | |
| NEW | ref\|XP_002007551.1\| | GI12323 [Drosophila mojavensis] >gb\|EDW18... | 52.8 | 3e-05 | G |
| NEW | ref\|XP_001327095.1\| | ankyrin repeat protein [Trichomonas vagin... | 52.8 | 3e-05 | G |
| NEW | ref\|NP_065210.2\| | ankyrin 1 isoform 2 [Homo sapiens] >gb\|EAW63... | 52.8 | 3e-05 | UG |
| NEW | prf\|\|1605244A | erythrocyte ankyrin | 52.8 | 3e-05 | |
| NEW | ref\|NP_000028.3\| | ankyrin 1 isoform 3 [Homo sapiens] >gb\|EAW63... | 52.8 | 3e-05 | G |
| NEW | emb\|CAA34611.1\| | alt. ankyrin (variant 2.2) [Homo sapiens] | 52.8 | 3e-05 | G |
| NEW | ref\|XP_882829.2\| | PREDICTED: similar to apoptosis-stimulating ... | 52.8 | 3e-05 | UG |
| NEW | gb\|AAA51732.1\| | ankyrin [Homo sapiens] | 52.8 | 3e-05 | G |
| NEW | gb\|AAI57919.1\| | Ankrd44 protein [Mus musculus] | 52.8 | 3e-05 | G |
| NEW | ref\|XP_001628783.1\| | predicted protein [Nematostella vectensis... | 52.8 | 3e-05 | G |
| NEW | ref\|XP_001328459.1\| | hypothetical protein [Trichomonas vaginal... | 52.8 | 3e-05 | G |
| NEW | ref\|XP_001139450.1\| | PREDICTED: ankyrin 1 isoform 3 [Pan trogl... | 52.8 | 3e-05 | UG |
| NEW | ref\|NP_001104253.1\| | ankyrin 1, erythroid isoform 1 [Mus muscu... | 52.8 | 3e-05 | UG |
| NEW | emb\|CAA34610.1\| | unnamed protein product [Homo sapiens] | 52.8 | 3e-05 | G |
| NEW | gb\|AAH79910.1\| | Ank1 protein [Mus musculus] | 52.8 | 3e-05 | G |
| NEW | gb\|ABG56392.1\| | glucose-regulated protein 78 [Paralichthys oli... | 52.8 | 3e-05 | |
| NEW | ref\|XP_002098422.1\| | GE23960 [Drosophila yakuba] >gb\|EDW98134.... | 52.8 | 3e-05 | G |
| NEW | ref\|XP_237153.4\| | PREDICTED: similar to ankyrin repeat domain ... | 52.8 | 3e-05 | UG |
| NEW | ref\|XP_001101829.1\| | PREDICTED: similar to UNCoordinated famil... | 52.8 | 3e-05 | UG |
| NEW | emb\|CAA48801.1\| | erythroid ankyrin [Mus musculus] | 52.8 | 3e-05 | G |
| NEW | gb\|EAW63241.1\| | ankyrin 1, erythrocytic, isoform CRA_a [Homo s... | 52.8 | 3e-05 | G |
| NEW | dbj\|BAE39999.1\| | unnamed protein product [Mus musculus] | 52.8 | 3e-05 | G |
| NEW | emb\|CAQ52953.1\| | CD4-specific ankyrin repeat protein D23.2 [sy... | 52.8 | 3e-05 | |
| NEW | ref\|XP_001319255.1\| | ankyrin repeat protein [Trichomonas vagin... | 52.8 | 3e-05 | G |
| NEW | sp\|Q02357.2\|ANK1_MOUSE | RecName: Full=Ankyrin-1; AltName: Full... | 52.8 | 3e-05 | G |
| NEW | gb\|AAA37236.1\| | ankyrin | 52.8 | 3e-05 | G |
| NEW | dbj\|BAE34375.1\| | unnamed protein product [Mus musculus] | 52.8 | 4e-05 | G |
| NEW | ref\|NP_112435.2\| | ankyrin 1, erythroid isoform 2 [Mus musculus... | 52.8 | 4e-05 | UG |
| NEW | gb\|EDL22556.1\| | ankyrin repeat and SAM domain containing 1, is... | 52.8 | 4e-05 | G |
| NEW | ref\|XP_001750549.1\| | hypothetical protein [Monosiga brevicolli... | 52.8 | 4e-05 | G |
| NEW | ref\|XP_001088146.1\| | PREDICTED: similar to ankyrin repeat doma... | 52.8 | 4e-05 | UG |
| NEW | dbj\|BAE28015.1\| | unnamed protein product [Mus musculus] | 52.8 | 4e-05 | G |
| NEW | dbj\|BAC97904.1\| | mKIAA0229 protein [Mus musculus] >gb\|EDL22557... | 52.8 | 4e-05 | G |
| NEW | gb\|AAI71944.1\| | Ank1 protein [Mus musculus] >gb\|AAI38030.1\| An... | 52.8 | 4e-05 | G |
| NEW | ref\|XP_001373148.1\| | PREDICTED: similar to protein phosphatase... | 52.8 | 4e-05 | UG |

| | | | | | |
|---|---|---|---|---|---|
| NEW | ref\|XP_001088469.1\| | PREDICTED: similar to ankyrin repeat doma... | 52.8 | 4e-05 | UG |
| NEW | gb\|EER15321.1\| | protein phosphatase 1 regulatory subunit 12B, ... | 52.8 | 4e-05 | |
| NEW | gb\|EAW70141.1\| | ankyrin repeat domain 44, isoform CRA_a [Homo ... | 52.8 | 4e-05 | G |
| NEW | gb\|AAH63622.1\| | ANKRD44 protein [Homo sapiens] | 52.4 | 4e-05 | G |
| NEW | gb\|AAX93155.1\| | unknown [Homo sapiens] | 52.4 | 4e-05 | G |
| NEW | gb\|AAH16985.2\| | ANKRD44 protein [Homo sapiens] | 52.4 | 4e-05 | G |
| NEW | ref\|NP_710181.1\| | ankyrin repeat domain 44 [Homo sapiens] >dbj... | 52.4 | 4e-05 | UG |
| NEW | sp\|Q8N8A2.3\|ANR44_HUMAN | RecName: Full=Serine/threonine-protei... | 52.4 | 4e-05 | G |
| NEW | pdb\|2J8S\|D | Chain D, Drug Export Pathway Of Multidrug Exporter... | 52.4 | 4e-05 | S |
| NEW | dbj\|BAH13433.1\| | unnamed protein product [Homo sapiens] | 52.4 | 4e-05 | G |
| NEW | ref\|NP_001101532.1\| | protein phosphatase 1, regulatory (inhibi... | 52.4 | 4e-05 | UG |
| NEW | gb\|AAH50586.2\| | ANKRD44 protein [Homo sapiens] | 52.4 | 4e-05 | G |
| NEW | ref\|XP_001492487.2\| | PREDICTED: protein phosphatase 1, regulat... | 52.4 | 4e-05 | UG |
| NEW | ref\|XP_001198404.1\| | PREDICTED: similar to ankyrin 2,3/unc44 [... | 52.4 | 5e-05 | UG |
| NEW | ref\|YP_002730162.1\| | pfs, nacht and ankyrin domain protein [Pe... | 52.4 | 5e-05 | G |
| NEW | ref\|XP_001948769.1\| | PREDICTED: similar to proteasome (prosome... | 52.4 | 5e-05 | UG |
| NEW | ref\|XP_001088357.1\| | PREDICTED: similar to ankyrin repeat doma... | 52.4 | 5e-05 | UG |
| NEW | ref\|XP_001181123.1\| | PREDICTED: similar to ankyrin 2,3/unc44 [... | 52.4 | 5e-05 | G |
| NEW | ref\|XP_002156272.1\| | PREDICTED: similar to predicted protein [... | 52.4 | 5e-05 | UG |
| NEW | ref\|XP_002124556.1\| | PREDICTED: similar to Ankyrin repeat doma... | 52.4 | 5e-05 | UG |
| NEW | dbj\|BAC29971.1\| | unnamed protein product [Mus musculus] | 52.4 | 5e-05 | G |
| NEW | ref\|XP_001088783.1\| | PREDICTED: similar to protein phosphatase... | 52.4 | 5e-05 | UG |
| NEW | ref\|XP_001087907.1\| | PREDICTED: similar to ankyrin repeat doma... | 52.4 | 5e-05 | UG |
| NEW | ref\|XP_516003.2\| | PREDICTED: ankyrin repeat domain 44 [Pan tro... | 52.4 | 5e-05 | UG |
| NEW | emb\|CAQ15730.1\| | novel protein (zgc:136667) [Danio rerio] | 52.4 | 5e-05 | G |
| NEW | gb\|EDL18608.1\| | protein phosphatase 1, regulatory (inhibitor) ... | 52.4 | 5e-05 | G |
| NEW | ref\|XP_001179137.1\| | PREDICTED: similar to ankyrin 2,3/unc44 [... | 52.4 | 5e-05 | G |
| NEW | ref\|XP_002043032.1\| | GM16283 [Drosophila sechellia] >gb\|EDW491... | 52.0 | 5e-05 | G |
| NEW | ref\|XP_002105237.1\| | GD18026 [Drosophila simulans] >gb\|EDX1474... | 52.0 | 6e-05 | G |
| NEW | gb\|AAL39916.1\| | SD01389p [Drosophila melanogaster] | 52.0 | 6e-05 | |
| NEW | ref\|XP_547996.2\| | PREDICTED: similar to protein phosphatase 1,... | 52.0 | 6e-05 | UG |
| NEW | ref\|NP_651624.2\| | CG10011 [Drosophila melanogaster] >gb\|AAF568... | 52.0 | 6e-05 | UG |
| NEW | ref\|XP_002070509.1\| | GK10993 [Drosophila willistoni] >gb\|EDW81... | 52.0 | 6e-05 | G |
| NEW | ref\|XP_002098692.1\| | GE10505 [Drosophila yakuba] >gb\|EDW98404.... | 52.0 | 6e-05 | G |
| NEW | ref\|XP_001981449.1\| | GG12063 [Drosophila erecta] >gb\|EDV53319.... | 52.0 | 6e-05 | G |
| NEW | ref\|NP_651143.1\| | CG4393 [Drosophila melanogaster] >gb\|AAF5613... | 52.0 | 6e-05 | UG |
| NEW | gb\|ABF85746.1\| | IP14385p [Drosophila melanogaster] | 52.0 | 6e-05 | |
| NEW | gb\|AAF61702.1\|AF222766_1 | ankyrin 1 [Bos taurus] | 52.0 | 6e-05 | G |
| NEW | ref\|XP_001892998.1\| | Heat shock 70 kDa protein C precursor [Br... | 52.0 | 6e-05 | G |
| NEW | ref\|NP_035755.1\| | apoptosis-stimulating protein of p53, 1 [Mus... | 52.0 | 6e-05 | UG |
| NEW | ref\|NP_001093346.1\| | hypothetical protein LOC100101286 [Xenopu... | 52.0 | 6e-05 | UG |
| NEW | dbj\|BAH28858.1\| | glucose regulated stress protein [Babesia mic... | 52.0 | 6e-05 | |
| NEW | ref\|XP_001955059.1\| | GF16435 [Drosophila ananassae] >gb\|EDV436... | 52.0 | 6e-05 | G |
| NEW | pdb\|2QYJ\|A | Chain A, Crystal Structure Of A Designed Full Cons... | 52.0 | 6e-05 | S |
| NEW | ref\|XP_001303322.1\| | uncoordinated [Trichomonas vaginalis G3] ... | 52.0 | 6e-05 | G |
| NEW | ref\|XP_785784.2\| | PREDICTED: similar to ankyrin 2,3/unc44 [Str... | 52.0 | 7e-05 | UG |
| NEW | gb\|AAL65911.1\|AF425651_1 | multiple ankyrin repeat single KH do... | 52.0 | 7e-05 | |
| NEW | ref\|NP_788733.1\| | multiple ankyrin repeats single KH domain, i... | 52.0 | 7e-05 | UG |
| NEW | ref\|XP_002604962.1\| | hypothetical protein BRAFLDRAFT_92602 [Br... | 52.0 | 7e-05 | G |
| NEW | ref\|XP_002104551.1\| | GD18385 [Drosophila simulans] >gb\|EDX1405... | 52.0 | 7e-05 | G |
| NEW | ref\|XP_001955683.1\| | GF16118 [Drosophila ananassae] >gb\|EDV442... | 52.0 | 7e-05 | G |
| NEW | ref\|XP_001982146.1\| | GG12437 [Drosophila erecta] >gb\|EDV54016.... | 52.0 | 7e-05 | G |
| NEW | ref\|XP_002069787.1\| | GK11389 [Drosophila willistoni] >gb\|EDW80... | 51.6 | 7e-05 | G |
| NEW | gb\|AAR82779.1\| | LD31436p [Drosophila melanogaster] | 51.6 | 7e-05 | |
| NEW | ref\|XP_001310920.1\| | hypothetical protein [Trichomonas vaginal... | 51.6 | 7e-05 | G |
| NEW | pdb\|1N0R\|A | Chain A, 4ank: A Designed Ankyrin Repeat Protein W... | 51.6 | 7e-05 | S |
| NEW | ref\|YP_001958455.1\| | hypothetical protein Aasi_1435 [Candidatu... | 51.6 | 7e-05 | G |
| NEW | gb\|AAH53732.1\| | Ppp1r13b protein [Mus musculus] | 51.6 | 7e-05 | G |
| NEW | ref\|XP_643155.1\| | heat shock protein Hsp70 family protein [Dic... | 51.6 | 7e-05 | UG |
| NEW | ref\|XP_421392.2\| | PREDICTED: similar to ASPP1 protein [Gallus ... | 51.6 | 7e-05 | UG |
| NEW | ref\|XP_002126516.1\| | PREDICTED: similar to ankyrin 2 [Ciona in... | 51.6 | 7e-05 | UG |
| NEW | ref\|XP_001357873.2\| | GA10007 [Drosophila pseudoobscura pseudoo... | 51.6 | 7e-05 | G |
| NEW | ref\|XP_002099266.1\| | GE23462 [Drosophila yakuba] >gb\|EDW98978.... | 51.6 | 7e-05 | G |
| NEW | ref\|XP_001311114.1\| | ankyrin repeat protein [Trichomonas vagin... | 51.6 | 7e-05 | G |
| NEW | ref\|XP_002055873.1\| | GJ10528 [Drosophila virilis] >gb\|EDW58985... | 51.6 | 7e-05 | G |

| | | | | | |
|---|---|---|---|---|---|
| NEW | ref\|XP_001204405.1\| | PREDICTED: similar to ankyrin 2,3/unc44 [... | 51.6 | 7e-05 | G |
| NEW | gb\|AAL89945.1\| | SD03956p [Drosophila melanogaster] | 51.6 | 7e-05 | |
| NEW | gb\|AAC33264.1\| | AFT protein [Arabidopsis thaliana] | 51.6 | 7e-05 | |
| NEW | gb\|AAB18178.1\| | heat shock protein 70 [Botryllus schlosseri] | 51.6 | 8e-05 | |
| NEW | ref\|XP_776877.1\| | hypothetical protein CNBC3680 [Cryptococcus ... | 51.6 | 8e-05 | G |
| NEW | emb\|CAG13205.1\| | unnamed protein product [Tetraodon nigroviridis] | 51.6 | 8e-05 | |
| NEW | ref\|XP_002020074.1\| | GL13699 [Drosophila persimilis] >gb\|EDW38... | 51.6 | 8e-05 | G |
| NEW | ref\|XP_001375721.1\| | PREDICTED: similar to KIAA0865 protein [M... | 51.6 | 8e-05 | UG |
| NEW | ref\|XP_001982019.1\| | GG11270 [Drosophila erecta] >gb\|EDV53889.... | 51.6 | 8e-05 | G |
| NEW | ref\|XP_001325333.1\| | ankyrin repeat protein [Trichomonas vagin... | 51.6 | 8e-05 | G |
| NEW | ref\|XP_002047131.1\| | GJ13261 [Drosophila virilis] >gb\|EDW69473... | 51.6 | 8e-05 | G |
| NEW | ref\|NP_001101083.1\| | ankyrin repeat and sterile alpha motif do... | 51.6 | 8e-05 | UG |
| NEW | ref\|XP_002193885.1\| | PREDICTED: ankyrin 2, neuronal [Taeniopyg... | 51.6 | 8e-05 | UG |
| NEW | ref\|XP_001579668.1\| | hypothetical protein [Trichomonas vaginal... | 51.6 | 8e-05 | G |
| NEW | ref\|NP_509019.1\| | Heat Shock Protein family member (hsp-3) [Ca... | 51.6 | 8e-05 | UG |
| NEW | ref\|XP_002200620.1\| | PREDICTED: protein phosphatase 1, regulat... | 51.6 | 9e-05 | UG |
| NEW | ref\|XP_418023.2\| | PREDICTED: similar to KIAA0229 [Gallus gallus] | 51.6 | 9e-05 | UG |
| NEW | ref\|XP_002339917.1\| | ankyrin, putative [Talaromyces stipitatus... | 51.6 | 9e-05 | G |
| NEW | ref\|XP_001955441.1\| | GF18767 [Drosophila ananassae] >gb\|EDV440... | 51.6 | 9e-05 | G |
| NEW | ref\|XP_002108424.1\| | hypothetical protein TRIADDRAFT_51290 [Tr... | 51.6 | 9e-05 | G |
| NEW | ref\|ZP_04048557.1\| | ankyrin repeat-containing protein [Brachys... | 51.6 | 9e-05 | |
| NEW | ref\|XP_001313709.1\| | ankyrin repeat protein [Trichomonas vagin... | 51.2 | 1e-04 | G |
| NEW | gb\|AAX14417.1\| | ankyrin repeat domain protein [Wolbachia endos... | 51.2 | 1e-04 | |
| NEW | ref\|XP_510191.2\| | PREDICTED: protein phosphatase 1, regulatory... | 51.2 | 1e-04 | UG |
| NEW | ref\|XP_001312437.1\| | ankyrin repeat protein [Trichomonas vagin... | 51.2 | 1e-04 | G |
| NEW | ref\|NP_001070395.1\| | ankyrin repeat and SOCS box-containing 3 ... | 51.2 | 1e-04 | UG |
| NEW | ref\|NP_495536.1\| | Heat Shock Protein family member (hsp-4) [Ca... | 51.2 | 1e-04 | UG |
| NEW | ref\|ZP_00373097.1\| | ankyrin repeat domain protein [Wolbachia e... | 51.2 | 1e-04 | |
| NEW | ref\|XP_001326506.1\| | hypothetical protein [Trichomonas vaginal... | 51.2 | 1e-04 | G |
| NEW | ref\|XP_001916958.1\| | PREDICTED: similar to myosin heavy chain ... | 51.2 | 1e-04 | UG |
| NEW | ref\|XP_001750370.1\| | hypothetical protein [Monosiga brevicolli... | 51.2 | 1e-04 | G |
| NEW | ref\|XP_001323837.1\| | ankyrin repeat protein [Trichomonas vagin... | 51.2 | 1e-04 | G |
| NEW | ref\|XP_001489679.2\| | PREDICTED: ankyrin 1, erythrocytic [Equus... | 51.2 | 1e-04 | UG |
| NEW | gb\|AAB47805.1\| | ankyrin [Homo sapiens] | 51.2 | 1e-04 | G |
| NEW | ref\|NP_179331.1\| | AKR2B (ANKYRIN REPEAT-CONTAINING 2B); protei... | 50.8 | 1e-04 | UG |
| NEW | ref\|XP_001653127.1\| | ion channel nompc [Aedes aegypti] >gb\|EAT... | 50.8 | 1e-04 | UG |
| NEW | ref\|XP_001510173.1\| | PREDICTED: similar to ankyrin 1, erythroc... | 50.8 | 1e-04 | UG |
| NEW | ref\|XP_001329921.1\| | hypothetical protein [Trichomonas vaginal... | 50.8 | 1e-04 | G |
| NEW | gb\|AAA92961.1\| | PP-1M | 50.8 | 1e-04 | G |
| NEW | ref\|XP_002032474.1\| | GM26576 [Drosophila sechellia] >gb\|EDW434... | 50.8 | 1e-04 | G |
| NEW | ref\|XP_001625396.1\| | predicted protein [Nematostella vectensis... | 50.8 | 1e-04 | UG |
| NEW | gb\|EEH19511.1\| | hsp70-like protein C [Paracoccidioides brasili... | 50.8 | 1e-04 | |
| NEW | ref\|XP_001917009.1\| | PREDICTED: similar to ankyrin repeat doma... | 50.8 | 1e-04 | UG |
| NEW | emb\|CAH69075.1\| | novel protein similar to vertebrate protein p... | 50.8 | 1e-04 | G |
| NEW | sp\|Q6DRG7.2\|MYPT1_DANRE | RecName: Full=Protein phosphatase 1 r... | 50.8 | 1e-04 | G |
| NEW | ref\|XP_397133.3\| | PREDICTED: similar to fem-1 homolog b, parti... | 50.8 | 1e-04 | UG |
| NEW | ref\|NP_001003870.1\| | protein phosphatase 1, regulatory subunit... | 50.8 | 1e-04 | UG |
| NEW | emb\|CAF90261.1\| | unnamed protein product [Tetraodon nigroviridis] | 50.8 | 1e-04 | |
| NEW | gb\|EDM16759.1\| | protein phosphatase 1, regulatory (inhibitor) ... | 50.8 | 1e-04 | G |
| NEW | gb\|EAW54942.1\| | hCG40985, isoform CRA_b [Homo sapiens] | 50.8 | 1e-04 | |
| NEW | emb\|CAM46983.1\| | transient receptor potential cation channel, ... | 50.8 | 1e-04 | G |
| NEW | gb\|AAK85149.2\| | heat shock protein 70 [Trichinella spiralis] | 50.8 | 1e-04 | |
| NEW | ref\|XP_001514343.1\| | PREDICTED: similar to OTTHUMP00000018693 ... | 50.8 | 1e-04 | UG |
| NEW | ref\|XP_002560948.1\| | Pc16g06120 [Penicillium chrysogenum Wisco... | 50.8 | 1e-04 | G |
| NEW | gb\|ACI26674.1\| | transient receptor potential cation channel su... | 50.8 | 1e-04 | G |
| NEW | gb\|EDL21701.1\| | mCG122391, isoform CRA_c [Mus musculus] | 50.8 | 1e-04 | |
| NEW | emb\|CAQ52950.1\| | CD4-specific ankyrin repeat protein D4.1 [syn... | 50.8 | 2e-04 | |
| NEW | ref\|NP_001007066.1\| | transient receptor potential cation chann... | 50.8 | 2e-04 | UG |
| NEW | ref\|XP_002032342.1\| | GM23569 [Drosophila sechellia] >gb\|EDW433... | 50.8 | 2e-04 | G |
| NEW | gb\|AAH98558.1\| | Ppp1r12a protein [Danio rerio] | 50.8 | 2e-04 | G |
| NEW | ref\|NP_965867.1\| | ankyrin repeat-containing protein [Wolbachia... | 50.4 | 2e-04 | G |
| NEW | ref\|XP_002192823.1\| | PREDICTED: similar to ankyrin repeat doma... | 50.4 | 2e-04 | UG |
| NEW | dbj\|BAD89540.1\| | heat shock protein 70 [Pocillopora damicornis] | 50.4 | 2e-04 | |
| NEW | gb\|AAQ93811.1\| | ankyrin repeat protein mbp3_5 [synthetic const... | 50.4 | 2e-04 | |
| NEW | ref\|XP_001494364.2\| | PREDICTED: similar to ankyrin repeat and ... | 50.4 | 2e-04 | UG |
| NEW | ref\|XP_001900823.1\| | Protein phosphatase 1 regulatory subunit ... | 50.4 | 2e-04 | G |

| | | | | | |
|---|---|---|---|---|---|
| NEW | gb\|EDM16760.1\| | protein phosphatase 1, regulatory (inhibitor) ... | 50.4 | 2e-04 | G |
| NEW | ref\|XP_001321366.1\| | hypothetical protein [Trichomonas vaginal... | 50.4 | 2e-04 | G |
| NEW | sp\|Q07E28.1\|CTTB2_NEONE | RecName: Full=Cortactin-binding prote... | 50.4 | 2e-04 | |
| NEW | gb\|EAX03804.1\| | ankyrin repeat and sterile alpha motif domain ... | 50.4 | 2e-04 | G |
| NEW | ref\|NP_001012933.1\| | ankyrin repeat domain 44 [Gallus gallus] ... | 50.4 | 2e-04 | UG |
| NEW | ref\|NP_082168.1\| | protein phosphatase 1, regulatory (inhibitor... | 50.4 | 2e-04 | UG |
| NEW | gb\|EDM16761.1\| | protein phosphatase 1, regulatory (inhibitor) ... | 50.4 | 2e-04 | G |
| NEW | ref\|NP_001153255.1\| | ankyrin repeat domain 42 [Pongo abelii] >... | 50.4 | 2e-04 | G |
| NEW | ref\|NP_056060.2\| | ankyrin repeat and sterile alpha motif domai... | 50.4 | 2e-04 | UG |
| NEW | gb\|EDM16763.1\| | protein phosphatase 1, regulatory (inhibitor) ... | 50.4 | 2e-04 | G |
| NEW | gb\|EAX03803.1\| | ankyrin repeat and sterile alpha motif domain ... | 50.4 | 2e-04 | G |
| NEW | sp\|Q10728.2\|MYPT1_RAT | RecName: Full=Protein phosphatase 1 reg... | 50.4 | 2e-04 | G |
| NEW | ref\|XP_611767.4\| | PREDICTED: similar to ankyrin repeat and ste... | 50.4 | 2e-04 | UG |
| NEW | gb\|EAX03800.1\| | ankyrin repeat and sterile alpha motif domain ... | 50.4 | 2e-04 | G |
| NEW | ref\|XP_863806.1\| | PREDICTED: similar to ankyrin repeat and ste... | 50.4 | 2e-04 | UG |
| NEW | dbj\|BAA81720.2\| | protein tyrosine kinase [Ephydatia fluviatilis] | 50.4 | 2e-04 | |
| NEW | gb\|EDM16762.1\| | protein phosphatase 1, regulatory (inhibitor) ... | 50.4 | 2e-04 | G |
| NEW | gb\|EDL21702.1\| | mCG122391, isoform CRA_d [Mus musculus] | 50.4 | 2e-04 | |
| NEW | ref\|XP_001989663.1\| | GH18670 [Drosophila grimshawi] >gb\|EDV927... | 50.4 | 2e-04 | G |
| NEW | sp\|P12794.1\|GRP78_PLAFA | RecName: Full=78 kDa glucose-regulate... | 50.4 | 2e-04 | |
| NEW | gb\|EDL21699.1\| | mCG122391, isoform CRA_a [Mus musculus] | 50.4 | 2e-04 | |
| NEW | ref\|XP_536014.2\| | PREDICTED: similar to ankyrin repeat domain ... | 50.4 | 2e-04 | UG |
| NEW | ref\|XP_001325696.1\| | ankyrin repeat protein [Trichomonas vagin... | 50.4 | 2e-04 | G |
| NEW | gb\|AAI25382.1\| | Ppp1r12a protein [Mus musculus] >gb\|EDL21703.1... | 50.4 | 2e-04 | G |
| NEW | ref\|XP_973611.2\| | PREDICTED: similar to conserved hypothetical... | 50.4 | 2e-04 | UG |
| NEW | ref\|XP_001583286.1\| | hypothetical protein [Trichomonas vaginal... | 50.4 | 2e-04 | G |
| NEW | ref\|XP_001982753.1\| | GG16463 [Drosophila erecta] >gb\|EDV45272.... | 50.4 | 2e-04 | G |
| NEW | ref\|XP_692033.3\| | PREDICTED: similar to novel apoptosis-stimul... | 50.4 | 2e-04 | UG |
| NEW | ref\|XP_001579684.1\| | ankyrin repeat protein [Trichomonas vagin... | 50.4 | 2e-04 | G |
| NEW | ref\|XP_001302398.1\| | ankyrin repeat protein [Trichomonas vagin... | 50.4 | 2e-04 | G |
| NEW | ref\|XP_782722.2\| | PREDICTED: hypothetical protein, partial [St... | 50.4 | 2e-04 | UG |
| NEW | ref\|XP_001325758.1\| | ankyrin repeat protein [Trichomonas vagin... | 50.4 | 2e-04 | G |
| NEW | ref\|NP_446342.1\| | protein phosphatase 1, regulatory (inhibitor... | 50.4 | 2e-04 | UG |
| NEW | dbj\|BAC11532.1\| | unnamed protein product [Homo sapiens] | 50.4 | 2e-04 | G |
| NEW | ref\|XP_001226325.1\| | hypothetical protein CHGG_08398 [Chaetomi... | 50.4 | 2e-04 | G |
| NEW | ref\|XP_001324629.1\| | ankyrin repeat protein [Trichomonas vagin... | 50.4 | 2e-04 | G |
| NEW | ref\|XP_001582849.1\| | ankyrin repeat protein [Trichomonas vagin... | 50.4 | 2e-04 | G |
| NEW | emb\|CAR63694.1\| | hypothetical protein [Angiostrongylus cantone... | 50.4 | 2e-04 | |
| NEW | dbj\|BAA13218.1\| | KIAA0229 [Homo sapiens] | 50.4 | 2e-04 | G |
| NEW | ref\|XP_001582824.1\| | ankyrin repeat protein [Trichomonas vagin... | 50.4 | 2e-04 | G |
| NEW | sp\|A0M8T5.1\|CTTB2_FELCA | RecName: Full=Cortactin-binding prote... | 50.4 | 2e-04 | G |
| NEW | ref\|XP_002104690.1\| | GD21080 [Drosophila simulans] >gb\|EDX1419... | 50.1 | 2e-04 | G |
| NEW | gb\|AAO25692.1\| | ankyrin repeat protein E4_8 [synthetic construct] | 50.1 | 2e-04 | |
| NEW | ref\|XP_001925002.1\| | PREDICTED: similar to Myosin-XVI (Unconve... | 50.1 | 2e-04 | UG |
| NEW | ref\|ZP_04048424.1\| | ankyrin repeat-containing protein [Brachys... | 50.1 | 2e-04 | |
| NEW | ref\|NP_001137358.1\| | protein phosphatase 1, regulatory (inhibi... | 50.1 | 2e-04 | UG |
| NEW | ref\|XP_001381112.1\| | PREDICTED: similar to ankyrin repeat doma... | 50.1 | 2e-04 | UG |
| NEW | dbj\|BAH22317.1\| | ankyrin motif protein [Wolbachia endosymbiont... | 50.1 | 2e-04 | |
| NEW | ref\|XP_001111692.1\| | PREDICTED: similar to ankyrin repeat and ... | 50.1 | 2e-04 | UG |
| NEW | sp\|Q3KP44.2\|ANR55_HUMAN | RecName: Full=Ankyrin repeat domain-c... | 50.1 | 2e-04 | G |
| NEW | gb\|AAQ93812.1\| | ankyrin repeat protein mbp3_16 [synthetic cons... | 50.1 | 2e-04 | |
| NEW | ref\|XP_518420.2\| | PREDICTED: ankyrin repeat and sterile alpha ... | 50.1 | 2e-04 | UG |
| NEW | gb\|AAY54247.1\| | ankyrin domain protein [Wolbachia pipientis] | 50.1 | 2e-04 | |
| NEW | ref\|XP_001584342.1\| | hypothetical protein [Trichomonas vaginal... | 50.1 | 2e-04 | G |
| NEW | ref\|XP_002124800.1\| | PREDICTED: similar to protein phosphatase... | 50.1 | 2e-04 | UG |
| NEW | ref\|XP_001201315.1\| | PREDICTED: similar to ankyrin 2,3/unc44, ... | 50.1 | 2e-04 | G |
| NEW | dbj\|BAG63921.1\| | unnamed protein product [Homo sapiens] | 50.1 | 2e-04 | G |
| NEW | ref\|YP_002840971.1\| | Ankyrin [Sulfolobus islandicus Y.N.15.51]... | 50.1 | 2e-04 | G |
| NEW | ref\|XP_001181547.1\| | PREDICTED: similar to ankyrin 2,3/unc44, ... | 50.1 | 2e-04 | G |
| NEW | ref\|NP_078945.2\| | ankyrin repeat domain 55 isoform 1 [Homo sap... | 50.1 | 3e-04 | UG |
| NEW | ref\|YP_001957821.1\| | hypothetical protein Aasi_0703 [Candidatu... | 50.1 | 3e-04 | G |
| NEW | ref\|XP_002189785.1\| | PREDICTED: hypothetical protein [Taeniopy... | 50.1 | 3e-04 | UG |
| NEW | ref\|XP_002194705.1\| | PREDICTED: protein phosphatase 1, regulat... | 50.1 | 3e-04 | UG |
| NEW | ref\|XP_001379318.1\| | PREDICTED: similar to ankyrin repeat doma... | 50.1 | 3e-04 | UG |
| NEW | ref\|NP_001072865.1\| | ankyrin repeat domain 55 [Xenopus (Silura... | 50.1 | 3e-04 | UG |

| | | | | | |
|---|---|---|---|---|---|
| NEW | gb\|AAO25688.1\| | ankyrin repeat protein E2_17 [synthetic constr... | 50.1 | 3e-04 | |
| NEW | gb\|AAA29501.1\| | BiP | 50.1 | 3e-04 | |
| NEW | gb\|EEH44461.1\| | conserved hypothetical protein [Paracoccidioid... | 49.7 | 3e-04 | |
| NEW | ref\|YP_001957322.1\| | Ankyrin [Candidatus Amoebophilus asiaticu... | 49.7 | 3e-04 | G |
| NEW | ref\|XP_001493575.1\| | PREDICTED: similar to several ankyrin rep... | 49.7 | 3e-04 | UG |
| NEW | ref\|XP_001926579.1\| | PREDICTED: similar to ankyrin repeat and ... | 49.7 | 3e-04 | UG |
| NEW | ref\|XP_001301749.1\| | ankyrin repeat protein [Trichomonas vagin... | 49.7 | 3e-04 | G |
| NEW | ref\|XP_001201356.1\| | PREDICTED: similar to ankyrin 2,3/unc44, ... | 49.7 | 3e-04 | G |
| NEW | ref\|XP_001252778.1\| | PREDICTED: similar to hCG2042411 [Bos tau... | 49.7 | 3e-04 | UG |
| NEW | emb\|CAP31983.1\| | C. briggsae CBR-HSP-4 protein [Caenorhabditis... | 49.7 | 3e-04 | |
| NEW | gb\|AAI01274.1\| | ANKDD1A protein [Homo sapiens] | 49.7 | 3e-04 | G |
| NEW | ref\|XP_002466088.1\| | hypothetical protein SORBIDRAFT_01g000990... | 49.7 | 3e-04 | UG |
| NEW | ref\|NP_001140813.1\| | hypothetical protein LOC100272888 [Zea ma... | 49.7 | 3e-04 | UG |
| NEW | ref\|YP_001497220.1\| | hypothetical protein NY2A_B024L [Parameci... | 49.7 | 3e-04 | G |
| NEW | ref\|XP_001342336.2\| | PREDICTED: similar to cask-interacting pr... | 49.7 | 3e-04 | UG |
| NEW | ref\|XP_001527821.1\| | conserved hypothetical protein [Lodderomy... | 49.7 | 3e-04 | G |
| NEW | ref\|XP_002186575.1\| | PREDICTED: fem-1 homolog b [Taeniopygia g... | 49.7 | 3e-04 | UG |
| NEW | ref\|XP_542665.2\| | PREDICTED: similar to myosin heavy chain Myr... | 49.7 | 3e-04 | UG |
| NEW | ref\|XP_001302312.1\| | hypothetical protein [Trichomonas vaginal... | 49.7 | 3e-04 | G |
| NEW | ref\|NP_001128485.1\| | ankyrin repeat domain 42 [Rattus norvegic... | 49.7 | 3e-04 | UG |
| NEW | dbj\|BAC28945.1\| | unnamed protein product [Mus musculus] | 49.7 | 3e-04 | G |
| NEW | ref\|XP_785836.2\| | PREDICTED: similar to ankyrin 2,3/unc44 [Str... | 49.7 | 3e-04 | UG |
| NEW | ref\|XP_002197988.1\| | PREDICTED: similar to ankyrin repeat and ... | 49.7 | 3e-04 | UG |
| NEW | gb\|AAA93010.1\| | PBGRP | 49.7 | 3e-04 | |
| NEW | ref\|XP_544723.2\| | PREDICTED: similar to ankyrin 3, epithelial ... | 49.7 | 3e-04 | UG |
| NEW | ref\|XP_001868363.1\| | conserved hypothetical protein [Culex qui... | 49.7 | 3e-04 | UG |
| NEW | ref\|NP_001086927.1\| | CASK interacting protein 2 [Xenopus laevi... | 49.7 | 3e-04 | UG |
| NEW | emb\|CAA45762.1\| | BiP [Schizosaccharomyces pombe] | 49.7 | 3e-04 | |
| NEW | ref\|XP_001329422.1\| | ankyrin repeat protein [Trichomonas vagin... | 49.7 | 3e-04 | G |
| NEW | ref\|XP_001328234.1\| | hypothetical protein [Trichomonas vaginal... | 49.7 | 3e-04 | G |
| NEW | gb\|EAW54944.1\| | hCG40985, isoform CRA_d [Homo sapiens] | 49.7 | 3e-04 | |
| NEW | ref\|XP_517755.2\| | PREDICTED: hypothetical protein [Pan troglod... | 49.7 | 3e-04 | UG |
| NEW | dbj\|BAE00682.1\| | unnamed protein product [Macaca fascicularis] | 49.7 | 3e-04 | |
| NEW | ref\|YP_001957949.1\| | hypothetical protein Aasi_0849 [Candidatu... | 49.7 | 3e-04 | G |
| NEW | ref\|XP_001329312.1\| | ankyrin repeat protein [Trichomonas vagin... | 49.7 | 3e-04 | G |
| NEW | ref\|XP_001321688.1\| | ankyrin repeat protein [Trichomonas vagin... | 49.7 | 3e-04 | G |
| NEW | ref\|XP_568651.1\| | heat shock protein [Cryptococcus neoformans ... | 49.7 | 3e-04 | G |
| NEW | ref\|XP_001321012.1\| | ankyrin repeat protein [Trichomonas vagin... | 49.3 | 3e-04 | G |
| NEW | gb\|EER23793.1\| | Chaperone protein BipA, putative [Coccidioides... | 49.3 | 4e-04 | |
| NEW | ref\|XP_001282379.1\| | ankyrin repeat protein [Trichomonas vagin... | 49.3 | 4e-04 | G |
| NEW | ref\|XP_001676844.1\| | Hypothetical protein CBG14829 [Caenorhabd... | 49.3 | 4e-04 | G |
| NEW | gb\|AAI01275.1\| | ANKDD1A protein [Homo sapiens] | 49.3 | 4e-04 | G |
| NEW | ref\|NP_001129639.1\| | caskin-like [Xenopus laevis] >gb\|AAH92148... | 49.3 | 4e-04 | UG |
| NEW | emb\|CAG09154.1\| | unnamed protein product [Tetraodon nigroviridis] | 49.3 | 4e-04 | |
| NEW | ref\|XP_002196767.1\| | PREDICTED: similar to ankyrin repeat and ... | 49.3 | 4e-04 | UG |
| NEW | emb\|CAQ52958.1\| | CD4-specific ankyrin repeat protein D57.2 [sy... | 49.3 | 4e-04 | |
| NEW | ref\|XP_782809.2\| | PREDICTED: similar to ankyrin 2,3/unc44 [Str... | 49.3 | 4e-04 | G |
| NEW | sp\|Q495B1.1\|AKD1A_HUMAN | RecName: Full=Ankyrin repeat and deat... | 49.3 | 4e-04 | G |
| NEW | gb\|AAI01277.1\| | ANKDD1A protein [Homo sapiens] | 49.3 | 4e-04 | G |
| NEW | ref\|XP_001314663.1\| | ankyrin repeat protein [Trichomonas vagin... | 49.3 | 4e-04 | G |
| NEW | ref\|XP_001317048.1\| | hypothetical protein [Trichomonas vaginal... | 49.3 | 4e-04 | G |
| NEW | ref\|XP_001352050.1\| | Heat shock protein [Plasmodium falciparum... | 49.3 | 4e-04 | UG |
| NEW | emb\|CAA41551.1\| | 70 kDa heat shock protein [Trypanosoma cruzi] | 49.3 | 4e-04 | |

Run PSI-Blast iteration 2 with max

Sequences with E-value WORSE than threshold

Run PSI-Blast iteration 2 with max

Alignments Select All Get selected sequences Distance tree of results Multiple alignment NEW

>**sp|Q5TZF3.1|ANR45_HUMAN** G RecName: Full=Ankyrin repeat domain-containing protein 45
**emb|CAH72999.1|** G ankyrin repeat domain 45 [Homo sapiens]

```
 emb|CAI20937.1|  ankyrin repeat domain 45 [Homo sapiens]
Length=282

 GENE ID: 339416 ANKRD45 | ankyrin repeat domain 45 [Homo sapiens]
(10 or fewer PubMed links)

 Score =  549 bits (1414),  Expect = 1e-154, Method: Compositional matrix adjust.
 Identities = 266/266 (100%), Positives = 266/266 (100%), Gaps = 0/266 (0%)

Query  1    MESEGPPESESSEFFSQQEEENEEEEAQEPEETGPKNPLLQPALTGDVEGLQKIFEDPEN  60
            MESEGPPESESSEFFSQQEEENEEEEAQEPEETGPKNPLLQPALTGDVEGLQKIFEDPEN
Sbjct  17   MESEGPPESESSEFFSQQEEENEEEEAQEPEETGPKNPLLQPALTGDVEGLQKIFEDPEN  76

Query  61   PHHEQAMQLLLEEDIVGRNLLYAACMAGQSDVIRALAKYGVNLNEKTTRGYTLLHCAAAW  120
            PHHEQAMQLLLEEDIVGRNLLYAACMAGQSDVIRALAKYGVNLNEKTTRGYTLLHCAAAW
Sbjct  77   PHHEQAMQLLLEEDIVGRNLLYAACMAGQSDVIRALAKYGVNLNEKTTRGYTLLHCAAAW  136

Query  121  GRLETLKALVELDVDIEALNFREERARDVAARYSQTECVEFLDWADARLTLKKYIAKVSL  180
            GRLETLKALVELDVDIEALNFREERARDVAARYSQTECVEFLDWADARLTLKKYIAKVSL
Sbjct  137  GRLETLKALVELDVDIEALNFREERARDVAARYSQTECVEFLDWADARLTLKKYIAKVSL  196

Query  181  AVTDTEKGSGKLLKEDKNTILSACRAKNEWLETHTEASINELFEQRQQLEDIVTPIFTKM  240
            AVTDTEKGSGKLLKEDKNTILSACRAKNEWLETHTEASINELFEQRQQLEDIVTPIFTKM
Sbjct  197  AVTDTEKGSGKLLKEDKNTILSACRAKNEWLETHTEASINELFEQRQQLEDIVTPIFTKM  256

Query  241  TTPCQVKSAKSVTSHDQKRSQDDTSN  266
            TTPCQVKSAKSVTSHDQKRSQDDTSN
Sbjct  257  TTPCQVKSAKSVTSHDQKRSQDDTSN  282


>ref|NP_940895.1|  ankyrin repeat domain 45 [Homo sapiens]
 dbj|BAC86865.1|  unnamed protein product [Homo sapiens]
 gb|AAI26356.1|  Ankyrin repeat domain 45 [Homo sapiens]
 gb|AAI26354.1|  Ankyrin repeat domain 45 [Homo sapiens]
 gb|EAW90950.1|  hCG1820390 [Homo sapiens]
Length=266

 GENE ID: 339416 ANKRD45 | ankyrin repeat domain 45 [Homo sapiens]
(10 or fewer PubMed links)

 Score =  548 bits (1413),  Expect = 1e-154, Method: Compositional matrix adjust.
 Identities = 266/266 (100%), Positives = 266/266 (100%), Gaps = 0/266 (0%)

Query  1    MESEGPPESESSEFFSQQEEENEEEEAQEPEETGPKNPLLQPALTGDVEGLQKIFEDPEN  60
            MESEGPPESESSEFFSQQEEENEEEEAQEPEETGPKNPLLQPALTGDVEGLQKIFEDPEN
Sbjct  1    MESEGPPESESSEFFSQQEEENEEEEAQEPEETGPKNPLLQPALTGDVEGLQKIFEDPEN  60

Query  61   PHHEQAMQLLLEEDIVGRNLLYAACMAGQSDVIRALAKYGVNLNEKTTRGYTLLHCAAAW  120
            PHHEQAMQLLLEEDIVGRNLLYAACMAGQSDVIRALAKYGVNLNEKTTRGYTLLHCAAAW
Sbjct  61   PHHEQAMQLLLEEDIVGRNLLYAACMAGQSDVIRALAKYGVNLNEKTTRGYTLLHCAAAW  120

Query  121  GRLETLKALVELDVDIEALNFREERARDVAARYSQTECVEFLDWADARLTLKKYIAKVSL  180
            GRLETLKALVELDVDIEALNFREERARDVAARYSQTECVEFLDWADARLTLKKYIAKVSL
Sbjct  121  GRLETLKALVELDVDIEALNFREERARDVAARYSQTECVEFLDWADARLTLKKYIAKVSL  180

Query  181  AVTDTEKGSGKLLKEDKNTILSACRAKNEWLETHTEASINELFEQRQQLEDIVTPIFTKM  240
            AVTDTEKGSGKLLKEDKNTILSACRAKNEWLETHTEASINELFEQRQQLEDIVTPIFTKM
Sbjct  181  AVTDTEKGSGKLLKEDKNTILSACRAKNEWLETHTEASINELFEQRQQLEDIVTPIFTKM  240

Query  241  TTPCQVKSAKSVTSHDQKRSQDDTSN  266
            TTPCQVKSAKSVTSHDQKRSQDDTSN
Sbjct  241  TTPCQVKSAKSVTSHDQKRSQDDTSN  266


>gb|EAW90951.1|  hCG2039282 [Homo sapiens]
Length=270

 Score =  546 bits (1408),  Expect = 7e-154, Method: Compositional matrix adjust.
 Identities = 265/265 (100%), Positives = 265/265 (100%), Gaps = 0/265 (0%)

Query  1    MESEGPPESESSEFFSQQEEENEEEEAQEPEETGPKNPLLQPALTGDVEGLQKIFEDPEN  60
            MESEGPPESESSEFFSQQEEENEEEEAQEPEETGPKNPLLQPALTGDVEGLQKIFEDPEN
Sbjct  6    MESEGPPESESSEFFSQQEEENEEEEAQEPEETGPKNPLLQPALTGDVEGLQKIFEDPEN  65

Query  61   PHHEQAMQLLLEEDIVGRNLLYAACMAGQSDVIRALAKYGVNLNEKTTRGYTLLHCAAAW  120
            PHHEQAMQLLLEEDIVGRNLLYAACMAGQSDVIRALAKYGVNLNEKTTRGYTLLHCAAAW
Sbjct  66   PHHEQAMQLLLEEDIVGRNLLYAACMAGQSDVIRALAKYGVNLNEKTTRGYTLLHCAAAW  125

Query  121  GRLETLKALVELDVDIEALNFREERARDVAARYSQTECVEFLDWADARLTLKKYIAKVSL  180
            GRLETLKALVELDVDIEALNFREERARDVAARYSQTECVEFLDWADARLTLKKYIAKVSL
Sbjct  126  GRLETLKALVELDVDIEALNFREERARDVAARYSQTECVEFLDWADARLTLKKYIAKVSL  185

Query  181  AVTDTEKGSGKLLKEDKNTILSACRAKNEWLETHTEASINELFEQRQQLEDIVTPIFTKM  240
            AVTDTEKGSGKLLKEDKNTILSACRAKNEWLETHTEASINELFEQRQQLEDIVTPIFTKM
Sbjct  186  AVTDTEKGSGKLLKEDKNTILSACRAKNEWLETHTEASINELFEQRQQLEDIVTPIFTKM  245

Query  241  TTPCQVKSAKSVTSHDQKRSQDDTS  265
            TTPCQVKSAKSVTSHDQKRSQDDTS
Sbjct  246  TTPCQVKSAKSVTSHDQKRSQDDTS  270


>ref|XP_001148842.1|  PREDICTED: hypothetical protein [Pan troglodytes]
Length=266

 GENE ID: 738604 ANKRD45 | ankyrin repeat domain 45 [Pan troglodytes]

 Score =  545 bits (1404),  Expect = 2e-153, Method: Compositional matrix adjust.
 Identities = 265/266 (99%), Positives = 265/266 (99%), Gaps = 0/266 (0%)

Query  1    MESEGPPESESSEFFSQQEEENEEEEAQEPEETGPKNPLLQPALTGDVEGLQKIFEDPEN  60
            MESEGPPESESSEFFSQQEEENEEEEAQEPEETGPKNPLLQPALTGDVEGLQKIFEDPEN
Sbjct  1    MESEGPPESESSEFFSQQEEENEEEEAQEPEETGPKNPLLQPALTGDVEGLQKIFEDPEN  60

Query  61   PHHEQAMQLLLEEDIVGRNLLYAACMAGQSDVIRALAKYGVNLNEKTTRGYTLLHCAAAW  120
            PHHEQAMQLLLEEDIVGRNLLYAACMAGQSDVIRALAKYGVNLNEKTTRGYTLLHCAAAW
```

```
Sbjct  61   PHHEQAMQLLLEEDIVGRNLLYAACMAGQSDVIRALAKYGVNLNEKTTRGYTLLHCAAAW  120

Query  121  GRLETLKALVELDVDIEALNFREERARDVAARYSQTECVEFLDWADARLTLKKYIAKVSL  180
            GRLETLKALVELDVDIEALNFREERARDVAARYSQTECVEFLDWADARLTLKKYIAKVSL
Sbjct  121  GRLETLKALVELDVDIEALNFREERARDVAARYSQTECVEFLDWADARLTLKKYIAKVSL  180

Query  181  AVTDTEKGSGKLLKEDKNTILSACRAKNEWLETHTEASINELFEQRQQLEDIVTPIFTKM  240
            AVTDTEKGSGKLLKEDKNTILSACRAKNEWLETHTEASINELFEQRQQLEDIVTPIFTKM
Sbjct  181  AVTDTEKGSGKLLKEDKNTILSACRAKNEWLETHTEASINELFEQRQQLEDIVTPIFTKM  240

Query  241  TTPCQVKSAKSVTSHDQKRSQDDTSN  266
            TTP QVKSAKSVTSHDQKRSQDDTSN
Sbjct  241  TTPRQVKSAKSVTSHDQKRSQDDTSN  266
```

>**ref|XP_001101749.1|** PREDICTED: similar to ankyrin repeat domain 45 [Macaca mulatta]
Length=266

**GENE ID: 706697 ANKRD45** | ankyrin repeat domain 45 [Macaca mulatta]

```
 Score =  500 bits (1288),  Expect = 5e-140, Method: Compositional matrix adjust.
 Identities = 257/266 (96%), Positives = 261/266 (98%), Gaps = 0/266 (0%)

Query  1    MESEGPPESESSEFFSQQEEENEEEEAQEPEETGPKNPLLQPALTGDVEGLQKIFEDPEN  60
            MESEGPPESESSEFFSQQEEE+EEEE QEPEETGP+NPLLQPALTGDVEGLQKIFEDPEN
Sbjct  1    MESEGPPESESSEFFSQQEEEDEEEEVQEPEETGPRNPLLQPALTGDVEGLQKIFEDPEN  60

Query  61   PHHEQAMQLLLEEDIVGRNLLYAACMAGQSDVIRALAKYGVNLNEKTTRGYTLLHCAAAW  120
            PHHEQAMQLLLEEDIVGRNLLYAACMAGQSDVIRALAKYGVNLNEKTTRGYTLLHCAAAW
Sbjct  61   PHHEQAMQLLLEEDIVGRNLLYAACMAGQSDVIRALAKYGVNLNEKTTRGYTLLHCAAAW  120

Query  121  GRLETLKALVELDVDIEALNFREERARDVAARYSQTECVEFLDWADARLTLKKYIAKVSL  180
            GRLETLKALVELDVDIEALNFREERARDVAARYSQTECVEFLDWADARLTLKKYIAKVSL
Sbjct  121  GRLETLKALVELDVDIEALNFREERARDVAARYSQTECVEFLDWADARLTLKKYIAKVSL  180

Query  181  AVTDTEKGSGKLLKEDKNTILSACRAKNEWLETHTEASINELFEQRQQLEDIVTPIFTKM  240
            AVTDTEKGSGKLLKEDKNTILSACRAKNEWLETHTEASINEL EQRQQLED VTPIF KM
Sbjct  181  AVTDTEKGSGKLLKEDKNTILSACRAKNEWLETHTEASINELLEQRQQLEDTVTPIFAKM  240

Query  241  TTPCQVKSAKSVTSHDQKRSQDDTSN  266
            TTP QVKSAKS+TSHDQKRSQDDTS+
Sbjct  241  TTPRQVKSAKSITSHDQKRSQDDTSH  266
```

>**ref|XP_853964.1|** PREDICTED: similar to ankyrin repeat domain 45 [Canis familiaris]
Length=321

**GENE ID: 611232 ANKRD45** | ankyrin repeat domain 45 [Canis lupus familiaris]

```
 Score =  450 bits (1158),  Expect = 5e-125, Method: Compositional matrix adjust.
 Identities = 234/258 (90%), Positives = 238/258 (92%), Gaps = 6/258 (2%)

Query  1    MESEGPPESESSE---FFSQQEEENEEEEAQEPEE---TGPKNPLLQPALTGDVEGLQKI  54
            ME EGPPESESSE   FFSQQEEE EEEE +E +E   TG  NPLLQPALTGDVEGLQKI
Sbjct  63   MEPEGPPESESSEKSIFFSQQEEEGEEEEDEEGKEPEETGTVNPLLQPALTGDVEGLQKI  122

Query  55   FEDPENPHHEQAMQLLLEEDIVGRNLLYAACMAGQSDVIRALAKYGVNLNEKTTRGYTLL  114
            FEDPENPHHEQAMQLLLEEDIVGRNLLYAACMAGQSDVIRALAKYGVNLNEKTTRGYTLL
Sbjct  123  FEDPENPHHEQAMQLLLEEDIVGRNLLYAACMAGQSDVIRALAKYGVNLNEKTTRGYTLL  182

Query  115  HCAAAWGRLETLKALVELDVDIEALNFREERARDVAARYSQTECVEFLDWADARLTLKKY  174
            HCAAAWGRLETLKALVELDVDIEALNFREERARDVAARYSQTECVEFLDWADARL LKKY
Sbjct  183  HCAAAWGRLETLKALVELDVDIEALNFREERARDVAARYSQTECVEFLDWADARLALKKY  242

Query  175  IAKVSLAVTDTEKGSGKLLKEDKNTILSACRAKNEWLETHTEASINELFEQRQQLEDIVT  234
            IAKVS AVTDTEKG GKL KEDKNTIL+ACR KNEWLETH EASINEL EQ+QQLEDIVT
Sbjct  243  IAKVSTAVTDTEKGPGKLFKEDKNTILTACRIKNEWLETHLEASINELSEQKQQLEDIVT  302

Query  235  PIFTKMTTPCQVKSAKSV  252
            PIFTKM TPCQVKSAKSV
Sbjct  303  PIFTKMATPCQVKSAKSV  320
```

>**ref|XP_001493013.1|** PREDICTED: similar to Ankyrin repeat domain-containing protein 45 [Equus caballus]
Length=366

**GENE ID: 100060817 LOC100060817** | similar to hCG2039282 [Equus caballus]

```
 Score =  422 bits (1084),  Expect = 2e-116, Method: Compositional matrix adjust.
 Identities = 218/263 (82%), Positives = 229/263 (87%), Gaps = 8/263 (3%)

Query  1    MESEGPPESESSE---FFSQQEEENEEEEAQE---PEETGPKNPLLQPALTGDVEGLQKI  54
            MESEG  +SESSE   FFSQ EEE E E+ +    PEETG  NPLLQPALTGDVEGLQ I
Sbjct  71   MESEGRQDSESSEKSAFFSQHEEEEEGEDEEAAREPEETGTINPLLQPALTGDVEGLQMI  130

Query  55   FEDPENPHHEQAMQLLLEEDIVGRNLLYAACMAGQSDVIRALAKYGVNLNEKTTRGYTLL  114
            FEDPENPHHEQAMQLLLEEDIVGRNLLYAACMAGQSDVIRALAKYGVNLNEKT RGYTLL
Sbjct  131  FEDPENPHHEQAMQLLLEEDIVGRNLLYAACMAGQSDVIRALAKYGVNLNEKTARGYTLL  190

Query  115  HCAAAWGRLETLKALVELDVDIEALNFREERARDVAARYSQTECVEFLDWADARLTLKKY  174
            HCAAAWGRLETLKALVELDVDIEALNFREERARDVAARYSQTECVEFLDWADARL LKKY
Sbjct  191  HCAAAWGRLETLKALVELDVDIEALNFREERARDVAARYSQTECVEFLDWADARLALKKY  250

Query  175  IAKVSLAVTDTEKGSGKLLKEDKNTILSACRAKNEWLETHTEASINELFEQRQQLEDIVT  234
            IAK S AVTDTEKG GKL KEDKNT+L+ACR +NEWLETH EASINE+FEQ+QQLEDIVT
Sbjct  251  IAKASAAVTDTEKGPGKLFKEDKNTVLNACRVRNEWLETHLEASINEIFEQKQQLEDIVT  310

Query  235  PIFTKMTTPC--QVKSAKSVTSH  255
            PI TKM TP    Q+    ++T H
Sbjct  311  PILTKMATPPTRQLSRKWALTGH  333
```

>**gb|EDM09419.1|** ankyrin repeat domain 45 (predicted) [Rattus norvegicus]
Length=285

```
 GENE ID: 289152 Ankrd45 | ankyrin repeat domain 45 [Rattus norvegicus]

 Score =  380 bits (976),  Expect = 7e-104, Method: Compositional matrix adjust.
 Identities = 182/222 (81%), Positives = 198/222 (89%), Gaps = 0/222 (0%)

Query  22   NEEEEAQEPEETGPKNPLLQPALTGDVEGLQKIFEDPENPHHEQAMQLLLEEDIVGRNLL  81
            ++ EE+QE +ETG +NPLLQP LTGDVEGLQKIFEDPENPHHE A+QLLLEEDIVGRNLL
Sbjct  21   HDYEESQEADETGTENPLLQPTLTGDVEGLQKIFEDPENPHHENAVQLLLEEDIVGRNLL  80

Query  82   YAACMAGQSDVIRALAKYGVNLNEKTTRGYTLLHCAAAWGRLETLKALVELDVDIEALNF  141
            YAACMAG+SDVIRALAKYGVNLNEKT RGYTLLHCAAAWGRLETLKALVELDVDIEALNF
Sbjct  81   YAACMAGKSDVIRALAKYGVNLNEKTARGYTLLHCAAAWGRLETLKALVELDVDIEALNF  140

Query  142  REERARDVAARYSQTECVEFLDWADARLTLKKYIAKVSLAVTDTEKGSGKLLKEDKNTIL  201
            R E+ARDVAARYSQ ECV FLD ADARL LKK+I K SL +TD EK  GKL KEDKN IL
Sbjct  141  RGEKARDVAARYSQAECVHFLDRADARLILKKFITKSSLIITDPEKTPGKLFKEDKNAIL  200

Query  202  SACRAKNEWLETHTEASINELFEQRQQLEDIVTPIFTKMTTP  243
            +ACR KNEWLE+H EASI+ELF Q+QQLEDIV+PI  KM+TP
Sbjct  201  NACRMKNEWLESHPEASISELFVQKQQLEDIVSPIIAKMSTP  242


>ref|XP_213908.4|  PREDICTED: similar to ankyrin repeat domain 45 [Rattus norvegicus]
 ref|XP_001074114.1|  PREDICTED: similar to ankyrin repeat domain 45 [Rattus norvegicus]
Length=357

 GENE ID: 289152 Ankrd45 | ankyrin repeat domain 45 [Rattus norvegicus]

 Score =  379 bits (972),  Expect = 2e-103, Method: Compositional matrix adjust.
 Identities = 182/222 (81%), Positives = 198/222 (89%), Gaps = 0/222 (0%)

Query  22   NEEEEAQEPEETGPKNPLLQPALTGDVEGLQKIFEDPENPHHEQAMQLLLEEDIVGRNLL  81
            ++ EE+QE +ETG +NPLLQP LTGDVEGLQKIFEDPENPHHE A+QLLLEEDIVGRNLL
Sbjct  131  HDYEESQEADETGTENPLLQPTLTGDVEGLQKIFEDPENPHHENAVQLLLEEDIVGRNLL  190

Query  82   YAACMAGQSDVIRALAKYGVNLNEKTTRGYTLLHCAAAWGRLETLKALVELDVDIEALNF  141
            YAACMAG+SDVIRALAKYGVNLNEKT RGYTLLHCAAAWGRLETLKALVELDVDIEALNF
Sbjct  191  YAACMAGKSDVIRALAKYGVNLNEKTARGYTLLHCAAAWGRLETLKALVELDVDIEALNF  250

Query  142  REERARDVAARYSQTECVEFLDWADARLTLKKYIAKVSLAVTDTEKGSGKLLKEDKNTIL  201
            R E+ARDVAARYSQ ECV FLD ADARL LKK+I K SL +TD EK  GKL KEDKN IL
Sbjct  251  RGEKARDVAARYSQAECVHFLDRADARLILKKFITKSSLIITDPEKTPGKLFKEDKNAIL  310

Query  202  SACRAKNEWLETHTEASINELFEQRQQLEDIVTPIFTKMTTP  243
            +ACR KNEWLE+H EASI+ELF Q+QQLEDIV+PI  KM+TP
Sbjct  311  NACRMKNEWLESHPEASISELFVQKQQLEDIVSPIIAKMSTP  352


>sp|Q810N6.1|ANR45_MOUSE  RecName: Full=Ankyrin repeat domain-containing protein 45
 gb|AAH49713.1|  Ankrd45 protein [Mus musculus]
Length=248

 GENE ID: 73844 Ankrd45 | ankyrin repeat domain 45 [Mus musculus]
(Over 10 PubMed links)

 Score =  374 bits (960),  Expect = 6e-102, Method: Compositional matrix adjust.
 Identities = 176/211 (83%), Positives = 191/211 (90%), Gaps = 0/211 (0%)

Query  33   TGPKNPLLQPALTGDVEGLQKIFEDPENPHHEQAMQLLLEEDIVGRNLLYAACMAGQSDV  92
            TG +NPLLQP LTGDVEGLQKIFEDPE+PHHE A+QLLLEEDIVGRNLLYAACMAG+SDV
Sbjct  32   TGAENPLLQPTLTGDVEGLQKIFEDPEHPHHEHAVQLLLEEDIVGRNLLYAACMAGKSDV  91

Query  93   IRALAKYGVNLNEKTTRGYTLLHCAAAWGRLETLKALVELDVDIEALNFREERARDVAAR  152
            I+ALAKYGVNLNE T RGYTLLHCAAAWGRLETLKALVELDVDIEALNFR E+ARDVAAR
Sbjct  92   IKALAKYGVNLNEATARGYTLLHCAAAWGRLETLKALVELDVDIEALNFRGEKARDVAAR  151

Query  153  YSQTECVEFLDWADARLTLKKYIAKVSLAVTDTEKGSGKLLKEDKNTILSACRAKNEWLE  212
            YSQ ECV FLDWADARL LKK I K SL +TD EKG GKL KEDK+TIL+ACR KNEWLE
Sbjct  152  YSQVECVNFLDWADARLILKKIITKSSLIITDPEKGPGKLFKEDKSTILNACRLKNEWLE  211

Query  213  THTEASINELFEQRQQLEDIVTPIFTKMTTP  243
            +H EASI+E+FEQ+QQLEDIV+PI  KM+TP
Sbjct  212  SHPEASISEIFEQKQQLEDIVSPILAKMSTP  242


>ref|NP_082940.1|  ankyrin repeat domain 45 [Mus musculus]
 gb|EDL39318.1|  mCG9408 [Mus musculus]
Length=282

 GENE ID: 73844 Ankrd45 | ankyrin repeat domain 45 [Mus musculus]
(Over 10 PubMed links)

 Score =  373 bits (957),  Expect = 1e-101, Method: Compositional matrix adjust.
 Identities = 176/211 (83%), Positives = 191/211 (90%), Gaps = 0/211 (0%)

Query  33   TGPKNPLLQPALTGDVEGLQKIFEDPENPHHEQAMQLLLEEDIVGRNLLYAACMAGQSDV  92
            TG +NPLLQP LTGDVEGLQKIFEDPE+PHHE A+QLLLEEDIVGRNLLYAACMAG+SDV
Sbjct  66   TGAENPLLQPTLTGDVEGLQKIFEDPEHPHHEHAVQLLLEEDIVGRNLLYAACMAGKSDV  125

Query  93   IRALAKYGVNLNEKTTRGYTLLHCAAAWGRLETLKALVELDVDIEALNFREERARDVAAR  152
            I+ALAKYGVNLNE T RGYTLLHCAAAWGRLETLKALVELDVDIEALNFR E+ARDVAAR
Sbjct  126  IKALAKYGVNLNEATARGYTLLHCAAAWGRLETLKALVELDVDIEALNFRGEKARDVAAR  185

Query  153  YSQTECVEFLDWADARLTLKKYIAKVSLAVTDTEKGSGKLLKEDKNTILSACRAKNEWLE  212
            YSQ ECV FLDWADARL LKK I K SL +TD EKG GKL KEDK+TIL+ACR KNEWLE
Sbjct  186  YSQVECVNFLDWADARLILKKIITKSSLIITDPEKGPGKLFKEDKSTILNACRLKNEWLE  245

Query  213  THTEASINELFEQRQQLEDIVTPIFTKMTTP  243
            +H EASI+E+FEQ+QQLEDIV+PI  KM+TP
Sbjct  246  SHPEASISEIFEQKQQLEDIVSPILAKMSTP  276


>dbj|BAB30413.1|  unnamed protein product [Mus musculus]
Length=164
```

```
 GENE ID: 73844 Ankrd45 | ankyrin repeat domain 45 [Mus musculus]
(Over 10 PubMed links)

 Score =  273 bits (699),  Expect = 1e-71, Method: Compositional matrix adjust.
 Identities = 129/158 (81%), Positives = 141/158 (89%), Gaps = 0/158 (0%)

Query  86   MAGQSDVIRALAKYGVNLNEKTTRGYTLLHCAAAWGRLETLKALVELDVDIEALNFREER  145
            MAG+SDVI+ALAKYGVNLNE T RGYTLLHCAAAWGRLETLKALVELDVDIEALNFR E+
Sbjct  1    MAGKSDVIKALAKYGVNLNEATARGYTLLHCAAAWGRLETLKALVELDVDIEALNFRGEK  60

Query  146  ARDVAARYSQTECVEFLDWADARLTLKKYIAKVSLAVTDTEKGSGKLLKEDKNTILSACR  205
            ARDVAARYSQ ECV FLDWADARL LKK I K SL +TD EKG GKL KEDK+TIL+ACR
Sbjct  61   ARDVAARYSQVECVNFLDWADARLILKKIITKSSLIITDPEKGPGKLFKEDKSTILNACR  120

Query  206  AKNEWLETHTEASINELFEQRQQLEDIVTPIFTKMTTP  243
             KNEWLE+H EASI+E+FEQ+QQLEDIV+PI  KM+TP
Sbjct  121  LKNEWLESHPEASISEIFEQKQQLEDIVSPILAKMSTP  158


>ref|XP_001373170.1|  PREDICTED: similar to hCG2039282 [Monodelphis domestica]
Length=235

 GENE ID: 100020813 LOC100020813 | similar to hCG2039282 [Monodelphis domestica]

 Score =  262 bits (670),  Expect = 2e-68, Method: Compositional matrix adjust.
 Identities = 132/225 (58%), Positives = 173/225 (76%), Gaps = 0/225 (0%)

Query  18   QEEENEEEEAQEPEETGPKNPLLQPALTGDVEGLQKIFEDPENPHHEQAMQLLLEEDIVG  77
            +E + ++E + +PEE   +NPLL   L+G++E L++IFEDPE P H +AM L+++ED++G
Sbjct  7    KEYDLDQELSLDPEELDGENPLLPTILSGNIEQLERIFEDPEEPLHSRAMSLIMKEDVIG  66

Query  78   RNLLYAACMAGQSDVIRALAKYGVNLNEKTTRGYTLLHCAAAWGRLETLKALVELDVDIE  137
            RNLL+ AC+AGQS+VI+ L KYGVNLNEKTTRGYTLLH AAAWGRL+T+K LVE +V+++
Sbjct  67   RNLLFTACIAGQSNVIKTLTKYGVNLNEKTTRGYTLLHIAAAWGRLDTVKMLVEQEVELD  126

Query  138  ALNFREERARDVAARYSQTECVEFLDWADARLTLKKYIAKVSLAVTDTEKGSGKLLKEDK  197
             LNF  E  RD+A R+SQ ECV +LD A ARL LKK IAKV    + D EKG GKL +EDK
Sbjct  127  VLNFLNETPRDIALRFSQMECVCYLDVAAARLALKKAIAKVQGIILDPEKGPGKLNREDK  186

Query  198  NTILSACRAKNEWLETHTEASINELFEQRQQLEDIVTPIFTKMTT  242
            N + S CR+K EWL+ H    ++ EL EQ+QQLEDIV PIF K++T
Sbjct  187  NLLNSTCRSKLEWLDLHPTPTVEELEEQKQQLEDIVNPIFVKIST  231


>ref|NP_001088745.1|  hypothetical protein LOC496009 [Xenopus laevis]
 gb|AAH87400.1|  LOC496009 protein [Xenopus laevis]
Length=227

 GENE ID: 496009 LOC496009 | hypothetical LOC496009 [Xenopus laevis]
(10 or fewer PubMed links)

 Score =  218 bits (554),  Expect = 6e-55, Method: Compositional matrix adjust.
 Identities = 100/208 (48%), Positives = 151/208 (72%), Gaps = 1/208 (0%)

Query  37   NPLLQPALTGDVEGLQKIFEDPENPHHEQAMQLLLEEDIVGRNLLYAACMAGQSDVIRAL  96
            NP+L+ AL  D++ L+ +FED   P   +A  LLL++D++GRN+L+ AC+ G+ ++++ L
Sbjct  11   NPVLECALKDDLQALKMLFEDAPEPGKGRATDLLLKKDLMGRNVLFPACILGRCEIVKEL  70

Query  97   AKYGVNLNEKTTRGYTLLHCAAAWGRLETLKALVELDVDIEALNFREERARDVAARYSQT  156
             KYG NLN +T+RGY+LLHCAAAWG+L+ LK L+ELD +++A NF  E+A ++A RY++T
Sbjct  71   IKYGANLNSRTSRGYSLLHCAAAWGQLDMLKTLIELDANVKACNFCNEKAYEIAVRYNKT  130

Query  157  ECVEFLDWADARLTLKKYIAKVSLAVTDTEKGSGKLLKEDKNTILSACRAKNEWLETHTE  216
            EC +FL WA+A+L LK YI+ V  + TD EK  GKL KE K+  ++AC++KN+WLE
Sbjct  131  ECADFLAWAEAKLELKMYISFVQQSFTDLEKTQGKLNKEYKHQTMAACKSKNDWLEHTKN  190

Query  217  ASINELFEQRQQLEDIVTPIFTKM-TTP  243
             + ++  EQ+ QLE I+  I +K+ TTP
Sbjct  191  PTTHDFVEQKLQLEAIMQTILSKLNTTP  218


>gb|AAH84990.1|  Ankrd45-prov protein [Xenopus tropicalis]
Length=205

 GENE ID: 496592 ankrd45 | ankyrin repeat domain 45
[Xenopus (Silurana) tropicalis] (10 or fewer PubMed links)

 Score =  206 bits (525),  Expect = 1e-51, Method: Compositional matrix adjust.
 Identities = 96/196 (48%), Positives = 141/196 (71%), Gaps = 2/196 (1%)

Query  47   DVEGLQKIFEDPENPHHEQAMQLLLEEDIVGRNLLYAACMAGQSDVIRALAKYGVNLNEK  106
            D++ L+ +FEDP  P  E+   LLL++D +GRN+L+ AC+ G+ +V++ L KYG ++N
Sbjct  1    DLQALKLLFEDPSEP--EKVTHLLLKKDFMGRNVLFPACILGRCEVVKELIKYGASVNSL  58

Query  107  TTRGYTLLHCAAAWGRLETLKALVELDVDIEALNFREERARDVAARYSQTECVEFLDWAD  166
            T+RGY+ LHCAAAWG+L+ LK LVE+  DI+A NF  E+A ++A RY++TEC +FL WA+
Sbjct  59   TSRGYSPLHCAAAWGQLDMLKTLVEMGADIKACNFCNEKAYEIAIRYNKTECADFLAWAE  118

Query  167  ARLTLKKYIAKVSLAVTDTEKGSGKLLKEDKNTILSACRAKNEWLETHTEASINELFEQR  226
            A+L LK YI+ V  ++TD EK  GKL KE KN  ++AC++KNEWLE      + ++  EQ+
Sbjct  119  AKLELKIYISFVHQSITDLEKLQGKLNKEYKNQTMAACKSKNEWLEHTKNPTTHDFVEQK  178

Query  227  QQLEDIVTPIFTKMTT  242
             QLE I+  IF+K+ T
Sbjct  179  LQLEAIMQTIFSKLKT  194


>emb|CAQ14704.1|  novel protein (zgc:112418) [Danio rerio]
Length=224

 Score =  189 bits (479),  Expect = 3e-46, Method: Compositional matrix adjust.
 Identities = 92/203 (45%), Positives = 134/203 (66%), Gaps = 1/203 (0%)

Query  39   LLQPALTGDVEGLQKIFEDPENPHHEQAMQLLLEEDIVGRNLLYAACMAGQSDVIRALAK  98
            +L  AL  D+EGL+ I E         Q+  +L E+D VGRN L+AACM G+S ++R L +
Sbjct  9    VLLCALDDDLEGLKGILERTFTDDAAQSENILWEKDEVGRNALFAACMMGRSAIVRELVQ  68
```

```
Query  99   YGV-NLNEKTTRGYTLLHCAAAWGRLETLKALVELDVDIEALNFREERARDVAARYSQTE  157
             G  ++NE T RGY+ LHC+A WG+L+TLK LVEL+ D +A+NFR E+A DVA RY + +
Sbjct  69   NGAADVNELTARGYSPLHCSAMWGQLDTLKTLVELNADFQAINFRGEKAVDVARRYDKLD  128

Query  158  CVEFLDWADARLTLKKYIAKVSLAVTDTEKGSGKLLKEDKNTILSACRAKNEWLETHTEA  217
            C E+L WA+A+  L+ +I +V   V D EK  GKL KEDKN  ++ C AK++W+      A
Sbjct  129  CAEYLAWAEAKQNLQAFIQEVRAIVADQEKVQGKLNKEDKNICINTCSAKSDWINNTRTA  188

Query  218  SINELFEQRQQLEDIVTPIFTKM  240
            +  +  EQ++ LED++ P+  K+
Sbjct  189  TAQDFIEQKKLLEDVLAPVLLKL  211
```

>**ref|NP_001018606.1|** ankyrin repeat domain 45 [Danio rerio]
**gb|AAH95811.1|** Zgc:112418 [Danio rerio]
Length=224

**GENE ID: 553808 zgc:112418** | zgc:112418 [Danio rerio]
(10 or fewer PubMed links)

Score = 188 bits (478), Expect = 4e-46, Method: Compositional matrix adjust.
Identities = 93/203 (45%), Positives = 133/203 (65%), Gaps = 1/203 (0%)

```
Query  39   LLQPALTGDVEGLQKIFEDPENPHHEQAMQLLLEEDIVGRNLLYAACMAGQSDVIRALAK  98
            +L  AL  D+EGL+ I E        Q+  +L E+D VGRN L+AACM G+S ++R L +
Sbjct  9    VLLCALDDDLEGLKGILERTFTDDAAQSENILWEKDEVGRNALFAACMMGRSAIVRELVQ  68

Query  99   YGV-NLNEKTTRGYTLLHCAAAWGRLETLKALVELDVDIEALNFREERARDVAARYSQTE  157
             G  ++NE T RGY+ LHC+A WG+L+TLK LVEL+ D +A+NFR E+A DVA RY + +
Sbjct  69   NGAADVNELTARGYSPLHCSAMWGQLDTLKTLVELNADFQAINFRGEKAVDVARRYDKLD  128

Query  158  CVEFLDWADARLTLKKYIAKVSLAVTDTEKGSGKLLKEDKNTILSACRAKNEWLETHTEA  217
            C E+L WA+A+  L+ +I +V   V D EK  GKL KEDKN  ++ C AK+EW+      A
Sbjct  129  CAEYLAWAEAKQNLQAFIQEVRAIVADQEKVQGKLNKEDKNICINTCSAKSEWINNTRTA  188

Query  218  SINELFEQRQQLEDIVTPIFTKM  240
            +     EQ++ LED++ P+  K+
Sbjct  189  TAQVFIEQKKLLEDVLAPVLLKL  211
```

>**ref|XP_002131508.1|** PREDICTED: similar to mCG9408 [Ciona intestinalis]
Length=238

**GENE ID: 100185472 LOC100185472** | similar to mCG9408 [Ciona intestinalis]

Score = 154 bits (390), Expect = 6e-36, Method: Compositional matrix adjust.
Identities = 81/226 (35%), Positives = 129/226 (57%), Gaps = 2/226 (0%)

```
Query  20   EENEEEEAQE-PEETGPKNPLLQPALTGDVEGLQKIFEDPENPHHEQAMQLLLEEDIVGR  78
            EE EE    +   PE+     N ++    L   D E    K+F+D ENP++E A  LL +    VG+
Sbjct  4    EEGEESPRESIPEKFDGGNVIMHCVLHEDKERFAKVFDDAENPYYESASVLLKQRSEVGK  63

Query  79   NLLYAACMAGQSDVIRALAKYGVNLNEKTTRGYTLLHCAAAWGRLETLKALVELDVDIEA  138
            + +  ACM G++D+++ + K G ++N      GY  +H AAAWG ++ L+ALVE    ++
Sbjct  64   SPIEVACMLGRADILKDIIKRGCDVNAANGSGYCPIHFAAAWGHVKCLEALVESGASVDC  123

Query  139  LNFREERARDVAARYSQTECVEFLDWADARLTLKKYIAKVSLAVTDTEKGSG-KLLKEDK  197
                 E A+++A RY     CV +L+WA A+L L  +I+      + D EK  G KL KE+K
Sbjct  124  TTRHGELAKEIAFRYDHNHCVYYLNWAAAKLALVAFISHARELIADPEKLQGIKLTKEEK  183

Query  198  NTILSACRAKNEWLETHTEASINELFEQRQQLEDIVTPIFTKMTTP  243
             T+L +C  K EWLE+ T+A+ ++  ++ +LE  +  I  K  +P
Sbjct  184  TTVLGSCSEKQEWLESTTDATADDFIAKKTELEKQIDVILNKTISP  229
```

>**ref|XP_001639234.1|** predicted protein [Nematostella vectensis]
**gb|EDO47171.1|** predicted protein [Nematostella vectensis]
Length=221

**GENE ID: 5519335 NEMVEDRAFT v1g236489** | hypothetical protein
[Nematostella vectensis] (10 or fewer PubMed links)

Score = 147 bits (372), Expect = 8e-34, Method: Compositional matrix adjust.
Identities = 74/203 (36%), Positives = 124/203 (61%), Gaps = 1/203 (0%)

```
Query  39   LLQPALTGDVEGLQKIFEDPENPHHEQAMQLLLEEDIVGRNLLYAACMAGQSDVIRALAK  98
            L   A  GD++ ++++FEDPE+P+   A    L + +  G++ +  A M G+++V+R L +
Sbjct  5    LFDFATEGDIDSIRQLFEDPESPYVTDASTELNKRNPDGKSAIDLAAMLGRNEVVRELLE  64

Query  99   YGVNLNEKTTRGYTLLHCAAAWGRLETLKALVELDVDIEALNFREERARDVAARYSQTEC  158
             G  +N KT +GYT LH AA WG++  LKALV    D++  N   ERAR+ A RY++ +C
Sbjct  65   RGAEVNSKTKKGYTCLHIAACWGQVGCLKALVASGADLQIRNAHGERAREAATRYNKVDC  124

Query  159  VEFLDWADARLTLKKYIAKVSLAVTDTEKGSGKLLKEDKNTILSACRAKNEWLETHTE-A  217
            +E+LD A+A+   LK  I      + D +K  G+  K+D+ +     C  K+EWLE + + A
Sbjct  125  IEYLDKAEAQFELKALITSTKETIADPDKHMGRFTKDDRVSGNRYCDEKSEWLENNADTA  184

Query  218  SINELFEQRQQLEDIVTPIFTKM  240
            S+ E+ +Q++ L  ++ PI +K+
Sbjct  185  SLEEIVKQKEDLAGVLQPILSKL  207
```

>**ref|XP_002609257.1|** hypothetical protein BRAFLDRAFT_124756 [Branchiostoma floridae]
**gb|EEN65267.1|** hypothetical protein BRAFLDRAFT_124756 [Branchiostoma floridae]
Length=241

**GENE ID: 7245666 BRAFLDRAFT 124756** | hypothetical protein
[Branchiostoma floridae] (10 or fewer PubMed links)

Score = 146 bits (369), Expect = 2e-33, Method: Compositional matrix adjust.
Identities = 76/206 (36%), Positives = 125/206 (60%), Gaps = 1/206 (0%)

```
Query  37   NPLLQPALTGDVEGLQKIFEDPENPHHEQAMQLLLEEDIVGRNLLYAACMAGQSDVIRAL  96
            N  +  AL+G+V  L +  +D E+P+H +  + L E D+ G++ L  A M G+ +V++ L
```

```
Sbjct  25   NLAMAAALSGEVSRLCRALDDEEDPYHPEIQERLNERDLEGKSPLDMASMLGRDNVVKEL  84

Query  97   AKYGVNLNEKTTRGYTLLHCAAAWGRLETLKALVELDVDIEALNFREERARDVAARYSQT  156
               G + N  T RGYT LH AA+WG+L  +K LV+   D++      ERAR++A RY+
Sbjct  85   LVRGADPNIATPRGYTALHRAASWGKLACVKILVQFQADLQKRTHHGERAREIAVRYAMD  144

Query  157  ECVEFLDWADARLTLKKYIAKVSLAVTDTEK-GSGKLLKEDKNTILSACRAKNEWLETHT  215
            +CV++LD  +AR TLK Y+A +   + D EK G+ KL KE+K +  +A   K +WLE  T
Sbjct  145  DCVQYLDRQEARQTLKSYVASMRETLADPEKMGAAKLTKEEKTSTNAALTEKEQWLENTT  204

Query  216  EASINELFEQRQQLEDIVTPIFTKMT  241
            +AS+ +  +++ +LE  +  +  K++
Sbjct  205  DASVQDYLDKKSELEQQMETVMLKIS  230
```

>**gb|ACC54983.1|** ankyrin repeat domain 45 [Xenopus borealis]
Length=130

```
 Score =  144 bits (362),  Expect = 1e-32, Method: Compositional matrix adjust.
 Identities = 64/129 (49%), Positives = 94/129 (72%), Gaps = 0/129 (0%)

Query  84   ACMAGQSDVIRALAKYGVNLNEKTTRGYTLLHCAAAWGRLETLKALVELDVDIEALNFRE  143
            AC+ G+ ++++ L K+G N+N  T+RGY+ LHCAAAWG+L+ LK LVELD +++A NF
Sbjct  1    ACILGRCEIVKELIKHGANVNSLTSRGYSPLHCAAAWGQLDMLKTLVELDANVKACNFCN  60

Query  144  ERARDVAARYSQTECVEFLDWADARLTLKKYIAKVSLAVTDTEKGSGKLLKEDKNTILSA  203
            E+  ++A RY++ EC +FL WA+A+L LK YI+ V  +    EK  GKL KE K+  ++A
Sbjct  61   EKGYEIAVRYNKIECADFLAWAEAKLDLKMYISFVQQSFIGLEKMQGKLNKEYKHQTMAA  120

Query  204  CRAKNEWLE  212
            C+AKN+WLE
Sbjct  121  CKAKNDWLE  129
```

>**ref|XP_783791.1|** PREDICTED: similar to Ankrd45 protein [Strongylocentrotus purpuratus]
 **ref|XP_001202089.1|** PREDICTED: similar to Ankrd45 protein [Strongylocentrotus purpuratus]
Length=274

 **GENE ID: 578535 LOC578535** | similar to Ankrd45 protein
[Strongylocentrotus purpuratus]

```
 Score =  128 bits (322),  Expect = 5e-28, Method: Compositional matrix adjust.
 Identities = 80/252 (31%), Positives = 126/252 (50%), Gaps = 36/252 (14%)

Query  28   QEPEETGPK--NPLLQPALTGDVEGLQKIFEDPENPHHEQA-------------------  66
            Q  + +G K  N ++Q A +GD   L + F + E+P+H++
Sbjct  15   QSGKRSGKKRLNIVIQCAASGDTSRLLECFANEEDPYHDRVESQLNSADEEGRSPVEIAV  74

Query  67   ------MQLLLEEDIVG---RNLLYA------ACMAGQSDVIRALAKYGVNLNEKTTRGY  111
                  M  LL+E   G   RN + A      AC+ G+++ ++AL   G + N  T RGY
Sbjct  75   TENQIEMLKLLQEKGSGLDTRNSMSARTPLDMACILGRNEALKALLAGGADANNSTKRGY  134

Query  112  TLLHCAAAWGRLETLKALVELDVDIEALNFREERARDVAARYSQTECVEFLDWADARLTL  171
            T +H AAAWGR++ LK LV+    +       ERARD A RY   +C  +LDW++AR  L
Sbjct  135  TAIHHAAAWGRMDCLKTLVKYGASLTIKTKHGERARDTALRYKHEDCSFYLDWSEARRGL  194

Query  172  KKYIAKVSLAVTDTEKGSGKLLKEDKNTILSACRAKNEWLETHTEASINELFEQRQQLED  231
               + +    + D +K  G+L K++K T L+ C  K EWL+++  ASI +  +Q + L
Sbjct  195  VSILQETKETIEDPQKLQGRLAKDEKMTGLNVCGEKQEWLDSNPNASIEDFRKQEEDLRT  254

Query  232  IVTPIFTKMTTP  243
             +  I  K++ P
Sbjct  255  SLEAILIKLSEP  266
```

>**ref|XP_002109388.1|** hypothetical protein TRIADDRAFT_53367 [Trichoplax adhaerens]
 **gb|EDV27554.1|** hypothetical protein TRIADDRAFT_53367 [Trichoplax adhaerens]
Length=232

 **GENE ID: 6751162 TRIADDRAFT_53367** | hypothetical protein [Trichoplax adhaerens]
(10 or fewer PubMed links)

```
 Score =  102 bits (254),  Expect = 4e-20, Method: Compositional matrix adjust.
 Identities = 64/203 (31%), Positives = 106/203 (52%), Gaps = 3/203 (1%)

Query  39   LLQPALTGDVEGLQKIFEDPENPHHEQAMQLLLEEDIVGRNLLYAACMAGQSDVIRALAK  98
            +++  +  D E +Q+   + E    E   +LL   D  G++ L  ACM G+ DVI  L
Sbjct  5    IIKAVIYNDAEKIQQFITNEEG---EINGELLDIRDDEGKSPLDIACMLGKVDVIEGLIA  61

Query  99   YGVNLNEKTTRGYTLLHCAAAWGRLETLKALVELDVDIEALNFREERARDVAARYSQTEC  158
             G N++  +++G T +H AA+WGR+E LK L E   ++   N + E  RD+A RY  + C
Sbjct  62   NGANMDSISSQGNTAMHRAASWGRIECLKILAEKGANLHLKNLKGECPRDIARRYGHSAC  121

Query  159  VEFLDWADARLTLKKYIAKVSLAVTDTEKGSGKLLKEDKNTILSACRAKNEWLETHTEAS  218
             ++L  A  R   K  +   +  +TD +  +GKL K+D+    +  +    EW E++T+
Sbjct  122  YDYLVRAAIRRNYKYKMLAYNETLTDHDLIAGKLNKDDRIQAAALFKTLTEWYESNTDPI  181

Query  219  INELFEQRQQLEDIVTPIFTKMT  241
            I E+ EQ + L + V P   K+
Sbjct  182  IAEVQEQTKVLLEGVLPFIRKLI  204
```

>**ref|XP_001255172.1|** PREDICTED: similar to solute carrier family 9, member 11 [Bos taurus]
Length=1267

 **GENE ID: 787966 SLC9A11** | solute carrier family 9, member 11 [Bos taurus]

```
 Score = 92.4 bits (228),  Expect = 5e-17, Method: Composition-based stats.
 Identities = 40/47 (85%), Positives = 43/47 (91%), Gaps = 0/47 (0%)

Query  197  KNTILSACRAKNEWLETHTEASINELFEQRQQLEDIVTPIFTKMTTP  243
            +N +LSACR KNEWLETH EASINELFEQ+QQLEDIVTPIFTKM TP
Sbjct  36   ENILLSACRVKNEWLETHLEASINELFEQKQQLEDIVTPIFTKMATP  82
```

```
>ref|XP_002572929.1| Ankrd45 protein [Schistosoma mansoni]
 emb|CAZ29161.1| Ankrd45 protein , putative [Schistosoma mansoni]
Length=207

 GENE ID: 8347287 Smp_130080 | Ankrd45 protein [Schistosoma mansoni]
(10 or fewer PubMed links)

 Score = 67.8 bits (164),  Expect = 1e-09, Method: Compositional matrix adjust.
 Identities = 58/220 (26%), Positives = 100/220 (45%), Gaps = 34/220 (15%)

Query  39   LLQPALTGDVEGLQKIFE----DPE---------NPHHEQAMQLLLEEDIVGRNLLYAAC  85
            L++  L GD + +QK+ +    DPE         +  H  A++L    D    RN++  A
Sbjct  4    LIELILQGDSDKIQKLLKEYDKDPEGYLQSMNEYDEMHNSAIELFTILD--SRNIIEKAI  61

Query  86   MAGQSDVIRALAKYGVNLNEKTTRGYTLLHCAAAWGRLETLKALVELDVDIEALNFREER  145
              G ++             LN      G  L+H AA W    + +K L    VD+    N   E
Sbjct  62   SNGYNE-----------LNIIGKNGCNLVHYAAMWNHADLIKYLYFAGVDVYRKNVHGET  110

Query  146  ARDVAARYSQTECVEFLDWADAR---LTLKKYIAKVSLAVTDTEKGSGKLLKEDKNTILS  202
            A  +A +Y Q E +  L+W + R   + L + + ++ LA +D    +    KE++    S
Sbjct  111  AHKLAVKYEQKEAMHILEWIECRDEFIMLIRLVREI-LATSDKNDYT----KEERKIADS  165

Query  203  ACRAKNEWLETHTEASINELFEQRQQLEDIVTPIFTKMTT  242
            AC     W+   + EA+++ L   +++Q+E IV P    K ++
Sbjct  166  ACLDGESWINKNKEATLSMLKTKKEQIELIVEPFLRKKSS  205


>ref|XP_002423923.1| hspc200, putative [Pediculus humanus corporis]
 gb|EEB11185.1| hspc200, putative [Pediculus humanus corporis]
Length=845

 GENE ID: 8238041 Phum_PHUM097030 | hspc200, putative
[Pediculus humanus corporis]

 Score = 62.8 bits (151),  Expect = 3e-08, Method: Compositional matrix adjust.
 Identities = 41/110 (37%), Positives = 56/110 (50%), Gaps = 10/110 (9%)

Query  63   HEQAMQLLLEE-------DIVGRNLLYAACMAGQSDVIRALAKYGV---NLNEKTTRGYT  112
            H+   ++LLL          D  G + L+ A  AG  D++R L  +G    N+N  T    T
Sbjct  30   HQDIVELLLTHEASTNILDAKGSSPLHLAAWAGNVDIVRLLLCHGPSVPNVNLTTKDHET  89

Query  113  LLHCAAAWGRLETLKALVELDVDIEALNFREERARDVAARYSQTECVEFL  162
             LHCAA +G  E +  L+E   D    N REE A D+AA+Y + E VE L
Sbjct  90   ALHCAAQYGHTEIVTLLLEHSCDPTIRNSREETALDLAAQYGRLETVELL  139


 Score = 37.7 bits (86),  Expect = 1.2, Method: Compositional matrix adjust.
 Identities = 23/94 (24%), Positives = 47/94 (50%), Gaps = 1/94 (1%)

Query  81   LYAACMAGQSDVIRALAKYGVNLNEKTTRGYTLLHCAAAWGRLETLKALVELDVDIEALN  140
            L+ A   G   V+  L   G+++N KT+ G T LH AA  G+L+ ++ L++  VD+   +
Sbjct  165  LHLASRNGHKLVVEILLSSGMDVNLKTSGG-TALHEAALCGKLDVVRTLLDAGVDLRLKD  223

Query  141  FREERARDVAARYSQTECVEFLDWADARLTLKKY  174
             ++     D+  ++ +     + L         ++K+
Sbjct  224  AKQNTVLDLLRQFPKHVTQDILTIIKKHQNVQKF  257


>ref|XP_002585519.1| hypothetical protein BRAFLDRAFT_133193 [Branchiostoma floridae]
 gb|EEN41530.1| hypothetical protein BRAFLDRAFT_133193 [Branchiostoma floridae]
Length=476

 GENE ID: 7238920 BRAFLDRAFT_133193 | hypothetical protein
[Branchiostoma floridae] (10 or fewer PubMed links)

 Score = 62.0 bits (149),  Expect = 6e-08, Method: Compositional matrix adjust.
 Identities = 51/155 (32%), Positives = 70/155 (45%), Gaps = 9/155 (5%)

Query  31   EETGPKNPLLQPALTGDVEGLQKIFEDPENPHHEQAMQLLLEE------DIVGRNLLYAA  84
            E  G   PL+   L   +E LQ       E  H E     LL          D  G  LL+ A
Sbjct  158  ENFGFCQPLMDKHL---LEALQHSSVSTEGDHDEACSVLLAGGADIGVVDHYGCTLLHYA  214

Query  85   CMAGQSDVIRALAKYGVNLNEKTTRGYTLLHCAAAWGRLETLKALVELDVDIEALNFREE  144
               G +D I  L    G +L+ + T G+ LLH AA  G  +T+  L++ DVDIEA +F
Sbjct  215  AFKGNNDAILLLLDRGADLDARNTYGHFLLHSAALGGHNDTINLLLDRDVDIEAEDFGGR  274

Query  145  RARDVAARYSQTECVEFLDWADARLTLKKYIAKVS  179
             A   AA+Y    + V+ L       LT K   A++++
Sbjct  275  TALHFAAQYGHHKTVQLLCSRGGDLTGKDKFAEMT  309


>ref|NP_999979.1| tumor protein p53 binding protein, 2 [Danio rerio]
 gb|AAH70005.1| Tumor protein p53 binding protein, 2 [Danio rerio]
Length=1060

 GENE ID: 407983 tp53bp2 | tumor protein p53 binding protein, 2 [Danio rerio]
(10 or fewer PubMed links)

 Score = 62.0 bits (149),  Expect = 7e-08, Method: Compositional matrix adjust.
 Identities = 38/132 (28%), Positives = 68/132 (51%), Gaps = 22/132 (16%)

Query  39   LLQPALTGDVEGLQKIF---EDPENPHHEQAMQLLLEEDIVGRNLLYAACMAGQSDVIRA  95
            LL   +L G+ + +Q++    EDP  P+ E            G   L+ A  AG +++++
Sbjct  862  LLDSSLEGEFDLVQRVIYEVEDPSQPNDE------------GITALHNAVCAGHTEIVKF  909

Query  96   LAKYGVNLNEKTTRGYTLLHCAAAWGRLETLKALVELDVDIEALNFRE-----ERARDVA  150
            L +YGVN+N    + G+T LHCAA+    ++  K LVE    + A+ + +     ++  ++
Sbjct  910  LVQYGVNVNAADSDGWTPLHCAASCNNVQVCKFLVESGAAVFAMTYSDMQTAADKCEEME  969

Query  151  ARYSQTECVEFL  162
               Y  T+C +FL
Sbjct  970  EGY--TQCSQFL  979
```

>**ref|XP_001918932.1|** PREDICTED: hypothetical protein [Danio rerio]
Length=1058

**GENE ID: 791864 LOC791864** | hypothetical LOC791864 [Danio rerio]

```
 Score = 61.6 bits (148),  Expect = 7e-08, Method: Compositional matrix adjust.
 Identities = 38/132 (28%), Positives = 68/132 (51%), Gaps = 22/132 (16%)

Query  39   LLQPALTGDVEGLQKIF---EDPENPHHEQAMQLLLEEDIVGRNLLYAACMAGQSDVIRA  95
            LL  +L G+ + +Q++    EDP  P+ E             G   L+ A  AG +++++
Sbjct  860  LLDSSLEGEFDLVQRVIYEVEDPSQPNDE------------GITALHNAVCAGHTEIVKF  907

Query  96   LAKYGVNLNEKTTRGYTLLHCAAAWGRLETLKALVELDVDIEALNFRE-----ERARDVA  150
            L +YGVN+N   + G+T LHCAA+   ++  K LVE    + A+ + +     ++  ++
Sbjct  908  LVQYGVNVNAADSDGWTPLHCAASCNNVQVCKFLVESGAAVFAMTYSDMQTAADKCEEME  967

Query  151  ARYSQTECVEFL  162
              Y+Q  C +FL
Sbjct  968  EGYTQ--CSQFL  977
```

>**gb|ABA54552.1|** 70 kDa heat shock protein [Mytilus edulis]
Length=86

```
 Score = 61.6 bits (148),  Expect = 7e-08, Method: Compositional matrix adjust.
 Identities = 29/85 (34%), Positives = 49/85 (57%), Gaps = 0/85 (0%)

Query  166  DARLTLKKYIAKVSLAVTDTEKGSGKLLKEDKNTILSACRAKNEWLETHTEASINELFEQ  225
            +A++ L+ +I      + D EK  G+L K+DKN I + C+ K EW+E  T+A+  +   Q
Sbjct  1    EAKVDLQNFIKTTQETLADGEKVQGRLTKDDKNIITNTCKEKAEWVERTTDATTKDFITQ  60

Query  226  RQQLEDIVTPIFTKMTTPCQVKSAK  250
            +  LE+++ PI  K+  P   +S K
Sbjct  61   KMALEEVINPIMQKLNEPPTERSEK  85
```

>**ref|XP_001368866.1|** PREDICTED: similar to hCG2042411 [Monodelphis domestica]
Length=225

**GENE ID: 100014587 LOC100014587** | similar to hCG2042411 [Monodelphis domestica]

```
 Score = 61.6 bits (148),  Expect = 8e-08, Method: Compositional matrix adjust.
 Identities = 44/140 (31%), Positives = 66/140 (47%), Gaps = 8/140 (5%)

Query  39   LLQPALTGDVEGLQKIFEDP-ENPHHEQAMQLLLEEDIVGRNLLYAACMAGQSDVIRALA  97
            L Q    GD   + +I +    NP+H+       + D   R  L+ A + G  D+ + L
Sbjct  4    LHQAVAVGDCSTVSRILKKGLYNPNHK-------DRDWNDRTPLHWAAIKGHVDLTKLLV  56

Query  98   KYGVNLNEKTTRGYTLLHCAAAWGRLETLKALVELDVDIEALNFREERARDVAARYSQTE  157
             YG      T  G+T  H AA  G LE LKAL  L   I+A +F  +  + +A  Y Q E
Sbjct  57   AYGARPCLVTDVGWTPAHFAAESGHLEVLKALHVLHAAIDAPDFFGDTPKRIAQIYGQEE  116

Query  158  CVEFLDWADARLTLKKYIAK  177
            C+EFL+ A+      + +A+
Sbjct  117  CMEFLESAEVECQAYRLMAQ  136
```

>**ref|XP_001513723.1|** PREDICTED: similar to tumor protein p53 binding protein, 2 [Ornithorhynchus anatinus]
Length=1141

**GENE ID: 100081599 LOC100081599** | similar to tumor protein p53 binding protein, 2 [Ornithorhynchus anatinus]

```
 Score = 60.8 bits (146),  Expect = 1e-07, Method: Compositional matrix adjust.
 Identities = 39/132 (29%), Positives = 68/132 (51%), Gaps = 22/132 (16%)

Query  39    LLQPALTGDVEGLQKIF---EDPENPHHEQAMQLLLEEDIVGRNLLYAACMAGQSDVIRA  95
             LL  +L G+ + +Q+I    EDP  P+ E             G   L+ A  AG +++++
Sbjct  943   LLDSSLEGEFDLVQRIIYEVEDPSLPNDE------------GITALHNAVCAGHTEIVKF  990

Query  96    LAKYGVNLNEKTTRGYTLLHCAAAWGRLETLKALVELDVDIEALNFRE-----ERARDVA  150
             L ++GVN+N   + G+T LHCAA+   ++  K LVE    + A+ + +     ++  ++
Sbjct  991   LVQFGVNVNAADSDGWTPLHCAASCNNVQVCKFLVESGAAVFAMTYSDMQTAADKCEEME  1050

Query  151   ARYSQTECVEFL  162
               YSQ  C +FL
Sbjct  1051  EGYSQ--CSQFL  1060
```

>**ref|XP_419394.2|** PREDICTED: similar to tumor protein p53 binding protein, 2 [Gallus gallus]
Length=1155

**GENE ID: 421329 TP53BP2** | tumor protein p53 binding protein, 2 [Gallus gallus]

```
 Score = 60.8 bits (146),  Expect = 1e-07, Method: Compositional matrix adjust.
 Identities = 38/132 (28%), Positives = 68/132 (51%), Gaps = 22/132 (16%)

Query  39    LLQPALTGDVEGLQKIF---EDPENPHHEQAMQLLLEEDIVGRNLLYAACMAGQSDVIRA  95
             LL  +L G+ + +Q+I    EDP  P+ E             G   L+ A  AG +++++
Sbjct  957   LLDSSLEGEFDLVQRIIYEVEDPSMPNDE------------GITALHNAVCAGHTEIVKF  1004

Query  96    LAKYGVNLNEKTTRGYTLLHCAAAWGRLETLKALVELDVDIEALNFRE-----ERARDVA  150
             L ++GVN+N   + G+T LHCAA+   ++  K LVE    + A+ + +     ++  ++
Sbjct  1005  LVQFGVNVNAADSDGWTPLHCAASCNNVQVCKFLVESGAAVFAMTYSDMQTAADKCEEME  1064

Query  151   ARYSQTECVEFL  162
               Y  T+C +FL
Sbjct  1065  EGY--TQCSQFL  1074
```

>**gb|AAH98968.1|** LOC414498 protein [Xenopus laevis]
Length=1141

**GENE ID: 414498 LOC414498** | hypothetical protein LOC414498 [Xenopus laevis]

(10 or fewer PubMed links)

```
 Score = 60.5 bits (145),  Expect = 2e-07, Method: Compositional matrix adjust.
 Identities = 39/132 (29%), Positives = 67/132 (50%), Gaps = 22/132 (16%)

Query  39    LLQPALTGDVEGLQKIF---EDPENPHHEQAMQLLLEEDIVGRNLLYAACMAGQSDVIRA  95
             LL  +L G+ + +Q+I    EDP  P+ E            G   L+ A  AG +++++
Sbjct  943   LLDSSLEGEFDLVQRIIYEVEDPSQPNDE------------GITALHNAVCAGHTEIVKF  990

Query  96    LAKYGVNLNEKTTRGYTLLHCAAAWGRLETLKALVELDVDIEALNFRE-----ERARDVA  150
             L ++GVN+N   + G+T LHCAA+   ++  K LVE    + A  + +     ++  ++
Sbjct  991   LVQFGVNVNAADSDGWTPLHCAASCNNVQVCKFLVESGAAVFATTYSDRQTAADKCEEME  1050

Query  151   ARYSQTECVEFL  162
               YSQ  C +FL
Sbjct  1051  EGYSQ--CSQFL  1060
```

>**gb|AAH68956.1|** LOC414498 protein [Xenopus laevis]
Length=1139

**GENE ID: 414498 LOC414498** | hypothetical protein LOC414498 [Xenopus laevis]
(10 or fewer PubMed links)

```
 Score = 60.5 bits (145),  Expect = 2e-07, Method: Compositional matrix adjust.
 Identities = 39/132 (29%), Positives = 67/132 (50%), Gaps = 22/132 (16%)

Query  39    LLQPALTGDVEGLQKIF---EDPENPHHEQAMQLLLEEDIVGRNLLYAACMAGQSDVIRA  95
             LL  +L G+ + +Q+I    EDP  P+ E            G   L+ A  AG +++++
Sbjct  941   LLDSSLEGEFDLVQRIIYEVEDPSQPNDE------------GITALHNAVCAGHTEIVKF  988

Query  96    LAKYGVNLNEKTTRGYTLLHCAAAWGRLETLKALVELDVDIEALNFRE-----ERARDVA  150
             L ++GVN+N   + G+T LHCAA+   ++  K LVE    + A  + +     ++  ++
Sbjct  989   LVQFGVNVNAADSDGWTPLHCAASCNNVQVCKFLVESGAAVFATTYSDRQTAADKCEEME  1048

Query  151   ARYSQTECVEFL  162
               YSQ  C +FL
Sbjct  1049  EGYSQ--CSQFL  1058
```

>**ref|NP_001087084.1|** hypothetical protein LOC446947 [Xenopus laevis]
**gb|AAI28682.1|** LOC446947 protein [Xenopus laevis]
Length=1111

**GENE ID: 446947 LOC446947** | hypothetical protein LOC446947 [Xenopus laevis]
(10 or fewer PubMed links)

```
 Score = 60.5 bits (145),  Expect = 2e-07, Method: Compositional matrix adjust.
 Identities = 39/132 (29%), Positives = 67/132 (50%), Gaps = 22/132 (16%)

Query  39    LLQPALTGDVEGLQKIF---EDPENPHHEQAMQLLLEEDIVGRNLLYAACMAGQSDVIRA  95
             LL  +L G+ + +Q+I    EDP  P+ E            G   L+ A  AG +++++
Sbjct  913   LLDSSLEGEFDLVQRIIYEVEDPSQPNDE------------GITALHNAVCAGHTEIVKF  960

Query  96    LAKYGVNLNEKTTRGYTLLHCAAAWGRLETLKALVELDVDIEALNFRE-----ERARDVA  150
             L ++GVN+N   + G+T LHCAA+   ++  K LVE    + A  + +     ++  ++
Sbjct  961   LVQFGVNVNAADSDGWTPLHCAASCNNVQVCKFLVESGAAVFATTYSDRQTAADKCEEME  1020

Query  151   ARYSQTECVEFL  162
               YSQ  C +FL
Sbjct  1021  EGYSQ--CSQFL  1030
```

>**gb|AAI71141.1|** LOC548790 protein [Xenopus tropicalis]
Length=1110

**GENE ID: 548790 TGas141e06.1** | tumor protein p53 binding protein, 2
[Xenopus (Silurana) tropicalis]

```
 Score = 60.5 bits (145),  Expect = 2e-07, Method: Compositional matrix adjust.
 Identities = 39/132 (29%), Positives = 67/132 (50%), Gaps = 22/132 (16%)

Query  39    LLQPALTGDVEGLQKIF---EDPENPHHEQAMQLLLEEDIVGRNLLYAACMAGQSDVIRA  95
             LL  +L G+ + +Q+I    EDP  P+ E            G   L+ A  AG +++++
Sbjct  912   LLDASLEGEFDLVQRIIYEVEDPSQPNDE------------GITALHNAVCAGHTEIVKF  959

Query  96    LAKYGVNLNEKTTRGYTLLHCAAAWGRLETLKALVELDVDIEALNFRE-----ERARDVA  150
             L ++GVN+N   + G+T LHCAA+   ++  K LVE    + A  + +     ++  ++
Sbjct  960   LVQFGVNVNAADSDGWTPLHCAASCNNVQVCKFLVESGAAVFATTYSDRQTAADKCEEME  1019

Query  151   ARYSQTECVEFL  162
               YSQ  C +FL
Sbjct  1020  EGYSQ--CSQFL  1029
```

>**ref|XP_002591229.1|** hypothetical protein BRAFLDRAFT_131415 [Branchiostoma floridae]
**gb|EEN47240.1|** hypothetical protein BRAFLDRAFT_131415 [Branchiostoma floridae]
Length=473

**GENE ID: 7225994 BRAFLDRAFT_131415** | hypothetical protein
[Branchiostoma floridae] (10 or fewer PubMed links)

```
 Score = 60.5 bits (145),  Expect = 2e-07, Method: Compositional matrix adjust.
 Identities = 50/155 (32%), Positives = 71/155 (45%), Gaps = 9/155 (5%)

Query  31    EETGPKNPLLQPALTGDVEGLQKIFEDPENPHHEQAMQLLLEE------DIVGRNLLYAA  84
             E  G   PL+   L   +E LQ         H+E    LL         D  G  LL+ A
Sbjct  155   ENFGFCQPLMDKHL---LEALQHSSVSTGGDHYEACAVLLAGGADIGVVDHYGCTLLHYA  211

Query  85    CMAGQSDVIRALAKYGVNLNEKTTRGYTLLHCAAAWGRLETLKALVELDVDIEALNFREE  144
              + G +D I  L   G +L+ + T G+ LLH AA  G  +T+  L++ DVDIEA +   +
Sbjct  212   ALKGNNDAILLLLDRGADLDARNTYGHFLLHSAALGGHNDTINLLLDRDVDIEAEDIGGK  271

Query  145   RARDVAARYSQTECVEFLDWADARLTLKKYIAKVS  179
              A   AA+Y   E V+ L      LT K   A+++
```

```
Sbjct  272  TALHFAAQYGHHETVQLLCSRGGDLTGKDEFAEMT  306
```

>**emb|CAF98676.1|** unnamed protein product [Tetraodon nigroviridis]
Length=1216

```
 Score = 60.5 bits (145),  Expect = 2e-07, Method: Compositional matrix adjust.
 Identities = 38/132 (28%), Positives = 68/132 (51%), Gaps = 22/132 (16%)

Query  39    LLQPALTGDVEGLQKIF---EDPENPHHEQAMQLLLEEDIVGRNLLYAACMAGQSDVIRA  95
             LL   +L G+ + +Q+I    EDP  P+ E             G    L+ A  AG +++++
Sbjct  943   LLDSSLEGEFDLVQRIIYEVEDPSQPNDE------------GITALHNAVCAGHTEIVKF  990

Query  96    LAKYGVNLNEKTTRGYTLLHCAAAWGRLETLKALVELDVDIEALNFRE-----ERARDVA  150
             L ++GVN+N    + G+T LHCAA+    ++  K LVE     + A+ + +      ++  ++
Sbjct  991   LVQFGVNVNAADSDGWTPLHCAASCNNVQVCKFLVESGAAVFAMTYSDMQTAADKCEEME  1050

Query  151   ARYSQTECVEFL  162
                 Y  T+C +FL
Sbjct  1051  EGY--TQCSQFL  1060
```

>**ref|NP_001016036.2|** hypothetical protein LOC548790 [Xenopus (Silurana) tropicalis]
 **emb|CAJ83754.1|** tumor protein p53 binding protein, 2 [Xenopus tropicalis]
Length=1101

 **GENE ID: 548790 TGas141e06.1** | tumor protein p53 binding protein, 2
[Xenopus (Silurana) tropicalis]

```
 Score = 60.5 bits (145),  Expect = 2e-07, Method: Compositional matrix adjust.
 Identities = 39/132 (29%), Positives = 67/132 (50%), Gaps = 22/132 (16%)

Query  39    LLQPALTGDVEGLQKIF---EDPENPHHEQAMQLLLEEDIVGRNLLYAACMAGQSDVIRA  95
             LL   +L G+ + +Q+I    EDP  P+ E             G    L+ A  AG +++++
Sbjct  903   LLDASLEGEFDLVQRIIYEVEDPSQPNDE------------GITALHNAVCAGHTEIVKF  950

Query  96    LAKYGVNLNEKTTRGYTLLHCAAAWGRLETLKALVELDVDIEALNFRE-----ERARDVA  150
             L ++GVN+N    + G+T LHCAA+    ++  K LVE     + A   + +      ++  ++
Sbjct  951   LVQFGVNVNAADSDGWTPLHCAASCNNVQVCKFLVESGAAVFATTYSDRQTAADKCEEME  1010

Query  151   ARYSQTECVEFL  162
               YSQ  C +FL
Sbjct  1011  EGYSQ--CSQFL  1020
```

>**ref|XP_002196970.1|** PREDICTED: tumor protein p53 binding protein, 2 [Taeniopygia
guttata]
Length=1149

 **GENE ID: 100221962 LOC100221962** | similar to tumor protein p53 binding protein,
2 [Taeniopygia guttata]

```
 Score = 60.5 bits (145),  Expect = 2e-07, Method: Compositional matrix adjust.
 Identities = 38/132 (28%), Positives = 68/132 (51%), Gaps = 22/132 (16%)

Query  39    LLQPALTGDVEGLQKIF---EDPENPHHEQAMQLLLEEDIVGRNLLYAACMAGQSDVIRA  95
             LL   +L G+ + +Q+I    EDP  P+ E             G    L+ A  AG +++++
Sbjct  951   LLDSSLEGEFDLVQRIIYEVEDPSMPNDE------------GITALHNAVCAGHTEIVKF  998

Query  96    LAKYGVNLNEKTTRGYTLLHCAAAWGRLETLKALVELDVDIEALNFRE-----ERARDVA  150
             L ++GVN+N    + G+T LHCAA+    ++  K LVE     + A+ + +      ++  ++
Sbjct  999   LVQFGVNVNAADSDGWTPLHCAASCNNVQVCKFLVESGAAVFAMTYSDMQTAADKCEEME  1058

Query  151   ARYSQTECVEFL  162
                 Y  T+C +FL
Sbjct  1059  EGY--TQCSQFL  1068
```

>**ref|XP_002407854.1|** conserved hypothetical protein [Ixodes scapularis]
 **gb|EEC10560.1|** conserved hypothetical protein [Ixodes scapularis]
Length=918

 **GENE ID: 8031915 IscW_ISCW007890** | hypothetical protein [Ixodes scapularis]

```
 Score = 60.5 bits (145),  Expect = 2e-07, Method: Compositional matrix adjust.
 Identities = 34/89 (38%), Positives = 46/89 (51%), Gaps = 4/89 (4%)

Query  78   RNLLYAACMAGQSDVIRALAKY----GVNLNEKTTRGYTLLHCAAAWGRLETLKALVELD  133
            R LL+ A   GQ  V+R LA+Y      +N+N K   G T +H AA  G +  +K LV LD
Sbjct  110  RTLLHLAAKYGQERVVRWLAEYMQNNNLNINRKDNDGNTPVHLAAKHGHISVIKTLVLLD  169

Query  134  VDIEALNFREERARDVAARYSQTECVEFL  162
              D+ A N +   R   VA +  Q  C + L
Sbjct  170  ADVTAQNEQGLRPHGVAVQSGQVACADHL  198
```

>**dbj|BAE90962.1|** unnamed protein product [Macaca fascicularis]
Length=517

```
 Score = 60.5 bits (145),  Expect = 2e-07, Method: Compositional matrix adjust.
 Identities = 38/132 (28%), Positives = 68/132 (51%), Gaps = 22/132 (16%)

Query  39   LLQPALTGDVEGLQKIF---EDPENPHHEQAMQLLLEEDIVGRNLLYAACMAGQSDVIRA  95
            LL   +L G+ + +Q+I    +DP  P+ E             G    L+ A  AG ++++R
Sbjct  319  LLDSSLEGEFDLVQRIIYEVDDPSLPNDE------------GITALHNAVCAGHTEIVRF  366

Query  96   LAKYGVNLNEKTTRGYTLLHCAAAWGRLETLKALVELDVDIEALNFRE-----ERARDVA  150
            L ++GVN+N    + G+T LHCAA+    ++  K LVE     + A+ + +      ++  ++
Sbjct  367  LVQFGVNVNAADSDGWTPLHCAASCNNVQVCKFLVESGAAVFAMTYSDMQTAADKCEEME  426

Query  151  ARYSQTECVEFL  162
                Y  T+C +FL
Sbjct  427  EGY--TQCSQFL  436
```

>**ref|XP_001897569.1|** Ankyrin repeat containing protein [Brugia malayi]
**gb|EDP33453.1|** Ankyrin repeat containing protein, putative [Brugia malayi]
Length=264

**GENE ID: 6101022 Bm1 30650** | Ankyrin repeat containing protein [Brugia malayi]
(10 or fewer PubMed links)

```
 Score = 59.7 bits (143),  Expect = 3e-07, Method: Compositional matrix adjust.
 Identities = 34/104 (32%), Positives = 52/104 (50%), Gaps = 8/104 (7%)

Query  35   PKNPLLQPALTGDVEGLQKIFEDPENPHHEQAMQLLLEEDIVGRNLLYAACMAGQSDVIR  94
            PK  +L  A  G++E L+ + E+  NP       LL   D+ G   L+ A  +G +D++
Sbjct  100  PKEQVLTAAEDGNLESLKDLIEN--NP------SLLSARDVDGYTALHRAAYSGHTDIVG  151

Query  95   ALAKYGVNLNEKTTRGYTLLHCAAAWGRLETLKALVELDVDIEA  138
             L   G N    T  G+T+LHCAA W   E +  L+    VD+ +
Sbjct  152  YLLSIGANPEWNTNDGWTVLHCAATWSMCEVVALLLRHGVDVNS  195
```

>**gb|AAI42600.1|** Si:dkeyp-34c12.2 protein [Danio rerio]
Length=996

**GENE ID: 568138 si:dkeyp-34c12.2** | si:dkeyp-34c12.2 [Danio rerio]
(10 or fewer PubMed links)

```
 Score = 59.7 bits (143),  Expect = 3e-07, Method: Compositional matrix adjust.
 Identities = 38/132 (28%), Positives = 68/132 (51%), Gaps = 22/132 (16%)

Query  39   LLQPALTGDVEGLQKIF---EDPENPHHEQAMQLLLEEDIVGRNLLYAACMAGQSDVIRA  95
            LL  +L G+ + +Q+I    +DP  P+ E            G   L+ A  AG +++++
Sbjct  865  LLDSSLEGEYDLVQRIIYEVDDPSLPNDE------------GITALHNAVCAGHTEIVKF  912

Query  96   LAKYGVNLNEKTTRGYTLLHCAAAWGRLETLKALVELDVDIEALNFRE-----ERARDVA  150
            L +YGVN+N   + G+T LHCAA+   ++  K LVE    + A+ + +     ++  ++
Sbjct  913  LVQYGVNVNAADSDGWTPLHCAASCNNVQVCKFLVESGAAVYAMTYSDLQTAADKCEEME  972

Query  151  ARYSQTECVEFL  162
              Y+Q  C +FL
Sbjct  973  EGYAQ--CSQFL  982
```

>**ref|NP_001038618.1|** tumor protein p53 binding protein, 2 [Danio rerio]
**emb|CAK11376.1|** novel protein similar to vertebrate tumor protein p53 binding protein, 2 (TP53BP2) [Danio rerio]
Length=1060

**GENE ID: 568138 si:dkeyp-34c12.2** | si:dkeyp-34c12.2 [Danio rerio]
(10 or fewer PubMed links)

```
 Score = 59.7 bits (143),  Expect = 3e-07, Method: Compositional matrix adjust.
 Identities = 38/132 (28%), Positives = 68/132 (51%), Gaps = 22/132 (16%)

Query  39   LLQPALTGDVEGLQKIF---EDPENPHHEQAMQLLLEEDIVGRNLLYAACMAGQSDVIRA  95
            LL  +L G+ + +Q+I    +DP  P+ E            G   L+ A  AG +++++
Sbjct  862  LLDSSLEGEYDLVQRIIYEVDDPSLPNDE------------GITALHNAVCAGHTEIVKF  909

Query  96   LAKYGVNLNEKTTRGYTLLHCAAAWGRLETLKALVELDVDIEALNFRE-----ERARDVA  150
            L +YGVN+N   + G+T LHCAA+   ++  K LVE    + A+ + +     ++  ++
Sbjct  910  LVQYGVNVNAADSDGWTPLHCAASCNNVQVCKFLVESGAAVYAMTYSDLQTAADKCEEME  969

Query  151  ARYSQTECVEFL  162
              Y+Q  C +FL
Sbjct  970  EGYAQ--CSQFL  979
```

>**ref|NP_001103240.2|** tumor protein p53 binding protein, 2-like [Danio rerio]
Length=1063

**GENE ID: 793439 CH211-151I8.3** | tumor protein p53 binding protein, 2-like [Danio rerio]

```
 Score = 59.7 bits (143),  Expect = 3e-07, Method: Compositional matrix adjust.
 Identities = 38/132 (28%), Positives = 68/132 (51%), Gaps = 22/132 (16%)

Query  39   LLQPALTGDVEGLQKIF---EDPENPHHEQAMQLLLEEDIVGRNLLYAACMAGQSDVIRA  95
            LL  +L G+ + +Q+I    +DP  P+ E            G   L+ A  AG +++++
Sbjct  865  LLDSSLEGEYDLVQRIIYEVDDPSLPNDE------------GITALHNAVCAGHTEIVKF  912

Query  96   LAKYGVNLNEKTTRGYTLLHCAAAWGRLETLKALVELDVDIEALNFRE-----ERARDVA  150
            L +YGVN+N   + G+T LHCAA+   ++  K LVE    + A+ + +     ++  ++
Sbjct  913  LVQYGVNVNAADSDGWTPLHCAASCNNVQVCKFLVESGAAVYAMTYSDLQTAADKCEEME  972

Query  151  ARYSQTECVEFL  162
              Y+Q  C +FL
Sbjct  973  EGYAQ--CSQFL  982
```

>**ref|XP_001923587.1|** PREDICTED: hypothetical protein LOC793439 [Danio rerio]
Length=1062

**GENE ID: 793439 CH211-151I8.3** | tumor protein p53 binding protein, 2-like [Danio rerio]

```
 Score = 59.7 bits (143),  Expect = 3e-07, Method: Compositional matrix adjust.
 Identities = 38/132 (28%), Positives = 68/132 (51%), Gaps = 22/132 (16%)

Query  39   LLQPALTGDVEGLQKIF---EDPENPHHEQAMQLLLEEDIVGRNLLYAACMAGQSDVIRA  95
            LL  +L G+ + +Q+I    +DP  P+ E            G   L+ A  AG +++++
Sbjct  864  LLDSSLEGEYDLVQRIIYEVDDPSLPNDE------------GITALHNAVCAGHTEIVKF  911

Query  96   LAKYGVNLNEKTTRGYTLLHCAAAWGRLETLKALVELDVDIEALNFRE-----ERARDVA  150
            L +YGVN+N   + G+T LHCAA+   ++  K LVE    + A+ + +     ++  ++
Sbjct  912  LVQYGVNVNAADSDGWTPLHCAASCNNVQVCKFLVESGAAVYAMTYSDLQTAADKCEEME  971

Query  151  ARYSQTECVEFL  162
```

```
                Y+Q  C +FL
Sbjct  972  EGYAQ--CSQFL  981


>dbj|BAG58337.1|  unnamed protein product [Homo sapiens]
Length=723

 GENE ID: 7159 TP53BP2 | tumor protein p53 binding protein, 2 [Homo sapiens]
(Over 10 PubMed links)

 Score = 59.3 bits (142),  Expect = 4e-07, Method: Compositional matrix adjust.
 Identities = 37/132 (28%), Positives = 68/132 (51%), Gaps = 22/132 (16%)

Query  39   LLQPALTGDVEGLQKIF---EDPENPHHEQAMQLLLEEDIVGRNLLYAACMAGQSDVIRA  95
            LL  +L G+ + +Q+I    +DP  P+ E             G   L+ A  AG +++++
Sbjct  557  LLDSSLEGEFDLVQRIIYEVDDPSLPNDE------------GITALHNAVCAGHTEIVKF  604

Query  96   LAKYGVNLNEKTTRGYTLLHCAAAWGRLETLKALVELDVDIEALNFRE-----ERARDVA  150
            L ++GVN+N   + G+T LHCAA+   ++   K LVE     + A+ + +      ++  ++
Sbjct  605  LVQFGVNVNAADSDGWTPLHCAASCNNVQVCKFLVESGAAVFAMTYSDMQTAADKCEEME  664

Query  151  ARYSQTECVEFL  162
              Y  T+C +FL
Sbjct  665  EGY--TQCSQFL  674


>ref|XP_863359.1|  PREDICTED: similar to 78 kDa glucose-regulated protein precursor
(GRP 78) (Immunoglobulin heavy chain binding protein) (BiP)
(Endoplasmic reticulum lumenal Ca(2+) binding protein grp78)
isoform 4 [Canis familiaris]
Length=601

 GENE ID: 480726 LOC480726 | similar to 78 kDa glucose-regulated protein
precursor (GRP 78) (Immunoglobulin heavy chain binding protein) (BiP)
(Endoplasmic reticulum lumenal Ca(2+) binding protein grp78)
[Canis lupus familiaris]

 Score = 59.3 bits (142),  Expect = 4e-07, Method: Compositional matrix adjust.
 Identities = 33/86 (38%), Positives = 48/86 (55%), Gaps = 0/86 (0%)

Query  155  QTECVEFLDWADARLTLKKYIAKVSLAVTDTEKGSGKLLKEDKNTILSACRAKNEWLETH  214
            Q E    +D  D R  L+ Y    +    + D EK  GKL  EDK T+  A    K EWLE+H
Sbjct  496  QIEVTFEIDRIDTRNELESYAYSLKNQIGDKEKLGGKLSSEDKETMEKAVEEKIEWLESH  555

Query  215  TEASINELFEQRQQLEDIVTPIFTKM  240
             +A I +    ++++LE+IV PI +K+
Sbjct  556  QDADIEDFKAKKKELEEIVQPIISKL  581


>gb|AAH98984.1|  LOC446947 protein [Xenopus laevis]
Length=1145

 GENE ID: 446947 LOC446947 | hypothetical protein LOC446947 [Xenopus laevis]
(10 or fewer PubMed links)

 Score = 59.3 bits (142),  Expect = 4e-07, Method: Compositional matrix adjust.
 Identities = 38/132 (28%), Positives = 67/132 (50%), Gaps = 22/132 (16%)

Query  39    LLQPALTGDVEGLQKIF---EDPENPHHEQAMQLLLEEDIVGRNLLYAACMAGQSDVIRA  95
             LL  +L G+ + +Q+I    +DP  P+ E             G   L+ A  AG +++++
Sbjct  947   LLDSSLEGEFDLVQRIIYEVDDPSQPNDE------------GITALHNAVCAGHTEIVKF  994

Query  96    LAKYGVNLNEKTTRGYTLLHCAAAWGRLETLKALVELDVDIEALNFRE-----ERARDVA  150
             L ++GVN+N   + G+T LHCAA+   ++   K LVE     + A   + +      ++  ++
Sbjct  995   LVQFGVNVNAADSDGWTPLHCAASCNNVQVCKFLVESGAAVFATTYSDRQTAADKCEEME  1054

Query  151   ARYSQTECVEFL  162
               YSQ  C +FL
Sbjct  1055  EGYSQ--CSQFL  1064


>gb|AAI10930.1|  LOC446947 protein [Xenopus laevis]
Length=1143

 GENE ID: 446947 LOC446947 | hypothetical protein LOC446947 [Xenopus laevis]
(10 or fewer PubMed links)

 Score = 59.3 bits (142),  Expect = 4e-07, Method: Compositional matrix adjust.
 Identities = 38/132 (28%), Positives = 67/132 (50%), Gaps = 22/132 (16%)

Query  39    LLQPALTGDVEGLQKIF---EDPENPHHEQAMQLLLEEDIVGRNLLYAACMAGQSDVIRA  95
             LL  +L G+ + +Q+I    +DP  P+ E             G   L+ A  AG +++++
Sbjct  945   LLDSSLEGEFDLVQRIIYEVDDPSQPNDE------------GITALHNAVCAGHTEIVKF  992

Query  96    LAKYGVNLNEKTTRGYTLLHCAAAWGRLETLKALVELDVDIEALNFRE-----ERARDVA  150
             L ++GVN+N   + G+T LHCAA+   ++   K LVE     + A   + +      ++  ++
Sbjct  993   LVQFGVNVNAADSDGWTPLHCAASCNNVQVCKFLVESGAAVFATTYSDRQTAADKCEEME  1052

Query  151   ARYSQTECVEFL  162
               YSQ  C +FL
Sbjct  1053  EGYSQ--CSQFL  1062


>gb|AAH81262.1|  LOC446947 protein [Xenopus laevis]
Length=1125

 GENE ID: 446947 LOC446947 | hypothetical protein LOC446947 [Xenopus laevis]
(10 or fewer PubMed links)

 Score = 59.3 bits (142),  Expect = 4e-07, Method: Compositional matrix adjust.
 Identities = 38/132 (28%), Positives = 67/132 (50%), Gaps = 22/132 (16%)

Query  39    LLQPALTGDVEGLQKIF---EDPENPHHEQAMQLLLEEDIVGRNLLYAACMAGQSDVIRA  95
             LL  +L G+ + +Q+I    +DP  P+ E             G   L+ A  AG +++++
Sbjct  927   LLDSSLEGEFDLVQRIIYEVDDPSQPNDE------------GITALHNAVCAGHTEIVKF  974
```

```
Query  96    LAKYGVNLNEKTTRGYTLLHCAAAWGRLETLKALVELDVDIEALNFRE-----ERARDVA  150
             L ++GVN+N   + G+T LHCAA+   ++  K LVE    + A  + +     ++  ++
Sbjct  975   LVQFGVNVNAADSDGWTPLHCAASCNNVQVCKFLVESGAAVFATTYSDRQTAADKCEEME  1034

Query  151   ARYSQTECVEFL  162
               YSQ  C +FL
Sbjct  1035  EGYSQ--CSQFL  1044
```

>**dbj|BAE91720.1|** unnamed protein product [Macaca fascicularis]
Length=566

```
 Score = 58.9 bits (141),  Expect = 4e-07, Method: Compositional matrix adjust.
 Identities = 37/132 (28%), Positives = 68/132 (51%), Gaps = 22/132 (16%)

Query  39   LLQPALTGDVEGLQKIF---EDPENPHHEQAMQLLLEEDIVGRNLLYAACMAGQSDVIRA  95
            LL  +L G+ + +Q+I    +DP  P+ E            G   L+ A  AG +++++
Sbjct  368  LLDSSLEGEFDLVQRIIYEVDDPSLPNDE------------GITALHNAVCAGHTEIVKF  415

Query  96   LAKYGVNLNEKTTRGYTLLHCAAAWGRLETLKALVELDVDIEALNFRE-----ERARDVA  150
            L ++GVN+N   + G+T LHCAA+   ++  K LVE    + A+ + +     ++  ++
Sbjct  416  LVQFGVNVNAADSDGWTPLHCAASCNNVQVCKFLVESGAAVFAMTYSDMQTAADKCEEME  475

Query  151  ARYSQTECVEFL  162
              Y  T+C +FL
Sbjct  476  EGY--TQCSQFL  485
```

>**ref|XP_002192655.1|** PREDICTED: heat shock 70kDa protein 5 [Taeniopygia guttata]
Length=651

**GENE ID: 100218236 LOC100218236** | heat shock 70kDa protein 5
[Taeniopygia guttata]

```
 Score = 58.9 bits (141),  Expect = 5e-07, Method: Compositional matrix adjust.
 Identities = 31/75 (41%), Positives = 44/75 (58%), Gaps = 0/75 (0%)

Query  166  DARLTLKKYIAKVSLAVTDTEKGSGKLLKEDKNTILSACRAKNEWLETHTEASINELFEQ  225
            DAR  L+ Y   +   + D EK  GKL  EDK TI  A   K EWLE+H +  I +   Q
Sbjct  558  DARNELESYAYSLKNQIGDKEKLGGKLSSEDKETIEKAVEEKIEWLESHQDGDIEDFKAQ  617

Query  226  RQQLEDIVTPIFTKM  240
            +++LE++V PI +K+
Sbjct  618  KKELEEVVQPIVSKL  632
```

>**dbj|BAH14474.1|** unnamed protein product [Homo sapiens]
Length=773

**GENE ID: 7159 TP53BP2** | tumor protein p53 binding protein, 2 [Homo sapiens]
(Over 10 PubMed links)

```
 Score = 58.9 bits (141),  Expect = 5e-07, Method: Compositional matrix adjust.
 Identities = 37/132 (28%), Positives = 68/132 (51%), Gaps = 22/132 (16%)

Query  39   LLQPALTGDVEGLQKIF---EDPENPHHEQAMQLLLEEDIVGRNLLYAACMAGQSDVIRA  95
            LL  +L G+ + +Q+I    +DP  P+ E            G   L+ A  AG +++++
Sbjct  575  LLDSSLEGEFDLVQRIIYEVDDPSLPNDE------------GITALHNAVCAGHTEIVKF  622

Query  96   LAKYGVNLNEKTTRGYTLLHCAAAWGRLETLKALVELDVDIEALNFRE-----ERARDVA  150
            L ++GVN+N   + G+T LHCAA+   ++  K LVE    + A+ + +     ++  ++
Sbjct  623  LVQFGVNVNAADSDGWTPLHCAASCNNVQVCKFLVESGAAVFAMTYSDMQTAADKCEEME  682

Query  151  ARYSQTECVEFL  162
              Y  T+C +FL
Sbjct  683  EGY--TQCSQFL  692
```

>**dbj|BAG58037.1|** unnamed protein product [Homo sapiens]
**dbj|BAH14387.1|** unnamed protein product [Homo sapiens]
Length=773

**GENE ID: 7159 TP53BP2** | tumor protein p53 binding protein, 2 [Homo sapiens]
(Over 10 PubMed links)

```
 Score = 58.9 bits (141),  Expect = 5e-07, Method: Compositional matrix adjust.
 Identities = 37/132 (28%), Positives = 68/132 (51%), Gaps = 22/132 (16%)

Query  39   LLQPALTGDVEGLQKIF---EDPENPHHEQAMQLLLEEDIVGRNLLYAACMAGQSDVIRA  95
            LL  +L G+ + +Q+I    +DP  P+ E            G   L+ A  AG +++++
Sbjct  575  LLDSSLEGEFDLVQRIIYEVDDPSLPNDE------------GITALHNAVCAGHTEIVKF  622

Query  96   LAKYGVNLNEKTTRGYTLLHCAAAWGRLETLKALVELDVDIEALNFRE-----ERARDVA  150
            L ++GVN+N   + G+T LHCAA+   ++  K LVE    + A+ + +     ++  ++
Sbjct  623  LVQFGVNVNAADSDGWTPLHCAASCNNVQVCKFLVESGAAVFAMTYSDMQTAADKCEEME  682

Query  151  ARYSQTECVEFL  162
              Y  T+C +FL
Sbjct  683  EGY--TQCSQFL  692
```

>**dbj|BAF38391.1|** heat shock protein 70kDa [Coturnix japonica]
Length=652

```
 Score = 58.9 bits (141),  Expect = 5e-07, Method: Compositional matrix adjust.
 Identities = 31/75 (41%), Positives = 45/75 (60%), Gaps = 0/75 (0%)

Query  166  DARLTLKKYIAKVSLAVTDTEKGSGKLLKEDKNTILSACRAKNEWLETHTEASINELFEQ  225
            DAR  L+ Y   +   + D EK  GKL  EDK TI  A   K EWLE+H +A I +    +
Sbjct  558  DARNELESYAYSLKNQIGDKEKLGGKLSSEDKETIEKAVEEKIEWLESHQDADIEDFKSK  617

Query  226  RQQLEDIVTPIFTKM  240
            +++LE++V PI +K+
Sbjct  618  KKELEEVVQPIVSKL  632
```

```
>ref|XP_001480035.1|  PREDICTED: similar to hCG2042411 [Mus musculus]
Length=184

 GENE ID: 100043332 Gm4368 | predicted gene 4368 [Mus musculus]

 Score = 58.9 bits (141),  Expect = 5e-07, Method: Compositional matrix adjust.
 Identities = 42/125 (33%), Positives = 61/125 (48%), Gaps = 8/125 (6%)

Query  39   LLQPALTGDVEGLQKIFEDPE-NPHHEQAMQLLLEEDIVGRNLLYAACMAGQSDVIRALA  97
            L Q    GD   ++KI +     +P+++ A       D   R  L+ A + GQ +VI  L
Sbjct  4    LHQAVAAGDCNSVKKILKKGLCDPNYKDA-------DWNDRTPLHWAAIRGQMEVIHLLI  56

Query  98   KYGVNLNEKTTRGYTLLHCAAAWGRLETLKALVELDVDIEALNFREERARDVAARYSQTE  157
            +YG      T  G+T  H AA  G L  LKAL  L   I+A +F  +  + +A  Y Q +
Sbjct  57   QYGARPCLVTDVGWTAAHFAAESGHLNVLKALHALPSAIDAADFFGDTPKRIAQIYGQKD  116

Query  158  CVEFL  162
            CV+FL
Sbjct  117  CVDFL  121


>dbj|BAD12571.1|  heat shock protein [Numida meleagris]
Length=652

 Score = 58.9 bits (141),  Expect = 5e-07, Method: Compositional matrix adjust.
 Identities = 31/75 (41%), Positives = 45/75 (60%), Gaps = 0/75 (0%)

Query  166  DARLTLKKYIAKVSLAVTDTEKGSGKLLKEDKNTILSACRAKNEWLETHTEASINELFEQ  225
            DAR  L+ Y   +   + D EK  GKL  EDK TI  A   K EWLE+H +A I +   +
Sbjct  558  DARNELESYAYSLKNQIGDKEKLGGKLSSEDKETIEKAVEEKIEWLESHQDADIEDFKSK  617

Query  226  RQQLEDIVTPIFTKM  240
            +++LE++V PI +K+
Sbjct  618  KKELEEVVQPIVSKL  632


>dbj|BAH11835.1|  unnamed protein product [Homo sapiens]
Length=367

 GENE ID: 7159 TP53BP2 | tumor protein p53 binding protein, 2 [Homo sapiens]
(Over 10 PubMed links)

 Score = 58.9 bits (141),  Expect = 5e-07, Method: Compositional matrix adjust.
 Identities = 37/132 (28%), Positives = 68/132 (51%), Gaps = 22/132 (16%)

Query  39   LLQPALTGDVEGLQKIF---EDPENPHHEQAMQLLLEEDIVGRNLLYAACMAGQSDVIRA  95
            LL  +L G+ + +Q+I    +DP  P+ E            G   L+ A  AG +++++
Sbjct  169  LLDSSLEGEFDLVQRIIYEVDDPSLPNDE------------GITALHNAVCAGHTEIVKF  216

Query  96   LAKYGVNLNEKTTRGYTLLHCAAAWGRLETLKALVELDVDIEALNFRE-----ERARDVA  150
            L ++GVN+N   + G+T LHCAA+   ++  K LVE    + A+ + +     ++  ++
Sbjct  217  LVQFGVNVNAADSDGWTPLHCAASCNNVQVCKFLVESGAAVFAMTYSDMQTAADKCEEME  276

Query  151  ARYSQTECVEFL  162
              Y+Q  C +FL
Sbjct  277  EGYTQ--CSQFL  286


>ref|NP_990822.1|  heat shock 70kDa protein 5 precursor [Gallus gallus]
 sp|Q90593.1|GRP78_CHICK  RecName: Full=78 kDa glucose-regulated protein; AltName: Full=GRP
78; AltName: Full=Heat shock 70 kDa protein 5; AltName:
Full=Immunoglobulin heavy chain-binding protein; Short=BiP;
Flags: Precursor
 gb|AAA48785.1|  78-kD glucose-regulated protein precursor
 dbj|BAF37040.1|  heat shock protein 70kDa [Coturnix japonica]
Length=652

 GENE ID: 396487 HSPA5 | heat shock 70kDa protein 5 (glucose-regulated protein,
78kDa) [Gallus gallus] (10 or fewer PubMed links)

 Score = 58.9 bits (141),  Expect = 5e-07, Method: Compositional matrix adjust.
 Identities = 31/75 (41%), Positives = 45/75 (60%), Gaps = 0/75 (0%)

Query  166  DARLTLKKYIAKVSLAVTDTEKGSGKLLKEDKNTILSACRAKNEWLETHTEASINELFEQ  225
            DAR  L+ Y   +   + D EK  GKL  EDK TI  A   K EWLE+H +A I +   +
Sbjct  558  DARNELESYAYSLKNQIGDKEKLGGKLSSEDKETIEKAVEEKIEWLESHQDADIEDFKSK  617

Query  226  RQQLEDIVTPIFTKM  240
            +++LE++V PI +K+
Sbjct  618  KKELEEVVQPIVSKL  632


>gb|AAH30894.1|  Trp53bp2 protein [Mus musculus]
Length=762

 GENE ID: 209456 Trp53bp2 | transformation related protein 53 binding protein 2
[Mus musculus] (Over 10 PubMed links)

 Score = 58.9 bits (141),  Expect = 5e-07, Method: Compositional matrix adjust.
 Identities = 37/132 (28%), Positives = 68/132 (51%), Gaps = 22/132 (16%)

Query  39   LLQPALTGDVEGLQKIF---EDPENPHHEQAMQLLLEEDIVGRNLLYAACMAGQSDVIRA  95
            LL  +L G+ + +Q+I    +DP  P+ E            G   L+ A  AG +++++
Sbjct  564  LLDSSLEGEFDLVQRIIYEVDDPSLPNDE------------GITALHNAVCAGHTEIVKF  611

Query  96   LAKYGVNLNEKTTRGYTLLHCAAAWGRLETLKALVELDVDIEALNFRE-----ERARDVA  150
            L ++GVN+N   + G+T LHCAA+   ++  K LVE    + A+ + +     ++  ++
Sbjct  612  LVQFGVNVNAADSDGWTPLHCAASCNNVQVCKFLVESGAAVFAMTYSDMQTAADKCEEME  671

Query  151  ARYSQTECVEFL  162
              Y  T+C +FL
Sbjct  672  EGY--TQCSQFL  681


>gb|EDL13107.1|  transformation related protein 53 binding protein 2 [Mus musculus]
```

```
Length=1117

 GENE ID: 209456 Trp53bp2 | transformation related protein 53 binding protein 2
[Mus musculus] (Over 10 PubMed links)

 Score = 58.9 bits (141),  Expect = 6e-07, Method: Compositional matrix adjust.
 Identities = 37/132 (28%), Positives = 68/132 (51%), Gaps = 22/132 (16%)

Query  39    LLQPALTGDVEGLQKIF---EDPENPHHEQAMQLLLEEDIVGRNLLYAACMAGQSDVIRA  95
             LL  +L G+ + +Q+I    +DP  P+ E            G   L+ A  AG +++++
Sbjct  927   LLDSSLEGEFDLVQRIIYEVDDPSLPNDE------------GITALHNAVCAGHTEIVKF  974

Query  96    LAKYGVNLNEKTTRGYTLLHCAAAWGRLETLKALVELDVDIEALNFRE-----ERARDVA  150
             L ++GVN+N   + G+T LHCAA+   ++  K LVE    + A+ + +     ++  ++
Sbjct  975   LVQFGVNVNAADSDGWTPLHCAASCNNVQVCKFLVESGAAVFAMTYSDMQTAADKCEEME  1034

Query  151   ARYSQTECVEFL  162
               Y  T+C +FL
Sbjct  1035  EGY--TQCSQFL  1044
```

**>gb|AAA21597.1|** p53-binding protein
Length=529

```
 GENE ID: 7159 TP53BP2 | tumor protein p53 binding protein, 2 [Homo sapiens]
(Over 10 PubMed links)

 Score = 58.9 bits (141),  Expect = 6e-07, Method: Compositional matrix adjust.
 Identities = 37/132 (28%), Positives = 68/132 (51%), Gaps = 22/132 (16%)

Query  39   LLQPALTGDVEGLQKIF---EDPENPHHEQAMQLLLEEDIVGRNLLYAACMAGQSDVIRA  95
            LL  +L G+ + +Q+I    +DP  P+ E            G   L+ A  AG +++++
Sbjct  331  LLDSSLEGEFDLVQRIIYEVDDPSLPNDE------------GITALHNAVCAGHTEIVKF  378

Query  96   LAKYGVNLNEKTTRGYTLLHCAAAWGRLETLKALVELDVDIEALNFRE-----ERARDVA  150
            L ++GVN+N   + G+T LHCAA+   ++  K LVE    + A+ + +     ++  ++
Sbjct  379  LVQFGVNVNAADSDGWTPLHCAASCNNVQVCKFLVESGAAVFAMTYSDMQTAADKCEEME  438

Query  151  ARYSQTECVEFL  162
              Y  T+C +FL
Sbjct  439  EGY--TQCSQFL  448
```

**>gb|EAW93247.1|** tumor protein p53 binding protein, 2, isoform CRA_a [Homo sapiens]
Length=1125

```
 GENE ID: 7159 TP53BP2 | tumor protein p53 binding protein, 2 [Homo sapiens]
(Over 10 PubMed links)

 Score = 58.9 bits (141),  Expect = 6e-07, Method: Compositional matrix adjust.
 Identities = 37/132 (28%), Positives = 68/132 (51%), Gaps = 22/132 (16%)

Query  39    LLQPALTGDVEGLQKIF---EDPENPHHEQAMQLLLEEDIVGRNLLYAACMAGQSDVIRA  95
             LL  +L G+ + +Q+I    +DP  P+ E            G   L+ A  AG +++++
Sbjct  927   LLDSSLEGEFDLVQRIIYEVDDPSLPNDE------------GITALHNAVCAGHTEIVKF  974

Query  96    LAKYGVNLNEKTTRGYTLLHCAAAWGRLETLKALVELDVDIEALNFRE-----ERARDVA  150
             L ++GVN+N   + G+T LHCAA+   ++  K LVE    + A+ + +     ++  ++
Sbjct  975   LVQFGVNVNAADSDGWTPLHCAASCNNVQVCKFLVESGAAVFAMTYSDMQTAADKCEEME  1034

Query  151   ARYSQTECVEFL  162
               Y  T+C +FL
Sbjct  1035  EGY--TQCSQFL  1044
```

**>dbj|BAE22185.1|** unnamed protein product [Mus musculus]
Length=859

```
 GENE ID: 209456 Trp53bp2 | transformation related protein 53 binding protein 2
[Mus musculus] (Over 10 PubMed links)

 Score = 58.5 bits (140),  Expect = 6e-07, Method: Compositional matrix adjust.
 Identities = 37/132 (28%), Positives = 68/132 (51%), Gaps = 22/132 (16%)

Query  39   LLQPALTGDVEGLQKIF---EDPENPHHEQAMQLLLEEDIVGRNLLYAACMAGQSDVIRA  95
            LL  +L G+ + +Q+I    +DP  P+ E            G   L+ A  AG +++++
Sbjct  661  LLDSSLEGEFDLVQRIIYEVDDPSLPNDE------------GITALHNAVCAGHTEIVKF  708

Query  96   LAKYGVNLNEKTTRGYTLLHCAAAWGRLETLKALVELDVDIEALNFRE-----ERARDVA  150
            L ++GVN+N   + G+T LHCAA+   ++  K LVE    + A+ + +     ++  ++
Sbjct  709  LVQFGVNVNAADSDGWTPLHCAASCNNVQVCKFLVESGAAVFAMTYSDMQTAADKCEEME  768

Query  151  ARYSQTECVEFL  162
              Y  T+C +FL
Sbjct  769  EGY--TQCSQFL  778
```

**>ref|NP_001026855.2|** tumor protein p53 binding protein, 2 isoform 1 [Homo sapiens]
**dbj|BAG10743.1|** tumor protein p53 binding protein, 2 [synthetic construct]
**dbj|BAG57677.1|** unnamed protein product [Homo sapiens]
**gb|AAH58918.2|** TP53BP2 protein [Homo sapiens]
Length=1134

```
 GENE ID: 7159 TP53BP2 | tumor protein p53 binding protein, 2 [Homo sapiens]
(Over 10 PubMed links)

 Score = 58.5 bits (140),  Expect = 6e-07, Method: Compositional matrix adjust.
 Identities = 37/132 (28%), Positives = 68/132 (51%), Gaps = 22/132 (16%)

Query  39    LLQPALTGDVEGLQKIF---EDPENPHHEQAMQLLLEEDIVGRNLLYAACMAGQSDVIRA  95
             LL  +L G+ + +Q+I    +DP  P+ E            G   L+ A  AG +++++
Sbjct  936   LLDSSLEGEFDLVQRIIYEVDDPSLPNDE------------GITALHNAVCAGHTEIVKF  983

Query  96    LAKYGVNLNEKTTRGYTLLHCAAAWGRLETLKALVELDVDIEALNFRE-----ERARDVA  150
             L ++GVN+N   + G+T LHCAA+   ++  K LVE    + A+ + +     ++  ++
```

```
Sbjct  984   LVQFGVNVNAADSDGWTPLHCAASCNNVQVCKFLVESGAAVFAMTYSDMQTAADKCEEME  1043

Query  151   ARYSQTECVEFL  162
               Y  T+C +FL
Sbjct  1044  EGY--TQCSQFL  1053


>ref|NP_005417.1| UG tumor protein p53 binding protein, 2 isoform 2 [Homo sapiens]
 gb|AAC50557.1| G Bbp/53BP2
Length=1005

 GENE ID: 7159 TP53BP2 | tumor protein p53 binding protein, 2 [Homo sapiens]
(Over 10 PubMed links)

 Score = 58.5 bits (140),  Expect = 6e-07, Method: Compositional matrix adjust.
 Identities = 37/132 (28%), Positives = 68/132 (51%), Gaps = 22/132 (16%)

Query  39   LLQPALTGDVEGLQKIF---EDPENPHHEQAMQLLLEEDIVGRNLLYAACMAGQSDVIRA  95
            LL  +L G+ + +Q+I    +DP  P+ E            G   L+ A  AG +++++
Sbjct  807  LLDSSLEGEFDLVQRIIYEVDDPSLPNDE------------GITALHNAVCAGHTEIVKF  854

Query  96   LAKYGVNLNEKTTRGYTLLHCAAAWGRLETLKALVELDVDIEALNFRE-----ERARDVA  150
            L ++GVN+N   + G+T LHCAA+   ++  K LVE    + A+ + +     ++  ++
Sbjct  855  LVQFGVNVNAADSDGWTPLHCAASCNNVQVCKFLVESGAAVFAMTYSDMQTAADKCEEME  914

Query  151  ARYSQTECVEFL  162
              Y  T+C +FL
Sbjct  915  EGY--TQCSQFL  924


>ref|XP_001511765.1| UG PREDICTED: similar to hCG2042411 [Ornithorhynchus anatinus]
Length=184

 GENE ID: 100080905 LOC100080905 | similar to hCG2042411
[Ornithorhynchus anatinus]

 Score = 58.5 bits (140),  Expect = 6e-07, Method: Compositional matrix adjust.
 Identities = 39/123 (31%), Positives = 64/123 (52%), Gaps = 8/123 (6%)

Query  46   GDVEGLQKIFEDPE-NPHHEQAMQLLLEEDIVGRNLLYAACMAGQSDVIRALAKYGVNLN  104
            GD + ++KI +    NP+++         D   R  L+ A + GQ+++++ L  YG
Sbjct  14   GDYDLVEKILKKGSCNPNYKDV-------DWNDRTPLHWAAIKGQTEMVKLLIDYGARPC  66

Query  105  EKTTRGYTLLHCAAAWGRLETLKALVELDVDIEALNFREERARDVAARYSQTECVEFLDW  164
              T  G+T  H AA  GRL  L+ L  L   I+A +F  +  + +A  Y Q +CV+FL+
Sbjct  67   LTTDVGWTAAHFAAESGRLGVLRTLHALHAAIDAPDFFGDTPKRIAQIYGQKDCVKFLET  126

Query  165  ADA  167
            A+A
Sbjct  127  AEA  129


>ref|XP_576520.1| G PREDICTED: similar to ankyrin repeat domain 42 [Rattus norvegicus]
 ref|XP_001068682.1| UG PREDICTED: similar to ankyrin repeat domain 42 [Rattus norvegicus]
Length=184

 GENE ID: 501105 RGD1560151 | similar to predicted CDS, mechanosensory
transduction channel NOMPC (1O503) [Rattus norvegicus]

 Score = 58.5 bits (140),  Expect = 6e-07, Method: Compositional matrix adjust.
 Identities = 41/126 (32%), Positives = 61/126 (48%), Gaps = 8/126 (6%)

Query  39   LLQPALTGDVEGLQKIFEDPE-NPHHEQAMQLLLEEDIVGRNLLYAACMAGQSDVIRALA  97
            L Q    GD   ++KI +    +P+++       + D   R  L+ A + GQ +VI  L
Sbjct  4    LHQAVAAGDYNSVKKILKKGLCDPNYK-------DVDWNDRTPLHWAAIRGQMEVIHLLI  56

Query  98   KYGVNLNEKTTRGYTLLHCAAAWGRLETLKALVELDVDIEALNFREERARDVAARYSQTE  157
            +YG      T  G+T  H AA  G L  LK L  L   I+A +F  +  + +A  Y Q +
Sbjct  57   QYGARPCLVTDVGWTAAHFAAESGHLNVLKTLHALHAAIDAADFFGDTPKRIAQIYGQKD  116

Query  158  CVEFLD  163
            CV+FLD
Sbjct  117  CVDFLD  122


>sp|Q13625.2|ASPP2_HUMAN G RecName: Full=Apoptosis-stimulating of p53 protein 2; AltName:
Full=Tumor suppressor p53-binding protein 2; Short=p53-binding
protein 2; Short=p53BP2; Short=53BP2; AltName: Full=Bcl2-binding
protein; Short=Bbp; AltName: Full=Renal carcinoma
antigen NY-REN-51
 emb|CAC83012.1| G ASPP2 protein [Homo sapiens]
Length=1128

 GENE ID: 7159 TP53BP2 | tumor protein p53 binding protein, 2 [Homo sapiens]
(Over 10 PubMed links)

 Score = 58.5 bits (140),  Expect = 6e-07, Method: Compositional matrix adjust.
 Identities = 37/132 (28%), Positives = 68/132 (51%), Gaps = 22/132 (16%)

Query  39    LLQPALTGDVEGLQKIF---EDPENPHHEQAMQLLLEEDIVGRNLLYAACMAGQSDVIRA  95
             LL  +L G+ + +Q+I    +DP  P+ E            G   L+ A  AG +++++
Sbjct  930   LLDSSLEGEFDLVQRIIYEVDDPSLPNDE------------GITALHNAVCAGHTEIVKF  977

Query  96    LAKYGVNLNEKTTRGYTLLHCAAAWGRLETLKALVELDVDIEALNFRE-----ERARDVA  150
             L ++GVN+N   + G+T LHCAA+   ++  K LVE    + A+ + +     ++  ++
Sbjct  978   LVQFGVNVNAADSDGWTPLHCAASCNNVQVCKFLVESGAAVFAMTYSDMQTAADKCEEME  1037

Query  151   ARYSQTECVEFL  162
               Y  T+C +FL
Sbjct  1038  EGY--TQCSQFL  1047


>ref|XP_223012.4| UG PREDICTED: similar to Apoptosis-stimulating of p53 protein 2
(Tumor suppressor p53-binding protein 2) [Rattus norvegicus]
```

```
Length=1159

 GENE ID: 305025 Tp53bp2 | tumor protein p53 binding protein, 2
[Rattus norvegicus]

 Score = 58.5 bits (140),  Expect = 6e-07, Method: Compositional matrix adjust.
 Identities = 37/132 (28%), Positives = 68/132 (51%), Gaps = 22/132 (16%)

Query  39    LLQPALTGDVEGLQKIF---EDPENPHHEQAMQLLLEEDIVGRNLLYAACMAGQSDVIRA  95
             LL  +L G+ + +Q+I    +DP  P+ E            G    L+ A  AG +++++
Sbjct  946   LLDSSLEGEFDLVQRIIYEVDDPSLPNDE------------GITALHNAVCAGHTEIVKF  993

Query  96    LAKYGVNLNEKTTRGYTLLHCAAAWGRLETLKALVELDVDIEALNFRE-----ERARDVA  150
             L ++GVN+N   + G+T LHCAA+   ++  K LVE    + A+ + +      ++  ++
Sbjct  994   LVQFGVNVNAADSDGWTPLHCAASCNNVQVCKFLVESGAAVFAMTYSDMQTAADKCEEME  1053

Query  151   ARYSQTECVEFL  162
               Y  T+C +FL
Sbjct  1054  EGY--TQCSQFL  1063


>ref|XP_001063503.1|  PREDICTED: similar to Apoptosis-stimulating of p53 protein 2
(Tumor suppressor p53-binding protein 2) [Rattus norvegicus]
Length=1129

 GENE ID: 305025 Tp53bp2 | tumor protein p53 binding protein, 2
[Rattus norvegicus]

 Score = 58.5 bits (140),  Expect = 7e-07, Method: Compositional matrix adjust.
 Identities = 37/132 (28%), Positives = 68/132 (51%), Gaps = 22/132 (16%)

Query  39    LLQPALTGDVEGLQKIF---EDPENPHHEQAMQLLLEEDIVGRNLLYAACMAGQSDVIRA  95
             LL  +L G+ + +Q+I    +DP  P+ E            G    L+ A  AG +++++
Sbjct  916   LLDSSLEGEFDLVQRIIYEVDDPSLPNDE------------GITALHNAVCAGHTEIVKF  963

Query  96    LAKYGVNLNEKTTRGYTLLHCAAAWGRLETLKALVELDVDIEALNFRE-----ERARDVA  150
             L ++GVN+N   + G+T LHCAA+   ++  K LVE    + A+ + +      ++  ++
Sbjct  964   LVQFGVNVNAADSDGWTPLHCAASCNNVQVCKFLVESGAAVFAMTYSDMQTAADKCEEME  1023

Query  151   ARYSQTECVEFL  162
               Y  T+C +FL
Sbjct  1024  EGY--TQCSQFL  1033


>gb|EDL94874.1|  rCG20309 [Rattus norvegicus]
Length=1119

 Score = 58.5 bits (140),  Expect = 7e-07, Method: Compositional matrix adjust.
 Identities = 37/132 (28%), Positives = 68/132 (51%), Gaps = 22/132 (16%)

Query  39    LLQPALTGDVEGLQKIF---EDPENPHHEQAMQLLLEEDIVGRNLLYAACMAGQSDVIRA  95
             LL  +L G+ + +Q+I    +DP  P+ E            G    L+ A  AG +++++
Sbjct  921   LLDSSLEGEFDLVQRIIYEVDDPSLPNDE------------GITALHNAVCAGHTEIVKF  968

Query  96    LAKYGVNLNEKTTRGYTLLHCAAAWGRLETLKALVELDVDIEALNFRE-----ERARDVA  150
             L ++GVN+N   + G+T LHCAA+   ++  K LVE    + A+ + +      ++  ++
Sbjct  969   LVQFGVNVNAADSDGWTPLHCAASCNNVQVCKFLVESGAAVFAMTYSDMQTAADKCEEME  1028

Query  151   ARYSQTECVEFL  162
               Y  T+C +FL
Sbjct  1029  EGY--TQCSQFL  1038


>gb|AAH40247.1|  TP53BP2 protein [Homo sapiens]
Length=1048

 GENE ID: 7159 TP53BP2 | tumor protein p53 binding protein, 2 [Homo sapiens]
(Over 10 PubMed links)

 Score = 58.5 bits (140),  Expect = 7e-07, Method: Compositional matrix adjust.
 Identities = 37/132 (28%), Positives = 68/132 (51%), Gaps = 22/132 (16%)

Query  39   LLQPALTGDVEGLQKIF---EDPENPHHEQAMQLLLEEDIVGRNLLYAACMAGQSDVIRA  95
            LL  +L G+ + +Q+I    +DP  P+ E            G    L+ A  AG +++++
Sbjct  850  LLDSSLEGEFDLVQRIIYEVDDPSLPNDE------------GITALHNAVCAGHTEIVKF  897

Query  96   LAKYGVNLNEKTTRGYTLLHCAAAWGRLETLKALVELDVDIEALNFRE-----ERARDVA  150
            L ++GVN+N   + G+T LHCAA+   ++  K LVE    + A+ + +      ++  ++
Sbjct  898  LVQFGVNVNAADSDGWTPLHCAASCNNVQVCKFLVESGAAVFAMTYSDMQTAADKCEEME  957

Query  151  ARYSQTECVEFL  162
              Y  T+C +FL
Sbjct  958  EGY--TQCSQFL  967


>sp|Q8CG79.3|ASPP2_MOUSE  RecName: Full=Apoptosis-stimulating of p53 protein 2; AltName:
Full=Tumor suppressor p53-binding protein 2; Short=p53-binding
protein 2; Short=p53BP2; Short=53BP2
Length=1128

 GENE ID: 209456 Trp53bp2 | transformation related protein 53 binding protein 2
[Mus musculus] (Over 10 PubMed links)

 Score = 58.5 bits (140),  Expect = 7e-07, Method: Compositional matrix adjust.
 Identities = 37/132 (28%), Positives = 68/132 (51%), Gaps = 22/132 (16%)

Query  39    LLQPALTGDVEGLQKIF---EDPENPHHEQAMQLLLEEDIVGRNLLYAACMAGQSDVIRA  95
             LL  +L G+ + +Q+I    +DP  P+ E            G    L+ A  AG +++++
Sbjct  930   LLDSSLEGEFDLVQRIIYEVDDPSLPNDE------------GITALHNAVCAGHTEIVKF  977

Query  96    LAKYGVNLNEKTTRGYTLLHCAAAWGRLETLKALVELDVDIEALNFRE-----ERARDVA  150
             L ++GVN+N   + G+T LHCAA+   ++  K LVE    + A+ + +      ++  ++
Sbjct  978   LVQFGVNVNAADSDGWTPLHCAASCNNVQVCKFLVESGAAVFAMTYSDMQTAADKCEEME  1037

Query  151   ARYSQTECVEFL  162
               Y  T+C +FL
Sbjct  1038  EGY--TQCSQFL  1047
```

```
>gb|EAW93248.1| tumor protein p53 binding protein, 2, isoform CRA_b [Homo sapiens]
Length=1067

 GENE ID: 7159 TP53BP2 | tumor protein p53 binding protein, 2 [Homo sapiens]
(Over 10 PubMed links)

 Score = 58.5 bits (140),  Expect = 7e-07, Method: Compositional matrix adjust.
 Identities = 37/132 (28%), Positives = 68/132 (51%), Gaps = 22/132 (16%)

Query  39   LLQPALTGDVEGLQKIF---EDPENPHHEQAMQLLLEEDIVGRNLLYAACMAGQSDVIRA  95
            LL  +L G+ + +Q+I    +DP  P+ E            G   L+ A  AG +++++
Sbjct  869  LLDSSLEGEFDLVQRIIYEVDDPSLPNDE------------GITALHNAVCAGHTEIVKF  916

Query  96   LAKYGVNLNEKTTRGYTLLHCAAAWGRLETLKALVELDVDIEALNFRE-----ERARDVA  150
            L ++GVN+N    + G+T LHCAA+    ++   K LVE     + A+ + +      ++  ++
Sbjct  917  LVQFGVNVNAADSDGWTPLHCAASCNNVQVCKFLVESGAAVFAMTYSDMQTAADKCEEME  976

Query  151  ARYSQTECVEFL  162
              Y  T+C +FL
Sbjct  977  EGY--TQCSQFL  986


>ref|NP_775554.2| tumor protein p53 binding protein, 2 [Mus musculus]
Length=1134

 GENE ID: 209456 Trp53bp2 | transformation related protein 53 binding protein 2
[Mus musculus] (Over 10 PubMed links)

 Score = 58.2 bits (139),  Expect = 8e-07, Method: Compositional matrix adjust.
 Identities = 37/132 (28%), Positives = 68/132 (51%), Gaps = 22/132 (16%)

Query  39    LLQPALTGDVEGLQKIF---EDPENPHHEQAMQLLLEEDIVGRNLLYAACMAGQSDVIRA  95
             LL  +L G+ + +Q+I    +DP  P+ E            G   L+ A  AG +++++
Sbjct  936   LLDSSLEGEFDLVQRIIYEVDDPSLPNDE------------GITALHNAVCAGHTEIVKF  983

Query  96    LAKYGVNLNEKTTRGYTLLHCAAAWGRLETLKALVELDVDIEALNFRE-----ERARDVA  150
             L ++GVN+N    + G+T LHCAA+    ++   K LVE     + A+ + +      ++  ++
Sbjct  984   LVQFGVNVNAADSDGWTPLHCAASCNNVQVCKFLVESGAAVFAMTYSDMQTAADKCEEME  1043

Query  151   ARYSQTECVEFL  162
               Y  T+C +FL
Sbjct  1044  EGY--TQCSQFL  1053


>ref|XP_547518.2| PREDICTED: similar to Apoptosis stimulating of p53 protein 2
(Tumor suppressor p53-binding protein 2) (p53-binding protein
2) (53BP2) (Bcl2-binding protein) (Bbp) [Canis familiaris]
Length=1263

 GENE ID: 490397 TP53BP2 | tumor protein p53 binding protein, 2
[Canis lupus familiaris]

 Score = 58.2 bits (139),  Expect = 9e-07, Method: Compositional matrix adjust.
 Identities = 47/190 (24%), Positives = 87/190 (45%), Gaps = 30/190 (15%)

Query  39    LLQPALTGDVEGLQKIF---EDPENPHHEQAMQLLLEEDIVGRNLLYAACMAGQSDVIRA  95
             LL  +L G+ + +Q+I    +DP  P+ E            G   L+ A  AG +++++
Sbjct  1056  LLDSSLEGEFDLVQRIIYEVDDPSLPNDE------------GITALHNAVCAGHTEIVKF  1103

Query  96    LAKYGVNLNEKTTRGYTLLHCAAAWGRLETLKALVELDVDIEALNFRE-----ERARDVA  150
             L ++GVN+N    + G+T LHCAA+    ++   K LVE     + A  + +      ++  ++
Sbjct  1104  LVQFGVNVNAADSDGWTPLHCAASCNNVQVCKFLVESGAAVFATTYSDMQTAADKCEEME  1163

Query  151   ARYSQTECVEFLDWADARLTLKKYIAKVSLAVTDTEKGSGKLLKEDKNTILSACRAKNE-  209
               Y  T+C +FL      ++ +        V   A+ D E  +G   L    +   ++  R ++E
Sbjct  1164  EGY--TQCSQFLYGVQEKMGIMN--KGVLYALWDYEPQNGDELPMREGDCMTVIRREDED  1219

Query  210   -----WLETH  214
                   W   H
Sbjct  1220  ETEWWWARLH  1229


>ref|XP_001488127.2| PREDICTED: tumor protein p53 binding protein, 2 [Equus caballus]
Length=1125

 GENE ID: 100055278 TP53BP2 | tumor protein p53 binding protein, 2
[Equus caballus]

 Score = 58.2 bits (139),  Expect = 9e-07, Method: Compositional matrix adjust.
 Identities = 37/132 (28%), Positives = 68/132 (51%), Gaps = 22/132 (16%)

Query  39    LLQPALTGDVEGLQKIF---EDPENPHHEQAMQLLLEEDIVGRNLLYAACMAGQSDVIRA  95
             LL  +L G+ + +Q+I    +DP  P+ E            G   L+ A  AG +++++
Sbjct  927   LLDSSLEGEFDLVQRIIYEVDDPSLPNDE------------GITALHNAVCAGHTEIVKF  974

Query  96    LAKYGVNLNEKTTRGYTLLHCAAAWGRLETLKALVELDVDIEALNFRE-----ERARDVA  150
             L ++GVN+N    + G+T LHCAA+    ++   K LVE     + A+ + +      ++  ++
Sbjct  975   LVQFGVNVNAADSDGWTPLHCAASCNNVQVCKFLVESGAAVFAMTYSDMQTAADKCEEME  1034

Query  151   ARYSQTECVEFL  162
               Y  T+C +FL
Sbjct  1035  EGY--TQCSQFL  1044


>gb|ABQ22601.1|  78 kDa glucose regulated protein precursor-like protein [Callithrix
jacchus]
Length=125

 Score = 58.2 bits (139),  Expect = 9e-07, Method: Compositional matrix adjust.
 Identities = 29/75 (38%), Positives = 44/75 (58%), Gaps = 0/75 (0%)

Query  166  DARLTLKKYIAKVSLAVTDTEKGSGKLLKEDKNTILSACRAKNEWLETHTEASINELFEQ  225
            D R  L+ Y    +    + D EK  GKL   EDK T+  A   K EWLE+H +A I +    +
Sbjct  31   DTRNELESYAYSLKNQIGDKEKLGGKLSSEDKETMEKAVEEKIEWLESHQDADIEDFKAK  90
```

```
Query  226  RQQLEDIVTPIFTKM  240
            +++LE++V PI +K+
Sbjct  91   KKELEEVVQPIISKL  105
```

>**ref|XP_002433656.1|** heat shock protein, putative [Ixodes scapularis]
**gb|EEC05056.1|** heat shock protein, putative [Ixodes scapularis]
Length=658

**GENE ID: 8051659 IscW_ISCW017754** | heat shock protein, putative
[Ixodes scapularis]

```
 Score = 57.8 bits (138),  Expect = 1e-06, Method: Compositional matrix adjust.
 Identities = 33/76 (43%), Positives = 43/76 (56%), Gaps = 0/76 (0%)

Query  165  ADARLTLKKYIAKVSLAVTDTEKGSGKLLKEDKNTILSACRAKNEWLETHTEASINELFE  224
             +AR  L+ Y    +    + D EK   GKL   EDK TI   A    K +WLE H++A    EL E
Sbjct  563  VEARNELESYAYSLKNQIGDKEKMGGKLSDEDKKTIEQAVDEKIKWLEQHSDADAEELKE  622

Query  225  QRQQLEDIVTPIFTKM  240
            Q++QL D V PI  K+
Sbjct  623  QKKQLADTVQPIVAKL  638
```

>**ref|XP_001376253.1|** PREDICTED: similar to tumor protein p53 binding protein, 2 [Monodelphis domestica]
Length=1501

**GENE ID: 100025256 LOC100025256** | similar to tumor protein p53 binding protein,
2 [Monodelphis domestica]

```
 Score = 57.8 bits (138),  Expect = 1e-06, Method: Compositional matrix adjust.
 Identities = 37/132 (28%), Positives = 68/132 (51%), Gaps = 22/132 (16%)

Query  39    LLQPALTGDVEGLQKIF---EDPENPHHEQAMQLLLEEDIVGRNLLYAACMAGQSDVIRA  95
             LL   +L G+ + +Q+I     +DP   P+ E              G     L+ A   AG +++++
Sbjct  1303  LLDSSLEGEFDLVQRIIYEVDDPSLPNDE------------GITALHNAVCAGHTEIVKF  1350

Query  96    LAKYGVNLNEKTTRGYTLLHCAAAWGRLETLKALVELDVDIEALNFRE-----ERARDVA  150
             L ++GVN+N    + G+T LHCAA+    ++   K LVE     + A++ + +      ++  ++
Sbjct  1351  LVQFGVNVNAADSDGWTPLHCAASCNNVQVCKFLVESGAAVFAMTYSDMQTAADKCEEME  1410

Query  151   ARYSQTECVEFL  162
               Y  T+C +FL
Sbjct  1411  EGY--TQCSQFL  1420
```

>**ref|XP_002578195.1|** fetal globin-inducing factor [Schistosoma mansoni]
**emb|CAZ34433.1|** fetal globin-inducing factor, putative [Schistosoma mansoni]
Length=228

**GENE ID: 8346505 Smp_069840** | fetal globin-inducing factor
[Schistosoma mansoni] (10 or fewer PubMed links)

```
 Score = 57.4 bits (137),  Expect = 1e-06, Method: Compositional matrix adjust.
 Identities = 30/88 (34%), Positives = 47/88 (53%), Gaps = 0/88 (0%)

Query  68   QLLLEEDIVGRNLLYAACMAGQSDVIRALAKYGVNLNEKTTRGYTLLHCAAAWGRLETLK  127
            +LL   +D   G    L+ A    G   +V++ L KYG N+N +T   G+T LH AA W +L   ++
Sbjct  90   ELLSAKDQDGYTALHRAAYGGHVEVLQYLMKYGANINNRTEDGWTPLHSAAFWNKLSCVQ  149

Query  128  ALVELDVDIEALNFREERARDVAARYSQ  155
             L+    D+ AL     + A  +A    +Q
Sbjct  150  LLISAGADLNALTNSGQTALHLAVSNNQ  177
```

>**emb|CAQ52955.1|** CD4-specific ankyrin repeat protein D27.2 [synthetic construct]
Length=169

```
 Score = 57.4 bits (137),  Expect = 1e-06, Method: Compositional matrix adjust.
 Identities = 30/86 (34%), Positives = 47/86 (54%), Gaps = 0/86 (0%)

Query  77   GRNLLYAACMAGQSDVIRALAKYGVNLNEKTTRGYTLLHCAAAWGRLETLKALVELDVDI  136
            GR   L+ A   G   +++   L K+G ++N +   G T LH AAAWG LE ++ L++   D+
Sbjct  47   GRTSLHLAAREGHLEIVEVLLKHGADVNAQDWYGSTPLHLAAAWGHLEIVEVLLKNVADV  106

Query  137  EALNFREERARDVAARYSQTECVEFL  162
             A++          +AA Y+   E VE L
Sbjct  107  NAMDGDGSTPLHLAAHYAHLEVVEVL  132


 Score = 48.5 bits (114),  Expect = 6e-04, Method: Compositional matrix adjust.
 Identities = 32/87 (36%), Positives = 46/87 (52%), Gaps = 1/87 (1%)

Query  76   VGRNLLYAACMAGQSDVIRALAKYGVNLNEKTTRGYTLLHCAAAWGRLETLKALVELDVD  135
            +G+ LL AA  AGQ D +R L   G ++N K   G T LH AA  G LE ++ L++   D
Sbjct  14   LGKRLLEAA-RAGQDDEVRILMANGADVNAKDDEGRTSLHLAAREGHLEIVEVLLKHGAD  72

Query  136  IEALNFREERARDVAARYSQTECVEFL  162
            + A ++          +AA +    E VE L
Sbjct  73   VNAQDWYGSTPLHLAAAWGHLEIVEVL  99
```

>**ref|XP_001926385.1|** PREDICTED: ankyrin 1, erythrocytic, partial [Sus scrofa]
Length=1455

**GENE ID: 100155697 LOC100155697** | similar to Ankyrin-1 (Erythrocyte ankyrin)
(Ankyrin-R) [Sus scrofa]

```
 Score = 57.4 bits (137),  Expect = 1e-06, Method: Compositional matrix adjust.
 Identities = 36/103 (34%), Positives = 55/103 (53%), Gaps = 7/103 (6%)

Query  35   PKNPLLQPALTGDVEGLQKIFEDPENPHHEQAMQLLLEEDIV------GRNLLYAACMAG  88
            P NPL   P L      GL  +     +  H +  ++LL +E I++        G    L+ A +AG
```

```
Sbjct  15   PSNPLSLP-LCSSQNGLNGLHLASKEGHVKMVVELLHKEIILETTTKKGNTALHIAALAG  73

Query  89   QSDVIRALAKYGVNLNEKTTRGYTLLHCAAAWGRLETLKALVE  131
            Q +V+R L  YG N+N ++ +G+T L+ AA    LE +K L+E
Sbjct  74   QDEVVRELVNYGANVNAQSQKGFTPLYMAAQENHLEVVKFLLE  116


 Score = 47.4 bits (111),  Expect = 0.002, Method: Compositional matrix adjust.
 Identities = 32/117 (27%), Positives = 53/117 (45%), Gaps = 7/117 (5%)

Query  63   HEQAMQLLLEED-------IVGRNLLYAACMAGQSDVIRALAKYGVNLNEKTTRGYTLLH  115
            H +  ++LL++        + G   L+ AC      V+  L K G +++  T  G T LH
Sbjct  334  HHRVAKVLLDKGAKPNSRALNGFTPLHIACKKNHIRVMELLLKTGASIDAVTESGLTPLH  393

Query  116  CAAAWGRLETLKALVELDVDIEALNFREERARDVAARYSQTECVEFLDWADARLTLK  172
             A+  G L  +K L++ D      N + E    +AAR   TE  ++L    A++  K
Sbjct  394  VASFMGHLPIVKNLLQRDASPNVSNVKVETPLHMAARAGHTEVAKYLLQNKAKVNAK  450


 Score = 44.7 bits (104),  Expect = 0.009, Method: Compositional matrix adjust.
 Identities = 28/88 (31%), Positives = 42/88 (47%), Gaps = 0/88 (0%)

Query  81   LYAACMAGQSDVIRALAKYGVNLNEKTTRGYTLLHCAAAWGRLETLKALVELDVDIEALN  140
            L+ A   G +++++ L +   N N  TT G+T LH AA  G +ET  AL+E       +
Sbjct  458  LHCAARIGHTNMVKLLLENNANPNLATTAGHTPLHIAAREGHVETALALLEKGASQACMT  517

Query  141  FREERARDVAARYSQTECVEFLDWADAR  168
             +      VAA+Y +    E L   DA
Sbjct  518  KKGFTPLHVAAKYGKVRVAELLLEHDAH  545


 Score = 42.0 bits (97),  Expect = 0.059, Method: Compositional matrix adjust.
 Identities = 31/108 (28%), Positives = 48/108 (44%), Gaps = 9/108 (8%)

Query  63   HEQAMQLLLEEDIVGRNL--------LYAACMAGQSDVIRALAKYGVNLNEKTTRGYTLL  114
            H + + LLL +   G NL        L+     G   V   L K+GV ++  T  GYT L
Sbjct  631  HAEMVALLLSKQANG-NLGNKSGLTPLHLVAQEGHVPVADVLIKHGVTVDATTRMGYTPL  689

Query  115  HCAAAWGRLETLKALVELDVDIEALNFREERARDVAARYSQTECVEFL  162
            H A+ +G ++ +K L++   D+ A            AA+   T+ V  L
Sbjct  690  HVASHYGNIKLVKFLLQHQADVNAKTKLGYSPLHQAAQQGHTDIVTLL  737


 Score = 38.5 bits (88),  Expect = 0.67, Method: Compositional matrix adjust.
 Identities = 28/107 (26%), Positives = 46/107 (42%), Gaps = 7/107 (6%)

Query  63   HEQAMQLLLEED-------IVGRNLLYAACMAGQSDVIRALAKYGVNLNEKTTRGYTLLH  115
            H   ++LLLE +          G   L+ A   G  +   AL + G +    T +G+T LH
Sbjct  466  HTNMVKLLLENNANPNLATTAGHTPLHIAAREGHVETALALLEKGASQACMTKKGFTPLH  525

Query  116  CAAAWGRLETLKALVELDVDIEALNFREERARDVAARYSQTECVEFL  162
             AA +G++    + L+E D    A          VA  ++   + V+ L
Sbjct  526  VAAKYGKVRVAELLLEHDAHPNAAGKNGLTPLHVAVHHNHLDIVKLL  572
```

>**ref|XP_001093747.1|** PREDICTED: tumor protein p53 binding protein, 2 [Macaca mulatta]
Length=1412

**GENE ID: 705383 TP53BP2** | tumor protein p53 binding protein, 2 [Macaca mulatta]

```
 Score = 57.4 bits (137),  Expect = 1e-06, Method: Compositional matrix adjust.
 Identities = 37/132 (28%), Positives = 68/132 (51%), Gaps = 22/132 (16%)

Query  39    LLQPALTGDVEGLQKIF---EDPENPHHEQAMQLLLEEDIVGRNLLYAACMAGQSDVIRA  95
             LL  +L G+ + +Q+I    +DP  P+ E            G   L+ A  AG +++++
Sbjct  1214  LLDSSLEGEFDLVQRIIYEVDDPSLPNDE------------GITALHNAVCAGHTEIVKF  1261

Query  96    LAKYGVNLNEKTTRGYTLLHCAAAWGRLETLKALVELDVDIEALNFRE-----ERARDVA  150
             L ++GVN+N   + G+T LHCAA+   ++  K LVE    + A+ + +     ++  ++
Sbjct  1262  LVQFGVNVNAADSDGWTPLHCAASCNNVQVCKFLVESGAAVFAMTYSDMQTAADKCEEME  1321

Query  151   ARYSQTECVEFL  162
               Y  T+C +FL
Sbjct  1322  EGY--TQCSQFL  1331
```

>**emb|CAQ52952.1|** CD4-specific ankyrin repeat protein D6.1 [synthetic construct]
Length=169

```
 Score = 57.4 bits (137),  Expect = 1e-06, Method: Compositional matrix adjust.
 Identities = 30/86 (34%), Positives = 47/86 (54%), Gaps = 0/86 (0%)

Query  77   GRNLLYAACMAGQSDVIRALAKYGVNLNEKTTRGYTLLHCAAAWGRLETLKALVELDVDI  136
            GR  L+ A   G  +++  L K+G ++N +   G T LH AAAWG LE ++ L++   D+
Sbjct  47   GRTPLHLAAREGHLEIVEVLLKHGADVNAQDWYGSTPLHLAAAWGHLEIVEVLLKNVADV  106

Query  137  EALNFREERARDVAARYSQTECVEFL  162
             A++        +AA Y+  E VE L
Sbjct  107  NAMDDDGSTPLHLAAHYAHLEVVEVL  132


 Score = 48.9 bits (115),  Expect = 5e-04, Method: Compositional matrix adjust.
 Identities = 32/87 (36%), Positives = 46/87 (52%), Gaps = 1/87 (1%)

Query  76   VGRNLLYAACMAGQSDVIRALAKYGVNLNEKTTRGYTLLHCAAAWGRLETLKALVELDVD  135
            +G+ LL AA  AGQ D +R L   G ++N K   G T LH AA  G LE ++ L++   D
Sbjct  14   LGKKLLEAA-RAGQDDEVRILMANGADVNAKDDEGRTPLHLAAREGHLEIVEVLLKHGAD  72

Query  136  IEALNFREERARDVAARYSQTECVEFL  162
            + A ++        +AA +   E VE L
Sbjct  73   VNAQDWYGSTPLHLAAAWGHLEIVEVL  99
```

>**emb|CAQ52956.1|** CD4-specific ankyrin repeat protein D29.2 [synthetic construct]
Length=169

```
 Score = 57.4 bits (137),  Expect = 2e-06, Method: Compositional matrix adjust.
 Identities = 30/86 (34%), Positives = 47/86 (54%), Gaps = 0/86 (0%)
```

```
Query  77   GRNLLYAACMAGQSDVIRALAKYGVNLNEKTTRGYTLLHCAAAWGRLETLKALVELDVDI  136
            GR  L+ A   G  +++  L K+G ++N +    G T LH AAAWG LE ++ L++   D+
Sbjct  47   GRTPLHLAAREGHLEIVEVLLKHGADVNAQDWYGSTPLHLAAAWGHLEIVEVLLKNVADV  106

Query  137  EALNFREERARDVAARYSQTECVEFL  162
             A++        +AA Y+  E VE L
Sbjct  107  NAMDDDGSTPLHLAAHYAHLEVVEVL  132


 Score = 48.9 bits (115),  Expect = 5e-04, Method: Compositional matrix adjust.
 Identities = 32/87 (36%), Positives = 46/87 (52%), Gaps = 1/87 (1%)

Query  76   VGRNLLYAACMAGQSDVIRALAKYGVNLNEKTTRGYTLLHCAAAWGRLETLKALVELDVD  135
            +G+ LL AA  AGQ D +R L   G ++N K   G T LH AA  G LE ++ L++   D
Sbjct  14   LGKKLLEAA-RAGQDDEVRILMANGADVNAKDDEGRTPLHLAAREGHLEIVEVLLKHGAD  72

Query  136  IEALNFREERARDVAARYSQTECVEFL  162
            + A ++       +AA +   E VE L
Sbjct  73   VNAQDWYGSTPLHLAAAWGHLEIVEVL  99


>ref|XP_002345952.1|  PREDICTED: similar to ankyrin repeat domain 2 [Homo sapiens]
Length=245

 GENE ID: 100293913 LOC100293913 | similar to ankyrin repeat domain 2
[Homo sapiens]

 Score = 57.4 bits (137),  Expect = 2e-06, Method: Compositional matrix adjust.
 Identities = 34/89 (38%), Positives = 47/89 (52%), Gaps = 0/89 (0%)

Query  78   RNLLYAACMAGQSDVIRALAKYGVNLNEKTTRGYTLLHCAAAWGRLETLKALVELDVDIE  137
            R  L+ A + GQ +VIR L +YG       T+ G+T   H AA  G L  LK L  L    I+
Sbjct  94   RTPLHWAAIKGQMEVIRLLIEYGARPCLVTSVGWTPAHFAAEAGHLNILKTLHALHAAID  153

Query  138  ALNFREERARDVAARYSQTECVEFLDWAD  166
            A +F  +  + +A  Y Q  CV FL+ A+
Sbjct  154  APDFFGDTPKRIAQIYGQKACVAFLEKAE  182


>ref|NP_001155907.1|  hypothetical protein LOC100287718 [Homo sapiens]
 dbj|BAG65187.1|  unnamed protein product [Homo sapiens]
Length=222

 GENE ID: 100287718 LOC100287718 | similar to ankyrin repeat domain 2
[Homo sapiens]

 Score = 57.4 bits (137),  Expect = 2e-06, Method: Compositional matrix adjust.
 Identities = 33/86 (38%), Positives = 45/86 (52%), Gaps = 0/86 (0%)

Query  78   RNLLYAACMAGQSDVIRALAKYGVNLNEKTTRGYTLLHCAAAWGRLETLKALVELDVDIE  137
            R  L+ A + GQ +VIR L +YG       T+ G+T   H AA  G L  LK L  L    I+
Sbjct  71   RTPLHWAAIKGQMEVIRLLIEYGARPCLVTSVGWTPAHFAAEAGHLNILKTLHALHAAID  130

Query  138  ALNFREERARDVAARYSQTECVEFLD  163
            A +F  +  + +A  Y Q  CV FL+
Sbjct  131  APDFFGDTPKRIAQIYGQKACVAFLE  156


>ref|XP_001917133.1|  PREDICTED: similar to heat shock 70kDa protein 5 [Equus caballus]
Length=754

 GENE ID: 100067235 LOC100067235 | similar to heat shock protein 70
[Equus caballus]

 Score = 57.0 bits (136),  Expect = 2e-06, Method: Compositional matrix adjust.
 Identities = 30/75 (40%), Positives = 44/75 (58%), Gaps = 0/75 (0%)

Query  166  DARLTLKKYIAKVSLAVTDTEKGSGKLLKEDKNTILSACRAKNEWLETHTEASINELFEQ  225
            D R  L+ Y   +   + D EK  GKL  EDK T+  A   K EWLE+H +A I +   +
Sbjct  660  DTRNELESYAYSLKNQIGDKEKLGGKLSSEDKETMEKAVEEKIEWLESHQDADIEDFKAK  719

Query  226  RQQLEDIVTPIFTKM  240
            +++LE+IV PI +K+
Sbjct  720  KKELEEIVQPIISKL  734


>pdb|1YCS|B  Chain B, P53-53bp2 Complex
Length=239

 Score = 57.0 bits (136),  Expect = 2e-06, Method: Compositional matrix adjust.
 Identities = 38/136 (27%), Positives = 69/136 (50%), Gaps = 22/136 (16%)

Query  35   PKNPLLQPALTGDVEGLQKIF---EDPENPHHEQAMQLLLEEDIVGRNLLYAACMAGQSD  91
            P   LL  +L G+ + +Q+I    +DP  P+ E            G   L+ A  AG ++
Sbjct  37   PLALLLDSSLEGEFDLVQRIIYEVDDPSLPNDE------------GITALHNAVCAGHTE  84

Query  92   VIRALAKYGVNLNEKTTRGYTLLHCAAAWGRLETLKALVELDVDIEALNFRE-----ERA  146
            +++ L ++GVN+N   + G+T LHCAA+    ++ K LVE    + A+ + +     ++
Sbjct  85   IVKFLVQFGVNVNAADSDGWTPLHCAASCNNVQVCKFLVESGAAVFAMTYSDMQTAADKC  144

Query  147  RDVAARYSQTECVEFL  162
             ++   Y+Q  C +FL
Sbjct  145  EEMEEGYTQ--CSQFL  158


>gb|AAA37315.1|  immunoglobulin heavy chain binding protein
Length=142

 GENE ID: 14828 Hspa5 | heat shock protein 5 [Mus musculus]
(Over 100 PubMed links)

 Score = 57.0 bits (136),  Expect = 2e-06, Method: Compositional matrix adjust.
 Identities = 29/75 (38%), Positives = 44/75 (58%), Gaps = 0/75 (0%)
```

```
Query  166  DARLTLKKYIAKVSLAVTDTEKGSGKLLKEDKNTILSACRAKNEWLETHTEASINELFEQ  225
            D R  L+ Y   +   + D EK  GKL  +DK T+  A   K EWLE+H +A I +   +
Sbjct  48   DTRNELESYAYSLKNQIGDKEKLGGKLSSDDKETMEKAVEEKIEWLESHQDADIEDFKAK  107

Query  226  RQQLEDIVTPIFTKM  240
            +++LE+IV PI +K+
Sbjct  108  KKELEEIVQPIISKL  122
```

>**ref|XP_641640.1|** hypothetical protein [Dictyostelium discoideum AX4]
**gb|EAL67733.1|** hypothetical protein DDB_G0279589 [Dictyostelium discoideum AX4]
Length=150

**GENE ID: 3392801 DDBDRAFT_0218169** | hypothetical protein
[Dictyostelium discoideum AX4] (10 or fewer PubMed links)

Score = 56.6 bits (135), Expect = 2e-06, Method: Compositional matrix adjust.
Identities = 31/76 (40%), Positives = 43/76 (56%), Gaps = 0/76 (0%)

```
Query  74   DIVGRNLLYAACMAGQSDVIRALAKYGVNLNEKTTRGYTLLHCAAAWGRLETLKALVELD  133
            D +G   L+ A  AG ++V+ AL   G N+N K   G T LH AA   RLET+K LV+
Sbjct  37   DHLGNTALHYASNAGHTEVVEALVNAGANINIKNKHGDTPLHKAAGRNRLETVKFLVKSK  96

Query  134  VDIEALNFREERARDV  149
             ++E  N  +ER  D+
Sbjct  97   ANVEIENVDKERPCDI  112
```

>**dbj|BAD90025.1|** glucose-regulated protein 78kDa [Oncorhynchus mykiss]
Length=634

**GENE ID: 100135840 grp78** | glucose-regulated protein 78kDa
[Oncorhynchus mykiss] (10 or fewer PubMed links)

Score = 56.6 bits (135), Expect = 2e-06, Method: Compositional matrix adjust.
Identities = 30/75 (40%), Positives = 45/75 (60%), Gaps = 0/75 (0%)

```
Query  166  DARLTLKKYIAKVSLAVTDTEKGSGKLLKEDKNTILSACRAKNEWLETHTEASINELFEQ  225
            DAR  L+ Y   +   + D EK  GKL  EDK TI  A   K EW+E+H EA + +   +
Sbjct  534  DARNELESYAYSLKNQIGDKEKLGGKLSAEDKETIEKAVEEKIEWMESHQEAELEDFQAK  593

Query  226  RQQLEDIVTPIFTKM  240
            +++LE++V PI +K+
Sbjct  594  KKELEEVVQPIVSKL  608
```

>**ref|NP_001135114.1|** 78 kDa glucose-regulated protein [Salmo salar]
**gb|ACI33778.1|** 78 kDa glucose-regulated protein precursor [Salmo salar]
Length=657

**GENE ID: 100196613 grp78** | 78 kDa glucose-regulated protein [Salmo salar]

Score = 56.6 bits (135), Expect = 2e-06, Method: Compositional matrix adjust.
Identities = 30/75 (40%), Positives = 45/75 (60%), Gaps = 0/75 (0%)

```
Query  166  DARLTLKKYIAKVSLAVTDTEKGSGKLLKEDKNTILSACRAKNEWLETHTEASINELFEQ  225
            DAR  L+ Y   +   + D EK  GKL  EDK TI  A   K EW+E+H EA + +   +
Sbjct  558  DARNELESYAYSLKNQIGDKEKLGGKLSAEDKETIEKAVEEKIEWMESHQEAELEDFQAK  617

Query  226  RQQLEDIVTPIFTKM  240
            +++LE++V PI +K+
Sbjct  618  KKELEEVVQPIVSKL  632
```

>**ref|XP_001144115.1|** PREDICTED: heat shock 70kDa protein 5 (glucose-regulated protein, 78kDa) isoform 1 [Pan troglodytes]
Length=615

**GENE ID: 464733 HSPA5** | heat shock 70kDa protein 5 (glucose-regulated protein, 78kDa) [Pan troglodytes]

Score = 56.6 bits (135), Expect = 2e-06, Method: Compositional matrix adjust.
Identities = 30/75 (40%), Positives = 44/75 (58%), Gaps = 0/75 (0%)

```
Query  166  DARLTLKKYIAKVSLAVTDTEKGSGKLLKEDKNTILSACRAKNEWLETHTEASINELFEQ  225
            D R  L+ Y   +   + D EK  GKL  EDK T+  A   K EWLE+H +A I +   +
Sbjct  521  DTRNELESYAYSLKNQIGDKEKLGGKLSSEDKETMEKAVEEKIEWLESHQDADIEDFKAK  580

Query  226  RQQLEDIVTPIFTKM  240
            +++LE+IV PI +K+
Sbjct  581  KKELEEIVQPIISKL  595
```

>**gb|EAW87621.1|** heat shock 70kDa protein 5 (glucose-regulated protein, 78kDa), isoform CRA_b [Homo sapiens]
Length=459

**GENE ID: 3309 HSPA5** | heat shock 70kDa protein 5 (glucose-regulated protein, 78kDa) [Homo sapiens] (Over 100 PubMed links)

Score = 56.6 bits (135), Expect = 3e-06, Method: Compositional matrix adjust.
Identities = 30/75 (40%), Positives = 44/75 (58%), Gaps = 0/75 (0%)

```
Query  166  DARLTLKKYIAKVSLAVTDTEKGSGKLLKEDKNTILSACRAKNEWLETHTEASINELFEQ  225
            D R  L+ Y   +   + D EK  GKL  EDK T+  A   K EWLE+H +A I +   +
Sbjct  365  DTRNELESYAYSLKNQIGDKEKLGGKLSSEDKETMEKAVEEKIEWLESHQDADIEDFKAK  424

Query  226  RQQLEDIVTPIFTKM  240
            +++LE+IV PI +K+
Sbjct  425  KKELEEIVQPIISKL  439
```

>**ref|XP_518520.2|** PREDICTED: meprin A, alpha (PABA peptide hydrolase) [Pan troglodytes]
Length=865

```
 GENE ID: 462745 MEP1A | meprin A, alpha (PABA peptide hydrolase)
[Pan troglodytes]

 Score = 56.6 bits (135),  Expect = 3e-06, Method: Compositional matrix adjust.
 Identities = 33/86 (38%), Positives = 45/86 (52%), Gaps = 0/86 (0%)

Query  78   RNLLYAACMAGQSDVIRALAKYGVNLNEKTTRGYTLLHCAAAWGRLETLKALVELDVDIE  137
            R  L+ A + GQ +VIR L +YG      T+ G+T  H AA  G L  LK L  L   I+
Sbjct  40   RTPLHWAAIKGQMEVIRLLIEYGARPCLVTSVGWTPAHFAAESGHLNVLKTLHALHAAID  99

Query  138  ALNFREERARDVAARYSQTECVEFLD  163
            A +F  +   + +A  Y Q  CV FL+
Sbjct  100  APDFFGDTPKRIAQIYGQKACVAFLE  125


>dbj|BAE39187.1|  unnamed protein product [Mus musculus]
Length=655

 GENE ID: 14828 Hspa5 | heat shock protein 5 [Mus musculus]
(Over 100 PubMed links)

 Score = 56.6 bits (135),  Expect = 3e-06, Method: Compositional matrix adjust.
 Identities = 30/75 (40%), Positives = 44/75 (58%), Gaps = 0/75 (0%)

Query  166  DARLTLKKYIAKVSLAVTDTEKGSGKLLKEDKNTILSACRAKNEWLETHTEASINELFEQ  225
            D R  L+ Y    +    + D EK  GKL  EDK T+  A    K EWLE+H +A I +   +
Sbjct  561  DTRNGLESYAYSLKNQIGDKEKLGGKLSSEDKETMEKAVEEKIEWLESHQDADIEDFKAK  620

Query  226  RQQLEDIVTPIFTKM  240
            +++LE+IV PI +K+
Sbjct  621  KKELEEIVQPIISKL  635


>ref|XP_392417.3|  PREDICTED: similar to CG4393-PA [Apis mellifera]
Length=814

 GENE ID: 408887 LOC408887 | similar to CG4393-PA [Apis mellifera]
(10 or fewer PubMed links)

 Score = 56.6 bits (135),  Expect = 3e-06, Method: Compositional matrix adjust.
 Identities = 37/112 (33%), Positives = 57/112 (50%), Gaps = 14/112 (12%)

Query  63   HEQAMQLLLEE-------DIVGRNLLYAACMAGQSDVIRALAKYG-----VNLNEKTTRG  110
            H++ ++LLL+         D  G + L+ A  AG ++++R +    G      VNL  K
Sbjct  61   HKEVVKLLLQYEASTNVVDAKGSSPLHLAAWAGDAEIVRLILTQGPSVPKVNLTTKDNE-  119

Query  111  YTLLHCAAAWGRLETLKALVELDVDIEALNFREERARDVAARYSQTECVEFL  162
             T LHCAA +G  E +  L++   D    N R E A D+AA+Y + E V+ L
Sbjct  120  -TALHCAAQYGHTEVVAQLLQYGCDPSIRNSRGESALDLAAQYGRLETVQLL  170


 Score = 42.0 bits (97),  Expect = 0.071, Method: Compositional matrix adjust.
 Identities = 19/50 (38%), Positives = 32/50 (64%), Gaps = 0/50 (0%)

Query  81   LYAACMAGQSDVIRALAKYGVNLNEKTTRGYTLLHCAAAWGRLETLKALV  130
            L+ A   G ++V+  L +YG + + + +RG + L  AA +GRLET++ LV
Sbjct  122  LHCAAQYGHTEVVAQLLQYGCDPSIRNSRGESALDLAAQYGRLETVQLLV  171


>ref|XP_974604.2|  PREDICTED: similar to CG4393 CG4393-PA [Tribolium castaneum]
Length=1299

 GENE ID: 663468 LOC663468 | similar to CG4393-PA [Tribolium castaneum]

 Score = 56.2 bits (134),  Expect = 3e-06, Method: Compositional matrix adjust.
 Identities = 38/116 (32%), Positives = 56/116 (48%), Gaps = 9/116 (7%)

Query  63   HEQAMQLLLEED-------IVGRNLLYAACMAGQSDVIRAL--AKYGVNLNEKTTRGYTL  113
            H + ++LLL+ D       + G + L+ A  +G  D+++ L        N+N  T    T
Sbjct  61   HREIVKLLLDHDASTNIVDVKGSSPLHLAAWSGNVDIVKLLLSGPSICNVNLTTQDDETA  120

Query  114  LHCAAAWGRLETLKALVELDVDIEALNFREERARDVAARYSQTECVEFLDWADARL  169
            LHCAA +G     +  L+E   D    N R E A D+AA+Y + E VE L    D  L
Sbjct  121  LHCAAQYGHTAVVSLLLEHACDPGIRNCRGETALDLAAQYGRLETVELLVRTDPSL  176


>ref|XP_001098999.1|  PREDICTED: heat shock 70kDa protein 5 (glucose-regulated protein,
78kDa) isoform 1 [Macaca mulatta]
Length=586

 GENE ID: 703193 HSPA5 | heat shock 70kDa protein 5 (glucose-regulated protein,
78kDa) [Macaca mulatta]

 Score = 56.2 bits (134),  Expect = 3e-06, Method: Compositional matrix adjust.
 Identities = 30/75 (40%), Positives = 44/75 (58%), Gaps = 0/75 (0%)

Query  166  DARLTLKKYIAKVSLAVTDTEKGSGKLLKEDKNTILSACRAKNEWLETHTEASINELFEQ  225
            D R  L+ Y    +    + D EK  GKL  EDK T+  A    K EWLE+H +A I +   +
Sbjct  492  DTRNELESYAYSLKNQIGDKEKLGGKLSSEDKETMEKAVEEKIEWLESHQDADIEDFKAK  551

Query  226  RQQLEDIVTPIFTKM  240
            +++LE+IV PI +K+
Sbjct  552  KKELEEIVQPIISKL  566


>gb|AAA52614.1|  GRP78 precursor
 emb|CAA61201.1|  BiP [Homo sapiens]
Length=653

 GENE ID: 3309 HSPA5 | heat shock 70kDa protein 5 (glucose-regulated protein,
78kDa) [Homo sapiens] (Over 100 PubMed links)

 Score = 56.2 bits (134),  Expect = 3e-06, Method: Compositional matrix adjust.
 Identities = 30/75 (40%), Positives = 44/75 (58%), Gaps = 0/75 (0%)
```

```
Query  166  DARLTLKKYIAKVSLAVTDTEKGSGKLLKEDKNTILSACRAKNEWLETHTEASINELFEQ  225
            D R  L+ Y    +    + D EK  GKL  EDK T+  A    K EWLE+H +A I +    +
Sbjct  559  DTRNELESYAYSLKNQIGDKEKLGGKLSSEDKETMEKAVEEKIEWLESHQDADIEDFKAK  618

Query  226  RQQLEDIVTPIFTKM  240
            +++LE+IV PI +K+
Sbjct  619  KKELEEIVQPIISKL  633
```

>**ref|NP_001068616.1|** heat shock 70kDa protein 5 precursor [Bos taurus]
**sp|Q0VCX2.1|GRP78_BOVIN** RecName: Full=78 kDa glucose-regulated protein; AltName: Full=GRP 78; AltName: Full=Heat shock 70 kDa protein 5; Flags: Precursor
**gb|AAI19954.1|** Heat shock 70kDa protein 5 (glucose-regulated protein, 78kDa) [Bos taurus]
**gb|ABS45042.1|** heat shock 70kDa protein 5 [Bos taurus]
Length=655

**GENE ID: 415113 HSPA5** | heat shock 70kDa protein 5 (glucose-regulated protein, 78kDa) [Bos taurus] (10 or fewer PubMed links)

```
 Score = 56.2 bits (134),  Expect = 3e-06, Method: Compositional matrix adjust.
 Identities = 30/75 (40%), Positives = 44/75 (58%), Gaps = 0/75 (0%)

Query  166  DARLTLKKYIAKVSLAVTDTEKGSGKLLKEDKNTILSACRAKNEWLETHTEASINELFEQ  225
            D R  L+ Y    +    + D EK  GKL  EDK T+  A    K EWLE+H +A I +    +
Sbjct  561  DTRNELESYAYSLKNQIGDKEKLGGKLSSEDKETMEKAVEEKIEWLESHQDADIEDFKAK  620

Query  226  RQQLEDIVTPIFTKM  240
            +++LE+IV PI +K+
Sbjct  621  KKELEEIVQPIISKL  635
```

>**ref|NP_001126927.1|** heat shock 70kDa protein 5 (glucose-regulated protein, 78kDa) precursor [Pongo abelii]
**sp|Q5R4P0.1|GRP78_PONAB** RecName: Full=78 kDa glucose-regulated protein; AltName: Full=GRP 78; AltName: Full=Heat shock 70 kDa protein 5; Flags: Precursor
**emb|CAH93276.1|** hypothetical protein [Pongo abelii]
Length=654

**GENE ID: 100173944 HSPA5** | heat shock 70kDa protein 5 (glucose-regulated protein, 78kDa) [Pongo abelii]

```
 Score = 56.2 bits (134),  Expect = 3e-06, Method: Compositional matrix adjust.
 Identities = 30/75 (40%), Positives = 44/75 (58%), Gaps = 0/75 (0%)

Query  166  DARLTLKKYIAKVSLAVTDTEKGSGKLLKEDKNTILSACRAKNEWLETHTEASINELFEQ  225
            D R  L+ Y    +    + D EK  GKL  EDK T+  A    K EWLE+H +A I +    +
Sbjct  560  DTRNELESYAYSLKNQIGDKEKLGGKLSSEDKETMEKAVEEKIEWLESHQDADIEDFKAK  619

Query  226  RQQLEDIVTPIFTKM  240
            +++LE+IV PI +K+
Sbjct  620  KKELEEIVQPIISKL  634
```

>**sp|Q3S4T7.1|GRP78_SPETR** RecName: Full=78 kDa glucose-regulated protein; AltName: Full=GRP 78; AltName: Full=Heat shock 70 kDa protein 5; Flags: Precursor
**gb|AAZ94625.1|** GRP78 [Spermophilus tridecemlineatus]
Length=654

```
 Score = 56.2 bits (134),  Expect = 3e-06, Method: Compositional matrix adjust.
 Identities = 30/75 (40%), Positives = 44/75 (58%), Gaps = 0/75 (0%)

Query  166  DARLTLKKYIAKVSLAVTDTEKGSGKLLKEDKNTILSACRAKNEWLETHTEASINELFEQ  225
            D R  L+ Y    +    + D EK  GKL  EDK T+  A    K EWLE+H +A I +    +
Sbjct  560  DTRNELESYAYSLKNQIGDKEKLGGKLSSEDKETMEKAVEEKIEWLESHQDADIEDFKAK  619

Query  226  RQQLEDIVTPIFTKM  240
            +++LE+IV PI +K+
Sbjct  620  KKELEEIVQPIISKL  634
```

>**dbj|BAB23387.1|** unnamed protein product [Mus musculus]
Length=655

**GENE ID: 14828 Hspa5** | heat shock protein 5 [Mus musculus] (Over 100 PubMed links)

```
 Score = 56.2 bits (134),  Expect = 3e-06, Method: Compositional matrix adjust.
 Identities = 30/75 (40%), Positives = 44/75 (58%), Gaps = 0/75 (0%)

Query  166  DARLTLKKYIAKVSLAVTDTEKGSGKLLKEDKNTILSACRAKNEWLETHTEASINELFEQ  225
            D R  L+ Y    +    + D EK  GKL  EDK T+  A    K EWLE+H +A I +    +
Sbjct  561  DTRNELESYAYSLKNQIGDKEKLGGKLSSEDKETMEKAVEEKIEWLESHQDADIEDFKAK  620

Query  226  RQQLEDIVTPIFTKM  240
            +++LE+IV PI +K+
Sbjct  621  KKELEEIVQPIISKL  635
```

>**gb|EDL08617.1|** heat shock 70kD protein 5 (glucose-regulated protein), isoform CRA_b [Mus musculus]
Length=507

**GENE ID: 14828 Hspa5** | heat shock protein 5 [Mus musculus] (Over 100 PubMed links)

```
 Score = 56.2 bits (134),  Expect = 3e-06, Method: Compositional matrix adjust.
 Identities = 30/75 (40%), Positives = 44/75 (58%), Gaps = 0/75 (0%)

Query  166  DARLTLKKYIAKVSLAVTDTEKGSGKLLKEDKNTILSACRAKNEWLETHTEASINELFEQ  225
            D R  L+ Y    +    + D EK  GKL  EDK T+  A    K EWLE+H +A I +    +
Sbjct  413  DTRNELESYAYSLKNQIGDKEKLGGKLSSEDKETMEKAVEEKIEWLESHQDADIEDFKAK  472
```

```
Query  226  RQQLEDIVTPIFTKM  240
            +++LE+IV PI +K+
Sbjct  473  KKELEEIVQPIISKL  487


>ref|XP_537847.2|  PREDICTED: similar to 78 kDa glucose-regulated protein precursor
(GRP 78) (Immunoglobulin heavy chain binding protein) (BiP)
(Endoplasmic reticulum lumenal Ca(2+) binding protein grp78)
isoform 1 [Canis familiaris]
Length=666

 GENE ID: 480726 LOC480726 | similar to 78 kDa glucose-regulated protein
precursor (GRP 78) (Immunoglobulin heavy chain binding protein) (BiP)
(Endoplasmic reticulum lumenal Ca(2+) binding protein grp78)
[Canis lupus familiaris]

 Score = 56.2 bits (134),  Expect = 3e-06, Method: Compositional matrix adjust.
 Identities = 30/75 (40%), Positives = 44/75 (58%), Gaps = 0/75 (0%)

Query  166  DARLTLKKYIAKVSLAVTDTEKGSGKLLKEDKNTILSACRAKNEWLETHTEASINELFEQ  225
            D R  L+ Y    +    + D EK   GKL   EDK T+   A    K EWLE+H +A I +    +
Sbjct  572  DTRNELESYAYSLKNQIGDKEKLGGKLSSEDKETMEKAVEEKIEWLESHQDADIEDFKAK  631

Query  226  RQQLEDIVTPIFTKM  240
            +++LE+IV PI +K+
Sbjct  632  KKELEEIVQPIISKL  646


>dbj|BAE30705.1|  unnamed protein product [Mus musculus]
Length=655

 GENE ID: 14828 Hspa5 | heat shock protein 5 [Mus musculus]
(Over 100 PubMed links)

 Score = 56.2 bits (134),  Expect = 3e-06, Method: Compositional matrix adjust.
 Identities = 30/75 (40%), Positives = 44/75 (58%), Gaps = 0/75 (0%)

Query  166  DARLTLKKYIAKVSLAVTDTEKGSGKLLKEDKNTILSACRAKNEWLETHTEASINELFEQ  225
            D R  L+ Y    +    + D EK   GKL   EDK T+   A    K EWLE+H +A I +    +
Sbjct  561  DTRNELESYAYSLKNQIGDKEKLGGKLSSEDKETMEKAVEEKIEWLESHQDADIEDFKAK  620

Query  226  RQQLEDIVTPIFTKM  240
            +++LE+IV PI +K+
Sbjct  621  KKELEEIVQPIISKL  635


>ref|NP_005338.1|  heat shock 70kDa protein 5 [Homo sapiens]
 ref|XP_001099110.1|  PREDICTED: heat shock 70kDa protein 5 (glucose-regulated protein,
78kDa) isoform 2 [Macaca mulatta]
 ref|XP_520257.2|  PREDICTED: heat shock 70kDa protein 5 (glucose-regulated protein,
78kDa) isoform 3 [Pan troglodytes]
 10 more sequence titles

ref|XP_001144270.1|  PREDICTED: heat shock 70kDa protein 5 (glucose-regulated protein,
78kDa) isoform 2 [Pan troglodytes]
 sp|P11021.2|GRP78_HUMAN  RecName: Full=78 kDa glucose-regulated protein; AltName: Full=GRP
78; AltName: Full=Heat shock 70 kDa protein 5; AltName:
Full=Immunoglobulin heavy chain-binding protein; Short=BiP;
AltName: Full=Endoplasmic reticulum lumenal Ca(2+)-binding
protein grp78; Flags: Precursor
 gb|AAF42836.1|AF216292_1  endoplasmic reticulum lumenal Ca2+ binding protein grp78; BiP
[Homo sapiens]
 emb|CAB71335.1|  glucose-regulated protein [Homo sapiens]
 gb|AAH20235.1|  Heat shock 70kDa protein 5 (glucose-regulated protein, 78kDa)
[Homo sapiens]
 gb|ABD04090.1|  heat shock 70kDa protein 5 (glucose-regulated protein, 78kDa)
[Homo sapiens]
 gb|EAW87620.1|  heat shock 70kDa protein 5 (glucose-regulated protein, 78kDa),
isoform CRA_a [Homo sapiens]
 gb|ABM83099.1|  heat shock 70kDa protein 5 (glucose-regulated protein, 78kDa)
[synthetic construct]
 gb|ABM86294.1|  heat shock 70kDa protein 5 (glucose-regulated protein, 78kDa)
[synthetic construct]
 emb|CAQ08732.1|  heat shock 70kDa protein 5 (glucose-regulated protein, 78kDa)
[Homo sapiens]
Length=654

 GENE ID: 3309 HSPA5 | heat shock 70kDa protein 5 (glucose-regulated protein,
78kDa) [Homo sapiens] (Over 100 PubMed links)

 Score = 56.2 bits (134),  Expect = 3e-06, Method: Compositional matrix adjust.
 Identities = 30/75 (40%), Positives = 44/75 (58%), Gaps = 0/75 (0%)

Query  166  DARLTLKKYIAKVSLAVTDTEKGSGKLLKEDKNTILSACRAKNEWLETHTEASINELFEQ  225
            D R  L+ Y    +    + D EK   GKL   EDK T+   A    K EWLE+H +A I +    +
Sbjct  560  DTRNELESYAYSLKNQIGDKEKLGGKLSSEDKETMEKAVEEKIEWLESHQDADIEDFKAK  619

Query  226  RQQLEDIVTPIFTKM  240
            +++LE+IV PI +K+
Sbjct  620  KKELEEIVQPIISKL  634


>ref|ZP_01731588.1|  hypothetical protein CY0110_09016 [Cyanothece sp. CCY0110]
 gb|EAZ89000.1|  hypothetical protein CY0110_09016 [Cyanothece sp. CCY0110]
Length=206

 Score = 56.2 bits (134),  Expect = 3e-06, Method: Compositional matrix adjust.
 Identities = 24/58 (41%), Positives = 40/58 (68%), Gaps = 0/58 (0%)

Query  81   LYAACMAGQSDVIRALAKYGVNLNEKTTRGYTLLHCAAAWGRLETLKALVELDVDIEA  138
            +Y A ++G+S ++R L +YG +++ KT +G+T LH AA+ G  + +K L+E  VDI A
```

```
Sbjct  83   MYGAVISGKSKIVRLLIQYGADIHAKTLKGHTTLHWAASHGHQDIIKLLLEQGVDINA  140
```

>**ref|XP_863385.1|** PREDICTED: similar to 78 kDa glucose-regulated protein precursor (GRP 78) (Immunoglobulin heavy chain binding protein) (BiP) (Endoplasmic reticulum lumenal Ca(2+) binding protein grp78) isoform 5 [Canis familiaris]
Length=653

**GENE ID: 480726 LOC480726** | similar to 78 kDa glucose-regulated protein precursor (GRP 78) (Immunoglobulin heavy chain binding protein) (BiP) (Endoplasmic reticulum lumenal Ca(2+) binding protein grp78) [Canis lupus familiaris]

```
 Score = 56.2 bits (134),  Expect = 3e-06, Method: Compositional matrix adjust.
 Identities = 30/75 (40%), Positives = 44/75 (58%), Gaps = 0/75 (0%)

Query  166  DARLTLKKYIAKVSLAVTDTEKGSGKLLKEDKNTILSACRAKNEWLETHTEASINELFEQ  225
            D R  L+ Y   +   + D EK  GKL  EDK T+  A   K EWLE+H +A I +   +
Sbjct  559  DTRNELESYAYSLKNQIGDKEKLGGKLSSEDKETMEKAVEEKIEWLESHQDADIEDFKAK  618

Query  226  RQQLEDIVTPIFTKM  240
            +++LE+IV PI +K+
Sbjct  619  KKELEEIVQPIISKL  633
```

>**dbj|BAE79724.1|** immunoglobulin heavy-chain binding protein [Macaca fuscata]
Length=654

```
 Score = 56.2 bits (134),  Expect = 3e-06, Method: Compositional matrix adjust.
 Identities = 30/75 (40%), Positives = 44/75 (58%), Gaps = 0/75 (0%)

Query  166  DARLTLKKYIAKVSLAVTDTEKGSGKLLKEDKNTILSACRAKNEWLETHTEASINELFEQ  225
            D R  L+ Y   +   + D EK  GKL  EDK T+  A   K EWLE+H +A I +   +
Sbjct  560  DTRNELESYAYSLKNQIGDKEKLGGKLSSEDKETMEKAVEEKIEWLESHQDADIEDFKAK  619

Query  226  RQQLEDIVTPIFTKM  240
            +++LE+IV PI +K+
Sbjct  620  KKELEEIVQPIISKL  634
```

>**dbj|BAA11462.1|** 78 kDa glucose-regulated protein [Mus musculus]
Length=655

**GENE ID: 14828 Hspa5** | heat shock protein 5 [Mus musculus] (Over 100 PubMed links)

```
 Score = 56.2 bits (134),  Expect = 3e-06, Method: Compositional matrix adjust.
 Identities = 30/75 (40%), Positives = 44/75 (58%), Gaps = 0/75 (0%)

Query  166  DARLTLKKYIAKVSLAVTDTEKGSGKLLKEDKNTILSACRAKNEWLETHTEASINELFEQ  225
            D R  L+ Y   +   + D EK  GKL  EDK T+  A   K EWLE+H +A I +   +
Sbjct  561  DTRNELESYAYSLKNQIGDKEKLGGKLSSEDKETMEKAVEEKIEWLESHQDADIEDFKAK  620

Query  226  RQQLEDIVTPIFTKM  240
            +++LE+IV PI +K+
Sbjct  621  KKELEEIVQPIISKL  635
```

>**emb|CAA05361.1|** BiP [Mus musculus]
Length=655

**GENE ID: 14828 Hspa5** | heat shock protein 5 [Mus musculus] (Over 100 PubMed links)

```
 Score = 56.2 bits (134),  Expect = 3e-06, Method: Compositional matrix adjust.
 Identities = 30/75 (40%), Positives = 44/75 (58%), Gaps = 0/75 (0%)

Query  166  DARLTLKKYIAKVSLAVTDTEKGSGKLLKEDKNTILSACRAKNEWLETHTEASINELFEQ  225
            D R  L+ Y   +   + D EK  GKL  EDK T+  A   K EWLE+H +A I +   +
Sbjct  561  DTRNELESYAYSLKNQIGDKEKLGGKLSSEDKETMEKAVEEKIEWLESHQDADIEDFKAK  620

Query  226  RQQLEDIVTPIFTKM  240
            +++LE+IV PI +K+
Sbjct  621  KKELEEIVQPIISKL  635
```

>**gb|AAF13605.1|AF188611_1** BiP protein [Homo sapiens]
Length=639

**GENE ID: 3309 HSPA5** | heat shock 70kDa protein 5 (glucose-regulated protein, 78kDa) [Homo sapiens] (Over 100 PubMed links)

```
 Score = 56.2 bits (134),  Expect = 3e-06, Method: Compositional matrix adjust.
 Identities = 30/75 (40%), Positives = 44/75 (58%), Gaps = 0/75 (0%)

Query  166  DARLTLKKYIAKVSLAVTDTEKGSGKLLKEDKNTILSACRAKNEWLETHTEASINELFEQ  225
            D R  L+ Y   +   + D EK  GKL  EDK T+  A   K EWLE+H +A I +   +
Sbjct  542  DTRNELESYAYSLKNQIGDKEKLGGKLSSEDKETMEKAVEEKIEWLESHQDADIEDFKAK  601

Query  226  RQQLEDIVTPIFTKM  240
            +++LE+IV PI +K+
Sbjct  602  KKELEEIVQPIISKL  616
```

>**ref|NP_071705.3|** heat shock protein 5 precursor [Mus musculus]
**ref|NP_001156906.1|** heat shock protein 5 precursor [Mus musculus]
**sp|P20029.3|GRP78_MOUSE** RecName: Full=78 kDa glucose-regulated protein; AltName: Full=GRP 78; AltName: Full=Heat shock 70 kDa protein 5; AltName: Full=Immunoglobulin heavy chain-binding protein; Short=BiP; Flags: Precursor
**12 more sequence titles**

**dbj|BAC36166.1|** unnamed protein product [Mus musculus]

```
 gb|AAH50927.1|  Heat shock protein 5 [Mus musculus]
 dbj|BAE28609.1|  unnamed protein product [Mus musculus]
 dbj|BAE40825.1|  unnamed protein product [Mus musculus]
 dbj|BAE27328.1|  unnamed protein product [Mus musculus]
 dbj|BAE38982.1|  unnamed protein product [Mus musculus]
 dbj|BAE30576.1|  unnamed protein product [Mus musculus]
 dbj|BAE30882.1|  unnamed protein product [Mus musculus]
 gb|AAI12964.1|  HSPA5 protein [Homo sapiens]
 emb|CAM24607.1|  heat shock 70kD protein 5 (glucose-regulated protein) [Mus musculus]
 gb|EDL08616.1|  heat shock 70kD protein 5 (glucose-regulated protein), isoform
CRA_a [Mus musculus]
 gb|EDL08618.1|  heat shock 70kD protein 5 (glucose-regulated protein), isoform
CRA_a [Mus musculus]
Length=655

 GENE ID: 14828 Hspa5 | heat shock protein 5 [Mus musculus]
(Over 100 PubMed links)

 Score = 56.2 bits (134),  Expect = 3e-06, Method: Compositional matrix adjust.
 Identities = 30/75 (40%), Positives = 44/75 (58%), Gaps = 0/75 (0%)

Query  166  DARLTLKKYIAKVSLAVTDTEKGSGKLLKEDKNTILSACRAKNEWLETHTEASINELFEQ  225
            D R  L+ Y   +   + D EK  GKL  EDK T+  A   K EWLE+H +A I +   +
Sbjct  561  DTRNELESYAYSLKNQIGDKEKLGGKLSSEDKETMEKAVEEKIEWLESHQDADIEDFKAK  620

Query  226  RQQLEDIVTPIFTKM  240
            +++LE+IV PI +K+
Sbjct  621  KKELEEIVQPIISKL  635


>dbj|BAE35314.1|  unnamed protein product [Mus musculus]
Length=655

 GENE ID: 14828 Hspa5 | heat shock protein 5 [Mus musculus]
(Over 100 PubMed links)

 Score = 56.2 bits (134),  Expect = 3e-06, Method: Compositional matrix adjust.
 Identities = 30/75 (40%), Positives = 44/75 (58%), Gaps = 0/75 (0%)

Query  166  DARLTLKKYIAKVSLAVTDTEKGSGKLLKEDKNTILSACRAKNEWLETHTEASINELFEQ  225
            D R  L+ Y   +   + D EK  GKL  EDK T+  A   K EWLE+H +A I +   +
Sbjct  561  DTRNELESYAYSLKNQIGDKEKLGGKLSSEDKETMEKAVEEKIEWLESHQDADIEDFKAK  620

Query  226  RQQLEDIVTPIFTKM  240
            +++LE+IV PI +K+
Sbjct  621  KKELEEIVQPIISKL  635


>ref|XP_001927830.1|  PREDICTED: heat shock 70kDa protein 5, partial [Sus scrofa]
Length=666

 GENE ID: 407060 HSPA5 | heat shock 70kDa protein 5 [Sus scrofa]
(10 or fewer PubMed links)

 Score = 56.2 bits (134),  Expect = 3e-06, Method: Compositional matrix adjust.
 Identities = 30/75 (40%), Positives = 44/75 (58%), Gaps = 0/75 (0%)

Query  166  DARLTLKKYIAKVSLAVTDTEKGSGKLLKEDKNTILSACRAKNEWLETHTEASINELFEQ  225
            D R  L+ Y   +   + D EK  GKL  EDK T+  A   K EWLE+H +A I +   +
Sbjct  560  DTRNELESYAYSLKNQIGDKEKLGGKLSSEDKETMEKAVEEKIEWLESHQDADIEDFKAK  619

Query  226  RQQLEDIVTPIFTKM  240
            +++LE+IV PI +K+
Sbjct  620  KKELEEIVQPIISKL  634


>sp|P07823.1|GRP78_MESAU  RecName: Full=78 kDa glucose-regulated protein; AltName: Full=GRP
78; AltName: Full=Heat shock 70 kDa protein 5; AltName:
Full=Immunoglobulin heavy chain-binding protein; Short=BiP;
Flags: Precursor
 pir||A27414  dnaK-type molecular chaperone GRP78 precursor - Chinese hamster
 gb|AAA51448.1|  glucose-regulated protein
Length=654

 Score = 56.2 bits (134),  Expect = 3e-06, Method: Compositional matrix adjust.
 Identities = 30/75 (40%), Positives = 44/75 (58%), Gaps = 0/75 (0%)

Query  166  DARLTLKKYIAKVSLAVTDTEKGSGKLLKEDKNTILSACRAKNEWLETHTEASINELFEQ  225
            D R  L+ Y   +   + D EK  GKL  EDK T+  A   K EWLE+H +A I +   +
Sbjct  560  DTRNELESYAYSLKNQIGDKEKLGGKLSSEDKETMEKAVEEKIEWLESHQDADIEDFKAK  619

Query  226  RQQLEDIVTPIFTKM  240
            +++LE+IV PI +K+
Sbjct  620  KKELEEIVQPIISKL  634


>gb|ACT46911.1|  heat shock 70kDa protein 5 isoform 2 [Cervus elaphus]
Length=655

 Score = 56.2 bits (134),  Expect = 3e-06, Method: Compositional matrix adjust.
 Identities = 30/75 (40%), Positives = 44/75 (58%), Gaps = 0/75 (0%)

Query  166  DARLTLKKYIAKVSLAVTDTEKGSGKLLKEDKNTILSACRAKNEWLETHTEASINELFEQ  225
            D R  L+ Y   +   + D EK  GKL  EDK T+  A   K EWLE+H +A I +   +
Sbjct  561  DTRNELESYAYSLKNQIGDKEKLGGKLSPEDKETMEKAVEEKIEWLESHQDADIEDFKAK  620

Query  226  RQQLEDIVTPIFTKM  240
            +++LE+IV PI +K+
Sbjct  621  KKELEEIVQPIISKL  635
```

>**gb|EDL93171.1|** heat shock 70kDa protein 5 (glucose-regulated protein), isoform CRA_b [Rattus norvegicus]
Length=507

**GENE ID: 25617 Hspa5** | heat shock protein 5 [Rattus norvegicus]
(Over 10 PubMed links)

```
 Score = 56.2 bits (134),  Expect = 4e-06, Method: Compositional matrix adjust.
 Identities = 30/75 (40%), Positives = 44/75 (58%), Gaps = 0/75 (0%)

Query  166  DARLTLKKYIAKVSLAVTDTEKGSGKLLKEDKNTILSACRAKNEWLETHTEASINELFEQ  225
            D R  L+ Y   +   + D EK  GKL  EDK T+  A   K EWLE+H +A I +   +
Sbjct  413  DTRNELESYAYSLKNQIGDKEKLGGKLSPEDKETMEKAVEEKIEWLESHQDADIEDFKAK  472

Query  226  RQQLEDIVTPIFTKM  240
            +++LE+IV PI +K+
Sbjct  473  KKELEEIVQPIISKL  487
```

>**gb|ACJ65009.1|** GRP78 [Ctenopharyngodon idella]
Length=653

```
 Score = 56.2 bits (134),  Expect = 4e-06, Method: Compositional matrix adjust.
 Identities = 35/97 (36%), Positives = 54/97 (55%), Gaps = 1/97 (1%)

Query  144  ERARDVAARYSQTECVEFLDWADARLTLKKYIAKVSLAVTDTEKGSGKLLKEDKNTILSA  203
            ER  + A R++  E  +  +  DAR  L+ Y   +   + D EK  GKL  EDK  I  A
Sbjct  537  ERMVNEAERFAD-EDKKLKERIDARNELESYAYSLKNQIGDKEKLGGKLSSEDKEAIEKA  595

Query  204  CRAKNEWLETHTEASINELFEQRQQLEDIVTPIFTKM  240
               K EWLE+H EA + +   ++++LE++V PI +K+
Sbjct  596  VEEKIEWLESHQEAELEDFQAKKKELEEVVQPIVSKL  632
```

>**ref|NP_037215.1|** heat shock protein 5 precursor [Rattus norvegicus]
**sp|P06761.1|GRP78_RAT** RecName: Full=78 kDa glucose-regulated protein; AltName: Full=GRP 78; AltName: Full=Heat shock 70 kDa protein 5; AltName: Full=Immunoglobulin heavy chain-binding protein; Short=BiP; AltName: Full=Steroidogenesis-activator polypeptide; Flags: Precursor
**gb|AAA40817.1|** preimmunoglobulin heavy chain binding protein
**gb|AAH62017.1|** Heat shock protein 5 [Rattus norvegicus]
**gb|EDL93169.1|** heat shock 70kDa protein 5 (glucose-regulated protein), isoform CRA_a [Rattus norvegicus]
**gb|EDL93170.1|** heat shock 70kDa protein 5 (glucose-regulated protein), isoform CRA_a [Rattus norvegicus]
Length=654

**GENE ID: 25617 Hspa5** | heat shock protein 5 [Rattus norvegicus]
(Over 10 PubMed links)

```
 Score = 55.8 bits (133),  Expect = 4e-06, Method: Compositional matrix adjust.
 Identities = 30/75 (40%), Positives = 44/75 (58%), Gaps = 0/75 (0%)

Query  166  DARLTLKKYIAKVSLAVTDTEKGSGKLLKEDKNTILSACRAKNEWLETHTEASINELFEQ  225
            D R  L+ Y   +   + D EK  GKL  EDK T+  A   K EWLE+H +A I +   +
Sbjct  560  DTRNELESYAYSLKNQIGDKEKLGGKLSPEDKETMEKAVEEKIEWLESHQDADIEDFKAK  619

Query  226  RQQLEDIVTPIFTKM  240
            +++LE+IV PI +K+
Sbjct  620  KKELEEIVQPIISKL  634
```

>**ref|ZP_02178274.1|** ankyrin repeat domain protein [Hydrogenivirga sp. 128-5-R1-1]
**gb|EDP74900.1|** ankyrin repeat domain protein [Hydrogenivirga sp. 128-5-R1-1]
Length=202

```
 Score = 55.8 bits (133),  Expect = 4e-06, Method: Compositional matrix adjust.
 Identities = 39/110 (35%), Positives = 51/110 (46%), Gaps = 7/110 (6%)

Query  60   NPHHEQAMQLLLEE--DIVGRNL-----LYAACMAGQSDVIRALAKYGVNLNEKTTRGYT  112
            N  H + + LLLE+  D+   RNL     L+ A   G +D++R L   G  LN +   G T
Sbjct  88   NNGHREIVILLLEKGADVNARNLNGWTPLHLASRNGYADIVRILVDRGAELNARNGAGLT  147

Query  113  LLHCAAAWGRLETLKALVELDVDIEALNFREERARDVAARYSQTECVEFL  162
             LH A   G L  +K LV    D+ A +     A D A  Y   E  EFL
Sbjct  148  PLHVAVMNGHLPVVKILVRSGADVSAKDNSGLTALDFAREYGHEEVAEFL  197
```

>**ref|NP_001080064.1|** heat shock 70kDa protein 5 (glucose-regulated protein, 78kDa) [Xenopus laevis]
**gb|AAH41200.1|** Hspa5 protein [Xenopus laevis]
Length=655

**GENE ID: 379756 hspa5** | heat shock 70kDa protein 5 (glucose-regulated protein, 78kDa) [Xenopus laevis] (10 or fewer PubMed links)

```
 Score = 55.8 bits (133),  Expect = 4e-06, Method: Compositional matrix adjust.
 Identities = 31/75 (41%), Positives = 43/75 (57%), Gaps = 0/75 (0%)

Query  166  DARLTLKKYIAKVSLAVTDTEKGSGKLLKEDKNTILSACRAKNEWLETHTEASINELFEQ  225
            D R  L+ Y   +   + D EK  GKL  EDK TI  A   K EWLE+H +A I +   +
Sbjct  561  DTRNELESYAYSLKNQIGDKEKLGGKLSSEDKETIEKAVEEKIEWLESHQDADIEDFKAK  620

Query  226  RQQLEDIVTPIFTKM  240
            +++LE+IV PI  K+
Sbjct  621  KKELEEIVQPIVGKL  635
```

>**gb|AAV66400.1|** heat-shock 70-kDa protein 5 [Macaca fascicularis]
Length=602

```
 Score = 55.8 bits (133),  Expect = 4e-06, Method: Compositional matrix adjust.
 Identities = 30/75 (40%), Positives = 44/75 (58%), Gaps = 0/75 (0%)
```

```
Query  166  DARLTLKKYIAKVSLAVTDTEKGSGKLLKEDKNTILSACRAKNEWLETHTEASINELFEQ  225
            D R  L+ Y    +    + D EK   GKL   EDK T+   A    K EWLE+H +A I +    +
Sbjct  528  DTRNELESYAYSLKNQIGDKEKLGGKLSSEDKETMEKAVEEKIEWLESHQDADIEDFKAK  587

Query  226  RQQLEDIVTPIFTKM  240
            +++LE+IV PI +K+
Sbjct  588  KKELEEIVQPIISKL  602
```

>**ref|XP_782071.1|** PREDICTED: hypothetical protein [Strongylocentrotus purpuratus]
**ref|XP_001198050.1|** PREDICTED: hypothetical protein [Strongylocentrotus purpuratus]
Length=548

**GENE ID: 576698 LOC576698** | hypothetical LOC576698
[Strongylocentrotus purpuratus]

```
 Score = 55.8 bits (133),  Expect = 4e-06, Method: Compositional matrix adjust.
 Identities = 44/120 (36%), Positives = 62/120 (51%), Gaps = 9/120 (7%)

Query  79   NLLYAACMA---GQSDVIRALAKYGV-NLNEKTTRGYTLLHCAAAWGRLETLKALVELDV  134
            NL + A M+    G     +R L + GV N+NEK   +G T   H AA   G+LE L+ LVE+
Sbjct  282  NLAFPAHMSAASGDLGHLRLLIEQGVVNINEKDDKGSTPAHRAAGNGKLECLQWLVEMGA  341

Query  135  DIEALNFREERARDVAARYSQTECVEFLDWADARLTLKKYIAKVSLAVTDTEKGSGKLLK  194
            +I    N     E    DVA R++Q   CV+ L     D+             A+V      +D E+    KLL+
Sbjct  342  NIHVQNSAGETPMDVAHRFAQLACVKLLKGDDS-----DSDAEVMGGYSDPEERQDKLLQ  396


 Score = 47.8 bits (112),  Expect = 0.001, Method: Compositional matrix adjust.
 Identities = 26/81 (32%), Positives = 41/81 (50%), Gaps = 0/81 (0%)

Query  82   YAACMAGQSDVIRALAKYGVNLNEKTTRGYTLLHCAAAWGRLETLKALVELDVDIEALNF  141
            + A + GQ    I+AL    GV++N +  RG +   H AAA G     TL +++      D E   N
Sbjct  117  HVAAIRGQDQCIQALTAQGVSMNARDLRGNSPAHLAAAHGNSYTLSSILRAGTDNEGKNI  176

Query  142  REERARDVAARYSQTECVEFL  162
                A   +AA + +   C++ L
Sbjct  177  TTWTATHIAAFHGRLGCLQLL  197
```

>**gb|AAH77757.1|** LOC397850 protein [Xenopus laevis]
Length=658

**GENE ID: 397850 LOC397850** | heavy-chain binding protein BiP [Xenopus laevis]
(10 or fewer PubMed links)

```
 Score = 55.8 bits (133),  Expect = 4e-06, Method: Compositional matrix adjust.
 Identities = 31/75 (41%), Positives = 43/75 (57%), Gaps = 0/75 (0%)

Query  166  DARLTLKKYIAKVSLAVTDTEKGSGKLLKEDKNTILSACRAKNEWLETHTEASINELFEQ  225
            D R  L+ Y    +    + D EK   GKL   EDK TI   A    K EWLE+H +A I +    +
Sbjct  561  DTRNELESYAYSLKNQIGDKEKLGGKLSSEDKETIEKAVEEKIEWLESHQDADIEDFKAK  620

Query  226  RQQLEDIVTPIFTKM  240
            +++LE+IV PI   K+
Sbjct  621  KKELEEIVQPIVGKL  635
```

>**ref|XP_001328606.1|** ankyrin repeat protein [Trichomonas vaginalis G3]
**gb|EAY16383.1|** ankyrin repeat protein, putative [Trichomonas vaginalis G3]
Length=574

**GENE ID: 4774392 TVAG_359920** | ankyrin repeat protein
[Trichomonas vaginalis G3] (10 or fewer PubMed links)

```
 Score = 55.8 bits (133),  Expect = 4e-06, Method: Compositional matrix adjust.
 Identities = 31/111 (27%), Positives = 59/111 (53%), Gaps = 0/111 (0%)

Query  72   EEDIVGRNLLYAACMAGQSDVIRALAKYGVNLNEKTTRGYTLLHCAAAWGRLETLKALVE  131
            E+D +G+   L+ A M     ++++ L   +G N++EK    G T LH A     +L+ ++ L+
Sbjct  416  EKDYLGKTALHIAEMFNNEEIVKFLLSHGANIDEKDNDGLTALHIAVKSNQLKIVEFLLS  475

Query  132  LDVDIEALNFREERARDVAARYSQTECVEFLDWADARLTLKKYIAKVSLAV  182
                  +I    ++   + A   +A + +Q + VEFL     A +   K Y+ K +L +
Sbjct  476  HGANINEKDYLGKTALHIAVKSNQLKIVEFLLSHGANINEKDYLGKTALHI  526


 Score = 49.3 bits (116),  Expect = 4e-04, Method: Compositional matrix adjust.
 Identities = 26/91 (28%), Positives = 49/91 (53%), Gaps = 0/91 (0%)

Query  72   EEDIVGRNLLYAACMAGQSDVIRALAKYGVNLNEKTTRGYTLLHCAAAWGRLETLKALVE  131
            E+D +G+   L+ A   + Q   ++   L   +G N++EK    G T LH A   +    ET++ L+
Sbjct  350  EKDYLGKTALHIAVKSNQLKIVEFLLSHGANIDEKNNDGLTALHFAVLYNDKETVEFLLS  409

Query  132  LDVDIEALNFREERARDVAARYSQTECVEFL  162
                  +I+   ++   + A   +A   ++   E V+FL
Sbjct  410  HGANIDEKDYLGKTALHIAEMFNNEEIVKFL  440


 Score = 48.9 bits (115),  Expect = 5e-04, Method: Compositional matrix adjust.
 Identities = 34/113 (30%), Positives = 54/113 (47%), Gaps = 0/113 (0%)

Query  72   EEDIVGRNLLYAACMAGQSDVIRALAKYGVNLNEKTTRGYTLLHCAAAWGRLETLKALVE  131
            E+D   G   L+ A   + Q   ++   L   +G N+NEK    G T LH A     +L+ ++ L+
Sbjct  317  EKDNDGLTALHIAVKSNQLKIVEFLLSHGANINEKDYLGKTALHIAVKSNQLKIVEFLLS  376

Query  132  LDVDIEALNFREERARDVAARYSQTECVEFLDWADARLTLKKYIAKVSLAVTD  184
                  +I+   N      A   A   Y+   E VEFL     A +   K Y+ K +L + +
Sbjct  377  HGANIDEKNNDGLTALHFAVLYNDKETVEFLLSHGANIDEKDYLGKTALHIAE  429


 Score = 43.9 bits (102),  Expect = 0.018, Method: Compositional matrix adjust.
 Identities = 29/106 (27%), Positives = 50/106 (47%), Gaps = 0/106 (0%)

Query  77   GRNLLYAACMAGQSDVIRALAKYGVNLNEKTTRGYTLLHCAAAWGRLETLKALVELDVDI  136
```

```
                 G   L+ A +    + +  L  +G N++EK   G T LH A  +   E +K L+    +I
Sbjct  388  GLTALHFAVLYNDKETVEFLLSHGANIDEKDYLGKTALHIAEMFNNEEIVKFLLSHGANI  447

Query  137  EALNFREERARDVAARYSQTECVEFLDWADARLTLKKYIAKVSLAV  182
            +  +     A  +A + +Q + VEFL    A +  K Y+ K +L +
Sbjct  448  DEKDNDGLTALHIAVKSNQLKIVEFLLSHGANINEKDYLGKTALHI  493
```

```
 Score = 43.9 bits (102),  Expect = 0.019, Method: Compositional matrix adjust.
 Identities = 26/91 (28%), Positives = 48/91 (52%), Gaps = 0/91 (0%)

Query  72   EEDIVGRNLLYAACMAGQSDVIRALAKYGVNLNEKTTRGYTLLHCAAAWGRLETLKALVE  131
            E+D  G   L+ A  + Q +++  L  +G N++EK   G T LH A    +L+ ++ L+
Sbjct  284  EKDNGGLTALHIAVDSNQLEIVEFLLSHGANIDEKDNDGLTALHIAVKSNQLKIVEFLLS  343

Query  132  LDVDIEALNFREERARDVAARYSQTECVEFL  162
               +I   ++  + A  +A + +Q + VEFL
Sbjct  344  HGANINEKDYLGKTALHIAVKSNQLKIVEFL  374
```

```
 Score = 42.0 bits (97),  Expect = 0.062, Method: Compositional matrix adjust.
 Identities = 29/94 (30%), Positives = 48/94 (51%), Gaps = 4/94 (4%)

Query  93   IRALAKY----GVNLNEKTTRGYTLLHCAAAWGRLETLKALVELDVDIEALNFREERARD  148
            I++L +Y    G N+NEK   G T LH A    +LE ++ L+    +I+  +     A
Sbjct  268  IQSLCEYFLSHGANINEKDNGGLTALHIAVDSNQLEIVEFLLSHGANIDEKDNDGLTALH  327

Query  149  VAARYSQTECVEFLDWADARLTLKKYIAKVSLAV  182
            +A + +Q + VEFL    A +  K Y+ K +L +
Sbjct  328  IAVKSNQLKIVEFLLSHGANINEKDYLGKTALHI  361
```

>**ref|XP_785346.1|** PREDICTED: hypothetical protein, partial [Strongylocentrotus purpuratus]
**ref|XP_001202564.1|** PREDICTED: hypothetical protein, partial [Strongylocentrotus purpuratus]
Length=475

**GENE ID: 580180 LOC580180** | hypothetical LOC580180
[Strongylocentrotus purpuratus]

```
 Score = 55.8 bits (133),  Expect = 4e-06, Method: Compositional matrix adjust.
 Identities = 36/88 (40%), Positives = 49/88 (55%), Gaps = 4/88 (4%)

Query  79   NLLYAACMA---GQSDVIRALAKYGV-NLNEKTTRGYTLLHCAAAWGRLETLKALVELDV  134
            NL + A M+   G    +R L + GV N+NEK  +G T  H AA  G+LE L+ LVE+
Sbjct  296  NLAFPAHMSAASGDLGHLRLLIEQGVVNINEKDDKGSTPAHRAAGNGKLECLQWLVEMGA  355

Query  135  DIEALNFREERARDVAARYSQTECVEFL  162
            +I   N   E   DVA R++Q  CV+ L
Sbjct  356  NIHVQNSAGETPMDVAHRFAQLACVKLL  383
```

```
 Score = 48.1 bits (113),  Expect = 8e-04, Method: Compositional matrix adjust.
 Identities = 26/81 (32%), Positives = 41/81 (50%), Gaps = 0/81 (0%)

Query  82   YAACMAGQSDVIRALAKYGVNLNEKTTRGYTLLHCAAAWGRLETLKALVELDVDIEALNF  141
            + A + GQ   I+AL   GV++N +  RG +  H AAA G   TL +++    D E  N
Sbjct  131  HVAAIRGQDQCIQALTAQGVSMNARDLRGNSPAHLAAAHGNSYTLSSILRAGTDNEGKNI  190

Query  142  REERARDVAARYSQTECVEFL  162
                A  +AA + +  C++ L
Sbjct  191  TTWTATHIAAFHGRLGCLQLL  211
```

```
 Score = 34.7 bits (78),  Expect = 9.3, Method: Compositional matrix adjust.
 Identities = 23/86 (26%), Positives = 39/86 (45%), Gaps = 4/86 (4%)

Query  82   YAACMAGQSDVIRALAKYGVNLNEKTTRGYTLLHCAAAWGRLETLKALVELDVDIE----  137
            + A   G+   ++ L K+G   +E    G    H AA  G L  LK LV     I
Sbjct  197  HIAAFHGRLGCLQLLLKWGARTDEADGNGNIPAHLAAQEGHLPCLKFLVSSGATISDTLG  256

Query  138  ALNFREERARDVAARYSQTECVEFLD  163
            A N   +  + +A++Y +  CV++++
Sbjct  257  ARNDNGDTPKTLASQYYKQHCVDYIN  282
```

>**ref|NP_001081462.1|** heavy-chain binding protein BiP precursor [Xenopus laevis]
**sp|Q91883.1|GRP78_XENLA** RecName: Full=78 kDa glucose-regulated protein; AltName: Full=GRP 78; AltName: Full=Heat shock 70 kDa protein 5; AltName: Full=Immunoglobulin heavy chain-binding protein; Short=BiP; Flags: Precursor
**gb|AAB08760.1|** heavy-chain binding protein BiP [Xenopus laevis]
Length=658

**GENE ID: 397850 LOC397850** | heavy-chain binding protein BiP [Xenopus laevis]
(10 or fewer PubMed links)

```
 Score = 55.8 bits (133),  Expect = 4e-06, Method: Compositional matrix adjust.
 Identities = 31/75 (41%), Positives = 43/75 (57%), Gaps = 0/75 (0%)

Query  166  DARLTLKKYIAKVSLAVTDTEKGSGKLLKEDKNTILSACRAKNEWLETHTEASINELFEQ  225
            D R  L+ Y   +   + D EK  GKL  EDK TI  A   K EWLE+H +A I +   +
Sbjct  561  DTRNELESYAYSLKNQIGDKEKLGGKLSSEDKETIEKAVEEKIEWLESHQDADIEDFKAK  620

Query  226  RQQLEDIVTPIFTKM  240
            +++LE+IV PI  K+
Sbjct  621  KKELEEIVQPIVGKL  635
```

>**ref|YP_002730164.1|** ankyrin domain protein [Persephonella marina EX-H1]
**gb|ACO04784.1|** ankyrin domain protein [Persephonella marina EX-H1]
Length=238

**GENE ID: 7675398 PERMA_0372** | ankyrin domain protein

[Persephonella marina EX-H1] (10 or fewer PubMed links)

```
 Score = 55.8 bits (133),  Expect = 5e-06, Method: Compositional matrix adjust.
 Identities = 35/125 (28%), Positives = 62/125 (49%), Gaps = 9/125 (7%)

Query  38   PLLQPALTGDVEGLQKIFEDPENPHHEQAMQLLLEEDIVGRNLLYAACMAGQSDVIRALA  97
            PL +    GD++ ++ + E  E+P+          +DI+G   L+ A   G +++ + L
Sbjct  50   PLNRAVSKGDIKLVKILLEKGEDPN---------SKDIIGWTPLHEAAFKGYTEIAKILI  100

Query  98   KYGVNLNEKTTRGYTLLHCAAAWGRLETLKALVELDVDIEALNFREERARDVAARYSQTE  157
            + G ++N K   G T LH A++ G L+ +K L++   DI A N +       AAR
Sbjct  101  EAGADVNAKDNDGETPLHIASSEGHLDMVKFLIKHGADINARNKKGRTPLHYAARGGNLS  160

Query  158  CVEFL  162
             V++L
Sbjct  161  VVKYL  165
```

>**ref|XP_002053831.1|** GJ23129 [Drosophila virilis]
 **gb|EDW67351.1|** GJ23129 [Drosophila virilis]
Length=1016

 **GENE ID: 6631026 Dvir\GJ23129** | GJ23129 gene product from transcript GJ23129-RA
[Drosophila virilis] (10 or fewer PubMed links)

```
 Score = 55.8 bits (133),  Expect = 5e-06, Method: Compositional matrix adjust.
 Identities = 39/117 (33%), Positives = 54/117 (46%), Gaps = 10/117 (8%)

Query  63   HEQAMQLLLEE-------DIVGRNLLYAACMAGQSDVIRAL---AKYGVNLNEKTTRGYT  112
            HE  ++LLL         D  G + L+ A  AG++D++R L        N N +T    T
Sbjct  60   HEDIVRLLLAHEASTNLPDSRGSSPLHLAAWAGETDIVRLLLTQPYRPANANLQTIEQET  119

Query  113  LLHCAAAWGRLETLKALVELDVDIEALNFREERARDVAARYSQTECVEFLDWADARL  169
             LHCAA  G    L  L+  D D    N R E   D+AA+Y + + V+ L  A   L
Sbjct  120  PLHCAAQHGHTGALALLLSHDADPNMRNSRGETPLDLAAQYGRLQAVQMLIRAHPEL  176
```

>**gb|AAD10949.1|** ankyrin repeat-containing protein 2 [Arabidopsis thaliana]
Length=342

```
 Score = 55.5 bits (132),  Expect = 5e-06, Method: Compositional matrix adjust.
 Identities = 41/123 (33%), Positives = 55/123 (44%), Gaps = 9/123 (7%)

Query  41   QPALTGDVEGLQKIFEDPENPHHEQAMQLLLEEDIVGRNLLYAACMAGQSDVIRALAKYG  100
            Q A  GDVEGL+       N          EED  GR  L+ AC  G+    + L   G
Sbjct  224  QTASLGDVEGLKAALASGGNKD---------EEDSEGRTALHFACGYGELKCAQVLIDAG  274

Query  101  VNLNEKTTRGYTLLHCAAAWGRLETLKALVELDVDIEALNFREERARDVAARYSQTECVE  160
             ++N       T LH AA +GR E++  L+E    +   N  E+   DVA   SQ E V+
Sbjct  275  ASVNAVDKNKNTPLHYAAGYGRKESVSLLLENGAAVTLQNLDEKTPIDVAKLNSQLEVVK  334

Query  161  FLD  163
             L+
Sbjct  335  LLE  337


 Score = 36.2 bits (82),  Expect = 3.9, Method: Compositional matrix adjust.
 Identities = 37/167 (22%), Positives = 69/167 (41%), Gaps = 20/167 (11%)

Query  37   NPLLQPAL----TGDVEGLQKIFEDPENPHH-EQAMQL----LLEEDIVG----------  77
            +P L+P L     G     + K + DPE      +AM +    L ++ +
Sbjct  158  DPELKPILDEIDAGGPSAMMKYWNDPEVLKKLGEAMGMPVAGLPDQTVSAEPEVAEEGEE  217

Query  78   -RNLLYAACMAGQSDVIRALAKYGVNLNEKTTRGYTLLHCAAAWGRLETLKALVELDVDI  136
              ++++      G  + ++A    G N +E+ + G T LH A  +G L+  + L++    +
Sbjct  218  EESIVHQTASLGDVEGLKAALASGGNKDEEDSEGRTALHFACGYGELKCAQVLIDAGASV  277

Query  137  EALNFREERARDVAARYSQTECVEFLDWADARLTLKKYIAKVSLAVT  183
             A++  +      AA Y + E V  L    A +TL+    K  + V
Sbjct  278  NAVDKNKNTPLHYAAGYGRKESVSLLLENGAAVTLQNLDEKTPIDVA  324
```

>**ref|XP_001198426.1|** PREDICTED: similar to ankyrin 2,3/unc44 [Strongylocentrotus purpuratus]
 **ref|XP_001196730.1|** PREDICTED: similar to ankyrin 2,3/unc44 [Strongylocentrotus purpuratus]
Length=2028

 **GENE ID: 762699 LOC762699** | similar to ankyrin 2,3/unc44
[Strongylocentrotus purpuratus]

```
 Score = 55.5 bits (132),  Expect = 5e-06, Method: Compositional matrix adjust.
 Identities = 48/203 (23%), Positives = 87/203 (42%), Gaps = 32/203 (15%)

Query  14    FFSQQEEENEEEEAQEPEETGPKNPLLQPALTGDVEGLQKI-----------------FE  56
             F S+  + NEE ++        + PLL  A  G+ E ++ +                 F
Sbjct  1319  FISKGVDVNEENDSG-------RIPLLGAAFHGNTEVMEYLSQQGSEVNKSDAKGWTPFN  1371

Query  57    DPENPHHEQAMQLLLEEDI-----VGRNLLYAACMAGQSDVIRALAKYGVNLNEKTTRGY  111
                   H +A++ L+ + +      G + LYAA + G  D+++     GV++NE+   G
Sbjct  1372  AAVQYGHLEAVKYLMTKGVELNRYAGNSPLYAAAVYGHVDLVKFFISKGVDVNEENGSGR  1431

Query  112   TLLHCAAAWGRLETLKALVELDVDIEALNFREERARDVAARYSQTECVEFLDWADARLTL  171
               LH AA  G +E ++ L++   ++   + R     + A +Y   E V++L    A   L
Sbjct  1432  IPLHGAAIHGNVEVMEYLIQQASEVNKSDARGWTPFNAAVQYGHIEAVKYLMTEGAE--L  1489

Query  172   KKYIAKVSLAVTDTEKGSGKLLK  194
              +Y     L  +  + G G L+K
Sbjct  1490  NRYAGMTPL-YSAAQSGHGDLVK  1511


 Score = 54.3 bits (129),  Expect = 1e-05, Method: Compositional matrix adjust.
 Identities = 44/171 (25%), Positives = 77/171 (45%), Gaps = 29/171 (16%)

Query  14    FFSQQEEENEEEEAQEPEETGPKNPLLQPALTGDVEGLQKIFED-------------PEN  60
             F S+  + NEE +      TG + PL   A+ G+VE ++ + +              P N
Sbjct  1125  FISKGADVNEEND------TG-RIPLHGAAIPGNVEVMEYLIQQASEVNKSDAKGWTPFN  1177
```

```
Query  61    P----HHEQAMQLLLEEDI-----VGRNLLYAACMAGQSDVIRALAKYGVNLNEKTTRGY  111
                   H +A++ L+ + +      G + LYAA + G  D+++     GVN+NE+   G
Sbjct  1178  AAVQYGHLEAVKYLMTKGVELNRYAGNSPLYAAAVYGHVDLVKFFISKGVNVNEENGSGR  1237

Query  112   TLLHCAAAWGRLETLKALVELDVDIEALNFREERARDVAARYSQTECVEFL  162
               LH AA  G  E +K L++   ++   +       + A +Y   E V++L
Sbjct  1238  IPLHGAAIHGNTEIMKYLIQQGSNVNKSSATGWTPFNAAVQYGHLEAVKYL  1288


 Score = 51.6 bits (122),  Expect = 7e-05, Method: Compositional matrix adjust.
 Identities = 41/171 (23%), Positives = 75/171 (43%), Gaps = 29/171 (16%)

Query  14    FFSQQEEENEEEEAQEPEETGPKNPLLQPALTGDVEGLQKIFED-------------PEN  60
             F S+  + NEE ++        + PLL  A  G+ E ++ + +              P N
Sbjct  931   FISKGVDVNEENDSG-------RIPLLGAAFHGNTEVMEYLIQQASEVNKSDAKGWTPFN  983

Query  61    P----HHEQAMQLLLEEDI-----VGRNLLYAACMAGQSDVIRALAKYGVNLNEKTTRGY  111
                   H +A++ L+ + +      G + LYAA + G  D+++     GV++NE+   G
Sbjct  984   AAVQYGHLEAVKYLMTKGVELNRYAGNSPLYAAAVYGHVDLVKFFISKGVDVNEENDSGR  1043

Query  112   TLLHCAAAWGRLETLKALVELDVDIEALNFREERARDVAARYSQTECVEFL  162
               LH AA  G  E +K L++   ++   +       + A +Y   E V++L
Sbjct  1044  IPLHGAAIHGNTEIMKYLIQQGSNVNKSSATGWTPFNAAVQYGHLEAVKYL  1094


 Score = 50.8 bits (120),  Expect = 2e-04, Method: Compositional matrix adjust.
 Identities = 32/123 (26%), Positives = 55/123 (44%), Gaps = 5/123 (4%)

Query  55    FEDPENPHHEQAMQLLLEEDIV-----GRNLLYAACMAGQSDVIRALAKYGVNLNEKTTR  109
             F D     H ++++ L+ E        G   LYAA  +G  D+++     GV++NE+
Sbjct  1564  FNDAVQGGHLESVKYLITEGAKQNRCDGMTPLYAAVQSGHIDLVKFFISKGVDVNEENDT  1623

Query  110   GYTLLHCAAAWGRLETLKALVELDVDIEALNFREERARDVAARYSQTECVEFLDWADARL  169
             G T LH AA    +E +  L+E   D+  ++         AA+    + V++L      +
Sbjct  1624  GRTPLHVAAIHANIEVMTYLIEQGSDVNKMDAEGWTPLHAAAQKGHLDIVDYLTLNGGDM  1683

Query  170   TLK  172
              LK
Sbjct  1684  NLK  1686


 Score = 48.1 bits (113),  Expect = 9e-04, Method: Compositional matrix adjust.
 Identities = 24/86 (27%), Positives = 44/86 (51%), Gaps = 0/86 (0%)

Query  77   GRNLLYAACMAGQSDVIRALAKYGVNLNEKTTRGYTLLHCAAAWGRLETLKALVELDVDI  136
            G + LY A + G  DV++ L   G ++NE+   G   LH AAA G +E ++ L++    +
Sbjct  326  GISPLYTASLGGHMDVVKFLISRGADVNEEGDEGRIPLHGAAARGYIEVMEYLIQQGSTV  385

Query  137  EALNFREERARDVAARYSQTECVEFL  162
               + +     + A +Y   + V++L
Sbjct  386  NKGDAKGWTPFNAAVKYGHLDAVKYL  411


 Score = 47.8 bits (112),  Expect = 0.001, Method: Compositional matrix adjust.
 Identities = 37/171 (21%), Positives = 75/171 (43%), Gaps = 29/171 (16%)

Query  14    FFSQQEEENEEEEAQEPEETGPKNPLLQPALTGDVEGLQKIFEDPENPH-----------  62
             F S+  + NEE ++        + PL   A+ G+ E ++ + +   N +
Sbjct  1028  FISKGVDVNEENDSG-------RIPLHGAAIHGNTEIMKYLIQQGSNVNKSSATGWTPFN  1080

Query  63    ------HEQAMQLLLEED-----IVGRNLLYAACMAGQSDVIRALAKYGVNLNEKTTRGY  111
                   H +A++ L+ E        G   LY+A ++G  D+++     G ++NE+   G
Sbjct  1081  AAVQYGHLEAVKYLMTEGAELNRYAGMTPLYSAALSGHVDLVKFFISKGADVNEENDTGR  1140

Query  112   TLLHCAAAWGRLETLKALVELDVDIEALNFREERARDVAARYSQTECVEFL  162
               LH AA  G +E ++ L++   ++   + +     + A +Y   E V++L
Sbjct  1141  IPLHGAAIPGNVEVMEYLIQQASEVNKSDAKGWTPFNAAVQYGHLEAVKYL  1191


 Score = 44.3 bits (103),  Expect = 0.013, Method: Compositional matrix adjust.
 Identities = 28/106 (26%), Positives = 54/106 (50%), Gaps = 6/106 (5%)

Query  63   HEQAMQLLLEED-----IVGRNLLYAACMAGQSDVIRA-LAKYGVNLNEKTTRGYTLLHC  116
            H +A++ L+ E        G N L+AA   G  D+++  ++  GV++NE+   G   LH
Sbjct  795  HLEAVKYLMTEGAKQNRFDGMNPLHAAAQFGHLDIVKFFISTKGVDVNEENDTGRIPLHG  854

Query  117  AAAWGRLETLKALVELDVDIEALNFREERARDVAARYSQTECVEFL  162
            AA  G +E ++ L++   ++   + +     + A +Y   E V++L
Sbjct  855  AAIHGNVEVMEYLIQQASEVNKSDAKGWTPFNAAVQYGHLEAVKYL  900


 Score = 43.5 bits (101),  Expect = 0.022, Method: Compositional matrix adjust.
 Identities = 35/151 (23%), Positives = 65/151 (43%), Gaps = 29/151 (19%)

Query  14   FFSQQEEENEEEEAQEPEETGPKNPLLQPALTGDVEGLQKIFEDPENPH-----------  62
            F S+  + NEE++  E        PL   A+ G +E ++ + +   N +
Sbjct  539  FISKGADVNEEDDEGEI-------PLHGAAVEGHIEVMEYLIQQGSNVNKIDAKGWTSFN  591

Query  63   ------HEQAMQLLLEEDIV-----GRNLLYAACMAGQSDVIRALAKYGVNLNEKTTRGY  111
                  H + ++ L+ E        G   LYAA  +   D+++     G ++NE+  +G
Sbjct  592  AAVQGGHLEGVKYLMTEGAKQNRYDGMTPLYAAAQSNHLDIVKFFISNGADVNEEHDKGM  651

Query  112  TLLHCAAAWGRLETLKALVELDVDIEALNFR  142
              LH AA+ G LE ++ L++   D+   + R
Sbjct  652  VPLHGAASGGHLEVMEYLIQQGSDVNKASTR  682


 Score = 43.1 bits (100),  Expect = 0.025, Method: Compositional matrix adjust.
 Identities = 27/105 (25%), Positives = 50/105 (47%), Gaps = 5/105 (4%)

Query  63   HEQAMQLLLEEDI-----VGRNLLYAACMAGQSDVIRALAKYGVNLNEKTTRGYTLLHCA  117
            H +A++ L+ E        G   LYAA   G  D+++ L   G ++NE+   G   LH A
Sbjct  698  HLEAVKCLMAEGAKQNRCAGMTPLYAAAHFGHLDIVKFLFSNGADVNEENDDGMIPLHGA  757

Query  118  AAWGRLETLKALVELDVDIEALNFREERARDVAARYSQTECVEFL  162
            A  G +E ++ L++   ++  ++ +       A +    E V++L
Sbjct  758  ATEGHMEVMEYLIQQGSELNKVDPKGRTPFIAAVQEGHLEAVKYL  802
```

```
 Score = 42.7 bits (99),  Expect = 0.038, Method: Compositional matrix adjust.
 Identities = 22/86 (25%), Positives = 42/86 (48%), Gaps = 0/86 (0%)

Query  77   GRNLLYAACMAGQSDVIRALAKYGVNLNEKTTRGYTLLHCAAAWGRLETLKALVELDVDI  136
            G   LY A + G  D+++ L     ++NE+  +G    LH AA   G+ + ++ L++   D+
Sbjct  423  GMTPLYDAAVLGYLDIVKYLISNAADVNEENDKGVIPLHGAAIQGQNKVMEYLIQQGSDV  482

Query  137  EALNFREERARDVAARYSQTECVEFL  162
               +         + A +Y   E V++L
Sbjct  483  NKKDNTGRTPFNAAVQYGHVEAVKYL  508


 Score = 41.2 bits (95),  Expect = 0.12, Method: Compositional matrix adjust.
 Identities = 26/105 (24%), Positives = 50/105 (47%), Gaps = 5/105 (4%)

Query  63    HEQAMQLLLEED-----IVGRNLLYAACMAGQSDVIRALAKYGVNLNEKTTRGYTLLHCA  117
             H +A++ L+ +         G + LYAA + G  D+++     GV++NE+    G   L  A
Sbjct  1281  HLEAVKYLMTKGAELNRYAGNSPLYAAAVYGHVDLVKFFISKGVDVNEENDSGRIPLLGA  1340

Query  118   AAWGRLETLKALVELDVDIEALNFREERARDVAARYSQTECVEFL  162
             A  G  E ++ L +   ++   + +     + A +Y   E V++L
Sbjct  1341  AFHGNTEVMEYLSQQGSEVNKSDAKGWTPFNAAVQYGHLEAVKYL  1385


 Score = 40.8 bits (94),  Expect = 0.15, Method: Compositional matrix adjust.
 Identities = 25/105 (23%), Positives = 50/105 (47%), Gaps = 5/105 (4%)

Query  63   HEQAMQLLLEED-----IVGRNLLYAACMAGQSDVIRALAKYGVNLNEKTTRGYTLLHCA  117
            H +A++ L+ +         G + LY A + G  D+++     GV++NE+    G   L  A
Sbjct  893  HLEAVKYLMTKGAELNRYAGNSPLYTAAVYGHVDLVKFFISKGVDVNEENDSGRIPLLGA  952

Query  118  AAWGRLETLKALVELDVDIEALNFREERARDVAARYSQTECVEFL  162
            A  G  E ++ L++   ++   + +     + A +Y   E V++L
Sbjct  953  AFHGNTEVMEYLIQQASEVNKSDAKGWTPFNAAVQYGHLEAVKYL  997


 Score = 40.4 bits (93),  Expect = 0.18, Method: Compositional matrix adjust.
 Identities = 21/59 (35%), Positives = 35/59 (59%), Gaps = 0/59 (0%)

Query  72    EEDIVGRNLLYAACMAGQSDVIRALAKYGVNLNEKTTRGYTLLHCAAAWGRLETLKALV  130
             EE+   GR   L+ A + G S+V++ L + G N+N+    G+T   + A    G LE++K L+
Sbjct  1522  EENDSGRIPLHGAAIHGSSEVMKYLIQQGSNVNKSDGTGWTPFNDAVQGGHLESVKYLI  1580


 Score = 38.9 bits (89),  Expect = 0.48, Method: Compositional matrix adjust.
 Identities = 39/171 (22%), Positives = 71/171 (41%), Gaps = 29/171 (16%)

Query  14    FFSQQEEENEEEEAQEPEETGPKNPLLQPALTGDVEGLQKIFED-------------PEN  60
             F S+   + NEE   +          + PL   A+ G+VE ++ + +              P N
Sbjct  1416  FISKGVDVNEENGSG-------RIPLHGAAIHGNVEVMEYLIQQASEVNKSDARGWTPFN  1468

Query  61    P----HHEQAMQLLLEED-----IVGRNLLYAACMAGQSDVIRALAKYGVNLNEKTTRGY  111
                   H +A++ L+ E         G    LY+A  +G  D+++       V++NE+    G
Sbjct  1469  AAVQYGHIEAVKYLMTEGAELNRYAGMTPLYSAAQSGHGDLVKFFISKEVDVNEENDSGR  1528

Query  112   TLLHCAAAWGRLETLKALVELDVDIEALNFREERARDVAARYSQTECVEFL  162
               LH AA  G  E +K L++   ++   +      + A +    E V++L
Sbjct  1529  IPLHGAAIHGSSEVMKYLIQQGSNVNKSDGTGWTPFNDAVQGGHLESVKYL  1579


 Score = 38.9 bits (89),  Expect = 0.50, Method: Compositional matrix adjust.
 Identities = 21/86 (24%), Positives = 41/86 (47%), Gaps = 0/86 (0%)

Query  77   GRNLLYAACMAGQSDVIRALAKYGVNLNEKTTRGYTLLHCAAAWGRLETLKALVELDVDI  136
            G   LYAA   G   ++       G ++NE+   G    LH AA   G +E ++ L++   ++
Sbjct  520  GMTPLYAAARFGHLHIVDYFISKGADVNEEDDEGEIPLHGAAVEGHIEVMEYLIQQGSNV  579

Query  137  EALNFREERARDVAARYSQTECVEFL  162
              ++ +   + + A +    E V++L
Sbjct  580  NKIDAKGWTSFNAAVQGGHLEGVKYL  605


 Score = 35.8 bits (81),  Expect = 4.7, Method: Compositional matrix adjust.
 Identities = 19/59 (32%), Positives = 33/59 (55%), Gaps = 0/59 (0%)

Query  72   EEDIVGRNLLYAACMAGQSDVIRALAKYGVNLNEKTTRGYTLLHCAAAWGRLETLKALV  130
            EE+  G    L+ A + GQ+ V+   L + G ++N+K    G T   + A   +G +E +K L+
Sbjct  451  EENDKGVIPLHGAAIQGQNKVMEYLIQQGSDVNKKDNTGRTPFNAAVQYGHVEAVKYLI  509


 Score = 35.4 bits (80),  Expect = 6.3, Method: Compositional matrix adjust.
 Identities = 30/125 (24%), Positives = 54/125 (43%), Gaps = 9/125 (7%)

Query  38   PLLQPALTGDVEGLQKIFEDPENPHHEQAMQLLLEEDIVGRNLLYAACMAGQSDVIRALA  97
            P+    AL G +E +  +     NP+                G   L+AA   G + ++  L
Sbjct  199  PMYNAALEGHLEDVDDLISRGANPNTPSKG---------GLRPLHAADREGHAHIVDFLI  249

Query  98   KYGVNLNEKTTRGYTLLHCAAAWGRLETLKALVELDVDIEALNFREERARDVAARYSQTE  157
              G +++ +    G T LH AAA G L+ L++LV  + ++    +      + A +    E
Sbjct  250  LQGADVSVECDLGQTPLHTAAAKGYLDILESLVSEETNVSMEDHTGWTPFNAAVQCGHLE  309

Query  158  CVEFL  162
             V++L
Sbjct  310  AVKYL  314
```

>**ref|XP_799947.2|** PREDICTED: similar to multiple ankyrin repeats single kh domain protein [Strongylocentrotus purpuratus]
**ref|XP_001184895.1|** PREDICTED: similar to multiple ankyrin repeats single kh domain protein [Strongylocentrotus purpuratus]
Length=557

**GENE ID: 577150 LOC577150** | similar to multiple ankyrin repeats single kh domain protein [Strongylocentrotus purpuratus]

```
 Score = 55.5 bits (132),  Expect = 5e-06, Method: Compositional matrix adjust.
```

```
 Identities = 32/93 (34%), Positives = 47/93 (50%), Gaps = 9/93 (9%)

Query  38   PLLQPALTGDVEGLQKIFEDPENPHHEQAMQLLLEEDIVGRNLLYAACMAGQSDVIRALA  97
            PLLQ A   DV   +K+ ED ++         +L++D  G + L+ AC+AG    +  L
Sbjct  19   PLLQAARNADVAAFKKMLEDGDD---------VLQKDERGLSPLHEACLAGYVRTVHLLI  69

Query  98   KYGVNLNEKTTRGYTLLHCAAAWGRLETLKALV  130
              G N+  +T  GYT L CAA  G  +  + LV
Sbjct  70   SIGANMEAETEEGYTPLLCAAGNGHNDITRILV  102
```

**>emb|CAQ52948.1|** CD4-specific ankyrin repeat protein D2.1 [synthetic construct]
Length=169

```
 Score = 55.5 bits (132),  Expect = 6e-06, Method: Compositional matrix adjust.
 Identities = 29/82 (35%), Positives = 44/82 (53%), Gaps = 0/82 (0%)

Query  81   LYAACMAGQSDVIRALAKYGVNLNEKTTRGYTLLHCAAAWGRLETLKALVELDVDIEALN  140
            L+ A M G  +++  L KYG ++N    + GYT LH AAA+G LE ++ L++    D+ A +
Sbjct  51   LHLAAMGGHLEIVEVLLKYGADVNAWDSWGYTPLHLAAAYGHLEIVEVLLKNGADVNASD  110

Query  141  FREERARDVAARYSQTECVEFL  162
                    +AA      E VE L
Sbjct  111  IDGWTPLHLAASNGHLEIVEVL  132


 Score = 49.3 bits (116),  Expect = 4e-04, Method: Compositional matrix adjust.
 Identities = 32/87 (36%), Positives = 44/87 (50%), Gaps = 1/87 (1%)

Query  76   VGRNLLYAACMAGQSDVIRALAKYGVNLNEKTTRGYTLLHCAAAWGRLETLKALVELDVD  135
            +G+ LL AA  AGQ D +R L   G ++N      G T LH AA  G LE ++ L++   D
Sbjct  14   LGKKLLEAA-RAGQDDEVRILMANGADVNAFDANGITSLHLAAMGGHLEIVEVLLKYGAD  72

Query  136  IEALNFREERARDVAARYSQTECVEFL  162
            + A +         +AA Y   E VE L
Sbjct  73   VNAWDSWGYTPLHLAAAYGHLEIVEVL  99


 Score = 38.5 bits (88),  Expect = 0.78, Method: Compositional matrix adjust.
 Identities = 22/82 (26%), Positives = 40/82 (48%), Gaps = 0/82 (0%)

Query  81   LYAACMAGQSDVIRALAKYGVNLNEKTTRGYTLLHCAAAWGRLETLKALVELDVDIEALN  140
            L+ A    G  +++  L K G ++N      G+T LH AA+ G LE ++ L++    D+   +
Sbjct  84   LHLAAAYGHLEIVEVLLKNGADVNASDIDGWTPLHLAASNGHLEIVEVLLKHSADVNTQD  143

Query  141  FREERARDVAARYSQTECVEFL  162
               + A D++         +  E L
Sbjct  144  KFGKTAFDISIDNGNKDLAEIL  165
```

**>ref|YP_001958145.1|** hypothetical protein Aasi_1071 [Candidatus Amoebophilus asiaticus 5a2]
**gb|ACE06416.1|** hypothetical protein Aasi_1071 [Candidatus Amoebophilus asiaticus 5a2]
Length=347

**GENE ID: 6377394 Aasi_1071** | hypothetical protein
[Candidatus Amoebophilus asiaticus 5a2]

```
 Score = 55.1 bits (131),  Expect = 6e-06, Method: Compositional matrix adjust.
 Identities = 36/107 (33%), Positives = 53/107 (49%), Gaps = 7/107 (6%)

Query  63   HEQAMQLLLEEDIV-------GRNLLYAACMAGQSDVIRALAKYGVNLNEKTTRGYTLLH  115
            H + + LLL+  I        G   L+ A + G  +VI+AL   G ++N K   G ++LH
Sbjct  205  HPETIHLLLQSGINVNVKNKDGNTALHGAAVYGYIEVIQALLAQGADVNSKNKDGNSVLH  264

Query  116  CAAAWGRLETLKALVELDVDIEALNFREERARDVAARYSQTECVEFL  162
             AAA+G+ E LK L++   DI A N     A  +AA   Q +     L
Sbjct  265  LAAAYGQTEVLKILLDAGADIHARNQENNSALHLAAYKCQDKATRIL  311


 Score = 53.5 bits (127),  Expect = 2e-05, Method: Compositional matrix adjust.
 Identities = 35/120 (29%), Positives = 58/120 (48%), Gaps = 9/120 (7%)

Query  43   ALTGDVEGLQKIFEDPENPHHEQAMQLLLEEDIVGRNLLYAACMAGQSDVIRALAKYGVN  102
            A +GDVE ++ +  +   N +            D  G + L+ A +     + I  L + G+N
Sbjct  168  AASGDVEMVKVLLTEGFNVY---------ANDSHGNSSLHFAAINNHPETIHLLLQSGIN  218

Query  103  LNEKTTRGYTLLHCAAAWGRLETLKALVELDVDIEALNFREERARDVAARYSQTECVEFL  162
            +N K   G T LH AA +G +E ++AL+    D+ + N        +AA Y QTE ++ L
Sbjct  219  VNVKNKDGNTALHGAAVYGYIEVIQALLAQGADVNSKNKDGNSVLHLAAAYGQTEVLKIL  278
```

**>ref|XP_001993641.1|** GH20822 [Drosophila grimshawi]
**gb|EDV94377.1|** GH20822 [Drosophila grimshawi]
Length=1338

**GENE ID: 6567512 Dgri\GH20822** | GH20822 gene product from transcript GH20822-RA
[Drosophila grimshawi] (10 or fewer PubMed links)

```
 Score = 55.1 bits (131),  Expect = 6e-06, Method: Compositional matrix adjust.
 Identities = 42/133 (31%), Positives = 61/133 (45%), Gaps = 15/133 (11%)

Query  63   HEQAMQLLLEE-------DIVGRNLLYAACMAGQSDVIRAL---AKYGVNLNEKTTRGYT  112
            HE  ++LLL          D  G + L+ +  AG++D++R L         N N +T    T
Sbjct  60   HEDIVRLLLAHEASTNLPDTRGSSPLHLSAWAGETDIVRLLLTQPYRPANANLQTIEQET  119

Query  113  LLHCAAAWGRLETLKALVELDVDIEALNFREERARDVAARYSQTECVEFLDWADARLTLK  172
             LHCAA  G    L  L+  D D    N R E   D+AA+Y + + V+ L  A   L
Sbjct  120  PLHCAAQHGHTGALALLLSHDADPNMRNSRGETPLDLAAQYGRLQAVQMLIRAHPEL---  176

Query  173  KYIAKVSLAVTDT  185
              I+ +S A  +T
Sbjct  177  --ISHLSTAAVET  187
```

```
>gb|AAA37742.1|  glucose-regulated protein 78
Length=168

 GENE ID: 14828 Hspa5 | heat shock protein 5 [Mus musculus]
(Over 100 PubMed links)

 Score = 55.1 bits (131),  Expect = 6e-06, Method: Compositional matrix adjust.
 Identities = 33/97 (34%), Positives = 54/97 (55%), Gaps = 1/97 (1%)

Query  144  ERARDVAARYSQTECVEFLDWADARLTLKKYIAKVSLAVTDTEKGSGKLLKEDKNTILSA  203
            ER  + A ++++ E   +   +   D R  L+ Y    +    + D EK   GKL  E K T+   A
Sbjct  53   ERMVNDAEKFAE-EDKKLKERIDTRNELESYAYSLKNQIGDKEKLGGKLSSEGKETMEKA  111

Query  204  CRAKNEWLETHTEASINELFEQRQQLEDIVTPIFTKM  240
               K EWLE+H +A I +   ++++LE+IV PI +K+
Sbjct  112  VEEKIEWLESHQDADIEDFKAKKKELEEIVQPIISKL  148


>emb|CAQ52951.1|  CD4-specific ankyrin repeat protein D5.1 [synthetic construct]
Length=169

 Score = 55.1 bits (131),  Expect = 7e-06, Method: Compositional matrix adjust.
 Identities = 29/82 (35%), Positives = 44/82 (53%), Gaps = 0/82 (0%)

Query  81   LYAACMAGQSDVIRALAKYGVNLNEKTTRGYTLLHCAAAWGRLETLKALVELDVDIEALN  140
            L+ A M G  +++  L KYG ++N    + GYT LH AAA+G LE ++ L++    D+ A +
Sbjct  51   LHLAAMGGHLEIVEVLLKYGADVNAWDSWGYTPLHLAAAYGHLEIVEVLLKNGADVNASD  110

Query  141  FREERARDVAARYSQTECVEFL  162
                    +AA      E VE L
Sbjct  111  IDGWTPLHLAASNGHLEIVEVL  132


 Score = 48.9 bits (115),  Expect = 5e-04, Method: Compositional matrix adjust.
 Identities = 32/87 (36%), Positives = 44/87 (50%), Gaps = 1/87 (1%)

Query  76   VGRNLLYAACMAGQSDVIRALAKYGVNLNEKTTRGYTLLHCAAAWGRLETLKALVELDVD  135
            +G+ LL AA  AGQ D +R L   G ++N      G T LH AA  G LE ++ L++   D
Sbjct  14   LGKKLLEAA-RAGQDDEVRILMANGADVNAFDANGITSLHLAAMGGHLEIVEVLLKYGAD  72

Query  136  IEALNFREERARDVAARYSQTECVEFL  162
            + A +         +AA Y   E VE L
Sbjct  73   VNAWDSWGYTPLHLAAAYGHLEIVEVL  99


 Score = 40.0 bits (92),  Expect = 0.26, Method: Compositional matrix adjust.
 Identities = 23/82 (28%), Positives = 41/82 (50%), Gaps = 0/82 (0%)

Query  81   LYAACMAGQSDVIRALAKYGVNLNEKTTRGYTLLHCAAAWGRLETLKALVELDVDIEALN  140
            L+ A    G  +++  L K G ++N     G+T LH AA+ G LE ++ L++    D+ A +
Sbjct  84   LHLAAAYGHLEIVEVLLKNGADVNASDIDGWTPLHLAASNGHLEIVEVLLKHGADVNAQD  143

Query  141  FREERARDVAARYSQTECVEFL  162
               + A D++       +  E L
Sbjct  144  KFGKTAFDISIDNGNKDLAEIL  165


>ref|XP_001602889.1|  PREDICTED: similar to IP14385p [Nasonia vitripennis]
Length=1357

 GENE ID: 100119039 LOC100119039 | similar to IP14385p [Nasonia vitripennis]

 Score = 55.1 bits (131),  Expect = 7e-06, Method: Compositional matrix adjust.
 Identities = 35/103 (33%), Positives = 53/103 (51%), Gaps = 7/103 (6%)

Query  63   HEQAMQLLLEEDIVGRNLLYAACMAGQSDVIRALAKYGV---NLNEKTTRGYTLLHCAAA  119
            HE +   ++     D  G + L+ A  +G S+++R +    G    N+N  T    T LHCAA
Sbjct  28   HEASTNII---DAKGSSPLHLAAWSGNSEIVRLILSQGPSVPNVNLTTKDNETALHCAAQ  84

Query  120  WGRLETLKALVELDVDIEALNFREERARDVAARYSQTECVEFL  162
            +G  E +  L++    D    N R E A D+AA+Y  + CVE L
Sbjct  85   YGHTEVVAQLLQYGCDPSIRNSRGESALDLAAQYG-SACVEVL  126


>gb|AAH93536.1|  LOC733200 protein [Xenopus laevis]
Length=895

 GENE ID: 733200 LOC733200 | hypothetical protein LOC733200 [Xenopus laevis]
(10 or fewer PubMed links)

 Score = 55.1 bits (131),  Expect = 7e-06, Method: Compositional matrix adjust.
 Identities = 30/74 (40%), Positives = 43/74 (58%), Gaps = 0/74 (0%)

Query  77   GRNLLYAACMAGQSDVIRALAKYGVNLNEKTTRGYTLLHCAAAWGRLETLKALVELDVDI  136
            G   L+ A    G ++V++ L + G ++N K    G+T LH AA WG+ E  K LVE   +I
Sbjct  72   GGTALHVAAAKGYAEVLKLLIQVGYDINVKDFDGWTPLHAAAHWGKEEACKILVENFCNI  131

Query  137  EALNFREERARDVA  150
            EA+N    + A DVA
Sbjct  132  EAVNKVGQTALDVA  145


>ref|XP_692620.3|  PREDICTED: similar to cask-interacting protein 2 [Danio rerio]
Length=1293

 GENE ID: 564179 CH211-119C20.3 | similar to cask-interacting protein 2
[Danio rerio]

 Score = 55.1 bits (131),  Expect = 7e-06, Method: Compositional matrix adjust.
 Identities = 32/100 (32%), Positives = 55/100 (55%), Gaps = 10/100 (10%)

Query  68   QLLLEEDIV-------GRN--LLYAACMAGQSDVIRALAKYGVNLNEKTTRGYTLLHCAA  118
            QLLL  ++V        GR+   L+ A    G  D+IR L K G+++N +TT+   T LH AA
Sbjct  165  QLLLNSNMVVALLEGNGRDNTPLHLAARNGHKDIIRLLLKAGIDIN-RTTKSGTALHEAA  223

Query  119  AWGRLETLKALVELDVDIEALNFREERARDVAARYSQTEC  158
```

```
             +G+ E +K L++  +D+   N   + A D+  +++ +
Sbjct  224  LYGKTEVVKLLLDAGIDVNIRNTYNQTALDIVNQFTTSHA  263


 Score = 35.0 bits (79),  Expect = 7.2, Method: Compositional matrix adjust.
 Identities = 24/103 (23%), Positives = 48/103 (46%), Gaps = 7/103 (6%)

Query  67   MQLLLE-------EDIVGRNLLYAACMAGQSDVIRALAKYGVNLNEKTTRGYTLLHCAAA  119
            + LLLE       +D  G   L+ A   G++D +   L + G ++N  +  G   LH AA
Sbjct  65   LSLLLEAQATVDIKDSNGMRPLHYAAWQGKADSVLLLLRAGASVNGASHDGQIPLHLAAQ  124

Query  120  WGRLETLKALVELDVDIEALNFREERARDVAARYSQTECVEFL  162
            +G  +   + L++    +    +N  ++    D+A   + + +   + L
Sbjct  125  YGHYDVSEMLLQHQSNPCTVNKVKKTPLDLACEFGRLKVTQLL  167


>ref|XP_001365714.1| PREDICTED: similar to glucose-regulated protein [Monodelphis
domestica]
Length=657

 GENE ID: 100015941 LOC100015941 | similar to glucose-regulated protein
[Monodelphis domestica]

 Score = 55.1 bits (131),  Expect = 8e-06, Method: Compositional matrix adjust.
 Identities = 29/75 (38%), Positives = 44/75 (58%), Gaps = 0/75 (0%)

Query  166  DARLTLKKYIAKVSLAVTDTEKGSGKLLKEDKNTILSACRAKNEWLETHTEASINELFEQ  225
            D+R  L+ Y    +    + D EK  GKL  EDK  +   A   K EWLE+H +A I +    +
Sbjct  564  DSRNELESYAYSLKNQIGDKEKLGGKLSSEDKEIVEKAVEEKIEWLESHQDAEIEDFKAK  623

Query  226  RQQLEDIVTPIFTKM  240
            +++LE+IV PI +K+
Sbjct  624  KKELEEIVQPIVSKL  638


>gb|ABK23212.1|  unknown [Picea sitchensis]
Length=347

 Score = 54.7 bits (130),  Expect = 9e-06, Method: Compositional matrix adjust.
 Identities = 37/111 (33%), Positives = 59/111 (53%), Gaps = 2/111 (1%)

Query  88   GQSDVIRALAKYGVNLNEKTTRGYTLLHCAAAWGRLETLKALVELDVDIEALNFREERAR  147
            G  + +R L K G + +EK T G T LH A  +G ++  + L+E  V ++AL+  +  A
Sbjct  234  GDVEGLRNLLKLGADKDEKDTEGRTALHFACGYGEVKCAEVLLEAGVMVDALDKNKNTAL  293

Query  148  DVAARYSQTECVEFLDWADARLTLKKYIAK--VSLAVTDTEKGSGKLLKED  196
              AA Y + ECV  L    A +TL+    K  + +A  + +K   KLL++D
Sbjct  294  HYAAGYGREECVGLLLKYGAAVTLQNLDGKTPIEVAKLNEQKEVLKLLEKD  344


>ref|XP_002001063.1| GI22180 [Drosophila mojavensis]
 gb|EDW16524.1| GI22180 [Drosophila mojavensis]
Length=2153

 GENE ID: 6575043 Dmoj\GI22180 | GI22180 gene product from transcript GI22180-RA
[Drosophila mojavensis] (10 or fewer PubMed links)

 Score = 54.7 bits (130),  Expect = 9e-06, Method: Compositional matrix adjust.
 Identities = 31/99 (31%), Positives = 53/99 (53%), Gaps = 9/99 (9%)

Query  37    NPLLQPALTGDVEGLQKIFEDPENPHHEQAMQLLLEEDIVGRNLLYAACMAGQSDVIRAL  96
             +PLL   +  G+ E   + + E+  +P           D +GR  L+AAC AG + V+R L
Sbjct  1488  SPLLVSSFEGNAEVCELLLENAADPD---------LADFMGRTPLWAACTAGHATVVRLL  1538

Query  97    AKYGVNLNEKTTRGYTLLHCAAAWGRLETLKALVELDVD  135
               +G  ++    + G T+L  AAA G +ET++ L++  +D
Sbjct  1539  LFWGCGIDCMDSEGRTVLSIAAAQGNVETVRQLLDRGLD  1577


 Score = 38.9 bits (89),  Expect = 0.49, Method: Compositional matrix adjust.
 Identities = 26/97 (26%), Positives = 47/97 (48%), Gaps = 8/97 (8%)

Query  63    HEQAMQLLLEE--------DIVGRNLLYAACMAGQSDVIRALAKYGVNLNEKTTRGYTLL  114
             H + ++LL+ +          D  GR  L AA  +G  D+++ L + G ++N    +G T L
Sbjct  1362  HSEVVRLLIAQPACKIDLADKEGRTALRAAAWSGHEDILKLLIESGADVNSVDRQGRTSL  1421

Query  115   HCAAAWGRLETLKALVELDVDIEALNFREERARDVAA  151
               A+  G  + ++ L+E   ++  L+     A  VAA
Sbjct  1422  IAASYMGHYDIVEILLENGANVNHLDLDGRSALCVAA  1458


 Score = 38.9 bits (89),  Expect = 0.53, Method: Compositional matrix adjust.
 Identities = 26/92 (28%), Positives = 44/92 (47%), Gaps = 1/92 (1%)

Query  77    GRNLLYAACMAGQSDVIRALAKYGVNLNEKTTRGYTLLHCAAAWGRLETLKALVEL-DVD  135
             G+     AC+ G  D +  L K+  ++N K      T L+  A    +L+T+K L+++ +VD
Sbjct  1652  GKTAFRLACLEGHMDTVEYLLKFCCDVNSKDADSRTTLYILALENKLDTVKYLLDMTNVD  1711

Query  136   IEALNFREERARDVAARYSQTECVEFLDWADA  167
             +   +      A  VAA      + V+ L  A A
Sbjct  1712  VNIPDSEGRTALHVAAWQGHADMVKTLIEAGA  1743


>ref|YP_920685.1| ankyrin [Thermofilum pendens Hrk 5]
 gb|ABL78682.1| Ankyrin [Thermofilum pendens Hrk 5]
Length=870

 GENE ID: 4600592 Tpen_1284 | ankyrin [Thermofilum pendens Hrk 5]
(10 or fewer PubMed links)

 Score = 54.7 bits (130),  Expect = 1e-05, Method: Composition-based stats.
 Identities = 36/96 (37%), Positives = 51/96 (53%), Gaps = 0/96 (0%)

Query  67   MQLLLEEDIVGRNLLYAACMAGQSDVIRALAKYGVNLNEKTTRGYTLLHCAAAWGRLETL  126
            ++LLLE       N L+AA   G+ + ++ L ++GVN N +    G TLLH AA  G +E +
Sbjct  489  IRLLLEHGAEPGNGLHAAVRCGRPECVKKLLEWGVNPNTRDNDGNTLLHAAAWNGDVEVI  548
```

```
Query  127  KALVELDVDIEALNFREERARDVAARYSQTECVEFL  162
             + L+E   DI A N   E    VAA      E V+ L
Sbjct  549  EILLERGADINARNKFGETPLHVAAERGNFEAVKLL  584
```

Score = 38.5 bits (88), Expect = 0.68, Method: Composition-based stats.
Identities = 36/133 (27%), Positives = 59/133 (44%), Gaps = 24/133 (18%)

```
Query  56   EDPENPHHE-------QAMQLLLEE--DIVGRN-----LLYAACMAGQSDVIRALAKYGV  101
            +D E P H+       +A++LLLE   D+  RN     L+ A    G  +++R L K+G
Sbjct  656  KDGETPLHKATSSGNVEAVRLLLEHGADVDARNDFGGTPLHHAAARGHLEIVRLLLKHGA  715

Query  102  NLNEKTTRGYTLLHCAA---------AWGR-LETLKALVELDVDIEALNFREERARDVAA  151
            + N + + G T LH  A         AW   L   + L+    D+ A + R++    +A
Sbjct  716  DSNARNSHGETPLHYVAEHADMCSKNAWDNCLRIAELLLIHGADVNARDSRDQTPLHIAV  775

Query  152  RYSQTECVEFLDW  164
             +   E +E   W
Sbjct  776  FFGSREHLEVARW  788
```

Score = 35.4 bits (80), Expect = 5.2, Method: Composition-based stats.
Identities = 37/143 (25%), Positives = 55/143 (38%), Gaps = 5/143 (3%)

```
Query  25   EEAQEPEETGPKN--PLLQPALTGDVEGLQKIFEDPENPHHEQAMQ---LLLEEDIVGRN  79
            E   +P   GP    PL   A+ G  E  + + E   +P+ +  +    L  E    GR
Sbjct  32   EGGVDPNAAGPAGLAPLHCAAIFGHAEAARLLLERGADPNVKDKITWDVLSSELGRKGRT  91

Query  80   LLYAACMAGQSDVIRALAKYGVNLNEKTTRGYTLLHCAAAWGRLETLKALVELDVDIEAL  139
             L+ A + G   V   L   G + N     G T LH AA  G   + + L++   D+ A
Sbjct  92   PLHWAAVYGHFVVAEVLLDRGADPNATDEEGNTPLHLAALLGFADIARLLLDRGADVNAK  151

Query  140  NFREERARDVAARYSQTECVEFL  162
            N   +     AA     E  + L
Sbjct  152  NSSGKTPLHYAAEQGSAEVAKLL  174
```

Score = 35.4 bits (80), Expect = 6.2, Method: Composition-based stats.
Identities = 19/55 (34%), Positives = 29/55 (52%), Gaps = 0/55 (0%)

```
Query  81   LYAACMAGQSDVIRALAKYGVNLNEKTTRGYTLLHCAAAWGRLETLKALVELDVD  135
            L+ A  +G +  ++AL + GV+ N     G   LHCAA +G  E  + L+E   D
Sbjct  15   LFRAVCSGDAKRVKALLEGGVDPNAAGPAGLAPLHCAAIFGHAEAARLLLERGAD  69
```

>**ref|XP_424401.2|** PREDICTED: hypothetical protein [Gallus gallus]
Length=2027

**GENE ID: 396311 ANK1** | ankyrin 1, erythrocytic [Gallus gallus]
(10 or fewer PubMed links)

Score = 54.7 bits (130), Expect = 1e-05, Method: Composition-based stats.
Identities = 31/93 (33%), Positives = 51/93 (54%), Gaps = 6/93 (6%)

```
Query  45   TGDVEGLQKIFEDPENPHHEQAMQLLLEEDIV------GRNLLYAACMAGQSDVIRALAK  98
            T +  GL  +    +  H +  ++LL +E ++      G   L+ A +AGQ DV+R L
Sbjct  78   TCNQNGLNALHLASKEGHTKMVVELLHKEIVLETTTKKGNTALHIAALAGQQDVVRELVN  137

Query  99   YGVNLNEKTTRGYTLLHCAAAWGRLETLKALVE  131
            YG N+N ++ +G+T L+ AA    LE +K L+E
Sbjct  138  YGANVNAQSQKGFTPLYMAAQENHLEVVKFLLE  170
```

Score = 43.1 bits (100), Expect = 0.028, Method: Composition-based stats.
Identities = 32/124 (25%), Positives = 54/124 (43%), Gaps = 7/124 (5%)

```
Query  63   HEQAMQLLLEED-------IVGRNLLYAACMAGQSDVIRALAKYGVNLNEKTTRGYTLLH  115
            H +  +LL+E+        + G   L+ AC      V+  L K G +++  T  G T LH
Sbjct  388  HHRVAKLLVEKGAKPNSRALNGFTPLHIACKKNHIRVMELLLKTGASIDAVTESGLTPLH  447

Query  116  CAAAWGRLETLKALVELDVDIEALNFREERARDVAARYSQTECVEFLDWADARLTLKKYI  175
             AA  G L  +K L++        N + E    +AAR    +  ++L   A++  K  +
Sbjct  448  VAAFMGHLPIVKTLLQRGASPNVSNVKVETPLHMAARAGHMDVAKYLIQNKAKINAKAKV  507

Query  176  AKVS  179
             + S
Sbjct  508  GRSS  511
```

>**ref|XP_001095508.1|** PREDICTED: similar to TRP (transient receptor potential) channel family member (trp-4) [Macaca mulatta]
Length=191

**GENE ID: 707084 LOC707084** | similar to TRP (transient receptor potential) channel family member (trp-4) [Macaca mulatta]

Score = 54.7 bits (130), Expect = 1e-05, Method: Compositional matrix adjust.
Identities = 31/86 (36%), Positives = 45/86 (52%), Gaps = 0/86 (0%)

```
Query  78   RNLLYAACMAGQSDVIRALAKYGVNLNEKTTRGYTLLHCAAAWGRLETLKALVELDVDIE  137
            R  L+ A + GQ +V+R L +YG      T+ G+T  H AA  G L  L+ L  L   I+
Sbjct  40   RTPLHWAAIKGQMEVLRLLIEYGARPCLVTSVGWTPAHFAAESGHLNVLRTLHALHAAID  99

Query  138  ALNFREERARDVAARYSQTECVEFLD  163
            A +F  +  + +A  Y Q  CV FL+
Sbjct  100  APDFFGDTPKRIAQIYGQKACVAFLE  125
```

>**ref|XP_001307066.1|** ankyrin repeat protein [Trichomonas vaginalis G3]
**gb|EAX94136.1|** ankyrin repeat protein, putative [Trichomonas vaginalis G3]
Length=759

**GENE ID: 4751856 TVAG_080420** | ankyrin repeat protein
[Trichomonas vaginalis G3] (10 or fewer PubMed links)

```
 Score = 54.7 bits (130),  Expect = 1e-05, Method: Compositional matrix adjust.
 Identities = 35/104 (33%), Positives = 52/104 (50%), Gaps = 2/104 (1%)

Query  72   EEDIVGRNLLYAACMAGQSDVIRALAKYGVNLNEKTTRGYTLLHCAAAWGRLETLKALVE  131
            E+D  GR  L+ A    + ++    L  +GVNLNE+   G T LH AA + ++ET + L+E
Sbjct  657  EKDNYGRTALHIAAQYNKKEIFELLLSHGVNLNERDKEGNTALHIAAQYNKIETAEFLIE  716

Query  132  LDVDIEALNFREERARDVAARYSQTECVEFLDWADARLTLKKYI  175
               +I    N      A  +A +Y+  E  E L     A  T+   YI
Sbjct  717  HGANINEKNNHGNTALYIAEQYNNKELAELLLSHGA--TINDYI  758


 Score = 48.1 bits (113),  Expect = 0.001, Method: Compositional matrix adjust.
 Identities = 29/93 (31%), Positives = 46/93 (49%), Gaps = 0/93 (0%)

Query  70   LLEEDIVGRNLLYAACMAGQSDVIRALAKYGVNLNEKTTRGYTLLHCAAAWGRLETLKAL  129
            L E+D   GR  L+ +        ++    L  +G N NEK   G T LH AA + + E  + L
Sbjct  622  LNEKDNYGRTALHISADYCYKEIFELLLSHGANFNEKDNYGRTALHIAAQYNKKEIFELL  681

Query  130  VELDVDIEALNFREERARDVAARYSQTECVEFL  162
            +   V++   +     A  +AA+Y++ E  EFL
Sbjct  682  LSHGVNLNERDKEGNTALHIAAQYNKIETAEFL  714


 Score = 47.4 bits (111),  Expect = 0.002, Method: Compositional matrix adjust.
 Identities = 40/143 (27%), Positives = 65/143 (45%), Gaps = 8/143 (5%)

Query  47   DVEGLQKIFEDPENPHHEQAMQLLL-------EEDIVGRNLLYAACMAGQSDVIRALAKY  99
            D +G   ++   EN + E A +LLL       E+D  G  +L  A  + + +  + L  +
Sbjct  527  DNDGKTALYIATENNNKEAA-ELLLSYGANINEKDNYGNTVLRIAAFSDKKETAKFLLSH  585

Query  100  GVNLNEKTTRGYTLLHCAAAWGRLETLKALVELDVDIEALNFREERARDVAARYSQTECV  159
            G N+NEK  +G T LH AA+  R E  + L+  DV++   +   +     A  ++A Y   E
Sbjct  586  GANINEKDNQGNTALHIAASHNRKEMAELLLSHDVNLNEKDNYGRTALHISADYCYKEIF  645

Query  160  EFLDWADARLTLKKYIAKVSLAV  182
            E L   A   K   + +L +
Sbjct  646  ELLLSHGANFNEKDNYGRTALHI  668


 Score = 47.4 bits (111),  Expect = 0.002, Method: Compositional matrix adjust.
 Identities = 38/130 (29%), Positives = 59/130 (45%), Gaps = 6/130 (4%)

Query  59   ENPHHEQAMQLLL------EEDIVGRNLLYAACMAGQSDVIRALAKYGVNLNEKTTRGYT  112
            EN + E A  LL       E+D  G+ +L+ A    ++   L  YG ++NEK  G T
Sbjct  275  ENNNKEIAELLLFYGANINEKDKDGKTVLHYAAENNNKEITEFLLLYGADINEKGEDGNT  334

Query  113  LLHCAAAWGRLETLKALVELDVDIEALNFREERARDVAARYSQTECVEFLDWADARLTLK  172
             LH AA    ETL  L+   +I   ++  + A ++A  +  E  E L +  A +  K
Sbjct  335  ALHYAAENNNKETLILLLSYGANINEKDYYGKTALNIALENNNKEIAELLLFYGANINEK  394

Query  173  KYIAKVSLAV  182
             Y  K +L +
Sbjct  395  DYYGKTALNI  404


 Score = 44.3 bits (103),  Expect = 0.012, Method: Compositional matrix adjust.
 Identities = 36/124 (29%), Positives = 56/124 (45%), Gaps = 7/124 (5%)

Query  67   MQLLL-------EEDIVGRNLLYAACMAGQSDVIRALAKYGVNLNEKTTRGYTLLHCAAA  119
            +QLL+       E+D  G+  LY A     +    L  YG N+NEK  G T+L  AA
Sbjct  513  IQLLITHGGNINEKDNDGKTALYIATENNNKEAAELLLSYGANINEKDNYGNTVLRIAAF  572

Query  120  WGRLETLKALVELDVDIEALNFREERARDVAARYSQTECVEFLDWADARLTLKKYIAKVS  179
              + ET K L+   +I   + +   A  +AA +++ E  E L  D  L  K  + +
Sbjct  573  SDKKETAKFLLSHGANINEKDNQGNTALHIAASHNRKEMAELLLSHDVNLNEKDNYGRTA  632

Query  180  LAVT  183
            L ++
Sbjct  633  LHIS  636


 Score = 37.4 bits (85),  Expect = 1.5, Method: Compositional matrix adjust.
 Identities = 32/111 (28%), Positives = 45/111 (40%), Gaps = 0/111 (0%)

Query  72   EEDIVGRNLLYAACMAGQSDVIRALAKYGVNLNEKTTRGYTLLHCAAAWGRLETLKALVE  131
            E+D  G+  L  A      ++   L  YG N+NEK  G T+LH AA     E  + L+
Sbjct  261  EKDYYGKTALNIALENNNKEIAELLLFYGANINEKDKDGKTVLHYAAENNNKEITEFLLL  320

Query  132  LDVDIEALNFREERARDVAARYSQTECVEFLDWADARLTLKKYIAKVSLAV  182
               DI       A    AA  +  E +  L    A +  K Y  K +L +
Sbjct  321  YGADINEKGEDGNTALHYAAENNNKETLILLLSYGANINEKDYYGKTALNI  371


 Score = 37.0 bits (84),  Expect = 1.8, Method: Compositional matrix adjust.
 Identities = 29/96 (30%), Positives = 44/96 (45%), Gaps = 10/96 (10%)

Query  72   EEDIVGRNLLYAACMAGQSDVIRALAKYGVNLNEKTTRGYTLLHCAAAWGRLETLKALVE  131
            E+D  G   L+ A    + ++   L  + VNLNEK   G T LH +A +   E  + L+
Sbjct  591  EKDNQGNTALHIAASHNRKEMAELLLSHDVNLNEKDNYGRTALHISADYCYKEIFELLLS  650

Query  132  LDVDIEALNFREE-----RARDVAARYSQTECVEFL  162
                  NF E+       A  +AA+Y++ E  E L
Sbjct  651  -----HGANFNEKDNYGRTALHIAAQYNKKEIFELL  681
```

>**ref|XP_001703461.1|** hypothetical protein CHLREDRAFT_127770 [Chlamydomonas reinhardtii]
**gb|AAO32623.1|** CR074 protein [Chlamydomonas reinhardtii]
**gb|EDP06143.1|** predicted protein [Chlamydomonas reinhardtii]
Length=368

**GENE ID: 5728898 ANK22a|ANK22b** | hypothetical protein
[Chlamydomonas reinhardtii] (Over 10 PubMed links)

```
 Score = 54.3 bits (129),  Expect = 1e-05, Method: Compositional matrix adjust.
 Identities = 32/104 (30%), Positives = 53/104 (50%), Gaps = 0/104 (0%)
```

```
Query  81   LYAACMAGQSDVIRALAKYGVNLNEKTTRGYTLLHCAAAWGRLETLKALVELDVDIEALN  140
            ++ A  AG  ++++ L   G N +E    G T LH AA +G LE ++ L++    ++A++
Sbjct  241  VHGAASAGDVELLKKLLAEGANADEADEEGRTALHFAAGYGELECVRMLIDAKAKLDAVD  300

Query  141  FREERARDVAARYSQTECVEFLDWADARLTLKKYIAKVSLAVTD  184
              +   A    AA Y Q E V+ L   + A   T K     K +L V +
Sbjct  301  TNQNTALHYAAGYGQAESVKILLSSGADRTSKNLDGKTALEVAE  344


>ref|XP_794552.2| PREDICTED: similar to ankyrin 2,3/unc44 [Strongylocentrotus purpuratus]
 ref|XP_001195269.1| PREDICTED: similar to ankyrin 2,3/unc44 [Strongylocentrotus purpuratus]
Length=3362

 GENE ID: 589826 LOC589826 | similar to ankyrin 2,3/unc44
[Strongylocentrotus purpuratus]

 Score = 54.3 bits (129),  Expect = 1e-05, Method: Compositional matrix adjust.
 Identities = 29/105 (27%), Positives = 53/105 (50%), Gaps = 5/105 (4%)

Query  63    HEQAMQLLLEED-----IVGRNLLYAACMAGQSDVIRALAKYGVNLNEKTTRGYTLLHCA  117
             H +A++ L+ E          GR  LYA+    G  D+++ L    G ++NE+    G    LH A
Sbjct  1911  HLEAVKYLIIEGAKQNRYAGRTPLYASAQCGHLDIVKFLVSKGADVNEEDDEGMIPLHGA  1970

Query  118   AAWGRLETLKALVELDVDIEALNFREERARDVAARYSQTECVEFL  162
             A+ G +E ++ L++    D+    + +       + A +Y    E V++L
Sbjct  1971  ASGGHVEVMEYLIQQGSDVNKADAKGWTPFNAAVQYGHLEAVKYL  2015


 Score = 52.8 bits (125),  Expect = 4e-05, Method: Compositional matrix adjust.
 Identities = 31/106 (29%), Positives = 51/106 (48%), Gaps = 0/106 (0%)

Query  77    GRNLLYAACMAGQSDVIRALAKYGVNLNEKTTRGYTLLHCAAAWGRLETLKALVELDVDI  136
             G    LYAA    G   D+I+ +    GV++NE+    +G T LH AAA G +E ++ L++    D+
Sbjct  2386  GMTPLYAAAQFGHLDIIKFVLSNGVDVNEEDDKGMTPLHGAAAGGHIEVIEYLIQQGSDV  2445

Query  137   EALNFREERARDVAARYSQTECVEFLDWADARLTLKKYIAKVSLAV  182
                + R        A        + V+FL     A  T   + +   + +A
Sbjct  2446  NKNDCRGWTPLHAAVTNGHLKVVQFLVAKGAHGTRYRGLTPLYIAT  2491


 Score = 48.1 bits (113),  Expect = 8e-04, Method: Compositional matrix adjust.
 Identities = 28/105 (26%), Positives = 50/105 (47%), Gaps = 5/105 (4%)

Query  63    HEQAMQLLLEED-----IVGRNLLYAACMAGQSDVIRALAKYGVNLNEKTTRGYTLLHCA  117
             H +A++ L+ E          G    LYAA    G  D+++ +    G ++NE+  +G    LH A
Sbjct  2008  HLEAVKYLMSEGAEQNRYDGMTPLYAAAQFGHLDIVKFVISKGADVNEEHNKGMIPLHGA  2067

Query  118   AAWGRLETLKALVELDVDIEALNFREERARDVAARYSQTECVEFL  162
             A   G ++ ++ L+E    D+    N          + A +    E V++L
Sbjct  2068  AVHGHVKVMEYLIEQGSDLNQANAEGGTPFNAAVQSGHLEAVKYL  2112


 Score = 47.4 bits (111),  Expect = 0.002, Method: Compositional matrix adjust.
 Identities = 28/91 (30%), Positives = 43/91 (47%), Gaps = 2/91 (2%)

Query  72    EEDIVGRNLLYAACMAGQSDVIRALAKYGVNLNEKTTRGYTLLHCAAAWGRLETLKALVE  131
             EED  G    L+ A    G  +VI   L + G ++N++    RG+T LH A    G L+ ++ LV
Sbjct  2414  EEDDKGMTPLHGAAAGGHIEVIEYLIQQGSDVNKNDCRGWTPLHAAVTNGHLKVVQFLVA  2473

Query  132   LDVDIEALNFREERARDVAARYSQTECVEFL  162
                        +R        +A +Y    + V+FL
Sbjct  2474  KGA--HGTRYRGLTPLYIATQYEHVDVVKFL  2502


 Score = 44.7 bits (104),  Expect = 0.010, Method: Compositional matrix adjust.
 Identities = 27/105 (25%), Positives = 48/105 (45%), Gaps = 5/105 (4%)

Query  63    HEQAMQLLLEED-----IVGRNLLYAACMAGQSDVIRALAKYGVNLNEKTTRGYTLLHCA  117
             H +A Q L+ +           G    LY+A + G  D++      GV++NE+    G    LH
Sbjct  2270  HLEAFQYLMTKGAELNRYDGMTPLYSASLYGHIDLVKFFISKGVDVNEENDAGRIPLHGT  2329

Query  118   AAWGRLETLKALVELDVDIEALNFREERARDVAARYSQTECVEFL  162
             A   G   E +K L++    D+    + +       + A +    E V++L
Sbjct  2330  AIHGNTEVMKYLIQQGSDVNKSDAKGWTPFNAAVQCGHLEAVKYL  2374


 Score = 43.9 bits (102),  Expect = 0.019, Method: Compositional matrix adjust.
 Identities = 33/129 (25%), Positives = 55/129 (42%), Gaps = 15/129 (11%)

Query  37    NPLLQPALTGDVEGLQKIFE---DPENPHHEQAMQLLLEEDIVGRNLLYAACMAGQSDVI  93
             +PL  + AL G +EG+Q +      DP  P                  G    L+AA    G    ++
Sbjct  1802  SPLYKAALEGHLEGVQDLISRGADPNKPSKG------------GLRPLHAAAQEGHRHIV  1849

Query  94    RALAKYGVNLNEKTTRGYTLLHCAAAWGRLETLKALVELDVDIEALNFREERARDVAARY  153
               L    G + NE+  +G T L   AAA G ++ ++ L+     ++    + +          A  Y
Sbjct  1850  ELLILQGADANEEDEKGNTPLLGAAAKGYMDVIECLIRQSSNVNRADDKGWTPFTAAVEY  1909

Query  154   SQTECVEFL  162
                  E V++L
Sbjct  1910  GHLEAVKYL  1918


 Score = 42.7 bits (99),  Expect = 0.033, Method: Compositional matrix adjust.
 Identities = 34/149 (22%), Positives = 65/149 (43%), Gaps = 26/149 (17%)

Query  38   PLLQPALTGDVEGLQKIFE---DPENPH--------------HEQAMQLLLEE--DI---  75
            PL + AL G+++ +Q +      +P NP                H    +  L+ +   D+
Sbjct  98   PLYKAALEGNLKDVQDLISRGANPNNPSKSGLRPLHAAAREGHAHIVDFLILQGADVSVE  157

Query  76   --VGRNLLYAACMAGQSDVIRALAKYGVNLNEKTTRGYTLLHCAAAWGRLETLKALVELD  133
              +G+   LY A    G  D++ +    G +LN++    G+T   + A    G L+ +K L+
Sbjct  158  CELGQTPLYTAANGYVDILESFIAEGSDLNQEDNTGWTSFNAAVQEGHLKAVKYLMTQG  217

Query  134  VDIEALNFREERARDVAARYSQTECVEFL  162
               +   ++ +     +AARY    E ++F
Sbjct  218  A--KRGRYKGKTPLYLAARYGHLEVIQFF  244
```

```
 Score = 42.7 bits (99),  Expect = 0.039, Method: Compositional matrix adjust.
 Identities = 29/115 (25%), Positives = 53/115 (46%), Gaps = 5/115 (4%)

Query  63    HEQAMQLLLEEDIVG---RNL--LYAACMAGQSDVIRALAKYGVNLNEKTTRGYTLLHCA  117
             H + +Q L+ +    G    R L  LY A       DV++ L    G ++NE+  RG + LH A
Sbjct  2464  HLKVVQFLVAKGAHGTRYRGLTPLYIATQYEHVDVVKFLVSSGYDVNERNERGKSPLHAA  2523

Query  118   AAWGRLETLKALVELDVDIEALNFREERARDVAARYSQTECVEFLDWADARLTLK  172
                G ++ +K LV  +  +   ++       + AA+    +  ++L     A + +K
Sbjct  2524  CYNGNMDIVKLLVHHNAHVNVQDYEGWTPLEAAAQEGHQDVEDYLALNGADMDVK  2578


 Score = 40.8 bits (94),  Expect = 0.13, Method: Compositional matrix adjust.
 Identities = 24/105 (22%), Positives = 49/105 (46%), Gaps = 5/105 (4%)

Query  63    HEQAMQLLLEED-----IVGRNLLYAACMAGQSDVIRALAKYGVNLNEKTTRGYTLLHCA  117
             H +A++ L+ E          G    L+AA    GQS +++      G ++N++    G    LH A
Sbjct  2173  HLEAVKYLMTEGAKQNRFNGMTPLFAAARFGQSHIVKFFMSNGADVNKENDNGRIPLHGA  2232

Query  118   AAWGRLETLKALVELDVDIEALNFREERARDVAARYSQTECVEFL  162
             A  G +E ++ L++    ++    + +       + A +     E  ++L
Sbjct  2233  AIRGHMEIMEYLIQQGSNVNKSDGKGWTPFNAAVQGGHLEAFQYL  2277


 Score = 39.7 bits (91),  Expect = 0.30, Method: Compositional matrix adjust.
 Identities = 24/78 (30%), Positives = 37/78 (47%), Gaps = 8/78 (10%)

Query  72   EEDIVGRNLLYAACMAGQSDVIRALAKYGVNLNEKTTRGYTLLHCAAAWGRLETLKALVE  131
            E +  G++ L+AAC   G   D+++ L      N+NE+    G+T L   AA   G   + +  L
Sbjct  538  ERNECGKSPLHAACYNGNMDIVKVLVHRKANVNEQDHDGWTPLEAAAQEGHQDIVNYLT-  596

Query  132  LDVDIEALNFREERARDV  149
                    LN   +    RDV
Sbjct  597  -------LNGADMNVRDV  607


 Score = 37.7 bits (86),  Expect = 1.2, Method: Compositional matrix adjust.
 Identities = 19/59 (32%), Positives = 32/59 (54%), Gaps = 0/59 (0%)

Query  72    EEDIVGRNLLYAACMAGQSDVIRALAKYGVNLNEKTTRGYTLLHCAAAWGRLETLKALV  130
             EED  G    L+ A    G  +V+  L + G ++N+    +G+T  + A  +G LE +K L+
Sbjct  1958  EEDDEGMIPLHGAASGGHVEVMEYLIQQGSDVNKADAKGWTPFNAAVQYGHLEAVKYLM  2016


>ref|NP_998223.1| heat shock 70kDa protein 5 [Danio rerio]
 gb|AAH52971.1| Heat shock protein 5 [Danio rerio]
Length=650

 GENE ID: 378848 hspa5 | heat shock protein 5 [Danio rerio]
(10 or fewer PubMed links)

 Score = 54.3 bits (129),  Expect = 1e-05, Method: Compositional matrix adjust.
 Identities = 34/97 (35%), Positives = 53/97 (54%), Gaps = 1/97 (1%)

Query  144  ERARDVAARYSQTECVEFLDWADARLTLKKYIAKVSLAVTDTEKGSGKLLKEDKNTILSA  203
            ER   + A R++  E   +  +  D+R  L+ Y   +   + D EK  GKL  EDK  I  A
Sbjct  537  ERMVNEAERFAD-EDKKLKERIDSRNELESYAYSLKNQIGDKEKLGGKLSSEDKEAIEKA  595

Query  204  CRAKNEWLETHTEASINELFEQRQQLEDIVTPIFTKM  240
               K EWLE H +A + E   ++++LE++V PI +K+
Sbjct  596  VEEKIEWLEAHQDADLEEFQAKKKELEEVVQPIVSKL  632


>ref|NP_195270.1| AKR2 (ANKYRIN REPEAT-CONTAINING PROTEIN 2); protein binding [Arabidopsis
thaliana]
 ref|NP_849497.1| AKR2 (ANKYRIN REPEAT-CONTAINING PROTEIN 2); protein binding [Arabidopsis
thaliana]
 ref|NP_849498.1| AKR2 (ANKYRIN REPEAT-CONTAINING PROTEIN 2); protein binding [Arabidopsis
thaliana]
 8 more sequence titles

sp|Q9SAR5.2|AKR2_ARATH RecName: Full=Ankyrin repeat domain-containing protein 2; Short=AtAKR2
 gb|AAK62427.1|AF386982_1 ankyrin repeat-containing protein 2 [Arabidopsis thaliana]
 emb|CAB54873.1| ankyrin repeat-containing protein 2 [Arabidopsis thaliana]
 emb|CAB80261.1| ankyrin repeat-containing protein 2 [Arabidopsis thaliana]
 gb|AAM10039.1| ankyrin repeat-containing protein 2 [Arabidopsis thaliana]
 gb|AAM64927.1| ankyrin repeat-containing protein 2 [Arabidopsis thaliana]
 dbj|BAH19543.1| AT4G35450 [Arabidopsis thaliana]
 dbj|BAH19853.1| AT4G35450 [Arabidopsis thaliana]
Length=342

 GENE ID: 829697 AKR2 | AKR2 (ANKYRIN REPEAT-CONTAINING PROTEIN 2); protein
binding [Arabidopsis thaliana] (10 or fewer PubMed links)

 Score = 54.3 bits (129),  Expect = 1e-05, Method: Compositional matrix adjust.
 Identities = 41/123 (33%), Positives = 54/123 (43%), Gaps = 9/123 (7%)

Query  41   QPALTGDVEGLQKIFEDPENPHHEQAMQLLLEEDIVGRNLLYAACMAGQSDVIRALAKYG  100
            Q A  GDVEGL+         N               EED  GR  L+ AC  G+     + L   G
Sbjct  224  QTASLGDVEGLKAALASGGNKD---------EEDSEGRTALHFACGYGELKCAQVLIDAG  274

Query  101  VNLNEKTTRGYTLLHCAAAWGRLETLKALVELDVDIEALNFREERARDVAARYSQTECVE  160
             ++N       T LH AA +GR E +  L+E    +  N  E+   DVA   SQ E V+
Sbjct  275  ASVNAVDKNKNTPLHYAAGYGRKECVSLLLENGAAVTLQNLDEKTPIDVAKLNSQLEVVK  334

Query  161  FLD  163
             L+
Sbjct  335  LLE  337


 Score = 40.0 bits (92),  Expect = 0.24, Method: Compositional matrix adjust.
```

```
 Identities = 38/167 (22%), Positives = 70/167 (41%), Gaps = 20/167 (11%)

Query  37    NPLLQPAL----TGDVEGLQKIFEDPENPHH-EQAMQL----LLEEDIVG----------  77
             +P L+P L     G    + K + DPE      +AM +    L ++ +
Sbjct  158   DPELKPILDEIDAGGPSAMMKYWNDPEVLKKLGEAMGMPVAGLPDQTVSAEPEVAEEGEE  217

Query  78    -RNLLYAACMAGQSDVIRALAKYGVNLNEKTTRGYTLLHCAAAWGRLETLKALVELDVDI  136
               ++++     G  + ++A    G N +E+ + G T LH A  +G L+  + L++    +
Sbjct  218   EESIVHQTASLGDVEGLKAALASGGNKDEEDSEGRTALHFACGYGELKCAQVLIDAGASV  277

Query  137   EALNFREERARDVAARYSQTECVEFLDWADARLTLKKYIAKVSLAVT  183
              A++  +      AA Y + ECV  L    A +TL+    K  + V
Sbjct  278   NAVDKNKNTPLHYAAGYGRKECVSLLLENGAAVTLQNLDEKTPIDVA  324


>ref|XP_001703462.1| hypothetical protein CHLREDRAFT_127770 [Chlamydomonas reinhardtii]
 gb|EDP06144.1| predicted protein [Chlamydomonas reinhardtii]
Length=389

 GENE ID: 5728898 ANK22a|ANK22b | hypothetical protein
[Chlamydomonas reinhardtii] (Over 10 PubMed links)

 Score = 54.3 bits (129),  Expect = 1e-05, Method: Compositional matrix adjust.
 Identities = 32/104 (30%), Positives = 53/104 (50%), Gaps = 0/104 (0%)

Query  81    LYAACMAGQSDVIRALAKYGVNLNEKTTRGYTLLHCAAAWGRLETLKALVELDVDIEALN  140
             ++ A  AG  ++++ L   G N +E    G T LH AA +G LE ++ L++    ++A++
Sbjct  262   VHGAASAGDVELLKKLLAEGANADEADEEGRTALHFAAGYGELECVRMLIDAKAKLDAVD  321

Query  141   FREERARDVAARYSQTECVEFLDWADARLTLKKYIAKVSLAVTD  184
               +  A   AA Y Q E V+ L  + A  T K    K +L V +
Sbjct  322   TNQNTALHYAAGYGQAESVKILLSSGADRTSKNLDGKTALEVAE  365


>gb|AAH63946.1| Heat shock protein 5 [Danio rerio]
 gb|AAT68067.1| immunoglobulin binding protein [Danio rerio]
Length=650

 GENE ID: 378848 hspa5 | heat shock protein 5 [Danio rerio]
(10 or fewer PubMed links)

 Score = 54.3 bits (129),  Expect = 1e-05, Method: Compositional matrix adjust.
 Identities = 34/97 (35%), Positives = 53/97 (54%), Gaps = 1/97 (1%)

Query  144   ERARDVAARYSQTECVEFLDWADARLTLKKYIAKVSLAVTDTEKGSGKLLKEDKNTILSA  203
             ER  + A R++  E  +  +  D+R  L+ Y   +   + D EK  GKL  EDK  I  A
Sbjct  537   ERMVNEAERFAD-EDKKLKERIDSRNELESYAYSLKNQIGDKEKLGGKLSSEDKEAIEKA  595

Query  204   CRAKNEWLETHTEASINELFEQRQQLEDIVTPIFTKM  240
                K EWLE H +A + E   ++++LE++V PI +K+
Sbjct  596   VEEKIEWLEAHQDADLEEFQAKKKELEEVVQPIVSKL  632


>emb|CAQ52949.1|  CD4-specific ankyrin repeat protein D3.1 [synthetic construct]
Length=169

 Score = 54.3 bits (129),  Expect = 1e-05, Method: Compositional matrix adjust.
 Identities = 29/82 (35%), Positives = 44/82 (53%), Gaps = 0/82 (0%)

Query  81    LYAACMAGQSDVIRALAKYGVNLNEKTTRGYTLLHCAAAWGRLETLKALVELDVDIEALN  140
             L+ A M G  +++  L KYG ++N   + GYT LH AAA+G LE ++ L++   D+ A +
Sbjct  51    LHLAAMEGHLEIVEVLLKYGADVNAWDSWGYTPLHLAAAYGHLEIVEVLLKKGADVNASD  110

Query  141   FREERARDVAARYSQTECVEFL  162
                     +AA      E VE L
Sbjct  111   IDGWTPLHLAASNGHLEIVEVL  132


 Score = 48.1 bits (113),  Expect = 9e-04, Method: Compositional matrix adjust.
 Identities = 32/87 (36%), Positives = 44/87 (50%), Gaps = 1/87 (1%)

Query  76    VGRNLLYAACMAGQSDVIRALAKYGVNLNEKTTRGYTLLHCAAAWGRLETLKALVELDVD  135
             +G+ LL AA  AGQ D +R L   G ++N     G T LH AA  G LE ++ L++   D
Sbjct  14    LGKKLLEAA-RAGQDDEVRILMANGADVNAFDANGITSLHLAAMEGHLEIVEVLLKYGAD  72

Query  136   IEALNFREERARDVAARYSQTECVEFL  162
             + A +        +AA Y   E VE L
Sbjct  73    VNAWDSWGYTPLHLAAAYGHLEIVEVL  99


 Score = 39.7 bits (91),  Expect = 0.30, Method: Compositional matrix adjust.
 Identities = 23/82 (28%), Positives = 41/82 (50%), Gaps = 0/82 (0%)

Query  81    LYAACMAGQSDVIRALAKYGVNLNEKTTRGYTLLHCAAAWGRLETLKALVELDVDIEALN  140
             L+ A   G  +++  L K G ++N     G+T LH AA+ G LE ++ L++   D+ A +
Sbjct  84    LHLAAAYGHLEIVEVLLKKGADVNASDIDGWTPLHLAASNGHLEIVEVLLKHGADVNAQD  143

Query  141   FREERARDVAARYSQTECVEFL  162
                + A D++      +  E L
Sbjct  144   KFGKTAFDISIDNGNEDLAEIL  165


>pdb|1SVX|A Chain A, Crystal Structure Of A Designed Selected Ankyrin Repeat
Protein In Complex With The Maltose Binding Protein
 gb|AAQ93810.1|  ankyrin repeat protein off7 [synthetic construct]
Length=169

 Score = 54.3 bits (129),  Expect = 1e-05, Method: Compositional matrix adjust.
 Identities = 28/89 (31%), Positives = 47/89 (52%), Gaps = 0/89 (0%)

Query  74    DIVGRNLLYAACMAGQSDVIRALAKYGVNLNEKTTRGYTLLHCAAAWGRLETLKALVELD  133
             D  G   L+ A  +G  +++  L K+G +++     GYT LH AA WG LE ++ L++
Sbjct  44    DNTGTTPLHLAAYSGHLEIVEVLLKHGADVDASDVFGYTPLHLAAYWGHLEIVEVLLKNG  103

Query  134   VDIEALNFREERARDVAARYSQTECVEFL  162
              D+ A++        +AA++   E VE L
```

```
Sbjct  104  ADVNAMDSDGMTPLHLAAKWGYLEIVEVL  132


 Score = 47.8 bits (112),  Expect = 0.001, Method: Compositional matrix adjust.
 Identities = 32/87 (36%), Positives = 45/87 (51%), Gaps = 1/87 (1%)

Query  76   VGRNLLYAACMAGQSDVIRALAKYGVNLNEKTTRGYTLLHCAAAWGRLETLKALVELDVD  135
            +GR LL AA  AGQ D +R L   G ++N     G T LH AA  G LE ++ L++   D
Sbjct  14   LGRKLLEAA-RAGQDDEVRILMANGADVNAADNTGTTPLHLAAYSGHLEIVEVLLKHGAD  72

Query  136  IEALNFREERARDVAARYSQTECVEFL  162
            ++A +         +AA +   E VE L
Sbjct  73   VDASDVFGYTPLHLAAYWGHLEIVEVL  99


 Score = 46.6 bits (109),  Expect = 0.002, Method: Compositional matrix adjust.
 Identities = 26/89 (29%), Positives = 44/89 (49%), Gaps = 0/89 (0%)

Query  74   DIVGRNLLYAACMAGQSDVIRALAKYGVNLNEKTTRGYTLLHCAAAWGRLETLKALVELD  133
            D+ G   L+ A   G  +++  L K G ++N   + G T LH AA WG LE ++ L++
Sbjct  77   DVFGYTPLHLAAYWGHLEIVEVLLKNGADVNAMDSDGMTPLHLAAKWGYLEIVEVLLKHG  136

Query  134  VDIEALNFREERARDVAARYSQTECVEFL  162
             D+ A +   + A D++      +  E L
Sbjct  137  ADVNAQDKFGKTAFDISIDNGNEDLAEIL  165


>ref|XP_002137001.1|  GA26825 [Drosophila pseudoobscura pseudoobscura]
 gb|EDY67559.1|  GA26825 [Drosophila pseudoobscura pseudoobscura]
Length=1044

 GENE ID: 6896797 Dpse\GA26825 | GA26825 gene product from transcript GA26825-RA
[Drosophila pseudoobscura pseudoobscura] (10 or fewer PubMed links)

 Score = 54.3 bits (129),  Expect = 1e-05, Method: Compositional matrix adjust.
 Identities = 38/117 (32%), Positives = 54/117 (46%), Gaps = 10/117 (8%)

Query  63   HEQAMQLLLEEDIV-------GRNLLYAACMAGQSDVIRALAKYG---VNLNEKTTRGYT  112
            HE  ++LLL  D          G + L+ A  AG++D++R L  +        N +T    T
Sbjct  61   HEDIVRLLLANDASPNLPDSRGSSPLHLAAWAGETDIVRLLLTHPYRPATANLQTIEQET  120

Query  113  LLHCAAAWGRLETLKALVELDVDIEALNFREERARDVAARYSQTECVEFLDWADARL  169
             LHCAA  G    L  L+  D D    N R E   D+AA+Y + + V+ L  A   L
Sbjct  121  PLHCAAQHGHTGALALLLGHDADPNMRNSRGETPLDLAAQYGRLQAVQMLIRAHPEL  177


>ref|XP_313120.4|  AGAP004215-PA [Anopheles gambiae str. PEST]
 gb|EAA08632.4|  AGAP004215-PA [Anopheles gambiae str. PEST]
Length=1986

 GENE ID: 1274056 AgaP AGAP004215 | AGAP004215-PA [Anopheles gambiae str. PEST]
(10 or fewer PubMed links)

 Score = 54.3 bits (129),  Expect = 1e-05, Method: Compositional matrix adjust.
 Identities = 40/141 (28%), Positives = 68/141 (48%), Gaps = 18/141 (12%)

Query  38    PLLQPALTGDVEGLQKIFEDPENPHHEQAMQLLLEEDIVGRNLLYAACMAGQSDVIRALA  97
             PLL   +  G+ E  + + E+  +P      M          GR  L+AAC +G ++V++ L
Sbjct  1304  PLLVSSFEGNAEICELLLENGADPDMADNM---------GRTPLWAACTSGHANVVKLLL  1354

Query  98    KYGVNLNEKTTRGYTLLHCAAAWGRLETLKALVELDVDIEALNFREERARDVAARYSQTE  157
              +G  ++    + G T+L   AAA G LET++ L++  +D          E  RD A  ++
Sbjct  1355  FWGCGIDCMDSEGRTVLSVAAAQGNLETVRQLLDRGLD--------ETHRDNAG-WTPLH  1405

Query  158   CVEFLDWADARLTLKKYIAKV  178
                F  +AD   + L +  AK+
Sbjct  1406  YAAFEGYADICVQLLESGAKI  1426


 Score = 43.1 bits (100),  Expect = 0.028, Method: Compositional matrix adjust.
 Identities = 27/97 (27%), Positives = 46/97 (47%), Gaps = 8/97 (8%)

Query  63    HEQAMQLLLEEDIV--------GRNLLYAACMAGQSDVIRALAKYGVNLNEKTTRGYTLL  114
             H + ++LL+E  +          GR  L AA  +G  D+++ L + G N+N    +G T L
Sbjct  1177  HTEVVKLLIETGVCAIDRADKEGRTALRAAAWSGNEDIVKILIEAGANVNSIDKQGRTSL  1236

Query  115   HCAAAWGRLETLKALVELDVDIEALNFREERARDVAA  151
               A+  G  + ++ L+E   D+   +      A  VAA
Sbjct  1237  IAASYMGHYDIVEILLENGADVNHTDLDGRNALCVAA  1273


>gb|AAA28074.1|  BiP, heat shock protein 3
Length=661

 Score = 53.9 bits (128),  Expect = 1e-05, Method: Compositional matrix adjust.
 Identities = 39/101 (38%), Positives = 52/101 (51%), Gaps = 5/101 (4%)

Query  163  DWADARLTLKKYIAKVSLAVTDTEKGSGKLLKEDKNTILSACRAKNEWLETHTEASINEL  222
            D A+AR   L+ Y   +   + D EK  GKL ++DK TI  A       WL ++ EAS  EL
Sbjct  562  DKAEARNELESYAYNLKNQIEDKEKLGGKLDEDDKKTIEEAVEEAISWLGSNAEASAEEL  621

Query  223  FEQRQQLEDIVTPIFTKMTTPCQVKSAKSVTSHDQKRSQDD  263
             EQ++ LE   V PI +K+       K A +      QKR  DD
Sbjct  622  KEQKKDLESKVQPIVSKL-----YKDAGAGERRPQKRDLDD  657


>emb|CAG12424.1|  unnamed protein product [Tetraodon nigroviridis]
Length=668

 Score = 53.9 bits (128),  Expect = 2e-05, Method: Compositional matrix adjust.
 Identities = 29/75 (38%), Positives = 42/75 (56%), Gaps = 0/75 (0%)

Query  166  DARLTLKKYIAKVSLAVTDTEKGSGKLLKEDKNTILSACRAKNEWLETHTEASINELFEQ  225
            DAR  L+ Y    +   + D EK  GKL  EDK TI  A      EW+E+H E+    +    +
Sbjct  557  DARNELESYAYSLKNQIGDKEKLGGKLSDEDKETIEKAVEETIEWMESHQESETEDFQAK  616
```

```
Query  226  RQQLEDIVTPIFTKM  240
            ++ LED+V PI +K+
Sbjct  617  KKSLEDVVQPIISKL  631


>dbj|BAE31621.1|  unnamed protein product [Mus musculus]
Length=655

 GENE ID: 14828 Hspa5 | heat shock protein 5 [Mus musculus]
(Over 100 PubMed links)

 Score = 53.9 bits (128),  Expect = 2e-05, Method: Compositional matrix adjust.
 Identities = 29/75 (38%), Positives = 43/75 (57%), Gaps = 0/75 (0%)

Query  166  DARLTLKKYIAKVSLAVTDTEKGSGKLLKEDKNTILSACRAKNEWLETHTEASINELFEQ  225
            D R  L+ Y   +   +  D EK  GKL  EDK T+  A   K EWL +H +A I +    +
Sbjct  561  DTRNELESYAYSLKNQIGDKEKLGGKLSSEDKETMEKAVEEKIEWLGSHQDADIEDFKAK  620

Query  226  RQQLEDIVTPIFTKM  240
            +++LE+IV PI +K+
Sbjct  621  KKELEEIVQPIISKL  635


>pdb|2BKG|A  Chain A, Crystal Structure Of E3_19 An Designed Ankyrin Repeat
Protein
 pdb|2BKG|B  Chain B, Crystal Structure Of E3_19 An Designed Ankyrin Repeat
Protein
 gb|AAO25690.1|  ankyrin repeat protein E3_19 [synthetic construct]
Length=166

 Score = 53.9 bits (128),  Expect = 2e-05, Method: Compositional matrix adjust.
 Identities = 34/87 (39%), Positives = 48/87 (55%), Gaps = 1/87 (1%)

Query  76   VGRNLLYAACMAGQSDVIRALAKYGVNLNEKTTRGYTLLHCAAAWGRLETLKALVELDVD  135
            +G+ LL AA  AGQ D +R L   G ++N + T G T LH AA  G LE ++ L++   D
Sbjct  14   LGKKLLEAA-RAGQDDEVRILMANGADVNAEDTYGDTPLHLAARVGHLEIVEVLLKNGAD  72

Query  136  IEALNFREERARDVAARYSQTECVEFL  162
            + AL+F        +AA+    E VE L
Sbjct  73   VNALDFSGSTPLHLAAKRGHLEIVEVL  99


 Score = 45.1 bits (105),  Expect = 0.008, Method: Compositional matrix adjust.
 Identities = 27/89 (30%), Positives = 43/89 (48%), Gaps = 0/89 (0%)

Query  74   DIVGRNLLYAACMAGQSDVIRALAKYGVNLNEKTTRGYTLLHCAAAWGRLETLKALVELD  133
            D  G   L+ A   G  +++  L KYG ++N   T G T LH AA  G LE ++ L++
Sbjct  77   DFSGSTPLHLAAKRGHLEIVEVLLKYGADVNADDTIGSTPLHLAADTGHLEIVEVLLKYG  136

Query  134  VDIEALNFREERARDVAARYSQTECVEFL  162
             D+ A +   + A D++      +  E L
Sbjct  137  ADVNAQDKFGKTAFDISIDNGNEDLAEIL  165


>ref|XP_794262.1|  PREDICTED: similar to ankyrin 2,3/unc44, partial [Strongylocentrotus
purpuratus]
 ref|XP_001199940.1|  PREDICTED: similar to ankyrin 2,3/unc44, partial [Strongylocentrotus
purpuratus]
Length=1790

 GENE ID: 589530 LOC589530 | similar to ankyrin 2,3/unc44
[Strongylocentrotus purpuratus]

 Score = 53.9 bits (128),  Expect = 2e-05, Method: Compositional matrix adjust.
 Identities = 30/105 (28%), Positives = 52/105 (49%), Gaps = 5/105 (4%)

Query  63   HEQAMQLLL-----EEDIVGRNLLYAACMAGQSDVIRALAKYGVNLNEKTTRGYTLLHCA  117
            H +A++ L+     +    GR  LYAA   G  D+++      G ++NE+  +G   LH A
Sbjct  221  HLEAVEYLMTKGAKQNRYAGRTPLYAAAQLGHLDIVKFFISKGADVNEEDEKGEIPLHGA  280

Query  118  AAWGRLETLKALVELDVDIEALNFREERARDVAARYSQTECVEFL  162
            A+ G LE +K L++    ++ N        + A +Y   E V++L
Sbjct  281  ASGGHLEVMKYLIQQGSNVNKANSEGWTPFNAAVQYGHLEAVKYL  325


 Score = 52.4 bits (124),  Expect = 5e-05, Method: Compositional matrix adjust.
 Identities = 43/171 (25%), Positives = 73/171 (42%), Gaps = 29/171 (16%)

Query  14   FFSQQEEENEEEEAQEPEETGPKNPLLQPALTGDVEGLQKIFEDPENPH-----------  62
            F S   + NEE+E  E        PL   A+ G+VE +  + +   + +
Sbjct  454  FISNGADVNEEDEEGEI-------PLHGAAIDGNVEVMAYLIQQGSDTNKCDADGWTPFN  506

Query  63   ------HEQAMQLLL-----EEDIVGRNLLYAACMAGQSDVIRALAKYGVNLNEKTTRGY  111
                  H ++++ L+     +    GR  LYAA   G  D++R      G ++NEK   G
Sbjct  507  AAIQYGHLESVKYLITKGAKQNRYAGRTPLYAAAQLGHLDIVRLFISNGADVNEKDEEGE  566

Query  112  TLLHCAAAWGRLETLKALVELDVDIEALNFREERARDVAARYSQTECVEFL  162
              LH AA  G +E +K L++  D+  ++       + A +  Q E V++L
Sbjct  567  IPLHGAANDGNVEVIKYLIQQGSDVNKMDAEGWTPFNAAVQEGQLEAVKYL  617


 Score = 49.3 bits (116),  Expect = 4e-04, Method: Compositional matrix adjust.
 Identities = 25/86 (29%), Positives = 44/86 (51%), Gaps = 0/86 (0%)

Query  77    GRNLLYAACMAGQSDVIRALAKYGVNLNEKTTRGYTLLHCAAAWGRLETLKALVELDVDI  136
             G   LYAA  +G  D+++      G ++NE+  +G   LH AA  G LE ++ L++   D+
Sbjct  1211  GMTPLYAAAQSGCLDIVKFFISNGADVNEEHDKGMIPLHGAAHRGHLEVMEYLIQQGADV  1270

Query  137   EALNFREERARDVAARYSQTECVEFL  162
                + +   + +  A +    E VE+L
Sbjct  1271  NKADAKGGTSFNAAVQGGHLEAVEYL  1296


 Score = 48.1 bits (113),  Expect = 8e-04, Method: Compositional matrix adjust.
 Identities = 25/86 (29%), Positives = 44/86 (51%), Gaps = 0/86 (0%)
```

```
Query  77    GRNLLYAACMAGQSDVIRALAKYGVNLNEKTTRGYTLLHCAAAWGRLETLKALVELDVDI  136
             G   LYAA  +G  D+++     G ++NE+   G   L  AAA G+LE ++ L++   D+
Sbjct  1017  GMTPLYAAAQSGHLDIVKFFISEGADVNEEDEEGMIPLRGAAAGGQLEVMEYLIQQGADV  1076

Query  137   EALNFREERARDVAARYSQTECVEFL  162
                + +   + + A +    E VE+L
Sbjct  1077  NKADAKGGTSFNAAVQGGHLEAVEYL  1102


 Score = 47.8 bits (112),  Expect = 0.001, Method: Compositional matrix adjust.
 Identities = 25/86 (29%), Positives = 44/86 (51%), Gaps = 0/86 (0%)

Query  77   GRNLLYAACMAGQSDVIRALAKYGVNLNEKTTRGYTLLHCAAAWGRLETLKALVELDVDI  136
            G   LYAA  +G  D+++     G ++NE+   G   L  AAA G+LE ++ L++   D+
Sbjct  726  GMTPLYAAAQSGHLDIVKFFISEGADVNEEDEEGMIPLRGAAAGGQLEVMEYLIQQGSDV  785

Query  137  EALNFREERARDVAARYSQTECVEFL  162
               + +   + + A +    E VE+L
Sbjct  786  NKADAKGGTSFNAAVQGGHLEAVEYL  811


 Score = 47.4 bits (111),  Expect = 0.001, Method: Compositional matrix adjust.
 Identities = 25/86 (29%), Positives = 44/86 (51%), Gaps = 0/86 (0%)

Query  77    GRNLLYAACMAGQSDVIRALAKYGVNLNEKTTRGYTLLHCAAAWGRLETLKALVELDVDI  136
             G   LYAA  +G  D+++     G ++NE+  R    LH AA  G+LE ++ L++   D+
Sbjct  1114  GMTPLYAAAQSGCLDIVKFFISNGADVNEEHARRMIPLHGAAHRGQLEVMEYLIQQGADV  1173

Query  137   EALNFREERARDVAARYSQTECVEFL  162
                + +   + + A +    E VE+L
Sbjct  1174  NKADAKGGTSFNAAVQGGHLEAVEYL  1199


 Score = 46.2 bits (108),  Expect = 0.003, Method: Compositional matrix adjust.
 Identities = 25/86 (29%), Positives = 42/86 (48%), Gaps = 0/86 (0%)

Query  77    GRNLLYAACMAGQSDVIRALAKYGVNLNEKTTRGYTLLHCAAAWGRLETLKALVELDVDI  136
             G   LYAA  +G  D+++     G ++NE+  +G   LH AA  G LE ++ L++   D
Sbjct  920   GMTPLYAAAQSGCLDIVKFFISNGADVNEEHDKGMIPLHGAACEGHLEVMEYLIQQGSDT  979

Query  137   EALNFREERARDVAARYSQTECVEFL  162
                +       + A +Y   E V++L
Sbjct  980   NKCDAEGWTPFNAAVQYGHLESVKYL  1005


 Score = 45.1 bits (105),  Expect = 0.008, Method: Compositional matrix adjust.
 Identities = 25/86 (29%), Positives = 42/86 (48%), Gaps = 0/86 (0%)

Query  77   GRNLLYAACMAGQSDVIRALAKYGVNLNEKTTRGYTLLHCAAAWGRLETLKALVELDVDI  136
            G   LYAA  +G+ D+++     G ++NE+  R    LH AA  G LE ++ L++   D
Sbjct  629  GMTPLYAAAQSGRLDIVKFFISNGADVNEEDDRRKIPLHGAACEGHLEVMEYLIQQGSDT  688

Query  137  EALNFREERARDVAARYSQTECVEFL  162
               +       + A +Y   E V++L
Sbjct  689  NKCDAEGWTPFNAAVQYGHLESVKYL  714


 Score = 44.3 bits (103),  Expect = 0.012, Method: Compositional matrix adjust.
 Identities = 24/86 (27%), Positives = 44/86 (51%), Gaps = 0/86 (0%)

Query  77   GRNLLYAACMAGQSDVIRALAKYGVNLNEKTTRGYTLLHCAAAWGRLETLKALVELDVDI  136
            G   LYAA  +G  D+++     G ++NE+  R    L+ AA  G+LE ++ L++   D+
Sbjct  823  GMTPLYAAAQSGCLDIVKFFISNGADVNEEHARRMIPLNGAAHRGQLEVMEYLIQQGADV  882

Query  137  EALNFREERARDVAARYSQTECVEFL  162
               + +   + + A +    E VE+L
Sbjct  883  NKADAKGGTSFNAAVQGGHLEAVEYL  908


 Score = 42.4 bits (98),  Expect = 0.047, Method: Compositional matrix adjust.
 Identities = 20/60 (33%), Positives = 33/60 (55%), Gaps = 0/60 (0%)

Query  77    GRNLLYAACMAGQSDVIRALAKYGVNLNEKTTRGYTLLHCAAAWGRLETLKALVELDVDI  136
             G   LYAA  +G  D+++     G ++NE+  +G   LH AA  G LE ++ L++   D+
Sbjct  1308  GMTPLYAAAQSGCLDIVKFFISNGADVNEEHDKGMIPLHGAAHRGHLEVMEYLIQQGADV  1367


 Score = 40.8 bits (94),  Expect = 0.13, Method: Compositional matrix adjust.
 Identities = 24/86 (27%), Positives = 39/86 (45%), Gaps = 0/86 (0%)

Query  77   GRNLLYAACMAGQSDVIRALAKYGVNLNEKTTRGYTLLHCAAAWGRLETLKALVELDVDI  136
            G+  LYAA      D++R     G ++NE+   G   LH AA  G +E +  L++   D
Sbjct  435  GKAPLYAAAKCSHLDIVRLFISNGADVNEEDEEGEIPLHGAAIDGNVEVMAYLIQQGSDT  494

Query  137  EALNFREERARDVAARYSQTECVEFL  162
               +       + A +Y   E V++L
Sbjct  495  NKCDADGWTPFNAAIQYGHLESVKYL  520


 Score = 40.4 bits (93),  Expect = 0.21, Method: Compositional matrix adjust.
 Identities = 39/172 (22%), Positives = 73/172 (42%), Gaps = 30/172 (17%)

Query  14   FFSQQEEENEEEEAQEPEETGPKNPLLQPALTGDVEGLQKIFEDPENPH-----------  62
            F S+  + NEE+E  E        PL   A  G +E ++ + +   N +
Sbjct  259  FISKGADVNEEDEKGEI-------PLHGAASGGHLEVMKYLIQQGSNVNKANSEGWTPFN  311

Query  63   ------HEQAMQLLLEEDIV-----GRNLLYAACMAGQSDVIRALAKYGVNLNEKTTRGY  111
                  H +A++ L+ +        G   LYAA  +G  ++++ +   G ++NE+  +
Sbjct  312  AAVQYGHLEAVKYLMTKGATQNRYNGMTPLYAAAQSGHLNIVQFVISKGADVNEEHDKRM  371

Query  112  TLLHCAAAWGRL-ETLKALVELDVDIEALNFREERARDVAARYSQTECVEFL  162
              LH AA+   L E +K L++   ++   N +     + A +Y   E V+ L
Sbjct  372  IPLHGAASGAHLIEVMKYLIQQGSNVNKTNLKGWTPFNAAVQYGYLEAVKCL  423


 Score = 37.7 bits (86),  Expect = 1.1, Method: Compositional matrix adjust.
 Identities = 18/50 (36%), Positives = 29/50 (58%), Gaps = 0/50 (0%)
```

```
Query  81    LYAACMAGQSDVIRALAKYGVNLNEKTTRGYTLLHCAAAWGRLETLKALV  130
             L+ A   G  +V+  L + G ++N+K   G+T LH A + G LE +K L+
Sbjct  1345  LHGAAHRGHLEVMEYLIQQGADVNKKDNTGWTPLHAAVSNGHLEVVKVLL  1394
```

**>ref|XP_001663294.1|** hypothetical protein AaeL_AAEL013079 [Aedes aegypti]
**gb|EAT34709.1|** conserved hypothetical protein [Aedes aegypti]
Length=1890

**GENE ID: 5577199 AaeL AAEL013079** | hypothetical protein [Aedes aegypti]
(10 or fewer PubMed links)

```
 Score = 53.9 bits (128),  Expect = 2e-05, Method: Compositional matrix adjust.
 Identities = 40/141 (28%), Positives = 68/141 (48%), Gaps = 18/141 (12%)

Query  38    PLLQPALTGDVEGLQKIFEDPENPHHEQAMQLLLEEDIVGRNLLYAACMAGQSDVIRALA  97
             PLL  +  G+ E  + + E+  +P     M          GR  L+AAC +G ++V++ L
Sbjct  1200  PLLVSSFEGNSEICELLLENGADPDMADHM---------GRTPLWAACTSGHANVVKLLL  1250

Query  98    KYGVNLNEKTTRGYTLLHCAAAWGRLETLKALVELDVDIEALNFREERARDVAARYSQTE  157
              +G  ++    + G T+L  AAA G LET++ L++  +D        E  RD A  ++
Sbjct  1251  FWGCGIDCMDSEGRTVLSVAAAQGNLETVRQLLDRGLD--------ETHRDNAG-WTPLH  1301

Query  158   CVEFLDWADARLTLKKYIAKV  178
                F  +AD  + L +  AK+
Sbjct  1302  YAAFEGYADICIQLLESGAKI  1322


 Score = 41.6 bits (96),  Expect = 0.078, Method: Compositional matrix adjust.
 Identities = 28/97 (28%), Positives = 45/97 (46%), Gaps = 8/97 (8%)

Query  63    HEQAMQLLLEE--------DIVGRNLLYAACMAGQSDVIRALAKYGVNLNEKTTRGYTLL  114
             H   ++LL+E         D  GR  L AA  +G  D+++ L + G N+N    +G T L
Sbjct  1073  HTDVVKLLIESGACAIDRADKEGRTALRAAAWSGNEDIVKILIEAGANVNSIDKQGRTSL  1132

Query  115   HCAAAWGRLETLKALVELDVDIEALNFREERARDVAA  151
               A+  G  + ++ L+E   D+   +      A  VAA
Sbjct  1133  IAASYMGHYDIVEILLENGADVNHTDLDGRNALCVAA  1169


 Score = 39.7 bits (91),  Expect = 0.33, Method: Compositional matrix adjust.
 Identities = 24/87 (27%), Positives = 42/87 (48%), Gaps = 1/87 (1%)

Query  77    GRNLLYAACMAGQSDVIRALAKYGVNLNEKTTRGYTLLHCAAAWGRLETLKALVEL-DVD  135
             G+      AC+ G   + ++ L K+G ++N K      T L+   A    +L+ +K L+E  +VD
Sbjct  1363  GKTAFRLACLEGHFECVQTLLKFGCDVNSKDADSRTTLYILALENKLKVVKFLLEYSNVD  1422

Query  136   IEALNFREERARDVAARYSQTECVEFL  162
             +    +      A   VA+      E V+ L
Sbjct  1423  VNIPDSEGRTALHVASWQGHAEMVKLL  1449
```

**>ref|XP_001179198.1|** PREDICTED: similar to ankyrin 2,3/unc44 [Strongylocentrotus purpuratus]
**ref|XP_001189921.1|** PREDICTED: similar to ankyrin 2,3/unc44 [Strongylocentrotus purpuratus]
Length=1597

**GENE ID: 752470 LOC752470** | similar to ankyrin 2,3/unc44
[Strongylocentrotus purpuratus]

```
 Score = 53.9 bits (128),  Expect = 2e-05, Method: Compositional matrix adjust.
 Identities = 40/153 (26%), Positives = 66/153 (43%), Gaps = 22/153 (14%)

Query  38   PLLQPALTGDVEGLQKIFEDPENPH-----------------HEQAMQLLLEEDIV----  76
            PL    A+ GD+E LQ +      N +                     H +A++ L+ E
Sbjct  589  PLHGAAIKGDIEMLQYLIHQRCNVNKKDNTGMTPLIAAVQNGHLEAVKYLMSEGAKQNRY  648

Query  77   -GRNLLYAACMAGQSDVIRALAKYGVNLNEKTTRGYTLLHCAAAWGRLETLKALVELDVD  135
             G +  Y+A ++G  D+++      GV +NE+   G   LH AA  G  E +K LV+   D
Sbjct  649  DGMSPPYSAALSGHGDLVKFFISKGVGVNEENDTGRIPLHAAAIHGNNEVMKYLVQQGSD  708

Query  136  IEALNFREERARDVAARYSQTECVEFLDWADAR  168
            +    + +      + A +Y   E V++L     AR
Sbjct  709  VNKSDAKGWTPFNAAVQYGHLEAVKYLMSEGAR  741


 Score = 46.2 bits (108),  Expect = 0.003, Method: Compositional matrix adjust.
 Identities = 33/125 (26%), Positives = 56/125 (44%), Gaps = 9/125 (7%)

Query  38   PLLQPALTGDVEGLQKIFEDPENPHHEQAMQLLLEEDIVGRNLLYAACMAGQSDVIRALA  97
            PL   AL G +EG++ +     NP+             G   L+AA   G + ++  L
Sbjct  135  PLYNAALEGHLEGVEDLISRGANPNKPSKG---------GLRPLHAAVQEGHTRIVDFLI  185

Query  98   KYGVNLNEKTTRGYTLLHCAAAWGRLETLKALVELDVDIEALNFREERARDVAARYSQTE  157
              G ++N +   R     LH AAA G L+ L++LV  + ++   +       + A +Y   E
Sbjct  186  LQGADVNIECERSTRPLHTAAAKGYLDILESLVAEETNVNMEDHTGWTPFNTAVQYGHLE  245

Query  158  CVEFL  162
            V++L
Sbjct  246  AVKYL  250


 Score = 46.2 bits (108),  Expect = 0.003, Method: Compositional matrix adjust.
 Identities = 29/105 (27%), Positives = 47/105 (44%), Gaps = 5/105 (4%)

Query  63   HEQAMQLLLEED-----IVGRNLLYAACMAGQSDVIRALAKYGVNLNEKTTRGYTLLHCA  117
            H +A++ L+ E            G   LYAA   G  D++       G ++NE+  +G    LH A
Sbjct  728  HLEAVKYLMSEGARQNKYYGMTPLYAAARFGHLDIVEFFISNGADVNEEDDKGRIPLHGA  787

Query  118  AAWGRLETLKALVELDVDIEALNFREERARDVAARYSQTECVEFL  162
            A  G ++ L+ L++    D+    +         D A +Y   E V  L
Sbjct  788  AINGNIDILEYLIQQGYDLNKRDTTGWTPFDAAVQYGHLEAVNCL  832


 Score = 45.4 bits (106),  Expect = 0.005, Method: Compositional matrix adjust.
 Identities = 29/105 (27%), Positives = 48/105 (45%), Gaps = 5/105 (4%)
```

```
Query  63   HEQAMQLLLEED-----IVGRNLLYAACMAGQSDVIRALAKYGVNLNEKTTRGYTLLHCA  117
            H +A++ L+ E        G   LYAA   G  D+++       V++NE+   G   LH A
Sbjct  243  HLEAVKYLITEGAKQNRYAGVTPLYAAARFGHLDIVKFFISKEVDVNEENDSGRIPLHGA  302

Query  118  AAWGRLETLKALVELDVDIEALNFREERARDVAARYSQTECVEFL  162
            +  G  E +K LV+   D+   +       + A +Y   E V++L
Sbjct  303  SIHGSTEVMKYLVQQGSDVNKSSATGWTPFNAAVQYGHLEAVKYL  347


 Score = 45.4 bits (106),  Expect = 0.006, Method: Compositional matrix adjust.
 Identities = 31/111 (27%), Positives = 50/111 (45%), Gaps = 5/111 (4%)

Query  63   HEQAMQLLLEEDIV-----GRNLLYAACMAGQSDVIRALAKYGVNLNEKTTRGYTLLHCA  117
            H +A++ L+ E        G   LYAA   G  D+++       V++NE+   G   LH A
Sbjct  340  HLEAVKYLMSEGAKQNRYDGVTPLYAAARFGHLDIVKFFISKEVDVNEENDSGRIPLHGA  399

Query  118  AAWGRLETLKALVELDVDIEALNFREERARDVAARYSQTECVEFLDWADAR  168
            +  G  E +K LV+   D+   +       + A +Y   E V++L    AR
Sbjct  400  SIHGSTEVMKYLVQQGSDVNKSSATGWTPFNAAVQYGHLEAVKYLMSEGAR  450


 Score = 43.1 bits (100),  Expect = 0.026, Method: Compositional matrix adjust.
 Identities = 21/59 (35%), Positives = 33/59 (55%), Gaps = 0/59 (0%)

Query  72   EEDIVGRNLLYAACMAGQSDVIRALAKYGVNLNEKTTRGYTLLHCAAAWGRLETLKALV  130
            EED  GR  L+ A + G  D++  L + G +LN++ T G+T    A  +G LE +  L+
Sbjct  775  EEDDKGRIPLHGAAINGNIDILEYLIQQGYDLNKRDTTGWTPFDAAVQYGHLEAVNCLM  833


 Score = 43.1 bits (100),  Expect = 0.027, Method: Compositional matrix adjust.
 Identities = 26/96 (27%), Positives = 49/96 (51%), Gaps = 12/96 (12%)

Query  72   EEDIVGRNLLYAACMAGQSDVIRALAKYGVNLNEKTTRGYTLLHCAAAWGRLETLKALVE  131
            EE+  GR  L+ A + G ++V++ L + G ++N+ +  G+T  + A  +G LE +K L+
Sbjct  290  EENDSGRIPLHGASIHGSTEVMKYLVQQGSDVNKSSATGWTPFNAAVQYGHLEAVKYLM-  348

Query  132  LDVDIEALNFREERARDV-----AARYSQTECVEFL  162
                  +   ++ R   V     AAR+   + V+F
Sbjct  349  ------SEGAKQNRYDGVTPLYAAARFGHLDIVKFF  378


 Score = 41.2 bits (95),  Expect = 0.11, Method: Compositional matrix adjust.
 Identities = 28/113 (24%), Positives = 52/113 (46%), Gaps = 0/113 (0%)

Query  81   LYAACMAGQSDVIRALAKYGVNLNEKTTRGYTLLHCAAAWGRLETLKALVELDVDIEALN  140
            LYAA + G  D++      G N+N++        LH AA+ G +E ++ L++   D+   +
Sbjct  848  LYAAAVFGHLDLVIHFISKGANVNQEDENEKIPLHGAASGGHIEVMEYLIQQGSDVNKND  907

Query  141  FREERARDVAARYSQTECVEFLDWADARLTLKKYIAKVSLAVTDTEKGSGKLL  193
             R       A++    E V+ L    A++T    ++ + +A  +      KLL
Sbjct  908  CRGWTPLRAASKNGHLEGVKLLMAKGAKITRLHGMSPLYIASYNGNMDIVKLL  960


 Score = 39.7 bits (91),  Expect = 0.29, Method: Compositional matrix adjust.
 Identities = 26/107 (24%), Positives = 47/107 (43%), Gaps = 6/107 (5%)

Query  63   HEQAMQLLLEED-----IVGRNLLYAACMAGQSDVIRALAKYGVNLNEKTTRGYTLLHCA  117
            H +A++ L+ E        G   LYAA   G  D++      G ++N +   G   LH A
Sbjct  437  HLEAVKYLMSEGARQNKYYGMTPLYAAARFGHLDIVEFFISNGADVNGEDDEGMIPLHGA  496

Query  118  AAWGRLETLKALVELDVDIEALNFREERARDVAARYSQTECVEFLDW  164
            A  G L+ ++ L++   ++   +       + A +Y     V++L W
Sbjct  497  ACGGHLKVMEYLIQQGSEMNKKDKSGWIPFNAAVQYGHLPAVKYL-W  542


 Score = 38.1 bits (87),  Expect = 1.0, Method: Compositional matrix adjust.
 Identities = 26/92 (28%), Positives = 42/92 (45%), Gaps = 13/92 (14%)

Query  63    HEQAMQLLLEED-----IVGRNLLYAACMAGQSDVIRALAKYGVNLNEKTTRGYTLLHCA  117
             H + ++LL+ +      + G + LY A   G  D+++ L  +  N+NE+   G+T L  A
Sbjct  922   HLEGVKLLMAKGAKITRLHGMSPLYIASYNGNMDIVKLLVHHNANVNEQDNDGWTPLEAA  981

Query  118   AAWGRLETLKALVELDVDIEALNFREERARDV  149
             A  G  + +  L        ALN      RDV
Sbjct  982   AQEGHQDIVNYL--------ALNGAGMHVRDV  1005
```

>**ref|XP_002188655.1|** PREDICTED: ankyrin repeat domain 42 [Taeniopygia guttata]
Length=652

 **GENE ID: 100229712 LOC100229712** | ankyrin repeat domain 42
[Taeniopygia guttata]

```
 Score = 53.9 bits (128),  Expect = 2e-05, Method: Compositional matrix adjust.
 Identities = 32/80 (40%), Positives = 43/80 (53%), Gaps = 1/80 (1%)

Query  84   ACMAGQSDVIRALAKYGV-NLNEKTTRGYTLLHCAAAWGRLETLKALVELDVDIEALNFR  142
            A   G   V+R L + GV N+NE+  +G TLLH AA  G +  L+ LVE+  D +  N
Sbjct  387  AAFNGDLLVVRRLVRGGVVNINERNDKGSTLLHKAAEQGHIHCLQWLVEMGADCDITNDA  446

Query  143  EERARDVAARYSQTECVEFL  162
             E  +DVA R+     VE L
Sbjct  447  GETPKDVAKRFGHLAAVELL  466
```

>**ref|NP_001088961.1|** hypothetical protein LOC496341 [Xenopus laevis]
 **gb|AAH88937.1|** LOC496341 protein [Xenopus laevis]
Length=1085

 **GENE ID: 496341 LOC496341** | hypothetical LOC496341 [Xenopus laevis]
(10 or fewer PubMed links)

```
 Score = 53.9 bits (128),  Expect = 2e-05, Method: Compositional matrix adjust.
 Identities = 40/130 (30%), Positives = 60/130 (46%), Gaps = 18/130 (13%)

Query  39    LLQPALTGDVEGLQKIF---EDPENPHHEQAMQLLLEEDIVGRNLLYAACMAGQSDVIRA  95
             LL  +L G+ + +Q+I    EDP  P+ E            G   L+ A  AG   +++
```

```
Sbjct  887   LLDASLEGEFDLVQRIIYEVEDPSKPNDE------------GITPLHNAVCAGHHHIVKF  934

Query  96    LAKYGVNLNEKTTRGYTLLHCAAAWGRLETLKALVELDVDIEALNFRE-ERARDVAARYS  154
             L  +GVN+N    + G+T LHCAA+    +   K LVE     I A     + E A D
Sbjct  935   LLDFGVNVNAADSDGWTPLHCAASCNSVHLCKMLVECGAAIFATTISDVETAADKCEEME  994

Query  155   Q--TECVEFL  162
             +    +C +FL
Sbjct  995   EGYIQCSQFL  1004
```

>**ref|NP_001087157.1|** MGC83480 protein [Xenopus laevis]
 **gb|AAH78098.1|** MGC83480 protein [Xenopus laevis]
Length=1071

 **GENE ID: 447046 MGC83480** | MGC83480 protein [Xenopus laevis]
(10 or fewer PubMed links)

```
 Score = 53.9 bits (128),  Expect = 2e-05, Method: Compositional matrix adjust.
 Identities = 34/103 (33%), Positives = 50/103 (48%), Gaps = 15/103 (14%)

Query  39   LLQPALTGDVEGLQKIF---EDPENPHHEQAMQLLLEEDIVGRNLLYAACMAGQSDVIRA  95
            LL   +L G+ + +Q+I     EDP  P+ E            G   L+ A  AG    +++
Sbjct  873  LLDASLEGEFDLVQRIIYEVEDPSKPNDE------------GITPLHNAVCAGHHHIVKF  920

Query  96   LAKYGVNLNEKTTRGYTLLHCAAAWGRLETLKALVELDVDIEA  138
            L   +GVN+N    + G+T LHCAA+    +   K LVE     I A
Sbjct  921  LIDFGVNVNAADSDGWTPLHCAASCNSVHLCKMLVECGAAIFA  963
```

>**gb|AAW25061.1|** SJCHGC02512 protein [Schistosoma japonicum]
Length=199

```
 Score = 53.9 bits (128),  Expect = 2e-05, Method: Compositional matrix adjust.
 Identities = 37/126 (29%), Positives = 61/126 (48%), Gaps = 4/126 (3%)

Query  18   QEEENEEEEAQEPE-ETGPKNPLLQPALTGDVEGLQKIFEDPENPHHEQAM---QLLLEE  73
            Q +EN+  +   E + +   P     +  A    D+  +  + E  +     E  M     QLLL +
Sbjct  7    QADENDINQWTEDQIKHDPNKQFIVAAENNDLATILSLIESAKQKGCEGEMEFKQLLLAK  66

Query  74   DIVGRNLLYAACMAGQSDVIRALAKYGVNLNEKTTRGYTLLHCAAAWGRLETLKALVELD  133
            D   G     L+ A     G   +V++ L K G ++N +T   G+T LH AA W +L   ++ L+
Sbjct  67   DQDGYTALHRAAYGGHIEVLQCLIKCGADVNNRTEDGWTPLHSAAFWNKLACVQLLISAG  126

Query  134  VDIEAL  139
             D+ AL
Sbjct  127  ADLNAL  132
```

>**dbj|BAD15288.1|** 78kDa glucose regulated protein [Crassostrea gigas]
Length=661

```
 Score = 53.9 bits (128),  Expect = 2e-05, Method: Compositional matrix adjust.
 Identities = 28/70 (40%), Positives = 43/70 (61%), Gaps = 0/70 (0%)

Query  171  LKKYIAKVSLAVTDTEKGSGKLLKEDKNTILSACRAKNEWLETHTEASINELFEQRQQLE  230
            L+ Y    +     + D EK   GKL   EDK TI   A    K +W+E++ +A + + +L   Q++LE
Sbjct  570  LESYAYSLKNQIGDKEKLGGKLSDEDKKTIEEAVDEKIKWMESNADAEVEDLKAQKKELE  629

Query  231  DIVTPIFTKM  240
            +IV PI TK+
Sbjct  630  EIVQPIMTKL  639
```

>**sp|Q96KQ4.2|ASPP1_HUMAN** RecName: Full=Apoptosis-stimulating of p53 protein 1; AltName: Full=Protein phosphatase 1 regulatory subunit 13B
 **emb|CAC83011.2|** ASPP1 protein [Homo sapiens]
Length=1090

 **GENE ID: 23368 PPP1R13B** | protein phosphatase 1, regulatory (inhibitor) subunit 13B [Homo sapiens] (Over 10 PubMed links)

```
 Score = 53.5 bits (127),  Expect = 2e-05, Method: Compositional matrix adjust.
 Identities = 50/177 (28%), Positives = 78/177 (44%), Gaps = 20/177 (11%)

Query  39    LLQPALTGDVEGLQKIF---EDPENPHHEQAMQLLLEEDIVGRNLLYAACMAGQSDVIRA  95
             LL   +L G+ + +Q+I     EDP  P+ E            G   L+ A  AG    +++
Sbjct  892   LLDASLEGEFDLVQRIIYEVEDPSKPNDE------------GITPLHNAVCAGHHHIVKF  939

Query  96    LAKYGVNLNEKTTRGYTLLHCAAAWGRLETLKALVELDVDIEALNFRE-ERARDVAARYS  154
             L  +GVN+N    + G+T LHCAA+    +   K LVE     I A     + E A D
Sbjct  940   LLDFGVNVNAADSDGWTPLHCAASCNSVHLCKQLVESGAAIFASTISDIETAADKCEEME  999

Query  155   Q--TECVEFLDWADARLTLKKYIAKVSLAVTDTEKGSGKLLKEDKNTILSACRAKNE  209
             +    +C +FL       +L +         V+ A+ D E  +    L   +    L+   R K+E
Sbjct  1000  EGYIQCSQFLYGVQEKLGVMN--KGVAYALWDYEAQNSDELSFHEGDALTILRRKDE  1054
```

>**ref|NP_056131.2|** apoptosis-stimulating protein of p53, 1 [Homo sapiens]
 **gb|EAW81847.1|** protein phosphatase 1, regulatory (inhibitor) subunit 13B, isoform CRA_c [Homo sapiens]
 **gb|AAI36528.1|** Protein phosphatase 1, regulatory (inhibitor) subunit 13B [Homo sapiens]
Length=1090

 **GENE ID: 23368 PPP1R13B** | protein phosphatase 1, regulatory (inhibitor) subunit 13B [Homo sapiens] (Over 10 PubMed links)

```
 Score = 53.5 bits (127),  Expect = 2e-05, Method: Compositional matrix adjust.
 Identities = 50/177 (28%), Positives = 78/177 (44%), Gaps = 20/177 (11%)

Query  39    LLQPALTGDVEGLQKIF---EDPENPHHEQAMQLLLEEDIVGRNLLYAACMAGQSDVIRA  95
             LL  +L G+ + +Q+I     EDP  P+ E            G   L+ A  AG    +++
Sbjct  892   LLDASLEGEFDLVQRIIYEVEDPSKPNDE------------GITPLHNAVCAGHHHIVKF  939
```

```
Query  96    LAKYGVNLNEKTTRGYTLLHCAAAWGRLETLKALVELDVDIEALNFRE-ERARDVAARYS  154
             L  +GVN+N   + G+T LHCAA+   +   K LVE    I A    + E A D
Sbjct  940   LLDFGVNVNAADSDGWTPLHCAASCNSVHLCKQLVESGAAIFASTISDIETAADKCEEME  999

Query  155   Q--TECVEFLDWADARLTLKKYIAKVSLAVTDTEKGSGKLLKEDKNTILSACRAKNE  209
             +   +C +FL     +L +      V+ A+ D E  +   L   +   L+  R K+E
Sbjct  1000  EGYIQCSQFLYGVQEKLGVMN--KGVAYALWDYEAQNSDELSFHEGDALTILRRKDE  1054
```

>**ref|XP_001844143.1|** ankyrin repeat domain-containing protein 50 [Culex quinquefasciatus]
**gb|EDS35996.1|** ankyrin repeat domain-containing protein 50 [Culex quinquefasciatus]
Length=1901

**GENE ID: 6033723 CpipJ_CPIJ002399** | ankyrin repeat domain-containing protein 50 [Culex quinquefasciatus]

Score = 53.5 bits (127), Expect = 2e-05, Method: Compositional matrix adjust.
Identities = 40/141 (28%), Positives = 68/141 (48%), Gaps = 18/141 (12%)

```
Query  38    PLLQPALTGDVEGLQKIFEDPENPHHEQAMQLLLEEDIVGRNLLYAACMAGQSDVIRALA  97
             PLL  +  G+ E  + + E+  +P     M         GR  L+AAC +G ++V++ L
Sbjct  1441  PLLVSSFEGNSEICELLLENGADPDMADHM---------GRTPLWAACTSGHANVVKLLL  1491

Query  98    KYGVNLNEKTTRGYTLLHCAAAWGRLETLKALVELDVDIEALNFREERARDVAARYSQTE  157
              +G  ++   + G T+L  AAA G LET++ L++  +D        E  RD A  ++
Sbjct  1492  FWGCGIDCMDSEGRTVLSVAAAQGNLETVRQLLDRGLD--------ETHRDNAG-WTPLH  1542

Query  158   CVEFLDWADARLTLKKYIAKV  178
                F  +AD  + L +  AK+
Sbjct  1543  YAAFEGYADICIQLLESGAKI  1563
```

Score = 41.6 bits (96), Expect = 0.073, Method: Compositional matrix adjust.
Identities = 28/97 (28%), Positives = 45/97 (46%), Gaps = 8/97 (8%)

```
Query  63    HEQAMQLLLEE--------DIVGRNLLYAACMAGQSDVIRALAKYGVNLNEKTTRGYTLL  114
             H   ++LL+E         D  GR  L AA  +G  D+++ L + G N+N    +G T L
Sbjct  1314  HTDVVKLLIESGSCAIDRADKEGRTALRAAAWSGNEDIVKILIEAGANVNSIDKQGRTSL  1373

Query  115   HCAAAWGRLETLKALVELDVDIEALNFREERARDVAA  151
               A+  G  + ++ L+E   D+   +     A  VAA
Sbjct  1374  IAASYMGHYDIVEILLESGADVNHTDLDGRNALCVAA  1410
```

Score = 41.6 bits (96), Expect = 0.085, Method: Compositional matrix adjust.
Identities = 25/87 (28%), Positives = 42/87 (48%), Gaps = 1/87 (1%)

```
Query  77    GRNLLYAACMAGQSDVIRALAKYGVNLNEKTTRGYTLLHCAAAWGRLETLKALVEL-DVD  135
             G+     AC+ G  + ++ L KYG ++N K     T L+  A   +L+ +K L+E  +VD
Sbjct  1604  GKTAFRLACLEGHFECVQTLLKYGCDVNSKDADSRTTLYILALENKLKVVKFLLEYSNVD  1663

Query  136   IEALNFREERARDVAARYSQTECVEFL  162
             +   +     A  VA+     E V+ L
Sbjct  1664  VNVPDSEGRSALHVASWQGHAEMVKLL  1690
```

>**ref|XP_001374556.1|** PREDICTED: similar to ankyrin repeat domain 16 [Monodelphis domestica]
Length=371

**GENE ID: 100022831 LOC100022831** | similar to ankyrin repeat domain 16 [Monodelphis domestica]

Score = 53.5 bits (127), Expect = 2e-05, Method: Compositional matrix adjust.
Identities = 41/118 (34%), Positives = 56/118 (47%), Gaps = 10/118 (8%)

```
Query  55   FEDPENPHHEQAMQLLL--------EEDIVGRNLLYAACMAGQSDVIRAL-AKYGVNLNE  105
            F D     H    QLLL         +DI+G   L+ A + GQ   I+ L A  GV ++E
Sbjct  224  FMDAIQCGHIDIAQLLLVIHEACFTAKDILGAQALHKAAVTGQDKAIQFLVASLGVGVDE  283

Query  106  KTTRG-YTLLHCAAAWGRLETLKALVELDVDIEALNFREERARDVAARYSQTECVEFL  162
            + T   YT LH AA  G   T+K L+ L  ++EA + R   A  +A       C+EFL
Sbjct  284  RVTSTQYTALHHAAEEGHTSTVKTLLSLGAELEAKDGRNRSALHLACAGQHKTCIEFL  341
```

>**ref|NP_001093487.1|** ankyrin repeat and sterile alpha motif domain containing 1B [Danio rerio]
**sp|A5PMU4.1|ANS1B_DANRE** RecName: Full=Ankyrin repeat and sterile alpha motif domain-containing protein 1B
**emb|CAN88710.1|** novel protein similar to human ankyrin repeat and sterile alpha motif domain containing 1A (ANKS1A) [Danio rerio]
**emb|CAN88193.1|** novel protein similar to human ankyrin repeat and sterile alpha motif domain containing 1A (ANKS1A) [Danio rerio]
**emb|CAN88370.1|** novel protein similar to human ankyrin repeat and sterile alpha motif domain containing 1A (ANKS1A) [Danio rerio]
**emb|CAN87827.1|** novel protein similar to human ankyrin repeat and sterile alpha motif domain containing 1A (ANKS1A) [Danio rerio]
Length=1280

**GENE ID: 565408 anks1b** | ankyrin repeat and sterile alpha motif domain containing 1B [Danio rerio]

Score = 53.5 bits (127), Expect = 2e-05, Method: Compositional matrix adjust.
Identities = 42/152 (27%), Positives = 70/152 (46%), Gaps = 18/152 (11%)

```
Query  38   PLLQPALTGDVEGLQKIFEDPENPHHEQAMQLLLEEDIVGRNLLYAACMAGQSDVIRALA  97
            PL   A  GDV+ +Q +       HH  +   + E+++     L+ A   G S+V+R L
Sbjct  94   PLHLAAWRGDVDIVQILI------HHGPSHSRVNEQNLEKETALHCAAQYGHSEVVRVLL  147

Query  98   KYGVNLNEKTTRGYTLLHCAAAWGRLETLKALVELDVDIEALNFREERARDVAARYSQTE  157
            +   + + + +RG T L  AA +GRL+ ++ L+    ++ + N R+     +AAR
Sbjct  148  QELTDPSMRNSRGETPLDLAALYGRLQVVRMLLTAHPNLMSCNTRKHTPLHLAARNGHYA  207

Query  158  CVEFLDWADARLTLKKYIAKVSLAVTDTEKGS  189
             V+ L  AD  +             T TEKGS
```

```
Sbjct  208  TVQVLLEADMDVN------------TQTEKGS  227
```

```
 Score = 38.1 bits (87),  Expect = 0.81, Method: Compositional matrix adjust.
 Identities = 25/92 (27%), Positives = 42/92 (45%), Gaps = 3/92 (3%)

Query  74   DIVGRNLLYAACMAGQSDVIRALAKYGVNLNEKTTRGYTLLHCAAAWGRLETLKALVELD  133
            D  G   L+ A + G  DV+  L ++  + N   ++G   LH AA  G ++ ++ L+
Sbjct  55   DGSGYTPLHHASLNGHRDVVLKLLQFEASTNVSDSKGCFPLHLAAWRGDVDIVQILIHHG  114

Query  134  VD---IEALNFREERARDVAARYSQTECVEFL  162
                 +   N  +E A   AA+Y  +E V  L
Sbjct  115  PSHSRVNEQNLEKETALHCAAQYGHSEVVRVL  146
```

>**emb|CAI11564.1|** novel protein similar to human and mouse CASK interacting protein 2 (CASKIN2) [Danio rerio]
Length=413

**GENE ID: 564253 CH211-119C20.2** | similar to cask-interacting protein 2 [Danio rerio]

```
 Score = 53.5 bits (127),  Expect = 2e-05, Method: Compositional matrix adjust.
 Identities = 32/100 (32%), Positives = 55/100 (55%), Gaps = 10/100 (10%)

Query  68   QLLLEEDIV-------GRN--LLYAACMAGQSDVIRALAKYGVNLNEKTTRGYTLLHCAA  118
            QLLL  ++V       GR+   L+ A   G  D+IR L K G+++N +TT+  T LH AA
Sbjct  133  QLLLNSNMVVALLEGNGRDNTPLHLAARNGHKDIIRLLLKAGIDIN-RTTKSGTALHEAA  191

Query  119  AWGRLETLKALVELDVDIEALNFREERARDVAARYSQTEC  158
             +G+ E +K L++  +D+   N   + A D+  +++ +
Sbjct  192  LYGKTEVVKLLLDAGIDVNIRNTYNQTALDIVNQFTTSHA  231
```

>**ref|XP_002109829.1|** hypothetical protein TRIADDRAFT_63625 [Trichoplax adhaerens]
**gb|EDV27995.1|** hypothetical protein TRIADDRAFT_63625 [Trichoplax adhaerens]
Length=666

**GENE ID: 6751044 TRIADDRAFT_63625** | hypothetical protein [Trichoplax adhaerens] (10 or fewer PubMed links)

```
 Score = 53.5 bits (127),  Expect = 2e-05, Method: Compositional matrix adjust.
 Identities = 29/76 (38%), Positives = 46/76 (60%), Gaps = 0/76 (0%)

Query  165  ADARLTLKKYIAKVSLAVTDTEKGSGKLLKEDKNTILSACRAKNEWLETHTEASINELFE  224
             +AR  L+ +   +   ++D EK  GKL  EDK TI +A   K +WLE++  A   EL
Sbjct  560  VEARNELESFAYSLKNQISDKEKLGGKLSDEDKKTIAAAVEDKIQWLESNPNAETEELKA  619

Query  225  QRQQLEDIVTPIFTKM  240
            ++++L+D+V PI TK+
Sbjct  620  KKKELDDVVQPITTKL  635
```

>**ref|NP_787123.1|** ankyrin, isoform C [Drosophila melanogaster]
**ref|NP_787124.1|** ankyrin, isoform D [Drosophila melanogaster]
**ref|NP_787122.1|** ankyrin, isoform B [Drosophila melanogaster]
**6 more sequence titles**

**ref|NP_787121.1|** ankyrin, isoform A [Drosophila melanogaster]
**gb|AAF59369.2|** ankyrin, isoform B [Drosophila melanogaster]
**gb|AAG22123.1|** ankyrin, isoform C [Drosophila melanogaster]
**gb|AAN06550.1|** ankyrin, isoform A [Drosophila melanogaster]
**gb|AAN06551.1|** ankyrin, isoform D [Drosophila melanogaster]
**gb|ABX00745.1|** LD10053p [Drosophila melanogaster]
Length=1549

**GENE ID: 43770 Ank** | Ankyrin [Drosophila melanogaster] (Over 10 PubMed links)

```
 Score = 53.5 bits (127),  Expect = 2e-05, Method: Compositional matrix adjust.
 Identities = 30/77 (38%), Positives = 44/77 (57%), Gaps = 6/77 (7%)

Query  67   MQLLLEEDIV------GRNLLYAACMAGQSDVIRALAKYGVNLNEKTTRGYTLLHCAAAW  120
            +QLLLE  ++      G   L+ A   G   V + L ++G N++E+T  GYT LH AA +
Sbjct  678  VQLLLEYGVISAAAKNGLTPLHVAAQEGHVLVSQILLEHGANISERTRNGYTPLHMAAHY  737

Query  121  GRLETLKALVELDVDIE  137
            G L+ +K  +E D DIE
Sbjct  738  GHLDLVKFFIENDADIE  754
```

```
 Score = 40.8 bits (94),  Expect = 0.14, Method: Compositional matrix adjust.
 Identities = 38/129 (29%), Positives = 59/129 (45%), Gaps = 19/129 (14%)

Query  64   EQAMQLL---LEEDIV---GRNLLYAACMAGQSDVIRALAKYGVNLNEKTTRGYTLLHCA  117
            E AMQLL    + +I+   G + L+ A   G  D+++ L +YGV ++     G T LH A
Sbjct  643  EIAMQLLQHGADVNIISKSGFSPLHLAAQGGNVDMVQLLLEYGV-ISAAAKNGLTPLHVA  701

Query  118  AAWGRLETLKALVELDVDIEALNFREERARD------VAARYSQTECVEFLDWADARLTL  171
            A  G +   + L+E   +I       ER R+      +AA Y   + V+F    DA + +
Sbjct  702  AQEGHVLVSQILLEHGANI------SERTRNGYTPLHMAAHYGHLDLVKFFIENDADIEM  755

Query  172  KKYIAKVSL  180
               I    L
Sbjct  756  SSNIGYTPL  764
```

```
 Score = 40.4 bits (93),  Expect = 0.18, Method: Compositional matrix adjust.
 Identities = 23/86 (26%), Positives = 40/86 (46%), Gaps = 0/86 (0%)

Query  77   GRNLLYAACMAGQSDVIRALAKYGVNLNEKTTRGYTLLHCAAAWGRLETLKALVELDVDI  136
            G   L+ AC   +  ++  L K+G N+   T  G T LH A+  G +  +  L++ +
Sbjct  399  GFTPLHIACKKNRIKMVELLIKHGANIGATTESGLTPLHVASFMGCINIVIYLLQHEASA  458

Query  137  EALNFREERARDVAARYSQTECVEFL  162
            +    R E    +AAR +Q + +  L
```

```
Sbjct  459   DLPTIRGETPLHLAARANQADIIRIL  484


 Score = 39.3 bits (90),  Expect = 0.45, Method: Compositional matrix adjust.
 Identities = 22/82 (26%), Positives = 39/82 (47%), Gaps = 0/82 (0%)

Query  81    LYAACMAGQSDVIRALAKYGVNLNEKTTRGYTLLHCAAAWGRLETLKALVELDVDIEALN  140
             L+ A   GQ ++++ L + G   N  T +G+T LH A  +G+   ++ L++    I+
Sbjct  534   LHIAAKEGQENIVQVLLENGAENNAVTKKGFTPLHLACKYGKQNVVQILLQNGASIDFQG  593

Query  141   FREERARDVAARYSQTECVEFL  162
               +     VA  Y+    VE L
Sbjct  594   KNDVTPLHVATHYNNPSIVELL  615


 Score = 38.1 bits (87),  Expect = 0.91, Method: Compositional matrix adjust.
 Identities = 18/42 (42%), Positives = 25/42 (59%), Gaps = 0/42 (0%)

Query  77    GRNLLYAACMAGQSDVIRALAKYGVNLNEKTTRGYTLLHCAA  118
             G   L+ A +AGQ DVI   L  Y  N+N ++  G+T L+ AA
Sbjct  106   GNTALHIASLAGQHDVINQLILYNANVNVQSLNGFTPLYMAA  147


 Score = 35.8 bits (81),  Expect = 5.0, Method: Compositional matrix adjust.
 Identities = 36/148 (24%), Positives = 65/148 (43%), Gaps = 5/148 (3%)

Query  75    IVGRNLLYAACMAGQSDVIRALAKYGVNLNEKTTRGYTLLHCAAAWGRLETLKALVELDV  134
             I G   L+ A  A Q+D+IR L +    ++      G T LH A+  G +  +  L++
Sbjct  463   IRGETPLHLAARANQADIIRILLR-SAKVDAIAREGQTPLHVASRLGNINIIMLLLQHGA  521

Query  135   DIEALNFREERARDVAARYSQTECVEFL--DWADARLTLKKYIAKVSLAVTDTEKGSGKL  192
             +I A +  +  A  +AA+  Q   V+ L  + A+     KK    + LA    ++   ++
Sbjct  522   EINAQSNDKYSALHIAAKEGQENIVQVLLENGAENNAVTKKGFTPLHLACKYGKQNVVQI  581

Query  193   LKEDKNTILSACRAKNEWLETHTEASIN  220
             L ++  +I    + KN+    H    N
Sbjct  582   LLQNGASI--DFQGKNDVTPLHVATHYN  607


 Score = 35.4 bits (80),  Expect = 6.3, Method: Compositional matrix adjust.
 Identities = 29/103 (28%), Positives = 44/103 (42%), Gaps = 6/103 (5%)

Query  42    PALTGDVEGLQKIFEDPENPHHEQAMQLL-----LEEDIVGR-NLLYAACMAGQSDVIRA  95
             P LT    GL   +   H E A  LL     ++E  V    L+ A   G   V +
Sbjct  325   PILTKTKNGLSALHMAAQGEHDEAAHLLLDNKAPVDEVTVDYLTALHVAAHCGHVKVAKL  384

Query  96    LAKYGVNLNEKTTRGYTLLHCAAAWGRLETLKALVELDVDIEA  138
             L  Y  N N +   G+T LH A    R++ ++ L++   +I A
Sbjct  385   LLDYKANPNARALNGFTPLHIACKKNRIKMVELLIKHGANIGA  427


 Score = 35.0 bits (79),  Expect = 8.4, Method: Compositional matrix adjust.
 Identities = 25/110 (22%), Positives = 51/110 (46%), Gaps = 3/110 (2%)

Query  63    HEQAMQLLLEEDIVGRNLLYAACMAGQSDVIRA---LAKYGVNLNEKTTRGYTLLHCAAA  119
             H++ + +LLE D+ G+   L A  +A + + + A   L ++  N +  +  G+T LH AA
Sbjct  184   HDKIVAVLLENDVRGKVRLPALHIAAKKNDVNAAKLLLQHDPNADIVSKSGFTPLHIAAH  243

Query  120   WGRLETLKALVELDVDIEALNFREERARDVAARYSQTECVEFLDWADARL  169
             +G ++    L+    D+   +          VA ++ +       L    A++
Sbjct  244   YGNVDIATLLLNNKADVNYVAKHNITPLHVACKWGKLSLCTLLLCRGAKI  293


>ref|XP_001607835.1|  PREDICTED: similar to ENSANGP00000016511 [Nasonia vitripennis]
Length=963

 GENE ID: 100124022 LOC100124022 | similar to ENSANGP00000016511
[Nasonia vitripennis]

 Score = 53.5 bits (127),  Expect = 2e-05, Method: Compositional matrix adjust.
 Identities = 33/93 (35%), Positives = 45/93 (48%), Gaps = 0/93 (0%)

Query  70    LLEEDIVGRNLLYAACMAGQSDVIRALAKYGVNLNEKTTRGYTLLHCAAAWGRLETLKAL  129
             LL  D  G+  L  AC+ GQ  V+  L + G + N+     G   LHCAAA G   TL  L
Sbjct  529   LLWRDDRGQGCLQLACVHGQVTVVDYLLERGADPNDVDAEGVNCLHCAAARGHQNTLLLL  588

Query  130   VELDVDIEALNFREERARDVAARYSQTECVEFL  162
             +  +  I+A + R   A   AA +    CV+ L
Sbjct  589   LHANARIDATDARGNSALHFAADHGHDACVKAL  621


>ref|XP_002054184.1|  GJ24299 [Drosophila virilis]
 gb|EDW67704.1|  GJ24299 [Drosophila virilis]
Length=2125

 GENE ID: 6630153 Dvir\GJ24299 | GJ24299 gene product from transcript GJ24299-RA
[Drosophila virilis] (10 or fewer PubMed links)

 Score = 53.5 bits (127),  Expect = 2e-05, Method: Compositional matrix adjust.
 Identities = 30/99 (30%), Positives = 52/99 (52%), Gaps = 9/99 (9%)

Query  37     NPLLQPALTGDVEGLQKIFEDPENPHHEQAMQLLLEEDIVGRNLLYAACMAGQSDVIRAL  96
               +PLL   +  G+ E   + + E+  +P             D +GR  L+AAC AG + V+R L
Sbjct  1459   SPLLVSSFEGNAEVCELLLENAADPD---------LADFMGRTPLWAACTAGHATVVRLL  1509

Query  97     AKYGVNLNEKTTRGYTLLHCAAAWGRLETLKALVELDVD  135
                +G  ++    + G T+L    AA G +ET++ L++  +D
Sbjct  1510   LFWGCGIDCMDSEGRTVLSIGAAQGNVETVRQLLDRGLD  1548


 Score = 37.7 bits (86),  Expect = 1.2, Method: Compositional matrix adjust.
 Identities = 25/97 (25%), Positives = 47/97 (48%), Gaps = 8/97 (8%)

Query  63     HEQAMQLLLEE--------DIVGRNLLYAACMAGQSDVIRALAKYGVNLNEKTTRGYTLL  114
               H + ++LL+ +           D  GR  L AA  +G  D+++ L + G ++N    +G T L
Sbjct  1333   HSEVVRLLIAQPACKIDLADKEGRTALRAAAWSGHEDILKLLIESGADVNSVDRQGRTSL  1392
```

```
Query  115   HCAAAWGRLETLKALVELDVDIEALNFREERARDVAA  151
               A+  G   + ++ L++    ++  L+      A  VAA
Sbjct  1393  IAASYMGHYDIVEILLDNGANVNHLDLDGRSALCVAA  1429
```

Score = 36.6 bits (83),  Expect = 2.4, Method: Compositional matrix adjust.
Identities = 25/92 (27%), Positives = 43/92 (46%), Gaps = 1/92 (1%)

```
Query  77    GRNLLYAACMAGQSDVIRALAKYGVNLNEKTTRGYTLLHCAAAWGRLETLKALVEL-DVD  135
             G+      AC+ G  D +   L K+  ++N K      T L+   A    +L+ +K L+++ +VD
Sbjct  1623  GKTAFRLACLEGHMDTVEFLLKFCCDVNSKDADSRTTLYILALENKLDIVKYLLDMTNVD  1682

Query  136   IEALNFREERARDVAARYSQTECVEFLDWADA  167
             +    +      A  VAA      + V+ L  A A
Sbjct  1683  VNIPDSEGRTALHVAAWQGHADMVKTLIEAGA  1714
```

Score = 35.4 bits (80),  Expect = 6.6, Method: Compositional matrix adjust.
Identities = 19/70 (27%), Positives = 35/70 (50%), Gaps = 1/70 (1%)

```
Query  73    EDIVGRNLLYAACMAGQSDVIRALAKY-GVNLNEKTTRGYTLLHCAAAWGRLETLKALVE  131
             +D    R  LY    +   + D+++ L      V++N    + G T LH AA   G   + +K L+E
Sbjct  1652  KDADSRTTLYILALENKLDIVKYLLDMTNVDVNIPDSEGRTALHVAAWQGHADMVKTLIE  1711

Query  132   LDVDIEALNF  141
                D+ +++
Sbjct  1712  AGADVNSMDL  1721
```

>**ref|NP_849499.1|** AKR2 (ANKYRIN REPEAT-CONTAINING PROTEIN 2); protein binding [Arabidopsis thaliana]
Length=304

**GENE ID: 829697 AKR2** | AKR2 (ANKYRIN REPEAT-CONTAINING PROTEIN 2); protein binding [Arabidopsis thaliana] (10 or fewer PubMed links)

Score = 53.5 bits (127),  Expect = 2e-05, Method: Compositional matrix adjust.
Identities = 41/123 (33%), Positives = 54/123 (43%), Gaps = 9/123 (7%)

```
Query  41    QPALTGDVEGLQKIFEDPENPHHEQAMQLLLEEDIVGRNLLYAACMAGQSDVIRALAKYG  100
             Q A  GDVEGL+         N            EED  GR  L+ AC  G+     + L   G
Sbjct  186   QTASLGDVEGLKAALASGGNKD---------EEDSEGRTALHFACGYGELKCAQVLIDAG  236

Query  101   VNLNEKTTRGYTLLHCAAAWGRLETLKALVELDVDIEALNFREERARDVAARYSQTECVE  160
              ++N        T LH AA +GR E +   L+E     +    N  E+    DVA   SQ E V+
Sbjct  237   ASVNAVDKNKNTPLHYAAGYGRKECVSLLLENGAAVTLQNLDEKTPIDVAKLNSQLEVVK  296

Query  161   FLD  163
              L+
Sbjct  297   LLE  299
```

Score = 38.9 bits (89),  Expect = 0.51, Method: Compositional matrix adjust.
Identities = 42/182 (23%), Positives = 80/182 (43%), Gaps = 22/182 (12%)

```
Query  37    NPLLQPAL----TGDVEGLQKIFEDPENPHH-EQAMQL----LLEEDIVG----------  77
             +P L+P L      G     + K + DPE       +AM +     L ++ +
Sbjct  120   DPELKPILDEIDAGGPSAMMKYWNDPEVLKKLGEAMGMPVAGLPDQTVSAEPEVAEEGEE  179

Query  78    -RNLLYAACMAGQSDVIRALAKYGVNLNEKTTRGYTLLHCAAAWGRLETLKALVELDVDI  136
               ++++      G   + ++A     G N +E+ + G T LH A   +G L+   + L++     +
Sbjct  180   EESIVHQTASLGDVEGLKAALASGGNKDEEDSEGRTALHFACGYGELKCAQVLIDAGASV  239

Query  137   EALNFREERARDVAARYSQTECVEFLDWADARLTLKKYIAK--VSLAVTDTEKGSGKLLK  194
              A++  +        AA Y + ECV  L     A +TL+     K  + +A  +++    KLL+
Sbjct  240   NAVDKNKNTPLHYAAGYGRKECVSLLLENGAAVTLQNLDEKTPIDVAKLNSQLEVVKLLE  299

Query  195   ED  196
             +D
Sbjct  300   KD  301
```

>**gb|AAC37208.1|** ankyrin [Drosophila melanogaster]
**prf||2022340A** ankyrin
Length=1549

Score = 53.5 bits (127),  Expect = 2e-05, Method: Compositional matrix adjust.
Identities = 30/77 (38%), Positives = 44/77 (57%), Gaps = 6/77 (7%)

```
Query  67    MQLLLEEDIV------GRNLLYAACMAGQSDVIRALAKYGVNLNEKTTRGYTLLHCAAAW  120
             +QLLLE  ++        G    L+ A    G    V + L ++G N++E+T   GYT LH AA +
Sbjct  678   VQLLLEYGVISAAAKNGLTPLHVAAQEGHVLVSQILLEHGANISERTRNGYTPLHMAAHY  737

Query  121   GRLETLKALVELDVDIE  137
             G L+ +K   +E D DIE
Sbjct  738   GHLDLVKFFIENDADIE  754
```

Score = 40.8 bits (94),  Expect = 0.15, Method: Compositional matrix adjust.
Identities = 38/129 (29%), Positives = 59/129 (45%), Gaps = 19/129 (14%)

```
Query  64    EQAMQLL---LEEDIV---GRNLLYAACMAGQSDVIRALAKYGVNLNEKTTRGYTLLHCA  117
             E AMQLL     + +I+    G + L+ A    G   D+++ L +YGV ++      G T LH A
Sbjct  643   EIAMQLLQHGADVNIISKSGFSPLHLAAQGGNVDMVQLLLEYGV-ISAAAKNGLTPLHVA  701

Query  118   AAWGRLETLKALVELDVDIEALNFREERARD------VAARYSQTECVEFLDWADARLTL  171
             A  G +    + L+E    +I        ER R+       +AA Y    + V+F    DA + +
Sbjct  702   AQEGHVLVSQILLEHGANI------SERTRNGYTPLHMAAHYGHLDLVKFFIENDADIEM  755

Query  172   KKYIAKVSL  180
                 I    L
Sbjct  756   SSNIGYTPL  764
```

Score = 40.4 bits (93),  Expect = 0.18, Method: Compositional matrix adjust.
Identities = 23/86 (26%), Positives = 40/86 (46%), Gaps = 0/86 (0%)

```
Query  77   GRNLLYAACMAGQSDVIRALAKYGVNLNEKTTRGYTLLHCAAAWGRLETLKALVELDVDI  136
            G   L+ AC   +  ++  L K+G N+    T  G T LH A+  G +  +  L++ +
Sbjct  399  GFTPLHIACKKNRIKMVELLIKHGANIGATTESGLTPLHVASFMGCINIVIYLLQHEASA  458

Query  137  EALNFREERARDVAARYSQTECVEFL  162
            +    R E    +AAR +Q + +  L
Sbjct  459  DLPTIRGETPLHLAARANQADIIRIL  484
```

Score = 39.3 bits (90), Expect = 0.46, Method: Compositional matrix adjust.
Identities = 22/82 (26%), Positives = 39/82 (47%), Gaps = 0/82 (0%)

```
Query  81   LYAACMAGQSDVIRALAKYGVNLNEKTTRGYTLLHCAAAWGRLETLKALVELDVDIEALN  140
            L+ A   GQ ++++ L + G   N   T +G+T LH A  +G+    ++ L++    I+
Sbjct  534  LHIAAKEGQENIVQVLLENGAENNAVTKKGFTPLHLACKYGKQNVVQILLQNGASIDFQG  593

Query  141  FREERARDVAARYSQTECVEFL  162
              +     VA  Y+    VE L
Sbjct  594  KNDVTPLHVATHYNNPSIVELL  615
```

Score = 38.1 bits (87), Expect = 0.92, Method: Compositional matrix adjust.
Identities = 18/42 (42%), Positives = 25/42 (59%), Gaps = 0/42 (0%)

```
Query  77   GRNLLYAACMAGQSDVIRALAKYGVNLNEKTTRGYTLLHCAA  118
            G   L+ A +AGQ DVI  L  Y  N+N ++  G+T L+ AA
Sbjct  106  GNTALHIASLAGQHDVINQLILYNANVNVQSLNGFTPLYMAA  147
```

Score = 35.8 bits (81), Expect = 4.3, Method: Compositional matrix adjust.
Identities = 36/149 (24%), Positives = 65/149 (43%), Gaps = 5/149 (3%)

```
Query  75   IVGRNLLYAACMAGQSDVIRALAKYGVNLNEKTTRGYTLLHCAAAWGRLETLKALVELDV  134
            I G   L+ A  A Q+D+IR L +    ++     G T LH A+  G +  +  L++
Sbjct  463  IRGETPLHLAARANQADIIRILLR-SAKVDAIVREGQTPLHVASRLGNINIIMLLLQHGA  521

Query  135  DIEALNFREERARDVAARYSQTECVEFL--DWADARLTLKKYIAKVSLAVTDTEKGSGKL  192
            +I A +  +  A  +AA+  Q   V+ L  + A+     KK    + LA    ++   ++
Sbjct  522  EINAQSNDKYSALHIAAKEGQENIVQVLLENGAENNAVTKKGFTPLHLACKYGKQNVVQI  581

Query  193  LKEDKNTILSACRAKNEWLETHTEASINE  221
            L ++  +I    + KN+    H     N
Sbjct  582  LLQNGASI--DFQGKNDVTPLHVATHYNN  608
```

Score = 35.4 bits (80), Expect = 6.6, Method: Compositional matrix adjust.
Identities = 29/103 (28%), Positives = 44/103 (42%), Gaps = 6/103 (5%)

```
Query  42   PALTGDVEGLQKIFEDPENPHHEQAMQLL-----LEEDIVGR-NLLYAACMAGQSDVIRA  95
            P LT    GL  +     +  H E A  LL     ++E  V     L+ A   G   V +
Sbjct  325  PILTKTKNGLSALHMAAQGEHDEAAHLLLDNKAPVDEVTVDYLTALHVAAHCGHVKVAKL  384

Query  96   LAKYGVNLNEKTTRGYTLLHCAAAWGRLETLKALVELDVDIEA  138
            L  Y  N N +   G+T LH A    R++ ++ L++    +I A
Sbjct  385  LLDYKANPNARALNGFTPLHIACKKNRIKMVELLIKHGANIGA  427
```

Score = 35.0 bits (79), Expect = 8.4, Method: Compositional matrix adjust.
Identities = 25/110 (22%), Positives = 51/110 (46%), Gaps = 3/110 (2%)

```
Query  63   HEQAMQLLLEEDIVGRNLLYAACMAGQSDVIRA---LAKYGVNLNEKTTRGYTLLHCAAA  119
            H++ + +LLE D+ G+   L A  +A + + + A   L ++   N +  +  G+T LH AA
Sbjct  184  HDKIVAVLLENDVRGKVRLPALHIAAKKNDVNAAKLLLQHDPNADIVSKSGFTPLHIAAH  243

Query  120  WGRLETLKALVELDVDIEALNFREERARDVAARYSQTECVEFLDWADARL  169
            +G ++     L+     D+   +           VA ++ +       L     A++
Sbjct  244  YGNVDIATLLLNNKADVNYVAKHNITPLHVACKWGKLSLCTLLLCRGAKI  293
```

>**ref|XP_391790.1|** hypothetical protein FG11614.1 [Gibberella zeae PH-1]
Length=997

**GENE ID: 2786423 FG11614.1** | hypothetical protein [Gibberella zeae PH-1]

Score = 53.5 bits (127), Expect = 2e-05, Method: Compositional matrix adjust.
Identities = 28/95 (29%), Positives = 50/95 (52%), Gaps = 0/95 (0%)

```
Query  77   GRNLLYAACMAGQSDVIRALAKYGVNLNEKTTRGYTLLHCAAAWGRLETLKALVELDVDI  136
            G   L+ A ++G  D++R L ++    L+ K  +   T LHCA    G ++ + AL+E +V I
Sbjct  837  GLTALHTASLSGDVDIVRVLLRHKPKLDLKAKKSGTALHCAVIKGSMDVVMALIEAEVPI  896

Query  137  EALNFREERARDVAARYSQTECVEFLDWADARLTL  171
              L+    + A   +A R ++T   +  L     A L++
Sbjct  897  NELDVYGDTASHIAVRMARTSILRILIRHGADLSI  931
```

Score = 37.0 bits (84), Expect = 2.2, Method: Compositional matrix adjust.
Identities = 22/83 (26%), Positives = 36/83 (43%), Gaps = 0/83 (0%)

```
Query  81   LYAACMAGQSDVIRALAKYGVNLNEKTTRGYTLLHCAAAWGRLETLKALVELDVDIEALN  140
            L+ A + G  DV+ AL +   V +NE    G T  H A     R   L+ L+     D+   N
Sbjct  874  LHCAVIKGSMDVVMALIEAEVPINELDVYGDTASHIAVRMARTSILRILIRHGADLSICN  933

Query  141  FREERARDVAARYSQTECVEFLD  163
               +  +D+A       + +  L+
Sbjct  934  STHQIPKDLAKSIGTFDIIPILE  956
```

>**ref|XP_001319508.1|** ankyrin repeat protein [Trichomonas vaginalis G3]
**gb|EAY07285.1|** ankyrin repeat protein, putative [Trichomonas vaginalis G3]
Length=477

**GENE ID: 4765174 TVAG_223480** | ankyrin repeat protein
[Trichomonas vaginalis G3] (10 or fewer PubMed links)

Score = 53.5 bits (127), Expect = 2e-05, Method: Compositional matrix adjust.
Identities = 32/90 (35%), Positives = 47/90 (52%), Gaps = 0/90 (0%)

```
Query  73   EDIVGRNLLYAACMAGQSDVIRALAKYGVNLNEKTTRGYTLLHCAAAWGRLETLKALVEL  132
            +D  G   L  A + G+ DV+   L   G N+  K+  G T L CA+A G LE +K L++
Sbjct  289  QDKYGNTPLLLASINGKLDVVNYLISIGFNIEVKSKIGNTPLICASANGNLEVVKYLIKA  348

Query  133  DVDIEALNFREERARDVAARYSQTECVEFL  162
              +IEA N     A  +A+    + E V+FL
Sbjct  349  GANIEAKNNNGNTALTLASHSGKLEIVKFL  378


 Score = 41.2 bits (95),  Expect = 0.12, Method: Compositional matrix adjust.
 Identities = 27/94 (28%), Positives = 42/94 (44%), Gaps = 0/94 (0%)

Query  76   VGRNLLYAACMAGQSDVIRALAKYGVNLNEKTTRGYTLLHCAAAWGRLETLKALVELDVD  135
            +G   L  A   G  +V++ L K G N+  K   G T L  A+  G+LE +K L+E
Sbjct  325  IGNTPLICASANGNLEVVKYLIKAGANIEAKNNNGNTALTLASHSGKLEIVKFLIESGAC  384

Query  136  IEALNFREERARDVAARYSQTECVEFLDWADARL  169
                N   +    +A++    E V++    DA L
Sbjct  385  KNTKNKNGDTPLIIASKSRHLEVVKYFISLDANL  418


>gb|AAA28076.1|  heat shock protein 4 [Caenorhabditis elegans]
Length=288

 Score = 53.5 bits (127),  Expect = 2e-05, Method: Compositional matrix adjust.
 Identities = 29/76 (38%), Positives = 45/76 (59%), Gaps = 0/76 (0%)

Query  165  ADARLTLKKYIAKVSLAVTDTEKGSGKLLKEDKNTILSACRAKNEWLETHTEASINELFE  224
             ++R  L+ Y  ++    + D EK  GKL  EDK +I SA      EWL ++ +AS   E  E
Sbjct  192  VESRNELEAYAYQIKTQIADKEKLGGKLTDEDKVSIESAVERAIEWLGSNQDASTEENKE  251

Query  225  QRQQLEDIVTPIFTKM  240
            Q+++LE +V PI +K+
Sbjct  252  QKKELESVVQPIVSKL  267


>ref|XP_001994442.1| GH16234 [Drosophila grimshawi]
 gb|EDV91071.1| GH16234 [Drosophila grimshawi]
Length=2124

 GENE ID: 6568485 Dgri\GH16234 | GH16234 gene product from transcript GH16234-RA
[Drosophila grimshawi] (10 or fewer PubMed links)

 Score = 53.5 bits (127),  Expect = 2e-05, Method: Compositional matrix adjust.
 Identities = 30/99 (30%), Positives = 52/99 (52%), Gaps = 9/99 (9%)

Query  37    NPLLQPALTGDVEGLQKIFEDPENPHHEQAMQLLLEEDIVGRNLLYAACMAGQSDVIRAL  96
             +PLL   +  G+ E   + + E+  +P            D +GR  L+AAC AG + V+R L
Sbjct  1456  SPLLVSSFEGNAEVCELLLENAADPD---------LADFMGRTPLWAACTAGHATVVRLL  1506

Query  97    AKYGVNLNEKTTRGYTLLHCAAAWGRLETLKALVELDVD  135
               +G  ++    + G T+L    AA G +ET++ L++  +D
Sbjct  1507  LFWGCGIDCMDSEGRTVLSIGAAQGNVETVRQLLDRGLD  1545


 Score = 37.7 bits (86),  Expect = 1.1, Method: Compositional matrix adjust.
 Identities = 25/97 (25%), Positives = 47/97 (48%), Gaps = 8/97 (8%)

Query  63    HEQAMQLLLEE--------DIVGRNLLYAACMAGQSDVIRALAKYGVNLNEKTTRGYTLL  114
             H + ++LL+ +         D  GR  L AA  +G  D+++ L + G ++N    +G T L
Sbjct  1330  HSEVVRLLIAQPACKIDLADKEGRTALRAAAWSGHEDILKLLIESGADVNSVDRQGRTSL  1389

Query  115   HCAAAWGRLETLKALVELDVDIEALNFREERARDVAA  151
               A+  G  + ++ L++   ++  L+     A  VAA
Sbjct  1390  IAASYMGHYDIVEILLDNGANVNHLDLDGRSALCVAA  1426


 Score = 35.4 bits (80),  Expect = 5.2, Method: Compositional matrix adjust.
 Identities = 19/70 (27%), Positives = 35/70 (50%), Gaps = 1/70 (1%)

Query  73    EDIVGRNLLYAACMAGQSDVIRALAKY-GVNLNEKTTRGYTLLHCAAAWGRLETLKALVE  131
             +D   R  LY   +  + D+++ L      V++N    + G T LH AA  G  + +K L+E
Sbjct  1649  KDADSRTTLYILALENKMDIVKYLLDMTNVDVNIPDSEGRTALHVAAWQGHTDMVKTLIE  1708

Query  132   LDVDIEALNF  141
                D+ +++
Sbjct  1709  AGADVNSMDL  1718


>ref|XP_001553065.1| hypothetical protein BC1G_08957 [Botryotinia fuckeliana B05.10]
 gb|EDN28891.1| hypothetical protein BC1G_08957 [Botryotinia fuckeliana B05.10]
Length=333

 GENE ID: 5433596 BC1G_08957 | hypothetical protein
[Botryotinia fuckeliana B05.10]

 Score = 53.5 bits (127),  Expect = 2e-05, Method: Compositional matrix adjust.
 Identities = 45/151 (29%), Positives = 68/151 (45%), Gaps = 11/151 (7%)

Query  62   HHEQAMQLLLEEDI-------VGRNLLYAACMAGQSDVIRALAKYGVNLNEKTTRGYTLL  114
            HH +    LLE               G  +L+ A   G  ++++ L +Y   L  +   G T L
Sbjct  114  HHVKTTDLLLEAGADTSMTLKGGETVLHIAARIGDLEMVKLLVEYEAYLEARDGWGNTPL  173

Query  115  HCAAAWGRLETLKALVELDVDIEALNFREERARDVAARYSQTECVEFLDWADARLTLKKY  174
              A+ WG   T+K L+E     DIEA + +   A  +A R+   E V+ L  +DA + +K
Sbjct  174  LSASRWGHPHTIKYLLEKGADIEARDMKGSTALLACRHDCVEAVKVLIHSDANVRVKDK  233

Query  175  IAKVSL----AVTDTEKGSGKLLKEDKNTIL  201
              +  L    A  D   G G  +KED   +L
Sbjct  234  KGRGPLHRAIAGVDFIDGVGAKVKEDMVRLL  264


>ref|XP_002020191.1| GL13852 [Drosophila persimilis]
 gb|EDW39003.1| GL13852 [Drosophila persimilis]
```

Length=1345

**GENE ID: 6595117 Dper\GL13852** | GL13852 gene product from transcript GL13852-RA
[Drosophila persimilis] (10 or fewer PubMed links)

```
 Score = 53.5 bits (127),  Expect = 2e-05, Method: Compositional matrix adjust.
 Identities = 38/117 (32%), Positives = 54/117 (46%), Gaps = 10/117 (8%)

Query  63   HEQAMQLLLEEDIV-------GRNLLYAACMAGQSDVIRALAKYG---VNLNEKTTRGYT  112
            HE  ++LLL  D         G + L+ A  AG++D++R L  +        N +T    T
Sbjct  61   HEDIVRLLLANDASPNLPDSRGSSPLHLAAWAGETDIVRLLLTHPYRPATANLQTIEQET  120

Query  113  LLHCAAAWGRLETLKALVELDVDIEALNFREERARDVAARYSQTECVEFLDWADARL  169
             LHCAA  G    L  L+  D D    N R E   D+AA+Y + + V+ L  A   L
Sbjct  121  PLHCAAQHGHTGALALLLGHDADPNMRNSRGETPLDLAAQYGRLQAVQMLIRAHPEL  177
```

>**dbj|BAF80467.1|** HSP70 protein [Poecilia reticulata]
Length=632

```
 Score = 53.1 bits (126),  Expect = 2e-05, Method: Compositional matrix adjust.
 Identities = 27/75 (36%), Positives = 44/75 (58%), Gaps = 0/75 (0%)

Query  166  DARLTLKKYIAKVSLAVTDTEKGSGKLLKEDKNTILSACRAKNEWLETHTEASINELFEQ  225
            D+R  L+ Y   +   + D EK  GKL  EDK  I  A   K EW+E+H +A + +   +
Sbjct  540  DSRNELESYAYSLKNQIGDKEKLGGKLSDEDKEAIEKAVEEKIEWMESHQDADLEDFQAK  599

Query  226  RQQLEDIVTPIFTKM  240
            +++LE++V PI +K+
Sbjct  600  KKELEEVVQPIISKL  614
```

>**ref|XP_001330150.1|** ankyrin repeat protein [Trichomonas vaginalis G3]
**gb|EAY01265.1|** ankyrin repeat protein, putative [Trichomonas vaginalis G3]
Length=354

**GENE ID: 4759090 TVAG_027330** | ankyrin repeat protein
[Trichomonas vaginalis G3] (10 or fewer PubMed links)

```
 Score = 53.1 bits (126),  Expect = 3e-05, Method: Compositional matrix adjust.
 Identities = 35/111 (31%), Positives = 53/111 (47%), Gaps = 0/111 (0%)

Query  72   EEDIVGRNLLYAACMAGQSDVIRALAKYGVNLNEKTTRGYTLLHCAAAWGRLETLKALVE  131
            E+D  G+  L+ A +    +    L  +G N+NEKT  G T LH AA   R ET + L+
Sbjct  73   EKDEEGKTALHIAAIYNSKETAEFLISHGANINEKTNNGKTALHIAADNNRKETAEFLIS  132

Query  132  LDVDIEALNFREERARDVAARYSQTECVEFLDWADARLTLKKYIAKVSLAV  182
               +I   +   + A  +AA+ ++ E VEFL    A +  K    K  L +
Sbjct  133  HGANINEKDIYGKTALHIAAKNNRKEIVEFLISHGANINEKDEDGKTELHI  183


 Score = 49.7 bits (117),  Expect = 3e-04, Method: Compositional matrix adjust.
 Identities = 32/111 (28%), Positives = 50/111 (45%), Gaps = 0/111 (0%)

Query  72   EEDIVGRNLLYAACMAGQSDVIRALAKYGVNLNEKTTRGYTLLHCAAAWGRLETLKALVE  131
            E+D  G   L+ A      +++  L  +G N+NEK   G T LH AA +   ET + L+
Sbjct  40   EKDEFGETSLHIAAYNDSKEIVEVLISHGANINEKDEEGKTALHIAAIYNSKETAEFLIS  99

Query  132  LDVDIEALNFREERARDVAARYSQTECVEFLDWADARLTLKKYIAKVSLAV  182
               +I       + A  +AA  ++ E  EFL    A +  K    K +L +
Sbjct  100  HGANINEKTNNGKTALHIAADNNRKETAEFLISHGANINEKDIYGKTALHI  150


 Score = 45.4 bits (106),  Expect = 0.006, Method: Compositional matrix adjust.
 Identities = 32/111 (28%), Positives = 49/111 (44%), Gaps = 0/111 (0%)

Query  72   EEDIVGRNLLYAACMAGQSDVIRALAKYGVNLNEKTTRGYTLLHCAAAWGRLETLKALVE  131
            E+DI G+  L+ A    + +++  L  +G N+NEK   G T LH AA      T + L+
Sbjct  139  EKDIYGKTALHIAAKNNRKEIVEFLISHGANINEKDEDGKTELHIAAENNSKATAEVLIS  198

Query  132  LDVDIEALNFREERARDVAARYSQTECVEFLDWADARLTLKKYIAKVSLAV  182
               +I   +   + A  +AA     E  EFL    A +  K    K  L +
Sbjct  199  HGANINEKDEYGQTALHIAAYNDSKEIAEFLISHGANINEKDEDGKTELHI  249


 Score = 39.3 bits (90),  Expect = 0.44, Method: Compositional matrix adjust.
 Identities = 27/87 (31%), Positives = 40/87 (45%), Gaps = 0/87 (0%)

Query  96   LAKYGVNLNEKTTRGYTLLHCAAAWGRLETLKALVELDVDIEALNFREERARDVAARYSQ  155
            L  +G N+NEK   G T LH AA     E ++ L+    +I   +   + A  +AA Y+
Sbjct  31   LISHGANINEKDEFGETSLHIAAYNDSKEIVEVLISHGANINEKDEEGKTALHIAAIYNS  90

Query  156  TECVEFLDWADARLTLKKYIAKVSLAV  182
             E  EFL    A +  K    K +L +
Sbjct  91   KETAEFLISHGANINEKTNNGKTALHI  117


 Score = 38.5 bits (88),  Expect = 0.62, Method: Compositional matrix adjust.
 Identities = 33/118 (27%), Positives = 50/118 (42%), Gaps = 1/118 (0%)

Query  72   EEDIVGRNLLYAACMAGQSDVIRALAKYGVNLNEKTTRGYTLLHCAAAWGRLETLKALVE  131
            E+D  G+  L+ A      ++    L  +G N+NEK   G T LH AA      T + L+
Sbjct  205  EKDEYGQTALHIAAYNDSKEIAEFLISHGANINEKDEDGKTELHIAAENNSKATAEVLIS  264

Query  132  LDVDIEALNFREERARDVAARYSQTECVEFLDWADARLTLKKYIAKVSLAVTDTEKGS  189
               +I   +   + A  +AA     E  EFL    A +  K    + +L +  TE  S
Sbjct  265  HGANINEKDEYGQTALHIAAYNDSKEIAEFLISHGANINEKDEYGQTALHIA-TENNS  321
```

>**gb|EDL99058.1|** similar to hypothetical protein DKFZp434D2328 (predicted) [Rattus
norvegicus]
Length=1102

```
 Score = 53.1 bits (126),  Expect = 3e-05, Method: Compositional matrix adjust.
 Identities = 40/135 (29%), Positives = 58/135 (42%), Gaps = 7/135 (5%)

Query  38   PLLQPALTGDVEGLQKIFEDPENPHHEQAMQLLLEE------DIVGRNLLYAACMAGQSD  91
```

```
            PLL      D  G   +     N H E    LL +       D   R  L+ A   G  D
Sbjct  128  PLLSSVNVSDRGGRTALHHAALNGHMEMVNLLLAKGANINAFDKKDRRALHWAAYMGHLD  187

Query  92   VIRALAKYGVNLNEKTTRGYTLLHCAAAWGRLETLKALVELDVDIEALNFREERARDVAA  151
            V+  L  +G  +  K  +GYT LH AA+ G++  +K L+ L V+I+ +N     A  +A
Sbjct  188  VVALLINHGAEVTCKDKKGYTPLHAAASNGQINVVKHLLNLGVEIDEINVYGNTALHIAC  247

Query  152  RYSQTECV-EFLDWA  165
               Q   V E +D+
Sbjct  248  YNGQDAVVNELIDYG  262


 Score = 43.1 bits (100),  Expect = 0.025, Method: Compositional matrix adjust.
 Identities = 33/104 (31%), Positives = 51/104 (49%), Gaps = 16/104 (15%)

Query  72   EEDIVGRNLLYAACMAGQSDVIRALAKYGVNLNEKTTRGYTLLHCAAA--WGRLETLKAL  129
            E ++ G   L+ AC  GQ  V+  L  YG N+N+    G+T LH AAA   G L  L+ L
Sbjct  234  EINVYGNTALHIACYNGQDAVVNELIDYGANVNQPNNSGFTPLHFAAASTHGAL-CLELL  292

Query  130  VE--LDVDIE-----------ALNFREERARDVAARYSQTECVE  160
            V    DV+I+           A++ R  R++ +     + +CV+
Sbjct  293  VNNGADVNIQSKDGKSPLHMTAVHGRFTRSQTLIQNGGEIDCVD  336


 Score = 40.0 bits (92),  Expect = 0.24, Method: Compositional matrix adjust.
 Identities = 36/128 (28%), Positives = 53/128 (41%), Gaps = 30/128 (23%)

Query  74   DIVGRNLLYAACMAGQSDVIRALAKYGVNLNEKTTRGYTLLHCAAAWGRLETLKALVELD  133
            D  GR  L+AA    G  + I+ L   G + ++K   G T LH AAA      +KALV
Sbjct  420  DTFGRTCLHAAAAGGNVECIKLLQSSGADFHKKDKCGRTPLHYAAANCHFHCIKALVTTG  479

Query  134  VDIE--------ALNFRE---------------------ERARDVAARYSQTECVEFLDW  164
             +I         AL++                       ERAR+V  + +   C+EFL
Sbjct  480  ANINETDNWGRTALHYAAASDMDRNKMILGNAHDNSEELERAREVKGKDAAL-CLEFLLQ  538

Query  165  ADARLTLK  172
             DA  +++
Sbjct  539  NDANPSIR  546


 Score = 35.4 bits (80),  Expect = 5.7, Method: Compositional matrix adjust.
 Identities = 47/204 (23%), Positives = 81/204 (39%), Gaps = 37/204 (18%)

Query  36   KNPLLQPALTGDVEGLQKIFEDPENPH--------------------HEQAMQLLLEE--  73
            + PL    + G    L+ + E  +NP                     H  A+ LLLE+
Sbjct  653  RTPLHASVINGHTLCLRLLLEIADNPEVVDVKDAKGQTPLMLAVAYGHIDAVSLLLEKEA  712

Query  74   -----DIVGRNLLYAACMAGQSDVIRALAKYGVNLNEKTTRGYTLLHCAAAWGRLETLKA  128
                 D VG   L+   M G  + ++ L +   ++  K +RG T LH AAA G    L
Sbjct  713  NVDAVDTVGCTALHRGIMTGHEECVQMLLEQEASILCKDSRGRTPLHYAAARGHATWLNE  772

Query  129  LVELDVDIEALNFREERARDVA--ARYSQTE-CVEFLDWADARLTLKKYIAK----VSLA  181
            LV++ +  E    ++ +       A Y+  E C+E L     +   +K+I      +  A
Sbjct  773  LVQIALSEEDCCLKDNQGYTPLHWACYNGNENCIEVL---LEQKCFRKFIGNPFTPLHCA  829

Query  182  VTDTEKGSGKLLKEDKNTILSACR  205
            + +  +    LL    ++ + +CR
Sbjct  830  IINGHESCASLLLGAIDSSIVSCR  853
```

>**dbj|BAA34491.1|** G KIAA0771 protein [Homo sapiens]
Length=948

**GENE ID: 23368 PPP1R13B** | protein phosphatase 1, regulatory (inhibitor) subunit 13B [Homo sapiens] (Over 10 PubMed links)

```
 Score = 53.1 bits (126),  Expect = 3e-05, Method: Compositional matrix adjust.
 Identities = 50/177 (28%), Positives = 78/177 (44%), Gaps = 20/177 (11%)

Query  39   LLQPALTGDVEGLQKIF---EDPENPHHEQAMQLLLEEDIVGRNLLYAACMAGQSDVIRA  95
            LL  +L G+ + +Q+I    EDP  P+ E            G    L+ A  AG   +++
Sbjct  750  LLDASLEGEFDLVQRIIYEVEDPSKPNDE------------GITPLHNAVCAGHHHIVKF  797

Query  96   LAKYGVNLNEKTTRGYTLLHCAAAWGRLETLKALVELDVDIEALNFRE-ERARDVAARYS  154
            L  +GVN+N   + G+T LHCAA+   +   K LVE    I A    + E A D
Sbjct  798  LLDFGVNVNAADSDGWTPLHCAASCNSVHLCKQLVESGAAIFASTISDIETAADKCEEME  857

Query  155  Q--TECVEFLDWADARLTLKKYIAKVSLAVTDTEKGSGKLLKEDKNTILSACRAKNE  209
            +   +C +FL     +L +      V+ A+ D E  +   L   +   L+  R K+E
Sbjct  858  EGYIQCSQFLYGVQEKLGVMN--KGVAYALWDYEAQNSDELSFHEGDALTILRRKDE  912
```

>**ref|XP_001235479.1|** UG PREDICTED: hypothetical protein [Gallus gallus]
Length=721

**GENE ID: 772327 ANKRD42** | ankyrin repeat domain 42 [Gallus gallus]

```
 Score = 53.1 bits (126),  Expect = 3e-05, Method: Compositional matrix adjust.
 Identities = 30/80 (37%), Positives = 45/80 (56%), Gaps = 1/80 (1%)

Query  84   ACMAGQSDVIRALAKYGV-NLNEKTTRGYTLLHCAAAWGRLETLKALVELDVDIEALNFR  142
            A   G   V+R L + GV N+NE+  +G TL+H AA  G +  L+ L+E+  D +  +
Sbjct  584  AAFRGDLLVLRRLVRCGVININERDDKGSTLMHKAAGQGHIHCLQWLIEMGADCDITDDA  643

Query  143  EERARDVAARYSQTECVEFL  162
             E  +DVA R++Q   VE L
Sbjct  644  GETPKDVAKRFAQLAAVELL  663


 Score = 37.7 bits (86),  Expect = 1.3, Method: Compositional matrix adjust.
 Identities = 24/88 (27%), Positives = 42/88 (47%), Gaps = 0/88 (0%)

Query  75   IVGRNLLYAACMAGQSDVIRALAKYGVNLNEKTTRGYTLLHCAAAWGRLETLKALVELDV  134
            + G    + A + GQ   ++AL   G NL  +  RG T  H AAA G+  TL+ ++
Sbjct  404  VRGWTAAHLAAIRGQDACMQALLINGANLEARDDRGCTPSHLAAAHGQSYTLQTILRSGA  463

Query  135  DIEALNFREERARDVAARYSQTECVEFL  162
```

```
            +  A +  + +    AA + +  C++ L
Sbjct  464  NANAADRNDWKPVHYAAFHGRLGCLQLL  491
```

>**ref|XP_692689.2|** PREDICTED: similar to cask-interacting protein 2, partial [Danio rerio]
Length=400

**GENE ID: 564253 CH211-119C20.2** | similar to cask-interacting protein 2 [Danio rerio]

```
 Score = 53.1 bits (126),  Expect = 3e-05, Method: Compositional matrix adjust.
 Identities = 32/103 (31%), Positives = 56/103 (54%), Gaps = 10/103 (9%)

Query  65   QAMQLLLEEDIV-------GRN--LLYAACMAGQSDVIRALAKYGVNLNEKTTRGYTLLH  115
            +  QLLL  ++V       GR+   L+ A   G  D+IR L K G+++N +TT+  T LH
Sbjct  162  KVTQLLLNSNMVVALLEGNGRDNTPLHLAARNGHKDIIRLLLKAGIDIN-RTTKSGTALH  220

Query  116  CAAAWGRLETLKALVELDVDIEALNFREERARDVAARYSQTEC  158
             AA +G+ E +K L++  +D+   N   + A D+  +++ +
Sbjct  221  EAALYGKTEVVKLLLDAGIDVNIRNTYNQTALDIVNQFTTSHA  263
```

>**ref|NP_065208.2|** ankyrin 1 isoform 4 [Homo sapiens]
**gb|EAW63247.1|** ankyrin 1, erythrocytic, isoform CRA_g [Homo sapiens]
Length=1856

**GENE ID: 286 ANK1** | ankyrin 1, erythrocytic [Homo sapiens]
(Over 10 PubMed links)

```
 Score = 53.1 bits (126),  Expect = 3e-05, Method: Compositional matrix adjust.
 Identities = 30/88 (34%), Positives = 49/88 (55%), Gaps = 6/88 (6%)

Query  50   GLQKIFEDPENPHHEQAMQLLLEEDIV------GRNLLYAACMAGQSDVIRALAKYGVNL  103
            GL  +    +  H +  ++LL +E I+      G   L+ A +AGQ +V+R L  YG N+
Sbjct  45   GLNGLHLASKEGHVKMVVELLHKEIILETTTKKGNTALHIAALAGQDEVVRELVNYGANV  104

Query  104  NEKTTRGYTLLHCAAAWGRLETLKALVE  131
            N ++ +G+T L+ AA    LE +K L+E
Sbjct  105  NAQSQKGFTPLYMAAQENHLEVVKFLLE  132


 Score = 46.6 bits (109),  Expect = 0.003, Method: Compositional matrix adjust.
 Identities = 28/87 (32%), Positives = 44/87 (50%), Gaps = 0/87 (0%)

Query  81   LYAACMAGQSDVIRALAKYGVNLNEKTTRGYTLLHCAAAWGRLETLKALVELDVDIEALN  140
            L+ A   G +++++ L +   N N  TT G+T LH AA  G +ET+ AL+E +     +
Sbjct  474  LHCAARIGHTNMVKLLLENNANPNLATTAGHTPLHIAAREGHVETVLALLEKEASQACMT  533

Query  141  FREERARDVAARYSQTECVEFLDWADA  167
             +      VAA+Y +     E L   DA
Sbjct  534  KKGFTPLHVAAKYGKVRVAELLLERDA  560


 Score = 45.1 bits (105),  Expect = 0.007, Method: Compositional matrix adjust.
 Identities = 31/117 (26%), Positives = 52/117 (44%), Gaps = 7/117 (5%)

Query  63   HEQAMQLLLEED-------IVGRNLLYAACMAGQSDVIRALAKYGVNLNEKTTRGYTLLH  115
            H +  ++LL++        + G   L+ AC      V+  L K G +++  T  G T LH
Sbjct  350  HHRVAKVLLDKGAKPNSRALNGFTPLHIACKKNHVRVMELLLKTGASIDAVTESGLTPLH  409

Query  116  CAAAWGRLETLKALVELDVDIEALNFREERARDVAARYSQTECVEFLDWADARLTLK  172
             A+  G L  +K L++        N + E    +AAR   TE  ++L    A++  K
Sbjct  410  VASFMGHLPIVKNLLQRGASPNVSNVKVETPLHMAARAGHTEVAKYLLQNKAKVNAK  466


 Score = 42.0 bits (97),  Expect = 0.070, Method: Compositional matrix adjust.
 Identities = 25/86 (29%), Positives = 39/86 (45%), Gaps = 0/86 (0%)

Query  77   GRNLLYAACMAGQSDVIRALAKYGVNLNEKTTRGYTLLHCAAAWGRLETLKALVELDVDI  136
            G   L+     G   V   L K+GV ++  T  GYT LH A+ +G ++ +K L++   D+
Sbjct  668  GLTPLHLVAQEGHVPVADVLIKHGVMVDATTRMGYTPLHVASHYGNIKLVKFLLQHQADV  727

Query  137  EALNFREERARDVAARYSQTECVEFL  162
             A           AA+   T+ V  L
Sbjct  728  NAKTKLGYSPLHQAAQQGHTDIVTLL  753


 Score = 38.9 bits (89),  Expect = 0.56, Method: Compositional matrix adjust.
 Identities = 40/167 (23%), Positives = 63/167 (37%), Gaps = 7/167 (4%)

Query  63   HEQAMQLLLEE----DIV---GRNLLYAACMAGQSDVIRALAKYGVNLNEKTTRGYTLLH  115
            H + M+LLL+     D V   G   L+ A   G   +++ L + G + N    +  T LH
Sbjct  383  HVRVMELLLKTGASIDAVTESGLTPLHVASFMGHLPIVKNLLQRGASPNVSNVKVETPLH  442

Query  116  CAAAWGRLETLKALVELDVDIEALNFREERARDVAARYSQTECVEFLDWADARLTLKKYI  175
             AA  G  E  K L++    + A    ++     AAR   T  V+ L   +A   L
Sbjct  443  MAARAGHTEVAKYLLQNKAKVNAKAKDDQTPLHCAARIGHTNMVKLLLENNANPNLATTA  502

Query  176  AKVSLAVTDTEKGSGKLLKEDKNTILSACRAKNEWLETHTEASINEL  222
                L +   E     +L   +     AC  K  +   H  A   ++
Sbjct  503  GHTPLHIAAREGHVETVLALLEKEASQACMTKKGFTPLHVAAKYGKV  549


 Score = 36.6 bits (83),  Expect = 2.8, Method: Compositional matrix adjust.
 Identities = 27/107 (25%), Positives = 46/107 (42%), Gaps = 7/107 (6%)

Query  63   HEQAMQLLLEED-------IVGRNLLYAACMAGQSDVIRALAKYGVNLNEKTTRGYTLLH  115
            H   ++LLLE +         G   L+ A   G  + + AL +   +    T +G+T LH
Sbjct  482  HTNMVKLLLENNANPNLATTAGHTPLHIAAREGHVETVLALLEKEASQACMTKKGFTPLH  541

Query  116  CAAAWGRLETLKALVELDVDIEALNFREERARDVAARYSQTECVEFL  162
             AA +G++   + L+E D    A          VA  ++  + V+ L
Sbjct  542  VAAKYGKVRVAELLLERDAHPNAAGKNGLTPLHVAVHHNNLDIVKLL  588


 Score = 35.0 bits (79),  Expect = 8.5, Method: Compositional matrix adjust.
```

Identities = 19/54 (35%), Positives = 29/54 (53%), Gaps = 0/54 (0%)

```
Query  77   GRNLLYAACMAGQSDVIRALAKYGVNLNEKTTRGYTLLHCAAAWGRLETLKALV  130
            G   L+ A    Q +V R+L +YG + N ++ +G T LH AA  G  E +  L+
Sbjct  602  GYTPLHIAAKQNQVEVARSLLQYGGSANAESVQGVTPLHLAAQEGHAEMVALLL  655
```

>**pir||B35049** ankyrin 1, erythrocyte splice form 3 - human
Length=1856

Score = 53.1 bits (126), Expect = 3e-05, Method: Compositional matrix adjust.
Identities = 30/88 (34%), Positives = 49/88 (55%), Gaps = 6/88 (6%)

```
Query  50   GLQKIFEDPENPHHEQAMQLLLEEDIV------GRNLLYAACMAGQSDVIRALAKYGVNL  103
            GL  +     + H +  ++LL +E I+      G   L+ A +AGQ +V+R L  YG N+
Sbjct  45   GLNGLHLASKEGHVKMVVELLHKEIILETTTKKGNTALHIAALAGQDEVVRELVNYGANV  104

Query  104  NEKTTRGYTLLHCAAAWGRLETLKALVE  131
            N ++ +G+T L+ AA    LE +K L+E
Sbjct  105  NAQSQKGFTPLYMAAQENHLEVVKFLLE  132
```

Score = 46.6 bits (109), Expect = 0.003, Method: Compositional matrix adjust.
Identities = 28/87 (32%), Positives = 44/87 (50%), Gaps = 0/87 (0%)

```
Query  81   LYAACMAGQSDVIRALAKYGVNLNEKTTRGYTLLHCAAAWGRLETLKALVELDVDIEALN  140
            L+ A   G +++++ L +   N N  TT G+T LH AA  G +ET+ AL+E +     +
Sbjct  474  LHCAARIGHTNMVKLLLENNANPNLATTAGHTPLHIAAREGHVETVLALLEKEASQACMT  533

Query  141  FREERARDVAARYSQTECVEFLDWADA  167
             +      VAA+Y +    E L   DA
Sbjct  534  KKGFTPLHVAAKYGKVRVAELLLERDA  560
```

Score = 45.1 bits (105), Expect = 0.007, Method: Compositional matrix adjust.
Identities = 31/117 (26%), Positives = 52/117 (44%), Gaps = 7/117 (5%)

```
Query  63   HEQAMQLLLEED-------IVGRNLLYAACMAGQSDVIRALAKYGVNLNEKTTRGYTLLH  115
            H +  ++LL++        + G   L+ AC      V+  L K G +++  T  G T LH
Sbjct  350  HHRVAKVLLDKGAKPNSRALNGFTPLHIACKKNHVRVMELLLKTGASIDAVTESGLTPLH  409

Query  116  CAAAWGRLETLKALVELDVDIEALNFREERARDVAARYSQTECVEFLDWADARLTLK  172
             A+  G L  +K L++        N + E    +AAR   TE  ++L    A++  K
Sbjct  410  VASFMGHLPIVKNLLQRGASPNVSNVKVETPLHMAARAGHTEVAKYLLQNKAKVNAK  466
```

Score = 42.0 bits (97), Expect = 0.070, Method: Compositional matrix adjust.
Identities = 25/86 (29%), Positives = 39/86 (45%), Gaps = 0/86 (0%)

```
Query  77   GRNLLYAACMAGQSDVIRALAKYGVNLNEKTTRGYTLLHCAAAWGRLETLKALVELDVDI  136
            G   L+     G   V   L K+GV ++  T  GYT LH A+ +G ++ +K L++   D+
Sbjct  668  GLTPLHLVAQEGHVPVADVLIKHGVMVDATTRMGYTPLHVASHYGNIKLVKFLLQHQADV  727

Query  137  EALNFREERARDVAARYSQTECVEFL  162
             A           AA+   T+ V  L
Sbjct  728  NAKTKLGYSPLHQAAQQGHTDIVTLL  753
```

Score = 38.9 bits (89), Expect = 0.55, Method: Compositional matrix adjust.
Identities = 40/167 (23%), Positives = 63/167 (37%), Gaps = 7/167 (4%)

```
Query  63   HEQAMQLLLEE----DIV---GRNLLYAACMAGQSDVIRALAKYGVNLNEKTTRGYTLLH  115
            H + M+LLL+     D V   G   L+ A   G   +++ L + G + N    +  T LH
Sbjct  383  HVRVMELLLKTGASIDAVTESGLTPLHVASFMGHLPIVKNLLQRGASPNVSNVKVETPLH  442

Query  116  CAAAWGRLETLKALVELDVDIEALNFREERARDVAARYSQTECVEFLDWADARLTLKKYI  175
             AA  G   E K L++    + A    ++     AAR   T  V+ L   +A   L
Sbjct  443  MAARAGHTEVAKYLLQNKAKVNAKAKDDQTPLHCAARIGHTNMVKLLLENNANPNLATTA  502

Query  176  AKVSLAVTDTEKGSGKLLKEDKNTILSACRAKNEWLETHTEASINEL  222
                L +   E     +L   +     AC  K  +   H  A   ++
Sbjct  503  GHTPLHIAAREGHVETVLALLEKEASQACMTKKGFTPLHVAAKYGKV  549
```

Score = 36.6 bits (83), Expect = 2.8, Method: Compositional matrix adjust.
Identities = 27/107 (25%), Positives = 46/107 (42%), Gaps = 7/107 (6%)

```
Query  63   HEQAMQLLLEED-------IVGRNLLYAACMAGQSDVIRALAKYGVNLNEKTTRGYTLLH  115
            H   ++LLLE +         G   L+ A   G  + + AL +   +    T +G+T LH
Sbjct  482  HTNMVKLLLENNANPNLATTAGHTPLHIAAREGHVETVLALLEKEASQACMTKKGFTPLH  541

Query  116  CAAAWGRLETLKALVELDVDIEALNFREERARDVAARYSQTECVEFL  162
             AA +G++   + L+E D    A          VA  ++  + V+ L
Sbjct  542  VAAKYGKVRVAELLLERDAHPNAAGKNGLTPLHVAVHHNNLDIVKLL  588
```

Score = 35.0 bits (79), Expect = 8.4, Method: Compositional matrix adjust.
Identities = 19/54 (35%), Positives = 29/54 (53%), Gaps = 0/54 (0%)

```
Query  77   GRNLLYAACMAGQSDVIRALAKYGVNLNEKTTRGYTLLHCAAAWGRLETLKALV  130
            G   L+ A    Q +V R+L +YG + N ++ +G T LH AA  G  E +  L+
Sbjct  602  GYTPLHIAAKQNQVEVARSLLQYGGSANAESVQGVTPLHLAAQEGHAEMVALLL  655
```

>**gb|EDL00012.1|** mCG117548 [Mus musculus]
Length=1102

Score = 53.1 bits (126), Expect = 3e-05, Method: Compositional matrix adjust.
Identities = 40/135 (29%), Positives = 58/135 (42%), Gaps = 7/135 (5%)

```
Query  38   PLLQPALTGDVEGLQKIFEDPENPHHEQAMQLLLEE------DIVGRNLLYAACMAGQSD  91
            PLL      D  G   +     N H E    LL +       D   R  L+ A   G  D
Sbjct  128  PLLSSVNVSDRGGRTALHHAALNGHMEMVNLLLAKGANINAFDKKDRRALHWAAYMGHLD  187

Query  92   VIRALAKYGVNLNEKTTRGYTLLHCAAAWGRLETLKALVELDVDIEALNFREERARDVAA  151
            V+  L  +G  +  K  +GYT LH AA+ G++  +K L+ L V+I+ +N     A  +A
Sbjct  188  VVALLINHGAEVTCKDKKGYTPLHAAASNGQISVVKHLLNLGVEIDEINVYGNTALHIAC  247
```

```
Query  152  RYSQTECV-EFLDWA  165
                Q   V E +D+
Sbjct  248  YNGQDAVVNELIDYG  262


 Score = 43.5 bits (101),  Expect = 0.024, Method: Compositional matrix adjust.
 Identities = 33/104 (31%), Positives = 51/104 (49%), Gaps = 16/104 (15%)

Query  72   EEDIVGRNLLYAACMAGQSDVIRALAKYGVNLNEKTTRGYTLLHCAAA--WGRLETLKAL  129
            E ++ G   L+ AC  GQ  V+  L  YG N+N+    G+T LH AAA   G L  L+ L
Sbjct  234  EINVYGNTALHIACYNGQDAVVNELIDYGANVNQPNNSGFTPLHFAASTHGAL-CLELL  292

Query  130  VE--LDVDIE-----------ALNFREERARDVAARYSQTECVE  160
            V    DV+I+           A++ R  R++ +      + +CV+
Sbjct  293  VNNGADVNIQSKDGKSPLHMTAVHGRFTRSQTLIQNGGEIDCVD  336


 Score = 39.3 bits (90),  Expect = 0.45, Method: Compositional matrix adjust.
 Identities = 22/63 (34%), Positives = 31/63 (49%), Gaps = 0/63 (0%)

Query  74   DIVGRNLLYAACMAGQSDVIRALAKYGVNLNEKTTRGYTLLHCAAAWGRLETLKALVELD  133
            D  GR  L+AA   G  + I+ L   G + ++K   G T LH AAA       +KALV
Sbjct  420  DTFGRTCLHAAAAGGNVECIKLLQSSGADFHKKDKCGRTPLHYAAANCHFHCIKALVTTG  479

Query  134  VDI  136
             ++
Sbjct  480  ANV  482


 Score = 37.0 bits (84),  Expect = 2.3, Method: Compositional matrix adjust.
 Identities = 33/124 (26%), Positives = 51/124 (41%), Gaps = 27/124 (21%)

Query  36   KNPLLQPALTGDVEGLQKIFEDPENPH--------------------HEQAMQLLLEE--  73
            + PL     + G     L+ + + E  +NP                    H  A+ LLLE+
Sbjct  653  RTPLHASVINGHTLCLRLLLETADNPEVVDVKDAKGQTPLMLAVAYGHIDAVSLLLEKEA  712

Query  74   -----DIVGRNLLYAACMAGQSDVIRALAKYGVNLNEKTTRGYTLLHCAAAWGRLETLKA  128
                 DIVG   L+    M G   + ++ L +    ++   K +RG T LH AAA G    L
Sbjct  713  NVDAVDIVGCTALHRGIMTGHEECVQMLLEQEASILCKDSRGRTPLHYAAARGHATWLNE  772

Query  129  LVEL  132
            L+++
Sbjct  773  LLQI  776


 Score = 35.0 bits (79),  Expect = 7.8, Method: Compositional matrix adjust.
 Identities = 44/159 (27%), Positives = 66/159 (41%), Gaps = 15/159 (9%)

Query  14   FFSQQEEENEEEEAQEPEETGPKNPLLQPALTGDVEGLQKIFEDPENPHHEQAMQLLLEE  73
             FS +    +    E    P+  G +   L    A  G+VE ++ +       + H          ++
Sbjct  403  LFSNEHVLSAGFEIDTPDTFG-RTCLHAAAAGGNVECIKLLQSSGADFH---------KK  452

Query  74   DIVGRNLLYAACMAGQSDVIRALAKYGVNLNEKTTRGYTLLHCAAAWGRLETLKALVELD  133
            D  GR  L+ A          I+AL   G N+NE    G T LH AAA           L
Sbjct  453  DKCGRTPLHYAAANCHFHCIKALVTTGANVNETDDWGRTALHYAAASDMDRNKMILGNAH  512

Query  134  VDIEALNFREERARDVAARYSQTECVEFLDWADARLTLK  172
             + E L    ERAR+V  + +    C+EFL    DA  +++
Sbjct  513  DNSEEL----ERAREVKEKDAAL-CLEFLLQNDANPSIR  546
```

>**ref|XP_539957.2|** PREDICTED: similar to ankyrin 1 isoform 3 [Canis familiaris]
Length=1881

**GENE ID: 482842 ANK1** | ankyrin 1, erythrocytic [Canis lupus familiaris]

```
 Score = 53.1 bits (126),  Expect = 3e-05, Method: Compositional matrix adjust.
 Identities = 30/88 (34%), Positives = 49/88 (55%), Gaps = 6/88 (6%)

Query  50   GLQKIFEDPENPHHEQAMQLLLEEDIV------GRNLLYAACMAGQSDVIRALAKYGVNL  103
            GL   +     +   H +   ++LL +E I+       G    L+ A +AGQ +V+R L  YG N+
Sbjct  71   GLNGLHLASKEGHVKMVVELLHKEIILETTTKKGNTALHIAALAGQDEVVRELVNYGANV  130

Query  104  NEKTTRGYTLLHCAAAWGRLETLKALVE  131
            N ++ +G+T L+ AA    LE +K L+E
Sbjct  131  NAQSQKGFTPLYMAAQENHLEVVKFLLE  158


 Score = 46.2 bits (108),  Expect = 0.003, Method: Compositional matrix adjust.
 Identities = 28/87 (32%), Positives = 44/87 (50%), Gaps = 0/87 (0%)

Query  81   LYAACMAGQSDVIRALAKYGVNLNEKTTRGYTLLHCAAAWGRLETLKALVELDVDIEALN  140
            L+ A   G +++++ L +   N N  TT G+T LH AA  G++ET  AL+E +      +
Sbjct  500  LHCAARIGHTNMVKLLLENNANPNLATTAGHTPLHIAAREGQVETALALLEKEASQACMT  559

Query  141  FREERARDVAARYSQTECVEFLDWADA  167
             +       VAA+Y +     E L   DA
Sbjct  560  KKGFTPLHVAAKYGKVRVAELLLGRDA  586


 Score = 45.1 bits (105),  Expect = 0.008, Method: Compositional matrix adjust.
 Identities = 31/117 (26%), Positives = 52/117 (44%), Gaps = 7/117 (5%)

Query  63   HEQAMQLLLEED-------IVGRNLLYAACMAGQSDVIRALAKYGVNLNEKTTRGYTLLH  115
            H +  ++LL++          + G   L+ AC       V+  L K G +++  T  G T LH
Sbjct  376  HHRVAKVLLDKGAKPNSRALNGFTPLHIACKKNHIRVMELLLKTGASIDAVTESGLTPLH  435

Query  116  CAAAWGRLETLKALVELDVDIEALNFREERARDVAARYSQTECVEFLDWADARLTLK  172
             A+  G L  +K L++          N + E    +AAR   TE  ++L    A++  K
Sbjct  436  VASFMGHLPIVKNLLQRGASPNVSNVKVETPLHMAARAGHTEVAKYLLQNKAKVNAK  492


 Score = 43.5 bits (101),  Expect = 0.022, Method: Compositional matrix adjust.
 Identities = 25/86 (29%), Positives = 40/86 (46%), Gaps = 0/86 (0%)

Query  77   GRNLLYAACMAGQSDVIRALAKYGVNLNEKTTRGYTLLHCAAAWGRLETLKALVELDVDI  136
            G   L+      G   V   L K+GV ++  T  GYT LH A+ +G ++ +K L++   D+
Sbjct  694  GLTPLHLVAQEGHVPVADVLIKHGVTVDATTRMGYTPLHVASHYGNIKLVKFLLQHQADV  753
```

```
Query  137  EALNFREERARDVAARYSQTECVEFL  162
             A   +       AA+   T+ V  L
Sbjct  754  NAKTKQGYSPLHQAAQQGHTDIVTLL  779
```

```
 Score = 37.0 bits (84),  Expect = 1.8, Method: Compositional matrix adjust.
 Identities = 40/167 (23%), Positives = 62/167 (37%), Gaps = 7/167 (4%)

Query  63   HEQAMQLLLEE----DIV---GRNLLYAACMAGQSDVIRALAKYGVNLNEKTTRGYTLLH  115
            H + M+LLL+     D V   G   L+ A   G   +++ L + G + N    +  T LH
Sbjct  409  HIRVMELLLKTGASIDAVTESGLTPLHVASFMGHLPIVKNLLQRGASPNVSNVKVETPLH  468

Query  116  CAAAWGRLETLKALVELDVDIEALNFREERARDVAARYSQTECVEFLDWADARLTLKKYI  175
             AA  G  E  K L++    + A    ++     AAR   T  V+ L   +A   L
Sbjct  469  MAARAGHTEVAKYLLQNKAKVNAKAKDDQTPLHCAARIGHTNMVKLLLENNANPNLATTA  528

Query  176  AKVSLAVTDTEKGSGKLLKEDKNTILSACRAKNEWLETHTEASINEL  222
                L +   E      L   +     AC  K  +   H  A   ++
Sbjct  529  GHTPLHIAAREGQVETALALLEKEASQACMTKKGFTPLHVAAKYGKV  575
```

```
 Score = 35.0 bits (79),  Expect = 8.6, Method: Compositional matrix adjust.
 Identities = 19/54 (35%), Positives = 29/54 (53%), Gaps = 0/54 (0%)

Query  77   GRNLLYAACMAGQSDVIRALAKYGVNLNEKTTRGYTLLHCAAAWGRLETLKALV  130
            G   L+ A    Q +V R+L +YG + N ++ +G T LH AA  G  E +  L+
Sbjct  628  GYTPLHIAAKQNQMEVARSLLQYGGSANAESVQGVTPLHLAAQEGHAEMVALLL  681
```

>**ref|XP_001139287.1|** PREDICTED: ankyrin 1 isoform 1 [Pan troglodytes]
Length=1856

**GENE ID: 736634 ANK1** | ankyrin 1, erythrocytic [Pan troglodytes]

```
 Score = 53.1 bits (126),  Expect = 3e-05, Method: Compositional matrix adjust.
 Identities = 30/88 (34%), Positives = 49/88 (55%), Gaps = 6/88 (6%)

Query  50   GLQKIFEDPENPHHEQAMQLLLEEDIV------GRNLLYAACMAGQSDVIRALAKYGVNL  103
            GL  +    +  H +  ++LL +E I+      G   L+ A +AGQ +V+R L  YG N+
Sbjct  45   GLNGLHLASKEGHVKMVVELLHKEIILETTTKGNTALHIAALAGQDEVVRELVNYGANV  104

Query  104  NEKTTRGYTLLHCAAAWGRLETLKALVE  131
            N ++ +G+T L+ AA    LE +K L+E
Sbjct  105  NAQSQKGFTPLYMAAQENHLEVVKFLLE  132
```

```
 Score = 46.6 bits (109),  Expect = 0.003, Method: Compositional matrix adjust.
 Identities = 28/87 (32%), Positives = 44/87 (50%), Gaps = 0/87 (0%)

Query  81   LYAACMAGQSDVIRALAKYGVNLNEKTTRGYTLLHCAAAWGRLETLKALVELDVDIEALN  140
            L+ A   G +++++ L +   N N  TT G+T LH AA  G +ET+ AL+E +      +
Sbjct  474  LHCAARIGHTNMVKLLLENNANPNLATTAGHTPLHIAAREGHVETVLALLEKEASQACMT  533

Query  141  FREERARDVAARYSQTECVEFLDWADA  167
             +      VAA+Y +    E L   DA
Sbjct  534  KKGFTPLHVAAKYGKVRVAELLLERDA  560
```

```
 Score = 45.1 bits (105),  Expect = 0.007, Method: Compositional matrix adjust.
 Identities = 31/117 (26%), Positives = 52/117 (44%), Gaps = 7/117 (5%)

Query  63   HEQAMQLLLEED-------IVGRNLLYAACMAGQSDVIRALAKYGVNLNEKTTRGYTLLH  115
            H +  ++LL++        + G   L+ AC      V+  L K G +++  T  G T LH
Sbjct  350  HHRVAKVLLDKGAKPNSRALNGFTPLHIACKKNHVRVMELLLKTGASIDAVTESGLTPLH  409

Query  116  CAAAWGRLETLKALVELDVDIEALNFREERARDVAARYSQTECVEFLDWADARLTLK  172
             A+  G L  +K L++        N + E    +AAR   TE  ++L    A++  K
Sbjct  410  VASFMGHLPIVKNLLQRGASPNVSNVKVETPLHMAARAGHTEVAKYLLQNKAKVNAK  466
```

```
 Score = 42.0 bits (97),  Expect = 0.071, Method: Compositional matrix adjust.
 Identities = 25/86 (29%), Positives = 39/86 (45%), Gaps = 0/86 (0%)

Query  77   GRNLLYAACMAGQSDVIRALAKYGVNLNEKTTRGYTLLHCAAAWGRLETLKALVELDVDI  136
            G   L+     G   V   L K+GV ++  T  GYT LH A+ +G ++ +K L++   D+
Sbjct  668  GLTPLHLVAQEGHVPVADVLIKHGVMVDATTRMGYTPLHVASHYGNIKLVKFLLQHQADV  727

Query  137  EALNFREERARDVAARYSQTECVEFL  162
             A           AA+   T+ V  L
Sbjct  728  NAKTKLGYSPLHQAAQQGHTDIVTLL  753
```

```
 Score = 38.9 bits (89),  Expect = 0.57, Method: Compositional matrix adjust.
 Identities = 40/167 (23%), Positives = 63/167 (37%), Gaps = 7/167 (4%)

Query  63   HEQAMQLLLEE----DIV---GRNLLYAACMAGQSDVIRALAKYGVNLNEKTTRGYTLLH  115
            H + M+LLL+     D V   G   L+ A   G   +++ L + G + N    +  T LH
Sbjct  383  HVRVMELLLKTGASIDAVTESGLTPLHVASFMGHLPIVKNLLQRGASPNVSNVKVETPLH  442

Query  116  CAAAWGRLETLKALVELDVDIEALNFREERARDVAARYSQTECVEFLDWADARLTLKKYI  175
             AA  G  E  K L++    + A    ++     AAR   T  V+ L   +A   L
Sbjct  443  MAARAGHTEVAKYLLQNKAKVNAKAKDDQTPLHCAARIGHTNMVKLLLENNANPNLATTA  502

Query  176  AKVSLAVTDTEKGSGKLLKEDKNTILSACRAKNEWLETHTEASINEL  222
                L +   E     +L   +     AC  K  +   H  A   ++
Sbjct  503  GHTPLHIAAREGHVETVLALLEKEASQACMTKKGFTPLHVAAKYGKV  549
```

```
 Score = 36.6 bits (83),  Expect = 2.8, Method: Compositional matrix adjust.
 Identities = 27/107 (25%), Positives = 46/107 (42%), Gaps = 7/107 (6%)

Query  63   HEQAMQLLLEED-------IVGRNLLYAACMAGQSDVIRALAKYGVNLNEKTTRGYTLLH  115
            H   ++LLLE +         G   L+ A   G  + + AL +   +    T +G+T LH
Sbjct  482  HTNMVKLLLENNANPNLATTAGHTPLHIAAREGHVETVLALLEKEASQACMTKKGFTPLH  541

Query  116  CAAAWGRLETLKALVELDVDIEALNFREERARDVAARYSQTECVEFL  162
             AA +G++   + L+E D    A          VA  ++  + V+ L
```

```
Sbjct  542  VAAKYGKVRVAELLLERDAHPNAAGKNGLTPLHVAVHHNNLDIVKLL  588


 Score = 35.0 bits (79),  Expect = 8.7, Method: Compositional matrix adjust.
 Identities = 19/54 (35%), Positives = 29/54 (53%), Gaps = 0/54 (0%)

Query  77   GRNLLYAACMAGQSDVIRALAKYGVNLNEKTTRGYTLLHCAAAWGRLETLKALV  130
            G   L+ A    Q +V R+L +YG + N ++ +G T LH AA  G  E +  L+
Sbjct  602  GYTPLHIAAKQNQVEVARSLLQYGGSANAESVQGVTPLHLAAQEGHAEMVALLL  655
```

>**ref|XP_001139606.1|** PREDICTED: ankyrin 1 isoform 4 [Pan troglodytes]
Length=1881

**GENE ID: 736634 ANK1** | ankyrin 1, erythrocytic [Pan troglodytes]

```
 Score = 53.1 bits (126),  Expect = 3e-05, Method: Compositional matrix adjust.
 Identities = 30/88 (34%), Positives = 49/88 (55%), Gaps = 6/88 (6%)

Query  50   GLQKIFEDPENPHHEQAMQLLLEEDIV------GRNLLYAACMAGQSDVIRALAKYGVNL  103
            GL  +    +  H +  ++LL +E I+      G   L+ A +AGQ +V+R L  YG N+
Sbjct  45   GLNGLHLASKEGHVKMVVELLHKEIILETTTKKGNTALHIAALAGQDEVVRELVNYGANV  104

Query  104  NEKTTRGYTLLHCAAAWGRLETLKALVE  131
            N ++ +G+T L+ AA    LE +K L+E
Sbjct  105  NAQSQKGFTPLYMAAQENHLEVVKFLLE  132


 Score = 46.6 bits (109),  Expect = 0.003, Method: Compositional matrix adjust.
 Identities = 28/88 (31%), Positives = 44/88 (50%), Gaps = 0/88 (0%)

Query  81   LYAACMAGQSDVIRALAKYGVNLNEKTTRGYTLLHCAAAWGRLETLKALVELDVDIEALN  140
            L+ A   G +++++ L +   N N  TT G+T LH AA  G +ET+ AL+E +     +
Sbjct  474  LHCAARIGHTNMVKLLLENNANPNLATTAGHTPLHIAAREGHVETVLALLEKEASQACMT  533

Query  141  FREERARDVAARYSQTECVEFLDWADAR  168
             +      VAA+Y +    E L   DA
Sbjct  534  KKGFTPLHVAAKYGKVRVAELLLERDAH  561


 Score = 45.4 bits (106),  Expect = 0.006, Method: Compositional matrix adjust.
 Identities = 31/117 (26%), Positives = 52/117 (44%), Gaps = 7/117 (5%)

Query  63   HEQAMQLLLEED-------IVGRNLLYAACMAGQSDVIRALAKYGVNLNEKTTRGYTLLH  115
            H +  ++LL++        + G   L+ AC      V+  L K G +++  T  G T LH
Sbjct  350  HHRVAKVLLDKGAKPNSRALNGFTPLHIACKKNHVRVMELLLKTGASIDAVTESGLTPLH  409

Query  116  CAAAWGRLETLKALVELDVDIEALNFREERARDVAARYSQTECVEFLDWADARLTLK  172
             A+  G L  +K L++        N + E    +AAR   TE  ++L    A++  K
Sbjct  410  VASFMGHLPIVKNLLQRGASPNVSNVKVETPLHMAARAGHTEVAKYLLQNKAKVNAK  466


 Score = 42.0 bits (97),  Expect = 0.068, Method: Compositional matrix adjust.
 Identities = 25/86 (29%), Positives = 39/86 (45%), Gaps = 0/86 (0%)

Query  77   GRNLLYAACMAGQSDVIRALAKYGVNLNEKTTRGYTLLHCAAAWGRLETLKALVELDVDI  136
            G   L+     G   V   L K+GV ++  T  GYT LH A+ +G ++ +K L++   D+
Sbjct  668  GLTPLHLVAQEGHVPVADVLIKHGVMVDATTRMGYTPLHVASHYGNIKLVKFLLQHQADV  727

Query  137  EALNFREERARDVAARYSQTECVEFL  162
             A            AA+   T+ V  L
Sbjct  728  NAKTKLGYSPLHQAAQQGHTDIVTLL  753


 Score = 38.9 bits (89),  Expect = 0.57, Method: Compositional matrix adjust.
 Identities = 40/167 (23%), Positives = 63/167 (37%), Gaps = 7/167 (4%)

Query  63   HEQAMQLLLEE----DIV---GRNLLYAACMAGQSDVIRALAKYGVNLNEKTTRGYTLLH  115
            H + M+LLL+     D V   G   L+ A   G   +++ L + G + N    +  T LH
Sbjct  383  HVRVMELLLKTGASIDAVTESGLTPLHVASFMGHLPIVKNLLQRGASPNVSNVKVETPLH  442

Query  116  CAAAWGRLETLKALVELDVDIEALNFREERARDVAARYSQTECVEFLDWADARLTLKKYI  175
             AA  G  E  K L++    + A    ++     AAR   T  V+ L   +A   L
Sbjct  443  MAARAGHTEVAKYLLQNKAKVNAKAKDDQTPLHCAARIGHTNMVKLLLENNANPNLATTA  502

Query  176  AKVSLAVTDTEKGSGKLLKEDKNTILSACRAKNEWLETHTEASINEL  222
                L +   E     +L   +     AC  K  +   H  A   ++
Sbjct  503  GHTPLHIAAREGHVETVLALLEKEASQACMTKKGFTPLHVAAKYGKV  549


 Score = 36.6 bits (83),  Expect = 2.7, Method: Compositional matrix adjust.
 Identities = 27/107 (25%), Positives = 46/107 (42%), Gaps = 7/107 (6%)

Query  63   HEQAMQLLLEED-------IVGRNLLYAACMAGQSDVIRALAKYGVNLNEKTTRGYTLLH  115
            H   ++LLLE +         G   L+ A   G  + + AL +   +    T +G+T LH
Sbjct  482  HTNMVKLLLENNANPNLATTAGHTPLHIAAREGHVETVLALLEKEASQACMTKKGFTPLH  541

Query  116  CAAAWGRLETLKALVELDVDIEALNFREERARDVAARYSQTECVEFL  162
             AA +G++   + L+E D    A          VA  ++  + V+ L
Sbjct  542  VAAKYGKVRVAELLLERDAHPNAAGKNGLTPLHVAVHHNNLDIVKLL  588


 Score = 35.0 bits (79),  Expect = 8.2, Method: Compositional matrix adjust.
 Identities = 19/54 (35%), Positives = 29/54 (53%), Gaps = 0/54 (0%)

Query  77   GRNLLYAACMAGQSDVIRALAKYGVNLNEKTTRGYTLLHCAAAWGRLETLKALV  130
            G   L+ A    Q +V R+L +YG + N ++ +G T LH AA  G  E +  L+
Sbjct  602  GYTPLHIAAKQNQVEVARSLLQYGGSANAESVQGVTPLHLAAQEGHAEMVALLL  655
```

>**ref|NP_065209.2|** ankyrin 1 isoform 1 [Homo sapiens]
**sp|P16157.3|ANK1_HUMAN** RecName: Full=Ankyrin-1; AltName: Full=Erythrocyte ankyrin; AltName: Full=Ankyrin-R
**gb|EAW63246.1|** ankyrin 1, erythrocytic, isoform CRA_f [Homo sapiens]
**gb|AAI56402.1|** Ankyrin 1, erythrocytic [synthetic construct]
Length=1881

```
 GENE ID: 286 ANK1 | ankyrin 1, erythrocytic [Homo sapiens]
(Over 10 PubMed links)

 Score = 53.1 bits (126),  Expect = 3e-05, Method: Compositional matrix adjust.
 Identities = 30/88 (34%), Positives = 49/88 (55%), Gaps = 6/88 (6%)

Query  50   GLQKIFEDPENPHHEQAMQLLLEEDIV------GRNLLYAACMAGQSDVIRALAKYGVNL  103
            GL  +    +  H +  ++LL +E I+      G   L+ A +AGQ +V+R L  YG N+
Sbjct  45   GLNGLHLASKEGHVKMVVELLHKEIILETTTKKGNTALHIAALAGQDEVVRELVNYGANV  104

Query  104  NEKTTRGYTLLHCAAAWGRLETLKALVE  131
            N ++ +G+T L+ AA    LE +K L+E
Sbjct  105  NAQSQKGFTPLYMAAQENHLEVVKFLLE  132


 Score = 46.6 bits (109),  Expect = 0.003, Method: Compositional matrix adjust.
 Identities = 28/88 (31%), Positives = 44/88 (50%), Gaps = 0/88 (0%)

Query  81   LYAACMAGQSDVIRALAKYGVNLNEKTTRGYTLLHCAAAWGRLETLKALVELDVDIEALN  140
            L+ A   G +++++ L +   N N  TT G+T LH AA  G +ET+ AL+E +     +
Sbjct  474  LHCAARIGHTNMVKLLLENNANPNLATTAGHTPLHIAAREGHVETVLALLEKEASQACMT  533

Query  141  FREERARDVAARYSQTECVEFLDWADAR  168
             +      VAA+Y +    E L   DA
Sbjct  534  KKGFTPLHVAAKYGKVRVAELLLERDAH  561


 Score = 45.1 bits (105),  Expect = 0.007, Method: Compositional matrix adjust.
 Identities = 31/117 (26%), Positives = 52/117 (44%), Gaps = 7/117 (5%)

Query  63   HEQAMQLLLEED-------IVGRNLLYAACMAGQSDVIRALAKYGVNLNEKTTRGYTLLH  115
            H +  ++LL++        + G   L+ AC      V+  L K G +++  T  G T LH
Sbjct  350  HHRVAKVLLDKGAKPNSRALNGFTPLHIACKKNHVRVMELLLKTGASIDAVTESGLTPLH  409

Query  116  CAAAWGRLETLKALVELDVDIEALNFREERARDVAARYSQTECVEFLDWADARLTLK  172
             A+  G L  +K L++        N + E    +AAR   TE  ++L    A++  K
Sbjct  410  VASFMGHLPIVKNLLQRGASPNVSNVKVETPLHMAARAGHTEVAKYLLQNKAKVNAK  466


 Score = 42.0 bits (97),  Expect = 0.069, Method: Compositional matrix adjust.
 Identities = 25/86 (29%), Positives = 39/86 (45%), Gaps = 0/86 (0%)

Query  77   GRNLLYAACMAGQSDVIRALAKYGVNLNEKTTRGYTLLHCAAAWGRLETLKALVELDVDI  136
            G   L+     G   V   L K+GV ++  T  GYT LH A+ +G ++ +K L++   D+
Sbjct  668  GLTPLHLVAQEGHVPVADVLIKHGVMVDATTRMGYTPLHVASHYGNIKLVKFLLQHQADV  727

Query  137  EALNFREERARDVAARYSQTECVEFL  162
             A           AA+   T+ V  L
Sbjct  728  NAKTKLGYSPLHQAAQQGHTDIVTLL  753


 Score = 38.9 bits (89),  Expect = 0.58, Method: Compositional matrix adjust.
 Identities = 40/167 (23%), Positives = 63/167 (37%), Gaps = 7/167 (4%)

Query  63   HEQAMQLLLEE----DIV---GRNLLYAACMAGQSDVIRALAKYGVNLNEKTTRGYTLLH  115
            H + M+LLL+     D V   G   L+ A   G   +++ L + G + N    +  T LH
Sbjct  383  HVRVMELLLKTGASIDAVTESGLTPLHVASFMGHLPIVKNLLQRGASPNVSNVKVETPLH  442

Query  116  CAAAWGRLETLKALVELDVDIEALNFREERARDVAARYSQTECVEFLDWADARLTLKKYI  175
             AA  G  E  K L++    + A    ++     AAR   T  V+ L   +A   L
Sbjct  443  MAARAGHTEVAKYLLQNKAKVNAKAKDDQTPLHCAARIGHTNMVKLLLENNANPNLATTA  502

Query  176  AKVSLAVTDTEKGSGKLLKEDKNTILSACRAKNEWLETHTEASINEL  222
                L +   E     +L   +     AC  K  +   H  A   ++
Sbjct  503  GHTPLHIAAREGHVETVLALLEKEASQACMTKKGFTPLHVAAKYGKV  549


 Score = 36.6 bits (83),  Expect = 2.7, Method: Compositional matrix adjust.
 Identities = 27/107 (25%), Positives = 46/107 (42%), Gaps = 7/107 (6%)

Query  63   HEQAMQLLLEED-------IVGRNLLYAACMAGQSDVIRALAKYGVNLNEKTTRGYTLLH  115
            H   ++LLLE +         G   L+ A   G  + + AL +    +     T +G+T LH
Sbjct  482  HTNMVKLLLENNANPNLATTAGHTPLHIAAREGHVETVLALLEKEASQACMTKKGFTPLH  541

Query  116  CAAAWGRLETLKALVELDVDIEALNFREERARDVAARYSQTECVEFL  162
             AA +G++   + L+E D    A          VA  ++  + V+ L
Sbjct  542  VAAKYGKVRVAELLLERDAHPNAAGKNGLTPLHVAVHHNNLDIVKLL  588


 Score = 35.0 bits (79),  Expect = 8.1, Method: Compositional matrix adjust.
 Identities = 19/54 (35%), Positives = 29/54 (53%), Gaps = 0/54 (0%)

Query  77   GRNLLYAACMAGQSDVIRALAKYGVNLNEKTTRGYTLLHCAAAWGRLETLKALV  130
            G   L+ A    Q +V R+L +YG + N ++ +G T LH AA  G  E +  L+
Sbjct  602  GYTPLHIAAKQNQVEVARSLLQYGGSANAESVQGVTPLHLAAQEGHAEMVALLL  655


>ref|XP_001099591.1| UG PREDICTED: similar to ankyrin 1 isoform 1 [Macaca mulatta]
Length=1914

 GENE ID: 710904 LOC710904 | similar to ankyrin 1 isoform 1 [Macaca mulatta]

 Score = 53.1 bits (126),  Expect = 3e-05, Method: Compositional matrix adjust.
 Identities = 30/88 (34%), Positives = 49/88 (55%), Gaps = 6/88 (6%)

Query  50   GLQKIFEDPENPHHEQAMQLLLEEDIV------GRNLLYAACMAGQSDVIRALAKYGVNL  103
            GL  +    +  H +  ++LL +E I+      G   L+ A +AGQ +V+R L  YG N+
Sbjct  78   GLNGLHLASKEGHVKMVVELLHKEIILETTTKKGNTALHIAALAGQDEVVRELVNYGANV  137

Query  104  NEKTTRGYTLLHCAAAWGRLETLKALVE  131
            N ++ +G+T L+ AA    LE +K L+E
Sbjct  138  NAQSQKGFTPLYMAAQENHLEVVKFLLE  165


 Score = 47.0 bits (110),  Expect = 0.002, Method: Compositional matrix adjust.
 Identities = 28/88 (31%), Positives = 44/88 (50%), Gaps = 0/88 (0%)
```

```
Query  81   LYAACMAGQSDVIRALAKYGVNLNEKTTRGYTLLHCAAAWGRLETLKALVELDVDIEALN  140
            L+ A   G +++++ L +   N N  TT G+T LH AA  G +ET+ AL+E +     +
Sbjct  507  LHCAARIGHTNMVKLLLENNANPNLATTAGHTPLHIAAREGHVETVLALLEKEASQACMT  566

Query  141  FREERARDVAARYSQTECVEFLDWADAR  168
             +      VAA+Y +    E L   DA
Sbjct  567  KKGFTPLHVAAKYGKVRVAELLLEQDAH  594


 Score = 45.4 bits (106),  Expect = 0.006, Method: Compositional matrix adjust.
 Identities = 31/117 (26%), Positives = 52/117 (44%), Gaps = 7/117 (5%)

Query  63   HEQAMQLLLEED-------IVGRNLLYAACMAGQSDVIRALAKYGVNLNEKTTRGYTLLH  115
            H +  ++LL++         + G   L+ AC      V+  L K G +++  T  G T LH
Sbjct  383  HHRVAKVLLDKGAKPNSRALNGFTPLHIACKKNHVRVMELLLKTGASIDAVTESGLTPLH  442

Query  116  CAAAWGRLETLKALVELDVDIEALNFREERARDVAARYSQTECVEFLDWADARLTLK  172
             A+  G L  +K L++         N + E    +AAR   TE  ++L    A++  K
Sbjct  443  VASFMGHLPIVKNLLQRGASPNVSNVKVETPLHMAARAGHTEVAKYLLQNKAKVNAK  499


 Score = 42.0 bits (97),  Expect = 0.057, Method: Compositional matrix adjust.
 Identities = 25/86 (29%), Positives = 39/86 (45%), Gaps = 0/86 (0%)

Query  77   GRNLLYAACMAGQSDVIRALAKYGVNLNEKTTRGYTLLHCAAAWGRLETLKALVELDVDI  136
            G   L+     G   V   L K+GV ++  T   GYT LH A+ +G ++ +K L++   D+
Sbjct  701  GLTPLHLVAQEGHVPVADVLIKHGVMVDATTRMGYTPLHVASHYGNIKLVKFLLQHQADV  760

Query  137  EALNFREERARDVAARYSQTECVEFL  162
             A            AA+   T+ V  L
Sbjct  761  NAKTKLGYSPLHQAAQQGHTDVVTLL  786


 Score = 38.9 bits (89),  Expect = 0.54, Method: Compositional matrix adjust.
 Identities = 40/167 (23%), Positives = 63/167 (37%), Gaps = 7/167 (4%)

Query  63   HEQAMQLLLEE----DIV---GRNLLYAACMAGQSDVIRALAKYGVNLNEKTTRGYTLLH  115
            H + M+LLL+     D V   G   L+ A   G   +++ L + G + N    +  T LH
Sbjct  416  HVRVMELLLKTGASIDAVTESGLTPLHVASFMGHLPIVKNLLQRGASPNVSNVKVETPLH  475

Query  116  CAAAWGRLETLKALVELDVDIEALNFREERARDVAARYSQTECVEFLDWADARLTLKKYI  175
             AA  G  E  K L++     + A     ++      AAR   T  V+ L   +A   L
Sbjct  476  MAARAGHTEVAKYLLQNKAKVNAKAKDDQTPLHCAARIGHTNMVKLLLENNANPNLATTA  535

Query  176  AKVSLAVTDTEKGSGKLLKEDKNTILSACRAKNEWLETHTEASINEL  222
                L +   E      +L    +      AC  K  +    H  A   ++
Sbjct  536  GHTPLHIAAREGHVETVLALLEKEASQACMTKKGFTPLHVAAKYGKV  582


 Score = 37.4 bits (85),  Expect = 1.7, Method: Compositional matrix adjust.
 Identities = 20/54 (37%), Positives = 29/54 (53%), Gaps = 0/54 (0%)

Query  77   GRNLLYAACMAGQSDVIRALAKYGVNLNEKTTRGYTLLHCAAAWGRLETLKALV  130
            G   L+ A     Q DV R+L +YG + N ++ +G T LH AA  G  E +  L+
Sbjct  635  GYTPLHIAAKQNQVDVARSLLQYGGSANAESVQGVTPLHLAAQEGHAEMVALLL  688


 Score = 36.6 bits (83),  Expect = 2.9, Method: Compositional matrix adjust.
 Identities = 27/107 (25%), Positives = 46/107 (42%), Gaps = 7/107 (6%)

Query  63   HEQAMQLLLEED-------IVGRNLLYAACMAGQSDVIRALAKYGVNLNEKTTRGYTLLH  115
            H   ++LLLE +           G   L+ A   G  + + AL +    +     T +G+T LH
Sbjct  515  HTNMVKLLLENNANPNLATTAGHTPLHIAAREGHVETVLALLEKEASQACMTKKGFTPLH  574

Query  116  CAAAWGRLETLKALVELDVDIEALNFREERARDVAARYSQTECVEFL  162
             AA +G++    + L+E D    A           VA  ++   + V+ L
Sbjct  575  VAAKYGKVRVAELLLEQDAHPNAAGKNGLTPLHVAVHHNNLDIVKLL  621


>ref|NP_001074902.1|  ankyrin repeat domain 44 [Mus musculus]
Length=1011

 GENE ID: 329154 Ankrd44 | ankyrin repeat domain 44 [Mus musculus]
(10 or fewer PubMed links)

 Score = 53.1 bits (126),  Expect = 3e-05, Method: Compositional matrix adjust.
 Identities = 40/135 (29%), Positives = 58/135 (42%), Gaps = 7/135 (5%)

Query  38   PLLQPALTGDVEGLQKIFEDPENPHHEQAMQLLLEE------DIVGRNLLYAACMAGQSD  91
            PLL      D  G    +      N H E     LL +        D    R  L+ A    G  D
Sbjct  128  PLLSSVNVSDRGGRTALHHAALNGHMEMVNLLLAKGANINAFDKKDRRALHWAAYMGHLD  187

Query  92   VIRALAKYGVNLNEKTTRGYTLLHCAAAWGRLETLKALVELDVDIEALNFREERARDVAA  151
            V+   L   +G   +  K   +GYT LH AA+ G++   +K L+ L V+I+ +N      A  +A
Sbjct  188  VVALLINHGAEVTCKDKKGYTPLHAAASNGQISVVKHLLNLGVEIDEINVYGNTALHIAC  247

Query  152  RYSQTECV-EFLDWA  165
               Q    V E +D+
Sbjct  248  YNGQDAVVNELIDYG  262


 Score = 43.1 bits (100),  Expect = 0.029, Method: Compositional matrix adjust.
 Identities = 33/104 (31%), Positives = 51/104 (49%), Gaps = 16/104 (15%)

Query  72   EEDIVGRNLLYAACMAGQSDVIRALAKYGVNLNEKTTRGYTLLHCAAA--WGRLETLKAL  129
            E ++ G     LH+ AC   GQ   V+   L   YG N+N++     G+T LH AAA    G L   L+ L
Sbjct  234  EINVYGNTALHIACYNGQDAVVNELIDYGANVNQPNNSGFTPLHFAAASTHGAL-CLELL  292

Query  130  VE--LDVDIE-----------ALNFREERARDVAARYSQTECVE  160
            V     DV+I+              A++ R   R++ +      + +CV+
Sbjct  293  VNNGADVNIQSKDGKSPLHMTAVHGRFTRSQTLIQNGGEIDCVD  336


 Score = 38.9 bits (89),  Expect = 0.49, Method: Compositional matrix adjust.
 Identities = 22/63 (34%), Positives = 31/63 (49%), Gaps = 0/63 (0%)

Query  74   DIVGRNLLYAACMAGQSDVIRALAKYGVNLNEKTTRGYTLLHCAAAWGRLETLKALVELD  133
            D   GR   L+AA    G   + I+ L     G + ++K     G T LH AAA         +KALV
```

```
Sbjct  420   DTFGRTCLHAAAAGGNVECIKLLQSSGADFHKKDKCGRTPLHYAAANCHFHCIKALVTTG  479

Query  134   VDI  136
              ++
Sbjct  480   ANV  482


 Score = 36.6 bits (83),  Expect = 2.5, Method: Compositional matrix adjust.
 Identities = 33/124 (26%), Positives = 51/124 (41%), Gaps = 27/124 (21%)

Query  36    KNPLLQPALTGDVEGLQKIFEDPENPH--------------------HEQAMQLLLEE--  73
             + PL    + G    L+ + E  +NP                     H  A+ LLLE+
Sbjct  653   RTPLHASVINGHTLCLRLLLETADNPEVVDVKDAKGQTPLMLAVAYGHIDAVSLLLEKEA  712

Query  74    -----DIVGRNLLYAACMAGQSDVIRALAKYGVNLNEKTTRGYTLLHCAAAWGRLETLKA  128
                  DIVG   L+   M G  + ++ L +   ++  K +RG T LH AAA G    L
Sbjct  713   NVDAVDIVGCTALHRGIMTGHEECVQMLLEQEASILCKDSRGRTPLHYAAARGHATWLNE  772

Query  129   LVEL  132
             L+++
Sbjct  773   LLQI  776


 Score = 35.0 bits (79),  Expect = 7.9, Method: Compositional matrix adjust.
 Identities = 44/159 (27%), Positives = 66/159 (41%), Gaps = 15/159 (9%)

Query  14    FFSQQEEENEEEEAQEPEETGPKNPLLQPALTGDVEGLQKIFEDPENPHHEQAMQLLLEE  73
              FS +    +    E    P+   G +   L    A  G+VE ++ +      + H        ++
Sbjct  403   LFSNEHVLSAGFEIDTPDTFG-RTCLHAAAAGGNVECIKLLQSSGADFH---------KK  452

Query  74    DIVGRNLLYAACMAGQSDVIRALAKYGVNLNEKTTRGYTLLHCAAAWGRLETLKALVELD  133
             D  GR  L+ A         I+AL   G N+NE    G T LH AAA           L
Sbjct  453   DKCGRTPLHYAAANCHFHCIKALVTTGANVNETDDWGRTALHYAAASDMDRNKMILGNAH  512

Query  134   VDIEALNFREERARDVAARYSQTECVEFLDWADARLTLK  172
              + E L    ERAR+V  + +   C+EFL   DA  +++
Sbjct  513   DNSEEL----ERAREVKEKDAAL-CLEFLLQNDANPSIR  546


>sp|B2RXR6.1|ANR44_MOUSE  RecName: Full=Serine/threonine-protein phosphatase 6 regulatory
ankyrin repeat subunit B; Short=Serine/threonine-protein
phosphatase 6 regulatory subunit ARS-B; Short=PP6-ARS-B; AltName:
Full=Ankyrin repeat domain-containing protein 44
 gb|AAI57952.1|  Ankrd44 protein [Mus musculus]
 gb|AAI72102.1|  Ankrd44 protein [Mus musculus]
Length=993

 GENE ID: 329154 Ankrd44 | ankyrin repeat domain 44 [Mus musculus]
(10 or fewer PubMed links)

 Score = 53.1 bits (126),  Expect = 3e-05, Method: Compositional matrix adjust.
 Identities = 40/135 (29%), Positives = 58/135 (42%), Gaps = 7/135 (5%)

Query  38    PLLQPALTGDVEGLQKIFEDPENPHHEQAMQLLLEE------DIVGRNLLYAACMAGQSD  91
             PLL       D  G   +     N H E    LL +        D    R  L+ A    G  D
Sbjct  128   PLLSSVNVSDRGGRTALHHAALNGHMEMVNLLLAKGANINAFDKKDRRALHWAAYMGHLD  187

Query  92    VIRALAKYGVNLNEKTTRGYTLLHCAAAWGRLETLKALVELDVDIEALNFREERARDVAA  151
             V+  L  +G  +  K  +GYT LH AA+ G++  +K L+ L V+I+ +N     A  +A
Sbjct  188   VVALLINHGAEVTCKDKKGYTPLHAAASNGQISVVKHLLNLGVEIDEINVYGNTALHIAC  247

Query  152   RYSQTECV-EFLDWA  165
                Q   V E +D+
Sbjct  248   YNGQDAVVNELIDYG  262


 Score = 43.1 bits (100),  Expect = 0.027, Method: Compositional matrix adjust.
 Identities = 33/104 (31%), Positives = 51/104 (49%), Gaps = 16/104 (15%)

Query  72    EEDIVGRNLLYAACMAGQSDVIRALAKYGVNLNEKTTRGYTLLHCAAA--WGRLETLKAL  129
             E ++ G    L+ AC   GQ   V+   L   YG N+N++     G+T LH AAA    G L   L+ L
Sbjct  234   EINVYGNTALHIACYNGQDAVVNELIDYGANVNQPNNSGFTPLHFAAASTHGAL-CLELL  292

Query  130   VE--LDVDIE-----------ALNFREERARDVAARYSQTECVE  160
             V    DV+I+             A++ R   R++ +       + +CV+
Sbjct  293   VNNGADVNIQSKDGKSPLHMTAVHGRFTRSQTLIQNGGEIDCVD  336


 Score = 38.9 bits (89),  Expect = 0.50, Method: Compositional matrix adjust.
 Identities = 22/63 (34%), Positives = 31/63 (49%), Gaps = 0/63 (0%)

Query  74    DIVGRNLLYAACMAGQSDVIRALAKYGVNLNEKTTRGYTLLHCAAAWGRLETLKALVELD  133
             D  GR  L+AA    G  + I+ L    G + ++K    G T LH AAA       +KALV
Sbjct  402   DTFGRTCLHAAAAGGNVECIKLLQSSGADFHKKDKCGRTPLHYAAANCHFHCIKALVTTG  461

Query  134   VDI  136
              ++
Sbjct  462   ANV  464


 Score = 36.6 bits (83),  Expect = 2.5, Method: Compositional matrix adjust.
 Identities = 33/124 (26%), Positives = 51/124 (41%), Gaps = 27/124 (21%)

Query  36    KNPLLQPALTGDVEGLQKIFEDPENPH--------------------HEQAMQLLLEE--  73
             + PL    + G    L+ + E  +NP                     H  A+ LLLE+
Sbjct  635   RTPLHASVINGHTLCLRLLLETADNPEVVDVKDAKGQTPLMLAVAYGHIDAVSLLLEKEA  694

Query  74    -----DIVGRNLLYAACMAGQSDVIRALAKYGVNLNEKTTRGYTLLHCAAAWGRLETLKA  128
                  DIVG   L+   M G  + ++ L +   ++  K +RG T LH AAA G    L
Sbjct  695   NVDAVDIVGCTALHRGIMTGHEECVQMLLEQEASILCKDSRGRTPLHYAAARGHATWLNE  754

Query  129   LVEL  132
             L+++
Sbjct  755   LLQI  758


 Score = 35.4 bits (80),  Expect = 6.0, Method: Compositional matrix adjust.
```

```
 Identities = 22/78 (28%), Positives = 33/78 (42%), Gaps = 4/78 (5%)

Query  81   LYAACMAGQSDVIRALAKYGVNLNEKTTRGYTLLHCAAAWGRLETLKALVELDVDIEALN  140
            L+ A +   SD  R L   G  ++   T G T LH AAA G +E +K L     D
Sbjct  376  LHLAALNAHSDCCRKLLSSGFEIDTPDTFGRTCLHAAAAGGNVECIKLLQSSGADFH---  432

Query  141  FREERARDVAARYSQTEC  158
             ++++       Y+   C
Sbjct  433  -KKDKCGRTPLHYAAANC  449
```

**>ref|NP_001135918.1|** ankyrin 1 isoform 9 [Homo sapiens]
Length=1897

**GENE ID: 286 ANK1** | ankyrin 1, erythrocytic [Homo sapiens]
(Over 10 PubMed links)

```
 Score = 53.1 bits (126),  Expect = 3e-05, Method: Compositional matrix adjust.
 Identities = 30/88 (34%), Positives = 49/88 (55%), Gaps = 6/88 (6%)

Query  50   GLQKIFEDPENPHHEQAMQLLLEEDIV------GRNLLYAACMAGQSDVIRALAKYGVNL  103
            GL  +    +  H +  ++LL +E I+      G   L+ A +AGQ +V+R L  YG N+
Sbjct  78   GLNGLHLASKEGHVKMVVELLHKEIILETTTKKGNTALHIAALAGQDEVVRELVNYGANV  137

Query  104  NEKTTRGYTLLHCAAAWGRLETLKALVE  131
            N ++ +G+T L+ AA    LE +K L+E
Sbjct  138  NAQSQKGFTPLYMAAQENHLEVVKFLLE  165


 Score = 46.6 bits (109),  Expect = 0.003, Method: Compositional matrix adjust.
 Identities = 28/87 (32%), Positives = 44/87 (50%), Gaps = 0/87 (0%)

Query  81   LYAACMAGQSDVIRALAKYGVNLNEKTTRGYTLLHCAAAWGRLETLKALVELDVDIEALN  140
            L+ A   G +++++ L +   N N  TT G+T LH AA  G +ET+ AL+E +     +
Sbjct  507  LHCAARIGHTNMVKLLLENNANPNLATTAGHTPLHIAAREGHVETVLALLEKEASQACMT  566

Query  141  FREERARDVAARYSQTECVEFLDWADA  167
             +      VAA+Y +   E L   DA
Sbjct  567  KKGFTPLHVAAKYGKVRVAELLLERDA  593


 Score = 45.1 bits (105),  Expect = 0.007, Method: Compositional matrix adjust.
 Identities = 31/117 (26%), Positives = 52/117 (44%), Gaps = 7/117 (5%)

Query  63   HEQAMQLLLEED-------IVGRNLLYAACMAGQSDVIRALAKYGVNLNEKTTRGYTLLH  115
            H +  ++LL++        + G   L+ AC      V+  L K G +++  T  G T LH
Sbjct  383  HHRVAKVLLDKGAKPNSRALNGFTPLHIACKKNHVRVMELLLKTGASIDAVTESGLTPLH  442

Query  116  CAAAWGRLETLKALVELDVDIEALNFREERARDVAARYSQTECVEFLDWADARLTLK  172
             A+  G L  +K L++        N + E    +AAR   TE  ++L    A++  K
Sbjct  443  VASFMGHLPIVKNLLQRGASPNVSNVKVETPLHMAARAGHTEVAKYLLQNKAKVNAK  499


 Score = 41.6 bits (96),  Expect = 0.072, Method: Compositional matrix adjust.
 Identities = 25/86 (29%), Positives = 39/86 (45%), Gaps = 0/86 (0%)

Query  77   GRNLLYAACMAGQSDVIRALAKYGVNLNEKTTRGYTLLHCAAAWGRLETLKALVELDVDI  136
            G   L+     G   V   L K+GV ++  T  GYT LH A+ +G ++ +K L++   D+
Sbjct  701  GLTPLHLVAQEGHVPVADVLIKHGVMVDATTRMGYTPLHVASHYGNIKLVKFLLQHQADV  760

Query  137  EALNFREERARDVAARYSQTECVEFL  162
             A           AA+   T+ V  L
Sbjct  761  NAKTKLGYSPLHQAAQQGHTDIVTLL  786


 Score = 38.5 bits (88),  Expect = 0.63, Method: Compositional matrix adjust.
 Identities = 40/167 (23%), Positives = 63/167 (37%), Gaps = 7/167 (4%)

Query  63   HEQAMQLLLEE----DIV---GRNLLYAACMAGQSDVIRALAKYGVNLNEKTTRGYTLLH  115
            H + M+LLL+     D V   G   L+ A   G   +++ L + G + N    +  T LH
Sbjct  416  HVRVMELLLKTGASIDAVTESGLTPLHVASFMGHLPIVKNLLQRGASPNVSNVKVETPLH  475

Query  116  CAAAWGRLETLKALVELDVDIEALNFREERARDVAARYSQTECVEFLDWADARLTLKKYI  175
             AA  G  E  K L++    + A    ++     AAR   T  V+ L   +A   L
Sbjct  476  MAARAGHTEVAKYLLQNKAKVNAKAKDDQTPLHCAARIGHTNMVKLLLENNANPNLATTA  535

Query  176  AKVSLAVTDTEKGSGKLLKEDKNTILSACRAKNEWLETHTEASINEL  222
                L +   E     +L   +     AC  K  +   H  A   ++
Sbjct  536  GHTPLHIAAREGHVETVLALLEKEASQACMTKKGFTPLHVAAKYGKV  582


 Score = 36.6 bits (83),  Expect = 2.8, Method: Compositional matrix adjust.
 Identities = 27/107 (25%), Positives = 46/107 (42%), Gaps = 7/107 (6%)

Query  63   HEQAMQLLLEED-------IVGRNLLYAACMAGQSDVIRALAKYGVNLNEKTTRGYTLLH  115
            H   ++LLLE +         G   L+ A   G  + + AL +   +    T +G+T LH
Sbjct  515  HTNMVKLLLENNANPNLATTAGHTPLHIAAREGHVETVLALLEKEASQACMTKKGFTPLH  574

Query  116  CAAAWGRLETLKALVELDVDIEALNFREERARDVAARYSQTECVEFL  162
             AA +G++   + L+E D    A          VA  ++  + V+ L
Sbjct  575  VAAKYGKVRVAELLLERDAHPNAAGKNGLTPLHVAVHHNNLDIVKLL  621


 Score = 35.0 bits (79),  Expect = 8.4, Method: Compositional matrix adjust.
 Identities = 19/54 (35%), Positives = 29/54 (53%), Gaps = 0/54 (0%)

Query  77   GRNLLYAACMAGQSDVIRALAKYGVNLNEKTTRGYTLLHCAAAWGRLETLKALV  130
            G   L+ A     Q +V R+L +YG + N ++ +G T LH AA  G  E +  L+
Sbjct  635  GYTPLHIAAKQNQVEVARSLLQYGGSANAESVQGVTPLHLAAQEGHAEMVALLL  688
```

**>dbj|BAD92655.1|** ankyrin 1 isoform 4 variant [Homo sapiens]
Length=1899

**GENE ID: 286 ANK1** | ankyrin 1, erythrocytic [Homo sapiens]
(Over 10 PubMed links)

```
 Score = 53.1 bits (126),  Expect = 3e-05, Method: Compositional matrix adjust.
 Identities = 30/88 (34%), Positives = 49/88 (55%), Gaps = 6/88 (6%)

Query  50   GLQKIFEDPENPHHEQAMQLLLEEDIV------GRNLLYAACMAGQSDVIRALAKYGVNL  103
            GL  +     + H +  ++LL +E I+      G   L+ A +AGQ +V+R L  YG N+
Sbjct  80   GLNGLHLASKEGHVKMVVELLHKEIILETTTKKGNTALHIAALAGQDEVVRELVNYGANV  139

Query  104  NEKTTRGYTLLHCAAAWGRLETLKALVE  131
            N ++ +G+T L+ AA    LE +K L+E
Sbjct  140  NAQSQKGFTPLYMAAQENHLEVVKFLLE  167


 Score = 46.6 bits (109),  Expect = 0.003, Method: Compositional matrix adjust.
 Identities = 28/87 (32%), Positives = 44/87 (50%), Gaps = 0/87 (0%)

Query  81   LYAACMAGQSDVIRALAKYGVNLNEKTTRGYTLLHCAAAWGRLETLKALVELDVDIEALN  140
            L+ A   G +++++ L +   N N  TT G+T LH AA  G +ET+ AL+E +     +
Sbjct  509  LHCAARIGHTNMVKLLLENNANPNLATTAGHTPLHIAAREGHVETVLALLEKEASQACMT  568

Query  141  FREERARDVAARYSQTECVEFLDWADA  167
             +       VAA+Y +    E L   DA
Sbjct  569  KKGFTPLHVAAKYGKVRVAELLLERDA  595


 Score = 45.1 bits (105),  Expect = 0.007, Method: Compositional matrix adjust.
 Identities = 31/117 (26%), Positives = 52/117 (44%), Gaps = 7/117 (5%)

Query  63   HEQAMQLLLEED-------IVGRNLLYAACMAGQSDVIRALAKYGVNLNEKTTRGYTLLH  115
            H +  ++LL++         + G   L+ AC       V+  L K G +++  T  G T LH
Sbjct  385  HHRVAKVLLDKGAKPNSRALNGFTPLHIACKKNHVRVMELLLKTGASIDAVTESGLTPLH  444

Query  116  CAAAWGRLETLKALVELDVDIEALNFREERARDVAARYSQTECVEFLDWADARLTLK  172
             A+  G L  +K L++         N + E    +AAR   TE  ++L    A++  K
Sbjct  445  VASFMGHLPIVKNLLQRGASPNVSNVKVETPLHMAARAGHTEVAKYLLQNKAKVNAK  501


 Score = 42.0 bits (97),  Expect = 0.070, Method: Compositional matrix adjust.
 Identities = 25/86 (29%), Positives = 39/86 (45%), Gaps = 0/86 (0%)

Query  77   GRNLLYAACMAGQSDVIRALAKYGVNLNEKTTRGYTLLHCAAAWGRLETLKALVELDVDI  136
            G   L+      G    V   L K+GV ++   T   GYT LH A+ +G ++ +K L++    D+
Sbjct  703  GLTPLHLVAQEGHVPVADVLIKHGVMVDATTRMGYTPLHVASHYGNIKLVKFLLQHQADV  762

Query  137  EALNFREERARDVAARYSQTECVEFL  162
             A            AA+   T+ V  L
Sbjct  763  NAKTKLGYSPLHQAAQQGHTDIVTLL  788


 Score = 38.9 bits (89),  Expect = 0.58, Method: Compositional matrix adjust.
 Identities = 40/167 (23%), Positives = 63/167 (37%), Gaps = 7/167 (4%)

Query  63   HEQAMQLLLEE----DIV---GRNLLYAACMAGQSDVIRALAKYGVNLNEKTTRGYTLLH  115
            H + M+LLL+      D V   G   L+ A   G   +++ L + G + N    +  T LH
Sbjct  418  HVRVMELLLKTGASIDAVTESGLTPLHVASFMGHLPIVKNLLQRGASPNVSNVKVETPLH  477

Query  116  CAAAWGRLETLKALVELDVDIEALNFREERARDVAARYSQTECVEFLDWADARLTLKKYI  175
             AA  G  E  K L++    + A    ++      AAR    T  V+ L    +A   L
Sbjct  478  MAARAGHTEVAKYLLQNKAKVNAKAKDDQTPLHCAARIGHTNMVKLLLENNANPNLATTA  537

Query  176  AKVSLAVTDTEKGSGKLLKEDKNTILSACRAKNEWLETHTEASINEL  222
                 L +   E      +L    +      AC  K  +    H  A    ++
Sbjct  538  GHTPLHIAAREGHVETVLALLEKEASQACMTKKGFTPLHVAAKYGKV  584


 Score = 36.6 bits (83),  Expect = 2.7, Method: Compositional matrix adjust.
 Identities = 27/107 (25%), Positives = 46/107 (42%), Gaps = 7/107 (6%)

Query  63   HEQAMQLLLEED-------IVGRNLLYAACMAGQSDVIRALAKYGVNLNEKTTRGYTLLH  115
            H   ++LLLE +         G   L+ A   G  + + AL +   +    T +G+T LH
Sbjct  517  HTNMVKLLLENNANPNLATTAGHTPLHIAAREGHVETVLALLEKEASQACMTKKGFTPLH  576

Query  116  CAAAWGRLETLKALVELDVDIEALNFREERARDVAARYSQTECVEFL  162
             AA +G++    + L+E D    A            VA  ++  + V+ L
Sbjct  577  VAAKYGKVRVAELLLERDAHPNAAGKNGLTPLHVAVHHNNLDIVKLL  623


 Score = 35.0 bits (79),  Expect = 8.0, Method: Compositional matrix adjust.
 Identities = 19/54 (35%), Positives = 29/54 (53%), Gaps = 0/54 (0%)

Query  77   GRNLLYAACMAGQSDVIRALAKYGVNLNEKTTRGYTLLHCAAAWGRLETLKALV  130
            G   L+ A     Q +V R+L +YG + N ++ +G T LH AA  G  E +  L+
Sbjct  637  GYTPLHIAAKQNQVEVARSLLQYGGSANAESVQGVTPLHLAAQEGHAEMVALLL  690


>ref|XP_001790685.1| UG PREDICTED: myosin XVI [Bos taurus]
Length=1717

 GENE ID: 617611 MYO16 | myosin XVI [Bos taurus]

 Score = 53.1 bits (126),  Expect = 3e-05, Method: Compositional matrix adjust.
 Identities = 28/75 (37%), Positives = 43/75 (57%), Gaps = 0/75 (0%)

Query  77   GRNLLYAACMAGQSDVIRALAKYGVNLNEKTTRGYTLLHCAAAWGRLETLKALVELDVDI  136
            G  LL+ AC +G   +V+   L ++G +LN    + +T LH AA +G+    +K L+     +
Sbjct  280  GVTLLHMACASGYKEVVSLLLEHGGDLNAADNQHWTPLHLAAKYGQANLVKLLLMHQANP  339

Query  137  EALNFREERARDVAA  151
              LN  EE+A DVAA
Sbjct  340  HLLNCHEEKASDVAA  354


 Score = 41.2 bits (95),  Expect = 0.12, Method: Compositional matrix adjust.
 Identities = 23/70 (32%), Positives = 35/70 (50%), Gaps = 0/70 (0%)

Query  93   IRALAKYGVNLNEKTTRGYTLLHCAAAWGRLETLKALVELDVDIEALNFREERARDVAAR  152
            ++      G N+NE+   G TLLH A A G   E +  L+E    D+ A + +       +AA+
Sbjct  263  VKHFLSCGGNVNERNDDGVTLLHMACASGYKEVVSLLLEHGGDLNAADNQHWTPLHLAAK  322
```

```
Query  153  YSQTECVEFL  162
            Y Q   V+ L
Sbjct  323  YGQANLVKLL  332


>emb|CAQ51694.1|  ankyrin repeat and SAM domain containing 1 [Mus musculus]
 emb|CAQ51766.1|  ankyrin repeat and SAM domain containing 1 [Mus musculus]
Length=1168

 Score = 53.1 bits (126),  Expect = 3e-05, Method: Compositional matrix adjust.
 Identities = 37/117 (31%), Positives = 55/117 (47%), Gaps = 10/117 (8%)

Query  63   HEQAMQLLLEEDIV-------GRNLLYAACMAGQSDVIRALAKYG---VNLNEKTTRGYT  112
            H   +++LL  D +       G   L+ A   G + ++R L + G     +NE+     T
Sbjct  88   HRDVVEVLLRNDALTNVADSKGCYPLHLAAWKGDAQIVRLLIQQGPSHTRVNEQNNDNET  147

Query  113  LLHCAAAWGRLETLKALVELDVDIEALNFREERARDVAARYSQTECVEFLDWADARL  169
             LHCAA +G  E +KAL+E   D    N + E   D+AA Y + E V+ L  A   L
Sbjct  148  ALHCAAQYGHTEVVKALLEELTDPTMRNNKFETPLDLAALYGRLEVVKLLLGAHPNL  204


 Score = 37.0 bits (84),  Expect = 2.3, Method: Compositional matrix adjust.
 Identities = 34/119 (28%), Positives = 58/119 (48%), Gaps = 12/119 (10%)

Query  63   HEQAMQLLLEE--DIVGRN-----LLYAACMAGQSDVIRALAKYGVNLNEKTTRGYTLLH  115
            H + ++ LLEE  D   RN      L  A + G+ +V++ L     NL   +TR +T LH
Sbjct  157  HTEVVKALLEELTDPTMRNNKFETPLDLAALYGRLEVVKLLLGAHPNLLSCSTRKHTPLH  216

Query  116  CAAAWGRLETLKALVELDVDIEALNFREERARDV--AARYSQTECVEFLDWADARLTLK  172
             AA  G    ++ L++  +D    N++ E    +  AA + +T+ V+ L  A   + +K
Sbjct  217  LAARNGHKAVVQVLLDAGMDS---NYQTEMGSALHEAALFGKTDVVQILLAAGIDVNIK  272


 Score = 36.6 bits (83),  Expect = 2.5, Method: Compositional matrix adjust.
 Identities = 30/125 (24%), Positives = 58/125 (46%), Gaps = 6/125 (4%)

Query  38   PLLQPALTGDVEGLQKIFEDPENPHHEQAMQLLLEEDIVGRNLLYAACMAGQSDVIRALA  97
            PL   A  GD + ++ + +  + P H +      E++      L+ A   G ++V++AL
Sbjct  112  PLHLAAWKGDAQIVRLLIQ--QGPSHTRVN----EQNNDNETALHCAAQYGHTEVVKALL  165

Query  98   KYGVNLNEKTTRGYTLLHCAAAWGRLETLKALVELDVDIEALNFREERARDVAARYSQTE  157
            +   +   +  +  T L  AA +GRLE +K L+    ++ + + R+     +AAR
Sbjct  166  EELTDPTMRNNKFETPLDLAALYGRLEVVKLLLGAHPNLLSCSTRKHTPLHLAARNGHKA  225

Query  158  CVEFL  162
             V+ L
Sbjct  226  VVQVL  230


>ref|XP_002007551.1|  GI12323 [Drosophila mojavensis]
 gb|EDW18027.1|  GI12323 [Drosophila mojavensis]
Length=1163

 GENE ID: 6581829 Dmoj\GI12323 | GI12323 gene product from transcript GI12323-RA
[Drosophila mojavensis] (10 or fewer PubMed links)

 Score = 52.8 bits (125),  Expect = 3e-05, Method: Compositional matrix adjust.
 Identities = 38/124 (30%), Positives = 63/124 (50%), Gaps = 9/124 (7%)

Query  76   VGRNLLYAACMAGQSDVIRALAKYGVNLNEKTTRGYTLLHCAAAWGRLETLKALVELDVD  135
             G   L+ A   G + V+R L   G N++ +   G+T LH AA WG+ E  + LVE   D
Sbjct  205  TGATALHVAAAKGYAKVMRLLLAAGCNVDRQDNDGWTPLHAAAHWGQKEAAEMLVESLAD  264

Query  136  IEALNFREERARDVAARYSQTECVEFLDWADARLTLKKY----IAKVSLAVTD-TEKGSG  190
            ++  N+  +   DVA R    + V+FL+   A    K+     I+++S A+ +  +K
Sbjct  265  MDITNYAGQTCIDVADR----KMVKFLEELRANKRNKRRPSSQISRISDAIENHVDKTRT  320

Query  191  KLLK  194
            KL++
Sbjct  321  KLVR  324


 Score = 46.6 bits (109),  Expect = 0.003, Method: Compositional matrix adjust.
 Identities = 27/106 (25%), Positives = 54/106 (50%), Gaps = 6/106 (5%)

Query  64   EQAMQLLLE------EDIVGRNLLYAACMAGQSDVIRALAKYGVNLNEKTTRGYTLLHCA  117
            E+ +QLL +       ++ G   L+ AC+  + D++  L ++G ++N +   G+T LH
Sbjct  60   EEVVQLLEQGADINTANVDGLTALHQACIDDKLDMVEFLIEHGADINRQDNEGWTPLHAT  119

Query  118  AAWGRLETLKALVELDVDIEALNFREERARDVAARYSQTECVEFLD  163
            A+ G +     LVE + D+ A+N   + A D+A        + +++
Sbjct  120  ASCGFVSIACYLVEHNADVAAVNSDGDLALDLAIDVQHMPMINYME  165


 Score = 38.5 bits (88),  Expect = 0.72, Method: Compositional matrix adjust.
 Identities = 21/77 (27%), Positives = 37/77 (48%), Gaps = 4/77 (5%)

Query  60   NPHHEQAMQLLLEEDIVGRNLLYAACMAGQSDVIRALAKYGVNLNEKTTRGYTLLHCAAA  119
            +P HE + ++      V      AAC++G  + +  L + G ++N     G T LH A
Sbjct  33   SPRHEHSRRIKFSSGCV----FLAACLSGDKEEVVQLLEQGADINTANVDGLTALHQACI  88

Query  120  WGRLETLKALVELDVDI  136
              +L+ ++ L+E   DI
Sbjct  89   DDKLDMVEFLIEHGADI  105


>ref|XP_001327095.1|  ankyrin repeat protein [Trichomonas vaginalis G3]
 gb|EAY14872.1|  ankyrin repeat protein, putative [Trichomonas vaginalis G3]
Length=789

 GENE ID: 4772871 TVAG_411380 | ankyrin repeat protein
[Trichomonas vaginalis G3] (10 or fewer PubMed links)

 Score = 52.8 bits (125),  Expect = 3e-05, Method: Compositional matrix adjust.
 Identities = 32/100 (32%), Positives = 48/100 (48%), Gaps = 0/100 (0%)
```

```
Query  73    EDIVGRNLLYAACMAGQSDVIRALAKYGVNLNEKTTRGYTLLHCAAAWGRLETLKALVEL  132
             +D+ GR+ LY A    Q +++  +  +G NLN     GYT LH AA    LE  + L+
Sbjct  336  KDLKGRDALYIATKQNQKNILEEILTHGANLNATYMEGYTALHIAAEKTSLEAAEILISH  395

Query  133  DVDIEALNFREERARDVAARYSQTECVEFLDWADARLTLK  172
              + I+ ++     A  +A     TE  EFL    A L +K
Sbjct  396  GIKIDEIDRNGRTALHIAVENMYTELSEFLITHGANLDIK  435
```

>**ref|NP_065210.2|** ankyrin 1 isoform 2 [Homo sapiens]
**gb|EAW63245.1|** ankyrin 1, erythrocytic, isoform CRA_e [Homo sapiens]
Length=1719

**GENE ID: 286 ANK1** | ankyrin 1, erythrocytic [Homo sapiens]
(Over 10 PubMed links)

Score = 52.8 bits (125), Expect = 3e-05, Method: Compositional matrix adjust.
Identities = 30/88 (34%), Positives = 49/88 (55%), Gaps = 6/88 (6%)

```
Query  50   GLQKIFEDPENPHHEQAMQLLLEEDIV------GRNLLYAACMAGQSDVIRALAKYGVNL  103
            GL  +    +  H +  ++LL +E I+      G   L+ A +AGQ +V+R L  YG N+
Sbjct  45   GLNGLHLASKEGHVKMVVELLHKEIILETTTKKGNTALHIAALAGQDEVVRELVNYGANV  104

Query  104  NEKTTRGYTLLHCAAAWGRLETLKALVE  131
            N ++ +G+T L+ AA    LE +K L+E
Sbjct  105  NAQSQKGFTPLYMAAQENHLEVVKFLLE  132
```

Score = 46.6 bits (109), Expect = 0.002, Method: Compositional matrix adjust.
Identities = 28/87 (32%), Positives = 44/87 (50%), Gaps = 0/87 (0%)

```
Query  81   LYAACMAGQSDVIRALAKYGVNLNEKTTRGYTLLHCAAAWGRLETLKALVELDVDIEALN  140
            L+ A   G +++++ L +   N N  TT G+T LH AA  G +ET+ AL+E +     +
Sbjct  474  LHCAARIGHTNMVKLLLENNANPNLATTAGHTPLHIAAREGHVETVLALLEKEASQACMT  533

Query  141  FREERARDVAARYSQTECVEFLDWADA  167
             +      VAA+Y +    E L   DA
Sbjct  534  KKGFTPLHVAAKYGKVRVAELLLERDA  560
```

Score = 45.4 bits (106), Expect = 0.006, Method: Compositional matrix adjust.
Identities = 31/117 (26%), Positives = 52/117 (44%), Gaps = 7/117 (5%)

```
Query  63   HEQAMQLLLEED-------IVGRNLLYAACMAGQSDVIRALAKYGVNLNEKTTRGYTLLH  115
            H +  ++LL++        + G   L+ AC       V+  L K G +++  T  G T LH
Sbjct  350  HHRVAKVLLDKGAKPNSRALNGFTPLHIACKKNHVRVMELLLKTGASIDAVTESGLTPLH  409

Query  116  CAAAWGRLETLKALVELDVDIEALNFREERARDVAARYSQTECVEFLDWADARLTLK  172
             A+  G L  +K L++        N + E    +AAR   TE  ++L    A++  K
Sbjct  410  VASFMGHLPIVKNLLQRGASPNVSNVKVETPLHMAARAGHTEVAKYLLQNKAKVNAK  466
```

Score = 42.0 bits (97), Expect = 0.072, Method: Compositional matrix adjust.
Identities = 25/86 (29%), Positives = 39/86 (45%), Gaps = 0/86 (0%)

```
Query  77   GRNLLYAACMAGQSDVIRALAKYGVNLNEKTTRGYTLLHCAAAWGRLETLKALVELDVDI  136
            G   L+     G   V   L K+GV ++  T  GYT LH A+ +G ++ +K L++   D+
Sbjct  668  GLTPLHLVAQEGHVPVADVLIKHGVMVDATTRMGYTPLHVASHYGNIKLVKFLLQHQADV  727

Query  137  EALNFREERARDVAARYSQTECVEFL  162
             A           AA+   T+ V  L
Sbjct  728  NAKTKLGYSPLHQAAQQGHTDIVTLL  753
```

Score = 38.9 bits (89), Expect = 0.50, Method: Compositional matrix adjust.
Identities = 40/167 (23%), Positives = 63/167 (37%), Gaps = 7/167 (4%)

```
Query  63   HEQAMQLLLEE----DIV---GRNLLYAACMAGQSDVIRALAKYGVNLNEKTTRGYTLLH  115
            H + M+LLL+     D V   G   L+ A   G   +++ L + G + N    +  T LH
Sbjct  383  HVRVMELLLKTGASIDAVTESGLTPLHVASFMGHLPIVKNLLQRGASPNVSNVKVETPLH  442

Query  116  CAAAWGRLETLKALVELDVDIEALNFREERARDVAARYSQTECVEFLDWADARLTLKKYI  175
             AA  G  E  K L++    + A    ++      AAR   T  V+ L   +A   L
Sbjct  443  MAARAGHTEVAKYLLQNKAKVNAKAKDDQTPLHCAARIGHTNMVKLLLENNANPNLATTA  502

Query  176  AKVSLAVTDTEKGSGKLLKEDKNTILSACRAKNEWLETHTEASINEL  222
                L +   E     +L   +     AC  K  +   H  A   ++
Sbjct  503  GHTPLHIAAREGHVETVLALLEKEASQACMTKKGFTPLHVAAKYGKV  549
```

Score = 36.6 bits (83), Expect = 2.9, Method: Compositional matrix adjust.
Identities = 27/107 (25%), Positives = 46/107 (42%), Gaps = 7/107 (6%)

```
Query  63   HEQAMQLLLEED-------IVGRNLLYAACMAGQSDVIRALAKYGVNLNEKTTRGYTLLH  115
            H   ++LLLE +         G   L+ A   G  + + AL +   +    T +G+T LH
Sbjct  482  HTNMVKLLLENNANPNLATTAGHTPLHIAAREGHVETVLALLEKEASQACMTKKGFTPLH  541

Query  116  CAAAWGRLETLKALVELDVDIEALNFREERARDVAARYSQTECVEFL  162
             AA +G++   + L+E D    A          VA  ++   + V+ L
Sbjct  542  VAAKYGKVRVAELLLERDAHPNAAGKNGLTPLHVAVHHNNLDIVKLL  588
```

Score = 34.7 bits (78), Expect = 9.2, Method: Compositional matrix adjust.
Identities = 19/54 (35%), Positives = 29/54 (53%), Gaps = 0/54 (0%)

```
Query  77   GRNLLYAACMAGQSDVIRALAKYGVNLNEKTTRGYTLLHCAAAWGRLETLKALV  130
            G   L+ A    Q +V R+L +YG + N ++ +G T LH AA  G  E +  L+
Sbjct  602  GYTPLHIAAKQNQVEVARSLLQYGGSANAESVQGVTPLHLAAQEGHAEMVALLL  655
```

>**prf||1605244A** erythrocyte ankyrin
Length=1881

Score = 52.8 bits (125), Expect = 3e-05, Method: Compositional matrix adjust.
Identities = 30/88 (34%), Positives = 49/88 (55%), Gaps = 6/88 (6%)

```
Query  50   GLQKIFEDPENPHHEQAMQLLLEEDIV------GRNLLYAACMAGQSDVIRALAKYGVNL  103
```

```
            GL  +     +  H +  ++LL +E I+       G    L+ A +AGQ +V+R L  YG N+
Sbjct  45   GLNGLHLASKEGHVKMVVELLHKEIILETTTKKGNTALHIAALAGQDEVVRELVNYGANV  104

Query  104  NEKTTRGYTLLHCAAAWGRLETLKALVE  131
            N ++ +G+T L+ AA    LE +K L+E
Sbjct  105  NAQSQKGFTPLYMAAQENHLEVVKFLLE  132


 Score = 46.6 bits (109),  Expect = 0.003, Method: Compositional matrix adjust.
 Identities = 28/88 (31%), Positives = 44/88 (50%), Gaps = 0/88 (0%)

Query  81   LYAACMAGQSDVIRALAKYGVNLNEKTTRGYTLLHCAAAWGRLETLKALVELDVDIEALN  140
            L+ A   G +++++ L +   N N  TT G+T LH AA  G +ET+ AL+E +      +
Sbjct  474  LHCAARIGHTNMVKLLLENNANPNLATTAGHTPLHIAAREGHVETVLALLEKEASQACMT  533

Query  141  FREERARDVAARYSQTECVEFLDWADAR  168
             +       VAA+Y +     E L   DA
Sbjct  534  KKGFTPLHVAAKYGKVRVAELLLERDAH  561


 Score = 45.1 bits (105),  Expect = 0.007, Method: Compositional matrix adjust.
 Identities = 31/117 (26%), Positives = 52/117 (44%), Gaps = 7/117 (5%)

Query  63   HEQAMQLLLEED-------IVGRNLLYAACMAGQSDVIRALAKYGVNLNEKTTRGYTLLH  115
            H +  ++LL++         + G   L+ AC       V+  L K G +++  T  G T LH
Sbjct  350  HHRVAKVLLDKGAKPNSRALNGFTPLHIACKKNHVRVMELLLKTGASIDAVTESGLTPLH  409

Query  116  CAAAWGRLETLKALVELDVDIEALNFREERARDVAARYSQTECVEFLDWADARLTLK  172
             A+  G L  +K L++         N + E    +AAR   TE  ++L    A++  K
Sbjct  410  VASFMGHLPIVKNLLQRGASPNVSNVKVETPLHMAARAGHTEVAKYLLQNKAKVNAK  466


 Score = 42.0 bits (97),  Expect = 0.071, Method: Compositional matrix adjust.
 Identities = 25/86 (29%), Positives = 39/86 (45%), Gaps = 0/86 (0%)

Query  77   GRNLLYAACMAGQSDVIRALAKYGVNLNEKTTRGYTLLHCAAAWGRLETLKALVELDVDI  136
            G   L+      G    V   L K+GV ++   T   GYT LH A+ +G ++ +K L++    D+
Sbjct  668  GLTPLHLVAQEGHVPVADVLIKHGVMVDATTRMGYTPLHVASHYGNIKLVKFLLQHQADV  727

Query  137  EALNFREERARDVAARYSQTECVEFL  162
             A            AA+    T+ V  L
Sbjct  728  NAKTKLGYSPLHQAAQQGHTDIVTLL  753


 Score = 38.9 bits (89),  Expect = 0.60, Method: Compositional matrix adjust.
 Identities = 40/167 (23%), Positives = 63/167 (37%), Gaps = 7/167 (4%)

Query  63   HEQAMQLLLEE----DIV---GRNLLYAACMAGQSDVIRALAKYGVNLNEKTTRGYTLLH  115
            H + M+LLL+      D V   G   L+ A    G    +++ L + G + N     +  T LH
Sbjct  383  HVRVMELLLKTGASIDAVTESGLTPLHVASFMGHLPIVKNLLQRGASPNVSNVKVETPLH  442

Query  116  CAAAWGRLETLKALVELDVDIEALNFREERARDVAARYSQTECVEFLDWADARLTLKKYI  175
             AA  G  E  K L++     + A     ++       AAR    T  V+ L    +A    L
Sbjct  443  MAARAGHTEVAKYLLQNKAKVNAKAKDDQTPLHCAARIGHTNMVKLLLENNANPNLATTA  502

Query  176  AKVSLAVTDTEKGSGKLLKEDKNTILSACRAKNEWLETHTEASINEL  222
                L +    E      +L    +    AC  K  +    H  A   ++
Sbjct  503  GHTPLHIAAREGHVETVLALLEKEASQACMTKKGFTPLHVAAKYGKV  549


 Score = 36.6 bits (83),  Expect = 2.9, Method: Compositional matrix adjust.
 Identities = 27/107 (25%), Positives = 46/107 (42%), Gaps = 7/107 (6%)

Query  63   HEQAMQLLLEED-------IVGRNLLYAACMAGQSDVIRALAKYGVNLNEKTTRGYTLLH  115
            H    ++LLE +              G    L+ A    G    + + AL +    +       T +G+T LH
Sbjct  482  HTNMVKLLLENNANPNLATTAGHTPLHIAAREGHVETVLALLEKEASQACMTKKGFTPLH  541

Query  116  CAAAWGRLETLKALVELDVDIEALNFREERARDVAARYSQTECVEFL  162
             AA +G++    + L+E D     A         VA   ++   + V+ L
Sbjct  542  VAAKYGKVRVAELLLERDAHPNAAGKNGLTPLHVAVHHNNLDIVKLL  588


 Score = 35.0 bits (79),  Expect = 8.5, Method: Compositional matrix adjust.
 Identities = 19/54 (35%), Positives = 29/54 (53%), Gaps = 0/54 (0%)

Query  77   GRNLLYAACMAGQSDVIRALAKYGVNLNEKTTRGYTLLHCAAAWGRLETLKALV  130
            G    L+ A     Q +V R+L +YG + N ++ +G T LH AA   G   E +  L+
Sbjct  602  GYTPLHIAAKQNQVEVARSLLQYGGSANAESVQGVTPLHLAAQEGHAEMVALLL  655


>ref|NP_000028.3|  ankyrin 1 isoform 3 [Homo sapiens]
 gb|EAW63249.1|  ankyrin 1, erythrocytic, isoform CRA_i [Homo sapiens]
Length=1880

 GENE ID: 286 ANK1 | ankyrin 1, erythrocytic [Homo sapiens]
(Over 10 PubMed links)

 Score = 52.8 bits (125),  Expect = 3e-05, Method: Compositional matrix adjust.
 Identities = 30/88 (34%), Positives = 49/88 (55%), Gaps = 6/88 (6%)

Query  50   GLQKIFEDPENPHHEQAMQLLLEEDIV------GRNLLYAACMAGQSDVIRALAKYGVNL  103
            GL  +     +  H +  ++LL +E I+       G    L+ A +AGQ +V+R L  YG N+
Sbjct  45   GLNGLHLASKEGHVKMVVELLHKEIILETTTKKGNTALHIAALAGQDEVVRELVNYGANV  104

Query  104  NEKTTRGYTLLHCAAAWGRLETLKALVE  131
            N ++ +G+T L+ AA    LE +K L+E
Sbjct  105  NAQSQKGFTPLYMAAQENHLEVVKFLLE  132


 Score = 46.2 bits (108),  Expect = 0.003, Method: Compositional matrix adjust.
 Identities = 28/87 (32%), Positives = 44/87 (50%), Gaps = 0/87 (0%)

Query  81   LYAACMAGQSDVIRALAKYGVNLNEKTTRGYTLLHCAAAWGRLETLKALVELDVDIEALN  140
            L+ A   G +++++ L +   N N  TT G+T LH AA  G +ET+ AL+E +      +
Sbjct  474  LHCAARIGHTNMVKLLLENNANPNLATTAGHTPLHIAAREGHVETVLALLEKEASQACMT  533

Query  141  FREERARDVAARYSQTECVEFLDWADA  167
```

```
            +      VAA+Y +    E L   DA
Sbjct  534  KKGFTPLHVAAKYGKVRVAELLLERDA  560


 Score = 45.1 bits (105),  Expect = 0.008, Method: Compositional matrix adjust.
 Identities = 31/117 (26%), Positives = 52/117 (44%), Gaps = 7/117 (5%)

Query  63   HEQAMQLLLEED-------IVGRNLLYAACMAGQSDVIRALAKYGVNLNEKTTRGYTLLH  115
            H +  ++LL++        + G   L+ AC      V+  L K G +++  T  G T LH
Sbjct  350  HHRVAKVLLDKGAKPNSRALNGFTPLHIACKKNHVRVMELLLKTGASIDAVTESGLTPLH  409

Query  116  CAAAWGRLETLKALVELDVDIEALNFREERARDVAARYSQTECVEFLDWADARLTLK  172
             A+  G L  +K L++         N + E    +AAR   TE  ++L    A++  K
Sbjct  410  VASFMGHLPIVKNLLQRGASPNVSNVKVETPLHMAARAGHTEVAKYLLQNKAKVNAK  466


 Score = 41.6 bits (96),  Expect = 0.080, Method: Compositional matrix adjust.
 Identities = 25/86 (29%), Positives = 39/86 (45%), Gaps = 0/86 (0%)

Query  77   GRNLLYAACMAGQSDVIRALAKYGVNLNEKTTRGYTLLHCAAAWGRLETLKALVELDVDI  136
            G   L+     G   V    L K+GV ++  T   GYT LH A+ +G ++ +K L++   D+
Sbjct  668  GLTPLHLVAQEGHVPVADVLIKHGVMVDATTRMGYTPLHVASHYGNIKLVKFLLQHQADV  727

Query  137  EALNFREERARDVAARYSQTECVEFL  162
             A           AA+    T+ V  L
Sbjct  728  NAKTKLGYSPLHQAAQQGHTDIVTLL  753


 Score = 38.5 bits (88),  Expect = 0.65, Method: Compositional matrix adjust.
 Identities = 40/168 (23%), Positives = 64/168 (38%), Gaps = 7/168 (4%)

Query  62   HHEQAMQLLLEE----DIV---GRNLLYAACMAGQSDVIRALAKYGVNLNEKTTRGYTLL  114
            +H + M+LLL+      D V    G   L+ A    G    +++ L + G + N     +  T L
Sbjct  382  NHVRVMELLLKTGASIDAVTESGLTPLHVASFMGHLPIVKNLLQRGASPNVSNVKVETPL  441

Query  115  HCAAAWGRLETLKALVELDVDIEALNFREERARDVAARYSQTECVEFLDWADARLTLKKY  174
            H AA  G  E  K L++     + A     ++       AAR    T  V+ L    +A    L
Sbjct  442  HMAARAGHTEVAKYLLQNKAKVNAKAKDDQTPLHCAARIGHTNMVKLLLENNANPNLATT  501

Query  175  IAKVSLAVTDTEKGSGKLLKEDKNTILSACRAKNEWLETHTEASINEL  222
                 L +    E      +L    +      AC  K  +    H  A    ++
Sbjct  502  AGHTPLHIAAREGHVETVLALLEKEASQACMTKKGFTPLHVAAKYGKV  549


 Score = 36.2 bits (82),  Expect = 3.1, Method: Compositional matrix adjust.
 Identities = 27/107 (25%), Positives = 46/107 (42%), Gaps = 7/107 (6%)

Query  63   HEQAMQLLLEED-------IVGRNLLYAACMAGQSDVIRALAKYGVNLNEKTTRGYTLLH  115
            H    ++LLLE +          G   L+ A   G  + + AL +    +     T +G+T LH
Sbjct  482  HTNMVKLLLENNANPNLATTAGHTPLHIAAREGHVETVLALLEKEASQACMTKKGFTPLH  541

Query  116  CAAAWGRLETLKALVELDVDIEALNFREERARDVAARYSQTECVEFL  162
             AA +G++    + L+E D    A          VA  ++  + V+ L
Sbjct  542  VAAKYGKVRVAELLLERDAHPNAAGKNGLTPLHVAVHHNNLDIVKLL  588


 Score = 34.7 bits (78),  Expect = 9.1, Method: Compositional matrix adjust.
 Identities = 19/54 (35%), Positives = 29/54 (53%), Gaps = 0/54 (0%)

Query  77   GRNLLYAACMAGQSDVIRALAKYGVNLNEKTTRGYTLLHCAAAWGRLETLKALV  130
            G   L+ A    Q +V R+L +YG + N ++ +G T LH AA  G  E +  L+
Sbjct  602  GYTPLHIAAKQNQVEVARSLLQYGGSANAESVQGVTPLHLAAQEGHAEMVALLL  655
```

**>emb|CAA34611.1|** alt. ankyrin (variant 2.2) [Homo sapiens]
Length=1719

**GENE ID: 286 ANK1** | ankyrin 1, erythrocytic [Homo sapiens]
(Over 10 PubMed links)

```
 Score = 52.8 bits (125),  Expect = 3e-05, Method: Compositional matrix adjust.
 Identities = 30/88 (34%), Positives = 49/88 (55%), Gaps = 6/88 (6%)

Query  50   GLQKIFEDPENPHHEQAMQLLLEEDIV------GRNLLYAACMAGQSDVIRALAKYGVNL  103
            GL   +     +  H +   ++LL +E I+       G    L+ A +AGQ +V+R L  YG N+
Sbjct  45   GLNGLHLASKEGHVKMVVELLHKEIILETTTKKGNTALHIAALAGQDEVVRELVNYGANV  104

Query  104  NEKTTRGYTLLHCAAAWGRLETLKALVE  131
            N ++ +G+T L+ AA    LE +K L+E
Sbjct  105  NAQSQKGFTPLYMAAQENHLEVVKFLLE  132


 Score = 46.6 bits (109),  Expect = 0.002, Method: Compositional matrix adjust.
 Identities = 28/87 (32%), Positives = 44/87 (50%), Gaps = 0/87 (0%)

Query  81   LYAACMAGQSDVIRALAKYGVNLNEKTTRGYTLLHCAAAWGRLETLKALVELDVDIEALN  140
            L+ A    G +++++ L +   N N  TT G+T LH AA  G +ET+ AL+E +      +
Sbjct  474  LHCAARIGHTNMVKLLLENNANPNLATTAGHTPLHIAAREGHVETVLALLEKEASQACMT  533

Query  141  FREERARDVAARYSQTECVEFLDWADA  167
             +       VAA+Y +    E L   DA
Sbjct  534  KKGFTPLHVAAKYGKVRVAELLLERDA  560


 Score = 45.4 bits (106),  Expect = 0.006, Method: Compositional matrix adjust.
 Identities = 31/117 (26%), Positives = 52/117 (44%), Gaps = 7/117 (5%)

Query  63   HEQAMQLLLEED-------IVGRNLLYAACMAGQSDVIRALAKYGVNLNEKTTRGYTLLH  115
            H +  ++LL++        + G   L+ AC      V+  L K G +++  T  G T LH
Sbjct  350  HHRVAKVLLDKGAKPNSRALNGFTPLHIACKKNHVRVMELLLKTGASIDAVTESGLTPLH  409

Query  116  CAAAWGRLETLKALVELDVDIEALNFREERARDVAARYSQTECVEFLDWADARLTLK  172
             A+  G L  +K L++         N + E    +AAR   TE  ++L    A++  K
Sbjct  410  VASFMGHLPIVKNLLQRGASPNVSNVKVETPLHMAARAGHTEVAKYLLQNKAKVNAK  466


 Score = 41.6 bits (96),  Expect = 0.073, Method: Compositional matrix adjust.
 Identities = 25/86 (29%), Positives = 39/86 (45%), Gaps = 0/86 (0%)
```

```
Query  77   GRNLLYAACMAGQSDVIRALAKYGVNLNEKTTRGYTLLHCAAAWGRLETLKALVELDVDI  136
            G   L+     G   V   L K+GV ++   T  GYT LH A+ +G ++ +K L++   D+
Sbjct  668  GLTPLHLVAQEGHVPVADVLIKHGVMVDATTRMGYTPLHVASHYGNIKLVKFLLQHQADV  727

Query  137  EALNFREERARDVAARYSQTECVEFL  162
             A            AA+    T+ V  L
Sbjct  728  NAKTKLGYSPLHQAAQQGHTDIVTLL  753


 Score = 38.9 bits (89),  Expect = 0.51, Method: Compositional matrix adjust.
 Identities = 40/167 (23%), Positives = 63/167 (37%), Gaps = 7/167 (4%)

Query  63   HEQAMQLLLEE----DIV---GRNLLYAACMAGQSDVIRALAKYGVNLNEKTTRGYTLLH  115
            H + M+LLL+     D V   G   L+ A    G   +++ L + G + N    +   T LH
Sbjct  383  HVRVMELLLKTGASIDAVTESGLTPLHVASFMGHLPIVKNLLQRGASPNVSNVKVETPLH  442

Query  116  CAAAWGRLETLKALVELDVDIEALNFREERARDVAARYSQTECVEFLDWADARLTLKKYI  175
             AA  G  E  K L++     + A     ++      AAR    T  V+ L    +A    L
Sbjct  443  MAARAGHTEVAKYLLQNKAKVNAKAKDDQTPLHCAARIGHTNMVKLLLENNANPNLATTA  502

Query  176  AKVSLAVTDTEKGSGKLLKEDKNTILSACRAKNEWLETHTEASINEL  222
                L +    E      +L    +      AC  K  +    H  A   ++
Sbjct  503  GHTPLHIAAREGHVETVLALLEKEASQACMTKKGFTPLHVAAKYGKV  549


 Score = 36.6 bits (83),  Expect = 2.9, Method: Compositional matrix adjust.
 Identities = 27/107 (25%), Positives = 46/107 (42%), Gaps = 7/107 (6%)

Query  63   HEQAMQLLLEED-------IVGRNLLYAACMAGQSDVIRALAKYGVNLNEKTTRGYTLLH  115
            H    ++LLE  +           G   L+ A    G  + + AL +    +     T +G+T LH
Sbjct  482  HTNMVKLLLENNANPNLATTAGHTPLHIAAREGHVETVLALLEKEASQACMTKKGFTPLH  541

Query  116  CAAAWGRLETLKALVELDVDIEALNFREERARDVAARYSQTECVEFL  162
             AA +G++    + L+E D     A                 VA  ++    + V+ L
Sbjct  542  VAAKYGKVRVAELLLERDAHPNAAGKNGLTPLHVAVHHNNLDIVKLL  588


 Score = 34.7 bits (78),  Expect = 9.2, Method: Compositional matrix adjust.
 Identities = 19/54 (35%), Positives = 29/54 (53%), Gaps = 0/54 (0%)

Query  77   GRNLLYAACMAGQSDVIRALAKYGVNLNEKTTRGYTLLHCAAAWGRLETLKALV  130
            G   L+ A     Q +V R+L +YG + N ++ +G T LH AA   G   E +   L+
Sbjct  602  GYTPLHIAAKQNQVEVARSLLQYGGSANAESVQGVTPLHLAAQEGHAEMVALLL  655


>ref|XP_882829.2| PREDICTED: similar to apoptosis-stimulating protein of p53, 1
isoform 4 [Bos taurus]
Length=1081

 GENE ID: 511414 PPP1R13B | protein phosphatase 1, regulatory (inhibitor)
subunit 13B [Bos taurus]

 Score = 52.8 bits (125),  Expect = 3e-05, Method: Compositional matrix adjust.
 Identities = 34/103 (33%), Positives = 50/103 (48%), Gaps = 15/103 (14%)

Query  39   LLQPALTGDVEGLQKIF---EDPENPHHEQAMQLLLEEDIVGRNLLYAACMAGQSDVIRA  95
            LL  +L G+ + +Q++    EDP   P+ E             G   L+ A  AG    ++R
Sbjct  883  LLDASLEGEFDLVQRVIYEVEDPSKPNDE------------GITPLHNAVCAGHHHIVRF  930

Query  96   LAKYGVNLNEKTTRGYTLLHCAAAWGRLETLKALVELDVDIEA  138
            L  +GVN+N   + G+T LHCAA+   +   K LVE    I A
Sbjct  931  LLDFGVNVNAADSDGWTPLHCAASCNSVHLCKQLVESGAAIFA  973


>gb|AAA51732.1| ankyrin [Homo sapiens]
Length=1880

 GENE ID: 286 ANK1 | ankyrin 1, erythrocytic [Homo sapiens]
(Over 10 PubMed links)

 Score = 52.8 bits (125),  Expect = 3e-05, Method: Compositional matrix adjust.
 Identities = 30/88 (34%), Positives = 49/88 (55%), Gaps = 6/88 (6%)

Query  50   GLQKIFEDPENPHHEQAMQLLLEEDIV------GRNLLYAACMAGQSDVIRALAKYGVNL  103
            GL  +    +  H +  ++LL +E I+      G    L+ A +AGQ +V+R L  YG N+
Sbjct  45   GLNGLHLASKEGHVKMVVELLHKEIILETTTKKGNTALHIAALAGQDEVVRELVNYGANV  104

Query  104  NEKTTRGYTLLHCAAAWGRLETLKALVE  131
            N ++ +G+T L+ AA    LE +K L+E
Sbjct  105  NAQSQKGFTPLYMAAQENHLEVVKFLLE  132


 Score = 46.6 bits (109),  Expect = 0.003, Method: Compositional matrix adjust.
 Identities = 28/87 (32%), Positives = 44/87 (50%), Gaps = 0/87 (0%)

Query  81   LYAACMAGQSDVIRALAKYGVNLNEKTTRGYTLLHCAAAWGRLETLKALVELDVDIEALN  140
            L+ A   G +++++ L +   N N  TT G+T LH AA  G +ET+ AL+E +     +
Sbjct  474  LHCAARIGHTNMVKLLLENNANPNLATTAGHTPLHIAAREGHVETVLALLEKEASQACMT  533

Query  141  FREERARDVAARYSQTECVEFLDWADA  167
             +       VAA+Y +    E L   DA
Sbjct  534  KKGFTPLHVAAKYGKVRVAELLLERDA  560


 Score = 45.1 bits (105),  Expect = 0.008, Method: Compositional matrix adjust.
 Identities = 31/117 (26%), Positives = 52/117 (44%), Gaps = 7/117 (5%)

Query  63   HEQAMQLLLEED-------IVGRNLLYAACMAGQSDVIRALAKYGVNLNEKTTRGYTLLH  115
            H +  ++LL++        + G   L+ AC       V+ +  L K G +++  T  G T LH
Sbjct  350  HHRVAKVLLDKGAKPNSRALNGFTPLHIACKKNHVRVMELLLKTGASIDAVTESGLTPLH  409

Query  116  CAAAWGRLETLKALVELDVDIEALNFREERARDVAARYSQTECVEFLDWADARLTLK  172
             A+  G L  +K L++         N + E    +AAR   TE  ++L     A++  K
Sbjct  410  VASFMGHLPIVKNLLQRGASPNVSNVKVETPLHMAARAGHTEVAKYLLQNKAKVNAK  466
```

```
 Score = 41.6 bits (96),  Expect = 0.077, Method: Compositional matrix adjust.
 Identities = 25/86 (29%), Positives = 39/86 (45%), Gaps = 0/86 (0%)

Query  77   GRNLLYAACMAGQSDVIRALAKYGVNLNEKTTRGYTLLHCAAAWGRLETLKALVELDVDI  136
            G   L+     G    V   L K+GV ++  T  GYT LH A+ +G ++ +K L++   D+
Sbjct  668  GLTPLHLVAQEGHVPVADVLIKHGVMVDATTRMGYTPLHVASHYGNIKLVKFLLQHQADV  727

Query  137  EALNFREERARDVAARYSQTECVEFL  162
             A           AA+   T+ V  L
Sbjct  728  NAKTKLGYSPLHQAAQQGHTDIVTLL  753


 Score = 38.5 bits (88),  Expect = 0.69, Method: Compositional matrix adjust.
 Identities = 40/168 (23%), Positives = 64/168 (38%), Gaps = 7/168 (4%)

Query  62   HHEQAMQLLLEE----DIV---GRNLLYAACMAGQSDVIRALAKYGVNLNEKTTRGYTLL  114
            +H + M+LLL+     D V   G   L+ A   G   +++ L + G + N    +  T L
Sbjct  382  NHVRVMELLLKTGASIDAVTESGLTPLHVASFMGHLPIVKNLLQRGASPNVSNVKVETPL  441

Query  115  HCAAAWGRLETLKALVELDVDIEALNFREERARDVAARYSQTECVEFLDWADARLTLKKY  174
            H AA  G  E  K L++    + A     ++     AAR   T  V+ L   +A   L
Sbjct  442  HMAARAGHTEVAKYLLQNKAKVNAKAKDDQTPLHCAARIGHTNMVKLLLENNANPNLATT  501

Query  175  IAKVSLAVTDTEKGSGKLLKEDKNTILSACRAKNEWLETHTEASINEL  222
                 L +   E     +L   +     AC  K  +   H  A   ++
Sbjct  502  AGHTPLHIAAREGHVETVLALLEKEASQACMTKKGFTPLHVAAKYGKV  549


 Score = 36.2 bits (82),  Expect = 3.1, Method: Compositional matrix adjust.
 Identities = 27/107 (25%), Positives = 46/107 (42%), Gaps = 7/107 (6%)

Query  63   HEQAMQLLLEED-------IVGRNLLYAACMAGQSDVIRALAKYGVNLNEKTTRGYTLLH  115
            H   ++LLLE +         G   L+ A   G  + + AL +   +    T +G+T LH
Sbjct  482  HTNMVKLLLENNANPNLATTAGHTPLHIAAREGHVETVLALLEKEASQACMTKKGFTPLH  541

Query  116  CAAAWGRLETLKALVELDVDIEALNFREERARDVAARYSQTECVEFL  162
             AA +G++   + L+E D    A          VA  ++  + V+ L
Sbjct  542  VAAKYGKVRVAELLLERDAHPNAAGKNGLTPLHVAVHHNNLDIVKLL  588


 Score = 34.7 bits (78),  Expect = 9.2, Method: Compositional matrix adjust.
 Identities = 19/54 (35%), Positives = 29/54 (53%), Gaps = 0/54 (0%)

Query  77   GRNLLYAACMAGQSDVIRALAKYGVNLNEKTTRGYTLLHCAAAWGRLETLKALV  130
            G   L+ A    Q +V R+L +YG + N ++ +G T LH AA  G  E +  L+
Sbjct  602  GYTPLHIAAKQNQVEVARSLLQYGGSANAESVQGVTPLHLAAQEGHAEMVALLL  655


>gb|AAI57919.1|  Ankrd44 protein [Mus musculus]
Length=993

 GENE ID: 329154 Ankrd44 | ankyrin repeat domain 44 [Mus musculus]
(10 or fewer PubMed links)

 Score = 52.8 bits (125),  Expect = 3e-05, Method: Compositional matrix adjust.
 Identities = 40/135 (29%), Positives = 58/135 (42%), Gaps = 7/135 (5%)

Query  38   PLLQPALTGDVEGLQKIFEDPENPHHEQAMQLLLEE------DIVGRNLLYAACMAGQSD  91
            PLL      D  G    +    N H E    LL +       D   R  L+ A   G  D
Sbjct  128  PLLSSVNVSDRGGRTALHHAALNGHMEMVNLLLAKGANINAFDKKDRRALHWAAYMGHLD  187

Query  92   VIRALAKYGVNLNEKTTRGYTLLHCAAAWGRLETLKALVELDVDIEALNFREERARDVAA  151
            V+  L  +G  +  K  +GYT LH AA+ G++  +K L+ L V+I+ +N     A  +A
Sbjct  188  VVALLINHGAEVTCKDKKGYTPLHAAASNGQISVVKHLLNLGVEIDEINVYGNTALHIAC  247

Query  152  RYSQTECV-EFLDWA  165
               Q   V E +D+
Sbjct  248  YNGQDAVVNELIDYG  262


 Score = 43.1 bits (100),  Expect = 0.028, Method: Compositional matrix adjust.
 Identities = 33/104 (31%), Positives = 51/104 (49%), Gaps = 16/104 (15%)

Query  72   EEDIVGRNLLYAACMAGQSDVIRALAKYGVNLNEKTTRGYTLLHCAAA--WGRLETLKAL  129
            E ++ G   L+ AC  GQ  V+  L  YG N+N+    G+T LH AAA   G L  L+ L
Sbjct  234  EINVYGNTALHIACYNGQDAVVNELIDYGANVNQPNNSGFTPLHFAAASTHGAL-CLELL  292

Query  130  VE--LDVDIE-----------ALNFREERARDVAARYSQTECVE  160
            V    DV+I+           A++ R  R++ +     + +CV+
Sbjct  293  VNNGADVNIQSKDGKSPLHMTAVHGRFTRSQTLIQNGGEIDCVD  336


 Score = 38.9 bits (89),  Expect = 0.52, Method: Compositional matrix adjust.
 Identities = 22/63 (34%), Positives = 31/63 (49%), Gaps = 0/63 (0%)

Query  74   DIVGRNLLYAACMAGQSDVIRALAKYGVNLNEKTTRGYTLLHCAAAWGRLETLKALVELD  133
            D  GR  L+AA   G  + I+ L   G + ++K   G T LH AAA      +KALV
Sbjct  402  DTFGRTCLHAAAAGGNVECIKLLQSSGADFHKKDKCGRTPLHYAAANCHFHCIKALVTTG  461

Query  134  VDI  136
             ++
Sbjct  462  ANV  464


 Score = 36.6 bits (83),  Expect = 2.6, Method: Compositional matrix adjust.
 Identities = 33/124 (26%), Positives = 51/124 (41%), Gaps = 27/124 (21%)

Query  36   KNPLLQPALTGDVEGLQKIFEDPENPH--------------------HEQAMQLLLEE--  73
            + PL    + G    L+ + E  +NP                     H  A+ LLLE+
Sbjct  635  RTPLHASVINGHTLCLRLLLETADNPEVVDVKDAKGQTPLMLAVAYGHIDAVSLLLEKEA  694

Query  74   -----DIVGRNLLYAACMAGQSDVIRALAKYGVNLNEKTTRGYTLLHCAAAWGRLETLKA  128
                 DIVG   L+   M G  + ++ L +   ++  K +RG T LH AAA G    L
Sbjct  695  NVDAVDIVGCTALHRGIMTGHEECVQMLLEQEASILCKDSRGRTPLHYAAARGHATWLNE  754

Query  129  LVEL  132
            L+++
Sbjct  755  LLQI  758
```

```
 Score = 35.4 bits (80),  Expect = 6.3, Method: Compositional matrix adjust.
 Identities = 22/78 (28%), Positives = 33/78 (42%), Gaps = 4/78 (5%)

Query  81   LYAACMAGQSDVIRALAKYGVNLNEKTTRGYTLLHCAAAWGRLETLKALVELDVDIEALN  140
            L+ A +   SD  R L   G ++    T G T LH AAA G +E +K L     D
Sbjct  376  LHLAALNAHSDCCRKLLSSGFEIDTPDTFGRTCLHAAAAGGNVECIKLLQSSGADFH---  432

Query  141  FREERARDVAARYSQTEC  158
             ++++       Y+   C
Sbjct  433  -KKDKCGRTPLHYAAANC  449


 Score = 35.0 bits (79),  Expect = 7.4, Method: Compositional matrix adjust.
 Identities = 32/125 (25%), Positives = 53/125 (42%), Gaps = 9/125 (7%)

Query  38   PLLQPALTGDVEGLQKIFEDPENPHHEQAMQLLLEEDIVGRNLLYAACMAGQSDVIRALA  97
            PL+Q   +GD E ++ +    E+      +  L  E    R  L+ A   G +++I  L
Sbjct  11   PLVQAIFSGDPEEIRMLIHKTED------VNALDSEK---RTPLHVAAFLGDAEIIELLI  61

Query  98   KYGVNLNEKTTRGYTLLHCAAAWGRLETLKALVELDVDIEALNFREERARDVAARYSQTE  157
              G  +N K     T LH A A    E ++ L++   D+ A +   +    VAA     +
Sbjct  62   LLGARVNAKDNMWLTPLHRAVASRSEEAVQVLIKHSADVNARDKNWQTPLHVAAANKAVK  121

Query  158  CVEFL  162
            C E +
Sbjct  122  CAEVI  126
```

>**ref|XP_001628783.1|** predicted protein [Nematostella vectensis]
**gb|EDO36720.1|** predicted protein [Nematostella vectensis]
Length=262

**GENE ID: 5508182 NEMVEDRAFT_v1g170793** | hypothetical protein
[Nematostella vectensis] (10 or fewer PubMed links)

```
 Score = 52.8 bits (125),  Expect = 3e-05, Method: Compositional matrix adjust.
 Identities = 37/108 (34%), Positives = 51/108 (47%), Gaps = 5/108 (4%)

Query  70   LLEEDIVGRNLLYAACMAGQSDVIRALAKYGVNLNEKTTRGYTLLHCAAAWGRLETLKAL  129
            LL+ED  G   L  AC  G   +++ L  YG NL+ +   G+++LH AA  G L  L+ L
Sbjct  41   LLDED--GLTALQRACFTGNLKLVQLLVSYGANLDIQDKEGWSVLHAAAVAGNLSILRYL  98

Query  130  VELDVDIEALNFREERARDVAARYSQTECVEFLDWADARLTLKKYIAK  177
            V +  D+   N   E   DVA   +   CV  L     R  L+K   K
Sbjct  99   VAVGADVSVRNDLGELPIDVA---TDVHCVIVLAEGMKRAGLRKLTDK  143
```

>**ref|XP_001328459.1|** hypothetical protein [Trichomonas vaginalis G3]
**gb|EAY16236.1|** hypothetical protein TVAG_341300 [Trichomonas vaginalis G3]
Length=446

**GENE ID: 4774244 TVAG_341300** | hypothetical protein [Trichomonas vaginalis G3]
(10 or fewer PubMed links)

```
 Score = 52.8 bits (125),  Expect = 3e-05, Method: Compositional matrix adjust.
 Identities = 36/119 (30%), Positives = 50/119 (42%), Gaps = 6/119 (5%)

Query  50   GLQKIFEDPENPHHEQAMQLLL------EEDIVGRNLLYAACMAGQSDVIRALAKYGVNL  103
            G   +FE     H E    L+        +D   R LL+ A   G  D+++ L   G ++
Sbjct  190  GFTPLFEAARQSHFEIVKYLISVGANIETKDSSKRTLLHNASYGGNLDIVKYLVSIGADI  249

Query  104  NEKTTRGYTLLHCAAAWGRLETLKALVELDVDIEALNFREERARDVAARYSQTECVEFL  162
            N K   G T    AA WG+L+ LK L+    D E  N   E     A  Y + E V +L
Sbjct  250  NAKDMDGETATSVAAMWGQLDVLKFLISAGADKEEKNNNGETTLWKAFEYEKYEIVIYL  308


 Score = 52.4 bits (124),  Expect = 5e-05, Method: Compositional matrix adjust.
 Identities = 30/92 (32%), Positives = 47/92 (51%), Gaps = 0/92 (0%)

Query  76   VGRNLLYAACMAGQSDVIRALAKYGVNLNEKTTRGYTLLHCAAAWGRLETLKALVELDVD  135
            +G   L+ A      ++++ L   G N+  K +   TLLH A+  G L+ +K LV +  D
Sbjct  189  LGFTPLFEAARQSHFEIVKYLISVGANIETKDSSKRTLLHNASYGGNLDIVKYLVSIGAD  248

Query  136  IEALNFREERARDVAARYSQTECVEFLDWADA  167
            I A +   E A  VAA + Q + ++FL  A A
Sbjct  249  INAKDMDGETATSVAAMWGQLDVLKFLISAGA  280


 Score = 35.4 bits (80),  Expect = 5.2, Method: Compositional matrix adjust.
 Identities = 19/52 (36%), Positives = 28/52 (53%), Gaps = 0/52 (0%)

Query  87   AGQSDVIRALAKYGVNLNEKTTRGYTLLHCAAAWGRLETLKALVELDVDIEA  138
            +G +  +  L   G +   K   G TLL CA+  G+LET+K L+ +  D EA
Sbjct  332  SGSTKTLEYLISNGTDKETKDDHGRTLLICASEKGQLETVKYLISIGADKEA  383
```

>**ref|XP_001139450.1|** PREDICTED: ankyrin 1 isoform 3 [Pan troglodytes]
Length=1719

**GENE ID: 736634 ANK1** | ankyrin 1, erythrocytic [Pan troglodytes]

```
 Score = 52.8 bits (125),  Expect = 3e-05, Method: Compositional matrix adjust.
 Identities = 30/88 (34%), Positives = 49/88 (55%), Gaps = 6/88 (6%)

Query  50   GLQKIFEDPENPHHEQAMQLLLEEDIV------GRNLLYAACMAGQSDVIRALAKYGVNL  103
            GL  +    +  H +  ++LL +E I+      G   L+ A +AGQ +V+R L  YG N+
Sbjct  45   GLNGLHLASKEGHVKMVVELLHKEIILETTTKKGNTALHIAALAGQDEVVRELVNYGANV  104

Query  104  NEKTTRGYTLLHCAAAWGRLETLKALVE  131
            N ++ +G+T L+ AA    LE +K L+E
Sbjct  105  NAQSQKGFTPLYMAAQENHLEVVKFLLE  132


 Score = 46.6 bits (109),  Expect = 0.002, Method: Compositional matrix adjust.
```

```
 Identities = 28/87 (32%), Positives = 44/87 (50%), Gaps = 0/87 (0%)

Query  81   LYAACMAGQSDVIRALAKYGVNLNEKTTRGYTLLHCAAAWGRLETLKALVELDVDIEALN  140
            L+ A   G +++++ L +   N N  TT G+T LH AA  G +ET+ AL+E +     +
Sbjct  474  LHCAARIGHTNMVKLLLENNANPNLATTAGHTPLHIAAREGHVETVLALLEKEASQACMT  533

Query  141  FREERARDVAARYSQTECVEFLDWADA  167
             +      VAA+Y +    E L   DA
Sbjct  534  KKGFTPLHVAAKYGKVRVAELLLERDA  560


 Score = 45.4 bits (106),  Expect = 0.006, Method: Compositional matrix adjust.
 Identities = 31/117 (26%), Positives = 52/117 (44%), Gaps = 7/117 (5%)

Query  63   HEQAMQLLLEED-------IVGRNLLYAACMAGQSDVIRALAKYGVNLNEKTTRGYTLLH  115
            H +  ++LL++        + G   L+ AC      V+  L K G +++   T  G T LH
Sbjct  350  HHRVAKVLLDKGAKPNSRALNGFTPLHIACKKNHVRVMELLLKTGASIDAVTESGLTPLH  409

Query  116  CAAAWGRLETLKALVELDVDIEALNFREERARDVAARYSQTECVEFLDWADARLTLK  172
             A+  G L  +K L++        N + E    +AAR   TE  ++L    A++  K
Sbjct  410  VASFMGHLPIVKNLLQRGASPNVSNVKVETPLHMAARAGHTEVAKYLLQNKAKVNAK  466


 Score = 42.0 bits (97),  Expect = 0.072, Method: Compositional matrix adjust.
 Identities = 25/86 (29%), Positives = 39/86 (45%), Gaps = 0/86 (0%)

Query  77   GRNLLYAACMAGQSDVIRALAKYGVNLNEKTTRGYTLLHCAAAWGRLETLKALVELDVDI  136
            G   L+     G   V   L K+GV ++  T  GYT LH A+ +G ++ +K L++   D+
Sbjct  668  GLTPLHLVAQEGHVPVADVLIKHGVMVDATTRMGYTPLHVASHYGNIKLVKFLLQHQADV  727

Query  137  EALNFREERARDVAARYSQTECVEFL  162
             A           AA+   T+ V  L
Sbjct  728  NAKTKLGYSPLHQAAQQGHTDIVTLL  753


 Score = 38.9 bits (89),  Expect = 0.51, Method: Compositional matrix adjust.
 Identities = 40/167 (23%), Positives = 63/167 (37%), Gaps = 7/167 (4%)

Query  63   HEQAMQLLLEE----DIV---GRNLLYAACMAGQSDVIRALAKYGVNLNEKTTRGYTLLH  115
            H + M+LLL+     D V   G   L+ A   G   +++ L + G + N    +  T LH
Sbjct  383  HVRVMELLLKTGASIDAVTESGLTPLHVASFMGHLPIVKNLLQRGASPNVSNVKVETPLH  442

Query  116  CAAAWGRLETLKALVELDVDIEALNFREERARDVAARYSQTECVEFLDWADARLTLKKYI  175
             AA  G  E  K L++    + A    ++     AAR   T  V+ L   +A   L
Sbjct  443  MAARAGHTEVAKYLLQNKAKVNAKAKDDQTPLHCAARIGHTNMVKLLLENNANPNLATTA  502

Query  176  AKVSLAVTDTEKGSGKLLKEDKNTILSACRAKNEWLETHTEASINEL  222
                L +   E     +L    +    AC  K  +   H  A   ++
Sbjct  503  GHTPLHIAAREGHVETVLALLEKEASQACMTKKGFTPLHVAAKYGKV  549


 Score = 36.6 bits (83),  Expect = 2.8, Method: Compositional matrix adjust.
 Identities = 27/107 (25%), Positives = 46/107 (42%), Gaps = 7/107 (6%)

Query  63   HEQAMQLLLEED-------IVGRNLLYAACMAGQSDVIRALAKYGVNLNEKTTRGYTLLH  115
            H   ++LLLE +         G   L+ A   G  + + AL +   +    T +G+T LH
Sbjct  482  HTNMVKLLLENNANPNLATTAGHTPLHIAAREGHVETVLALLEKEASQACMTKKGFTPLH  541

Query  116  CAAAWGRLETLKALVELDVDIEALNFREERARDVAARYSQTECVEFL  162
             AA +G++   + L+E D    A          VA  ++  + V+ L
Sbjct  542  VAAKYGKVRVAELLLERDAHPNAAGKNGLTPLHVAVHHNNLDIVKLL  588


 Score = 34.7 bits (78),  Expect = 9.4, Method: Compositional matrix adjust.
 Identities = 19/54 (35%), Positives = 29/54 (53%), Gaps = 0/54 (0%)

Query  77   GRNLLYAACMAGQSDVIRALAKYGVNLNEKTTRGYTLLHCAAAWGRLETLKALV  130
            G   L+ A    Q +V R+L +YG + N ++ +G T LH AA  G  E +  L+
Sbjct  602  GYTPLHIAAKQNQVEVARSLLQYGGSANAESVQGVTPLHLAAQEGHAEMVALLL  655


>ref|NP_001104253.1| ankyrin 1, erythroid isoform 1 [Mus musculus]
 dbj|BAE27815.1| unnamed protein product [Mus musculus]
Length=1907

 GENE ID: 11733 Ank1 | ankyrin 1, erythroid [Mus musculus]
(Over 10 PubMed links)

 Score = 52.8 bits (125),  Expect = 3e-05, Method: Compositional matrix adjust.
 Identities = 30/88 (34%), Positives = 49/88 (55%), Gaps = 6/88 (6%)

Query  50   GLQKIFEDPENPHHEQAMQLLLEEDIV------GRNLLYAACMAGQSDVIRALAKYGVNL  103
            GL  +     +  H +  ++LL +E I+      G   L+ A +AGQ +V+R L  YG N+
Sbjct  78   GLNGLHLASKEGHVKMVVELLHKEIILETTTKKGNTALHIAALAGQDEVVRELVNYGANV  137

Query  104  NEKTTRGYTLLHCAAAWGRLETLKALVE  131
            N ++ +G+T L+ AA    LE +K L+E
Sbjct  138  NAQSQKGFTPLYMAAQENHLEVVKFLLE  165


 Score = 44.3 bits (103),  Expect = 0.013, Method: Compositional matrix adjust.
 Identities = 29/107 (27%), Positives = 48/107 (44%), Gaps = 7/107 (6%)

Query  63   HEQAMQLLLEED-------IVGRNLLYAACMAGQSDVIRALAKYGVNLNEKTTRGYTLLH  115
            H +  ++LL++        + G   L+ AC      V+  L K G +++   T  G T LH
Sbjct  383  HHRVAKVLLDKGAKPNSRALNGFTPLHIACKKNHIRVMELLLKTGASIDAVTESGLTPLH  442

Query  116  CAAAWGRLETLKALVELDVDIEALNFREERARDVAARYSQTECVEFL  162
             A+  G L  +K L++        N + E    +AAR   TE  ++L
Sbjct  443  VASFMGHLPIVKNLLQRGASPNVSNVKVETPLHMAARAGHTEVAKYL  489


 Score = 43.5 bits (101),  Expect = 0.019, Method: Compositional matrix adjust.
 Identities = 27/87 (31%), Positives = 43/87 (49%), Gaps = 0/87 (0%)

Query  81   LYAACMAGQSDVIRALAKYGVNLNEKTTRGYTLLHCAAAWGRLETLKALVELDVDIEALN  140
            L+ A   G + +++ L + G + N  TT G+T LH AA  G ++T  AL+E +     +
```

```
Sbjct  507  LHCAARIGHTGMVKLLLENGASPNLATTAGHTPLHTAAREGHVDTALALLEKEASQACMT  566

Query  141  FREERARDVAARYSQTECVEFLDWADA  167
             +      VAA+Y +    E L   DA
Sbjct  567  KKGFTPLHVAAKYGKVRLAELLLEHDA  593


 Score = 42.4 bits (98),  Expect = 0.051, Method: Compositional matrix adjust.
 Identities = 25/86 (29%), Positives = 39/86 (45%), Gaps = 0/86 (0%)

Query  77   GRNLLYAACMAGQSDVIRALAKYGVNLNEKTTRGYTLLHCAAAWGRLETLKALVELDVDI  136
            G   L+     G   V   L K+GV ++  T  GYT LH A+ +G ++ +K L++   D+
Sbjct  701  GLTPLHLVSQEGHVPVADVLIKHGVTVDATTRMGYTPLHVASHYGNIKLVKFLLQHQADV  760

Query  137  EALNFREERARDVAARYSQTECVEFL  162
             A           AA+   T+ V  L
Sbjct  761  NAKTKLGYSPLHQAAQQGHTDIVTLL  786


 Score = 35.0 bits (79),  Expect = 7.5, Method: Compositional matrix adjust.
 Identities = 28/102 (27%), Positives = 46/102 (45%), Gaps = 7/102 (6%)

Query  68   QLLLEEDI----VGRN---LLYAACMAGQSDVIRALAKYGVNLNEKTTRGYTLLHCAAAW  120
            +LLLE D      G+N    L+ A      D+++ L   G + +      GYT LH AA
Sbjct  586  ELLLEHDAHPNAAGKNGLTPLHVAVHHNNLDIVKLLLPRGGSPHSPAWNGYTPLHIAAKQ  645

Query  121  GRLETLKALVELDVDIEALNFREERARDVAARYSQTECVEFL  162
             ++E  ++L++      A + +       +AA+   TE V  L
Sbjct  646  NQIEVARSLLQYGGSANAESVQGVTPLHLAAQEGHTEMVALL  687


 Score = 35.0 bits (79),  Expect = 8.6, Method: Compositional matrix adjust.
 Identities = 28/107 (26%), Positives = 44/107 (41%), Gaps = 7/107 (6%)

Query  63   HEQAMQLLLEED-------IVGRNLLYAACMAGQSDVIRALAKYGVNLNEKTTRGYTLLH  115
            H   ++LLLE            G   L+ A   G  D   AL +    +    T +G+T LH
Sbjct  515  HTGMVKLLLENGASPNLATTAGHTPLHTAAREGHVDTALALLEKEASQACMTKKGFTPLH  574

Query  116  CAAAWGRLETLKALVELDVDIEALNFREERARDVAARYSQTECVEFL  162
             AA +G++    + L+E D    A          VA  ++  + V+ L
Sbjct  575  VAAKYGKVRLAELLLEHDAHPNAAGKNGLTPLHVAVHHNNLDIVKLL  621


 Score = 34.7 bits (78),  Expect = 9.9, Method: Compositional matrix adjust.
 Identities = 19/54 (35%), Positives = 29/54 (53%), Gaps = 0/54 (0%)

Query  77   GRNLLYAACMAGQSDVIRALAKYGVNLNEKTTRGYTLLHCAAAWGRLETLKALV  130
            G   L+ A    Q +V R+L +YG + N ++ +G T LH AA  G  E +  L+
Sbjct  635  GYTPLHIAAKQNQIEVARSLLQYGGSANAESVQGVTPLHLAAQEGHTEMVALLL  688
```

>**emb|CAA34610.1|** unnamed protein product [Homo sapiens]
Length=1881

**GENE ID: 286 ANK1** | ankyrin 1, erythrocytic [Homo sapiens]
(Over 10 PubMed links)

```
 Score = 52.8 bits (125),  Expect = 3e-05, Method: Compositional matrix adjust.
 Identities = 30/88 (34%), Positives = 49/88 (55%), Gaps = 6/88 (6%)

Query  50   GLQKIFEDPENPHHEQAMQLLLEEDIV------GRNLLYAACMAGQSDVIRALAKYGVNL  103
            GL  +    +  H +   ++LL +E I+       G   L+ A +AGQ +V+R L  YG N+
Sbjct  45   GLNGLHLASKEGHVKMVVELLHKEIILETTTKKGNTALHIAALAGQDEVVRELVNYGANV  104

Query  104  NEKTTRGYTLLHCAAAWGRLETLKALVE  131
            N ++ +G+T L+ AA    LE +K L+E
Sbjct  105  NAQSQKGFTPLYMAAQENHLEVVKFLLE  132


 Score = 46.6 bits (109),  Expect = 0.003, Method: Compositional matrix adjust.
 Identities = 28/88 (31%), Positives = 44/88 (50%), Gaps = 0/88 (0%)

Query  81   LYAACMAGQSDVIRALAKYGVNLNEKTTRGYTLLHCAAAWGRLETLKALVELDVDIEALN  140
            L+ A    G +++++ L +   N N  TT G+T LH AA  G +ET+ AL+E +     +
Sbjct  474  LHCAARIGHTNMVKLLLENNANPNLATTAGHTPLHIAAREGHVETVLALLEKEASQACMT  533

Query  141  FREERARDVAARYSQTECVEFLDWADAR  168
             +      VAA+Y +    E L   DA
Sbjct  534  KKGFTPLHVAAKYGKVRVAELLLERDAH  561


 Score = 45.1 bits (105),  Expect = 0.007, Method: Compositional matrix adjust.
 Identities = 31/117 (26%), Positives = 52/117 (44%), Gaps = 7/117 (5%)

Query  63   HEQAMQLLLEED-------IVGRNLLYAACMAGQSDVIRALAKYGVNLNEKTTRGYTLLH  115
            H +   ++LL++         + G   L+ AC      V+   L K G +++   T  G T LH
Sbjct  350  HHRVAKVLLDKGAKPNSRALNGFTPLHIACKKNHVRVMELLLKTGASIDAVTESGLTPLH  409

Query  116  CAAAWGRLETLKALVELDVDIEALNFREERARDVAARYSQTECVEFLDWADARLTLK  172
             A+  G L  +K L++         N + E    +AAR   TE  ++L    A++  K
Sbjct  410  VASFMGHLPIVKNLLQRGASPNVSNVKVETPLHMAARAGHTEVAKYLLQNKAKVNAK  466


 Score = 41.6 bits (96),  Expect = 0.074, Method: Compositional matrix adjust.
 Identities = 25/86 (29%), Positives = 39/86 (45%), Gaps = 0/86 (0%)

Query  77   GRNLLYAACMAGQSDVIRALAKYGVNLNEKTTRGYTLLHCAAAWGRLETLKALVELDVDI  136
            G   L+     G   V   L K+GV ++  T  GYT LH A+ +G ++ +K L++   D+
Sbjct  668  GLTPLHLVAQEGHVPVADVLIKHGVMVDATTRMGYTPLHVASHYGNIKLVKFLLQHQADV  727

Query  137  EALNFREERARDVAARYSQTECVEFL  162
             A           AA+   T+ V  L
Sbjct  728  NAKTKLGYSPLHQAAQQGHTDIVTLL  753


 Score = 38.5 bits (88),  Expect = 0.63, Method: Compositional matrix adjust.
 Identities = 40/167 (23%), Positives = 63/167 (37%), Gaps = 7/167 (4%)
```

```
Query  63   HEQAMQLLLEE----DIV---GRNLLYAACMAGQSDVIRALAKYGVNLNEKTTRGYTLLH  115
            H + M+LLL+     D V   G   L+ A   G   +++ L + G + N    +  T LH
Sbjct  383  HVRVMELLLKTGASIDAVTESGLTPLHVASFMGHLPIVKNLLQRGASPNVSNVKVETPLH  442

Query  116  CAAAWGRLETLKALVELDVDIEALNFREERARDVAARYSQTECVEFLDWADARLTLKKYI  175
             AA  G  E  K L++    + A    ++      AAR    T  V+ L    +A   L
Sbjct  443  MAARAGHTEVAKYLLQNKAKVNAKAKDDQTPLHCAARIGHTNMVKLLLENNANPNLATTA  502

Query  176  AKVSLAVTDTEKGSGKLLKEDKNTILSACRAKNEWLETHTEASINEL  222
                L +   E      +L    +      AC  K  +   H  A   ++
Sbjct  503  GHTPLHIAAREGHVETVLALLEKEASQACMTKKGFTPLHVAAKYGKV  549
```

Score = 36.6 bits (83),  Expect = 3.0, Method: Compositional matrix adjust.
Identities = 27/107 (25%), Positives = 46/107 (42%), Gaps = 7/107 (6%)

```
Query  63   HEQAMQLLLEED-------IVGRNLLYAACMAGQSDVIRALAKYGVNLNEKTTRGYTLLH  115
            H   ++LLE +          G   L+ A   G   + + AL +    +     T +G+T LH
Sbjct  482  HTNMVKLLLENNANPNLATTAGHTPLHIAAREGHVETVLALLEKEASQACMTKKGFTPLH  541

Query  116  CAAAWGRLETLKALVELDVDIEALNFREERARDVAARYSQTECVEFL  162
             AA +G++    + L+E D    A           VA  ++  + V+ L
Sbjct  542  VAAKYGKVRVAELLLERDAHPNAAGKNGLTPLHVAVHHNNLDIVKLL  588
```

Score = 35.0 bits (79),  Expect = 8.6, Method: Compositional matrix adjust.
Identities = 19/54 (35%), Positives = 29/54 (53%), Gaps = 0/54 (0%)

```
Query  77   GRNLLYAACMAGQSDVIRALAKYGVNLNEKTTRGYTLLHCAAAWGRLETLKALV  130
            G   L+ A    Q +V R+L +YG + N ++ +G T LH AA  G  E +  L+
Sbjct  602  GYTPLHIAAKQNQVEVARSLLQYGGSANAESVQGVTPLHLAAQEGHAEMVALLL  655
```

>**gb|AAH79910.1|** Ank1 protein [Mus musculus]
Length=1887

**GENE ID: 11733 Ank1** | ankyrin 1, erythroid [Mus musculus]
(Over 10 PubMed links)

Score = 52.8 bits (125),  Expect = 3e-05, Method: Compositional matrix adjust.
Identities = 30/88 (34%), Positives = 49/88 (55%), Gaps = 6/88 (6%)

```
Query  50   GLQKIFEDPENPHHEQAMQLLLEEDIV------GRNLLYAACMAGQSDVIRALAKYGVNL  103
            GL   +    +  H +  ++LL +E I+       G    L+ A +AGQ +V+R L  YG N+
Sbjct  41   GLNGLHLASKEGHVKMVVELLHKEIILETTTKKGNTALHIAALAGQDEVVRELVNYGANV  100

Query  104  NEKTTRGYTLLHCAAAWGRLETLKALVE  131
            N ++ +G+T L+ AA     LE +K L+E
Sbjct  101  NAQSQKGFTPLYMAAQENHLEVVKFLLE  128
```

Score = 44.3 bits (103),  Expect = 0.013, Method: Compositional matrix adjust.
Identities = 29/107 (27%), Positives = 48/107 (44%), Gaps = 7/107 (6%)

```
Query  63   HEQAMQLLLEED-------IVGRNLLYAACMAGQSDVIRALAKYGVNLNEKTTRGYTLLH  115
            H +  ++LL++          + G   L+ AC      V+   L K G +++  T  G T LH
Sbjct  346  HHRVAKVLLDKGAKPNSRALNGFTPLHIACKKNHIRVMELLLKTGASIDAVTESGLTPLH  405

Query  116  CAAAWGRLETLKALVELDVDIEALNFREERARDVAARYSQTECVEFL  162
             A+  G L  +K L++         N + E     +AAR   TE  ++L
Sbjct  406  VASFMGHLPIVKNLLQRGASPNVSNVKVETPLHMAARAGHTEVAKYL  452
```

Score = 43.5 bits (101),  Expect = 0.020, Method: Compositional matrix adjust.
Identities = 27/87 (31%), Positives = 43/87 (49%), Gaps = 0/87 (0%)

```
Query  81   LYAACMAGQSDVIRALAKYGVNLNEKTTRGYTLLHCAAAWGRLETLKALVELDVDIEALN  140
            L+ A    G + +++ L + G + N  TT G+T LH AA  G ++T  AL+E +      +
Sbjct  470  LHCAARIGHTGMVKLLLENGASPNLATTAGHTPLHTAAREGHVDTALALLEKEASQACMT  529

Query  141  FREERARDVAARYSQTECVEFLDWADA  167
             +       VAA+Y +     E L   DA
Sbjct  530  KKGFTPLHVAAKYGKVRLAELLLEHDA  556
```

Score = 42.4 bits (98),  Expect = 0.051, Method: Compositional matrix adjust.
Identities = 25/86 (29%), Positives = 39/86 (45%), Gaps = 0/86 (0%)

```
Query  77   GRNLLYAACMAGQSDVIRALAKYGVNLNEKTTRGYTLLHCAAAWGRLETLKALVELDVDI  136
            G   L+     G    V   L K+GV ++   T   GYT LH A+ +G ++ +K L++   D+
Sbjct  664  GLTPLHLVSQEGHVPVADVLIKHGVTVDATTRMGYTPLHVASHYGNIKLVKFLLQHQADV  723

Query  137  EALNFREERARDVAARYSQTECVEFL  162
             A            AA+    T+ V  L
Sbjct  724  NAKTKLGYSPLHQAAQQGHTDIVTLL  749
```

Score = 35.0 bits (79),  Expect = 8.0, Method: Compositional matrix adjust.
Identities = 28/102 (27%), Positives = 46/102 (45%), Gaps = 7/102 (6%)

```
Query  68   QLLLEEDI----VGRN---LLYAACMAGQSDVIRALAKYGVNLNEKTTRGYTLLHCAAAW  120
            +LLLE D       G+N    L+ A       D+++ L    G + +      GYT LH AA
Sbjct  549  ELLLEHDAHPNAAGKNGLTPLHVAVHHNNLDIVKLLLPRGGSPHSPAWNGYTPLHIAAKQ  608

Query  121  GRLETLKALVELDVDIEALNFREERARDVAARYSQTECVEFL  162
             ++E  ++L++      A + +       +AA+   TE V  L
Sbjct  609  NQIEVARSLLQYGGSANAESVQGVTPLHLAAQEGHTEMVALL  650
```

Score = 35.0 bits (79),  Expect = 8.6, Method: Compositional matrix adjust.
Identities = 28/107 (26%), Positives = 44/107 (41%), Gaps = 7/107 (6%)

```
Query  63   HEQAMQLLLEED-------IVGRNLLYAACMAGQSDVIRALAKYGVNLNEKTTRGYTLLH  115
            H   ++LLLE             G   L+ A   G   D   AL +    +     T +G+T LH
Sbjct  478  HTGMVKLLLENGASPNLATTAGHTPLHTAAREGHVDTALALLEKEASQACMTKKGFTPLH  537

Query  116  CAAAWGRLETLKALVELDVDIEALNFREERARDVAARYSQTECVEFL  162
             AA +G++    + L+E D    A           VA  ++  + V+ L
```

```
Sbjct  538  VAAKYGKVRLAELLLEHDAHPNAAGKNGLTPLHVAVHHNNLDIVKLL  584
```

>**gb|ABG56392.1|** glucose-regulated protein 78 [Paralichthys olivaceus]
Length=654

```
 Score = 52.8 bits (125),  Expect = 3e-05, Method: Compositional matrix adjust.
 Identities = 27/75 (36%), Positives = 44/75 (58%), Gaps = 0/75 (0%)

Query  166  DARLTLKKYIAKVSLAVTDTEKGSGKLLKEDKNTILSACRAKNEWLETHTEASINELFEQ  225
            DAR  L+ Y   +   + D EK  GKL  +DK  I  A   K EW+E+H +A + +   +
Sbjct  558  DARNELESYAYSLKNQIGDKEKLGGKLSDDDKEAIEKAVEEKIEWMESHQDAELEDFQAK  617

Query  226  RQQLEDIVTPIFTKM  240
            +++LE++V PI +K+
Sbjct  618  KKELEEVVQPIISKL  632
```

>**ref|XP_002098422.1|** GE23960 [Drosophila yakuba]
 **gb|EDW98134.1|** GE23960 [Drosophila yakuba]
Length=1035

 **GENE ID: 6537881 Dyak\GE23960** | GE23960 gene product from transcript GE23960-RA
[Drosophila yakuba] (10 or fewer PubMed links)

```
 Score = 52.8 bits (125),  Expect = 3e-05, Method: Compositional matrix adjust.
 Identities = 37/117 (31%), Positives = 55/117 (47%), Gaps = 10/117 (8%)

Query  63   HEQAMQLLLEE-------DIVGRNLLYAACMAGQSDVIRALAKYG---VNLNEKTTRGYT  112
            HE  ++LLL         D  G + L+ A  AG+++++R L  +     + N +T    T
Sbjct  61   HEDIVRLLLAHEASPNLPDSRGSSPLHLAAWAGETEIVRLLLTHPYRPASANLQTIEQET  120

Query  113  LLHCAAAWGRLETLKALVELDVDIEALNFREERARDVAARYSQTECVEFLDWADARL  169
             LHCAA  G    L  L+  D D    N R E   D+AA+Y + + V+ L  A   L
Sbjct  121  PLHCAAQHGHTGALALLLHHDADPNMRNSRGETPLDLAAQYGRLQAVQMLIRAHPEL  177
```

>**ref|XP_237153.4|** PREDICTED: similar to ankyrin repeat domain 28 [Rattus norvegicus]
 **ref|XP_001066455.1|** PREDICTED: similar to ankyrin repeat domain 28 [Rattus norvegicus]
Length=1103

 **GENE ID: 301415 Ankrd44** | ankyrin repeat domain 44 [Rattus norvegicus]

```
 Score = 52.8 bits (125),  Expect = 3e-05, Method: Compositional matrix adjust.
 Identities = 40/135 (29%), Positives = 58/135 (42%), Gaps = 7/135 (5%)

Query  38   PLLQPALTGDVEGLQKIFEDPENPHHEQAMQLLLEE------DIVGRNLLYAACMAGQSD  91
            PLL      D  G   +     N H E    LL +       D   R  L+ A   G  D
Sbjct  192  PLLSSVNVSDRGGRTALHHAALNGHMEMVNLLLAKGANINAFDKKDRRALHWAAYMGHLD  251

Query  92   VIRALAKYGVNLNEKTTRGYTLLHCAAAWGRLETLKALVELDVDIEALNFREERARDVAA  151
            V+  L  +G  +  K  +GYT LH AA+ G++  +K L+ L V+I+ +N     A  +A
Sbjct  252  VVALLINHGAEVTCKDKKGYTPLHAAASNGQINVVKHLLNLGVEIDEINVYGNTALHIAC  311

Query  152  RYSQTECV-EFLDWA  165
               Q   V E +D+
Sbjct  312  YNGQDAVVNELIDYG  326


 Score = 45.8 bits (107),  Expect = 0.004, Method: Compositional matrix adjust.
 Identities = 33/100 (33%), Positives = 47/100 (47%), Gaps = 1/100 (1%)

Query  74   DIVGRNLLYAACMAGQSDVIRALAKYGVNLNEKTTRGYTLLHCAAAWGRLETLKALVELD  133
            D  GR  L+AA   G  + I+ L   G + ++K   G T LH AAA      +KALV
Sbjct  466  DTFGRTCLHAAAAGGNVECIKLLQSSGADFHKKDKCGRTPLHYAAANCHFHCIKALVTTG  525

Query  134  VDI-EALNFREERARDVAARYSQTECVEFLDWADARLTLK  172
             +I E  N+        AA     +C+EFL    DA  +++
Sbjct  526  ANINETDNWGRTALHYAAASDMDRKCLEFLLQNDANPSIR  565


 Score = 43.1 bits (100),  Expect = 0.028, Method: Compositional matrix adjust.
 Identities = 33/104 (31%), Positives = 51/104 (49%), Gaps = 16/104 (15%)

Query  72   EEDIVGRNLLYAACMAGQSDVIRALAKYGVNLNEKTTRGYTLLHCAAA--WGRLETLKAL  129
            E ++ G   L+ AC  GQ  V+   L  YG N+N+    G+T LH AAA   G L  L+ L
Sbjct  298  EINVYGNTALHIACYNGQDAVVNELIDYGANVNQPNNSGFTPLHFAAASTHGAL-CLELL  356

Query  130  VE--LDVDIE-----------ALNFREERARDVAARYSQTECVE  160
            V    DV+I+           A++ R  R++ +     + +CV+
Sbjct  357  VNNGADVNIQSKDGKSPLHMTAVHGRFTRSQTLIQNGGEIDCVD  400


 Score = 36.2 bits (82),  Expect = 3.1, Method: Compositional matrix adjust.
 Identities = 40/158 (25%), Positives = 64/158 (40%), Gaps = 11/158 (6%)

Query  81   LYAACMAGQSDVIRALAKYGVNLNEKTTRGYTLLHCAAAWGRLETLKALVELDVDIEALN  140
            L+ A +   SD  R L   G  ++   T G T LH AAA G +E +K L     D    +
Sbjct  440  LHLAALNAHSDCCRKLLSSGFEIDTPDTFGRTCLHAAAAGGNVECIKLLQSSGADFHKKD  499

Query  141  FREERARDVAARYSQTECVEFLDWADARLTLKKYIAKVSL---AVTDTEKGSGK-LLKED  196
                     AA      C++ L    A +       + +L   A +D ++   + LL+ D
Sbjct  500  KCGRTPLHYAAANCHFHCIKALVTTGANINETDNWGRTALHYAAASDMDRKCLEFLLQND  559

Query  197  KNTILSACRAKNEWLETHTEASINELFEQRQQLEDIVT  234
             N  +   R K  +   H  A+    +  RQ LE +++
Sbjct  560  ANPSI---RDKEGYNSIHYAAA----YGHRQCLELVMS  590


 Score = 35.8 bits (81),  Expect = 4.3, Method: Compositional matrix adjust.
 Identities = 47/204 (23%), Positives = 81/204 (39%), Gaps = 37/204 (18%)

Query  36   KNPLLQPALTGDVEGLQKIFEDPENPH--------------------HEQAMQLLLEE--  73
            + PL    + G    L+ + E  +NP                     H  A+ LLLE+
Sbjct  682  RTPLHASVINGHTLCLRLLLEIADNPEVVDVKDAKGQTPLMLAVAYGHIDAVSLLLEKEA  741
```

```
Query  74   -----DIVGRNLLYAACMAGQSDVIRALAKYGVNLNEKTTRGYTLLHCAAAWGRLETLKA  128
                 D VG   L+   M G  + ++ L +   ++  K +RG T LH AAA G    L
Sbjct  742  NVDAVDTVGCTALHRGIMTGHEECVQMLLEQEASILCKDSRGRTPLHYAAARGHATWLNE  801

Query  129  LVELDVDIEALNFREERARDVA--ARYSQTE-CVEFLDWADARLTLKKYIAK----VSLA  181
            LV++ +  E    ++ +        A Y+  E C+E L     +   +K+I      +  A
Sbjct  802  LVQIALSEEDCCLKDNQGYTPLHWACYNGNENCIEVL---LEQKCFRKFIGNPFTPLHCA  858

Query  182  VTDTEKGSGKLLKEDKNTILSACR  205
            + +  +     LL    ++ + +CR
Sbjct  859  IINGHESCASLLLGAIDSSIVSCR  882
```

Score = 35.0 bits (79), Expect = 7.8, Method: Compositional matrix adjust.
Identities = 32/127 (25%), Positives = 53/127 (41%), Gaps = 9/127 (7%)

```
Query  36   KNPLLQPALTGDVEGLQKIFEDPENPHHEQAMQLLLEEDIVGRNLLYAACMAGQSDVIRA  95
            K PL+Q    +GD E ++ +    E+ +            D   R  L+ A   G +++I
Sbjct  73   KPPLVQAIFSGDPEEIRLLIHKTEDVN---------ALDSEKRTPLHVAAFLGDAEIIEL  123

Query  96   LAKYGVNLNEKTTRGYTLLHCAAAWGRLETLKALVELDVDIEALNFREERARDVAARYSQ  155
            L   G  +N K     T LH A A    E ++ L++    D+ A +    +    VAA
Sbjct  124  LILSGARVNAKDNMWLTPLHRAVASRSEEAVQVLIKHSADVNARDKNWQSPVHVAAANKA  183

Query  156  TECVEFL  162
             +C E +
Sbjct  184  VKCAEVI  190
```

>**ref|XP_001101829.1|** PREDICTED: similar to UNCoordinated family member (unc-44) [Macaca mulatta]
Length=555

**GENE ID: 712746 LOC712746** | similar to UNCoordinated family member (unc-44) [Macaca mulatta]

Score = 52.8 bits (125), Expect = 3e-05, Method: Compositional matrix adjust.
Identities = 37/110 (33%), Positives = 58/110 (52%), Gaps = 11/110 (10%)

```
Query  63   HEQAMQLLL-------EEDIVGRNLLYAACMAGQSDVIRALAKYGVNLNEKTTRGYTLLH  115
            HEQA++LLL       EED  G N L  +   G   +++ L   G  ++ K+  G TLLH
Sbjct  116  HEQAVRLLLEHEAAVDEEDAFGMNALLLSAWFGHLRILQILVNSGAKIHCKSKDGLTLLH  175

Query  116  CAAAWGRLETLKALVELDVDIEALNFREERARDV---AARYSQTECVEFL  162
            CAA  G +  L  ++E D++  AL+   ++  R     AA + Q + ++FL
Sbjct  176  CAAQKGHVPVLAFIME-DLEDVALDHVDKLGRTAFHRAAEHGQLDALDFL  224
```

Score = 46.6 bits (109), Expect = 0.002, Method: Compositional matrix adjust.
Identities = 43/166 (25%), Positives = 64/166 (38%), Gaps = 26/166 (15%)

```
Query  23   EEEEAQEPEETGPKNPLLQPALTGDVEGLQKIFEDPENPH-----------------HEQ  65
            E E A + E+    N LL  A  G +  LQ +         H                 H
Sbjct  125  EHEAAVDEEDAFGMNALLLSAWFGHLRILQILVNSGAKIHCKSKDGLTLLHCAAQKGHVP  184

Query  66   AMQLLLEE---------DIVGRNLLYAACMAGQSDVIRALAKYGVNLNEKTTRGYTLLHC  116
             +  ++E+           D +GR    + A    GQ D +  L    G + + K    G T LH
Sbjct  185  VLAFIMEDLEDVALDHVDKLGRTAFHRAAEHGQLDALDFLVGSGCDHSVKDKEGNTALHL  244

Query  117  AAAWGRLETLKALVELDVDIEALNFREERARDVAARYSQTECVEFL  162
            AA  G +  L+ LV++ +D+E  N      A   AA  +  +CV  L
Sbjct  245  AAGRGHMAVLQRLVDIGLDLEEQNAEGLTALHAAAGGTHPDCVRLL  290
```

>**emb|CAA48801.1|** erythroid ankyrin [Mus musculus]
Length=1848

**GENE ID: 11733 Ank1** | ankyrin 1, erythroid [Mus musculus]
(Over 10 PubMed links)

Score = 52.8 bits (125), Expect = 3e-05, Method: Compositional matrix adjust.
Identities = 30/88 (34%), Positives = 49/88 (55%), Gaps = 6/88 (6%)

```
Query  50   GLQKIFEDPENPHHEQAMQLLLEEDIV------GRNLLYAACMAGQSDVIRALAKYGVNL  103
            GL   +     +  H +  ++LL +E I+      G   L+ A +AGQ +V+R L  YG N+
Sbjct  49   GLNGLHLASKEGHVKMVVELLHKEIILETTTKKGNTALHIAALAGQDEVVRELVNYGANV  108

Query  104  NEKTTRGYTLLHCAAAWGRLETLKALVE  131
            N ++ +G+T L+ AA    LE +K L+E
Sbjct  109  NAQSQKGFTPLYMAAQENHLEVVKFLLE  136
```

Score = 44.3 bits (103), Expect = 0.012, Method: Compositional matrix adjust.
Identities = 29/107 (27%), Positives = 48/107 (44%), Gaps = 7/107 (6%)

```
Query  63   HEQAMQLLLEED-------IVGRNLLYAACMAGQSDVIRALAKYGVNLNEKTTRGYTLLH  115
            H +  ++LL++          + G    L+ AC       V+  L K G +++  T  G T LH
Sbjct  354  HHRVAKVLLDKGAKPNSRALNGFTPLHIACKKNHIRVMELLLKTGASIDAVTESGLTPLH  413

Query  116  CAAAWGRLETLKALVELDVDIEALNFREERARDVAARYSQTECVEFL  162
             A+  G L  +K L++        N + E    +AAR   TE  ++L
Sbjct  414  VASFMGHLPIVKNLLQRGASPNVSNVKVETPLHMAARAGHTEVAKYL  460
```

Score = 43.5 bits (101), Expect = 0.020, Method: Compositional matrix adjust.
Identities = 27/87 (31%), Positives = 43/87 (49%), Gaps = 0/87 (0%)

```
Query  81   LYAACMAGQSDVIRALAKYGVNLNEKTTRGYTLLHCAAAWGRLETLKALVELDVDIEALN  140
            L+ A    G + +++ L + G + N  TT G+T LH AA  G ++T  AL+E +      +
Sbjct  478  LHCAARIGHTGMVKLLLENGASPNLATTAGHTPLHTAAREGHVDTALALLEKEASQACMT  537

Query  141  FREERARDVAARYSQTECVEFLDWADA  167
             +        VAA+Y +    E L   DA
Sbjct  538  KKGFTPLHVAAKYGKVRLAELLLEHDA  564
```

Score = 42.4 bits (98), Expect = 0.052, Method: Compositional matrix adjust.

```
 Identities = 25/86 (29%), Positives = 39/86 (45%), Gaps = 0/86 (0%)

Query  77   GRNLLYAACMAGQSDVIRALAKYGVNLNEKTTRGYTLLHCAAAWGRLETLKALVELDVDI  136
            G   L+     G   V   L K+GV ++  T  GYT LH A+ +G ++ +K L++   D+
Sbjct  672  GLTPLHLVSQEGHVPVADVLIKHGVTVDATTRMGYTPLHVASHYGNIKLVKFLLQHQADV  731

Query  137  EALNFREERARDVAARYSQTECVEFL  162
             A          AA+   T+ V  L
Sbjct  732  NAKTKLGYSPLHQAAQQGHTDIVTLL  757


 Score = 35.0 bits (79),  Expect = 7.6, Method: Compositional matrix adjust.
 Identities = 28/102 (27%), Positives = 46/102 (45%), Gaps = 7/102 (6%)

Query  68   QLLLEEDI----VGRN---LLYAACMAGQSDVIRALAKYGVNLNEKTTRGYTLLHCAAAW  120
            +LLLE D      G+N    L+ A      D+++ L   G + +     GYT LH AA
Sbjct  557  ELLLEHDAHPNAAGKNGLTPLHVAVHHNNLDIVKLLLPRGGSPHSPAWNGYTPLHIAAKQ  616

Query  121  GRLETLKALVELDVDIEALNFREERARDVAARYSQTECVEFL  162
             ++E  ++L++      A + +      +AA+   TE V  L
Sbjct  617  NQIEVARSLLQYGGSANAESVQGVTPLHLAAQEGHTEMVALL  658


 Score = 35.0 bits (79),  Expect = 8.5, Method: Compositional matrix adjust.
 Identities = 28/107 (26%), Positives = 44/107 (41%), Gaps = 7/107 (6%)

Query  63   HEQAMQLLLEED-------IVGRNLLYAACMAGQSDVIRALAKYGVNLNEKTTRGYTLLH  115
            H   ++LLLE           G   L+ A   G  D   AL +   +    T +G+T LH
Sbjct  486  HTGMVKLLLENGASPNLATTAGHTPLHTAAREGHVDTALALLEKEASQACMTKKGFTPLH  545

Query  116  CAAAWGRLETLKALVELDVDIEALNFREERARDVAARYSQTECVEFL  162
             AA +G++   + L+E D    A          VA  ++  + V+ L
Sbjct  546  VAAKYGKVRLAELLLEHDAHPNAAGKNGLTPLHVAVHHNNLDIVKLL  592
```

>**gb|EAW63241.1|** ankyrin 1, erythrocytic, isoform CRA_a [Homo sapiens]
Length=1726

**GENE ID: 286 ANK1** | ankyrin 1, erythrocytic [Homo sapiens]
(Over 10 PubMed links)

```
 Score = 52.8 bits (125),  Expect = 3e-05, Method: Compositional matrix adjust.
 Identities = 30/88 (34%), Positives = 49/88 (55%), Gaps = 6/88 (6%)

Query  50   GLQKIFEDPENPHHEQAMQLLLEEDIV------GRNLLYAACMAGQSDVIRALAKYGVNL  103
            GL  +    +  H +  ++LL +E I+      G   L+ A +AGQ +V+R L  YG N+
Sbjct  45   GLNGLHLASKEGHVKMVVELLHKEIILETTTKKGNTALHIAALAGQDEVVRELVNYGANV  104

Query  104  NEKTTRGYTLLHCAAAWGRLETLKALVE  131
            N ++ +G+T L+ AA    LE +K L+E
Sbjct  105  NAQSQKGFTPLYMAAQENHLEVVKFLLE  132


 Score = 46.2 bits (108),  Expect = 0.003, Method: Compositional matrix adjust.
 Identities = 28/87 (32%), Positives = 44/87 (50%), Gaps = 0/87 (0%)

Query  81   LYAACMAGQSDVIRALAKYGVNLNEKTTRGYTLLHCAAAWGRLETLKALVELDVDIEALN  140
            L+ A   G +++++ L +   N N  TT G+T LH AA  G +ET+ AL+E +     +
Sbjct  474  LHCAARIGHTNMVKLLLENNANPNLATTAGHTPLHIAAREGHVETVLALLEKEASQACMT  533

Query  141  FREERARDVAARYSQTECVEFLDWADA  167
             +      VAA+Y +    E L   DA
Sbjct  534  KKGFTPLHVAAKYGKVRVAELLLERDA  560


 Score = 45.1 bits (105),  Expect = 0.008, Method: Compositional matrix adjust.
 Identities = 31/117 (26%), Positives = 52/117 (44%), Gaps = 7/117 (5%)

Query  63   HEQAMQLLLEED-------IVGRNLLYAACMAGQSDVIRALAKYGVNLNEKTTRGYTLLH  115
            H +  ++LL++        + G   L+ AC      V+  L K G +++  T  G T LH
Sbjct  350  HHRVAKVLLDKGAKPNSRALNGFTPLHIACKKNHVRVMELLLKTGASIDAVTESGLTPLH  409

Query  116  CAAAWGRLETLKALVELDVDIEALNFREERARDVAARYSQTECVEFLDWADARLTLK  172
             A+  G L  +K L++        N + E    +AAR   TE  ++L    A++  K
Sbjct  410  VASFMGHLPIVKNLLQRGASPNVSNVKVETPLHMAARAGHTEVAKYLLQNKAKVNAK  466


 Score = 41.6 bits (96),  Expect = 0.085, Method: Compositional matrix adjust.
 Identities = 31/108 (28%), Positives = 48/108 (44%), Gaps = 9/108 (8%)

Query  63   HEQAMQLLLEEDIVGRNL--------LYAACMAGQSDVIRALAKYGVNLNEKTTRGYTLL  114
            H + + LLL +   G NL        L+     G   V   L K+GV ++  T  GYT L
Sbjct  647  HAEMVALLLSKQANG-NLGNKSGLTPLHLVAQEGHVPVADVLIKHGVMVDATTRMGYTPL  705

Query  115  HCAAAWGRLETLKALVELDVDIEALNFREERARDVAARYSQTECVEFL  162
            H A+ +G ++ +K L++   D+ A           AA+   T+ V  L
Sbjct  706  HVASHYGNIKLVKFLLQHQADVNAKTKLGYSPLHQAAQQGHTDIVTLL  753


 Score = 38.5 bits (88),  Expect = 0.67, Method: Compositional matrix adjust.
 Identities = 40/167 (23%), Positives = 63/167 (37%), Gaps = 7/167 (4%)

Query  63   HEQAMQLLLEE----DIV---GRNLLYAACMAGQSDVIRALAKYGVNLNEKTTRGYTLLH  115
            H + M+LLL+     D V   G   L+ A   G   +++ L + G + N    +  T LH
Sbjct  383  HVRVMELLLKTGASIDAVTESGLTPLHVASFMGHLPIVKNLLQRGASPNVSNVKVETPLH  442

Query  116  CAAAWGRLETLKALVELDVDIEALNFREERARDVAARYSQTECVEFLDWADARLTLKKYI  175
             AA  G  E  K L++    + A    ++     AAR   T  V+ L   +A   L
Sbjct  443  MAARAGHTEVAKYLLQNKAKVNAKAKDDQTPLHCAARIGHTNMVKLLLENNANPNLATTA  502

Query  176  AKVSLAVTDTEKGSGKLLKEDKNTILSACRAKNEWLETHTEASINEL  222
                L +   E     +L   +     AC  K  +   H  A   ++
Sbjct  503  GHTPLHIAAREGHVETVLALLEKEASQACMTKKGFTPLHVAAKYGKV  549


 Score = 36.2 bits (82),  Expect = 3.6, Method: Compositional matrix adjust.
 Identities = 27/107 (25%), Positives = 46/107 (42%), Gaps = 7/107 (6%)
```

```
Query  63    HEQAMQLLLEED-------IVGRNLLYAACMAGQSDVIRALAKYGVNLNEKTTRGYTLLH  115
             H   ++LLLE +         G   L+ A   G  + + AL +   +    T +G+T LH
Sbjct  482   HTNMVKLLLENNANPNLATTAGHTPLHIAAREGHVETVLALLEKEASQACMTKKGFTPLH  541

Query  116   CAAAWGRLETLKALVELDVDIEALNFREERARDVAARYSQTECVEFL  162
              AA +G++   + L+E D    A          VA  ++  + V+ L
Sbjct  542   VAAKYGKVRVAELLLERDAHPNAAGKNGLTPLHVAVHHNNLDIVKLL  588
```

>**dbj|BAE39999.1|** unnamed protein product [Mus musculus]
Length=655

**GENE ID: 14828 Hspa5** | heat shock protein 5 [Mus musculus]
(Over 100 PubMed links)

Score = 52.8 bits (125), Expect = 3e-05, Method: Compositional matrix adjust.
Identities = 26/59 (44%), Positives = 38/59 (64%), Gaps = 0/59 (0%)

```
Query  182   VTDTEKGSGKLLKEDKNTILSACRAKNEWLETHTEASINELFEQRQQLEDIVTPIFTKM  240
             + D EK  GKL  EDK T+  A   K EWLE+H +A I +   ++++LE+IV PI +K+
Sbjct  577   IGDKEKLGGKLSSEDKETMEKAVEEKIEWLESHQDADIEDFKAKKKELEEIVQPIISKL  635
```

>**emb|CAQ52953.1|** CD4-specific ankyrin repeat protein D23.2 [synthetic construct]
Length=136

Score = 52.8 bits (125), Expect = 3e-05, Method: Compositional matrix adjust.
Identities = 34/87 (39%), Positives = 45/87 (51%), Gaps = 1/87 (1%)

```
Query  76    VGRNLLYAACMAGQSDVIRALAKYGVNLNEKTTRGYTLLHCAAAWGRLETLKALVELDVD  135
             +G+ LL AA  AGQ D +R L   G ++N   T G T LH AAAWG LE +  L++   D
Sbjct  14    LGKKLLEAA-RAGQDDEVRILMANGADVNATDTLGRTPLHMAAAWGHLEIVDVLLKHGAD  72

Query  136   IEALNFREERARDVAARYSQTECVEFL  162
             + A+         +AA     E VE L
Sbjct  73    VNAIEEVGMTPLHLAAFLGHLEIVEVL  99
```

Score = 46.6 bits (109), Expect = 0.002, Method: Compositional matrix adjust.
Identities = 27/89 (30%), Positives = 45/89 (50%), Gaps = 0/89 (0%)

```
Query  74    DIVGRNLLYAACMAGQSDVIRALAKYGVNLNEKTTRGYTLLHCAAAWGRLETLKALVELD  133
             D +GR  L+ A   G  +++  L K+G ++N     G T LH AA  G LE ++ L++
Sbjct  44    DTLGRTPLHMAAAWGHLEIVDVLLKHGADVNAIEEVGMTPLHLAAFLGHLEIVEVLLKSG  103

Query  134   VDIEALNFREERARDVAARYSQTECVEFL  162
              D+ A +   + A D++  Y   +  E L
Sbjct  104   ADVNAQDKFGKTAFDISIDYGNEDLAEIL  132
```

>**ref|XP_001319255.1|** ankyrin repeat protein [Trichomonas vaginalis G3]
**gb|EAY07032.1|** ankyrin repeat protein, putative [Trichomonas vaginalis G3]
Length=770

**GENE ID: 4764909 TVAG_311570** | ankyrin repeat protein
[Trichomonas vaginalis G3] (10 or fewer PubMed links)

Score = 52.8 bits (125), Expect = 3e-05, Method: Compositional matrix adjust.
Identities = 36/130 (27%), Positives = 61/130 (46%), Gaps = 7/130 (5%)

```
Query  58    PENPHHEQAMQLLL-------EEDIVGRNLLYAACMAGQSDVIRALAKYGVNLNEKTTRG  110
              EN + ++  +LL+       E+D VG+  L+ A     + +    L  +G+N+NEKT  G
Sbjct  419   AENSNGKETAELLISHGININEKDNVGKTALHYAAYYNRKETAEVLISHGININEKTNDG  478

Query  111   YTLLHCAAAWGRLETLKALVELDVDIEALNFREERARDVAARYSQTECVEFLDWADARLT  170
              T LH A ++   ET + L+   ++I   +   + A   A  Y+  E VE L      +
Sbjct  479   ETALHIATSYNNRETAEILISHGININEKDNVGKTALHYATYYNNRETVELLISHGININ  538

Query  171   LKKYIAKVSL  180
              K  + K +L
Sbjct  539   EKDNVGKTAL  548
```

Score = 52.0 bits (123), Expect = 6e-05, Method: Compositional matrix adjust.
Identities = 36/124 (29%), Positives = 58/124 (46%), Gaps = 2/124 (1%)

```
Query  72    EEDIVGRNLLYAACMAGQSDVIRALAKYGVNLNEKTTRGYTLLHCAAAWGRLETLKALVE  131
             E+D VG+  L+ A      + +  L  +G+N+NEK   G T LH AA + R ET + L+
Sbjct  506   EKDNVGKTALHYATYYNNRETVELLISHGININEKDNVGKTALHYAAYYNRKETAEILIS  565

Query  132   LDVDIEALNFREERARDVAARYSQTECVEFLDWADARLTLKKYIAKVSL--AVTDTEKGS  189
               ++I   +   + A   A  Y+  E  E L      +  K  + K +L  A T   K +
Sbjct  566   HGININEKDNDGKTALHYATYYNNRETAEILISHGININEKDNVGKTALHYAATGNSKET  625

Query  190   GKLL  193
              ++L
Sbjct  626   AEVL  629
```

Score = 51.2 bits (121), Expect = 1e-04, Method: Compositional matrix adjust.
Identities = 29/91 (31%), Positives = 45/91 (49%), Gaps = 0/91 (0%)

```
Query  72    EEDIVGRNLLYAACMAGQSDVIRALAKYGVNLNEKTTRGYTLLHCAAAWGRLETLKALVE  131
             E+D VG+  L+ A      +    L  +G+N+NEKT  G T LH A ++   ET + L+
Sbjct  605   EKDNVGKTALHYAATGNSKETAEVLISHGININEKTNDGETALHIATSYNNRETAEILIS  664

Query  132   LDVDIEALNFREERARDVAARYSQTECVEFL  162
               ++I       + A   AA Y++ E  E L
Sbjct  665   HGININEKTNNGKTALHCAAYYNRKETAELL  695
```

Score = 50.1 bits (118), Expect = 2e-04, Method: Compositional matrix adjust.
Identities = 28/86 (32%), Positives = 42/86 (48%), Gaps = 0/86 (0%)

```
Query  77    GRNLLYAACMAGQSDVIRALAKYGVNLNEKTTRGYTLLHCAAAWGRLETLKALVELDVDI  136
             G   L+ A      +    L  +G+N+NEKT  G T LHCAA + R ET + L+   + I
Sbjct  643   GETALHIATSYNNRETAEILISHGININEKTNNGKTALHCAAYYNRKETAELLISYGISI  702
```

```
Query  137  EALNFREERARDVAARYSQTECVEFL  162
               +   + A  +AA ++  E  E L
Sbjct  703  NEKDNDGKTALHIAADHNGKEIAELL  728


 Score = 43.9 bits (102),  Expect = 0.016, Method: Compositional matrix adjust.
 Identities = 27/89 (30%), Positives = 44/89 (49%), Gaps = 0/89 (0%)

Query  72   EEDIVGRNLLYAACMAGQSDVIRALAKYGVNLNEKTTRGYTLLHCAAAWGRLETLKALVE  131
            ++D  G   L+ A      +++   L  +G+N+NEKT  G T LH AA+    ET + L+
Sbjct  307  QKDYAGETALHNAAYYNSNEIAEVLISHGININEKTQYGKTALHIAASENSKETAEVLIS  366

Query  132  LDVDIEALNFREERARDVAARYSQTECVE  160
              ++I   +   E A  +AA  +  E  E
Sbjct  367  HGININEKDNDGETALRIAASENNKETAE  395


 Score = 43.1 bits (100),  Expect = 0.026, Method: Compositional matrix adjust.
 Identities = 28/91 (30%), Positives = 41/91 (45%), Gaps = 0/91 (0%)

Query  72   EEDIVGRNLLYAACMAGQSDVIRALAKYGVNLNEKTTRGYTLLHCAAAWGRLETLKALVE  131
            E+D  G+  L+ A       +    L  +G+N+NEK   G T LH AA     ET + L+
Sbjct  572  EKDNDGKTALHYATYYNNRETAEILISHGININEKDNVGKTALHYAATGNSKETAEVLIS  631

Query  132  LDVDIEALNFREERARDVAARYSQTECVEFL  162
              ++I        E A  +A  Y+  E  E L
Sbjct  632  HGININEKTNDGETALHIATSYNNRETAEIL  662


 Score = 41.2 bits (95),  Expect = 0.11, Method: Compositional matrix adjust.
 Identities = 25/86 (29%), Positives = 40/86 (46%), Gaps = 0/86 (0%)

Query  77   GRNLLYAACMAGQSDVIRALAKYGVNLNEKTTRGYTLLHCAAAWGRLETLKALVELDVDI  136
            G   L+ A      +    L  +G+N+NEK   G T LH A  +   ET++ L+   ++I
Sbjct  478  GETALHIATSYNNRETAEILISHGININEKDNVGKTALHYATYYNNRETVELLISHGINI  537

Query  137  EALNFREERARDVAARYSQTECVEFL  162
               +   + A   AA Y++ E  E L
Sbjct  538  NEKDNVGKTALHYAAYYNRKETAEIL  563


 Score = 35.8 bits (81),  Expect = 4.0, Method: Compositional matrix adjust.
 Identities = 24/86 (27%), Positives = 38/86 (44%), Gaps = 0/86 (0%)

Query  77   GRNLLYAACMAGQSDVIRALAKYGVNLNEKTTRGYTLLHCAAAWGRLETLKALVELDVDI  136
            G+  L+ A     + +    L  YG+++NEK    G T LH AA      E  + L+   + I
Sbjct  676  GKTALHCAAYYNRKETAELLISYGISINEKDNDGKTALHIAADHNGKEIAELLISYGISI  735

Query  137  EALNFREERARDVAARYSQTECVEFL  162
               +   + A  +AA  +  E  E L
Sbjct  736  NEKDNDGKTALHIAADQNSKETAELL  761


 Score = 35.8 bits (81),  Expect = 4.9, Method: Compositional matrix adjust.
 Identities = 21/65 (32%), Positives = 33/65 (50%), Gaps = 0/65 (0%)

Query  72   EEDIVGRNLLYAACMAGQSDVIRALAKYGVNLNEKTTRGYTLLHCAAAWGRLETLKALVE  131
            E+D  G+  L+ A      ++    L  YG+++NEK   G T LH AA     ET + L+
Sbjct  704  EKDNDGKTALHIAADHNGKEIAELLISYGISINEKDNDGKTALHIAADQNSKETAELLIS  763

Query  132  LDVDI  136
              ++I
Sbjct  764  HGINI  768
```

>**sp|Q02357.2|ANK1_MOUSE** RecName: Full=Ankyrin-1; AltName: Full=Erythrocyte ankyrin
Length=1862

**GENE ID: 11733 Ank1** | ankyrin 1, erythroid [Mus musculus]
(Over 10 PubMed links)

```
 Score = 52.8 bits (125),  Expect = 3e-05, Method: Compositional matrix adjust.
 Identities = 30/88 (34%), Positives = 49/88 (55%), Gaps = 6/88 (6%)

Query  50   GLQKIFEDPENPHHEQAMQLLLEEDIV------GRNLLYAACMAGQSDVIRALAKYGVNL  103
            GL  +     +  H +  ++LL +E I+      G    L+ A +AGQ +V+R L  YG N+
Sbjct  41   GLNGLHLASKEGHVKMVVELLHKEIILETTTKKGNTALHIAALAGQDEVVRELVNYGANV  100

Query  104  NEKTTRGYTLLHCAAAWGRLETLKALVE  131
            N ++ +G+T L+ AA    LE +K L+E
Sbjct  101  NAQSQKGFTPLYMAAQENHLEVVKFLLE  128


 Score = 44.3 bits (103),  Expect = 0.013, Method: Compositional matrix adjust.
 Identities = 29/107 (27%), Positives = 48/107 (44%), Gaps = 7/107 (6%)

Query  63   HEQAMQLLLEED-------IVGRNLLYAACMAGQSDVIRALAKYGVNLNEKTTRGYTLLH  115
            H +  ++LL++           + G   L+ AC       V+   L K G +++  T  G T LH
Sbjct  346  HHRVAKVLLDKGAKPNSRALNGFTPLHIACKKNHIRVMELLLKTGASIDAVTESGLTPLH  405

Query  116  CAAAWGRLETLKALVELDVDIEALNFREERARDVAARYSQTECVEFL  162
             A+  G L  +K L++         N + E    +AAR   TE  ++L
Sbjct  406  VASFMGHLPIVKNLLQRGASPNVSNVKVETPLHMAARAGHTEVAKYL  452


 Score = 43.5 bits (101),  Expect = 0.020, Method: Compositional matrix adjust.
 Identities = 27/87 (31%), Positives = 43/87 (49%), Gaps = 0/87 (0%)

Query  81   LYAACMAGQSDVIRALAKYGVNLNEKTTRGYTLLHCAAAWGRLETLKALVELDVDIEALN  140
            L+ A    G + +++ L + G + N  TT G+T LH AA  G ++T  AL+E +      +
Sbjct  470  LHCAARIGHTGMVKLLLENGASPNLATTAGHTPLHTAAREGHVDTALALLEKEASQACMT  529

Query  141  FREERARDVAARYSQTECVEFLDWADA  167
             +       VAA+Y  +    E L   DA
Sbjct  530  KKGFTPLHVAAKYGKVRLAELLLEHDA  556
```

```
 Score = 42.4 bits (98),  Expect = 0.053, Method: Compositional matrix adjust.
 Identities = 25/86 (29%), Positives = 39/86 (45%), Gaps = 0/86 (0%)

Query  77   GRNLLYAACMAGQSDVIRALAKYGVNLNEKTTRGYTLLHCAAAWGRLETLKALVELDVDI  136
            G   L+     G   V   L K+GV ++  T  GYT LH A+ +G ++ +K L++   D+
Sbjct  664  GLTPLHLVSQEGHVPVADVLIKHGVTVDATTRMGYTPLHVASHYGNIKLVKFLLQHQADV  723

Query  137  EALNFREERARDVAARYSQTECVEFL  162
             A           AA+   T+ V  L
Sbjct  724  NAKTKLGYSPLHQAAQQGHTDIVTLL  749


 Score = 35.0 bits (79),  Expect = 7.9, Method: Compositional matrix adjust.
 Identities = 28/102 (27%), Positives = 46/102 (45%), Gaps = 7/102 (6%)

Query  68   QLLLEEDI----VGRN---LLYAACMAGQSDVIRALAKYGVNLNEKTTRGYTLLHCAAAW  120
            +LLLE D      G+N    L+ A      D+++ L   G + +     GYT LH AA
Sbjct  549  ELLLEHDAHPNAAGKNGLTPLHVAVHHNNLDIVKLLLPRGGSPHSPAWNGYTPLHIAAKQ  608

Query  121  GRLETLKALVELDVDIEALNFREERARDVAARYSQTECVEFL  162
             ++E  ++L++      A + +      +AA+   TE V  L
Sbjct  609  NQIEVARSLLQYGGSANAESVQGVTPLHLAAQEGHTEMVALL  650


 Score = 35.0 bits (79),  Expect = 8.4, Method: Compositional matrix adjust.
 Identities = 28/107 (26%), Positives = 44/107 (41%), Gaps = 7/107 (6%)

Query  63   HEQAMQLLLEED-------IVGRNLLYAACMAGQSDVIRALAKYGVNLNEKTTRGYTLLH  115
            H   ++LLLE           G   L+ A   G  D   AL +   +    T +G+T LH
Sbjct  478  HTGMVKLLLENGASPNLATTAGHTPLHTAAREGHVDTALALLEKEASQACMTKKGFTPLH  537

Query  116  CAAAWGRLETLKALVELDVDIEALNFREERARDVAARYSQTECVEFL  162
             AA +G++   + L+E D    A          VA  ++  + V+ L
Sbjct  538  VAAKYGKVRLAELLLEHDAHPNAAGKNGLTPLHVAVHHNNLDIVKLL  584
```

>**gb|AAA37236.1|** ankyrin
Length=1862

**GENE ID: 11733 Ank1** | ankyrin 1, erythroid [Mus musculus]
(Over 10 PubMed links)

```
 Score = 52.8 bits (125),  Expect = 3e-05, Method: Compositional matrix adjust.
 Identities = 30/88 (34%), Positives = 49/88 (55%), Gaps = 6/88 (6%)

Query  50   GLQKIFEDPENPHHEQAMQLLLEEDIV------GRNLLYAACMAGQSDVIRALAKYGVNL  103
            GL  +    +  H +  ++LL +E I+      G   L+ A +AGQ +V+R L  YG N+
Sbjct  41   GLNGLHLASKEGHVKMVVELLHKEIILETTTKKGNTALHIAALAGQDEVVRELVNYGANV  100

Query  104  NEKTTRGYTLLHCAAAWGRLETLKALVE  131
            N ++ +G+T L+ AA    LE +K L+E
Sbjct  101  NAQSQKGFTPLYMAAQENHLEVVKFLLE  128


 Score = 44.3 bits (103),  Expect = 0.013, Method: Compositional matrix adjust.
 Identities = 29/107 (27%), Positives = 48/107 (44%), Gaps = 7/107 (6%)

Query  63   HEQAMQLLLEED-------IVGRNLLYAACMAGQSDVIRALAKYGVNLNEKTTRGYTLLH  115
            H +  ++LL++        + G   L+ AC      V+  L K G +++  T  G T LH
Sbjct  346  HHRVAKVLLDKGAKPNSRALNGFTPLHIACKKNHIRVMELLLKTGASIDAVTESGLTPLH  405

Query  116  CAAAWGRLETLKALVELDVDIEALNFREERARDVAARYSQTECVEFL  162
             A+  G L  +K L++        N + E    +AAR   TE  ++L
Sbjct  406  VASFMGHLPIVKNLLQRGASPNVSNVKVETPLHMAARAGHTEVAKYL  452


 Score = 43.5 bits (101),  Expect = 0.020, Method: Compositional matrix adjust.
 Identities = 27/87 (31%), Positives = 43/87 (49%), Gaps = 0/87 (0%)

Query  81   LYAACMAGQSDVIRALAKYGVNLNEKTTRGYTLLHCAAAWGRLETLKALVELDVDIEALN  140
            L+ A   G + +++ L + G + N  TT G+T LH AA  G ++T  AL+E +     +
Sbjct  470  LHCAARIGHTGMVKLLLENGASPNLATTAGHTPLHTAAREGHVDTALALLEKEASQACMT  529

Query  141  FREERARDVAARYSQTECVEFLDWADA  167
             +      VAA+Y +    E L   DA
Sbjct  530  KKGFTPLHVAAKYGKVRLAELLLEHDA  556


 Score = 41.2 bits (95),  Expect = 0.10, Method: Compositional matrix adjust.
 Identities = 25/86 (29%), Positives = 39/86 (45%), Gaps = 0/86 (0%)

Query  77   GRNLLYAACMAGQSDVIRALAKYGVNLNEKTTRGYTLLHCAAAWGRLETLKALVELDVDI  136
            G   L+     G   V   L K+GV ++  T  GYT LH A+ +G ++ +K L++   D+
Sbjct  664  GLTPLHLVSQEGHVLVADVLIKHGVTVDATTRMGYTPLHVASHYGNIKLVKFLLQHQADV  723

Query  137  EALNFREERARDVAARYSQTECVEFL  162
             A           AA+   T+ V  L
Sbjct  724  NAKTKLGYSPLHQAAQQGHTDIVTLL  749


 Score = 35.0 bits (79),  Expect = 8.5, Method: Compositional matrix adjust.
 Identities = 28/102 (27%), Positives = 46/102 (45%), Gaps = 7/102 (6%)

Query  68   QLLLEEDI----VGRN---LLYAACMAGQSDVIRALAKYGVNLNEKTTRGYTLLHCAAAW  120
            +LLLE D      G+N    L+ A      D+++ L   G + +     GYT LH AA
Sbjct  549  ELLLEHDAHPNAAGKNGLTPLHVAVHHNNLDIVKLLLPRGGSPHSPAWNGYTPLHIAAKQ  608

Query  121  GRLETLKALVELDVDIEALNFREERARDVAARYSQTECVEFL  162
             ++E  ++L++      A + +      +AA+   TE V  L
Sbjct  609  NQIEVARSLLQYGGSANAESVQGVTPLHLAAQEGHTEMVALL  650


 Score = 35.0 bits (79),  Expect = 8.6, Method: Compositional matrix adjust.
 Identities = 28/107 (26%), Positives = 44/107 (41%), Gaps = 7/107 (6%)

Query  63   HEQAMQLLLEED-------IVGRNLLYAACMAGQSDVIRALAKYGVNLNEKTTRGYTLLH  115
            H   ++LLLE           G   L+ A   G  D   AL +   +    T +G+T LH
Sbjct  478  HTGMVKLLLENGASPNLATTAGHTPLHTAAREGHVDTALALLEKEASQACMTKKGFTPLH  537
```

```
Query  116  CAAAWGRLETLKALVELDVDIEALNFREERARDVAARYSQTECVEFL  162
             AA +G++   + L+E D    A          VA  ++  + V+ L
Sbjct  538  VAAKYGKVRLAELLLEHDAHPNAAGKNGLTPLHVAVHHNNLDIVKLL  584
```

>**dbj|BAE34375.1|** unnamed protein product [Mus musculus]
Length=1744

**GENE ID: 11733 Ank1** | ankyrin 1, erythroid [Mus musculus]
(Over 10 PubMed links)

```
 Score = 52.8 bits (125),  Expect = 4e-05, Method: Compositional matrix adjust.
 Identities = 30/88 (34%), Positives = 49/88 (55%), Gaps = 6/88 (6%)

Query  50   GLQKIFEDPENPHHEQAMQLLLEEDIV------GRNLLYAACMAGQSDVIRALAKYGVNL  103
            GL  +    +  H +  ++LL +E I+      G   L+ A +AGQ +V+R L  YG N+
Sbjct  78   GLNGLHLASKEGHVKMVVELLHKEIILETTTKKGNTALHIAALAGQDEVVRELVNYGANV  137

Query  104  NEKTTRGYTLLHCAAAWGRLETLKALVE  131
            N ++ +G+T L+ AA    LE +K L+E
Sbjct  138  NAQSQKGFTPLYMAAQENHLEVVKFLLE  165


 Score = 44.3 bits (103),  Expect = 0.013, Method: Compositional matrix adjust.
 Identities = 29/107 (27%), Positives = 48/107 (44%), Gaps = 7/107 (6%)

Query  63   HEQAMQLLLEED-------IVGRNLLYAACMAGQSDVIRALAKYGVNLNEKTTRGYTLLH  115
            H +  ++LL++        + G   L+ AC      V+  L K G +++  T  G T LH
Sbjct  383  HHRVAKVLLDKGAKPNSRALNGFTPLHIACKKNHIRVMELLLKTGASIDAVTESGLTPLH  442

Query  116  CAAAWGRLETLKALVELDVDIEALNFREERARDVAARYSQTECVEFL  162
             A+  G L  +K L++        N + E    +AAR   TE  ++L
Sbjct  443  VASFMGHLPIVKNLLQRGASPNVSNVKVETPLHMAARAGHTEVAKYL  489


 Score = 42.4 bits (98),  Expect = 0.055, Method: Compositional matrix adjust.
 Identities = 25/86 (29%), Positives = 39/86 (45%), Gaps = 0/86 (0%)

Query  77   GRNLLYAACMAGQSDVIRALAKYGVNLNEKTTRGYTLLHCAAAWGRLETLKALVELDVDI  136
            G   L+     G   V   L K+GV ++  T  GYT LH A+ +G ++ +K L++   D+
Sbjct  701  GLTPLHLVSQEGHVPVADVLIKHGVTVDATTRMGYTPLHVASHYGNIKLVKFLLQHQADV  760

Query  137  EALNFREERARDVAARYSQTECVEFL  162
             A           AA+   T+ V  L
Sbjct  761  NAKTKLGYSPLHQAAQQGHTDIVTLL  786


 Score = 42.0 bits (97),  Expect = 0.062, Method: Compositional matrix adjust.
 Identities = 26/87 (29%), Positives = 42/87 (48%), Gaps = 0/87 (0%)

Query  81   LYAACMAGQSDVIRALAKYGVNLNEKTTRGYTLLHCAAAWGRLETLKALVELDVDIEALN  140
            L+ A   G + +++ L + G + N  TT G+T LH A   G ++T  AL+E +     +
Sbjct  507  LHCAARIGHTGMVKLLLENGASPNLATTAGHTPLHTATREGHVDTALALLEKEASQACMT  566

Query  141  FREERARDVAARYSQTECVEFLDWADA  167
             +      VAA+Y +    E L   DA
Sbjct  567  KKGFTPLHVAAKYGKVRLAELLLEHDA  593


 Score = 35.0 bits (79),  Expect = 8.4, Method: Compositional matrix adjust.
 Identities = 28/102 (27%), Positives = 46/102 (45%), Gaps = 7/102 (6%)

Query  68   QLLLEEDI----VGRN---LLYAACMAGQSDVIRALAKYGVNLNEKTTRGYTLLHCAAAW  120
            +LLLE D      G+N    L+ A      D+++ L   G + +     GYT LH AA
Sbjct  586  ELLLEHDAHPNAAGKNGLTPLHVAVHHNNLDIVKLLLPRGGSPHSPAWNGYTPLHIAAKQ  645

Query  121  GRLETLKALVELDVDIEALNFREERARDVAARYSQTECVEFL  162
             ++E  ++L++      A + +      +AA+   TE V  L
Sbjct  646  NQIEVARSLLQYGGSANAESVQGVTPLHLAAQEGHTEMVALL  687
```

>**ref|NP_112435.2|** ankyrin 1, erythroid isoform 2 [Mus musculus]
**gb|EDL32870.1|** ankyrin 1, erythroid [Mus musculus]
Length=1848

**GENE ID: 11733 Ank1** | ankyrin 1, erythroid [Mus musculus]
(Over 10 PubMed links)

```
 Score = 52.8 bits (125),  Expect = 4e-05, Method: Compositional matrix adjust.
 Identities = 30/88 (34%), Positives = 49/88 (55%), Gaps = 6/88 (6%)

Query  50   GLQKIFEDPENPHHEQAMQLLLEEDIV------GRNLLYAACMAGQSDVIRALAKYGVNL  103
            GL  +    +  H +  ++LL +E I+      G   L+ A +AGQ +V+R L  YG N+
Sbjct  49   GLNGLHLASKEGHVKMVVELLHKEIILETTTKKGNTALHIAALAGQDEVVRELVNYGANV  108

Query  104  NEKTTRGYTLLHCAAAWGRLETLKALVE  131
            N ++ +G+T L+ AA    LE +K L+E
Sbjct  109  NAQSQKGFTPLYMAAQENHLEVVKFLLE  136


 Score = 44.3 bits (103),  Expect = 0.013, Method: Compositional matrix adjust.
 Identities = 29/107 (27%), Positives = 48/107 (44%), Gaps = 7/107 (6%)

Query  63   HEQAMQLLLEED-------IVGRNLLYAACMAGQSDVIRALAKYGVNLNEKTTRGYTLLH  115
            H +  ++LL++        + G   L+ AC      V+  L K G +++  T  G T LH
Sbjct  354  HHRVAKVLLDKGAKPNSRALNGFTPLHIACKKNHIRVMELLLKTGASIDAVTESGLTPLH  413

Query  116  CAAAWGRLETLKALVELDVDIEALNFREERARDVAARYSQTECVEFL  162
             A+  G L  +K L++        N + E    +AAR   TE  ++L
Sbjct  414  VASFMGHLPIVKNLLQRGASPNVSNVKVETPLHMAARAGHTEVAKYL  460


 Score = 43.5 bits (101),  Expect = 0.021, Method: Compositional matrix adjust.
 Identities = 27/87 (31%), Positives = 43/87 (49%), Gaps = 0/87 (0%)

Query  81   LYAACMAGQSDVIRALAKYGVNLNEKTTRGYTLLHCAAAWGRLETLKALVELDVDIEALN  140
```

```
            L+ A   G + +++ L + G + N  TT G+T LH AA  G ++T  AL+E +     +
Sbjct  478  LHCAARIGHTGMVKLLLENGASPNLATTAGHTPLHTAAREGHVDTALALLEKEASQACMT  537

Query  141  FREERARDVAARYSQTECVEFLDWADA  167
             +      VAA+Y +    E L   DA
Sbjct  538  KKGFTPLHVAAKYGKVRLAELLLEHDA  564
```

Score = 42.4 bits (98), Expect = 0.054, Method: Compositional matrix adjust.
Identities = 25/86 (29%), Positives = 39/86 (45%), Gaps = 0/86 (0%)

```
Query  77   GRNLLYAACMAGQSDVIRALAKYGVNLNEKTTRGYTLLHCAAAWGRLETLKALVELDVDI  136
            G   L+     G   V   L K+GV ++  T  GYT LH A+ +G ++ +K L++   D+
Sbjct  672  GLTPLHLVSQEGHVPVADVLIKHGVTVDATTRMGYTPLHVASHYGNIKLVKFLLQHQADV  731

Query  137  EALNFREERARDVAARYSQTECVEFL  162
             A           AA+   T+ V  L
Sbjct  732  NAKTKLGYSPLHQAAQQGHTDIVTLL  757
```

Score = 35.0 bits (79), Expect = 7.9, Method: Compositional matrix adjust.
Identities = 28/102 (27%), Positives = 46/102 (45%), Gaps = 7/102 (6%)

```
Query  68   QLLLEEDI----VGRN---LLYAACMAGQSDVIRALAKYGVNLNEKTTRGYTLLHCAAAW  120
            +LLLE D      G+N    L+ A      D+++ L   G + +     GYT LH AA
Sbjct  557  ELLLEHDAHPNAAGKNGLTPLHVAVHHNNLDIVKLLLPRGGSPHSPAWNGYTPLHIAAKQ  616

Query  121  GRLETLKALVELDVDIEALNFREERARDVAARYSQTECVEFL  162
             ++E  ++L++      A + +      +AA+   TE V  L
Sbjct  617  NQIEVARSLLQYGGSANAESVQGVTPLHLAAQEGHTEMVALL  658
```

Score = 35.0 bits (79), Expect = 8.4, Method: Compositional matrix adjust.
Identities = 28/107 (26%), Positives = 44/107 (41%), Gaps = 7/107 (6%)

```
Query  63   HEQAMQLLLEED-------IVGRNLLYAACMAGQSDVIRALAKYGVNLNEKTTRGYTLLH  115
            H   ++LLLE           G   L+ A   G  D   AL +   +    T +G+T LH
Sbjct  486  HTGMVKLLLENGASPNLATTAGHTPLHTAAREGHVDTALALLEKEASQACMTKKGFTPLH  545

Query  116  CAAAWGRLETLKALVELDVDIEALNFREERARDVAARYSQTECVEFL  162
             AA +G++   + L+E D    A          VA ++   + V+ L
Sbjct  546  VAAKYGKVRLAELLLEHDAHPNAAGKNGLTPLHVAVHHNNLDIVKLL  592
```

>**gb|EDL22556.1|** ankyrin repeat and SAM domain containing 1, isoform CRA_a [Mus musculus]
Length=1126

**GENE ID: 224650 Anks1** | ankyrin repeat and SAM domain containing 1
[Mus musculus] (Over 10 PubMed links)

Score = 52.8 bits (125), Expect = 4e-05, Method: Compositional matrix adjust.
Identities = 37/117 (31%), Positives = 55/117 (47%), Gaps = 10/117 (8%)

```
Query  63   HEQAMQLLLEEDIV-------GRNLLYAACMAGQSDVIRALAKYG---VNLNEKTTRGYT  112
            H   +++LL  D +       G   L+ A   G + ++R L + G     +NE+     T
Sbjct  88   HRDVVEVLLRNDALTNVADSKGCYPLHLAAWKGDAQIVRLLIQQGPSHTRVNEQNNDNET  147

Query  113  LLHCAAAWGRLETLKALVELDVDIEALNFREERARDVAARYSQTECVEFLDWADARL  169
             LHCAA +G  E +KAL+E   D    N + E   D+AA Y + E V+ L  A   L
Sbjct  148  ALHCAAQYGHTEVVKALLEELTDPTMRNNKFETPLDLAALYGRLEVVKLLLGAHPNL  204
```

Score = 36.6 bits (83), Expect = 2.4, Method: Compositional matrix adjust.
Identities = 30/125 (24%), Positives = 58/125 (46%), Gaps = 6/125 (4%)

```
Query  38   PLLQPALTGDVEGLQKIFEDPENPHHEQAMQLLLEEDIVGRNLLYAACMAGQSDVIRALA  97
            PL   A  GD + ++ + +  + P H +      E++     L+ A    G ++V++AL
Sbjct  112  PLHLAAWKGDAQIVRLLIQ--QGPSHTRVN----EQNNDNETALHCAAQYGHTEVVKALL  165

Query  98   KYGVNLNEKTTRGYTLLHCAAAWGRLETLKALVELDVDIEALNFREERARDVAARYSQTE  157
            +   +   +  +  T L  AA +GRLE +K L+    ++ + + R+     +AAR
Sbjct  166  EELTDPTMRNNKFETPLDLAALYGRLEVVKLLLGAHPNLLSCSTRKHTPLHLAARNGHKA  225

Query  158  CVEFL  162
             V+ L
Sbjct  226  VVQVL  230
```

Score = 36.6 bits (83), Expect = 2.7, Method: Compositional matrix adjust.
Identities = 34/119 (28%), Positives = 58/119 (48%), Gaps = 12/119 (10%)

```
Query  63   HEQAMQLLLEE--DIVGRN-----LLYAACMAGQSDVIRALAKYGVNLNEKTTRGYTLLH  115
            H + ++ LLEE  D   RN      L  A + G+ +V++ L     NL   +TR +T LH
Sbjct  157  HTEVVKALLEELTDPTMRNNKFETPLDLAALYGRLEVVKLLLGAHPNLLSCSTRKHTPLH  216

Query  116  CAAAWGRLETLKALVELDVDIEALNFREERARDV--AARYSQTECVEFLDWADARLTLK  172
             AA  G    ++ L++  +D    N++ E    +  AA + +T+ V+ L  A   + +K
Sbjct  217  LAARNGHKAVVQVLLDAGMDS---NYQTEMGSALHEAALFGKTDVVQILLAAGIDVNIK  272
```

>**ref|XP_001750549.1|** hypothetical protein [Monosiga brevicollis MX1]
**gb|EDQ84645.1|** predicted protein [Monosiga brevicollis MX1]
Length=1123

**GENE ID: 5895782 MONBRDRAFT_39188** | hypothetical protein
[Monosiga brevicollis MX1]

Score = 52.8 bits (125), Expect = 4e-05, Method: Compositional matrix adjust.
Identities = 58/184 (31%), Positives = 78/184 (42%), Gaps = 20/184 (10%)

```
Query  73   EDIVGRNLLYAACMAGQSDVIRALAKYGVNLNEKTTRGYTLLHCAAAWGRLETLKALVEL  132
            ED  GR  L+ AC+ G   V+  L K+GV+   K     T LH A+ +GR+E +  L+E
Sbjct  42   EDADGRTPLHYACLCGHIMVVETLLKHGVDARVKNKDRLTPLHMASRFGRIEVVNLLLER  101

Query  133  DVDIEALNFREERARDVAARYSQTECVEFL--DWADARLTLK-KYIAKVSLAVTDTEKGS  189
             VD  A +  +  A   A      + VE L    ADA+   K K  A V +AV   +K
```

```
Sbjct  102  GVDARAKDKDDCTALHHACDLGHVQLVEMLLKHGADAQAKTKTKSTAAVVVAV---DKWL  158

Query  190  GKLLKEDKNT--------ILSACRAKNEWLET---HTEASINELFEQRQQLEDIVTPIFT  238
              LL E   T         +   R   +  L T    H +  IN    E     L  +   P F
Sbjct  159  KPLLPERAPTHPLPHLDAFIRGARKHDPNLRTAVVHIQKIIN---EAPMDLPAMDWPAFQ  215

Query  239  KMTT  242
             MTT
Sbjct  216  AMTT  219
```

>**ref|XP_001088146.1|** UG PREDICTED: similar to ankyrin repeat domain 28 isoform 2 [Macaca mulatta]
Length=640

**GENE ID: 696592 LOC696592** | similar to ankyrin repeat domain 28
[Macaca mulatta]

```
 Score = 52.8 bits (125),  Expect = 4e-05, Method: Compositional matrix adjust.
 Identities = 40/135 (29%), Positives = 58/135 (42%), Gaps = 7/135 (5%)

Query  38   PLLQPALTGDVEGLQKIFEDPENPHHEQAMQLLLEE------DIVGRNLLYAACMAGQSD  91
            PLL      D  G    +      N H E    LL +        D   R  L+ A   G  D
Sbjct  128  PLLSSVNVSDRGGRTALHHAALNGHVEMVNLLLAKGANINAFDKKDRRALHWAAYMGHLD  187

Query  92   VIRALAKYGVNLNEKTTRGYTLLHCAAAWGRLETLKALVELDVDIEALNFREERARDVAA  151
            V+  L  +G  +  K  +GYT LH AA+ G++  +K L+ L V+I+ +N      A  +A
Sbjct  188  VVALLINHGAEVTCKDKKGYTPLHAAASNGQINVVKHLLNLGVEIDEINVYGNTALHIAC  247

Query  152  RYSQTECV-EFLDWA  165
               Q   V E +D+
Sbjct  248  YNGQDAVVNELIDYG  262


 Score = 43.9 bits (102),  Expect = 0.018, Method: Compositional matrix adjust.
 Identities = 33/104 (31%), Positives = 51/104 (49%), Gaps = 16/104 (15%)

Query  72   EEDIVGRNLLYAACMAGQSDVIRALAKYGVNLNEKTTRGYTLLHCAAA--WGRLETLKAL  129
            E ++ G   L+ AC   GQ   V+   L   YG N+N+     G+T LH AAA     G L  L+ L
Sbjct  234  EINVYGNTALHIACYNGQDAVVNELIDYGANVNQPNNNGFTPLHFAAASTHGAL-CLELL  292

Query  130  VE--LDVDIE-----------ALNFREERARDVAARYSQTECVE  160
            V    DV+I+            A++ R  R++ +      + +CV+
Sbjct  293  VNNGADVNIQSKDGKSPLHMTAVHGRFTRSQTLIQNGGEIDCVD  336


 Score = 35.4 bits (80),  Expect = 5.4, Method: Compositional matrix adjust.
 Identities = 32/125 (25%), Positives = 53/125 (42%), Gaps = 9/125 (7%)

Query  38   PLLQPALTGDVEGLQKIFEDPENPHHEQAMQLLLEEDIVGRNLLYAACMAGQSDVIRALA  97
            PL+Q    +GD E ++ +     E+       +  L  E     R  L+ A    G +++I  L
Sbjct  11   PLVQAIFSGDPEEIRMLIHKTED------VNTLDSEK---RTPLHVAAFLGDAEIIELLI  61

Query  98   KYGVNLNEKTTRGYTLLHCAAAWGRLETLKALVELDVDIEALNFREERARDVAARYSQTE  157
              G  +N K      T LH A A    E ++ L++    D+ A +    +    VAA      +
Sbjct  62   LSGARVNAKDNMWLTPLHRAVASRSEEAVQVLIKHSADVNARDKNWQTPLHVAAANKAVK  121

Query  158  CVEFL  162
            C E +
Sbjct  122  CAEVI  126
```

>**dbj|BAE28015.1|** G unnamed protein product [Mus musculus]
Length=1878

**GENE ID: 11733 Ank1** | ankyrin 1, erythroid [Mus musculus]
(Over 10 PubMed links)

```
 Score = 52.8 bits (125),  Expect = 4e-05, Method: Compositional matrix adjust.
 Identities = 30/88 (34%), Positives = 49/88 (55%), Gaps = 6/88 (6%)

Query  50   GLQKIFEDPENPHHEQAMQLLLEEDIV------GRNLLYAACMAGQSDVIRALAKYGVNL  103
            GL   +     H +   ++LL +E I+        G    L+ A +AGQ +V+R L   YG N+
Sbjct  49   GLNGLHLASKEGHVKMVVELLHKEIILETTTKKGNTALHIAALAGQDEVVRELVNYGANV  108

Query  104  NEKTTRGYTLLHCAAAWGRLETLKALVE  131
            N ++ +G+T L+ AA    LE +K L+E
Sbjct  109  NAQSQKGFTPLYMAAQENHLEVVKFLLE  136


 Score = 44.3 bits (103),  Expect = 0.013, Method: Compositional matrix adjust.
 Identities = 29/107 (27%), Positives = 48/107 (44%), Gaps = 7/107 (6%)

Query  63   HEQAMQLLLEED-------IVGRNLLYAACMAGQSDVIRALAKYGVNLNEKTTRGYTLLH  115
            H +   ++LL++        + G    L+ AC       V+   L K G +++   T   G T LH
Sbjct  354  HHRVAKVLLDKGAKPNSRALNGFTPLHIACKKNHIRVMELLLKTGASIDAVTESGLTPLH  413

Query  116  CAAAWGRLETLKALVELDVDIEALNFREERARDVAARYSQTECVEFL  162
             A+   G L   +K L++         N + E     +AAR    TE   ++L
Sbjct  414  VASFMGHLPIVKNLLQRGASPNVSNVKVETPLHMAARAGHTEVAKYL  460


 Score = 43.5 bits (101),  Expect = 0.020, Method: Compositional matrix adjust.
 Identities = 27/87 (31%), Positives = 43/87 (49%), Gaps = 0/87 (0%)

Query  81   LYAACMAGQSDVIRALAKYGVNLNEKTTRGYTLLHCAAAWGRLETLKALVELDVDIEALN  140
            L+ A    G + +++ L + G + N   TT G+T LH AA  G ++T   AL+E +      +
Sbjct  478  LHCAARIGHTGMVKLLLENGASPNLATTAGHTPLHTAAREGHVDTALALLEKEASQACMT  537

Query  141  FREERARDVAARYSQTECVEFLDWADA  167
             +        VAA+Y +     E L    DA
Sbjct  538  KKGFTPLHVAAKYGKVRLAELLLEHDA  564


 Score = 42.4 bits (98),  Expect = 0.053, Method: Compositional matrix adjust.
 Identities = 25/86 (29%), Positives = 39/86 (45%), Gaps = 0/86 (0%)
```

```
Query  77   GRNLLYAACMAGQSDVIRALAKYGVNLNEKTTRGYTLLHCAAAWGRLETLKALVELDVDI  136
            G   L+     G   V   L K+GV ++  T  GYT LH A+ +G ++ +K L++   D+
Sbjct  672  GLTPLHLVSQEGHVPVADVLIKHGVTVDATTRMGYTPLHVASHYGNIKLVKFLLQHQADV  731

Query  137  EALNFREERARDVAARYSQTECVEFL  162
             A           AA+   T+ V  L
Sbjct  732  NAKTKLGYSPLHQAAQQGHTDIVTLL  757


 Score = 35.0 bits (79),  Expect = 7.9, Method: Compositional matrix adjust.
 Identities = 28/102 (27%), Positives = 46/102 (45%), Gaps = 7/102 (6%)

Query  68   QLLLEEDI----VGRN---LLYAACMAGQSDVIRALAKYGVNLNEKTTRGYTLLHCAAAW  120
            +LLLE D      G+N     L+ A      D+++ L   G + +     GYT LH AA
Sbjct  557  ELLLEHDAHPNAAGKNGLTPLHVAVHHNNLDIVKLLLPRGGSPHSPAWNGYTPLHIAAKQ  616

Query  121  GRLETLKALVELDVDIEALNFREERARDVAARYSQTECVEFL  162
             ++E  ++L++      A + +      +AA+   TE V  L
Sbjct  617  NQIEVARSLLQYGGSANAESVQGVTPLHLAAQEGHTEMVALL  658


 Score = 35.0 bits (79),  Expect = 8.7, Method: Compositional matrix adjust.
 Identities = 28/107 (26%), Positives = 44/107 (41%), Gaps = 7/107 (6%)

Query  63   HEQAMQLLLEED-------IVGRNLLYAACMAGQSDVIRALAKYGVNLNEKTTRGYTLLH  115
            H   ++LLLE           G   L+ A   G  D   AL +   +    T +G+T LH
Sbjct  486  HTGMVKLLLENGASPNLATTAGHTPLHTAAREGHVDTALALLEKEASQACMTKKGFTPLH  545

Query  116  CAAAWGRLETLKALVELDVDIEALNFREERARDVAARYSQTECVEFL  162
             AA +G++   + L+E D    A          VA  ++  + V+ L
Sbjct  546  VAAKYGKVRLAELLLEHDAHPNAAGKNGLTPLHVAVHHNNLDIVKLL  592
```

>**dbj|BAC97904.1|** mKIAA0229 protein [Mus musculus]
**gb|EDL22557.1|** ankyrin repeat and SAM domain containing 1, isoform CRA_b [Mus musculus]
Length=1198

**GENE ID: 224650 Anks1** | ankyrin repeat and SAM domain containing 1
[Mus musculus] (Over 10 PubMed links)

```
 Score = 52.8 bits (125),  Expect = 4e-05, Method: Compositional matrix adjust.
 Identities = 37/117 (31%), Positives = 55/117 (47%), Gaps = 10/117 (8%)

Query  63   HEQAMQLLLEEDIV-------GRNLLYAACMAGQSDVIRALAKYG---VNLNEKTTRGYT  112
            H   +++LL  D +       G   L+ A   G + ++R L + G     +NE+     T
Sbjct  118  HRDVVEVLLRNDALTNVADSKGCYPLHLAAWKGDAQIVRLLIQQGPSHTRVNEQNNDNET  177

Query  113  LLHCAAAWGRLETLKALVELDVDIEALNFREERARDVAARYSQTECVEFLDWADARL  169
             LHCAA +G  E +KAL+E   D    N + E   D+AA Y + E V+ L  A   L
Sbjct  178  ALHCAAQYGHTEVVKALLEELTDPTMRNNKFETPLDLAALYGRLEVVKLLLGAHPNL  234


 Score = 36.6 bits (83),  Expect = 2.5, Method: Compositional matrix adjust.
 Identities = 34/119 (28%), Positives = 58/119 (48%), Gaps = 12/119 (10%)

Query  63   HEQAMQLLLEE--DIVGRN-----LLYAACMAGQSDVIRALAKYGVNLNEKTTRGYTLLH  115
            H + ++ LLEE  D   RN      L  A + G+ +V++ L     NL   +TR +T LH
Sbjct  187  HTEVVKALLEELTDPTMRNNKFETPLDLAALYGRLEVVKLLLGAHPNLLSCSTRKHTPLH  246

Query  116  CAAAWGRLETLKALVELDVDIEALNFREERARDV--AARYSQTECVEFLDWADARLTLK  172
             AA  G    ++ L++  +D    N++ E    +  AA + +T+ V+ L  A   + +K
Sbjct  247  LAARNGHKAVVQVLLDAGMDS---NYQTEMGSALHEAALFGKTDVVQILLAAGIDVNIK  302


 Score = 36.6 bits (83),  Expect = 2.9, Method: Compositional matrix adjust.
 Identities = 30/125 (24%), Positives = 58/125 (46%), Gaps = 6/125 (4%)

Query  38   PLLQPALTGDVEGLQKIFEDPENPHHEQAMQLLLEEDIVGRNLLYAACMAGQSDVIRALA  97
            PL   A  GD + ++ + +    P H +      E++      L+ A   G ++V++AL
Sbjct  142  PLHLAAWKGDAQIVRLLIQ--QGPSHTRVN----EQNNDNETALHCAAQYGHTEVVKALL  195

Query  98   KYGVNLNEKTTRGYTLLHCAAAWGRLETLKALVELDVDIEALNFREERARDVAARYSQTE  157
            +   +   +  +  T L  AA +GRLE +K L+    ++ + + R+     +AAR
Sbjct  196  EELTDPTMRNNKFETPLDLAALYGRLEVVKLLLGAHPNLLSCSTRKHTPLHLAARNGHKA  255

Query  158  CVEFL  162
             V+ L
Sbjct  256  VVQVL  260
```

>**gb|AAI71944.1|** Ank1 protein [Mus musculus]
**gb|AAI38030.1|** Ank1 protein [Mus musculus]
Length=1852

**GENE ID: 11733 Ank1** | ankyrin 1, erythroid [Mus musculus]
(Over 10 PubMed links)

```
 Score = 52.8 bits (125),  Expect = 4e-05, Method: Compositional matrix adjust.
 Identities = 30/88 (34%), Positives = 49/88 (55%), Gaps = 6/88 (6%)

Query  50   GLQKIFEDPENPHHEQAMQLLLEEDIV------GRNLLYAACMAGQSDVIRALAKYGVNL  103
            GL  +    +  H +  ++LL +E I+      G    L+ A +AGQ +V+R L  YG N+
Sbjct  49   GLNGLHLASKEGHVKMVVELLHKEIILETTTKKGNTALHIAALAGQDEVVRELVNYGANV  108

Query  104  NEKTTRGYTLLHCAAAWGRLETLKALVE  131
            N ++ +G+T L+ AA    LE +K L+E
Sbjct  109  NAQSQKGFTPLYMAAQENHLEVVKFLLE  136
```

Select All Get selected sequences Distance tree of results Multiple alignment NEW
