# Supplementary figures and images for: Promoter Complexity and Tissue-Specific Expression of Stress Response Components in Mytilus galloprovincialis, a Sessile Marine Invertebrate Species
Source: PLoS Comput Biol. 2010 Jul 8;6(7):e1000847. doi: 10.1371/journal.pcbi.1000847 (PMC2900285; doi:10.1371/journal.pcbi.1000847)

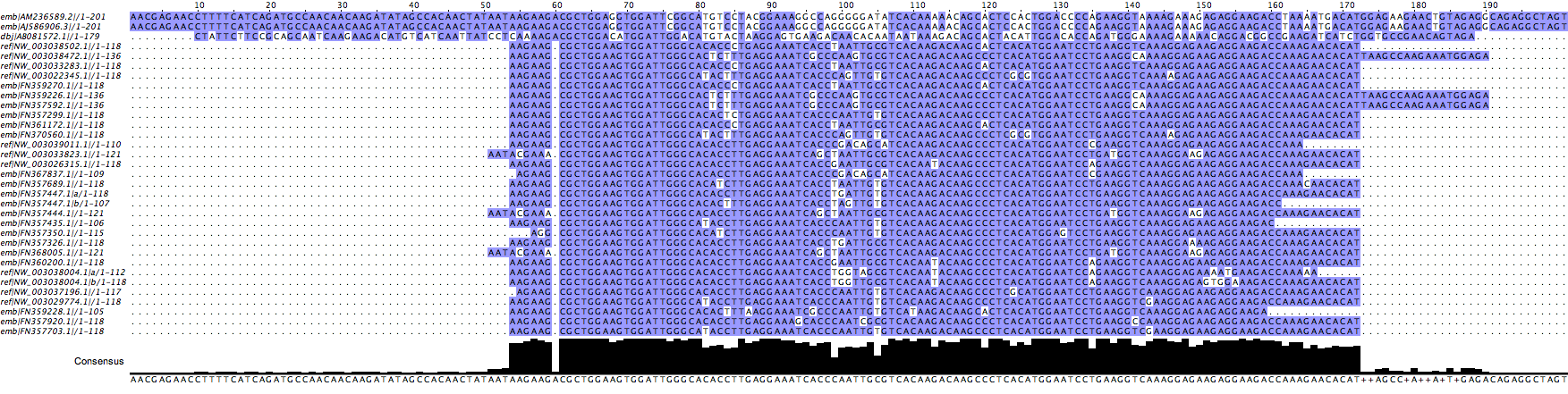

Supplement: Protocol S1 — 18 Supplement files plus an index file: 3 Supplementary figures, 2 Supplementary tables - referenced in text as Protocol S1; index provided with an explanation of the directory contents. (5.18 MB ZIP) [file pcbi.1000847.s001.zip › SUPPLEMENTS18/SupplFigure2.1c.png]

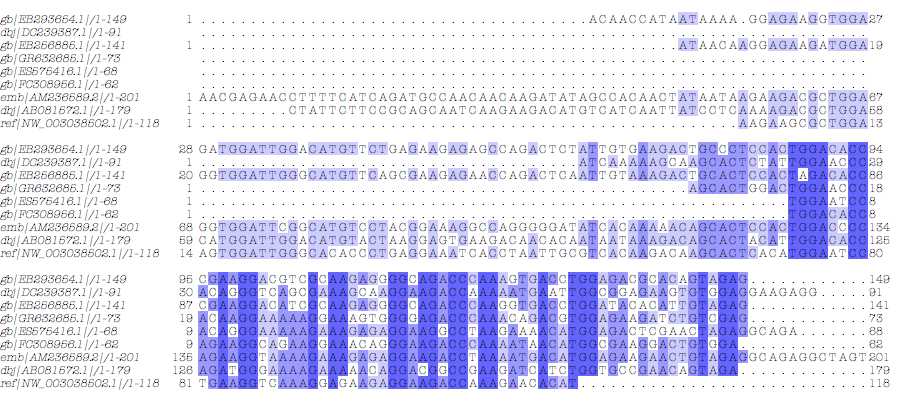

Supplement: Protocol S1 — 18 Supplement files plus an index file: 3 Supplementary figures, 2 Supplementary tables - referenced in text as Protocol S1; index provided with an explanation of the directory contents. (5.18 MB ZIP) [file pcbi.1000847.s001.zip › SUPPLEMENTS18/SupplFigure2.2c.png]
